# SMART-Guided Isolation and Identification of Seven-Membered Cembranolides with Anti-Inflammation Activity from the Soft Coral *Sinularia mollis*

**DOI:** 10.3390/md23120465

**Published:** 2025-12-05

**Authors:** Huiyue Hou, Pinglin Li

**Affiliations:** 1 Key Laboratory of Marine Drugs, Chinese Ministry of Education, School of Medicine and Pharmacy, Ocean University of China, Qingdao 266003, China; aranah@163.com; 2 Laboratory of Marine Drugs and Biological Products, National Laboratory for Marine Science and Technology, Qingdao 266235, China

**Keywords:** *Sinularia mollis*, seven-membered cembranolides, SMART, anti-inflammatory

## Abstract

The first systematic chemical investigation on *Sinularia mollis* resulted in the isolation and identification of 36 seven-membered cembranolides, including 14 new compounds named sinumollolides A–N (**1**–**14**) and 22 known analogs (**15**–**36**) by HSQC-based small molecule accurate recognition technology (SMART). Their structures were characterized by spectroscopic methods (1D/2D NMR and UV), HRESIMS, quantum chemical calculations (DP4+ analysis and ECD calculations), and X-ray diffraction analysis. In zebrafish assays, compounds **1**, **2**, **4**, and **5** exhibited anti-inflammatory activity at 20 μM by inhibiting the number of macrophages around the neuromasts, with inhibition rates ranging from 30.4% to 45.6%. Moreover, the two most bioactive and less toxic compounds, **1** and **5,** featuring a 14-membered macrocyclic lactone scaffold with several hydroxyl groups and a seven-membered *α*, *β*-unsaturated lactone moiety, can inhibit inflammation by suppressing the secretion of inflammatory cytokines at 10 μM in LPS-stimulated BV-2 cells.

## 1. Introduction

Soft corals of the genus *Sinularia* (family Alcyoniidae), widely distribute from the waters of East Africa to the Western Pacific, comprise approximately 150 species and over 70 of these species have been chemically investigated [1]. Diterpenes of the cembrane type are some of the most frequent secondary metabolites and most structurally diverse class from various *Sinularia* species [2]. The cembrane-type diterpenes feature a core 14-membered carbocyclic skeleton, typically featuring an isopropyl group at C-1 and three methyl groups at the C-4, C-8, and C-12 positions, and are categorized into several subtypes, such as isopropyl cembranes, isopropenyl cembranes, and cembranolides by enzymatic processes [3,4]. Research on cembranes has garnered considerable attention due to the wide range of biological activities, including anti-inflammatory activity [5,6], anti-cancer activity [7], cytotoxic activity [8], anti-fouling activity [9], antioxidant activity [10], and anti-hepatitis B virus activity [11].

Particularly, in those subtypes of cembranes, cembranolides are a series of diverse and complex cembrane-type diterpenoids with a typical 14-membered carbocyclic skeleton fused with a five-, six-, or seven-membered lactone moiety bearing either an unsaturation or an exo-double bond, and they demonstrate numerous biological activities, especially anti-inflammatory activities [12]. Notably, extensive research on five-membered cembranolides has illustrated key anti-inflammatory functional groups like the 3,4-epoxy functionality, *β*-hydroperoxyl group at C-7, the acetoxy group at C-13, and the acetoxy group at C-18 [2] For instance, five-membered cembranolide sinularolide F showed potential anti-inflammatory activities against LPS-stimulated RAW 264.7 cells [13]. While six- and seven-membered cembranolides have been reported to exhibit anti-inflammatory activity, research on them remains relatively insufficient; only no more than 20 six- and seven-membered cembranolides have been reported to exhibit anti-inflammatory activity in previous studies [2]. For example, flexibilin D, a six-membered cembranolide, was found to reduce the levels of iNOS and COX-2 to 19.27 ± 2.72% and 30.08 ± 9.07% at 20 μM, respectively [14]. Seven-membered cembranolide sinusiaeolide A showed significant inhibitory effects on IL-1*β* and IL-6 in RAW 264.7 cells [15]. Recent studies on the structure–activity relationship (SAR) of seven-membered cembranolides have indicated that a seven-membered lactone moiety at C-1 is essential for their anti-inflammatory activity [16,17]. The compounds, 11-epi-sinulariolide acetate and 5-dehydrosinulariolide, can inhibit inflammation by suppressing NF-*κ*B and MAPK signaling pathways, and reduce the secretion of inflammatory cytokines [17]. Although five-membered cembranolides have been reported to exhibit anti-inflammatory activity, studies on six- and seven-membered cembranolides and their structure–activity relationships remain relatively limited. Therefore, we employed the SMART technique to guide the isolation of six- and seven-membered cembranolides, aiming to discover anti-inflammatory cembranolide compounds and analyze their structure–activity relationships, thereby providing references for subsequent research.

SMART, the artificial intelligence tool, utilizes a Siamese architecture based on convolutional neural networks and employs Squeeze-Net to map ^1^H-^13^C HSQC spectra into a 180-dimensional embedding space. The algorithm was trained on 53,076 HSQC spectra of natural products, including 25,434 experimental and 27,642 predicted spectra, enabling structural annotation of unknown compounds. Compared to conventional methods, SMART-guided screening and isolation significantly shorten experimental cycles and enable targeted isolation of known structures. The entire workflow, from NMR data acquisition to structural prediction, is completed within 30 min, with structural prediction taking only about 8 s [18]. This tool facilitates the rapid mining of targeted compounds, making it invaluable for screening and prioritizing samples [19,20,21].

**Figure 1:**
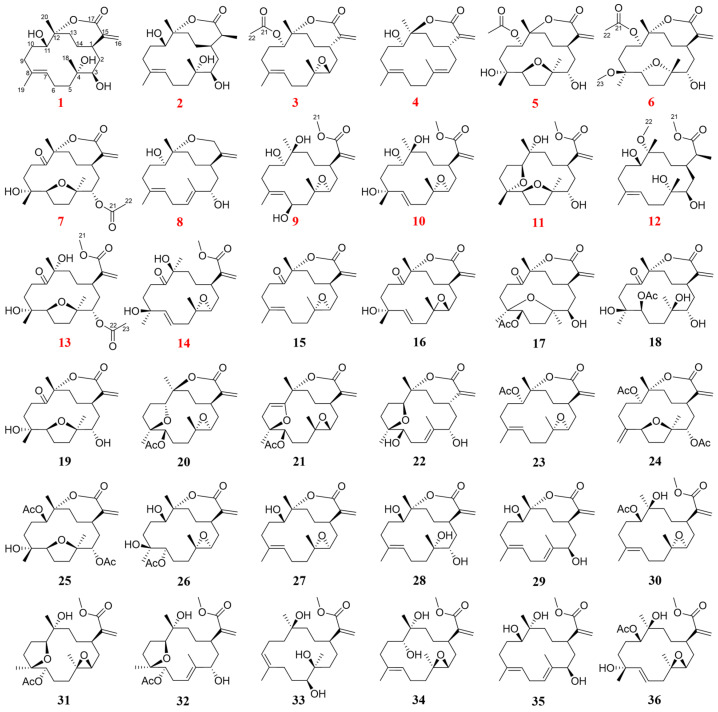
Structures of compounds **1**−**36** (red numbers represent new compounds).

Herein, to streamline the isolation process and achieve the targeted isolation of seven-membered cembranolides, the soft coral *S. mollis* collected from the South China Sea was subjected to its first chemical investigation using an HSQC-based SMART-guided strategy. A total of 36 cembrane-type diterpenoids were isolated and structurally identified, including 22 seven-membered cembranolides and 14 related analogs (Figure 1). Furthermore, we evaluated their anti-inflammatory activities using zebrafish assays, and we elucidated their potential anti-inflammatory effects in LPS-induced BV-2 microglial cells.

## 2. Results and Discussion

### 2.1. SMART-Guided Isolation

The methanolic extract of *S. mollis* was fractionated into 14 primary fractions (Frs. 1–14). These fractions were subjected to HSQC analysis, and the resulting data were uploaded to the SMART platform to assess their chemical profiles. The analysis revealed that Frs. 6, 7, 9, 10, and 13 were correlated with cembranolides. Consequently, five fractions were prioritized for further chromatographic separation, leading to the successful isolation of 23 cembranolides, one cembranoid with a seven-membered ether ring, and 12 related analogs. In contrast to traditional separation strategies that necessitate the extensive screening of every fraction, the SMART method rapidly identified five specific fractions rich in seven-membered cembranolides from the fourteen primary fractions. By offering accurate structural predictions, this approach significantly minimized redundancy and effectively guided the subsequent chromatographic separation process.

### 2.2. Structure Elucidation

**Figure 2:**
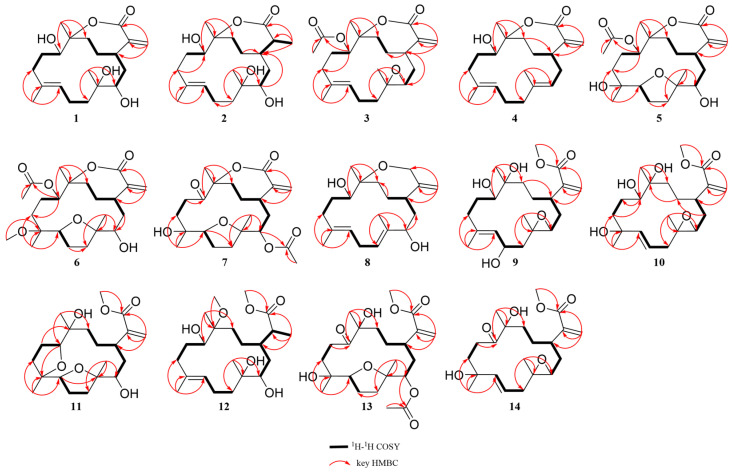
Key ^1^H-^1^H COSY and HMBC correlations of **1**–**14**.

**Sinumollolide A (1)** had a molecular formula of C_20_H_32_O_5_, determined by the HRESIMS [M + H]^+^ ion at *m*/*z* 353.2321 (calcd for C_20_H_33_O_5_, *m*/*z* 353.2323), and it showed five degrees of unsaturation. The ^1^H NMR (Table 1) spectrum showed the presence of three olefinic protons at *δ*_H_ 6.34 (1H, s, H-16a), *δ*_H_ 5.85 (1H, s, H-16b), and *δ*_H_ 5.09 (1H, d, H-7); two oxygenated protons at *δ*_H_ 4.16 (1H, d, H-11) and *δ*_H_ 3.27 (1H, d, H-3); and three methyl groups at *δ*_H_ 1.29 (3H, s, H-20), *δ*_H_ 1.49 (3H, s, H-19), and *δ*_H_ 1.21 (3H, s, H-18). The ^13^C NMR (Table 2) and HSQC spectral data revealed the presence of 20 carbon resonances, divided into five non-protonated carbons (*δ*_C_ 172.0, 143.6, 136.2, 89.4, and 75.9), four methine carbons (*δ*_C_ 128.7, 74.0, 68.7, and 38.5), eight methylene carbons (*δ*_C_ 126.0, 39.8, 37.8, 37.2, 32.8, 31.3, 29.1, and 23.4), and three methyl carbons (*δ*_C_ 24.1, 23.3, and 15.8). The presence of two double bonds in **1** was evident from the ^1^H NMR signals at *δ*_H_ 5.09 and *δ*_H_ 6.34, 5.85 (H-16a/b), as well as the ^13^C NMR signals at *δ*_C_ 136.2 (C-8), 128.7 (C-7), 143.6 (C-15), and 126.0 (C-16). The HMBC correlations (Figure 2) from H-16 to C-1, C-15, and C-17; from H-20 to C-13, C-12, and C-11; from H-19 to C-7, C-8, and C-9; and from H-18 to C-3, C-4, and C-5 combined with the ^1^H–^1^H COSY correlations: H-3/H-2/H-1/H-14/H-13, H-9/H-10/H-11, and H-5/H-6/H-7 established a cembrane skeleton. Three of the five degrees of unsaturation were accounted for by two olefinic bonds and one ester carbonyl group (*δ*_C_ 172.0, C-17), indicating that **1** must be a bicyclic compound. The *α*, *β*-unsaturated lactone substructure was confirmed by the HMBC correlations of H-16a/b to C-1, C-15, and C-17 combined with the molecular formula. Additionally, the resonance of C-12 at *δ*_C_ 89.4 (oxygenated quaternary carbon) indicated that **1** was a cembranolide. Thus, the planar structure of **1** was established (Figure 1).

**Figure 3:**
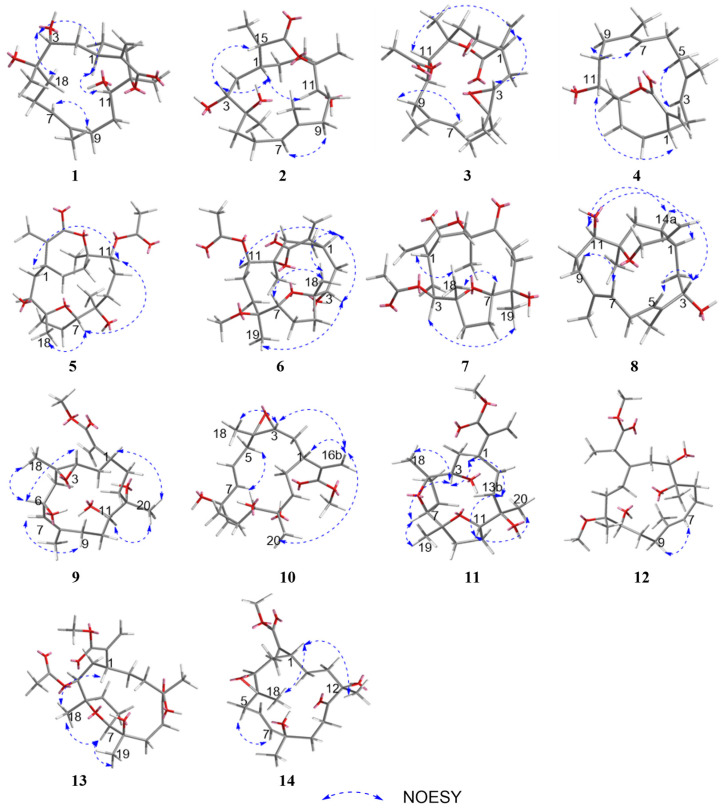
Key NOESY correlations of **1**–**14**.

**Figure 4:**
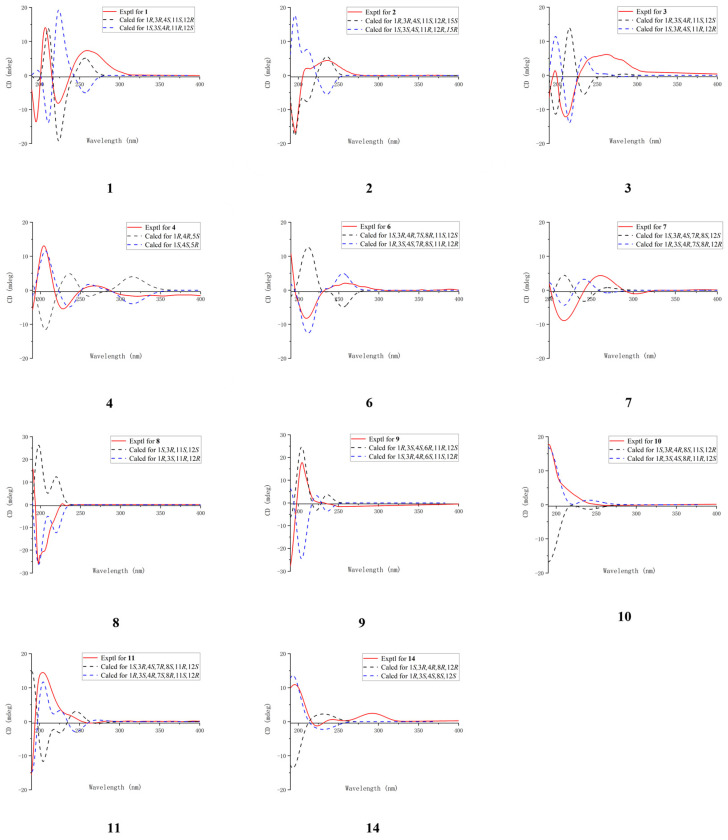
Comparison of the experimental and calculated ECD spectra of compounds **1**−**4**, **6**−**11**, and **14**.

**Figure 5:**
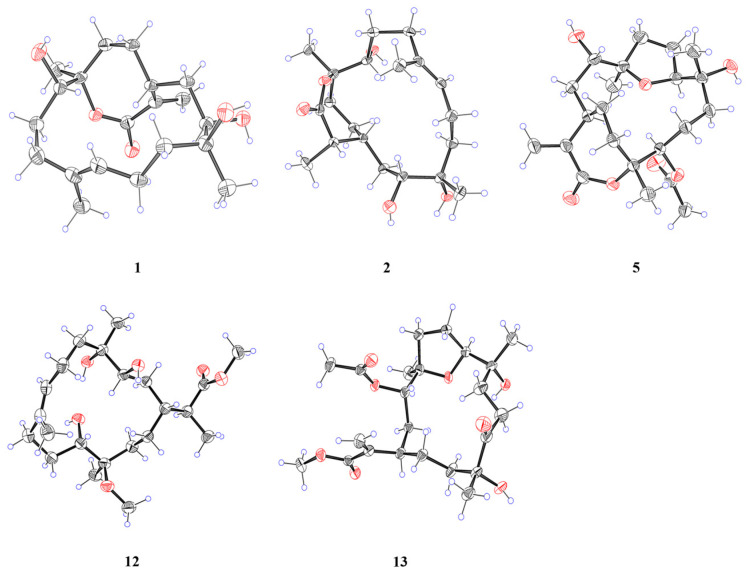
X-ray crystal structures of **1**, **2**, **5**, **12**, and **13**.

The configuration of the double bond was assigned as *E* based on the NOESY correlations between H-9b and H-7 (Figure 3). The NOESY correlations of H-1/H-11, H-3/H-18, and H-3/H-1 implied that H-1, H-3, H-11, and H-18 were on the same side of the molecule. To confirm the holistic relative stereochemistry of **1**, the GIAO method was employed at the PCM/B3LYP/6-311+G (d, p) level was employed to calculate the ^1^H and ^13^C NMR chemical shifts for two candidate configurations: **1a** (1R*, 3R*, 4S*, 11S*, 12R*) and **1b** (1R*, 3R*, 4S*, 11S*, 12S*). The DP4+ probability analyses were performed, which indicated the **1a** configurations. Furthermore, the absolute configuration of 1*R*, 3*R*, 4*S*, 11*S*, 12*R* was suggested by electronic circular dichroism (ECD) calculations performed using the time-dependent density functional theory (TDDFT) method at the RB3LYP/DGDZVP level (Figure 4). The absolute configuration of **1** was further confirmed by single-crystal X-ray diffraction analysis as 1*R*, 3*R*, 4*S*, 11*S*, 12*R*. (Figure 5). These results show that the application of DP4+ is reliable for the stereochemical elucidation of most compounds.

Analysis of the 1D NMR and 2D NMR (Table 1 and Table 2 and Figure 2) data, along with UV and HRESIMS, revealed that **sinumollolide B (2)** possesses the same carbon skeleton as **1**. A significant difference between **1** and **2** was the reduced double bond at *Δ*^15,16^, which was further confirmed by the HMBC (Figure 2) correlations from H-16 to C-1, C-15, and C-17, as well as the ^1^H−^1^H COSY correlations of H-16/H-15/H-1. The NOESY (Figure 3) correlations of H-9/H-7, H-3/H-1/H-15, and H-11/H-1 indicated the 8*E*, 1R*, 3R*, 11S*, 15S* configuration, but the chirality of C-4 and C-12 could not be elucidated from NOESY data. Accordingly, NMR shifts and DP4+ probability analysis were calculated for four possible relative configurations, **2a** (1R*, 3R*, 4R*, 11S*, 12R*, 15S*), **2b** (1R*, 3R*, 4R*, 11S*, 12S*, 15S*), **2c** (1R*, 3R*, 4S*, 11S*, 12R*, 15S*), and **2d** (1R*, 3R*, 4S*, 11S*, 12S*, 15S*), and the results showed that **2c** was the best fit. The absolute configuration of **2** was determined as 1*R*, 3*R*, 4*S*, 11*S*, 12*R*, 15*S* by ECD calculations (Figure 4) and single crystal X-ray diffraction (Figure 5).

The ^1^H and ^13^C NMR data (Table 1 and Table 2) displayed close similarity between **sinumollolide C (3)** and **1**. The most pronounced differences were the presence of an acetyl group in **3** at C-11 and an oxygen bridge between C-3 and C-4, as verified by HMBC correlations from H-22 to C-21, from H-11 to C-21, and the key chemical shift observed for C-4 and C-3 in comparison with the known compound **15**. In the NOESY (Figure 3) spectrum of **3**, the *E*-configuration of *Δ*^7,8^ was confirmed by the correlation between H-7 and H-9b. The relative configuration of **3** was based on NOESY correlations of H-3/H-1 and H-11/H-1, suggested the **3a** (1R*, 3S*, 4R*, 11S*, 12S*), **3b** (1R*, 3S*, 4S*, 11S*, 12S*), **3c** (1R*, 3S*, 4R*, 11S*, 12R*), and **3d** (1R*, 3S*, 4S*, 11S*, 12R*) configurations. The relative configuration of the C-4 and C-12 chiral centers in **3** was determined by calculating the above four relative configurations. Ultimately, the absolute configuration of **3** was suggested by DP4+ analysis (100% for **3a**) combined with ECD calculations as 1*S*, 3*R*, 4*S*, 11*R*, 12*R* (Figure 4).

The carbon skeleton of **sinumollolide D (4)** was readily assignable as the same as **1** by 1D NMR, HSQC, HMBC, and COSY correlations. One difference was the presence of an additional olefinic bond (δ_C_ 136.6 and 125.0) at *Δ*^3,4^. Further confirmation of this new bond came from a key HMBC (Figure 2) correlation between H-18, C-3, and C-4. The configurations of the *Δ*^3,4^ and the *Δ*^7,8^ double bonds were assigned as *E* based on the NOESY (Figure 3) correlations from H-9a to H-7 and from H-3 to H-5a/b, respectively. Furthermore, the NOESY correlation of H-1/H-11 confirmed the partial relative configuration of 1R*,11S*. The DP4+ analysis deduced the entire relative configuration as 1R*,11S*, 12R*, and the ECD calculation determined the absolute configuration of 1*S*, 11*R*, 12*S* (Figure 4).

**Sinumollolide E (5)** was isolated as a colorless crystal. Similar to compounds **1**–**4**, the analysis of 1D (Table 1 and Table 2) and 2D (Figure 2) NMR correlations from four methyl groups established the consecutive fragment of the classic 12,17-*α*, *β*-unsaturated seven-membered cembranolides skeleton with an acetyl group at C-11, which was indicated by HMBC correlations from H-11 to C-21 and H-22 to C-21. Based on the HRESIMS data, compound **5** had the molecular formula C_22_H_34_O_7_ and six degrees of unsaturation. A tricyclic framework was thus required to account for these after excluding one double bond and two ester carbonyls. The presence of an oxygen bridge between C-4 and C-7 was confirmed by the molecular formula, the remaining one degree of unsaturation, and the resonances of C-7 at *δ*_C_ 85.7 and C-4 at *δ*_C_ 88.9. Therefore, the planar structure of **5** was established. The NOESY spectrum (Figure 3) correlations from H-7 to H-18, H-7 to H-11, and H-11 to H-1 were observed, and the chirality of C-3 and C-12 could not be elucidated. Fortunately, a suitable crystal of **5** was obtained, and its absolute structure (1*R*, 3*S*, 4*R*, 7*S*, 8*R*, 11*R*, 12*R*) was unambiguously determined by single-crystal X-ray diffraction with Cu K*α* radiation (Figure 5).

Analysis of the 1D, 2D NMR data (Table 1 and Table 2 and Figure 2), and HRESIMS revealed that **sinumollolide F (6)** possessed the same carbon skeleton as **5**, except for the presence of a methoxy group in **6**. The methoxy group was placed at C-8 (*δ*_C_ 78.3) because of the clear HMBC signal from C-23 (*δ*_C_ 49.1) to C-8. Additionally, the NOESY correlations from H-1 to H-11, H-1 to H-3, H-7 to H-18, H-1 to H-18, and H-19 to H-3 indicated two possible configurations **6a** (1S*, 3R*, 4R*, 7S*, 8R*, 11S*, 12R*) and **6b** (1S*, 3R*, 4R*, 7S*, 8R*, 11S*, 12S*). The DP4+ analysis deduced the relative configuration as **6b**, and the absolute configuration 1*R*, 3*S*, 4*S*, 7*R*, 8*S*, 11*R*, 12*R* was determined by ECD calculations (Figure 4).

The ^1^H and ^13^C NMR data (Table 1 and Table 2) displayed the close similarity of **sinumollolide G (7)** with **5**. Two major differences were the presence of the acetoxyl group at C-11 in **5** instead of the acetoxyl group at C-3 (*δ*_C_ 76.1) in **7**, which was further confirmed by the HMBC (Figure 2) correlation from H-22 (*δ*_H_ 2.02) to C-21 (*δ*_C_ 170.1) and H-3 (*δ*_H_ 4.72) to C-21, and a keto group at C-11 in **7** instead of the acetoxyl group at C-11 in **5**. The NOESY (Figure 3) correlations from H-1 to H-18, H-7 to H-18, and H-3 to H-19 indicated that there were four possible configurations: **7a** (1S*, 3S*, 4S*, 7R*, 8R*, 12S*), **7b** (1S*, 3R*, 4S*, 7R*, 8S*, 12S*), **7c** (1S*, 3S*, 4S*, 7R*, 8R*, 12S*), and **7d** (1S*, 3R*, 4S*, 7R*, 8S*, 12S*). This was also confirmed by the DP4+ analysis, which supported the **7d**. The absolute configuration of **7** was 1*R*, 3*S*, 4*R*, 7*S*, 8*R*, 12*R*, indicated by ECD calculation (Figure 4).

The 1D and 2D NMR (Table 2 and Table 3 and Figure 2) spectra of **sinumollolide H (8)** were quite similar to **29**, indicating that **8** had an analogous planar structure of **29** except for the presence of a methylene group at C-17 (*δ*_C_ 64.9) in **8** instead of the lactone group in **29**. Compound **8** comprised a 7-membered ether ring, rather than the *α*, *β*-unsaturated lactone found in other related compounds. The NOESY (Figure 3) correlations from H-9a/b to H-7 and H-5b to H-3 indicated both the *E*-configurations of *Δ*^4,5^ and *Δ*^7,8^, while correlations from H-1 to H-11 and H-3 to H-14a, and H-14a to H-11 confirmed the partial relative configurations of 1R*,3S*,11R*,12R*, and 1R*,3S*,11R*,12S*. The DP4+ analysis predicted the relative configuration (12R*) to match the experimental results with 100% probability. The absolute configuration of 1*R*, 3*S*, 11*R*, 12*R* was determined by ECD calculations (Figure 4).

A comparison of the 1D NMR (Table 2 and Table 3) spectra of **sinumollolide I (9)** and **27** revealed similar structural relationships. The most notable difference was the presence of a hydroxyl group at C-6 (*δ*_C_ 65.7) in **9** and that the seven-membered lactone ring of **27** underwent ring-opening, resulting in a quaternary carbon at C-12 (*δ*_C_ 74.3) with a hydroxyl substitution and a methoxy group connected to C-17, indicated by HMBC correlations from H-21 to C-17. To determine the relative configuration of **9**, the NOESY signals from H-7 to H-9b indicated that the *Δ*^7,8^ double bond had an *E* configuration, as well as the related signals from H-1 to H-11, H-11 to H-20, H-6 to H-3, and H-6 to H-18. Thus, two possible configurations, **9a** (1R*, 3S*, 4S*, 6R*, 11R*, 12S*) and **9b** (1S*, 3S*, 4S*, 6R*, 11S*, 12R*), were calculated and **9a** showed the best result. Based on the ECD analysis, the structure and absolute configuration of **9** were tentatively determined to be 1*R*, 3*S*, 4*S*, 6*R*, 11*R*, 12*S* (Figure 4).

Analysis of the 1D NMR (Table 2 and Table 3) data, 2D NMR (Figure 2) data, and HRESIMS revealed that **sinumollolide J (10)** possessed the same carbon skeleton as **9**, with the main differences observed in the 2D NMR data for C-6, C-7, and C-8. This suggested that the trisubstituted double bond *Δ*^7,8^ and the hydroxyl group at C-6 in **9** were replaced by the disubstituted double bond *Δ*^6,7^ and a hydroxyl group at C-8 (*δ*_C_ 84.3) in **10**, as indicated by the HMBC correlations from H-18 to C-7, C-8, and C-9. The NOESY experiment (Figure 3) of **10** showed correlations from H-7 to H-5, which indicated the *E* geometry of *Δ*^6,7^. Additional NOESY correlations were observed of H-16b to H-3, H-1, and H-20, of H-3 to H-18, and of H-7 to H-5. Thus, two possible configurations, **10a** (1R*, 3R*, 4R*, 8R*, 11S*, 12R*) and **10b** (1R*, 3R*, 4R*, 8S*, 11S*, 12R*) were calculated, and **10b** showed the best result. The absolute configuration of **10** were determined as 1*R*, 3*S*, 4*S*, 8*R*, 11*R*, 12*S* by ECD calculation (Figure 4).

**Sinumollolide K (11)** was a colorless oil. The analysis of the 1D and 2D NMR (Table 2 and Table 3 and Figure 2) data indicated that **11** possessed a similar planar structure to **5**. One difference between **5** and **11** was that the seven-membered lactone ring of **11** underwent ring-opening, resulting in a quaternary carbon at C-12 (*δ*_C_ 75.7) with a hydroxyl substitution and a methoxy group connected to C-17, indicated by HMBC correlation from H-21 to C-17. Another difference between **5** and **11** was the presence of a new five-membered ring provided by the key HMBC correlation from H-11 to C-8. The NOESY correlations from H-1 to H-3, H-3 to H-7, H-7 to H-19, H-3 to H-18, H-11 to H-20, H-11 to H-13a and H-13a to H-1 confirmed the relative configurations of **11**. The absolute configuration of **11** was finally determined to be 1*R*, 3*S*, 4*R*, 7*S*, 8*R*, 11*S*, 12*R* by ECD comparison (Figure 4).

**Sinumollolide L (12)** was isolated as a colorless crystal. The NMR spectra of **12** (Table 2 and Table 3 and Figure 2) showed great similarities to those of the co-isolated compound **2**, with main differences in the ^1^H NMR of *δ*_H_ 3.67 (3H, s) and *δ*_H_ 3.14 (3H, s). The HMBC correlations H-21 (*δ*_H_ 3.67) to C-17 (*δ*_C_ 177.6) and H-22 (*δ*_H_ 3.14) to C-12 (*δ*_C_ 78.5) suggested the opening of the seven-membered lactone ring and two methoxy substitutions. The absolute configuration of **12** was determined as 1*R*, 3*R*, 4*R*, 11*S*, 12*R*, 15*S* by X-ray crystallography (Figure 5).

A comparison of the 1D NMR spectra of **sinumollolide M (13)** with those of **7** (Table 2 and Table 3) revealed a similar structural relationship. The noticeable difference occurred in the opening of the seven-membered lactone ring in **13**. The key HMBC (Figure 2) correlation from H-21 (*δ*_H_ 3.69) to C-17 (*δ*_C_ 166.5) also verified this deduction. Thus, **13** was determined to be the open-loop derivative of **7**. Finally, the absolute configuration of **13** was unambiguously determined as 1*R*, 3*S*, 4*R*, 7*S*, 8*R*, 12*R* (Figure 5) by single-crystal X-ray diffraction with Cu K*α* radiation.

**Sinumollolide N (14)** was isolated as a colorless oil. By comparing the 1D NMR spectra (Table 2 and Table 3), it was found that **14** and **10** had similar planar structures. The molecular formula was elucidated as C_21_H_32_O_6_ based on the HRESIMS data, which was two mass units less than that of **10**. The key HMBC (Figure 2) correlation from H-20 to C-11 also verified that the hydroxyl-substituted C-11 in **10** was oxidized to a carbonyl group in **14**. The NOESY (Figure 3) correlations H-7 to H-5a suggested that the *E* geometry of the double bond *Δ*^6,7^, and the relative configuration of **14,** was determined by the correlations from H-18 to H-1 and H-1 to H-12. To confirm the relative stereochemistry of C-3 and C-8, the computed chemical shifts in the four configurations, **14a** (1S*, 3R*, 4R*, 8R*, 12R*), **14b** (1S*, 3R*, 4R*, 8S*, 12R*), **14c** (1S*, 3S*, 4R*, 8R*, 12R*), and **14d** (1S*, 3S*, 4R*, 8R*, 12R*), were compared with the experimental values, and the results showed that **14a** provided the best fit. The absolute configuration of **14** was finally determined to be 1*R*, 3*S*, 4*S*, 8*S*, 12*S* by comparing the experimental and calculated ECD spectra (Figure 4).

Those known cembranolides diterpenes were identified as 11-dehydrosinulariolide (**15**) [22], Flexibilisolide D (**16**) [23], querciformolide A (**17**) [24], sinulaflexiolide C (**18**) [25], sinulariolone (**19**) [24], 5,8-epoxycembranolide (**20**) [26], flexibilisolide E (**21**) [23], flexibolide (**22**) [26], 11-epi-sinulariolide acetate (**23**) [27], granosolide B (**24**) [24], dendronpholide Q (**25**) [28], sinulariaoid D (**26**) [8], sandensolide (**27**) [29], capillolide (**28**) [30], (−)-sandensolide (**29**) [31], granosolide D (**30**) [32], sinulaflexiolide E (**31**) [25], dendronpholide P (**32**) [28], sinuflexibilin A (**33**) [33], flexibilisin B (**34**) [34], dendronpholide C (**35**) [28], and granosolide C (**36**) [32], by comparing with reported spectroscopic data, respectively.

### 2.3. Biological Activity and SAR

**Figure 6:**
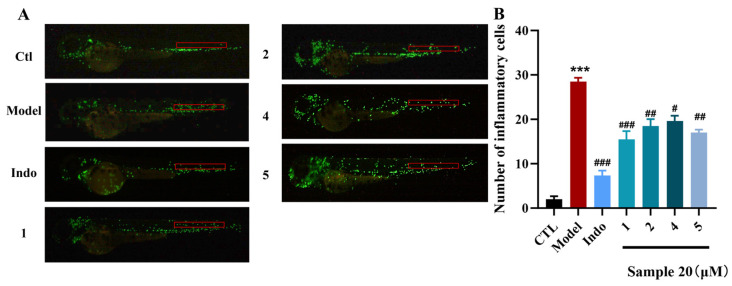
Anti-inflammatory effect of compounds **1**, **2**, **4**, and **5**. (**A**) Inflammatory areas (red box) in transgenic fluorescent zebrafish (Tg: zlyz-EGFP) generated by CuSO_4_ that express enhanced green fluorescent protein after being treated with compounds **1**, **2**, **4**, and **5**. (**B**) Quantitative evaluation of fluorescent macrophage counts in the vicinity of inflammatory sites in zebrafish treated with compounds **1**, **2**, **4**, and **5**. Ordinary one-way ANOVA, *n* = 3 biological replicates. *** *p* ≤ 0.001 vs. CTL, # *p* ≤ 0.05 vs. model, ## *p* ≤ 0.01 vs. model, ### *p* ≤ 0.001 vs. model.

Zebrafish serves as an ideal vertebrate model for neurological research because it exhibits high homology with humans, possesses a nervous system that retains key mammalian characteristics, including conserved brain structure and myelination, and offers a short developmental cycle and a relatively simple neural system for practical experimental advantages [35]. The zebrafish were divided into blank (CTL), model, positive drug (indomethacin), and sample groups. Exposure of model zebrafish to CuSO_4_ resulted in a significant increase in the number of macrophages within the zebrafish neuromast region compared to the control group. The number of macrophages in the neuromast region significantly increased from 2.00 ± 0.68 in the control group to 28.50 ± 0.85 in model zebrafish (*p* < 0.001) (Figure 6A). This finding suggested that initial nerve injury triggers the activation and aggregation of local immune cells, leading to a subsequent neuroinflammatory response. The positive drug group, indomethacin (Indo) at a concentration of 20 μM reduced the number of macrophages in the neuromasts of inflamed zebrafish, with an inhibition rate of 73.9%. After treatment with compounds **1**−**14** at 20 μM, as shown in Figure 6B, compounds **1**, **2**, **4**, and **5** showed moderate anti-inflammatory activity by alleviating migration and decreasing the number of macrophages surrounding the neuromasts in CuSO_4_-induced transgenic fluorescent zebrafish with the inhibition rates 45.6%, 35.1%, 30.4% and 39.7%, respectively. In addition, compounds did not cause obvious developmental abnormalities or death in zebrafish at 20 μM, confirming their low toxicity.

Analysis of the structure-activity relationship (SAR) revealed several key determinants for the bioactivity of these cembranolides. The presence of the seven-membered lactone moiety (**2**, **16**) conferred significantly greater activity than its absence (**12**, **14**), indicating the fragment was essential. Furthermore, the presence of a C-15, C-16 double bond enhanced anti-inflammatory activity, evidenced by the comparison between **1** and **2**. Hydroxyl substitution at the C-3 position is an essential group for activity (**7** vs. **19**). Conversely, in seven-membered cembranolides featuring a C-4 and C-7 furan ring, substitution at the C-8 position with a methoxy group or configuration of C-4, C-7, and C-8 was detrimental, leading to a marked reduction or even a complete loss of activity (**5** vs. **6**).

**Figure 7:**
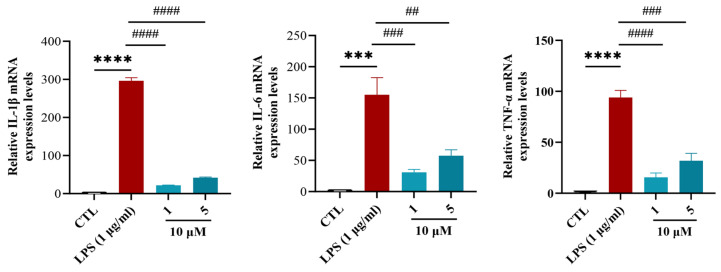
The effects of compounds **1** and **5** on the mRNA levels of IL-1*β*, IL-6, and TNF*-α* in LPS-induced BV-2 cells. Ordinary one-way ANOVA, *n* = 3 biological replicates. *** *p* ≤ 0.001 vs. CTL, **** *p* ≤ 0.0001 vs. CTL, # *p* ≤ 0.05 vs. LPS (1 μg/mL), ## *p* ≤ 0.01 vs. LPS (1 μg/mL), ### *p* ≤ 0.001 vs. LPS (1 μg/mL), #### *p* ≤ 0.0001 vs. LPS (1 μg/mL).

Anti-inflammatory effects were investigated in an LPS-induced BV-2 microglial model to circumvent the confounding influences of peripheral inflammation and complex in vivo cellular interactions. LPS stimulation significantly upregulated the mRNA levels of TNF-*α*, IL-1*β*, and IL-6 in BV-2 cells, indicating the successful activation of microglia and initiation of an inflammatory response [36]. In addition, we used CCK8 to detect whether the cytotoxicity of compounds **1** and **5** had a significant impact on BV-2 cell viability at 10 μM, showing inhibition rates of only 5.6% and 7.4%, respectively. These favorable safety profiles supported their selection for subsequent anti-inflammatory studies. Cells were divided into blank (CTL), LPS-stimulated (1 μg/mL LPS), and sample groups (1 μg/mL LPS + 10μM compounds **1** or **5**) (*n* = 3 biological replicates). In the LPS-treated sample group, treatment with compounds **1** and **5** at 10 μM downregulated the mRNA levels of TNF-*α*, IL-1*β*, and IL-6 compared with the LPS-treated group. Among these, compound **1** exhibited the best inhibitory effect (Figure 7). This demonstrated that the compounds inhibited levels of inflammatory factors and directly inhibited inflammation in microglia.

Compounds **1** and **5** suppressed TNF-α, IL-1*β*, and IL-6 in LPS-stimulated BV-2 cells at 10 μM, with compound **1** showing comparable or superior inhibitory effects. Based on previous studies [17], seven-membered lactone ring cembrane diterpenes, 11-epi-sinulariolide acetate and 5-dehydrosinulariolide, inhibit LPS-induced expression of inflammatory cytokines TNF-*α*, IL-1*β*, and IL-6 in RAW 264.7 macrophages by suppressing the phosphorylation of NF-*κ*B p65, ERK1/2, and JNK3 signaling molecules.

## 3. Materials and Methods

### 3.1. General Experimental Procedures

Thin-layer chromatography (TLC) was performed by pre-coated silica gel plates (GF254, Qingdao, China), and the chromo-genic reagent was EtOH with 5% H_2_SO_4_. Column chromatography (CC) was performed with silica gel (100–400 mesh, Qingdao Marine Chemical Inc., Qingdao, China) and ODS silica gel (50 μm, Merck, Darmstadt, Germany). HPLC was performed on a Waters 2695/2998 instrument with a PDA detector (Milford, Worcester, MA, American), equipped with an analytic reversed-phased column (Silgreen C_18_, 10 × 250 mm, 5 μm, Beijing, China). Chiral separations were performed on the same system using a Daicel Chiral pack IC column (4.6 × 250 mm, 5 μm, Osaka, Japan). NMR spectra were recorded on Bruker AVANCE NEO 400 MHz and Bruker AVANCE NEO 500 MHz (Bruker, Faellanden, Switzerland). HRESIMS data were acquired by a Waters Micro-mass Q-Tof Ultima GLOBAL GAA076 LC mass spectrometer (Autospec-Ultima-TOF, Waters, Shanghai, China). UV and CD spectra were taken on a Jasco J-810 spectropolarimeter (Beckman Ltd., Shanghai, China). Optical rotation spectra were measured by a Jasco P-1020 digital polarimeter (JASCO Corporation, Tokyo, Japan). Single-crystal X-ray diffraction data were collected by a Bruker D8 Venture diffractometer with Cu Kα radiation (Bruker, Beijing, China). Melting points were determined on a SWG X-4A microscopic melting point apparatus (Shanghai Yidian Physical Optics Instrument Co., Ltd., Shanghai, China).

### 3.2. Soft Coral Material

The soft coral *Sinularia mollis* was collected from the Xisha Islands in the South China Sea in 2018. The specimen was identified by Prof. Ping-Jyun Sung of the Institute of Marine Biotechnology, Museum of Marine Biology and Aquarium, Pingtung 944, Taiwan. A voucher specimen (No. XS-ly-39), frozen immediately at −20 °C, was deposited at the School of Medicine and Pharmacy, Ocean University of China, Qingdao, China.

### 3.3. Extraction and Isolation

The fresh specimen of *Sinularia mollis* (7.3 kg, wet weight) was crushed and extracted with MeOH six times (each time for seven days) at room temperature, and then the concentrated residue (107.2 g) was dissolved in MeOH (1 L) to remove salts. The total extract was divided into 14 fractions (Frs. 1–14) by the vacuum liquid chromatography on a silica gel column eluting with a gradient of petroleum ether/acetone (from 50:1 to 1:1, V:V) and CH_2_Cl_2_/MeOH (from 10:1 to 1:1, V:V). See the Supplementary Materials for more details.

Sinumollolide A (**1**): colorless crystal; [*α*${]}_{D}^{25}$ +21.9 (*c* 0.70, CH_3_OH); m. p.: 145.4 °C–149.9 °C; UV (CH_3_OH) *λ*_max_ (log *ε*): 198 (3.1) nm; ^1^H and ^13^C NMR (CD_3_OD) see Table 1 and Table 2; Positive HRESIMS (*m*/*z*): [M + H]^+^ calcd for C_20_H_33_O_5_, 353.2323; found, 353.2321. The crystallographic data have been deposited at the Cambridge Crystallographic Data Center as CCDC 2493949.

Sinumollolide B (**2**): colorless crystals; [*α*${]}_{D}^{25}$ +9.6 (*c* 0.28, CH_3_OH); m. p.: 140.4 °C− 143.6 °C; UV (CH_3_OH) *λ*_max_ (log *ε*): 193 (3.1) nm; ^1^H and ^13^C NMR (CD_3_OD) see Table 1 and Table 2; Positive HRESIMS (*m*/*z*): [M + H]^+^ calcd for C_20_H_35_O_5_, 355.2479; found, 355.2478. The crystallographic data have been deposited at the Cambridge Crystallographic Data Center as CCDC 2493954.

Sinumollolide C (**3**): colorless oil; [*α*${]}_{D}^{25}$ +11.1 (*c* 0.09, CH_3_OH); UV (CH_3_OH) *λ*_max_ (log *ε*): 194 (2.6) nm; ^1^H and ^13^C NMR (CDCl_3_) see Table 1 and Table 2; Positive HRESIMS (*m*/*z*): [M + H]^+^ calcd for C_22_H_33_O_5_, 377.2323; found, 377.2320.

Sinumollolide D (**4**): colorless oil; [*α*${]}_{D}^{25}$ +17.6 (*c* 0.07, CH_3_OH); UV (CH_3_OH) *λ*_max_ (log *ε*): 193 (2.7) nm; ^1^H and ^13^C NMR (CDCl_3_) see Table 1 and Table 2; Positive HRESIMS (*m*/*z*): [M + H]^+^ calcd for C_20_H_31_O_3_, 319.2268; found, 319.2259.

Sinumollolide E (**5**): colorless crystals; [*α*${]}_{D}^{25}$ +6.5 (*c* 0.93, CH_3_OH); m. p.: 208.2 °C–210.3 °C; UV (CH_3_OH) *λ*_max_ (log *ε*): 207 (3.1) nm; ^1^H and ^13^C NMR (CD_3_OD) see Table 1 and Table 2; Positive HRESIMS (*m*/*z*): [M + H]^+^ calcd for C_22_H_35_O_7_, 411.2377; found, 411.2373. The crystallographic data have been deposited at the Cambridge Crystallographic Data Center as CCDC 2493967.

Sinumollolide F (**6**): colorless oil; [*α*${]}_{D}^{25}$ −10.0 (*c* 0.05, CH_3_OH); UV (CH_3_OH) *λ*_max_ (log *ε*): 201 (2.5) nm; ^1^H and ^13^C NMR (CDCl_3_) see Table 1 and Table 2; Positive HRESIMS (*m*/*z*): [M + H]^+^ calcd for C_23_H_37_O_7_, 425.2534; found, 425.2543.

Sinumollolide G (**7**): colorless oil; [*α*${]}_{D}^{25}$ −0.8 (*c* 0.65, CH_3_OH); UV (CH_3_OH) *λ*_max_ (log *ε*): 211 (2.7) nm; ^1^H and ^13^C NMR (CDCl_3_) see Table 1 and Table 2; Positive HRESIMS (*m*/*z*): [M + H]^+^ calcd for C_22_H_33_O_7_, 409.2221; found, 409.2226.

Sinumollolide H (**8**): colorless oil; [*α*${]}_{D}^{25}$ −4.3 (*c* 0.07, CH_3_OH); UV (CH_3_OH) *λ*_max_ (log *ε*): 197 (3.1) nm; ^1^H and ^13^C NMR (CDCl_3_) see Table 2 and Table 3; Positive HRESIMS (*m*/*z*): [M −H_2_O+ H]^+^ calcd for C_20_H_31_O_2_, 303.2319; found, 303.2320.

Sinumollolide I (**9**): colorless oil; [*α*${]}_{D}^{25}$ −9.1 (*c* 0.05, CH_3_OH); UV (CH_3_OH) *λ*_max_ (log *ε*): 197 (3.1) nm; ^1^H and ^13^C NMR (CDCl_3_) see Table 2 and Table 3; Positive HRESIMS (*m*/*z*): [M + H]^+^ calcd for C_21_H_35_O_6_, 383.2428; found, 383.2432.

Sinumollolide J (**10**): colorless oil; [*α*${]}_{D}^{25}$ −3.5 (*c* 0.14, CH_3_OH); UV (CH_3_OH) *λ*_max_ (log *ε*): 194 (2.7) nm; ^1^H and ^13^C NMR (CDCl_3_) see Table 2 and Table 3; Positive HRESIMS (*m*/*z*): [M + Na]^+^ calcd for C_21_H_34_O_6_Na, 405.2248; found, 405.2259.

Sinumollolide K (**11**): colorless oil; [*α*${]}_{D}^{25}$ −1.1 (*c* 0.25, CH_3_OH); UV (CH_3_OH) *λ*_max_ (log *ε*): 196 (3.0) nm; ^1^H and ^13^C NMR (CDCl_3_) see Table 2 and Table 3; Positive HRESIMS (*m*/*z*): [M + H]^+^ calcd for C_21_H_35_O_6_Na, 383.2428; found, 383.2430.

Sinumollolide L (**12**): colorless crystals; [*α*${]}_{D}^{25}$ −1.5 (*c* 0.34, CH_3_OH); m. p.: 143.7 °C–147.2 °C; UV (CH_3_OH) *λ*_max_ (log *ε*): 192 (2.6) nm; ^1^H and ^13^C NMR (CDCl_3_) see Table 2 and Table 3; Positive HRESIMS (*m*/*z*): [M + Na]^+^ calcd for C_22_H_40_O_6_Na, 423.2717; found, 423.2722. The crystallographic data have been deposited at the Cambridge Crystallographic Data Center as CCDC 2493976.

Sinumollolide M (**13**): colorless crystals; [*α*${]}_{D}^{25}$ +8.3 (*c* 0.70, CH_3_OH); m. p.: 158.0 °C–162.1 °C; UV (CH_3_OH) *λ*_max_ (log *ε*): 194 (3.2), 208 (3.1) nm; ^1^H and ^13^C NMR (DMSO-*d*_6_) see Table 2 and Table 3; Positive HRESIMS (*m*/*z*): [M + Na]^+^ calcd for C_23_H_36_O_8_Na, 463.2302; found, 463.2307. The crystallographic data have been deposited at the Cambridge Crystallographic Data Center as CCDC 2493978.

Sinumollolide N (**14**): colorless oil; [*α*${]}_{D}^{25}$ −3.2 (*c* 0.15, CH_3_OH); UV (CH_3_OH) *λ*_max_ (log *ε*): 196 (3.0) nm; ^1^H and ^13^C NMR (CDCl_3_) see Table 2 and Table 3; Positive HRESIMS (*m*/*z*): [M + H]^+^ calcd for C_21_H_33_O_6_, 381.2272; found, 381.2277.

### 3.4. HSQC-Based SMART Analysis

HSQC spectra of 14 primary fractions were acquired by Bruker AVANCE NEO 500 MHz NMR (solvents: CD_3_OD, CDCl_3_, DMSO-*d*_6_) and processed with MestReNova (baseline correction, phase adjustment, peak picking). The HSQC spectra of the fractions were uploaded to the SMART 2.1 online platform (http://smart.ucsd.edu/classic, accessed on 20 December 2023) for dereplication and scaffold predictions. The platform generated candidate structures with cosine similarity scores (>0.7 significant) and predicted cembranolide-enriched fractions (Frs. 6, 7, 9, 10, 13), guiding their prioritization for further separation.

### 3.5. X-Ray Diffraction Data Analysis

The crystallographic data and X-ray structure analyses of **1**, **2**, **5**, **12**, and **13** (Figure 5) were provided in the Supplementary Material.

### 3.6. Anti-Inflammatory Assays in Zebrafish

The assay was performed using 3 dpf (days post-fertilization) healthy macrophage fluorescent transgenic zebrafish (Tg: zlyz-EGFP), which were provided by the Zebrafish Drug Screening Platform of the Institute of Biology, Shandong Academy of Sciences. The zebrafish were divided into blank (CTL), model, positive drug (indomethacin), and sample groups (*n* = 3 biological replicates). Following a 2 h co-incubation with compounds **1**−**14** (for sample groups), the zebrafish underwent a 1 h treatment with 20 μM CuSO_4_ in the dark. Macrophage counts around neuromasts were performed using a fluorescence microscope (Tokyo, Japan), and statistical analysis was performed using ordinary one-way ANOVA (GraphPad Prism 10), with *p* < 0.05 considered significant.

### 3.7. Anti-Inflammatory Assays in BV-2 Cells

**Cell culture.** Mouse microglial cells (BV-2 cell line) were obtained from Servicebio Biotechnology Co. (Wuhan, China). The cells were grown in DMEM supplemented with 10% FBS. The cells were incubated in a condition of 5% CO_2_ and 95% humidity at 37 °C. **Quantitative real-time polymerase chain reaction (RT-qPCR)**. Total RNA was extracted using TRIzol and reverse-transcribed into cDNA using the BeyoRT™ II First Strand cDNA Synthesis kit (RNase H-) (D7168L, Beyotime, Shanghai, China). Real-time PCR was performed using Hieff^®^ qPCR SYBR Green Master Mix (11203ES03, Yeasen, Shanghai, China). Relative mRNA expression of the respective genes was normalized to GAPDH in the same sample using the 2–ΔΔCT method. The mouse primers used in this study are listed below: IL-1*β*, 5′-CTTTCCCGTGGACCTTCCA′-3′ and 5′-CTCGGAGCCTGTAGTGCAGTT-3′; TNF-*α*, 5′-ACAAGGCTGCCCCGACTAC-3′ and 5′-TGGGCTCATACCAGGGTTTG-3′; IL-6, 5′-ACCACTCCCAACAGACCTGTCT-3′ and 5′-CAGATTGTTTTCTGCAAGTGCAT’-3′.

### 3.8. Statistical Analysis

All data were presented as mean ± standard deviation (SD) from at least three independent experiments. Statistical significance was determined using one-way analysis of variance (ANOVA), followed by Dunnett’s post hoc test with GraphPad Prism 10. A *p*-value of less than 0.05 (*p* < 0.05) was considered statistically significant.

## 4. Conclusions

Marine cembranolides fused with a seven-membered lactone moiety at C-1 are known for their anti-inflammatory activities. Inspired by this, we conducted the first targeted investigation of *S. mollis*. Employing a SMART-guided isolation strategy, we efficiently obtained 36 cembranolides, including 14 new (**1**–**14**) and 22 known (**15**–**36**) analogs. The anti-inflammatory potential of 14 new compounds was evaluated in CuSO_4_-induced transgenic fluorescent zebrafish. Compounds **1**, **2**, **4**, and **5** exhibited potent inhibition rates at 20 μM, with an inhibition from 30.4% to 45.6% compared to the positive drug group. Subsequent inflammatory effects studies revealed that compounds **1** and **5** at 10 μM significantly suppressed the LPS-induced production of pro-inflammatory cytokines TNF-*α*, IL-1*β*, and IL-6. In summary, these findings enrich the chemical diversity of marine cembranolides and confirm their anti-inflammatory activities. They provide potential active structures and experimental basis for the development of anti-inflammatory agents targeting immune cell function, offering valuable insights into soft coral-derived anti-inflammatory drug discovery.

## Figures and Tables

**Table 1:** ^1^H NMR spectroscopic data for compounds **1**, **2**, and **5** in CD_3_OD and **3**, **4**, **6**, and **7** in CDCl_3_.

No.	1 ^a^	2 ^a^	3 ^b^	4 ^b^	5 ^a^	6 ^a^	7 ^a^
	*δ*_H_ (*J* in Hz)	*δ*_H_ (*J* in Hz)	*δ*_H_ (*J* in Hz)	*δ*_H_ (*J* in Hz)	*δ*_H_ (*J* in Hz)	*δ*_H_ (*J* in Hz)	*δ*_H_ (*J* in Hz)
1	2.35, m	1.93, m	2.46, m	2.17, m	3.37, m	3.31, m	3.04, m
2a	2.14, m	1.86, m	1.91, m	2.36, m	1.87, m	1.86, m	1.95, m
2b	1.63, m	1.23, m	1.50, m	1.75, m	1.87, m	1.75, m	1.60, m
3	3.27, d (7.0)	3.33, d (6.0)	3.06, dd (10.8, 2.4)	5.51, m	3.75, dd (11.0, 4.5)	3.79, dd (11.5, 5.0)	4.72, dd (11.5, 3.0)
5a	1.86, m	1.78, m	2.15, m	2.27, m	1.75, m	1.86, m	1.71, m
5b	1.45, m	1.39, m	1.33, m	2.05, m	1.61, m	1.57, m	1.53, m
6a	2.11, m	2.07, m	2.36, m	2.48, m	2.03, m	2.03, m	1.95, m
6b	1.72, m	1.82, m	2.15, m	2.03, m	2.03, m	2.03, m	1.82, m
7	5.09, d (8.5)	5.09, d (9.0)	5.29, d (10.4)	5.04, d (11.2)	4.00, dd (9.0, 5.0)	4.20, t (8.0)	3.95, t (6.5)
9a	2.21, m	2.13, m	2.08, m	2.24, m	1.75, m	1.76, m	2.21, m
9b	2.14, m	2.13, m	1.88, m	2.13, m	1.59, m	1.59, m	1.63, m
10a	2.10, m	1.96, m	1.58, m	1.94, m	1.61, m	1.60, m	3.61, m
10b	1.42, m	1.34, m	1.58, m	1.35, m	1.61, m	1.47, m	2.62, m
11	4.16, d (7.0)	4.14, d (7.5)	5.49, d (9.2)	4.04, d (10.8)	6.44, m	6.34, d (10.5)	
13a	2.14, m	2.09, m	1.81, m	2.15, m	2.01, m	1.96, m	2.36, dd (15.0, 5.5)
13b	1.78, m	1.64, m	1.80, m	2.15, m	2.01, m	1.84, m	1.90, m
14a	2.12, m	1.86, m	2.55, m	2.21, m	2.52, m	2.42, m	2.17, m
14b	1.20, m	1.31, m	1.17, m	1.20, m	1.13, m	1.12, m	1.19, d (6.0)
15		2.90, m					
16a	6.35, s	1.25, d (7.5)	6.30, s	6.26, s	6.20, s	6.28, s	6.30, s
16b	5.85, s		5.43, s	5.41, s	5.60, s	5.49, s	5.44, s
18	1.21, s	1.21, s	1.21, s	1.52, s	1.16, s	1.18, s	1.13, s
19	1.49, s	1.48, s	1.61, s	1.55, s	1.17, s	1.09, s	1.13, s
20	1.29, s	1.22, s	1.39, s	1.31, s	1.36, s	1.35, s	1.45, s
22			2.11, s		2.14, s	2.13, s	2.02, s
23						3.18, s	

^a^ Recorded at 500 MHz. ^b^ at 400 MHz.

**Table 2:** ^13^C NMR spectroscopic data for compounds **1**, **2**, and **5** in CD_3_OD, **3**, **4**, **6**–**12**, and **14** in CDCl_3_, and **13** in DMSO-*d*_6_.

No.	1 ^c^	2 ^c^	3 ^d^	4 ^d^	5 ^c^	6 ^c^	7 ^c^	8 ^d^	9 ^c^	10 ^c^	11 ^d^	12 ^c^	13 ^d^	14 ^c^
	*δ*_C,_ Type	*δ*_C,_ Type	*δ*_C,_ Type	*δ*_C,_ Type	*δ*_C,_ Type	*δ*_C,_ Type	*δ*_C,_ Type	*δ*_C,_ Type	*δ*_C,_ Type	*δ*_C,_ Type	*δ*_C,_ Type	*δ*_C,_ Type	*δ*_C,_ Type	*δ*_C,_ Type
1	38.5, CH	34.8, CH	34.9, CH	42.6, CH	33.9, CH	32.6, CH	32.6, CH	33.2, CH	37.7, CH	39.5, CH	38.6, CH	35.9, CH	36.0, CH	35.8, CH
2	37.2, CH_2_	37.2, CH_2_	33.1, CH_2_	31.5, CH_2_	38.3, CH_2_	37.5, CH_2_	34.8, CH_2_	37.7, CH_2_	33.3, CH_2_	33.7, CH_2_	35.6, CH_2_	31.6, CH_2_	29.0, CH_2_	30.3, CH_2_
3	74.0, CH	74.2, CH	63.7, CH	125.0, CH	75.7, CH	75.5, CH	76.1, CH	77.6, CH	60.8, CH	60.0, CH	75.4, CH	70.0, CH	71.3, CH	58.3, CH
4	75.9, C	76.2, C	60.7, C	136.6, C	88.9, C	87.5, C	85.7, C	133.1, C	58.1, C	61.1, C	85.4, C	75.3, C	83.4, C	61.2, C
5	39.8, CH_2_	39.8, CH_2_	38.2, CH_2_	39.0, CH_2_	38.3, CH_2_	37.3, CH_2_	37.1, CH_2_	128.0, CH	46.8, CH_2_	43.0, CH_2_	37.5, CH_2_	38.9, CH_2_	34.8, CH_2_	40.8, CH_2_
6	23.4, CH_2_	23.7, CH_2_	25.1, CH_2_	26.2, CH_2_	26.3, CH_2_	25.0, CH_2_	25.8, CH_2_	27.1, CH_2_	65.7, CH	125.6, CH	28.1, CH_2_	23.5, CH_2_	27.1, CH_2_	123.9, CH
7	128.7, CH	128.8, CH	127.5, CH	127.1, CH	85.7, CH	83.0, CH	85.3, CH	124.7, CH	128.1, CH	135.5, CH	82.5, CH	127.0, CH	88.1, CH	139.4, CH
8	136.2, C	135.3, C	133.5, C	134.3, C	74.9, C	78.3, C	73.9, C	135.7, C	141.3, C	84.3, C	87.1, C	133.1, C	71.3, C	73.2, C
9	37.8, CH_2_	37.4, CH_2_	35.4, CH_2_	35.8, CH_2_	40.5, CH_2_	33.1, CH_2_	34.0, CH_2_	35.7, CH_2_	36.8, CH_2_	34.4, CH_2_	30.2, CH_2_	35.1, CH_2_	30.5, CH_2_	33.6, CH_2_
10	29.1, CH_2_	28.8, CH_2_	25.3, CH_2_	26.8, CH_2_	26.8, CH_2_	25.8, CH_2_	34.1, CH_2_	26.9, CH_2_	29.6, CH_2_	24.7, CH_2_	27.1, CH_2_	25.5, CH_2_	30.6, CH_2_	32.8, CH_2_
11	68.7, CH	68.6, CH	70.9, CH	68.3, CH	75.9, CH	74.4, CH	211.0, C	69.0, CH	74.5, CH	77.6, CH	86.5, CH	68.9, CH	214.2, C	215.8, C
12	89.4, C	88.6, C	85.9, C	87.6, C	90.3, C	88.1, C	91.8, C	78.9, C	74.3, C	74.5, C	75.7, C	78.5, C	77.6, C	79.1, C
13	32.8, CH_2_	33.1, CH_2_	31.9, CH_2_	31.9, CH_2_	34.4, CH_2_	33.7, CH_2_	34.4, CH_2_	30.9, CH_2_	37.1, CH_2_	37.5, CH_2_	35.0, CH_2_	26.2, CH_2_	38.3, CH_2_	36.4, CH_2_
14	31.3, CH_2_	26.4, CH_2_	31.6, CH_2_	30.9, CH_2_	30.4, CH_2_	29.5, CH_2_	30.4, CH_2_	29.3, CH_2_	26.6, CH_2_	26.4, CH_2_	25.7, CH_2_	19.6, CH_2_	24.2, CH_2_	24.7, CH_2_
15	143.6, C	42.1, CH	144.4, C	145.4, C	146.3, C	144.4, C	144.5, C	154.1, C	143.5, C	143.0, C	144.7, C	43.3, CH	142.5, C	141.3, C
16	126.0, CH_2_	11.0, CH_3_	124.6, CH_2_	123.1, CH_2_	124.6, CH_2_	124.2, CH_2_	124.7, CH_2_	102.4, CH_2_	124.7, CH_2_	124.7, CH_2_	123.1, CH_2_	15.8, CH_3_	123.2, CH_2_	125.2, CH_2_
17	172.0, C	181.5, C	168.8, C	169.7, C	171.4, C	169.1, C	168.6, C	64.9, C	167.7, C	167.4, C	167.2, C	177.6, C	166.5, C	167.6, C
18	24.1, CH_3_	24.0, CH_3_	16.3, CH_3_	14.9, CH_3_	15.6, CH_3_	15.2, CH_3_	18.6, CH_3_	9.5, CH_3_	17.8, CH_3_	17.3, CH_3_	18.3, CH_3_	23.6, CH_3_	20.9, CH_3_	18.1, CH_3_
19	15.8, CH_3_	15.7, CH_3_	15.9, CH_3_	15,8, CH_3_	18.9, CH_3_	16.8, CH_3_	23.8, CH_3_	15.3, CH_3_	16.5, CH_3_	24.5, CH_3_	24.5, CH_3_	15.8, CH_3_	26.2, CH_3_	31.2, CH_3_
20	23.3, CH_3_	23.1, CH_3_	24.4, CH_3_	23.3, CH_3_	24.2, CH_3_	24.3, CH_3_	29.5, CH_3_	18.2, CH_3_	24.8, CH_3_	25.1, CH_3_	22.8, CH_3_	17.4, CH_3_	22.6, CH_3_	27.6, CH_3_
21			170.5, C		173.0, C	171.3, C	170.1, C		52.2, CH_3_	52.1, CH_3_	52.1, CH_3_	51.6, CH_3_	170.1, C	52.2, CH_3_
22			21.4, CH_3_		20.9, CH_3_	21.2, CH_3_	21.3, CH_3_					49.4, CH_3_	20.9, CH_3_	
23						49.1, CH_3_							51.7, CH_3_	

^c^ Recorded at 125 MHz. ^d^ at 100 MHz.

**Table 3:** ^1^H NMR spectroscopic data for compounds **8**–**12**, **14** in CDCl_3_, and **13** in DMSO-*d*_6_.

No.	8 ^b^	9 ^a^	10 ^a^	11 ^b^	12 ^a^	13 ^b^	14 ^a^
	*δ*_H_ (*J* in Hz)	*δ*_H_ (*J* in Hz)	*δ*_H_ (*J* in Hz)	*δ*_H_ (*J* in Hz)	*δ*_H_ (*J* in Hz)	*δ*_H_ (*J* in Hz)	*δ*_H_ (*J* in Hz)
1	2.28, m	2.76, m	2.61, m	2.82, m	2.00, m	2.60, m	2.87, m
2a	1.75, m	1.67, m	1.93, m	2.27, m	1.78, t (13.5)	2.02, m	2.12, m
2b	1.59, m	1.67, m	1.50, m	1.61, m	1.01, m	1.85, m	1.37, m
3	4.20, m	2.73, t (6.0)	2.81, dd (8.5, 5.0)	3.47, t (4.0)	3.60, d (10.5)	4.46, d (9.6)	2.84, dd (11.0, 4.0)
5a	5.41, d (11.2)	2.34, dd (13.5, 3.0)	2.69, dd (14.0, 6.0)	1.89, m	1.97, m	1.65, m	2.48, dd (14.0, 10.0)
5b		1.44, t (11.5)	1.93, m	1.71, m	1.64, m	1.43, m	2.26, dd (14.0, 5.5)
6a	3.16, m	4.49, td (10.5, 2.5)	5.53, dd (9.0, 6.0)	2.22, m	2.42, m	1.71, m	5.57, m
6b	2.41, m			1.66, m	2.15, m	1.46, m	
7	5.35, t (8.0)	5.22, d (9.5)	5.40, d (16.5)	4.20, dd (10.0, 3.6)	5.43, d (8.0)	3.83, m	5.45, d (15.5)
9a	2.17, m	2.25, m	2.19, m	1.97, m	2.23, m	1.80, m	1.95, m
9b	2.17, m	2.17, m	2.19, m	1.52, m	2.14, m	1.33, m	1.88, m
10a	2.00, m	1.67, m	1.50, m	2.10, m	1.86, m	3.29, m	2.74, m
10b	1.19, m	1.57, m	1.50, m	1.92, m	1.28, m	2.49, m	2.74, m
11	3.63, d (10.0)	3.55, d (10.0)	3.34, t (6.5)	4.05, dd (9.6, 4.8)	3.37, d (11.5)		
13a	1.78, m	1.63, m	1.50, m	2.50, t (9.2)	1.58, m	1.97, m	1.85, m
13b	1.54, m	1.50, m	1.50, m	1.35, m	1.38, m	1.49, m	1.47, m
14a	1.88, m	1.62, m	1.50, m	1.58, m	1.50, m	1.27, m	1.61, m
14b	0.88, m	1.52, m	1.50, m	1.39, m	1.42, m	0.88, m	1.23, m
15					2.37, m		
16a	4.67, s	6.29, s	6.26, s	6.17, s	1.19, s	5.95, s	6.35, s
16b	4.67, s	5.59, s	5.58, s	5.52, s		5.28, s	5.52, s
17a	4.28, m						
17b	4.20, m						
18	1.66, s	1.28, s	1.39, s	1.12, s	1.09, d (7.0)	1.07, s	1.30, s
19	1.59, s	1.78, s	1.39, s	1.12, s	1.60, s	0.96, s	1.33, s
20	0.97, s	1.24, s	1.24, s	1.11, s	1.05, s	1.12, s	1.34, s
21		3.75, s	3.75, s	3.76, s	3.67, s	-	3.76, s
22					3.14, s	1.82, s	
23						3.69, s	

^a^ Recorded at 500 MHz. ^b^ at 400 MHz.

## Data Availability

Data are contained within the article or Supplementary Materials; further inquiries can be directed to the corresponding author.

## Supplementary Materials

The following supporting information can be downloaded at: https://www.mdpi.com/article/10.3390/md23120465/s1, Figures S1–S5: SMART results of Frs. 6, 7, 9, 10, and 13; Figures S6–S19: NMR, UV and MS spectrum of compounds **1**–**14**; Tables S1–S5: X-ray crystallographic analysis of **1**, **2**, **5**, **12**, and **13**. Tables S6–16: Calculation Details of **1**–**4**, **6**–**11**, and **14**.
