# Supplementary material for: SMART-Guided Isolation and Identification of Seven-Membered Cembranolides with Anti-Inflammation Activity from the Soft Coral Sinularia mollis
Source: Mar Drugs. 2025 Dec 5;23(12):465. doi: 10.3390/md23120465 (PMC12734715; doi:10.3390/md23120465)
Supplement: Supplementary file 1 [file marinedrugs-23-00465-s001.zip › marinedrugs-4008402-supplementary.pdf]

Supporting Information

SMART-guided Isolation and Identification of Seven-membered Cembranolides with Anti-inflammation Activity from the Soft Coral *Sinularia Mollis*

Huiyue Hou, [1] Pinglin Li, [1, 2, *]

[1] Key Laboratory of Marine Drugs, Chinese Ministry of Education, School of Medicine and Pharmacy, Ocean University of China, Qingdao 266003, People's Republic of China

[2] Laboratory of Marine Drugs and Biological Products, National Laboratory for Marine Science and Technology, Qingdao 266235, People's Republic of China

* Corresponding author.

E-mail address: lipinglin@ouc.edu.cn

Table of Contents

1. SMART-guided isolation 4

Figure.S1 HSQC spectrum and SMART results (top 10 structures) of **Fr.6** 4
Figure.S2 HSQC spectrum and SMART results (top 10 structures) of **Fr.7** 4
Figure.S3 HSQC spectrum and SMART results (top 10 structures) of **Fr.9** 4
Figure.S4 HSQC spectrum and SMART results (top 10 structures) of **Fr.10** 4
Figure.S5 HSQC spectrum and SMART results (top 10 structures) of **Fr.13** 5

2. Extraction and Isolation.. 5

3. NMR and MS spectrum of 1–14 7

Figure.S6 Spectra (HRESIMS, UV, ^{1}H NMR, ^{13}C NMR, HSQC, HMBC, ^{1}H–^{1}H COSY, NOESY) for compound 1 7
Figure.S7 Spectra (HRESIMS, UV, ^{1}H NMR, ^{13}C NMR, HSQC, HMBC, ^{1}H–^{1}H COSY, NOESY) for compound 2 11
Figure.S8 Spectra (HRESIMS, UV, ^{1}H NMR, ^{13}C NMR, HSQC, HMBC, ^{1}H–^{1}H COSY, NOESY) for compound 3 15
Figure.S9 Spectra (HRESIMS, UV, ^{1}H NMR, ^{13}C NMR, HSQC, HMBC, ^{1}H–^{1}H COSY, NOESY) for compound 4 19
Figure.S10 Spectra (HRESIMS, UV, ^{1}H NMR, ^{13}C NMR, HSQC, HMBC, ^{1}H–^{1}H COSY, NOESY) for compound 5 23
Figure.S11 Spectra (HRESIMS, UV, ^{1}H NMR, ^{13}C NMR, HSQC, HMBC, ^{1}H–^{1}H COSY, NOESY) for compound 6 27
Figure.S12 Spectra (HRESIMS, UV, ^{1}H NMR, ^{13}C NMR, HSQC, HMBC, ^{1}H–^{1}H COSY, NOESY) for compound 7 31
Figure.S13 Spectra (HRESIMS, UV, ^{1}H NMR, ^{13}C NMR, HSQC, HMBC, ^{1}H–^{1}H COSY, NOESY) for compound 8 35
Figure.S14 Spectra (HRESIMS, UV, ^{1}H NMR, ^{13}C NMR, HSQC, HMBC, ^{1}H–^{1}H COSY, NOESY) for compound 9 39
Figure.S15 Spectra (HRESIMS, UV, ^{1}H NMR, ^{13}C NMR, HSQC, HMBC, ^{1}H–^{1}H COSY, NOESY) for compound 10 43
Figure.S16 Spectra (HRESIMS, UV, ^{1}H NMR, ^{13}C NMR, HSQC, HMBC, ^{1}H–^{1}H COSY, NOESY) for compound 11 47
Figure.S17 Spectra (HRESIMS, UV, ^{1}H NMR, ^{13}C NMR, HSQC, HMBC, ^{1}H–^{1}H COSY, NOESY) for compound 12 51
Figure.S18 Spectra (HRESIMS, UV, ^{1}H NMR, ^{13}C NMR, HSQC, HMBC, ^{1}H–^{1}H COSY, NOESY) for compound 13 55
Figure.S19 Spectra (HRESIMS, UV, ^{1}H NMR, ^{13}C NMR, HSQC, HMBC, ^{1}H–^{1}H COSY, NOESY) for compound 14 59

4. X-ray crystallographic analysis of 1,2,5,12, and 13 62

Table S1. Crystallographic data of 1 62
Table S2. Crystallographic data of 2 63
Table S3. Crystallographic data of 5 64
Table S4. Crystallographic data of 12 65

Table S5. Crystallographic data of 13 66
5. Calculation Details 66
Table S6. Calculation process of 1 67
Table S7. Calculation process of 2 75
Table S8. Calculation process of 3 88
Table S9. Calculation process of 4 96
Table S10. Calculation process of 6 100
Table S11. Calculation process of 7 106
Table S12. Calculation process of 8 114
Table S13. Calculation process of 9 118
Table S14. Calculation process of 10 124
Table S15. Calculation process of 11 130
Table S16. Calculation process of 14 133

1. SMART-guided isolation

Figure.S1 HSQC spectrum and SMART results (top 10 structures) of **Fr.6**

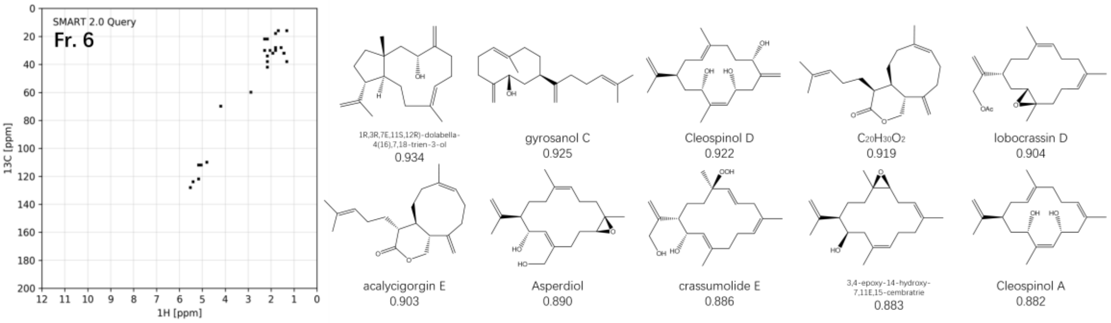

Figure.S2 HSQC spectrum and SMART results (top 10 structures) of **Fr.7**

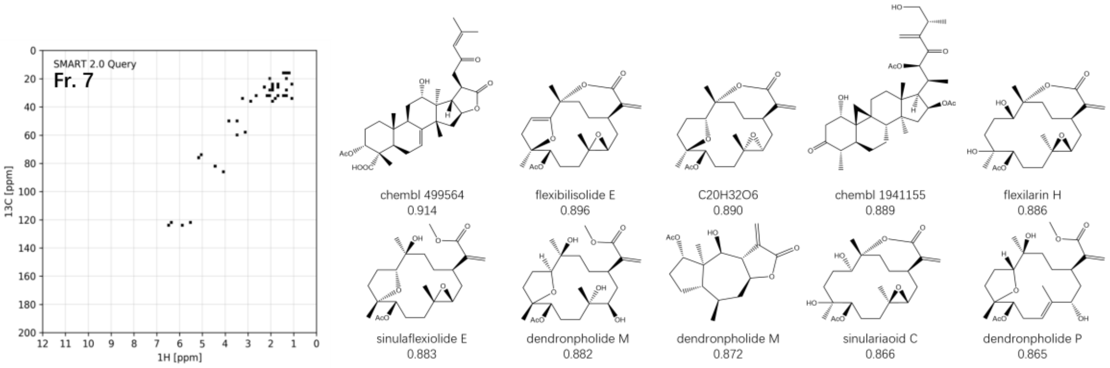

Figure.S3 HSQC spectrum and SMART results (top 10 structures) of **Fr.9**

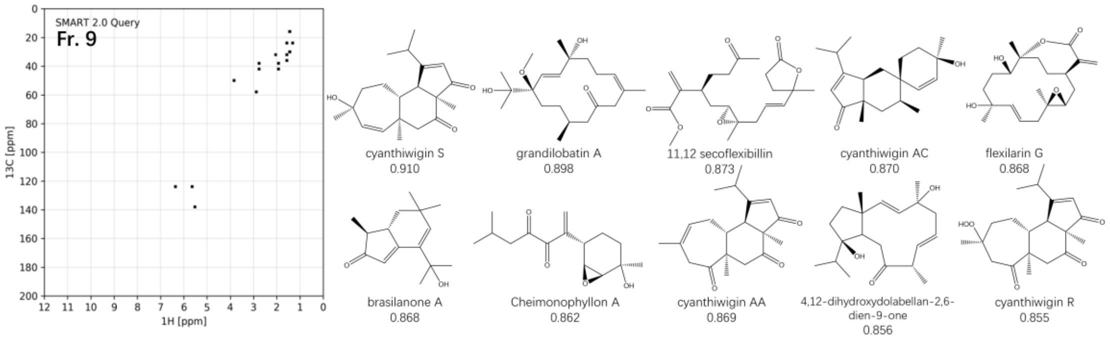

Figure.S4 HSQC spectrum and SMART results (top 10 structures) of **Fr.10**

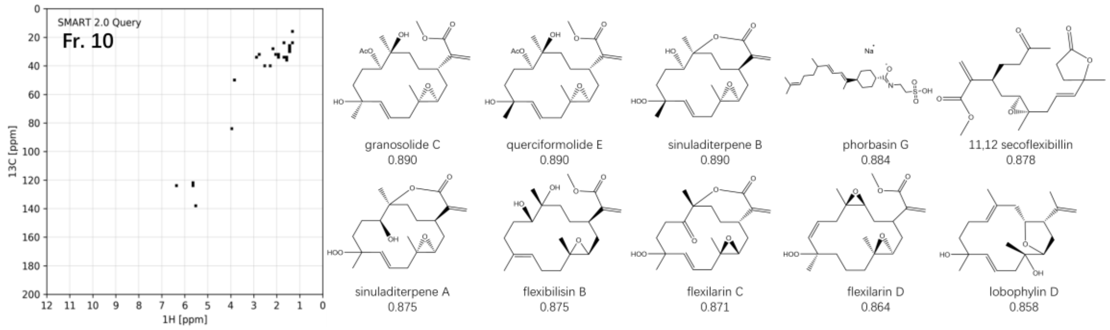

Figure.S5 HSQC spectrum and SMART results (top 10 structures) of **Fr.13**

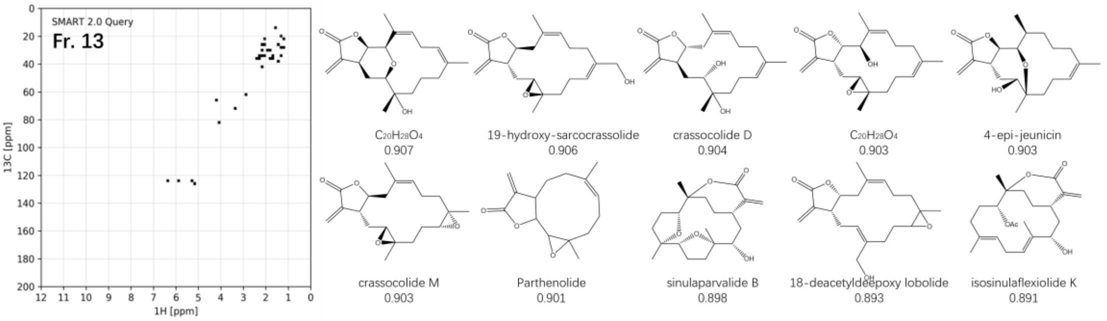

2. Extraction and Isolation

The fresh specimen of *Sinularia Mollis* (7.3 kg, wet weight) was crushed and extracted with MeOH six times (each time for seven days) at room temperature, and then the concentrated residue (107.2 g) was dissolved in MeOH (1 L) to remove salts. The total extract was divided into 14 fractions (Frs. 1–14) by the vacuum liquid chromatography on a silica gel column eluting with a gradient of petroleum ether/acetone (from 50:1 to 1:1, V: V) and CH_2Cl_2/ MeOH (from 10:1 to 1:1, V: V). Fr. 6 (4.14g) was subjected to a silica gel CC eluted with petroleum ether-acetone (from 30:1 to 0:1, v/v) to give six (Frs. 6.1–6.6) subfractions. Fraction 6.4 (759.4mg) was separated by MPLC (C-18 ODS) with MeOH/H_2O (75: 25, V: V) to yield seven subfractions (Frs. 6.4.1–6.4.5), Fr. 6.4.2 (283.7 mg), Fr. 6.4.3 (16.5 mg) and Fr. 6.4.4 (10.1 mg)were further separated by semi-preparative HPLC (ODS, 5 μm, 250 × 10 mm) with MeOH/H_2O (60:30, V: V) as the mobile phase at a flow rate of 2 mL/min to obtain **15** (20 mg, t_R 17.6 min), **3** (2.7 mg, t_R 15.8 min), **4** (2.6mg, t_R 24.5 min), and **30** (10.4mg, t_R 15.6 min), respectively.Fr.6.5 (108mg) was purified to obtain compound **8** (2.6mg, t_R 24.5 min). Fr. 6.6 (70 mg) was further separated under uniform conditions to afford compound **16** (1.4 mg, t_R 7.4 min). Fraction 7 (8.17 g) was initially fractioned by silica gel CC eluting with petroleum ether-acetone (from 20:1 to 0:1, V: V) to give five (Frs. 7.1–7.5) subfractions. Portion 7.3 was separated by MPLC with a gradient of MeOH/H_2O (60:40, V: V) to afford four subfractions (Frs. 7.3.1–7.3.4). Fr. 7.3.1 was purified using semi-preparative HPLC (MeOH/H_2O, 65: 35, V/V; 2 mL/min) to afford **17** (14 mg, t_R 12.8 min) and **18** (4 mg, t_R 14 min). The half of Fr. 7.4 (4.13 g) was separated by MPLC (C-18 ODS) with MeOH/H_2O (65:35, V: V) to yield seven subfractions (Frs. 7.4.1–7.4.7). Fr. 7.4.4 (638.1 mg) was isolated by semi-preparative HPLC eluted with MeOH/H_2O (55:45, V: V) to obtain compound **20** (31.3 mg, t_R 12.9 min), **21** (7.3 mg, t_R 12.9 min), and **31** (8.3 mg, t_R 13.8 min). Fr. 7.4.5 (58.4mg) was also purified by semi-preparative HPLC (ODS, 5 μM, 250 × 10 mm; MeOH/H_2O 75: 25, V/V; 2 mL/min) to afford **24** (4.1 mg, t_R 25 min). Fr. 7.4.7 (89.2 mg) was purified using semi-preparative HPLC (MeOH/H_2O, 65:35, V/V; 2 mL/min) to afford **23** (76 mg, t_R 10 min). Fr. 7.5 (692.4 mg) was separated by MPLC (C-18 ODS) with MeOH/H_2O (60:40, V: V) to yield six subfractions (Frs. 7.5.1–7.5.6). Fr. 7.5.1 was purified using semi-preparative HPLC (CH_3CN/H_2O, 30: 70, V: V, 2 mL/min) to afford **19** (6.3mg, t_R 8.5 min). Fr. 9 (3.1 g) was isolated on silica gel CC eluting with petroleum ether-acetone (from 8: 1 to 0: 1, V: V) to give subfractions Fr. 9.1–Fr. 9.4. Fr. 9.4 (1.3 g) was separated by MPLC (C-18 ODS) with MeOH/H_2O (50: 50, V: V) to yield four subfractions (Fr. 9.4.1 to Fr. 9.4.4). Fr. 9.4.1 (568.9 mg) was isolated by semi-preparative HPLC eluted with CH_3CN/H_2O (30: 70, V: V) to yield five subfractions (Fr. 9.4.1.1 to Fr. 9.4.1.5). Fr. 9.4.1.2 (79.7mg) and Fr. 9.4.1.3 (45.9 mg) were purified by semi-preparative HPLC (CH_3CN/H_2O, 25:75, V: V) to obtain compounds **9** (2.2 mg, t_R 4.4 min) and **22** (2.6 mg, t_R 15 min), respectively.

Fr. 9.4.2 (330.9 mg) was isolated by semi-preparative HPLC eluted with CH_3CN/H_2O (35: 65, V: V) to yield five subfractions (Fr. 9.4.2.1 to Fr. 9.4.2.3). **35** (10.5mg, t_R 11.3 min) was purified from Fr. 9.4.2.1 (194.8mg). Fr. 9.4.3 (420.3mg) was separated by semi-preparative HPLC eluted with CH_3CN/H_2O (37: 63, V: V) to yield six subfractions (Fr. 9.4.3.1 to Fr. 9.4.3.6). Fr. 9.4.3.2 (129.5 mg) was purified by semi-preparative HPLC (CH_3CN/H_2O, 25:75) to obtain compounds **27** (27.1 mg, t_R 14 min). The half of Fr. 10 (13.62 g) was subjected to a silica gel CC (petroleum ether-acetone, from 10: 1 to 0: 1, V/V) to give four fractions Fr. 10.1-Fr. 10.4. Fr. 10.2 (345.3 mg) was separated by MPLC (C-18 ODS) with $MeOH/H_2O$ (60: 40, V: V) to give five fractions Fr. 10.2.1–Fr. 10.2.5. Fr. 10.2.3 (52.1 mg) was purified using semi-preparative HPLC ($MeOH/H_2O$, 35: 65, V/V; 2 mL/min) to afford **29** (9.6 mg, t_R 20 min). **32** (50 mg, t_R 17.5 min) was purified from Fr. 10.2.4 (152mg). Fr. 10.3 (3 g) was separated by MPLC (C-18 ODS) with $MeOH/H_2O$ (50: 50, V: V) to give four fractions Fr. 10.3.1–Fr. 10.3.4. Fr. 10.3.1 (659.3 mg) was then purified using semi-preparative HPLC ($MeOH/H_2O$, 40: 60, V/V; 2 mL/min) to give four fractions (Fr. 10.3.1.1–Fr. 10.3.1.4). **33** (20.0 mg, t_R 9.9 min) was purified from Fr. 10.3.1.3 (117.1mg). Fr. 10.3.1.4 (85.7mg) was purified using semi-preparative HPLC (CH_3CN/H_2O, 15:85, V/V; 2 mL/min) to afford **26** (7.3 mg, t_R 4.8 min) and **28** (11.9 mg, t_R 20 min). Fr. 10.3.2 (212.9 mg) was then purified using semi-preparative HPLC ($MeOH/H_2O$, 40: 60, V/V; 2 mL/min) to give six fractions Fr. 10.3.2.1–Fr. 10.3.2.6. **10** (5.7 mg, t_R 8.1 min) was isolated from Fr. 10.3.2.2 (12.2mg). Fr.10.3.2.3 (19mg) was purified to afford **14** (6.3 mg, t_R 9.7 min). Fr. 10.3.2.6 (57.1 mg) was purified using semi-preparative HPLC (CH_3CN/H_2O, 25:75, V/V; 2 mL/min) to afford **7** (25 mg, t_R 29.5 min). **34** (30.6 mg, t_R 6.5 min) was purified from Fr.10.3.3 (100mg). Fr. 10.4 (880.4mg) was separated by semi-preparative HPLC eluted with CH_3OH/H_2O (60: 40, V: V) to yield six subfractions (Fr. 10.4.1 to Fr. 10.4.6). **11** (22.6 mg, t_R 4.5 min) and **12** (13.6 mg, t_R 25.5 min) were isolated from Fr. 10.4.3 (89.5mg) and Fr. 10.4.6 (81mg). Fr. 10.4.4 (75.6 mg) was purified using semi-preparative HPLC (CH_3CN/H_2O, 15:85, V/V; 2 mL/min) to afford **6** (2 mg, t_R 17.6 min), **25** (2 mg, t_R 13.2 min). Fr. 13 (3.4 g) was subjected to a silica gel CC (petroleum ether-acetone, from 5: 1 to 0: 1, V/V) to give six fractions Fr. 13.1-Fr. 13.6. **13** (14.0 mg, t_R 21.9 min) was isolated from Fr. 13.4 (89.1mg). Fr. 13.5 (334.8 mg) was subjected to C18 MPLC using $MeOH/H_2O$ (50:50, V: V) to afford four subfractions (Fr. 13.5.1-13.5.4). Fr. 13.5.2 (80.6 mg) was purified using semi-preparative HPLC (CH_3CN/H_2O, 45:55, V/V, 2 mL/min) to afford **1** (14 mg, t_R 15.1 min), **2** (5 mg, t_R 18.3 min) and **5** (18 mg, t_R 20 min).

3. NMR and MS spectrum of 1–14

Figure.S6 Spectra (HRESIMS, UV, ^{1}H NMR, ^{13}C NMR, HSQC, HMBC, ^{1}H–^{1}H COSY, NOESY) for compound 1

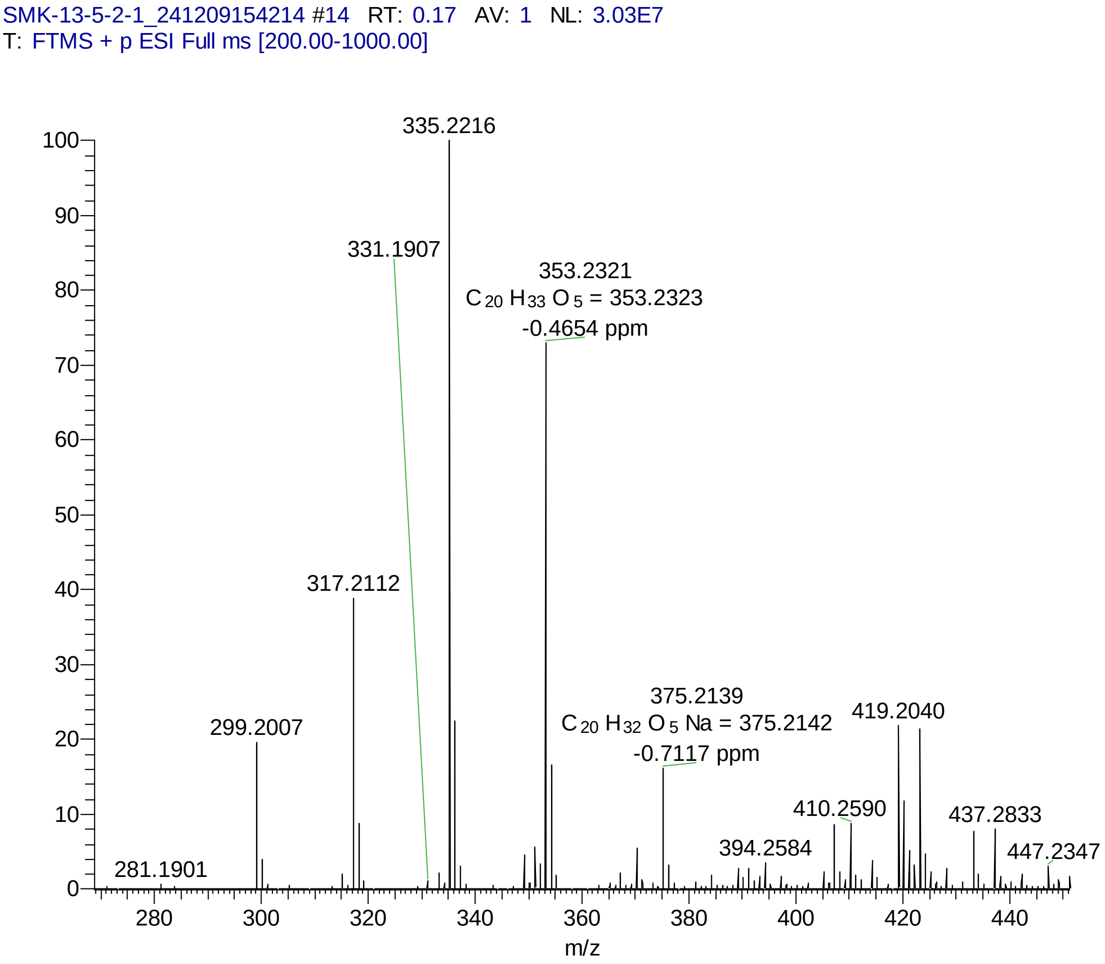

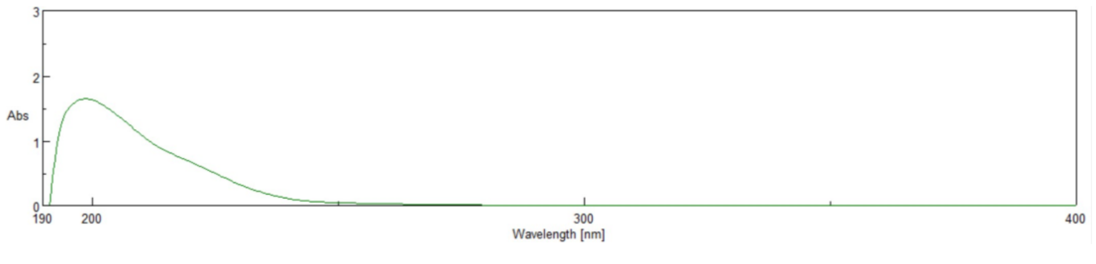

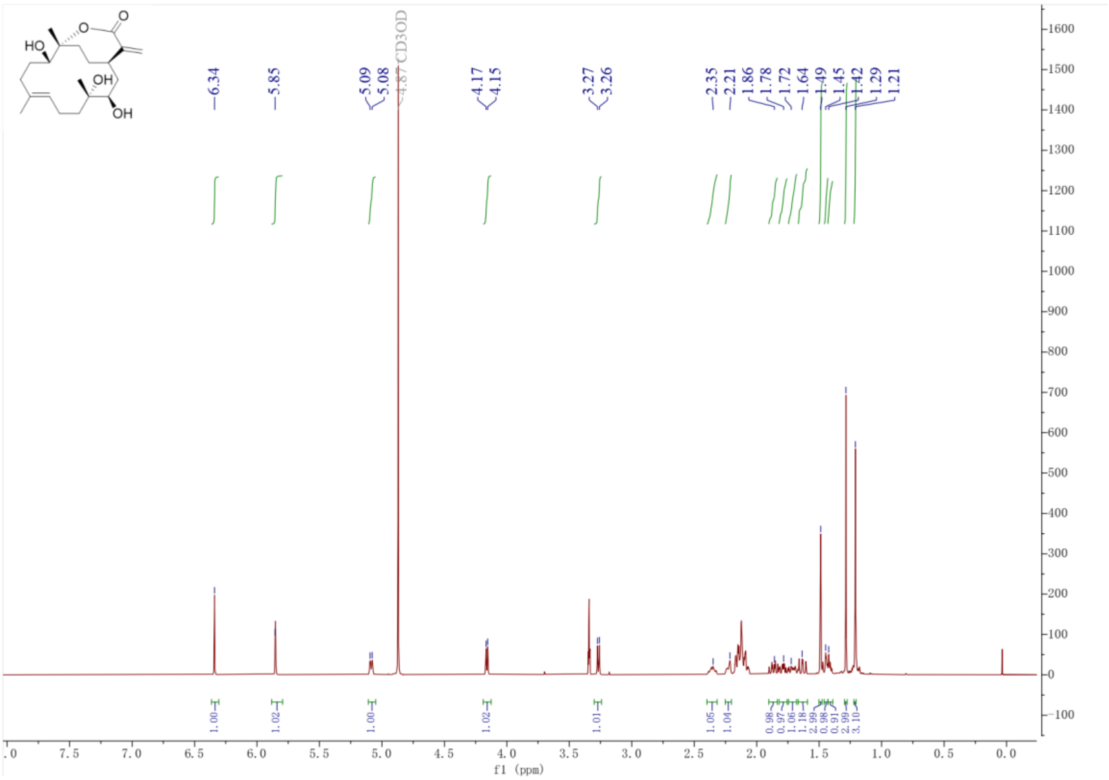
HO
O
O
OH
OH
6.34
5.85
5.09
5.08
4.87 CD3OD
4.17
4.15
3.27
3.26
2.35
2.21
1.86
1.78
1.72
1.64
1.49
1.45
1.42
1.29
1.21
1.00
1.02
1.00
1.02
1.01
1.05
1.04
f1 (ppm)

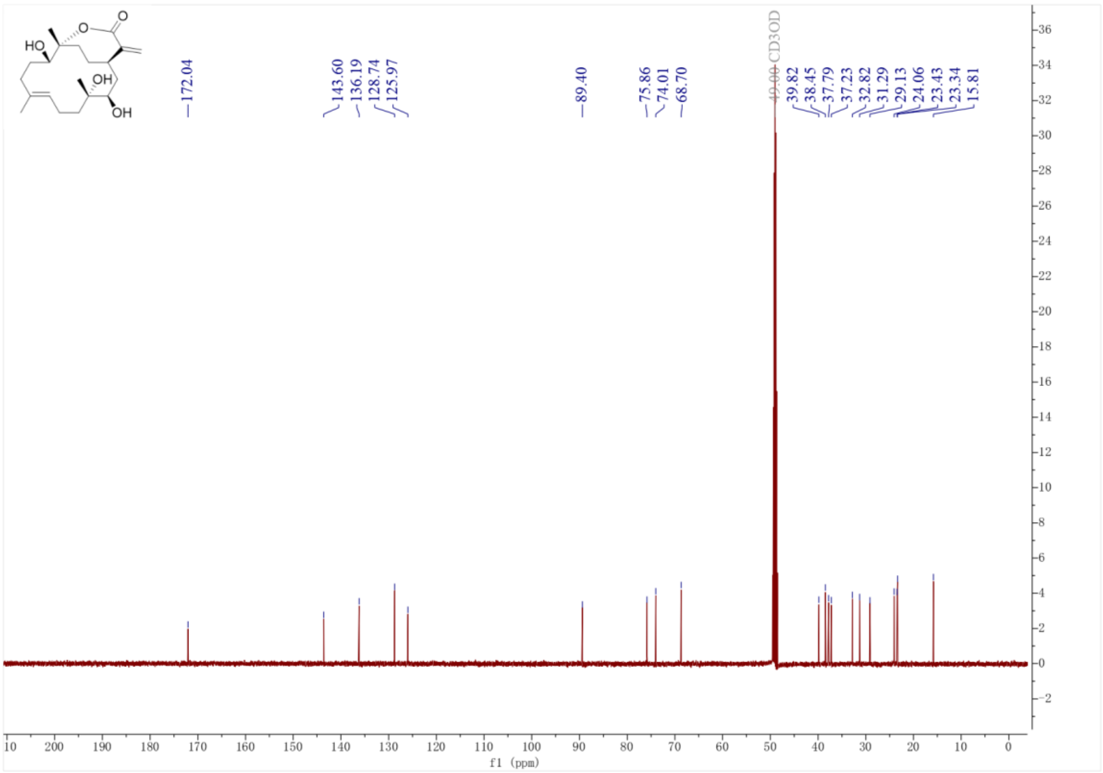
HO
O
O
OH
OH
172.04
143.60
136.19
128.74
125.97
89.40
75.86
74.01
68.70
49.00 CD3OD
39.82
38.45
37.79
37.23
32.82
31.29
29.13
24.06
23.43
23.34
15.81
f1 (ppm)

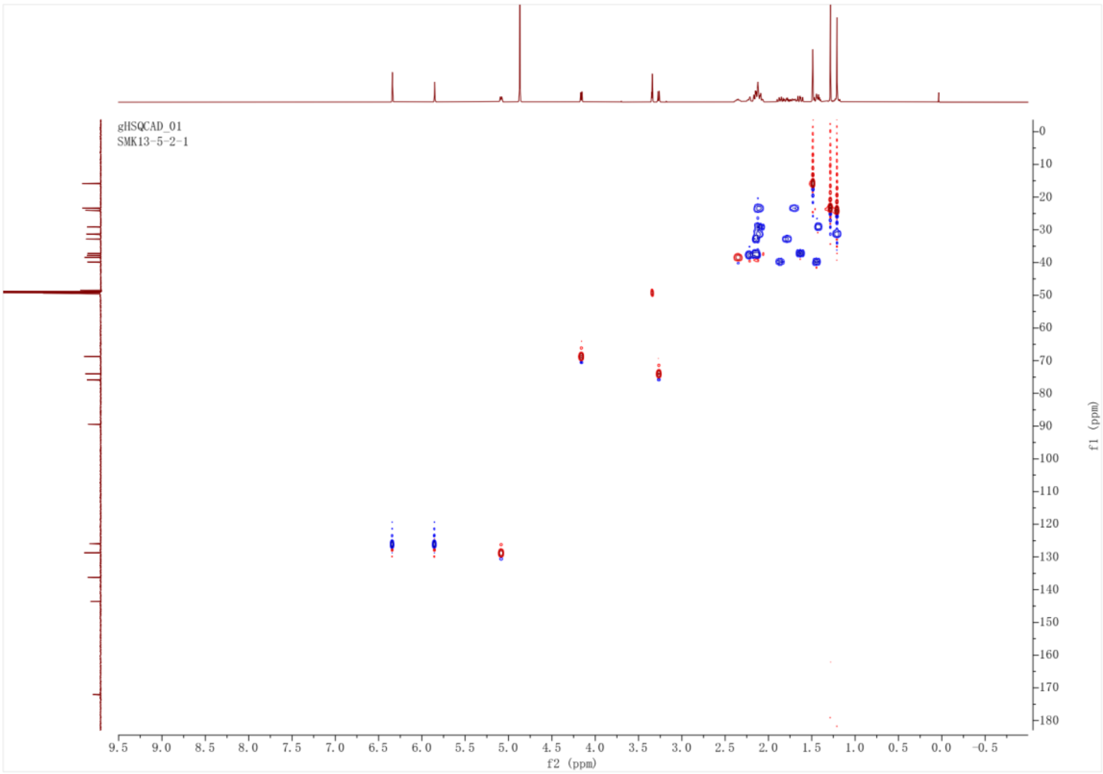
gHSQCAD_01
SMK13-5-2-1
f2 (ppm)
f1 (ppm)

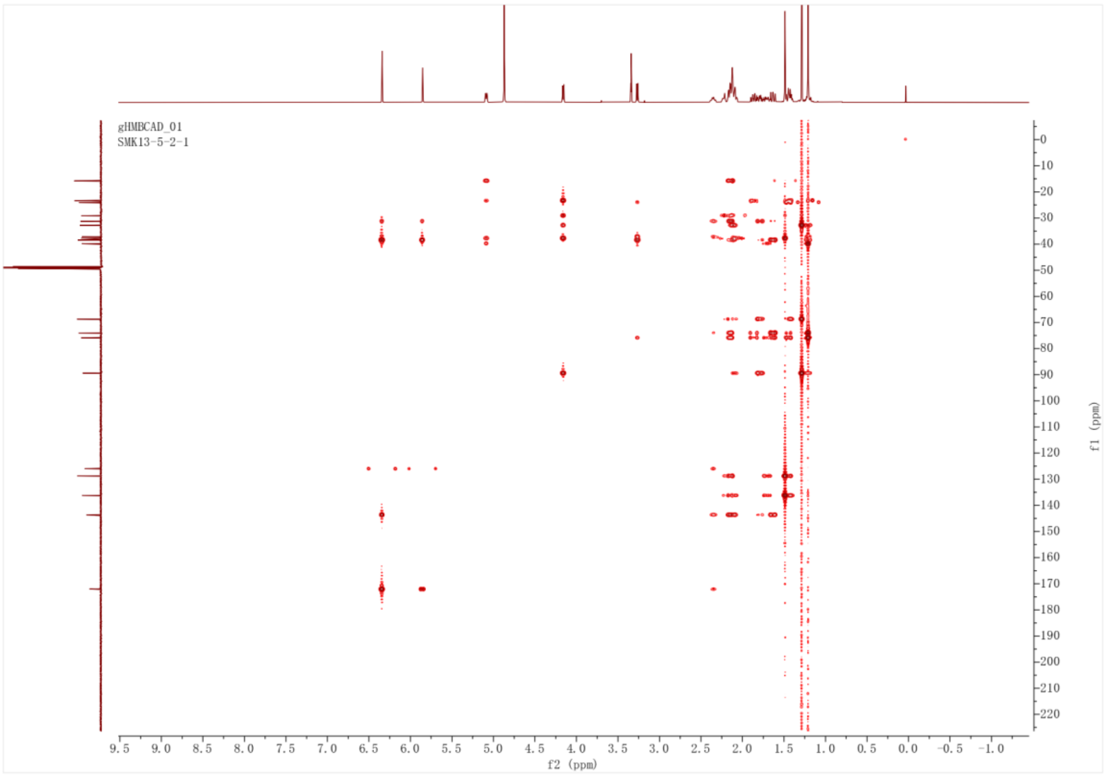
gHMBCAD_01
SMK13-5-2-1
f2 (ppm)
f1 (ppm)

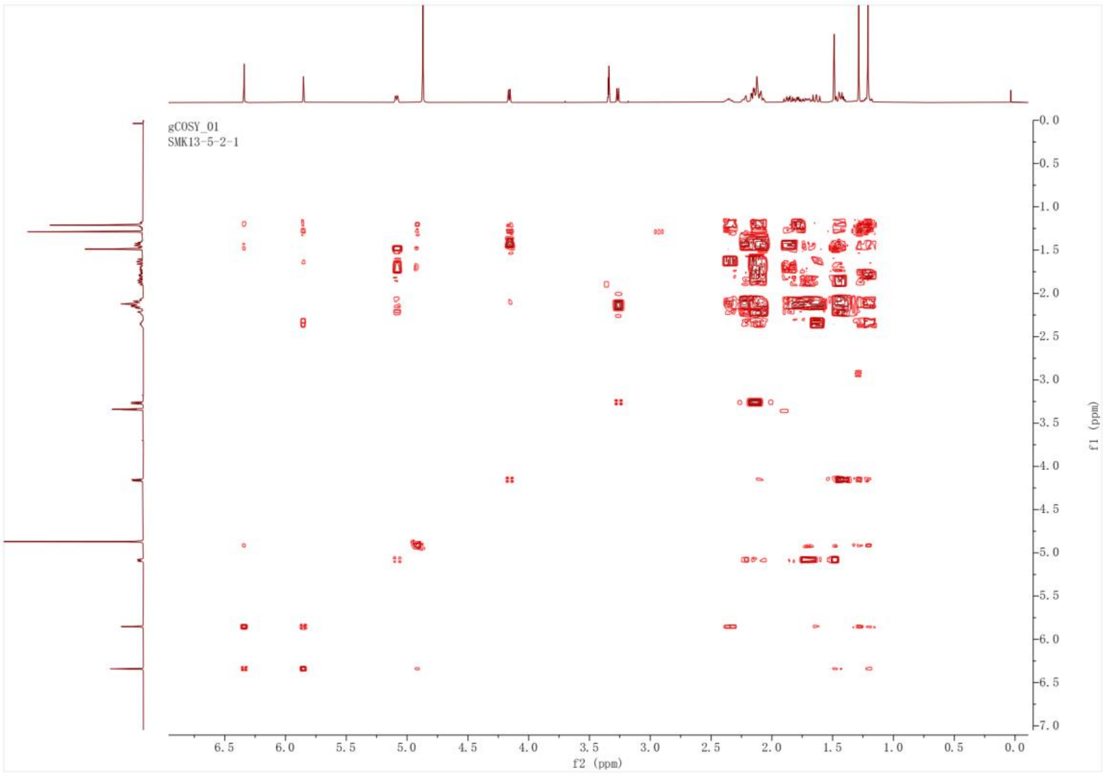
gCOSY_01
SMK13-5-2-1
f2 (ppm)
f1 (ppm)

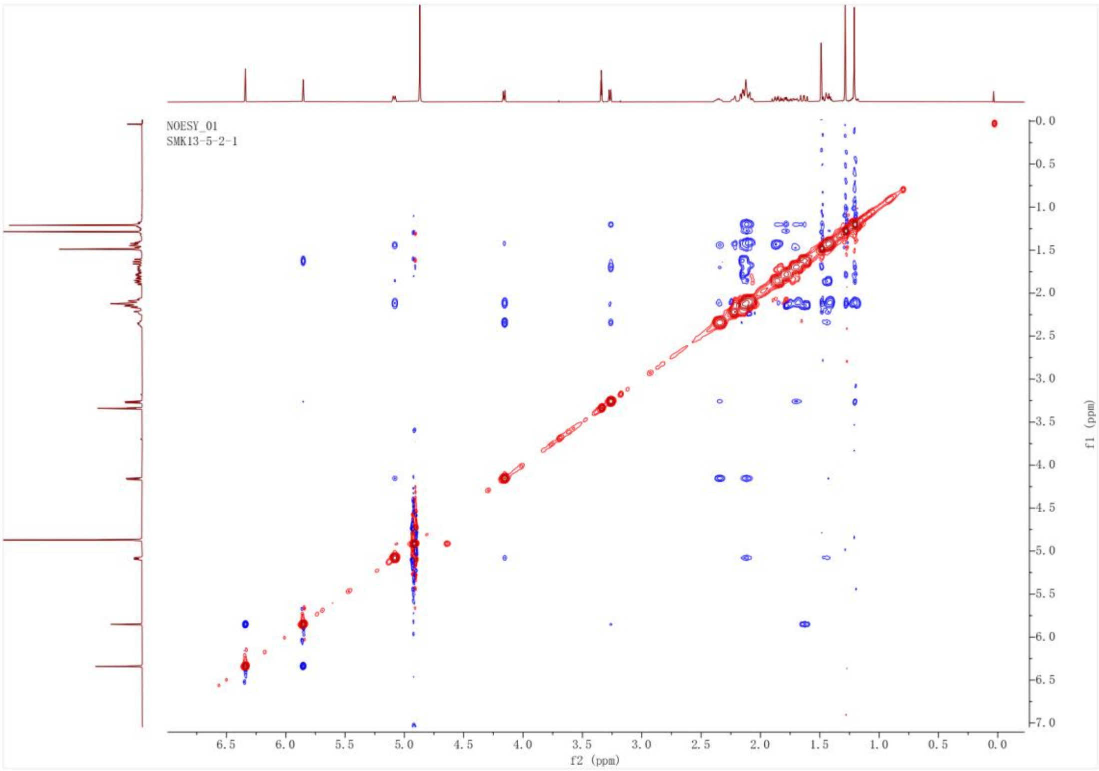
NOESY_01
SMK13-5-2-1
f2 (ppm)
f1 (ppm)

Figure.S7 Spectra (HRESIMS, UV, ^{1}H NMR, ^{13}C NMR, HSQC, HMBC, ^{1}H–^{1}H COSY, NOESY) for compound 2

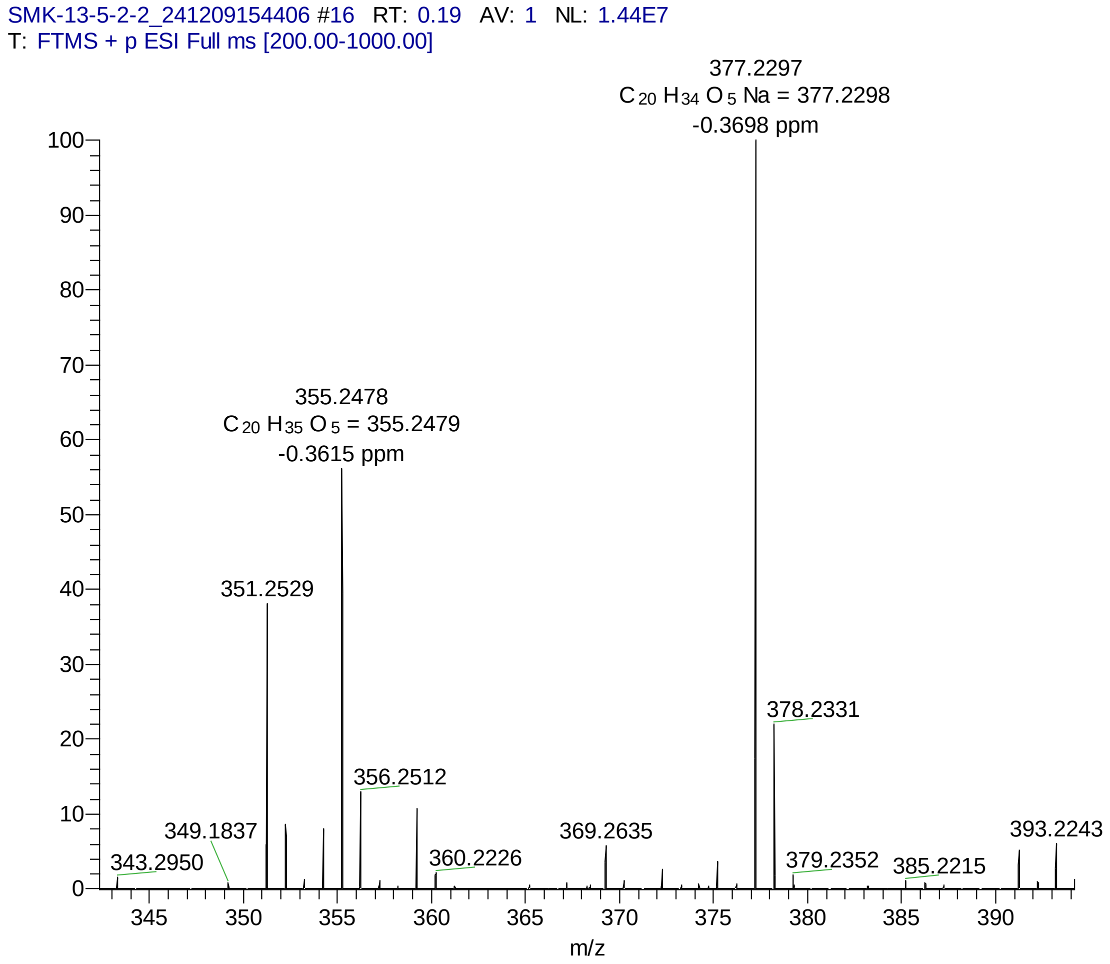

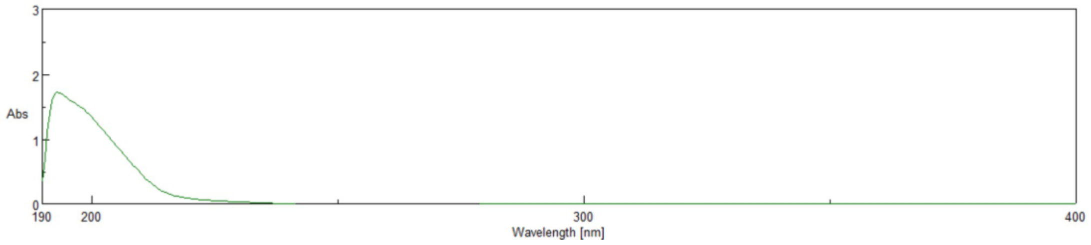

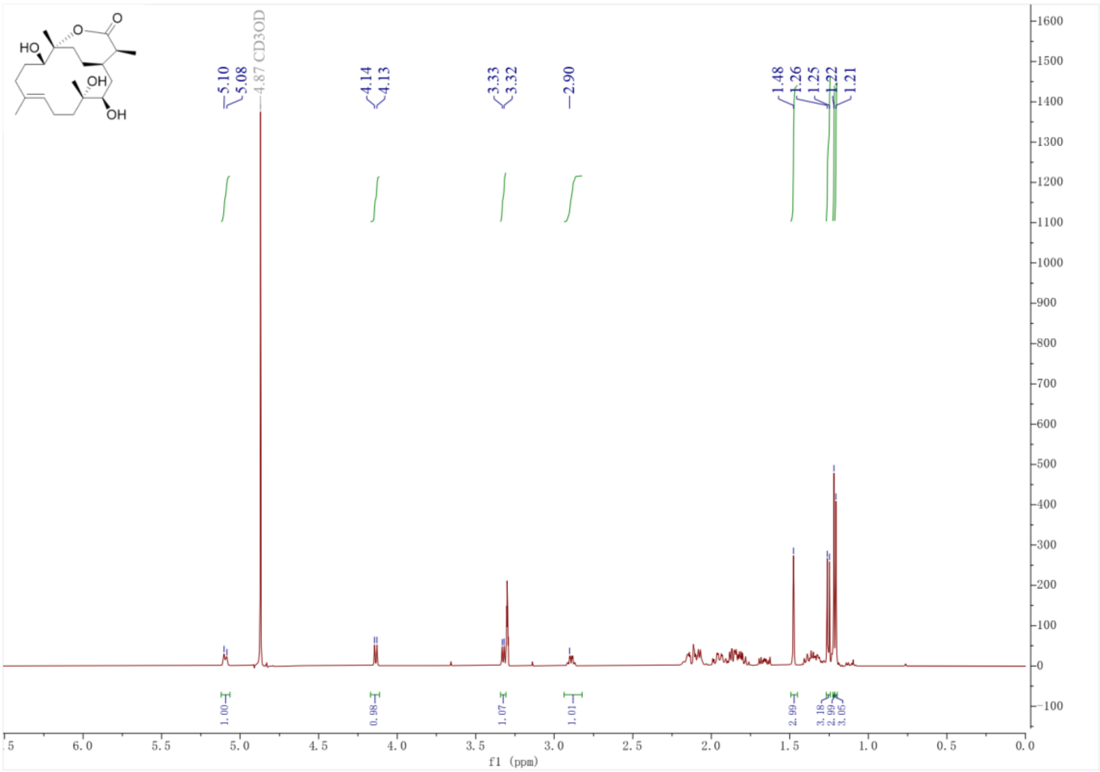
HO
O
O
OH
OH
5.10
5.08
4.87 CD3OD
4.14
4.13
3.33
3.32
2.90
1.48
1.26
1.25
1.22
1.21
1.00
0.98
1.07
1.01
2.99
3.18
2.99
3.05
f1 (ppm)

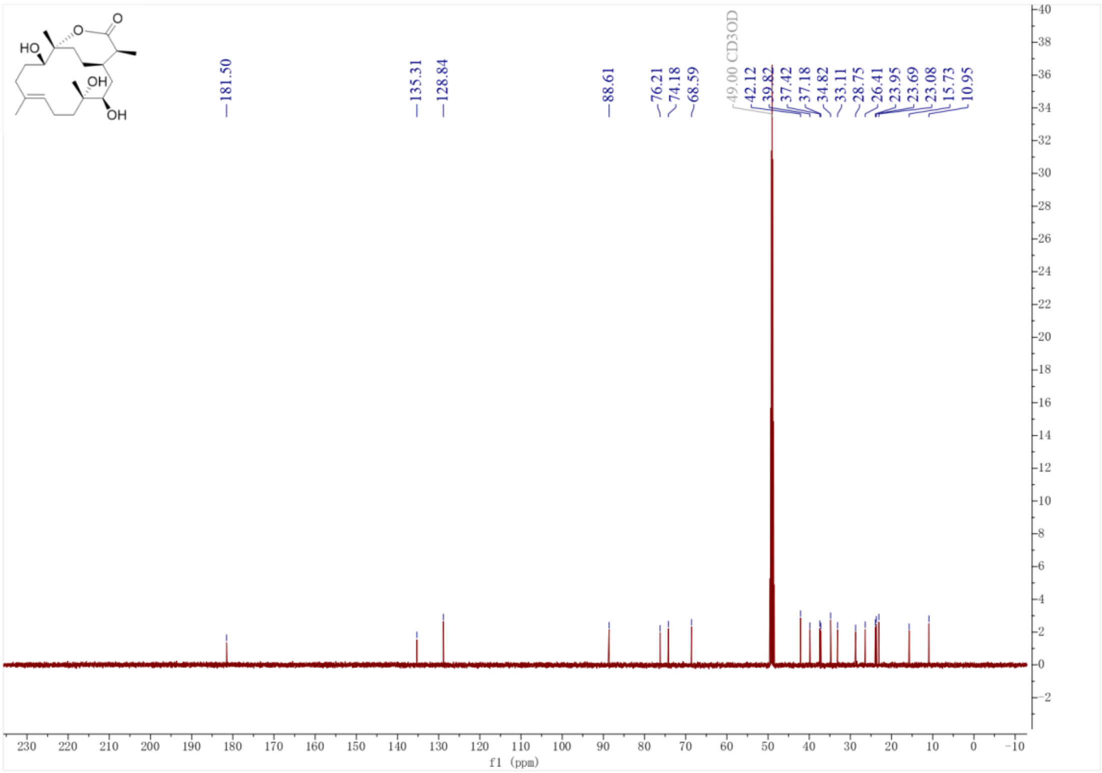
HO
O
O
OH
OH
181.50
135.31
128.84
88.61
76.21
74.18
68.59
49.00 CD3OD
42.12
39.82
37.42
37.18
34.82
33.11
28.75
26.41
23.95
23.69
23.08
15.73
10.95
f1 (ppm)

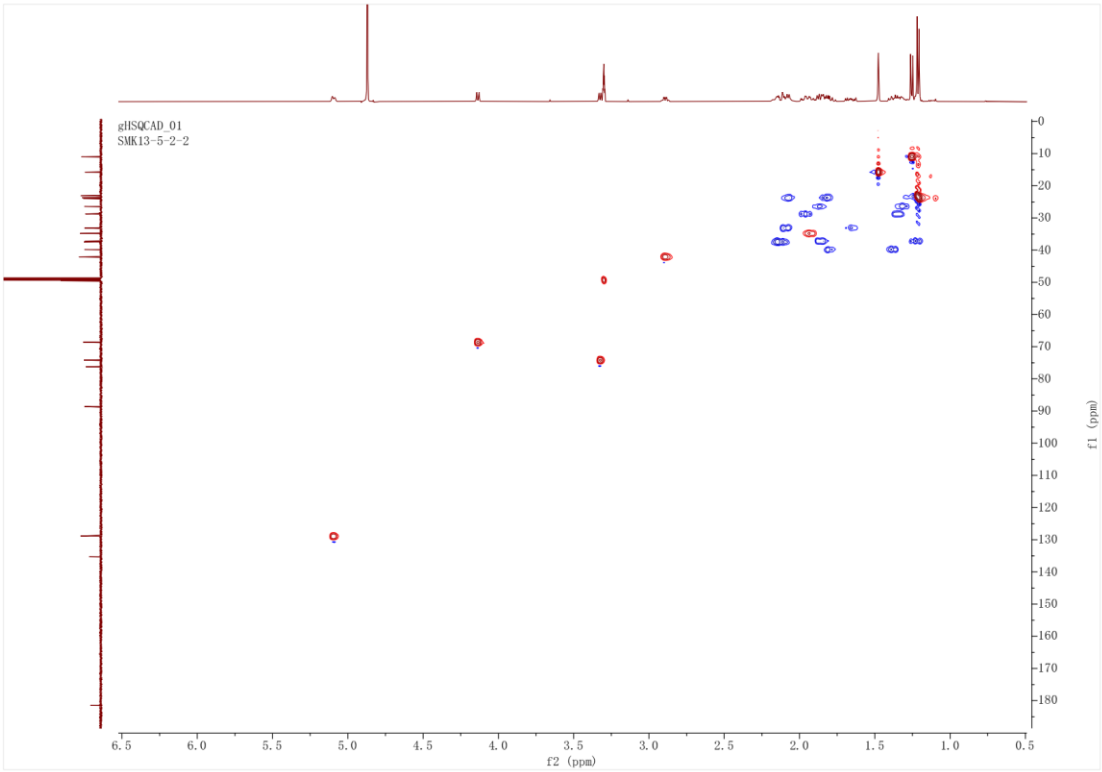
gHSQCAD_01
SMK13-5-2-2
f2 (ppm)
f1 (ppm)

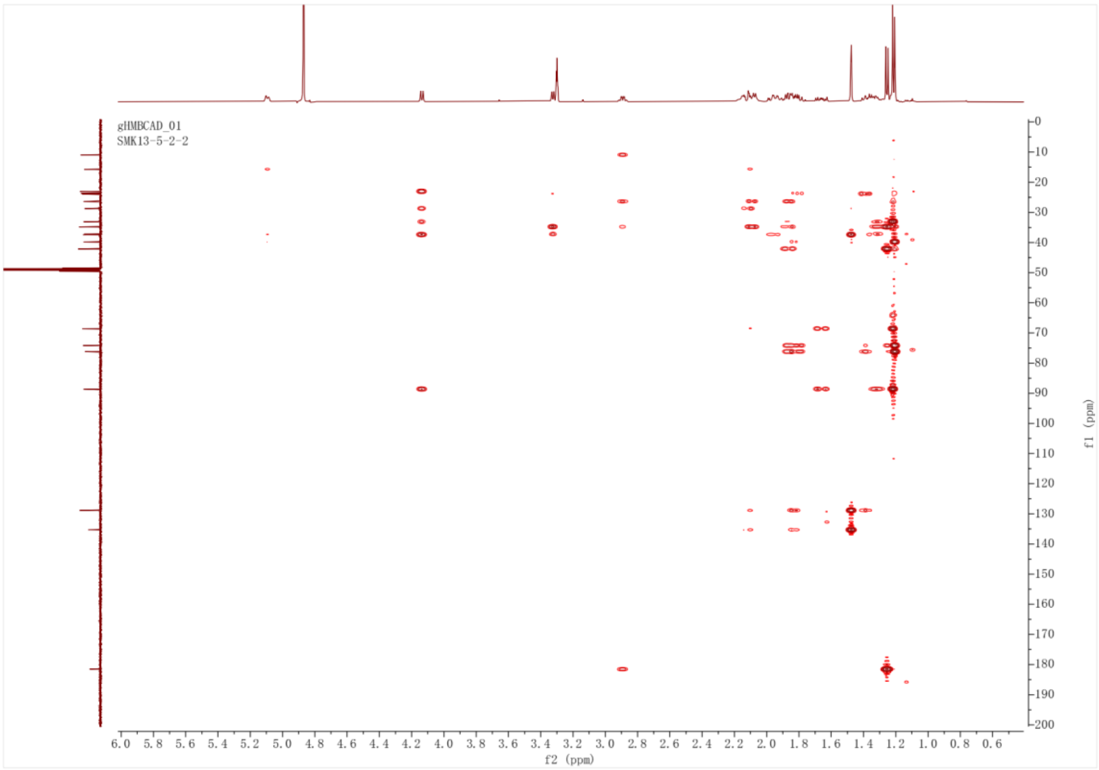
gHMBCAD_01
SMK13-5-2-2
f2 (ppm)
f1 (ppm)

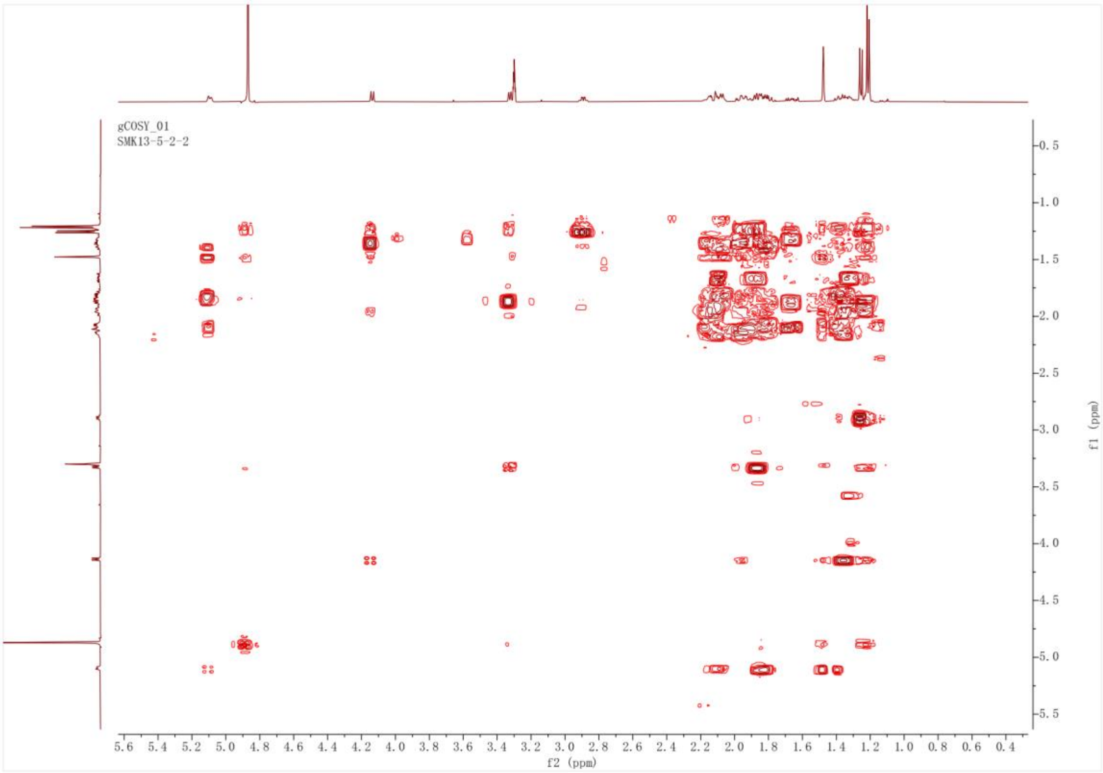
gCOSY_01
SMK13-5-2-2
f1 (ppm)
f2 (ppm)

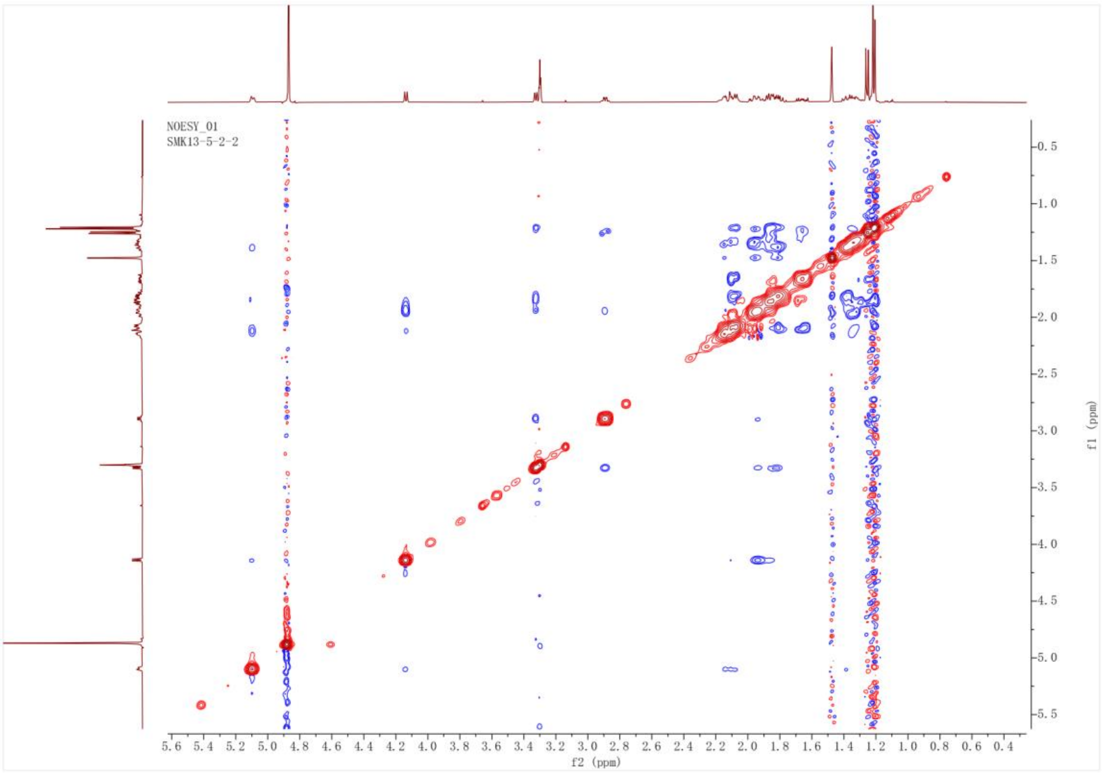
NOESY_01
SMK13-5-2-2
f1 (ppm)
f2 (ppm)

Figure.S8 Spectra (HRESIMS, UV, ^{1}H NMR, ^{13}C NMR, HSQC, HMBC, ^{1}H–^{1}H COSY, NOESY) for compound 3

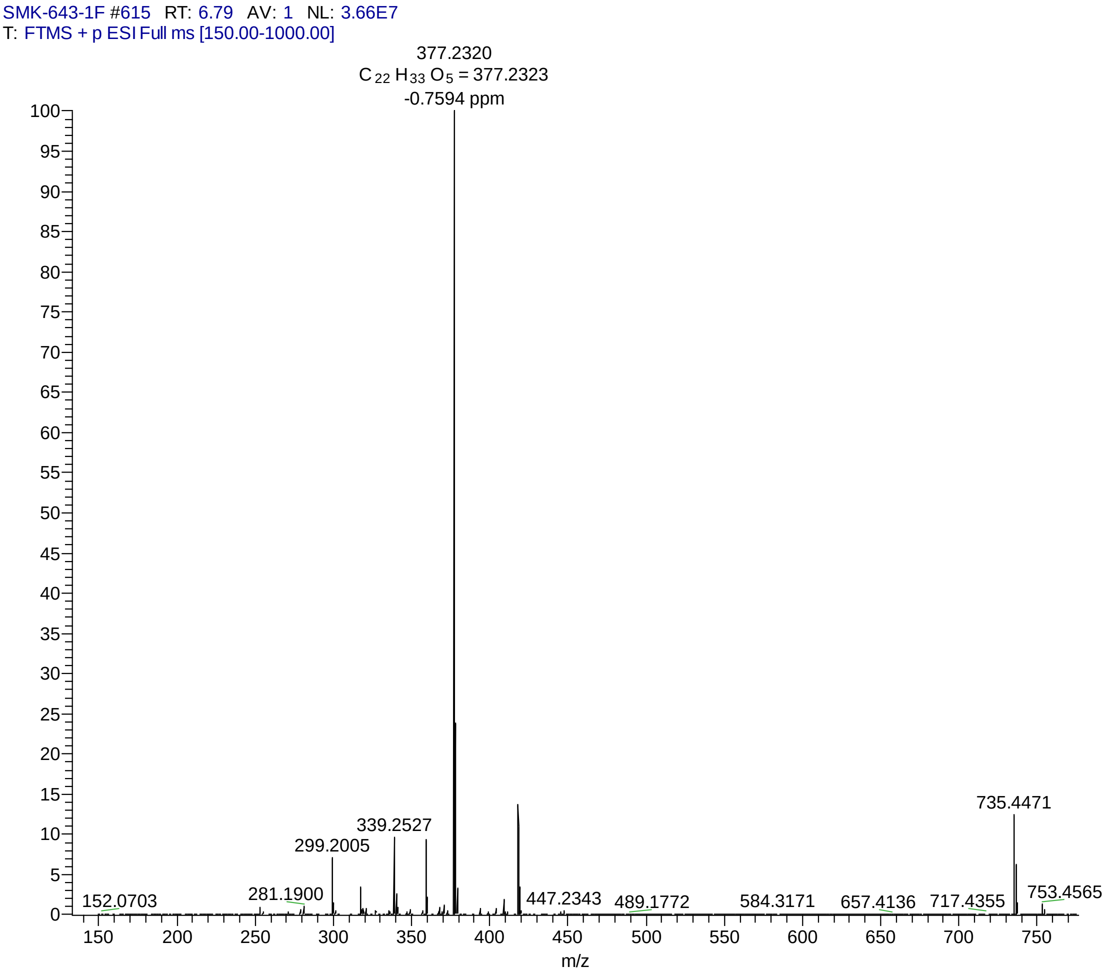

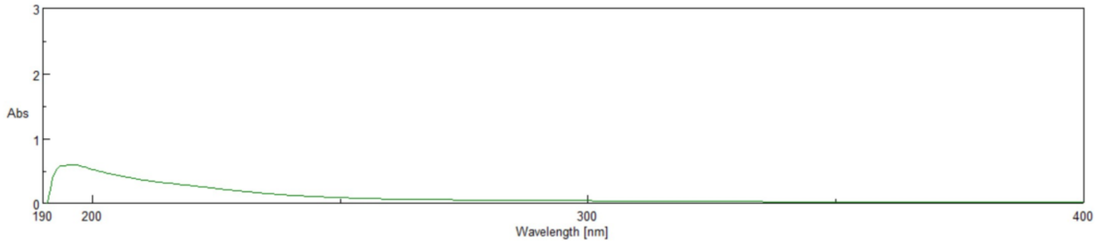

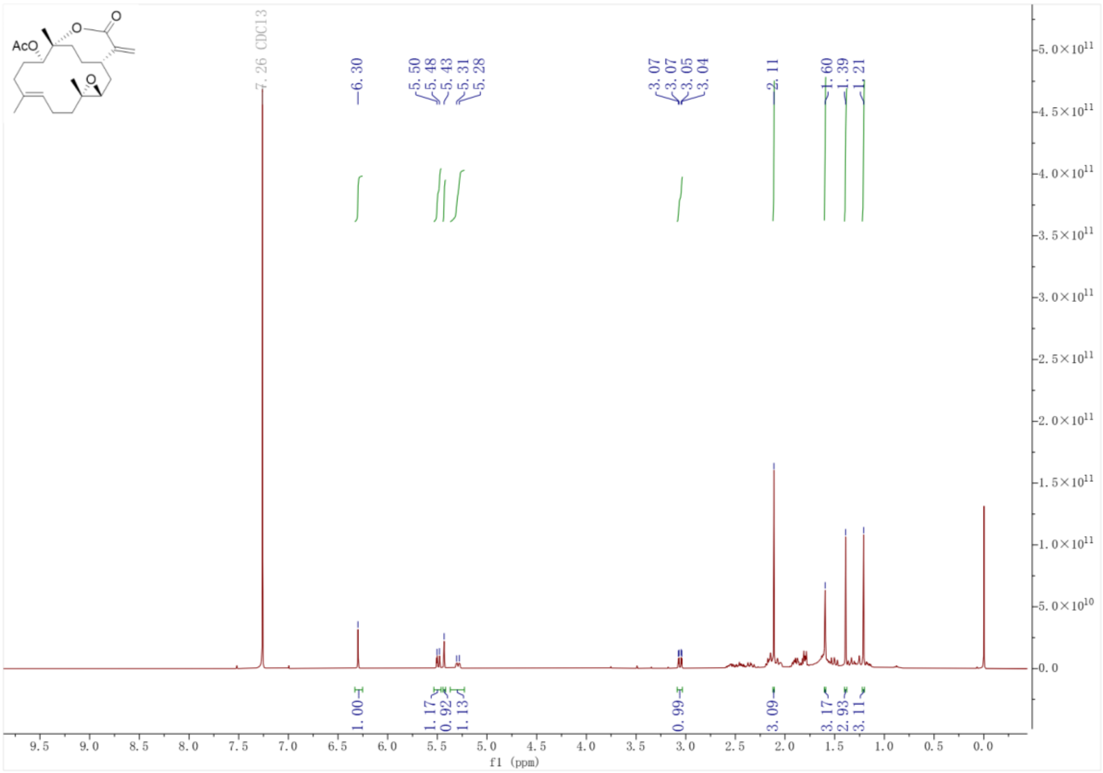
AcO
7.26 CDCl3
6.30
5.50
5.48
5.43
5.31
5.28
3.07
3.07
3.05
3.04
2.11
1.60
1.39
1.21
1.00
1.17
0.92
1.13
0.99
3.09
3.17
2.93
3.11
f1 (ppm)

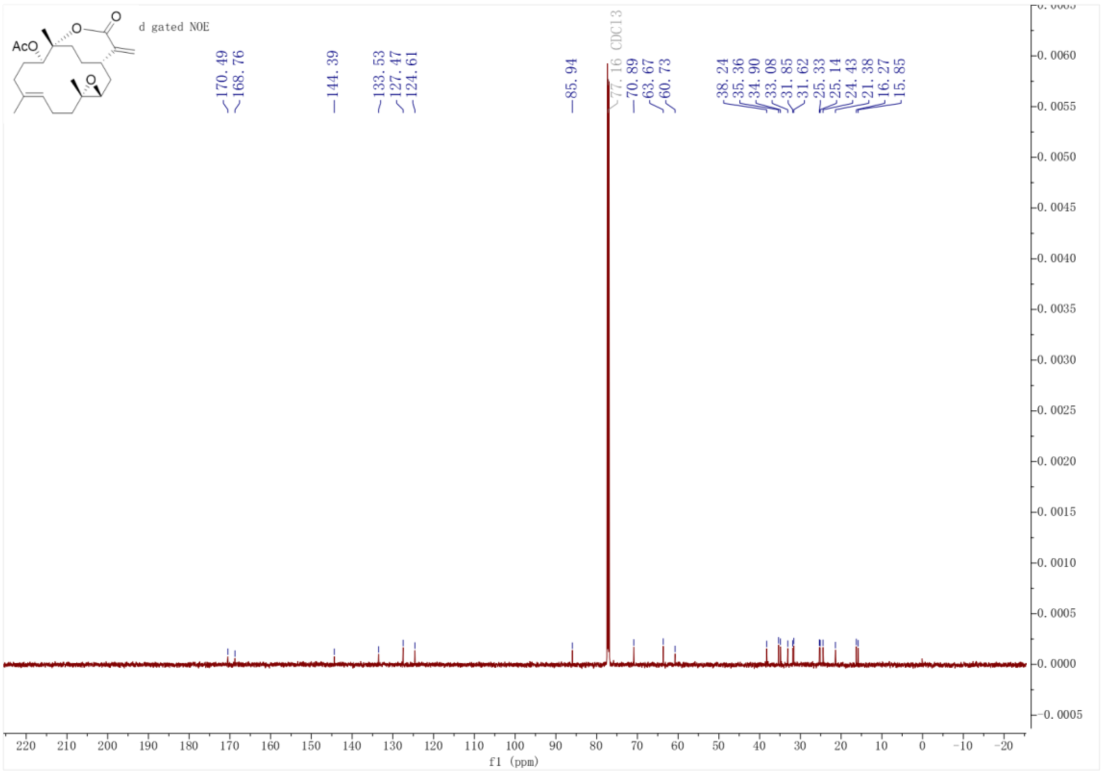
AcO
d gated NOE
170.49
168.76
144.39
133.53
127.47
124.61
85.94
77.16 CDCl3
70.89
63.67
60.73
38.24
35.36
34.90
33.08
31.85
31.62
25.33
25.14
24.43
21.38
16.27
15.85
f1 (ppm)

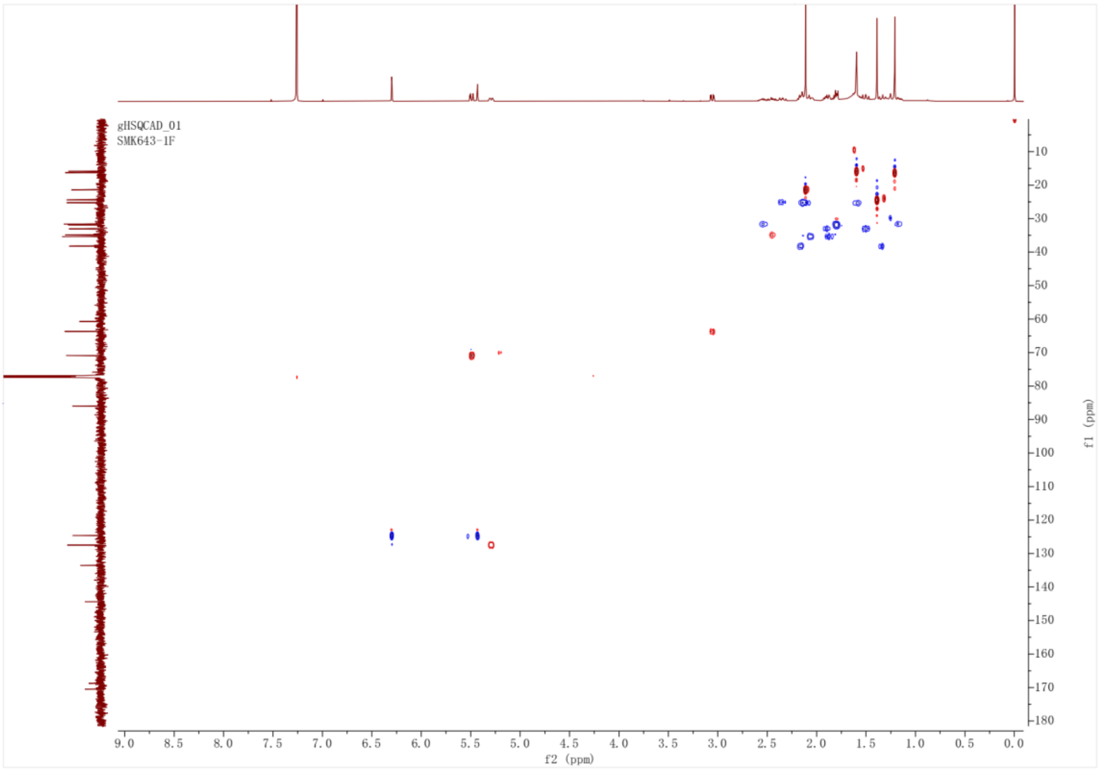
gHSQCAD_01
SMK643-1F
f2 (ppm)
f1 (ppm)

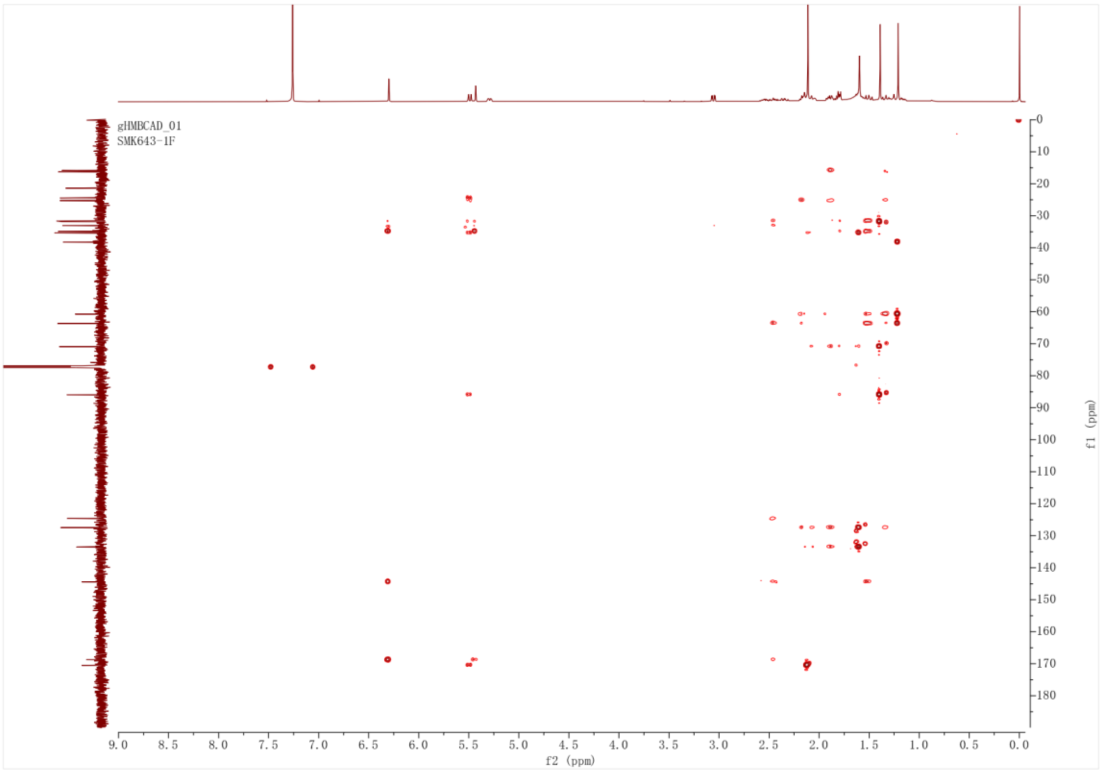
gHMBCAD_01
SMK643-1F
f2 (ppm)
f1 (ppm)

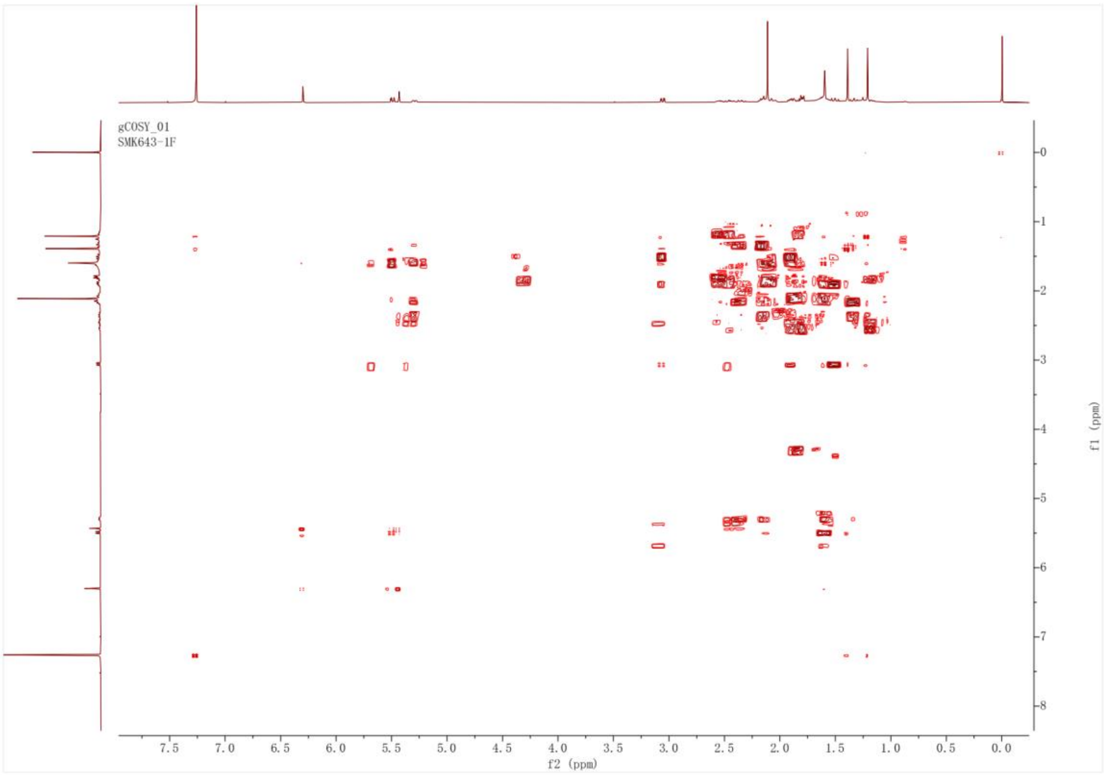
gCOSY_01
SMK643-1F
f2 (ppm)
f1 (ppm)

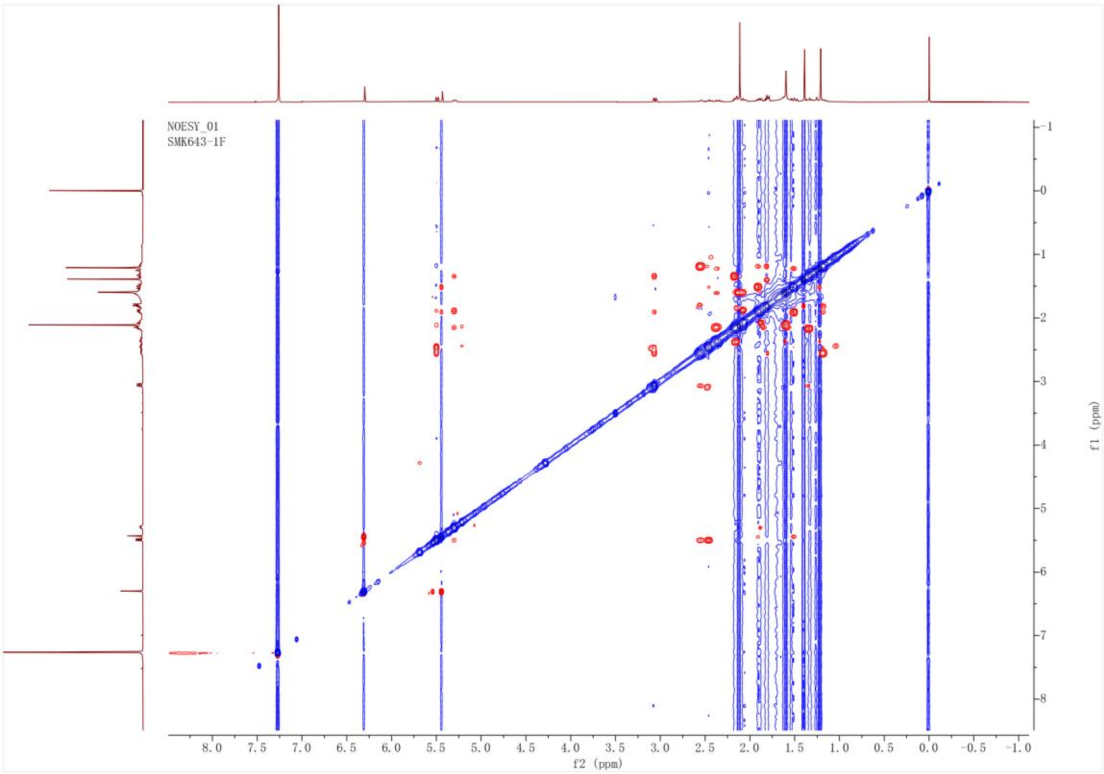
NOESY_01
SMK643-1F
f2 (ppm)
f1 (ppm)

Figure.S9 Spectra (HRESIMS, UV, ^{1}H NMR, ^{13}C NMR, HSQC, HMBC, ^{1}H–^{1}H COSY, NOESY) for compound 4

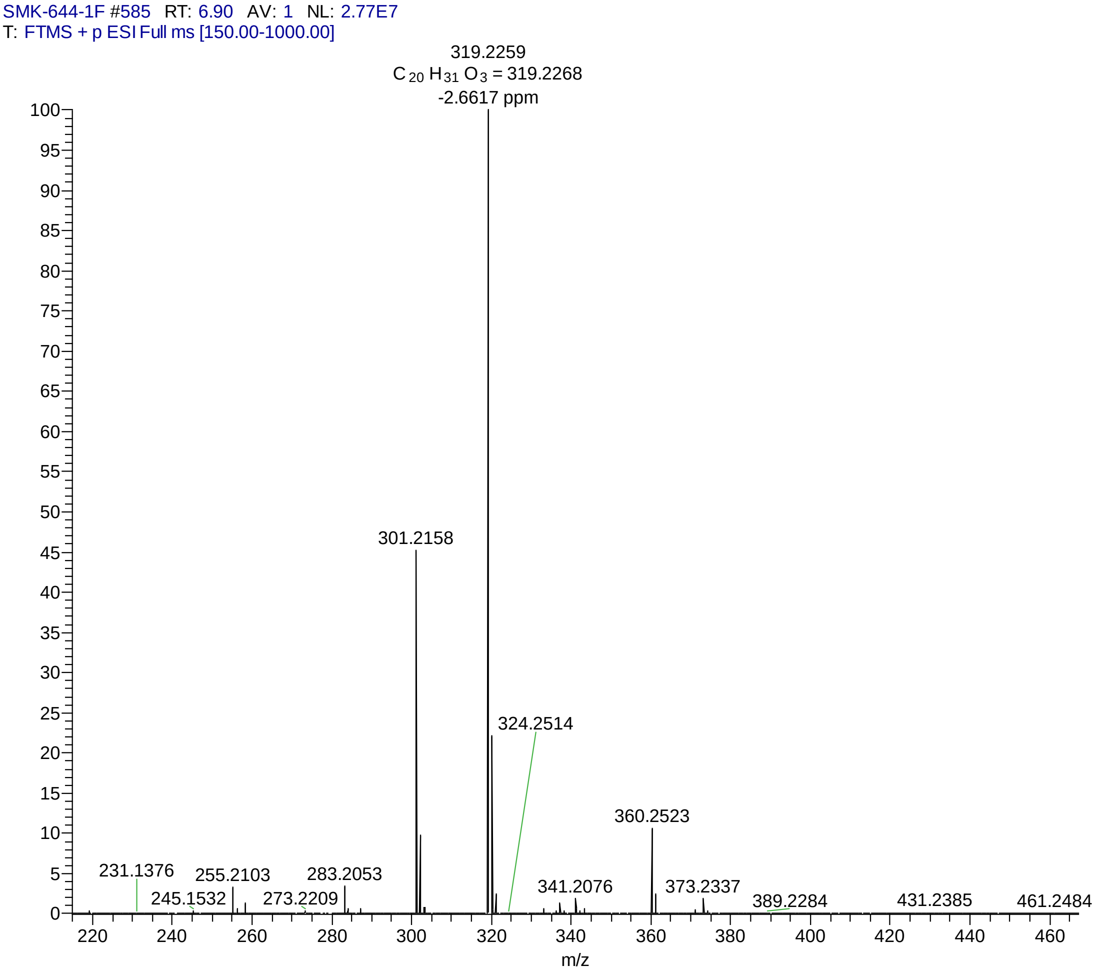

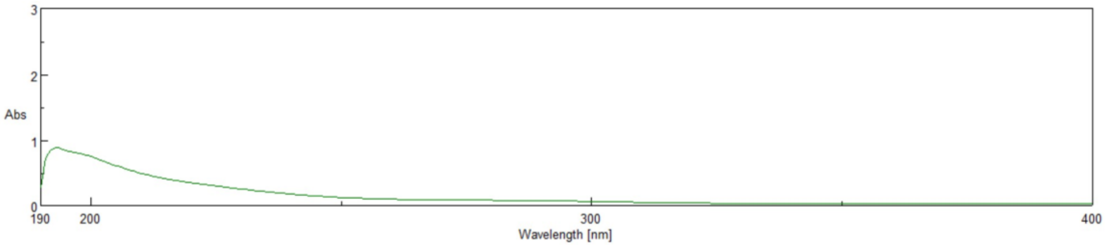

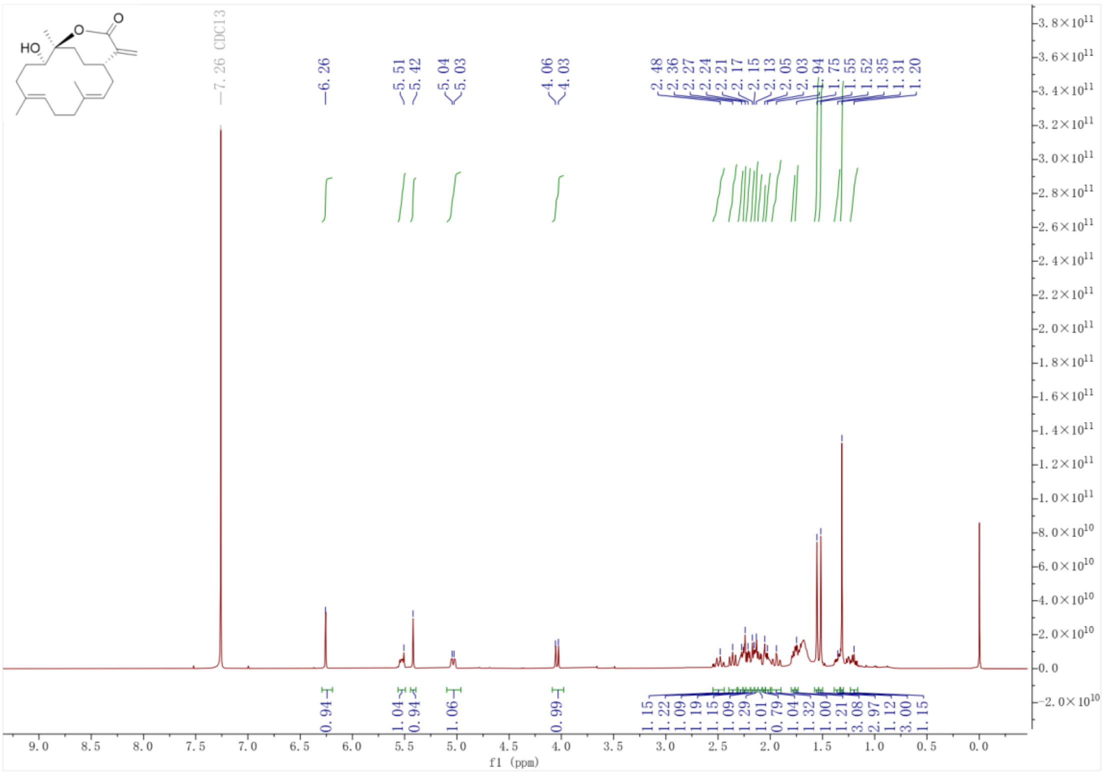
HO
O
O
7.26 CDCl3
6.26
5.51
5.42
5.04
5.03
4.06
4.03
2.48
2.36
2.27
2.24
2.21
2.17
2.15
2.13
2.05
2.03
1.94
1.75
1.55
1.52
1.35
1.31
1.20
0.94
1.04
0.94
1.06
0.99
1.15
1.22
1.09
1.19
1.15
1.09
1.29
1.01
0.79
1.04
1.32
1.00
1.21
3.08
2.97
1.12
3.00
1.15
f1 (ppm)

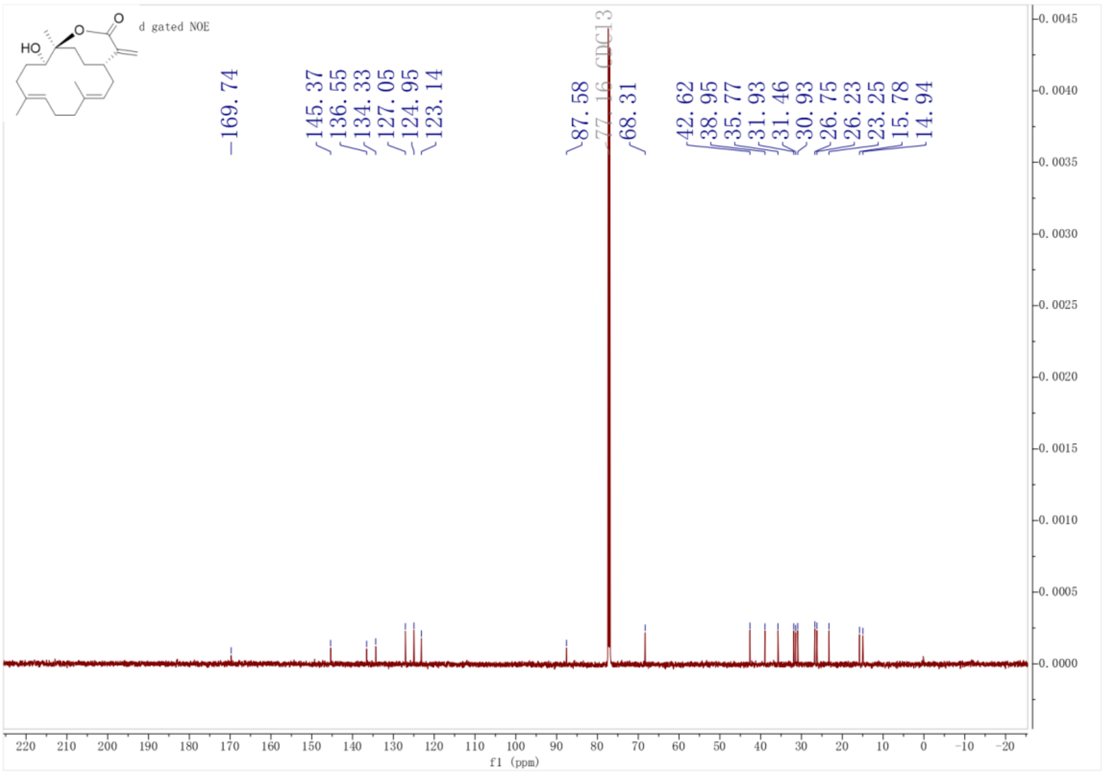
HO
O
O
d gated NOE
169.74
145.37
136.55
134.33
127.05
124.95
123.14
87.58
77.16 CDCl3
68.31
42.62
38.95
35.77
31.93
31.46
30.93
26.75
26.23
23.25
15.78
14.94
f1 (ppm)

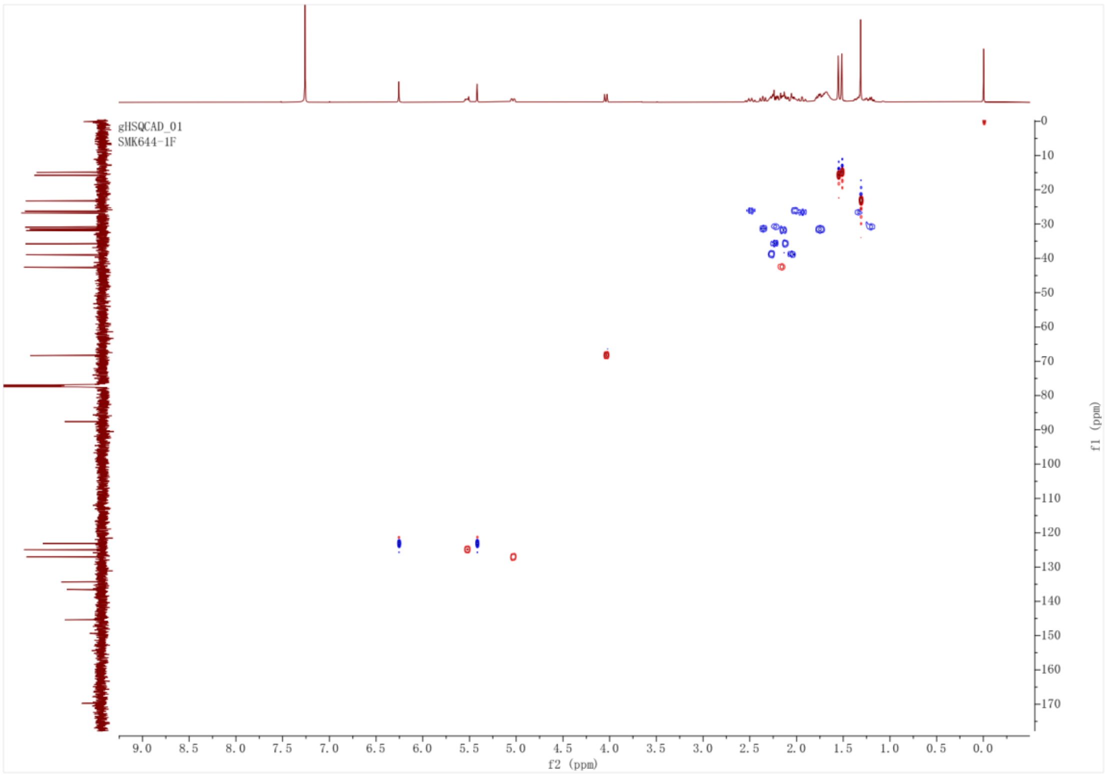
gHSQCAD_01
SMK644-1F
f2 (ppm)
f1 (ppm)

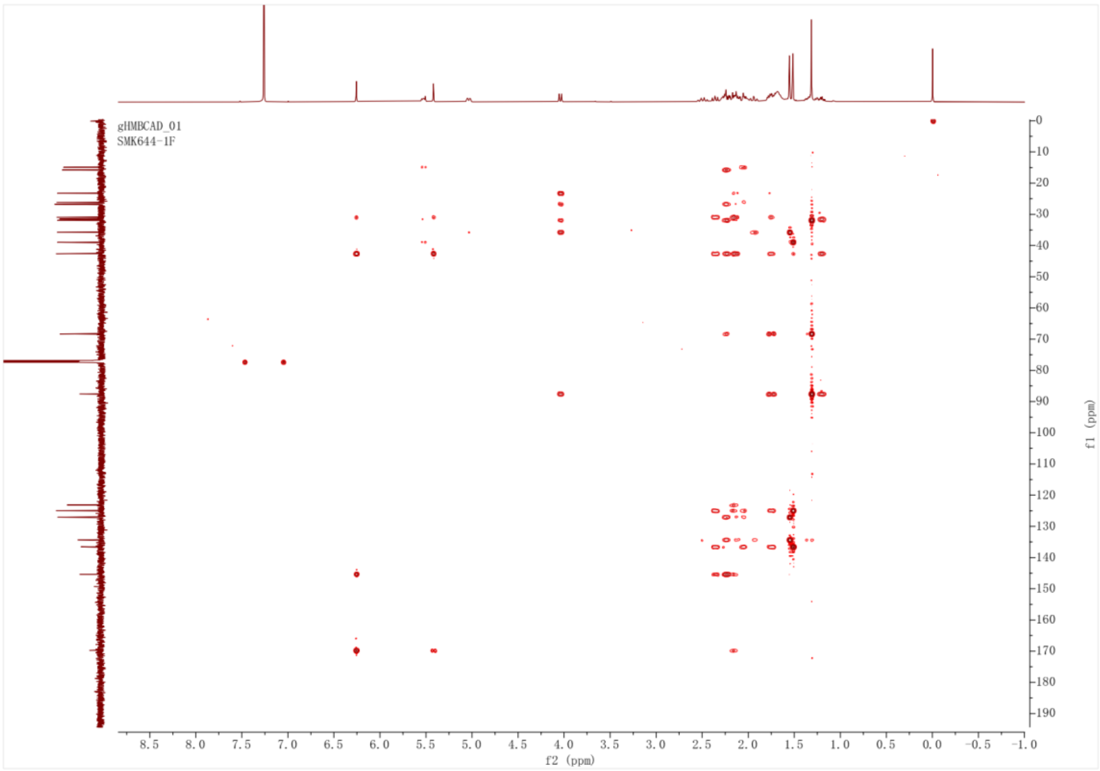
gHMBCAD_01
SMK644-1F
f2 (ppm)
f1 (ppm)

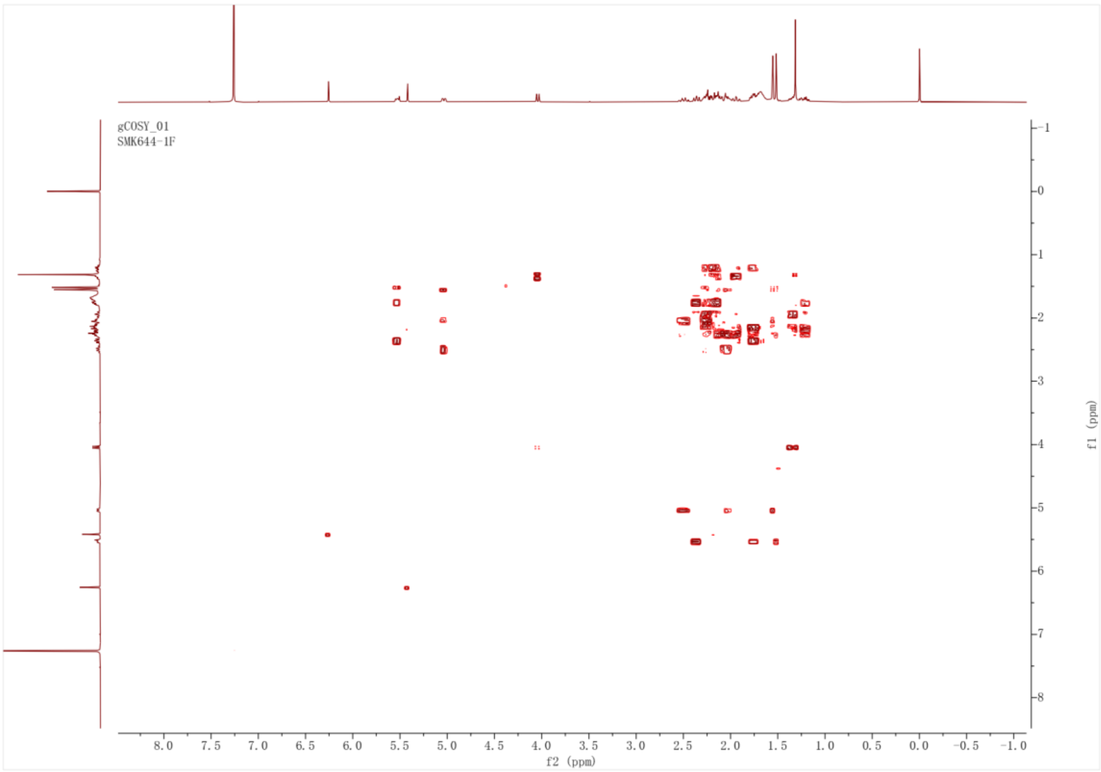
gCOSY_01
SMK644-1F
f2 (ppm)
f1 (ppm)

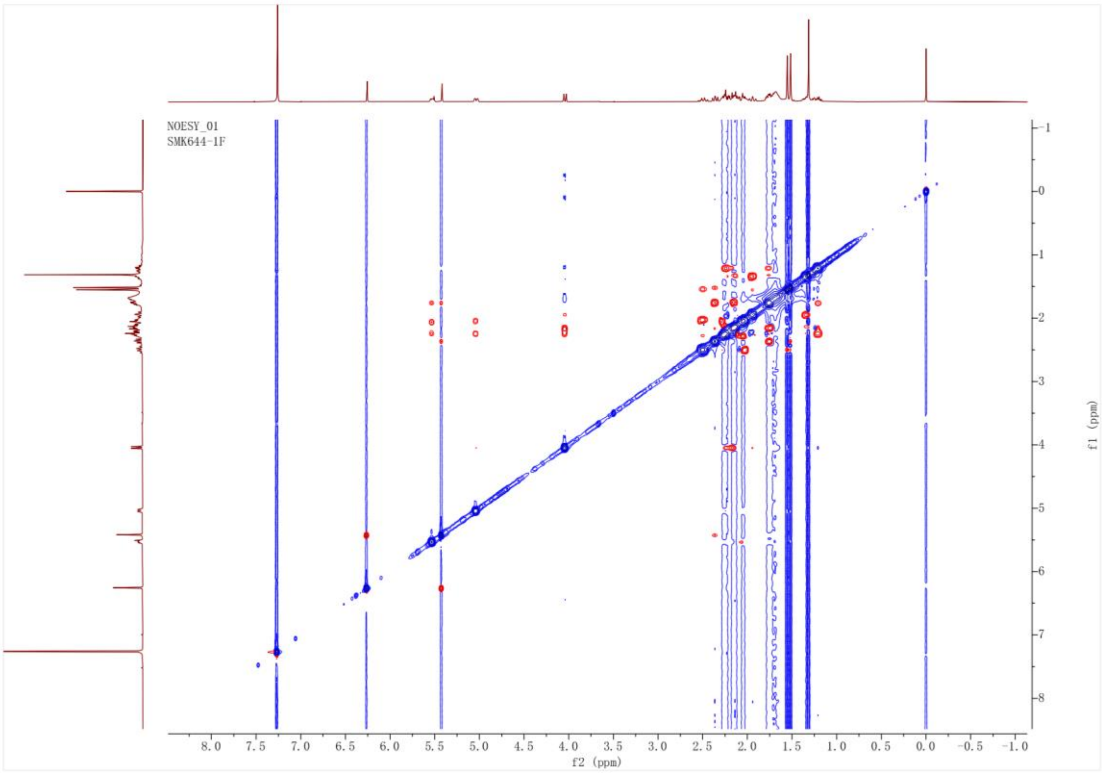
NOESY_01
SMK644-1F
f2 (ppm)
f1 (ppm)

Figure.S10 Spectra (HRESIMS, UV, ^{1}H NMR, ^{13}C NMR, HSQC, HMBC, ^{1}H–^{1}H COSY, NOESY) for compound 5

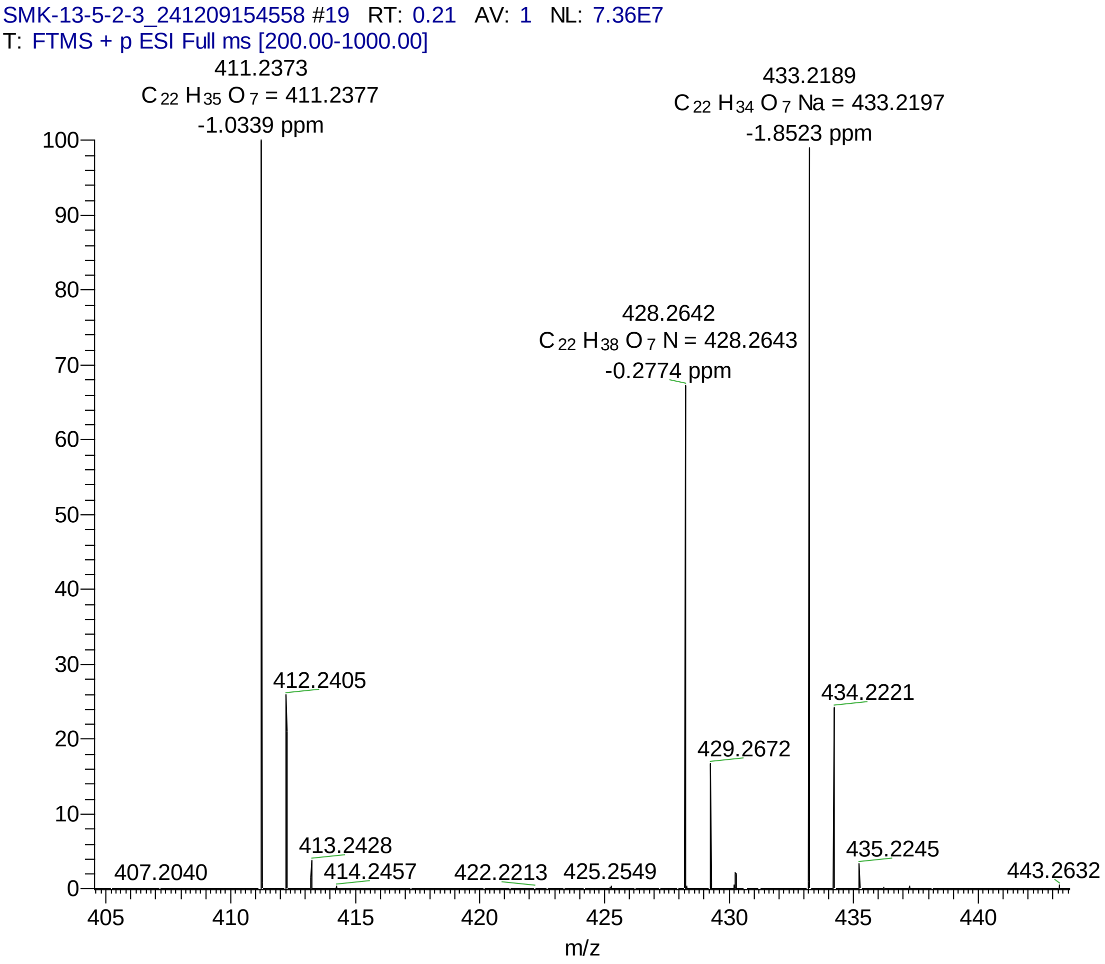

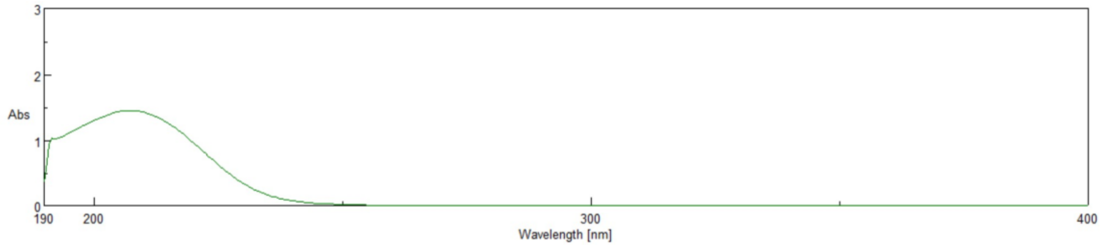

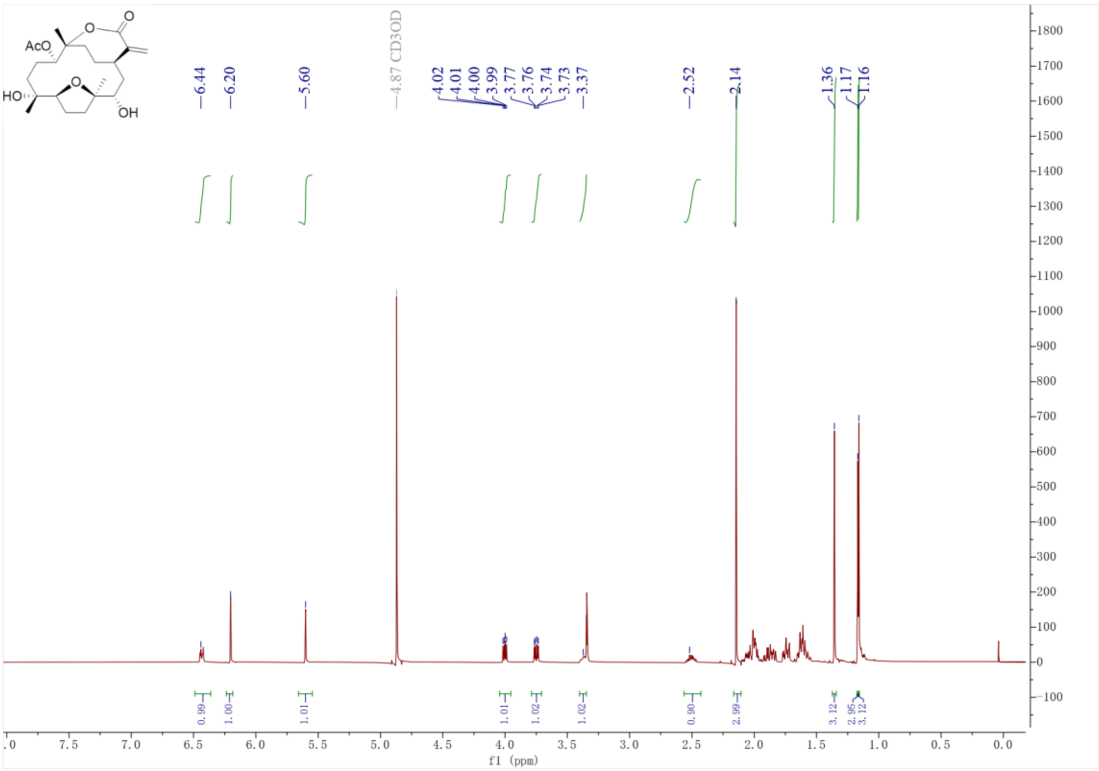
AcO
O
O
HO
O
OH
6.44
6.20
5.60
4.87 CD3OD
4.02
4.01
4.00
3.99
3.77
3.76
3.74
3.73
3.37
2.52
2.14
1.36
1.17
1.16
0.99
1.00
1.01
1.01
1.02
1.02
0.90
2.99
3.12
2.95
3.12
f1 (ppm)

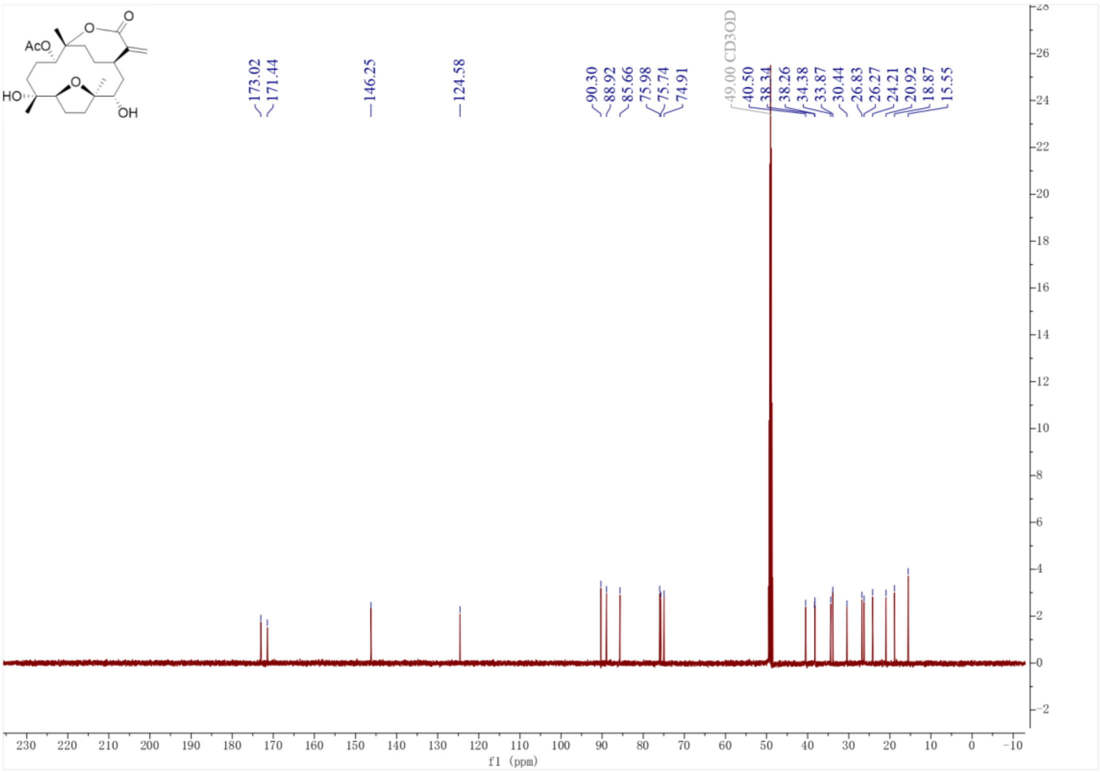
AcO
O
O
HO
O
OH
173.02
171.44
146.25
124.58
90.30
88.92
85.66
75.98
75.74
74.91
49.00 CD3OD
40.50
38.34
38.26
34.38
33.87
30.44
26.83
26.27
24.21
20.92
18.87
15.55
f1 (ppm)

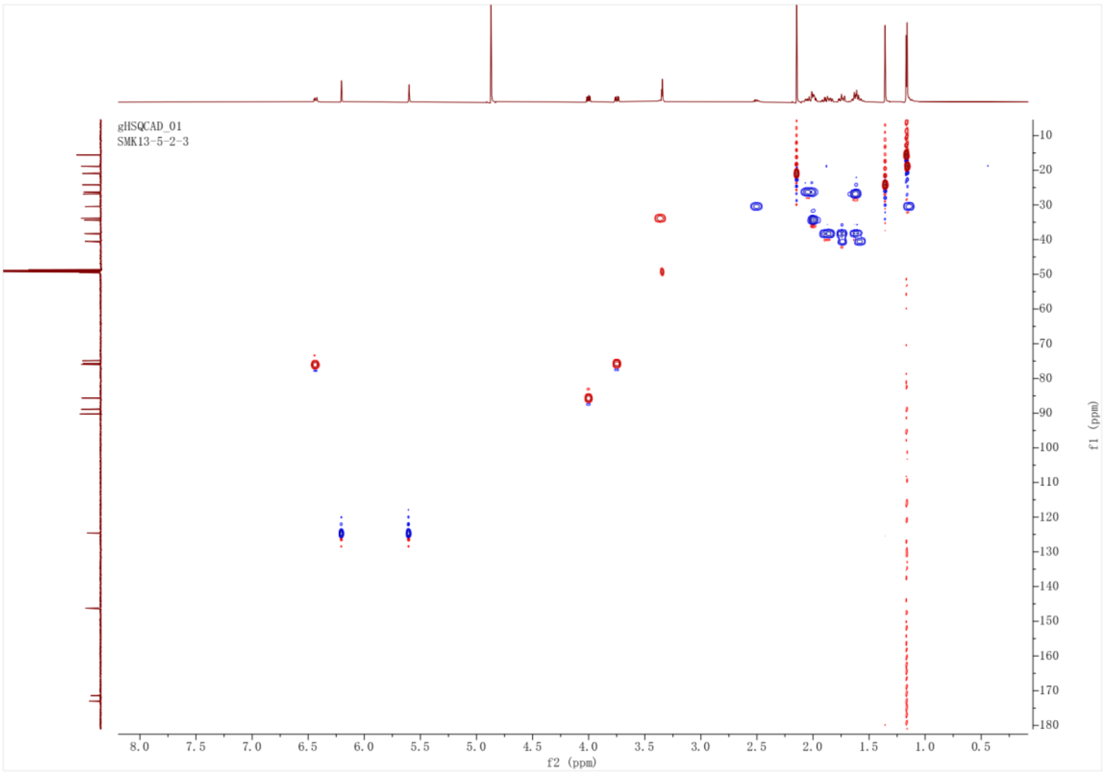
gHSQCAD_01
SMK13-5-2-3
f2 (ppm)
f1 (ppm)

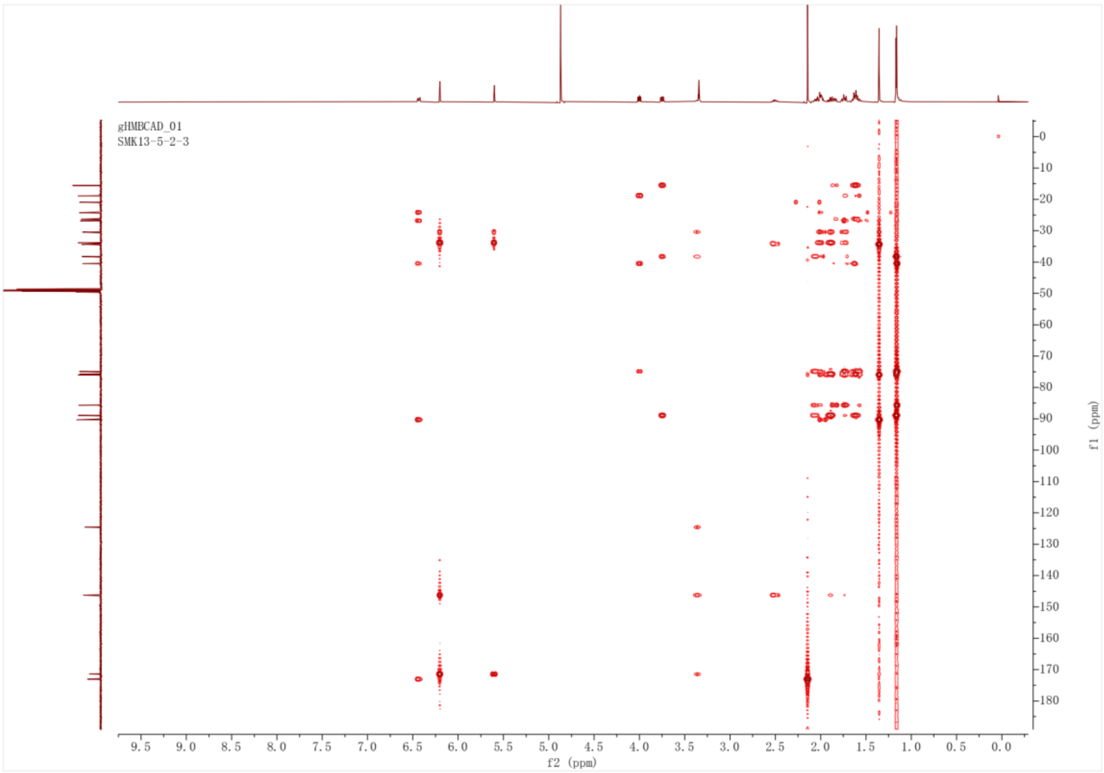
gHMBCAD_01
SMK13-5-2-3
f2 (ppm)
f1 (ppm)

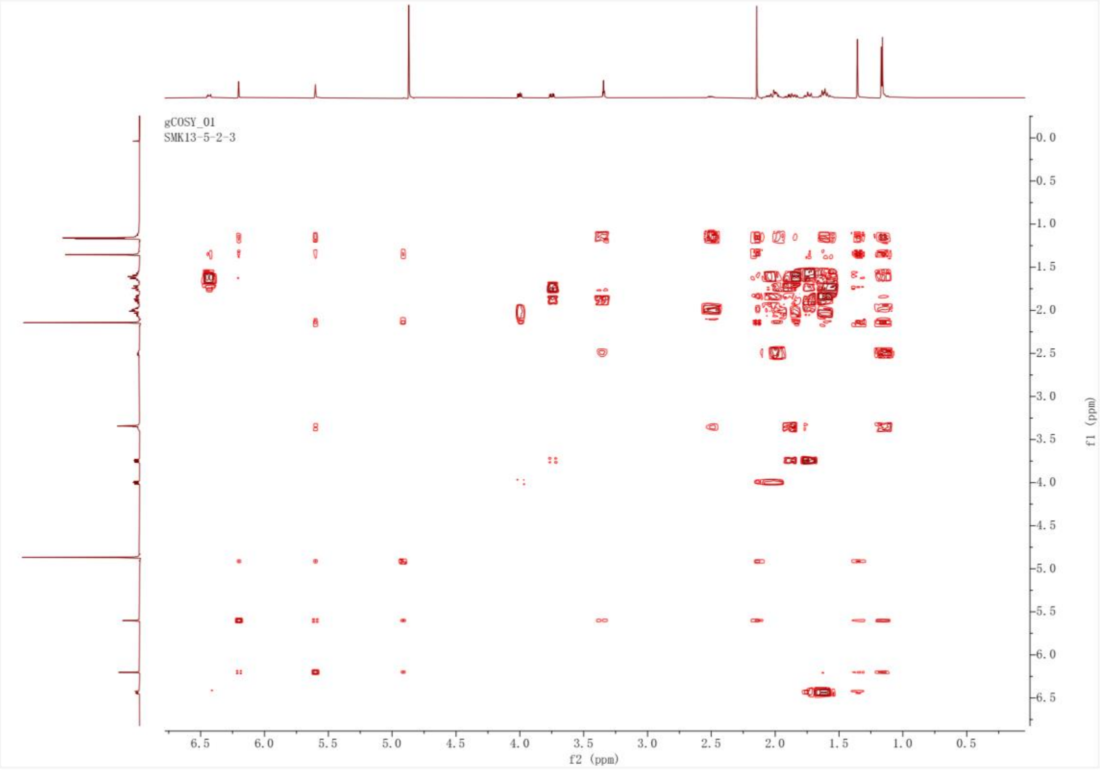
gCOSY_01
SMK13-5-2-3
f2 (ppm)
f1 (ppm)

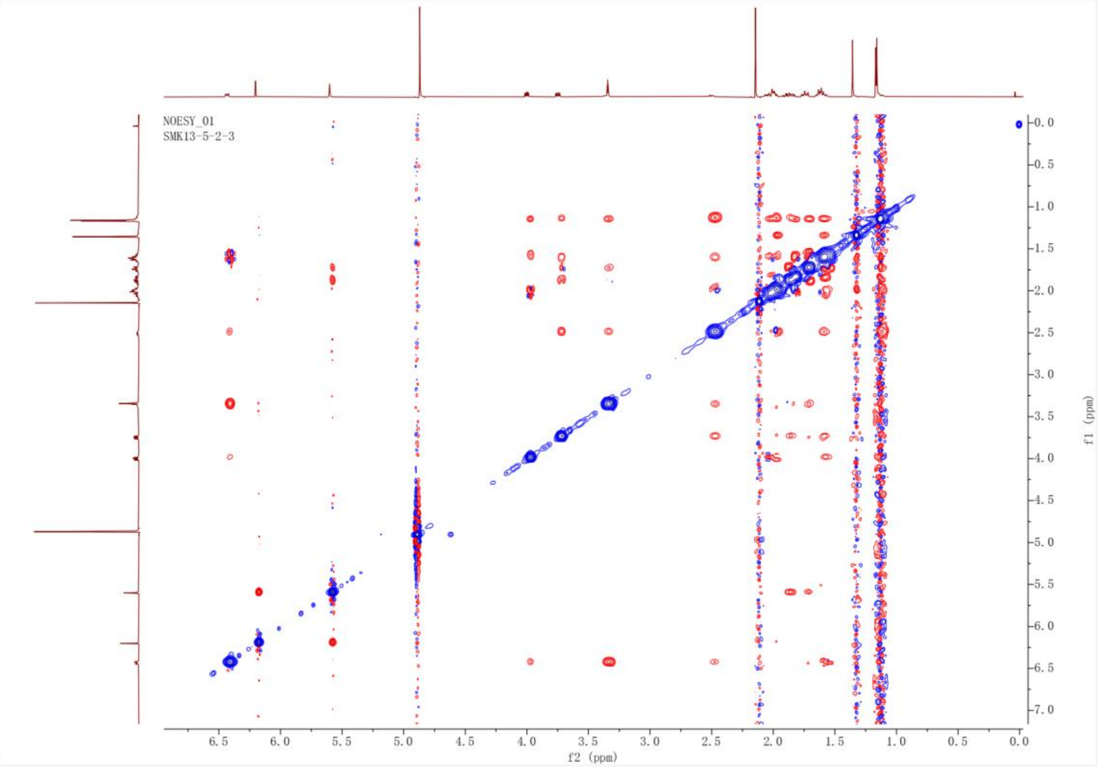
NOESY_01
SMK13-5-2-3
f2 (ppm)
f1 (ppm)

Figure.S11 Spectra (HRESIMS, UV, ^{1}H NMR, ^{13}C NMR, HSQC, HMBC, ^{1}H–^{1}H COSY, NOESY) for compound 6

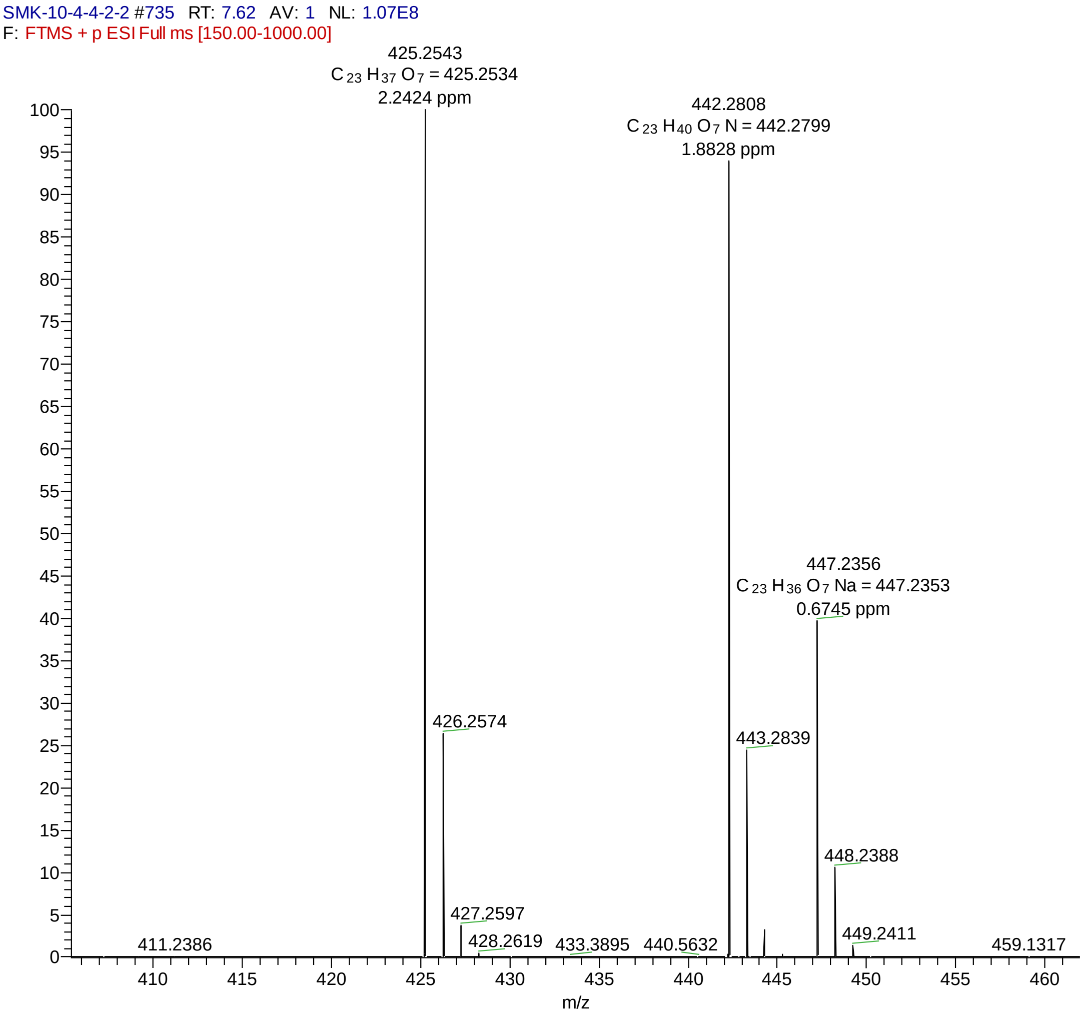

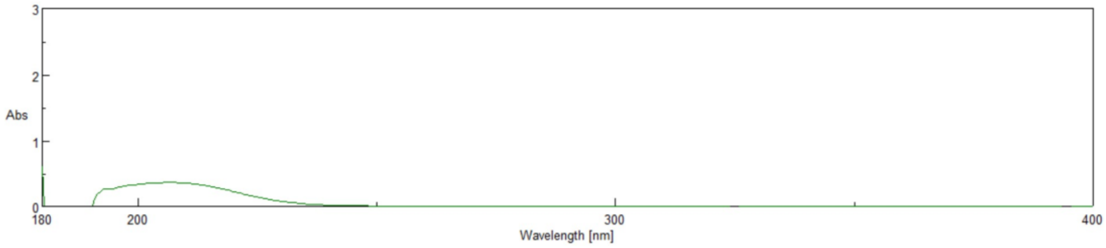

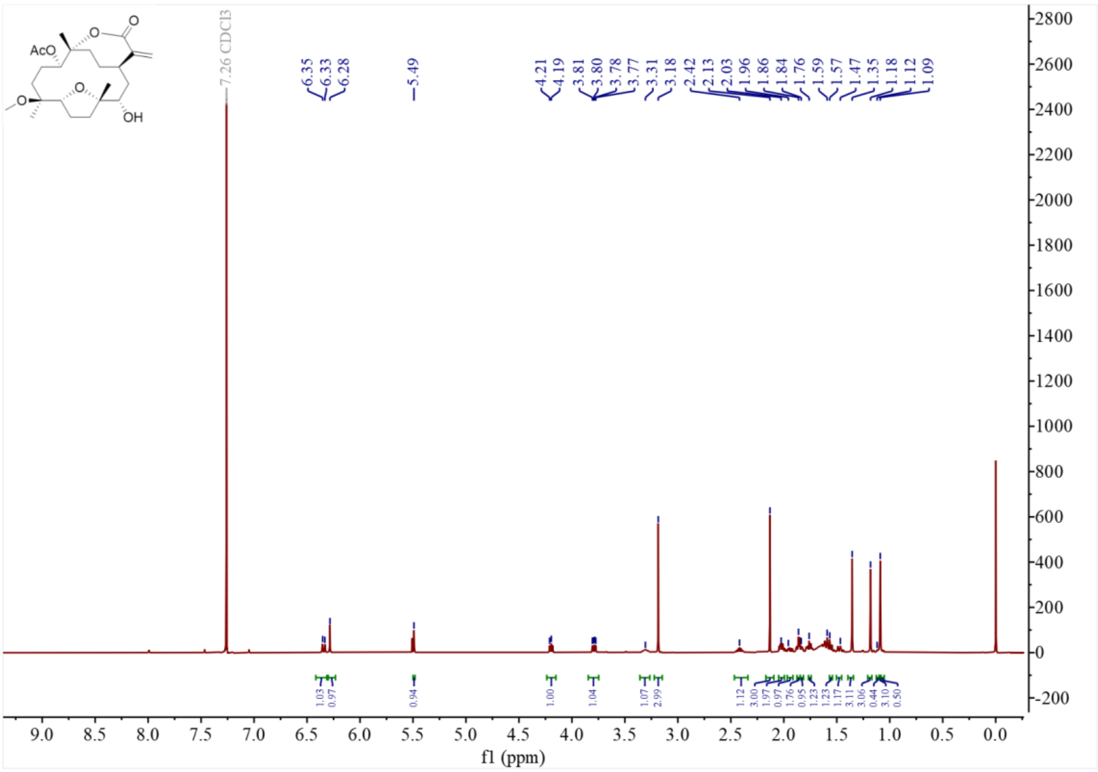

AcO
O
O
O
OH
7.26 CDCl3
6.35
6.33
6.28
5.49
4.21
4.19
3.81
3.80
3.78
3.77
3.31
3.18
2.42
2.13
2.03
1.96
1.86
1.84
1.76
1.59
1.57
1.47
1.35
1.18
1.12
1.09
f1 (ppm)

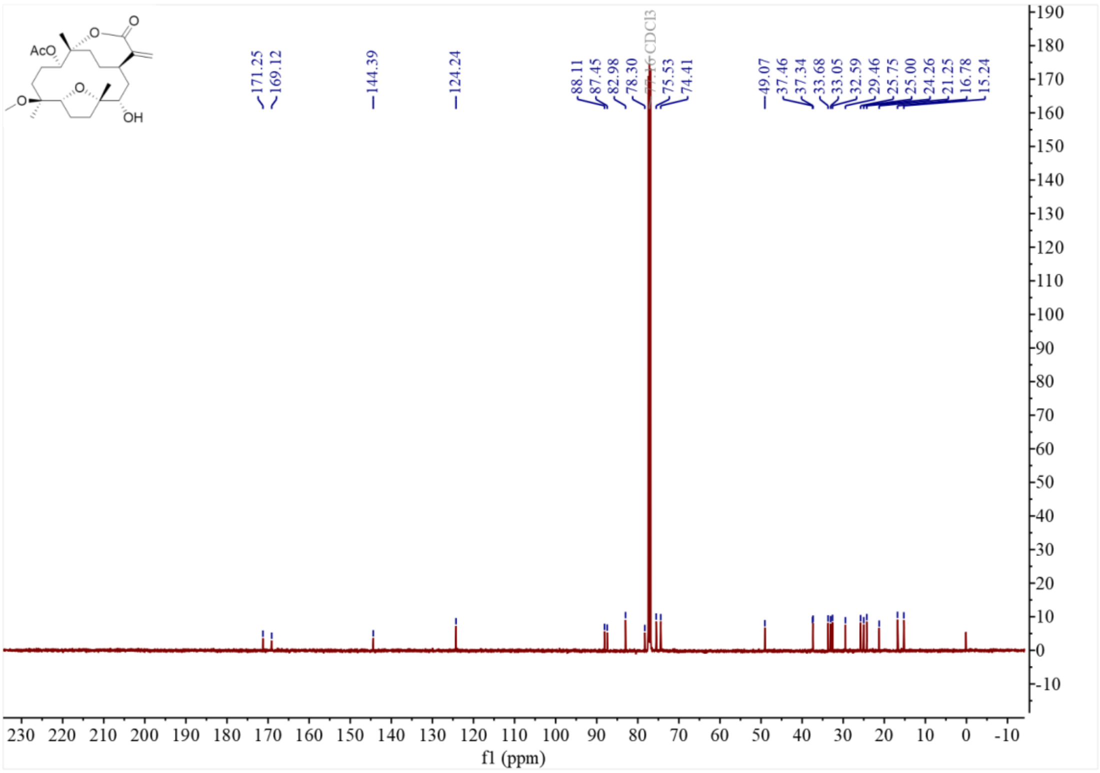

AcO
O
O
O
OH
171.25
169.12
144.39
124.24
88.11
87.45
82.98
78.30
75.53
74.41
49.07
37.46
37.34
33.68
33.05
32.59
29.46
25.75
25.00
24.26
21.25
16.78
15.24
f1 (ppm)

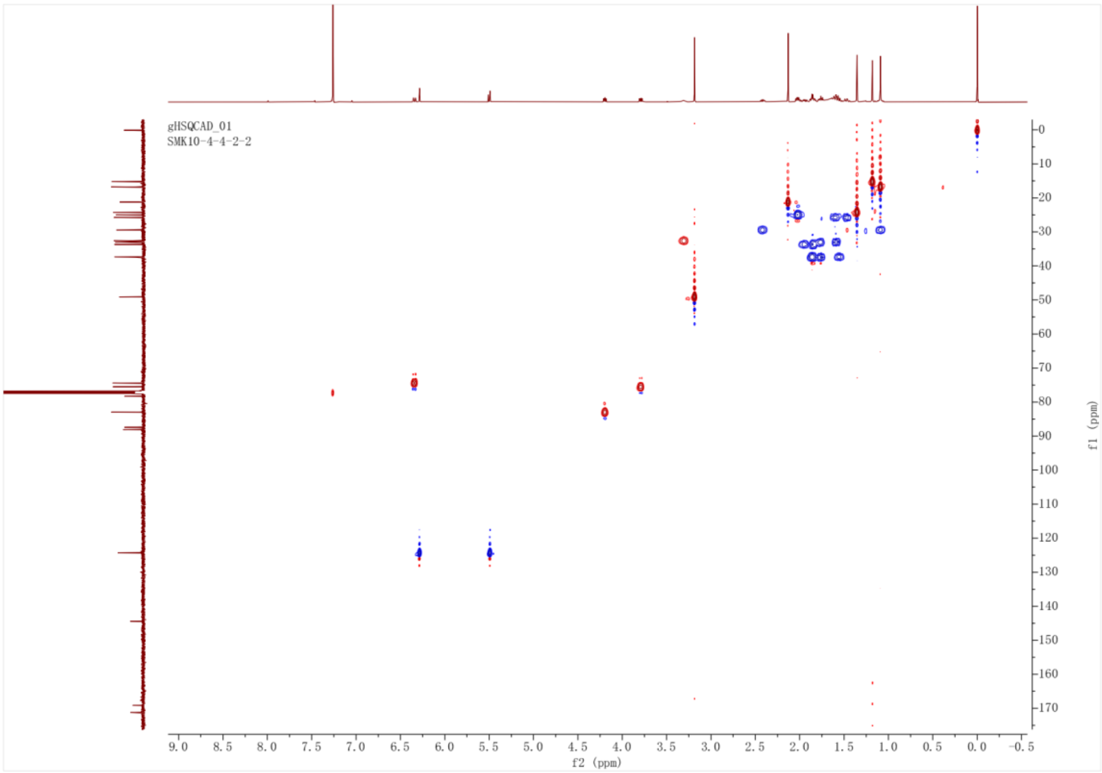
gHSQCAD_01
SMK10-4-4-2-2
f2 (ppm)
f1 (ppm)

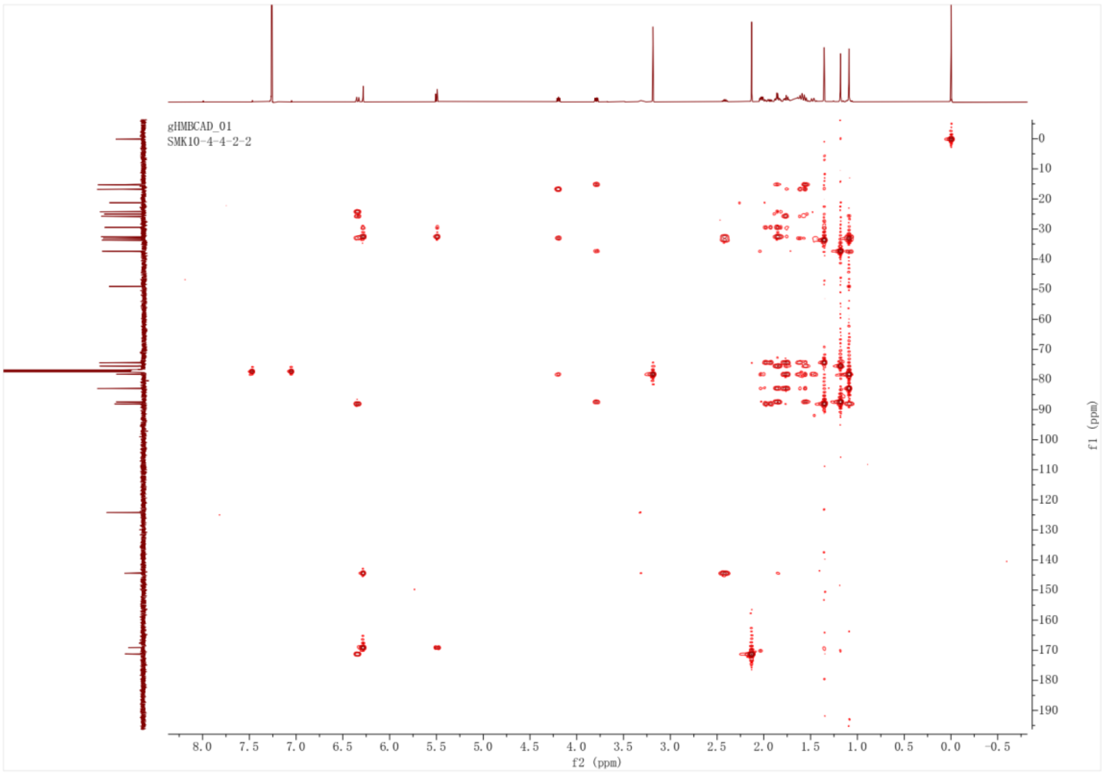
gHMBCAD_01
SMK10-4-4-2-2
f2 (ppm)
f1 (ppm)

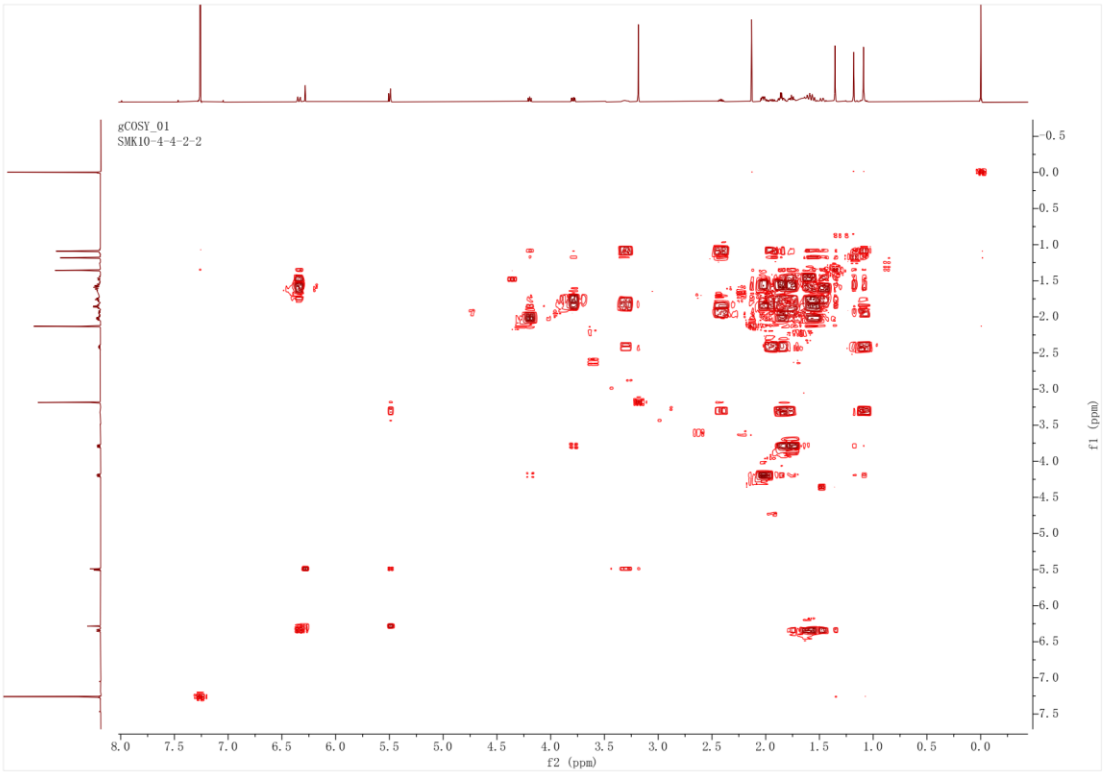
gCOSY_01
SMK10-4-4-2-2
f2 (ppm)
f1 (ppm)

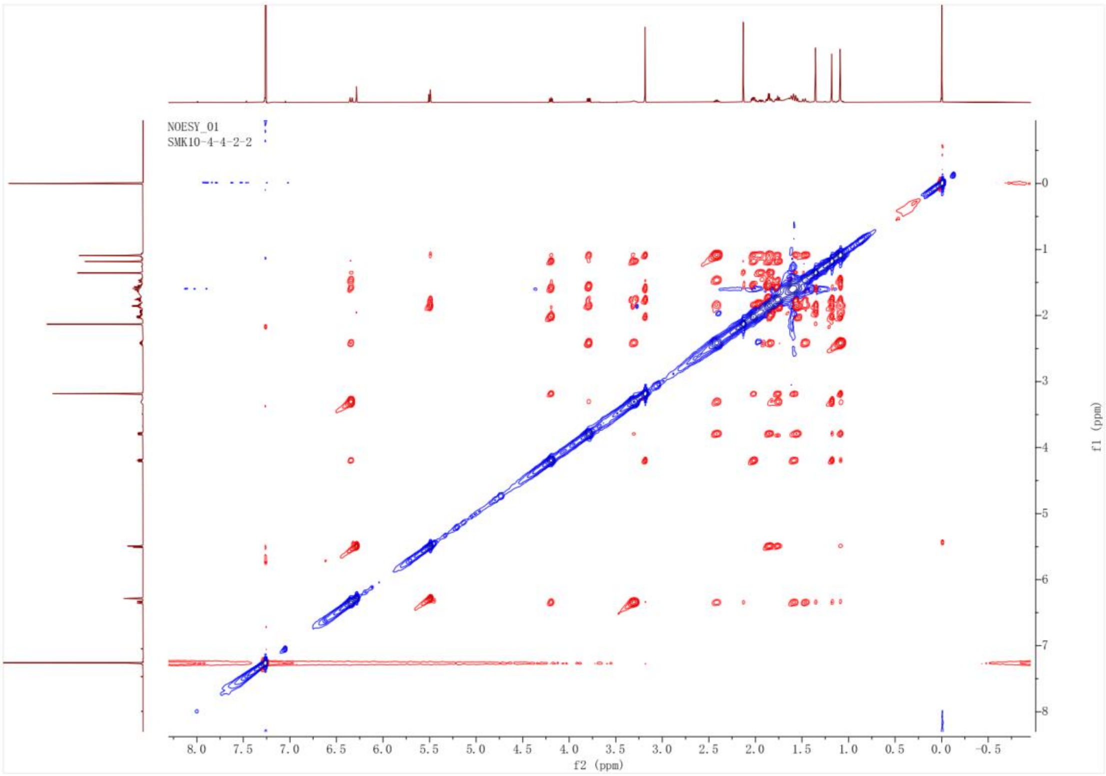
NOESY_01
SMK10-4-4-2-2
f2 (ppm)
f1 (ppm)

Figure.S12 Spectra (HRESIMS, UV, ^{1}H NMR, ^{13}C NMR, HSQC, HMBC, ^{1}H–^{1}H COSY, NOESY) for compound 7

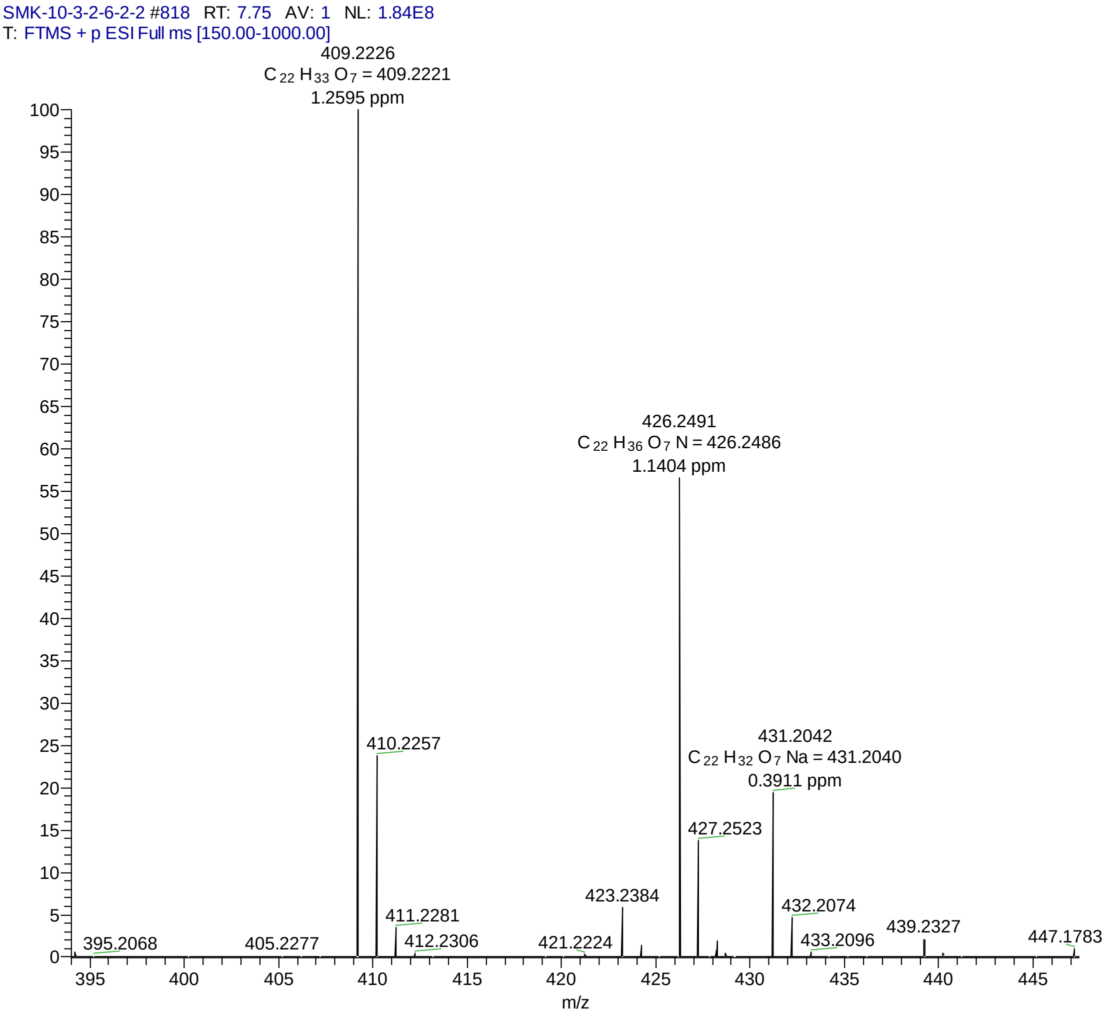

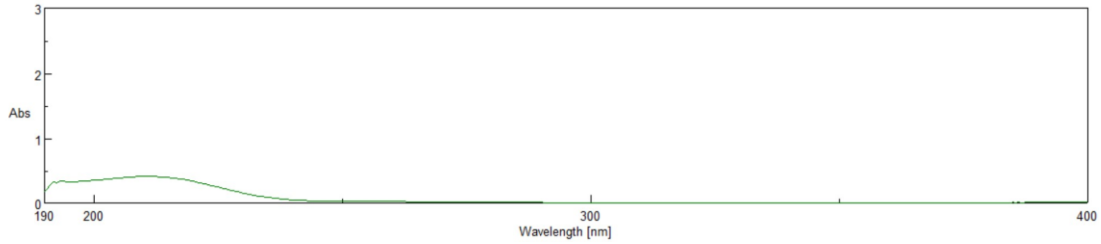

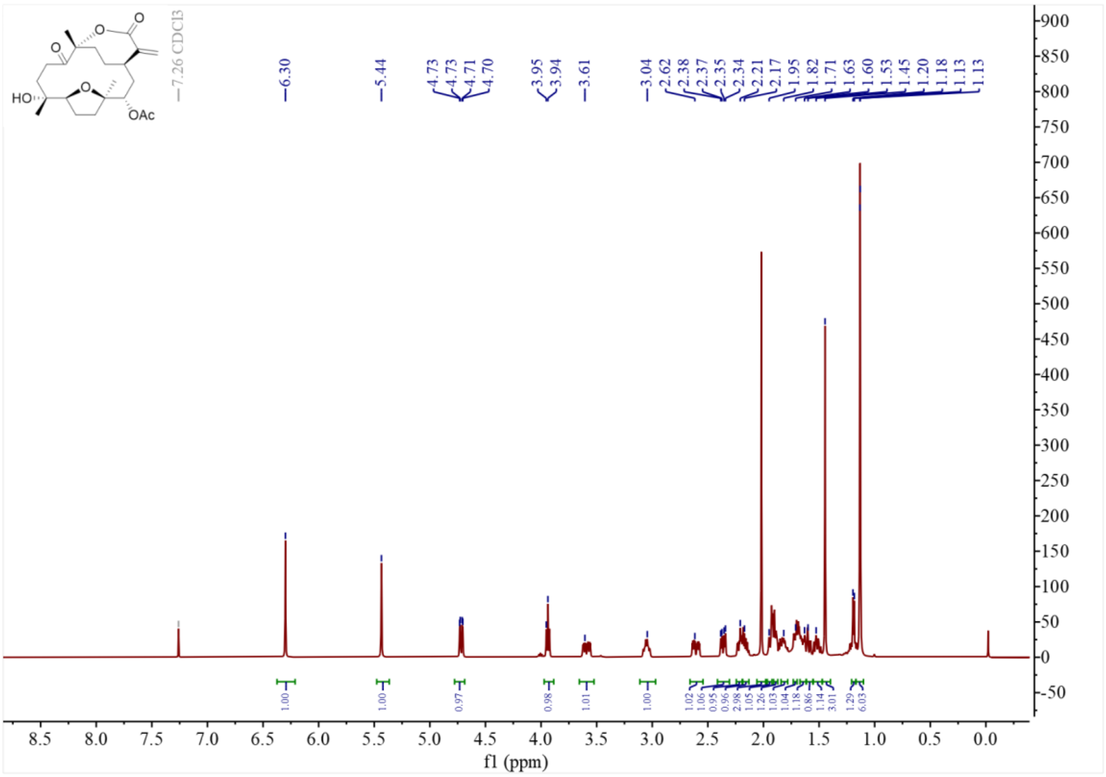
HO
O
O
O
O
OAc
7.26 CDCl3
6.30
5.44
4.73
4.73
4.71
4.70
3.95
3.94
3.61
3.04
2.62
2.38
2.37
2.35
2.34
2.21
2.17
1.95
1.82
1.71
1.63
1.60
1.53
1.45
1.20
1.18
1.13
1.13
f1 (ppm)

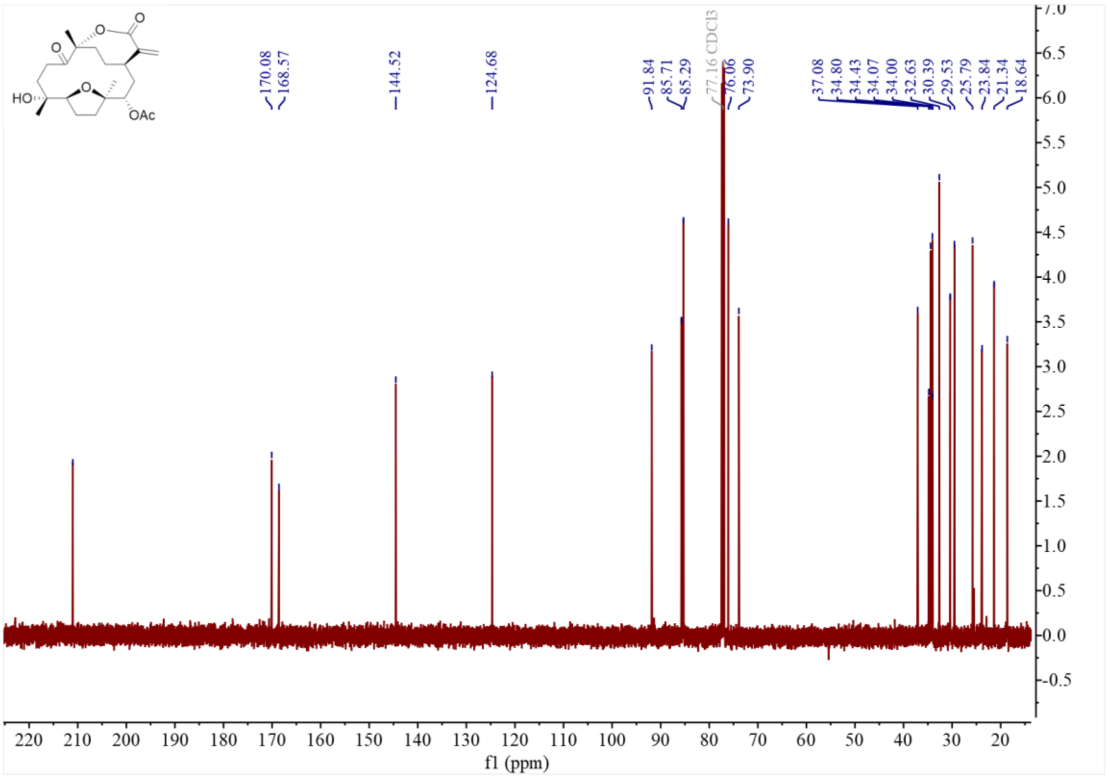
HO
O
O
O
O
OAc
170.08
168.57
144.52
124.68
91.84
85.71
85.29
77.16 CDCl3
76.06
73.90
37.08
34.80
34.43
34.07
34.00
32.63
30.39
29.53
25.79
23.84
21.34
18.64
f1 (ppm)

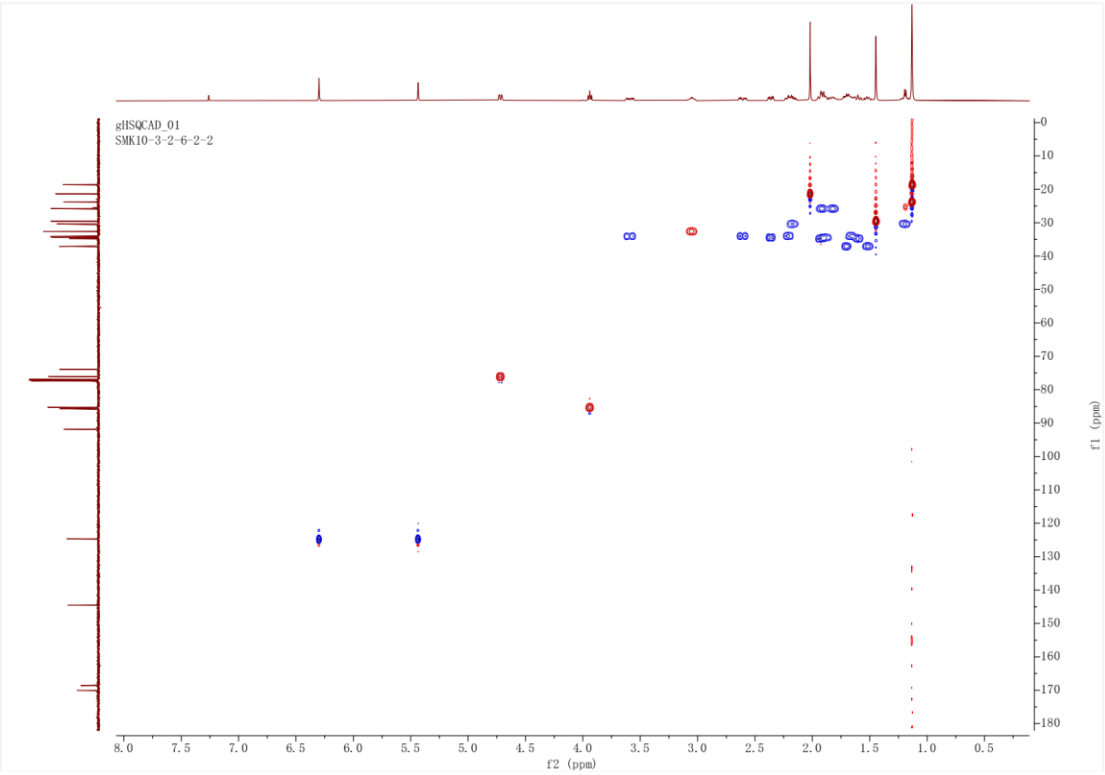
gHSQCAD_01
SMK10-3-2-6-2-2
f2 (ppm)
f1 (ppm)

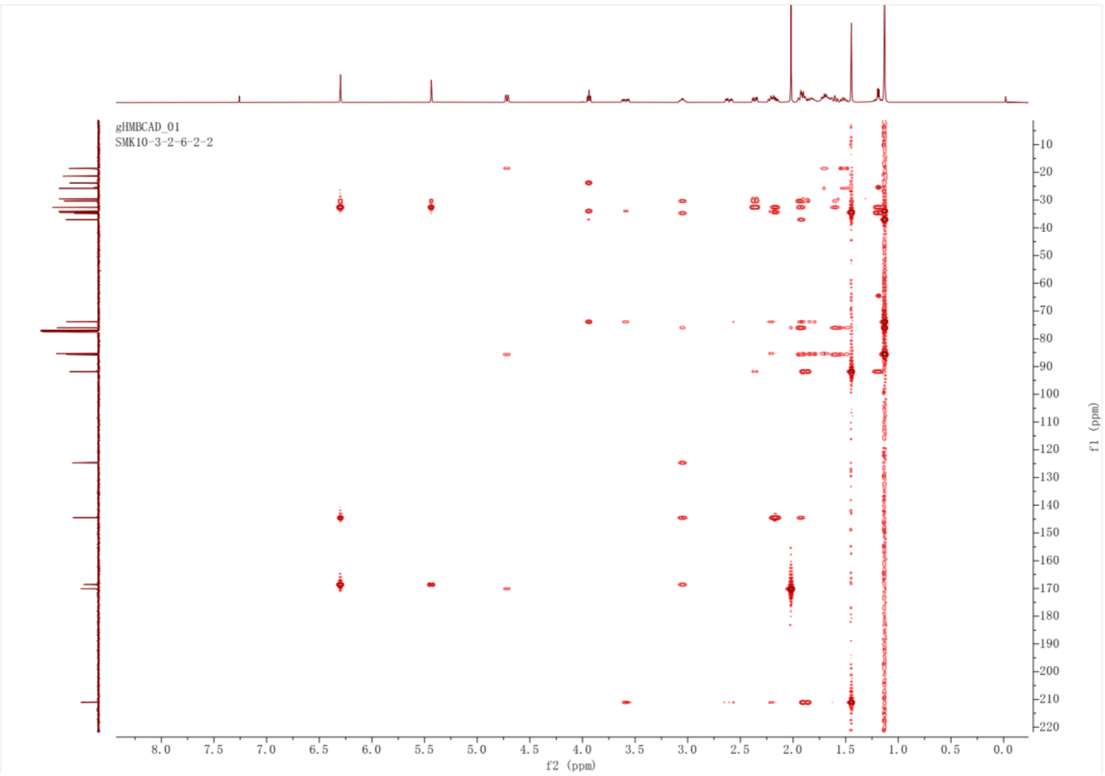
gHMBCAD_01
SMK10-3-2-6-2-2
f2 (ppm)
f1 (ppm)

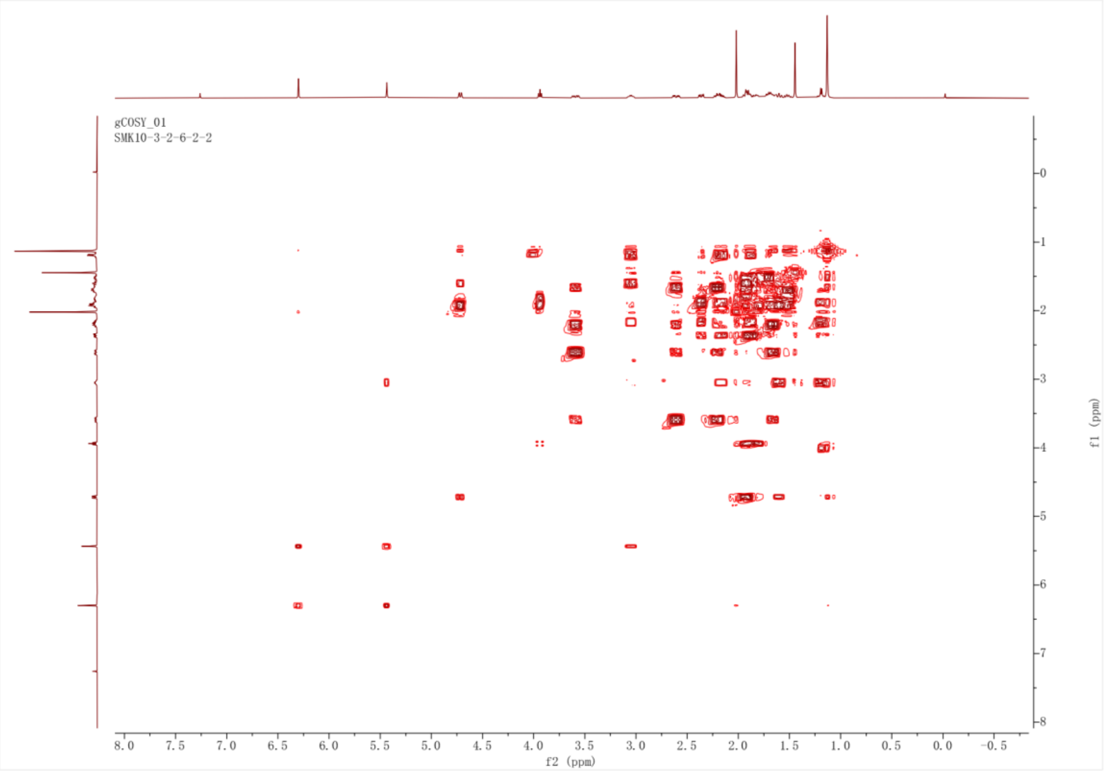
gCOSY_01
SMK10-3-2-6-2-2
f2 (ppm)
f1 (ppm)

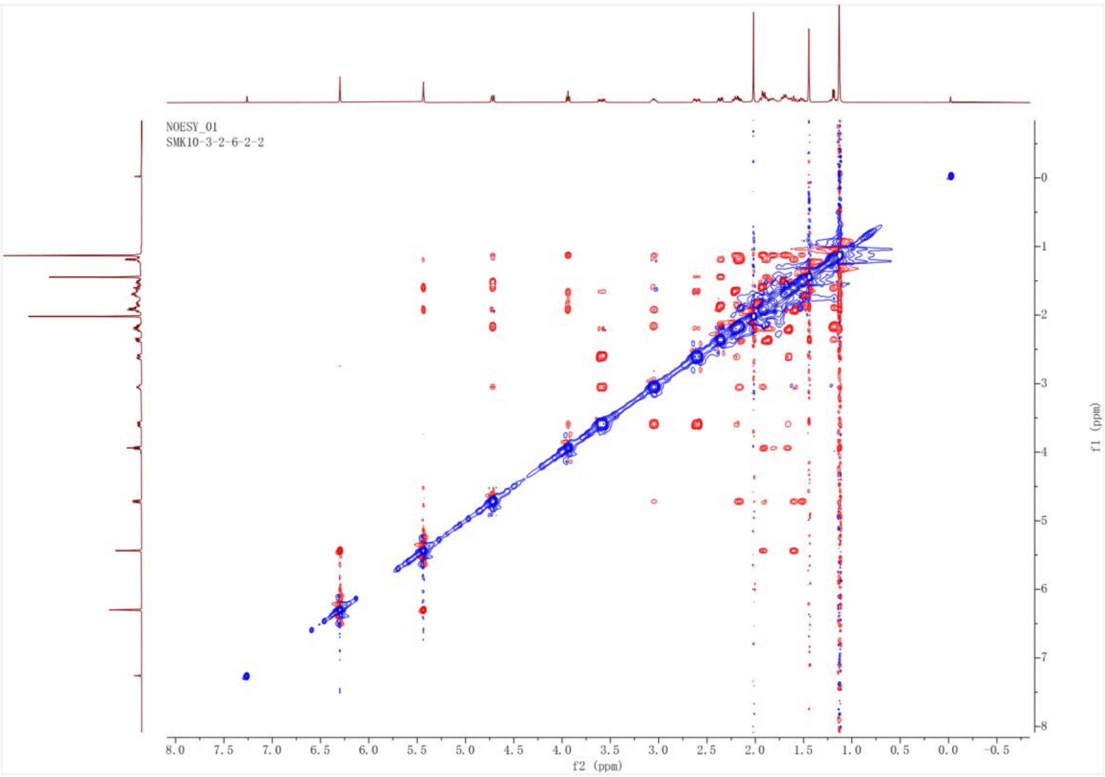
NOESY_01
SMK10-3-2-6-2-2
f2 (ppm)
f1 (ppm)

Figure.S13 Spectra (HRESIMS, UV, ^{1}H NMR, ^{13}C NMR, HSQC, HMBC, ^{1}H–^{1}H COSY, NOESY) for compound 8

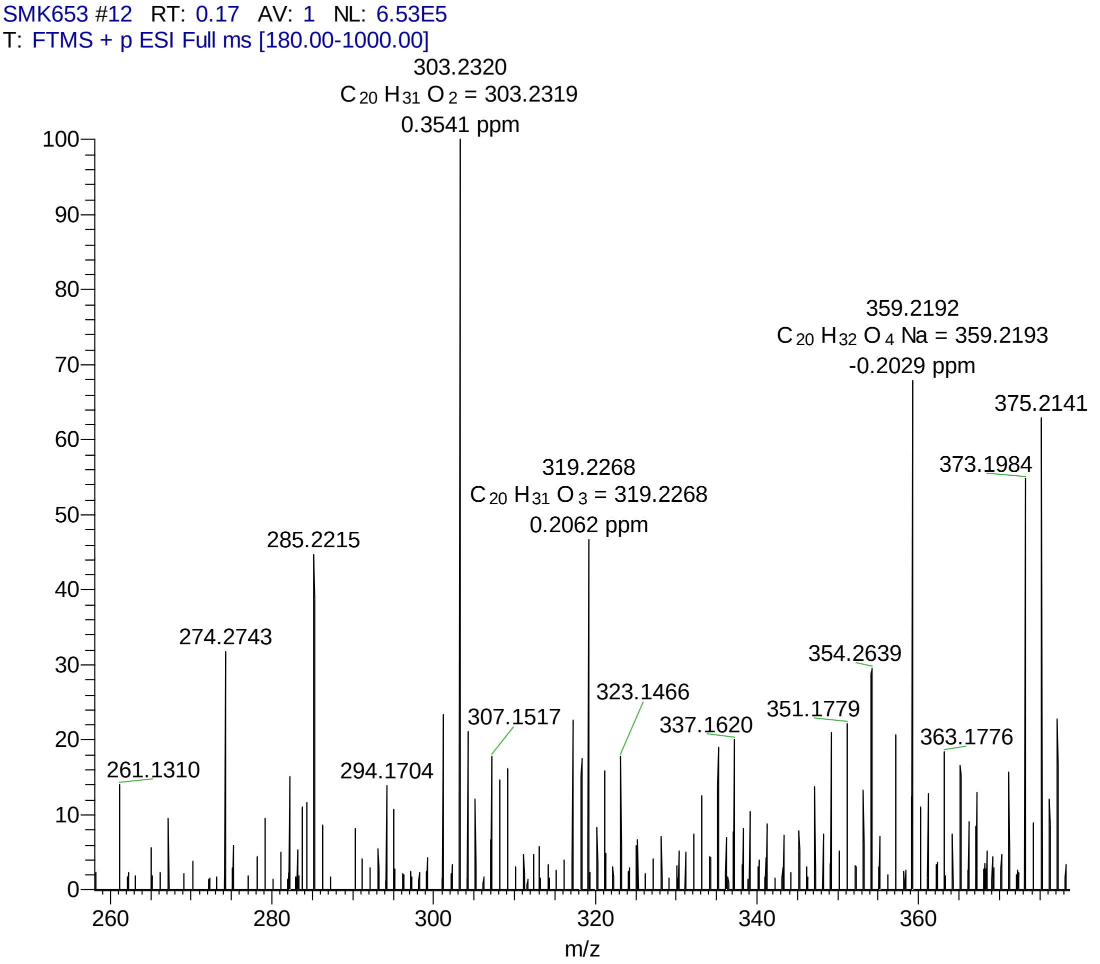

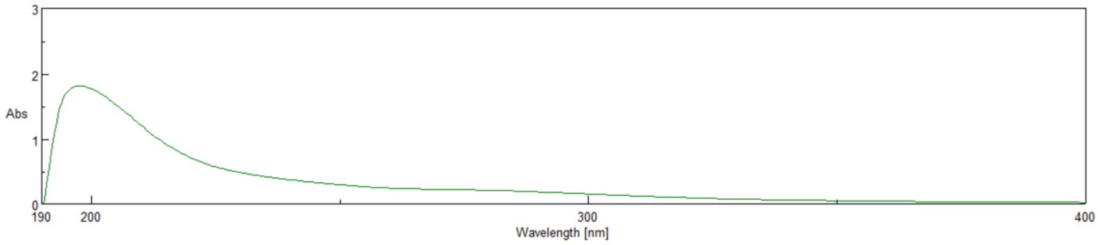

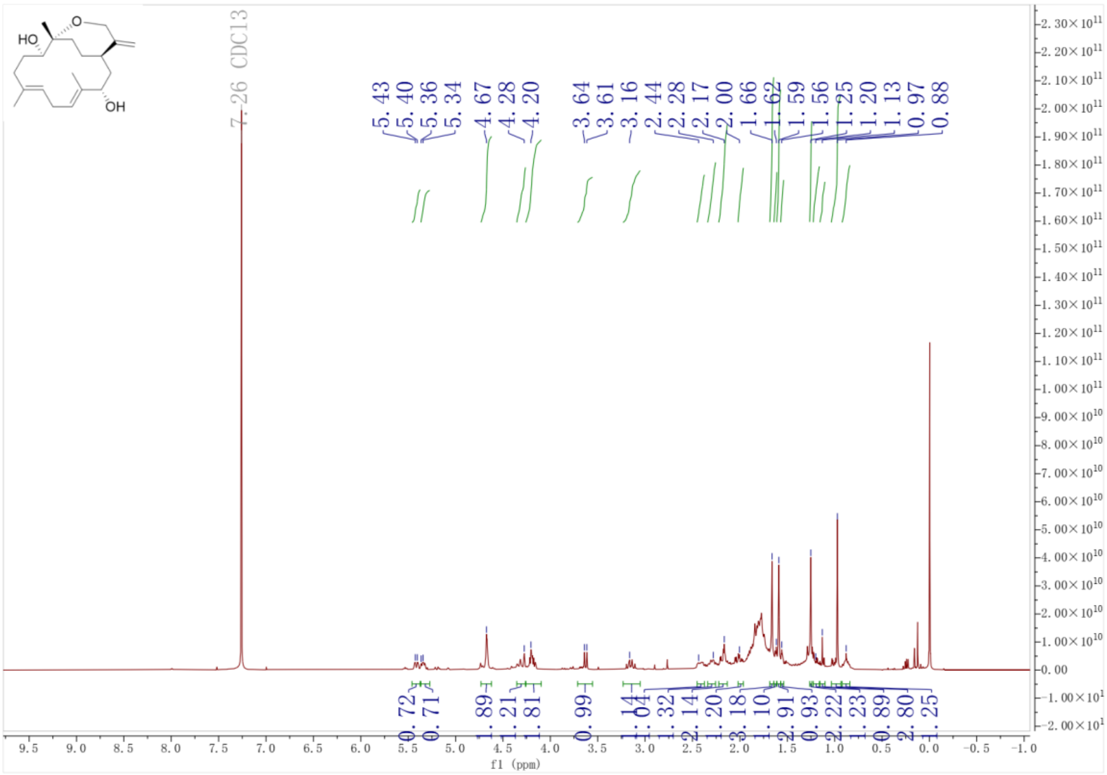
HO
O
OH
7.26 CDCl3
f1 (ppm)

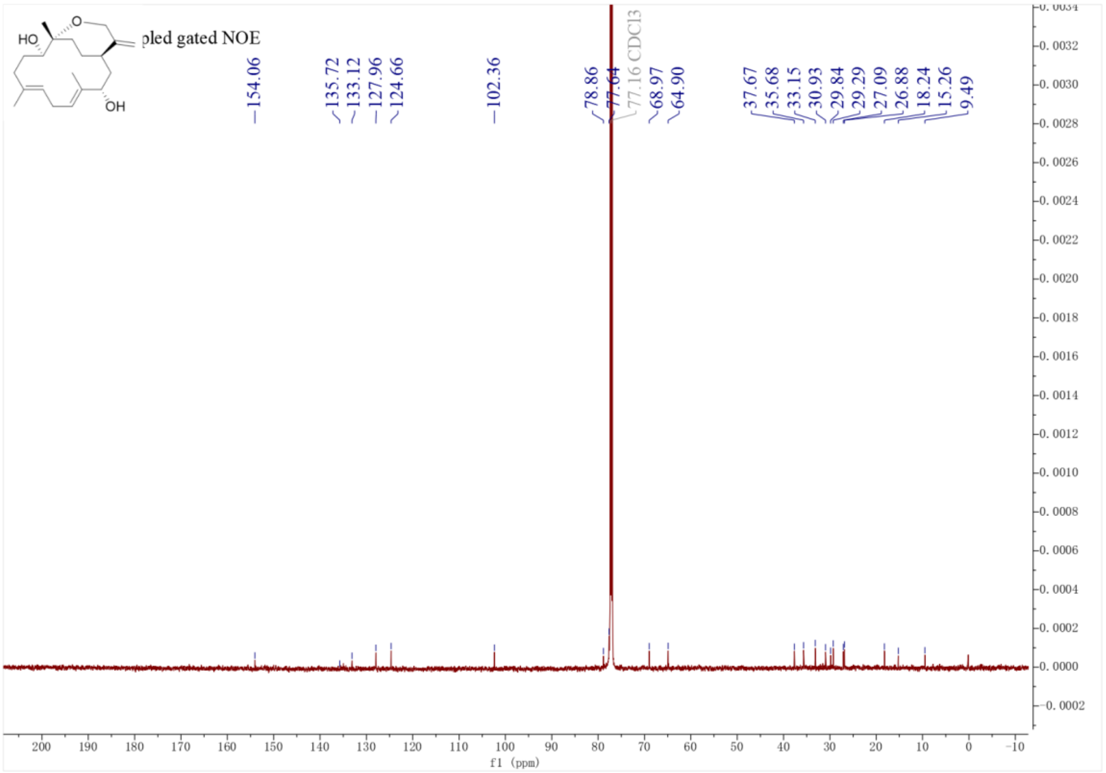
HO
O
OH
pled gated NOE
77.16 CDCl3
f1 (ppm)

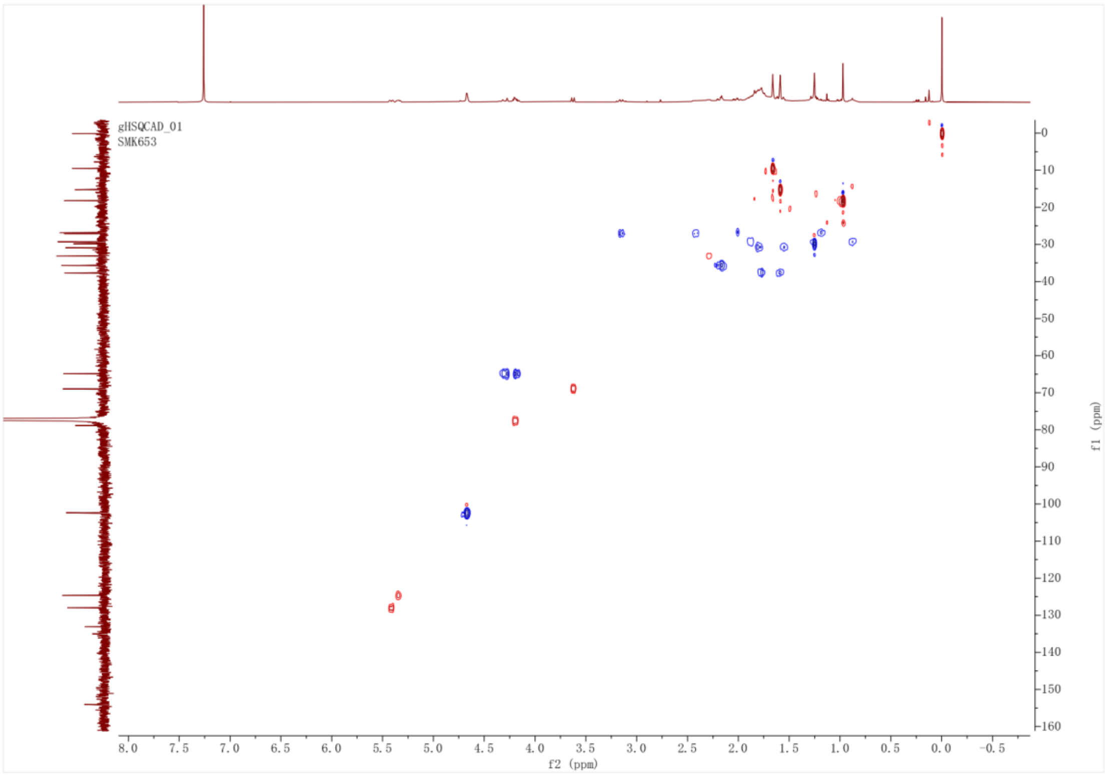
gHSQCAD_01
SMK653
f2 (ppm)
f1 (ppm)

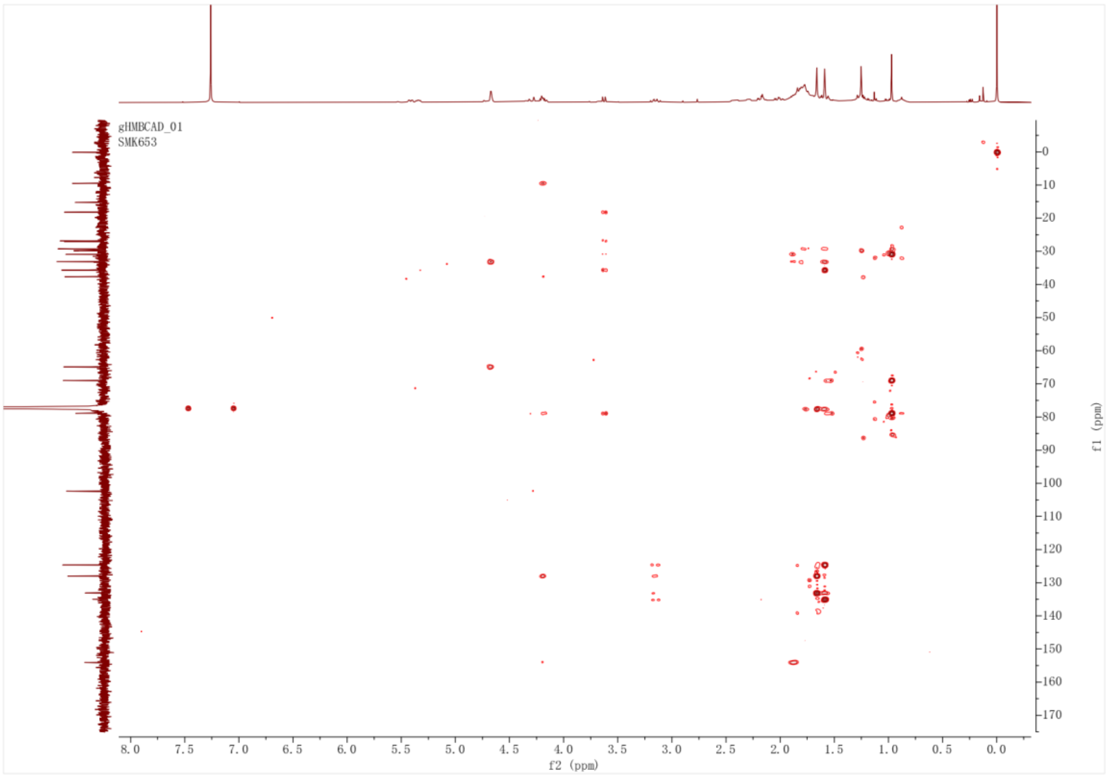
gHMBCAD_01
SMK653
f2 (ppm)
f1 (ppm)

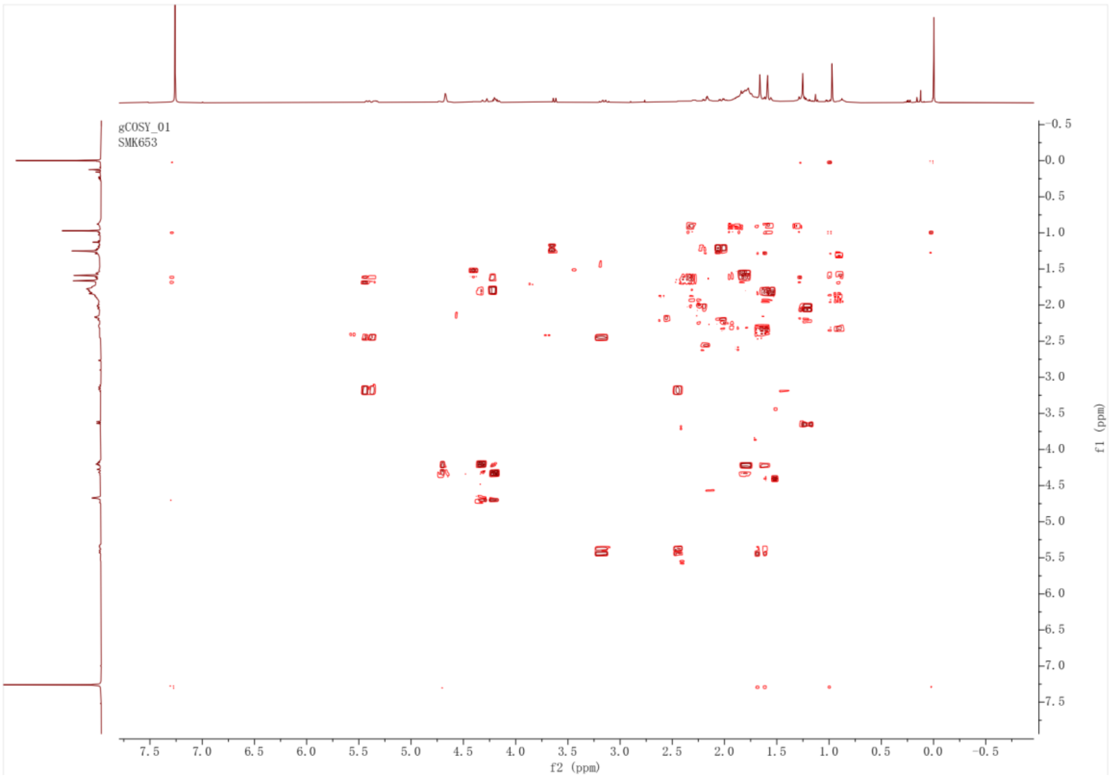
gCOSY_01
SMK653
f2 (ppm)
f1 (ppm)

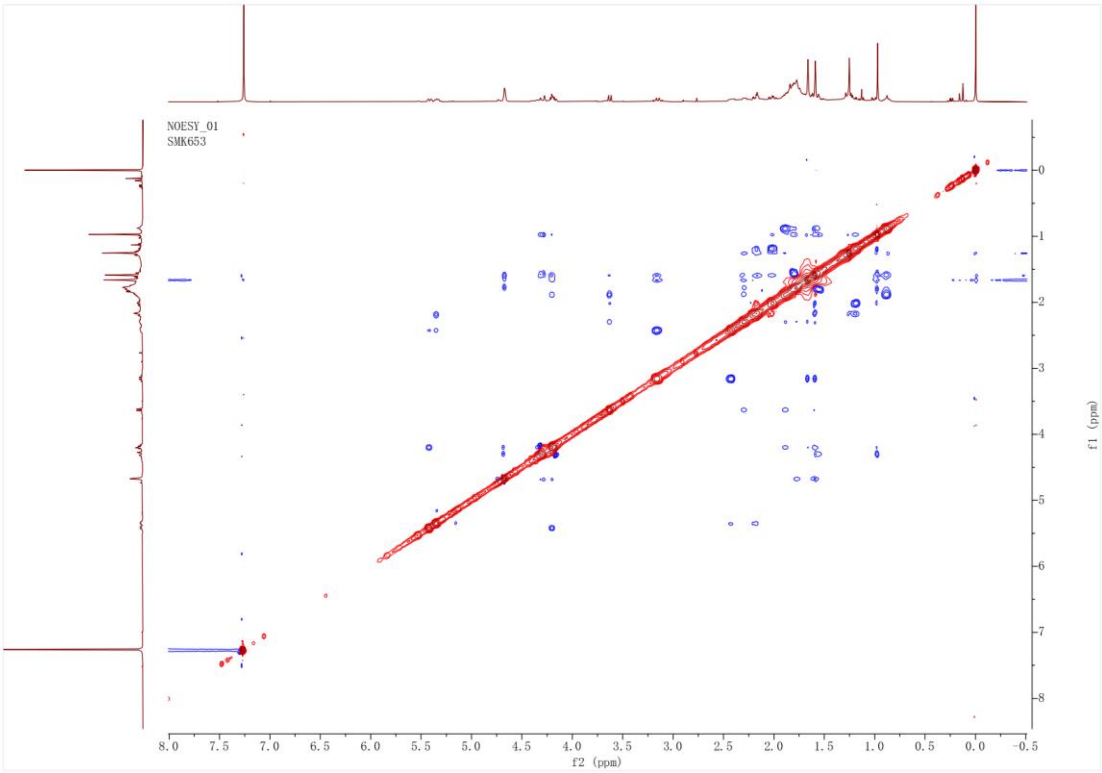
NOESY_01
SMK653
f2 (ppm)
f1 (ppm)

Figure.S14 Spectra (HRESIMS, UV, ^{1}H NMR, ^{13}C NMR, HSQC, HMBC, ^{1}H–^{1}H COSY, NOESY) for compound 9

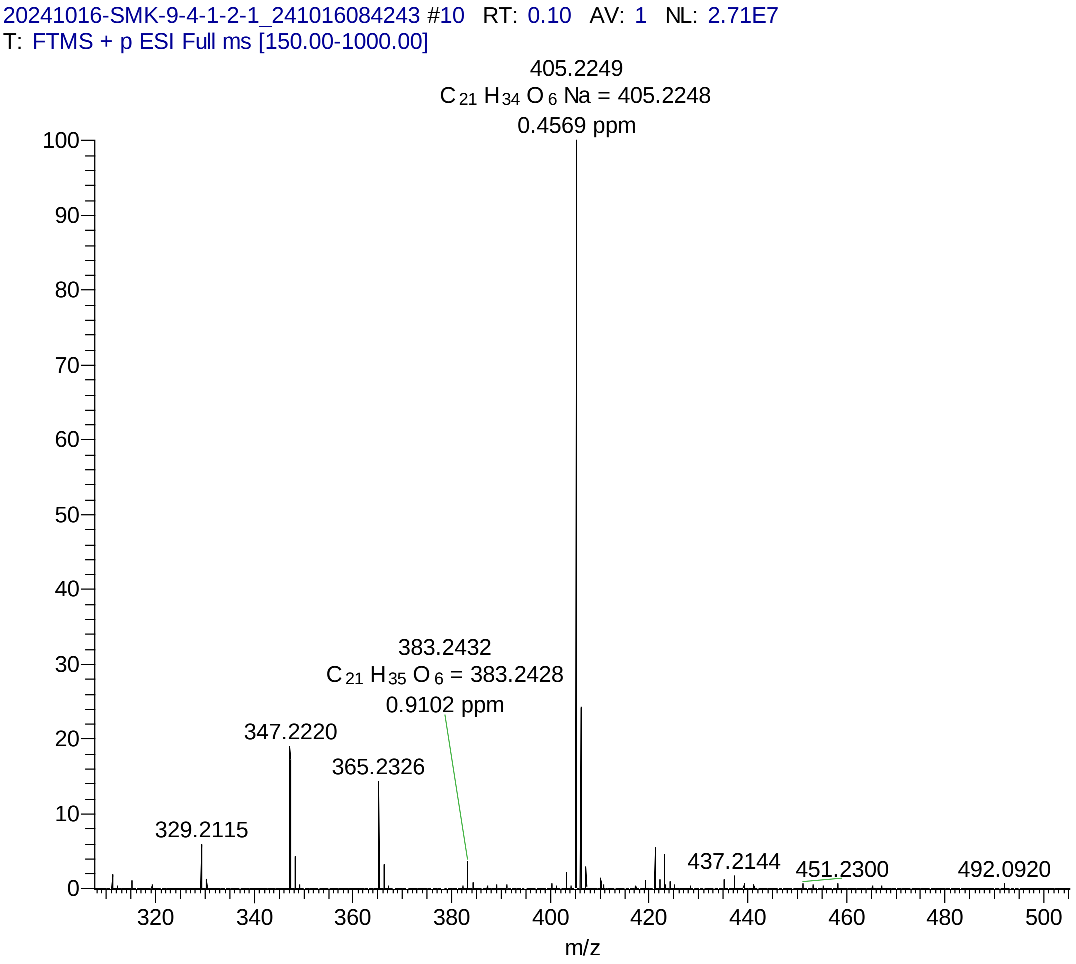

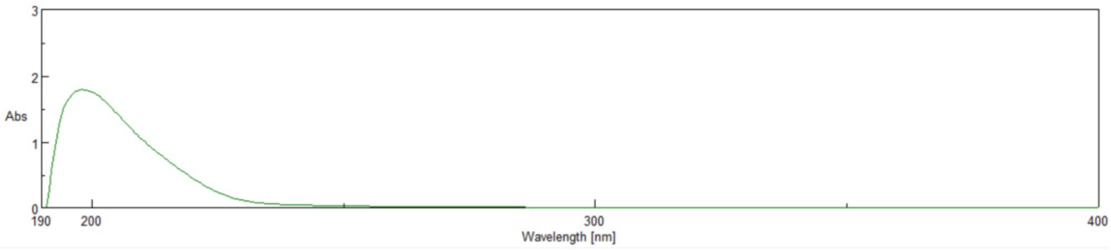

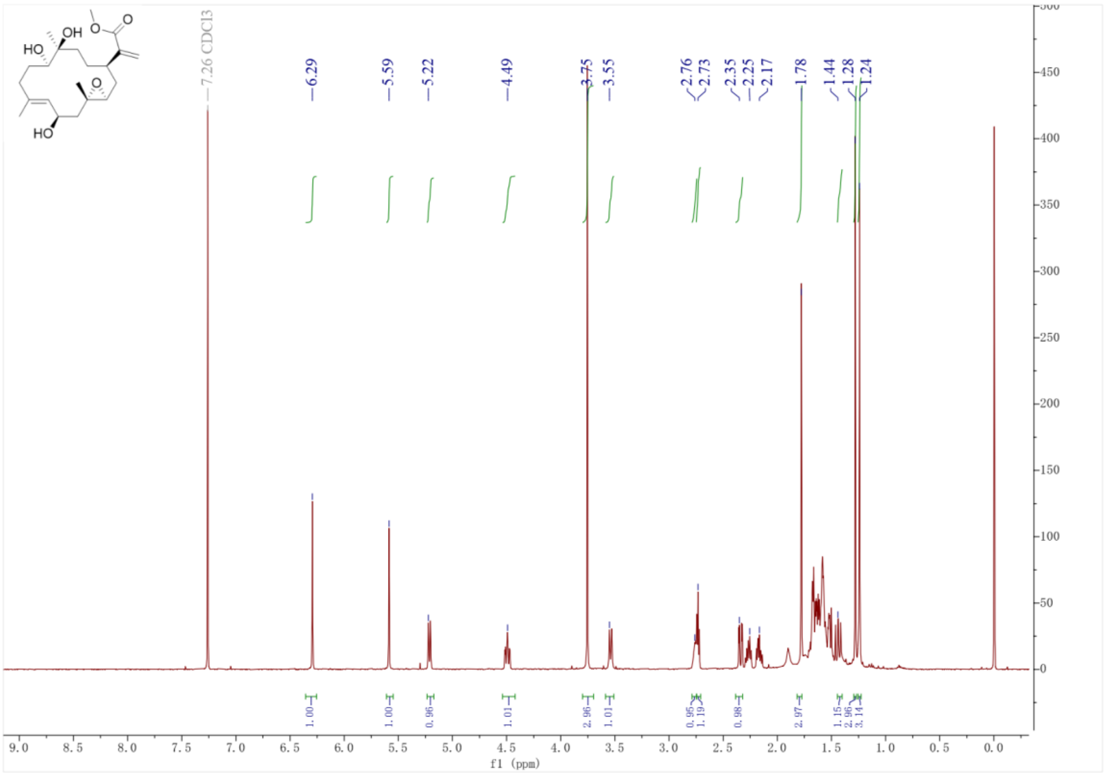
HO
OH
O
O
O
HO
7.26 CDCl3
6.29
5.59
5.22
4.49
3.75
3.55
2.76
2.73
2.35
2.25
2.17
1.78
1.44
1.28
1.24
1.00
1.00
0.96
1.01
2.96
1.01
0.95
1.19
0.98
2.97
1.15
2.96
3.14
f1 (ppm)

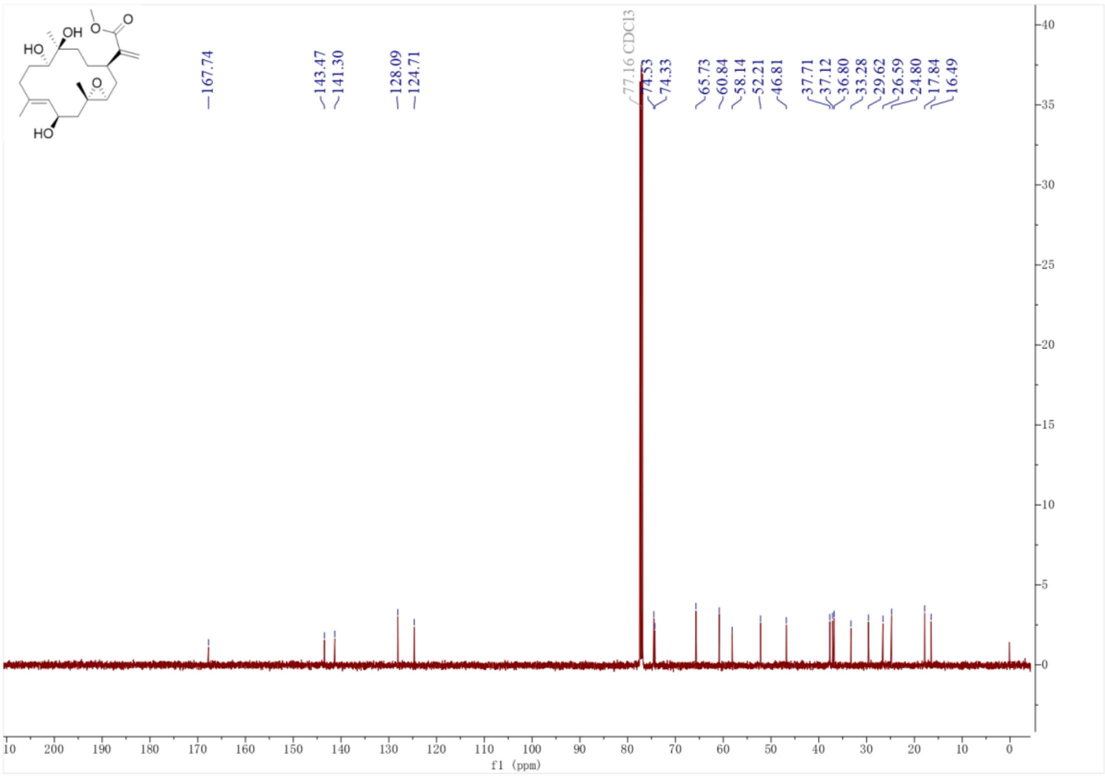
HO
OH
O
O
O
HO
167.74
143.47
141.30
128.09
124.71
77.16 CDCl3
74.53
74.33
65.73
60.84
58.14
52.21
46.81
37.71
37.12
36.80
33.28
29.62
26.59
24.80
17.84
16.49
f1 (ppm)

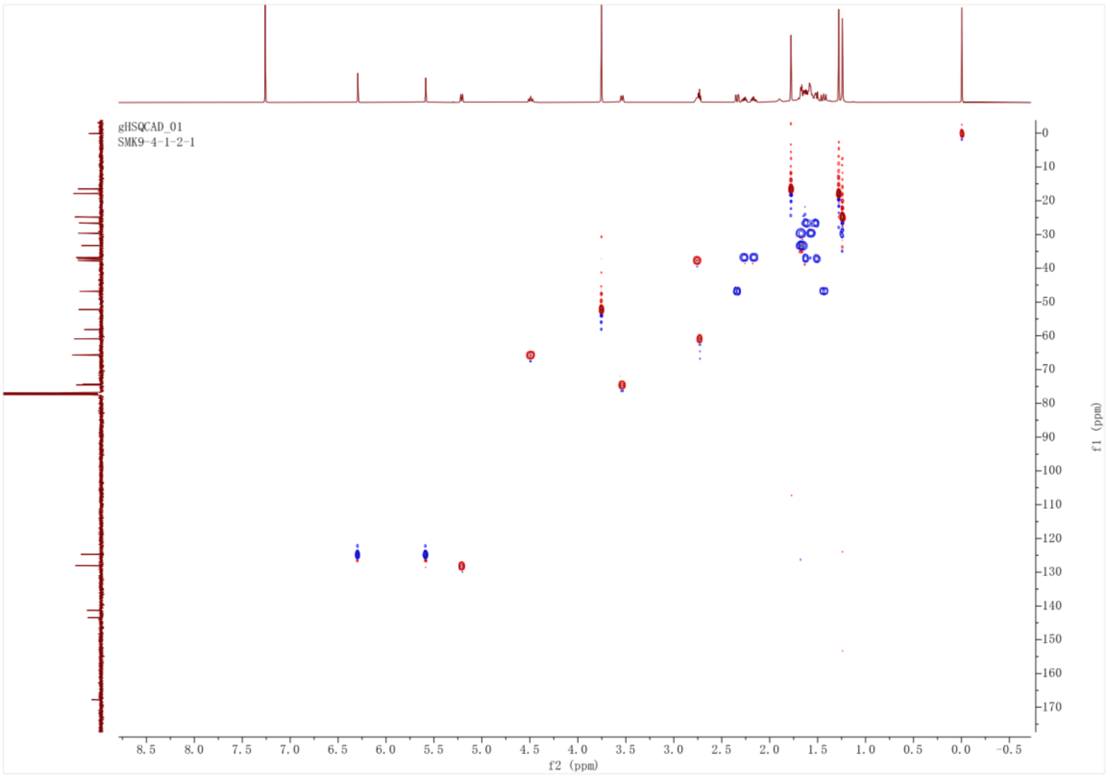
gHSQCAD_01
SMK9-4-1-2-1
f2 (ppm)
f1 (ppm)

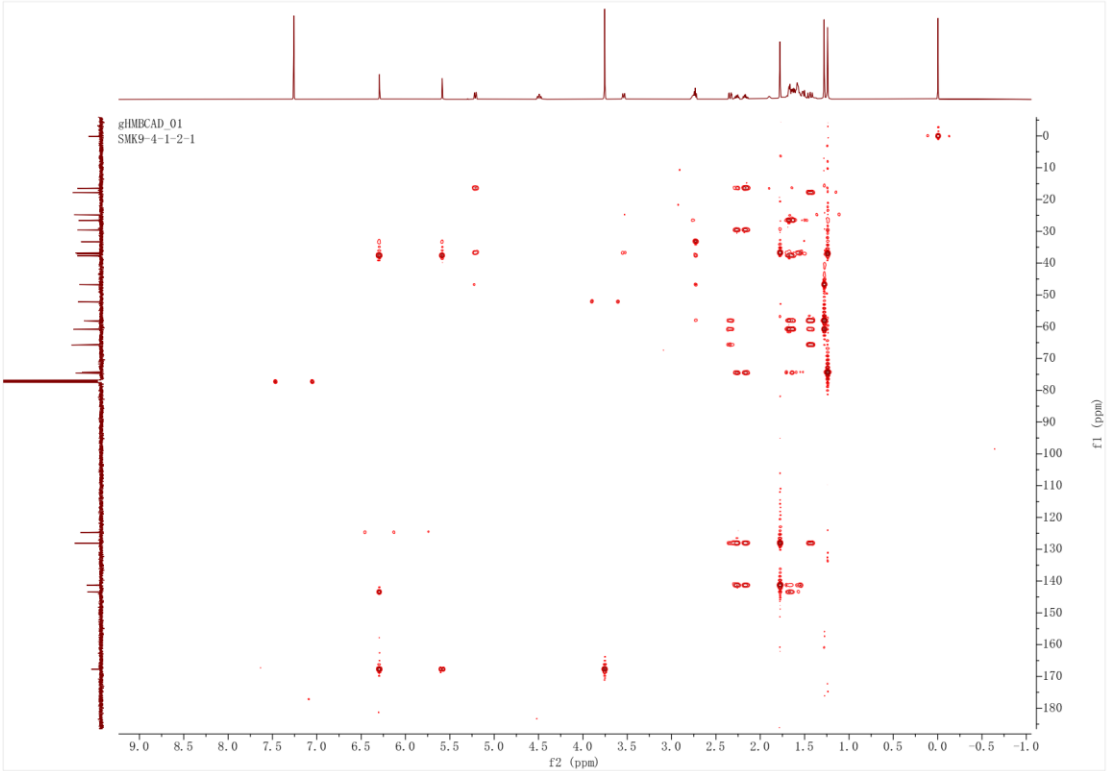
gHMBCAD_01
SMK9-4-1-2-1
f2 (ppm)
f1 (ppm)

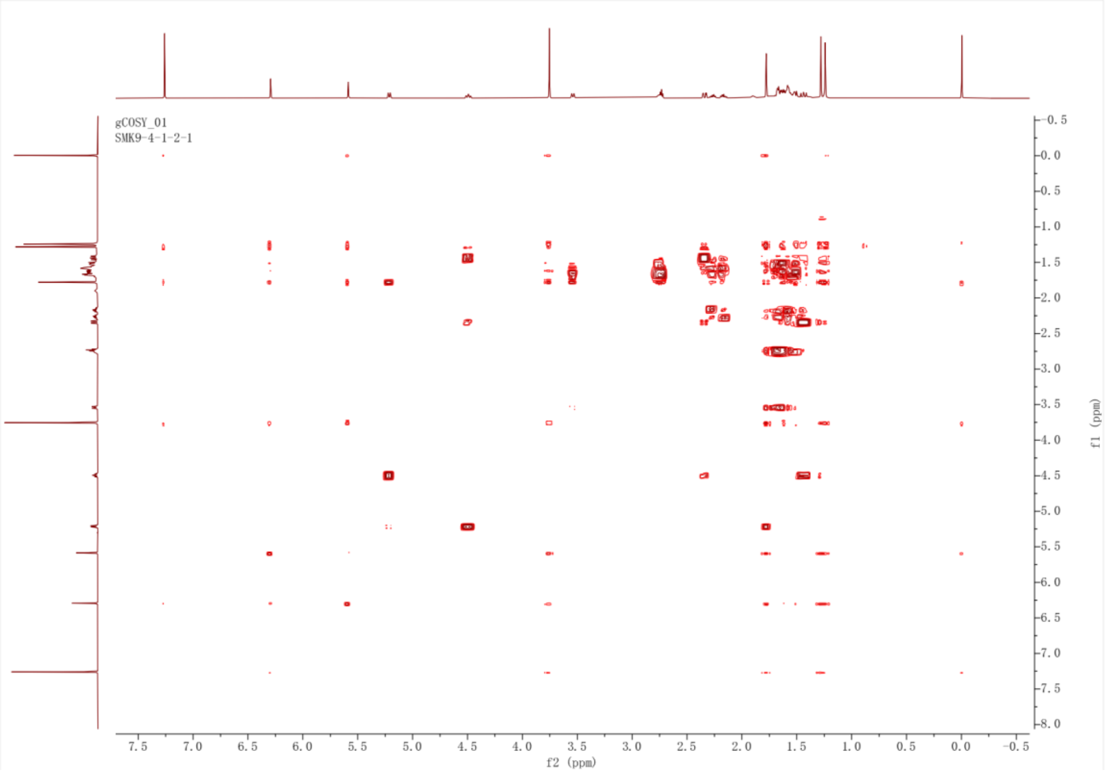
gCOSY_01
SMK9-4-1-2-1
f2 (ppm)
f1 (ppm)

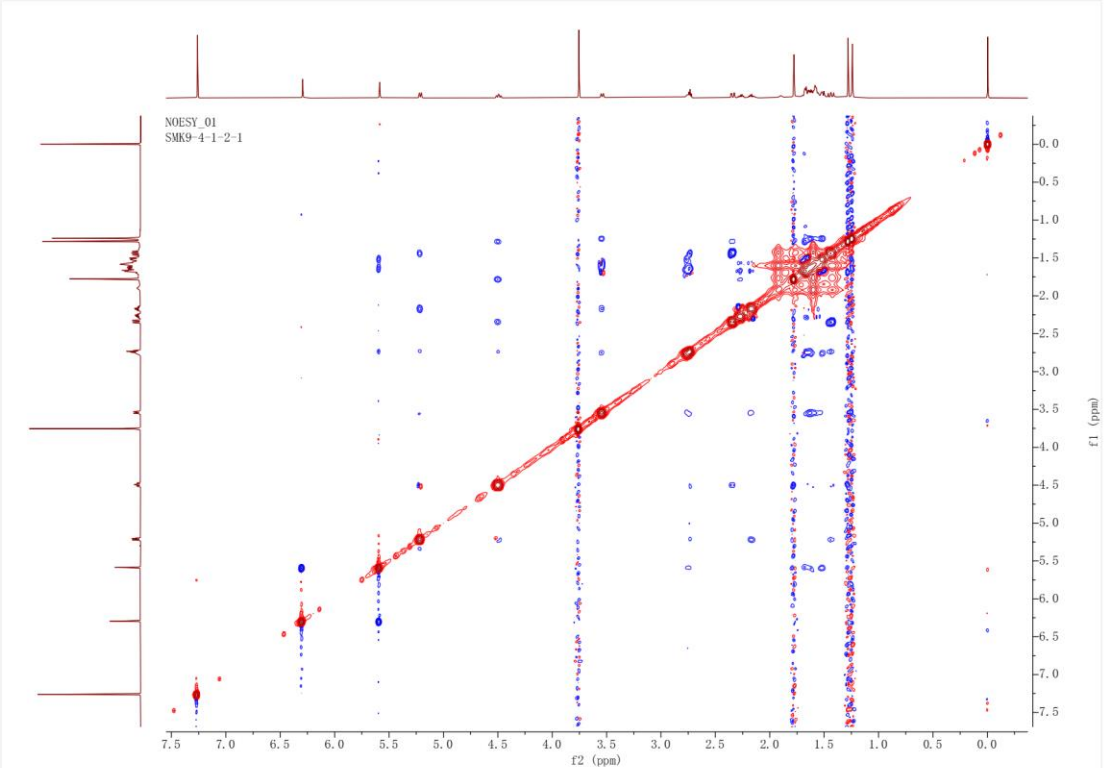
NOESY_01
SMK9-4-1-2-1
f2 (ppm)
f1 (ppm)

Figure.S15 Spectra (HRESIMS, UV, ^{1}H NMR, ^{13}C NMR, HSQC, HMBC, ^{1}H–^{1}H COSY, NOESY) for compound 10

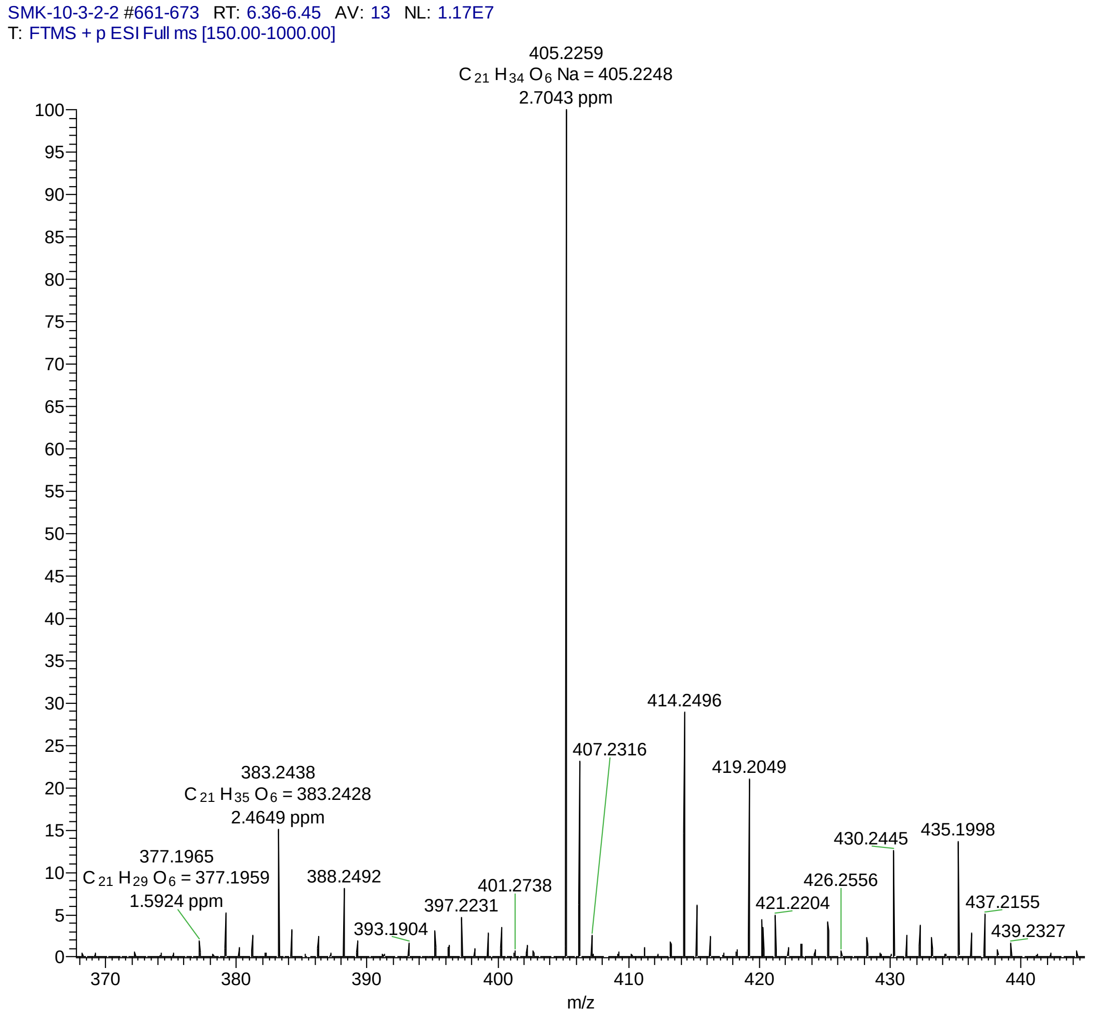

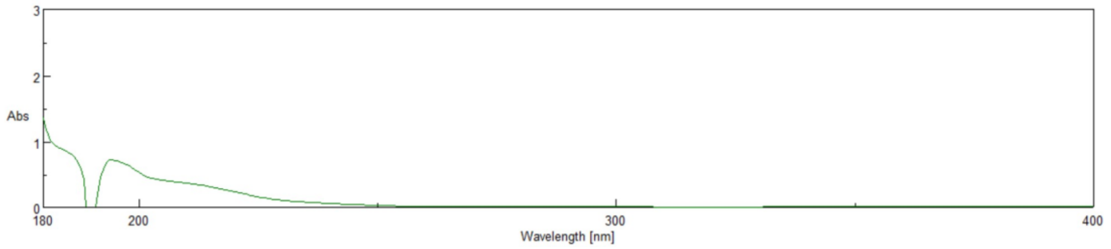

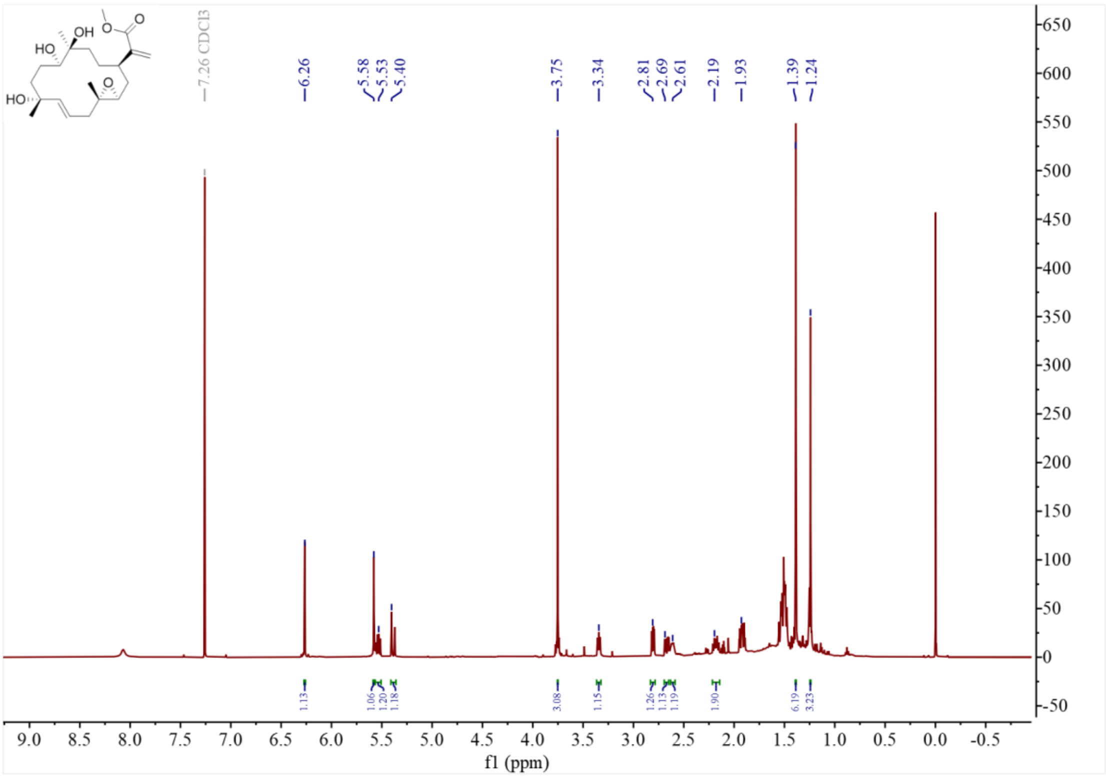
HO
OH
O
O
HO
O
7.26 CDCl3
6.26
5.58
5.53
5.40
3.75
3.34
2.81
2.69
2.61
2.19
1.93
1.39
1.24
1.13
1.06
1.20
1.18
3.08
1.15
1.26
1.13
1.19
1.90
6.19
3.23
9.0 8.5 8.0 7.5 7.0 6.5 6.0 5.5 5.0 4.5 4.0 3.5 3.0 2.5 2.0 1.5 1.0 0.5 0.0 -0.5
f1 (ppm)
650 600 550 500 450 400 350 300 250 200 150 100 50 0 -50

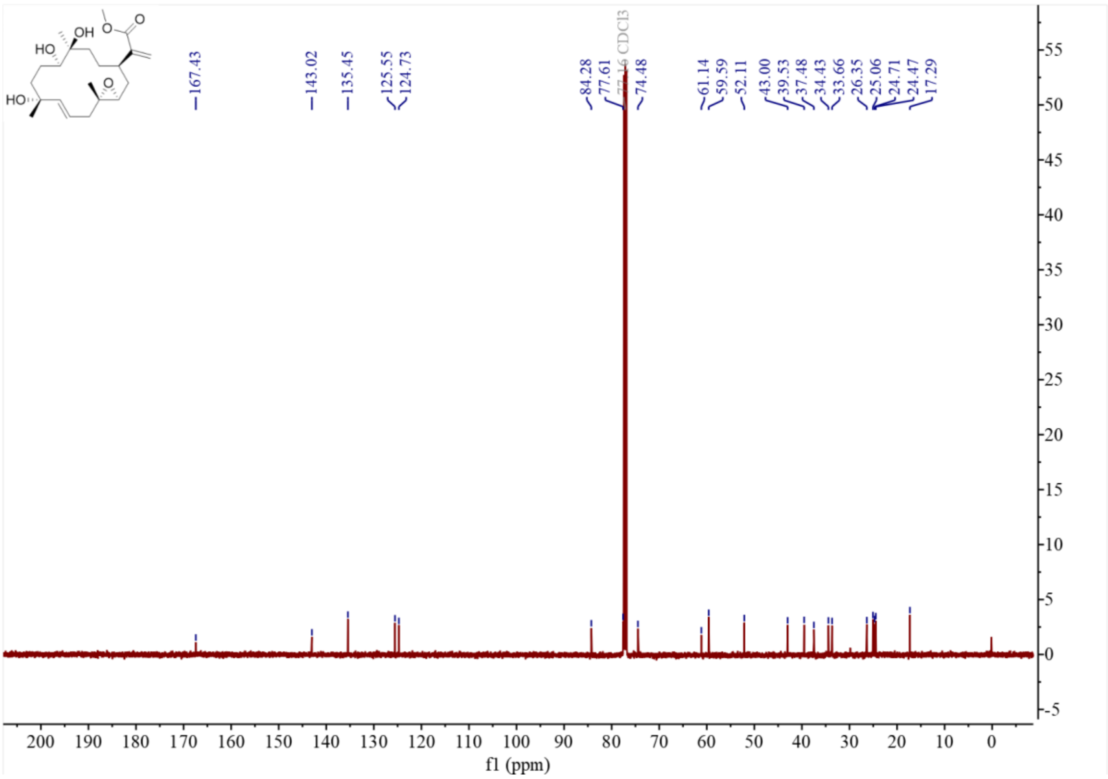
HO
OH
O
O
HO
O
167.43
143.02
135.45
125.55
124.73
84.28
77.61
77.16 CDCl3
74.48
61.14
59.59
52.11
43.00
39.53
37.48
34.43
33.66
26.35
25.06
24.71
24.47
17.29
200 190 180 170 160 150 140 130 120 110 100 90 80 70 60 50 40 30 20 10 0
f1 (ppm)
55 50 45 40 35 30 25 20 15 10 5 0 -5

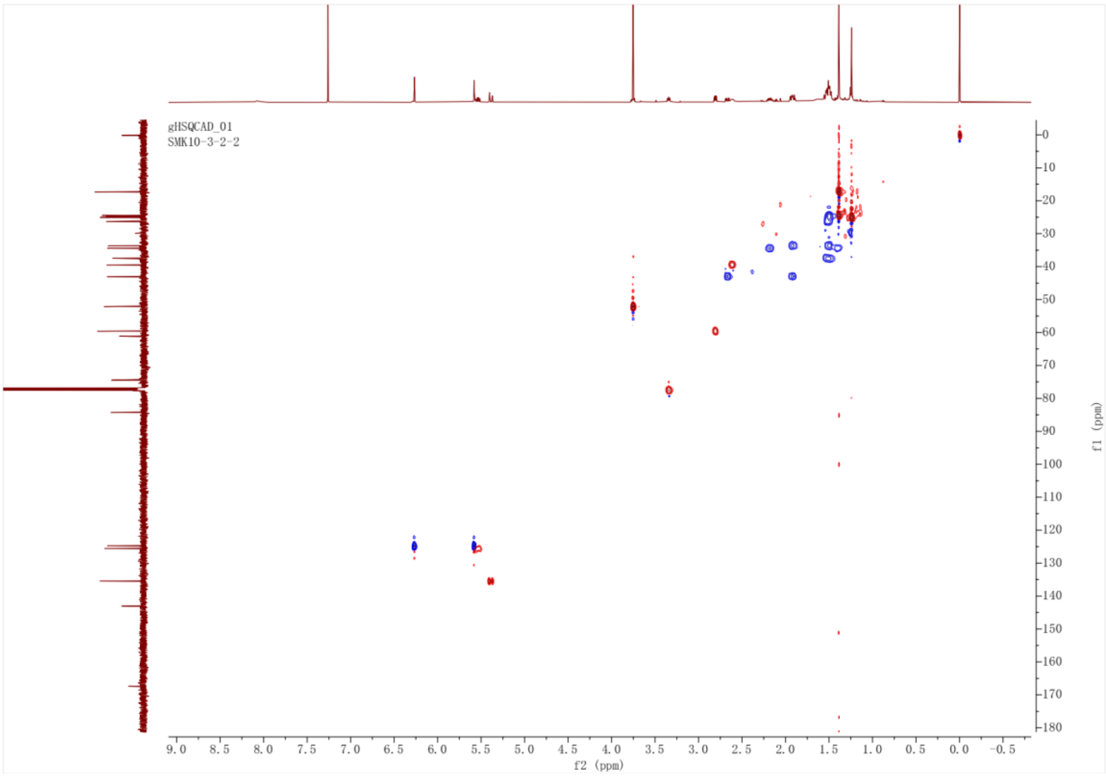
gHSQCAD_01
SMK10-3-2-2
f2 (ppm)
f1 (ppm)

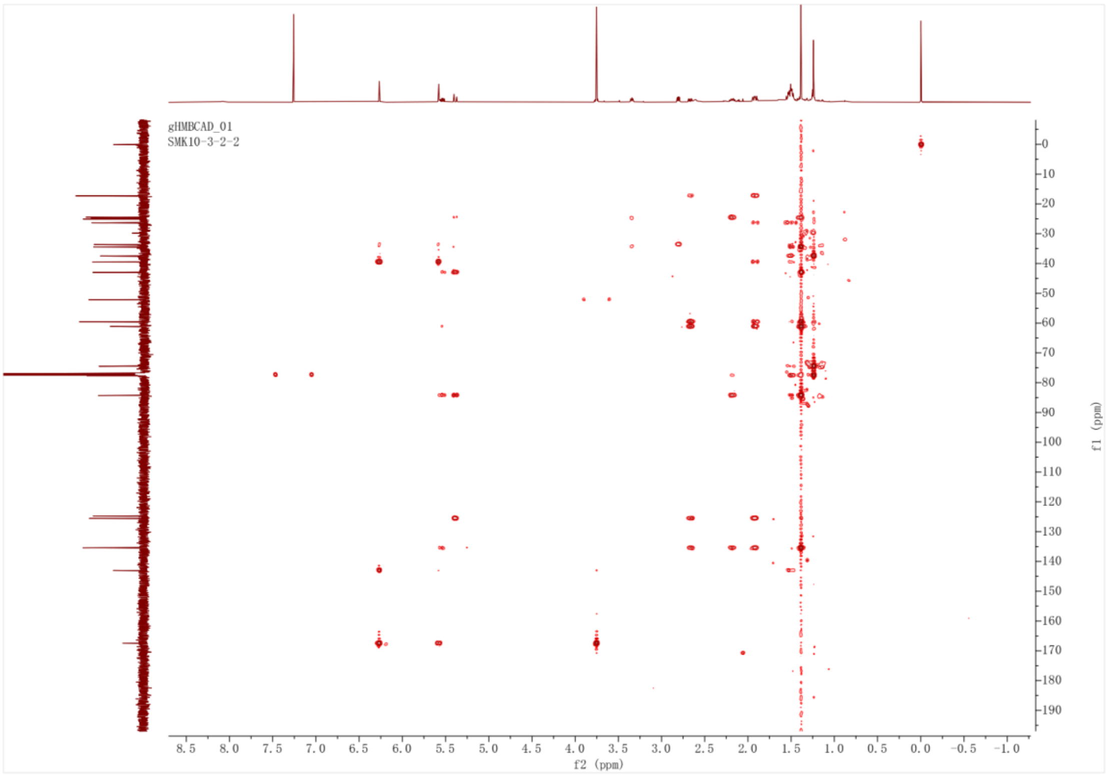
gHMBCAD_01
SMK10-3-2-2
f2 (ppm)
f1 (ppm)

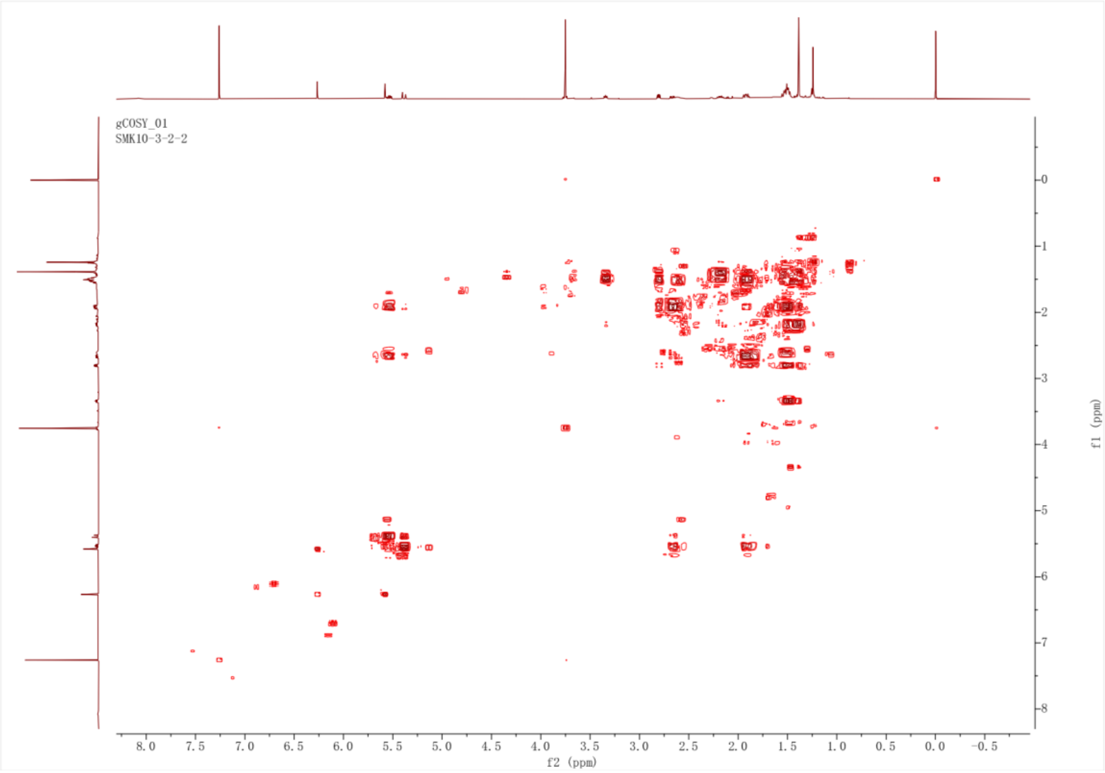
gCOSY_01
SMK10-3-2-2
f2 (ppm)
f1 (ppm)

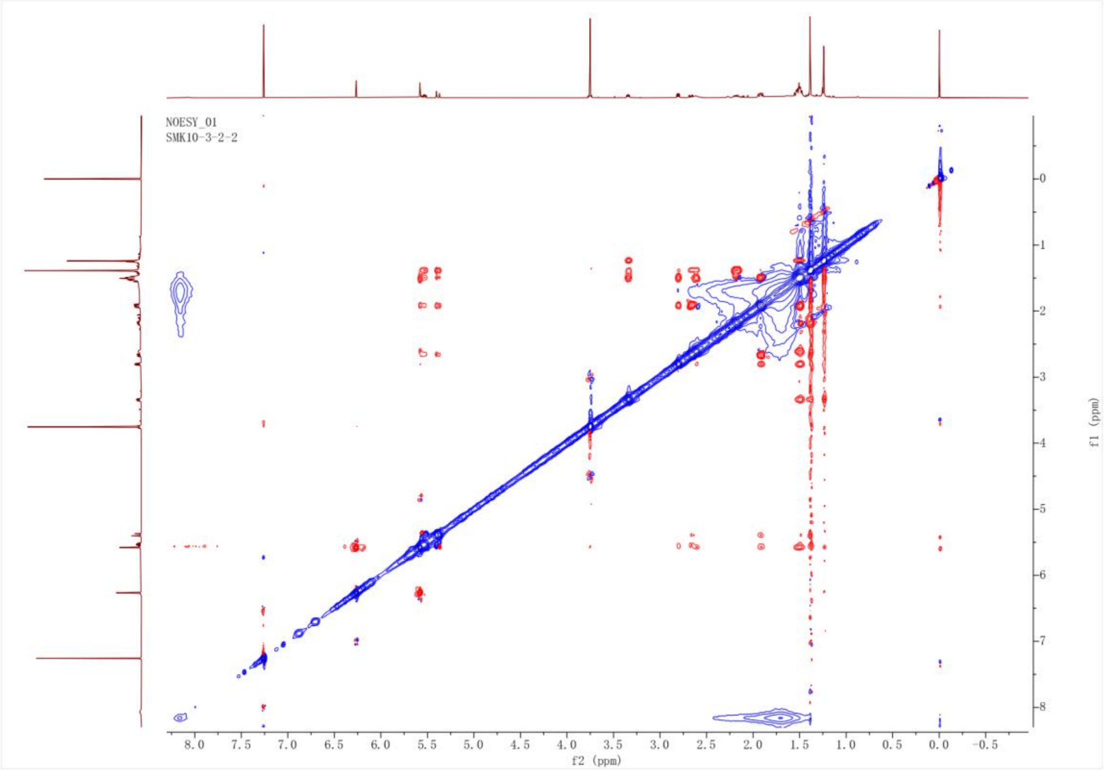
NOESY_01
SMK10-3-2-2
f2 (ppm)
f1 (ppm)

Figure.S16 Spectra (HRESIMS, UV, ^{1}H NMR, ^{13}C NMR, HSQC, HMBC, ^{1}H–^{1}H COSY, NOESY) for compound 11

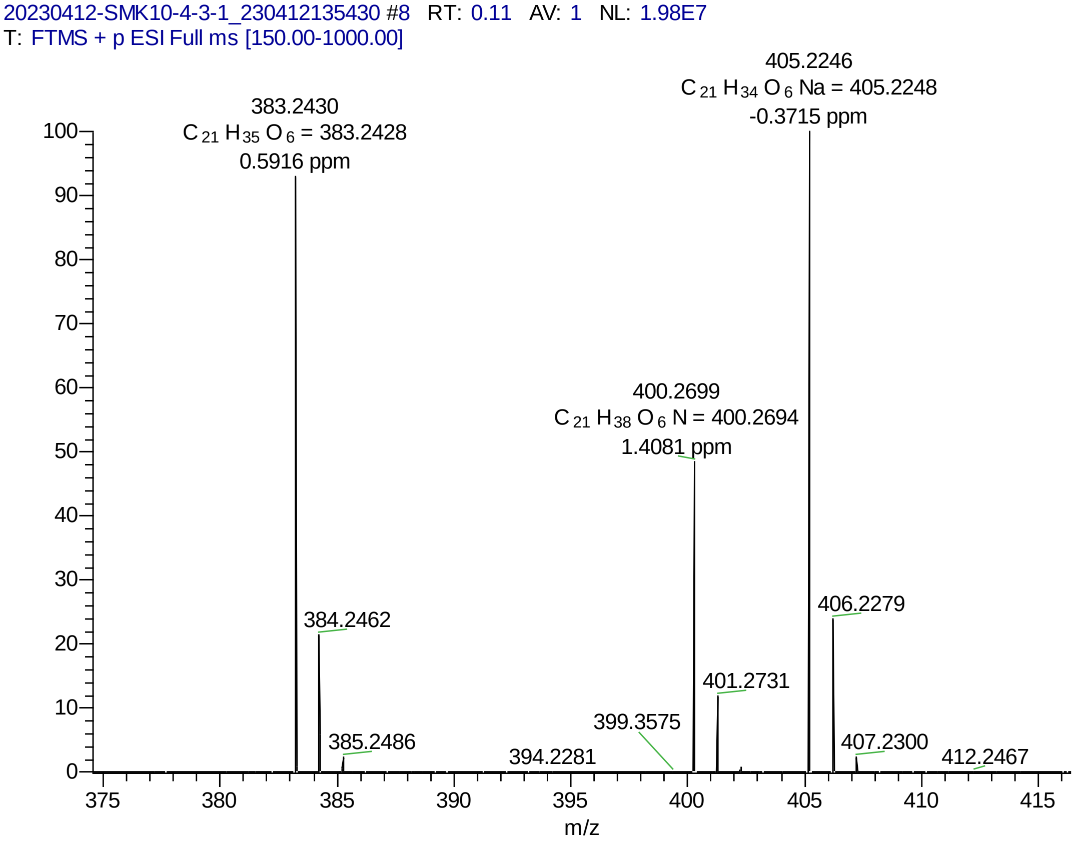

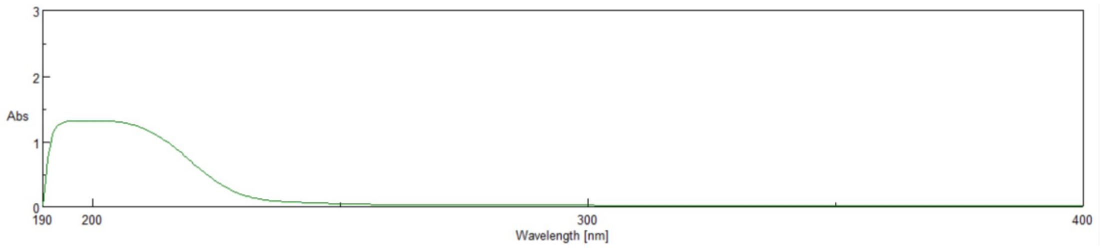

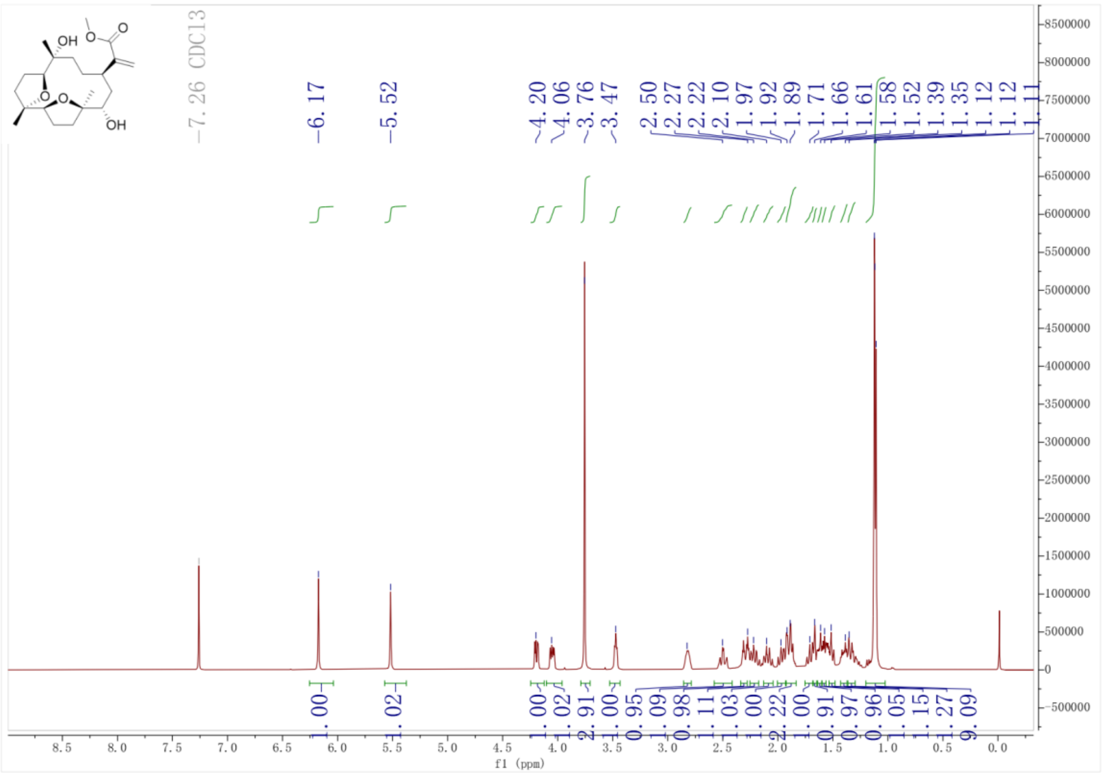

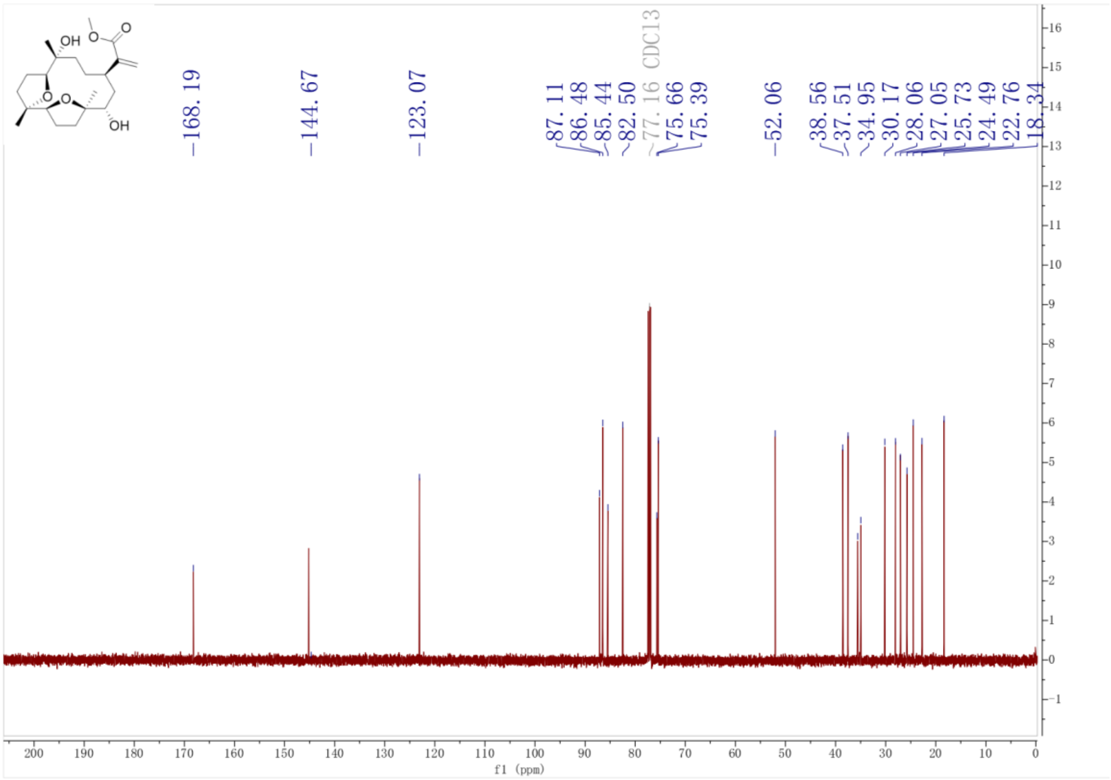

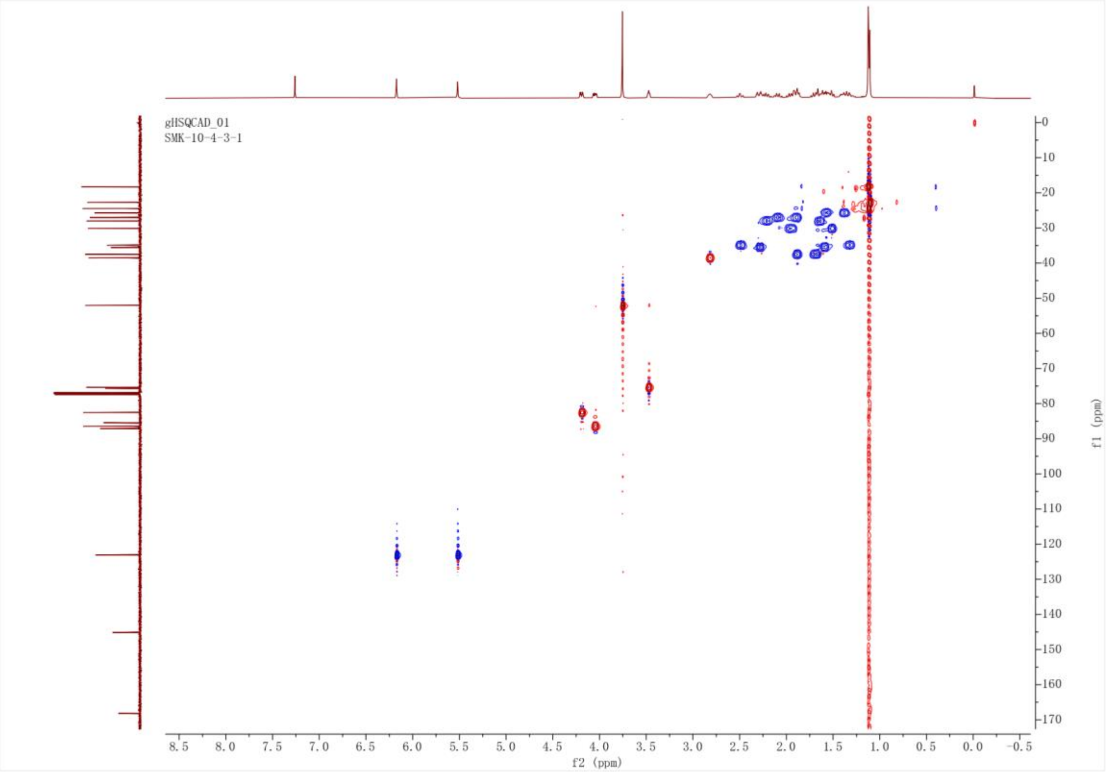
gHSQCAD_01
SMK-10-4-3-1
f2 (ppm)
f1 (ppm)

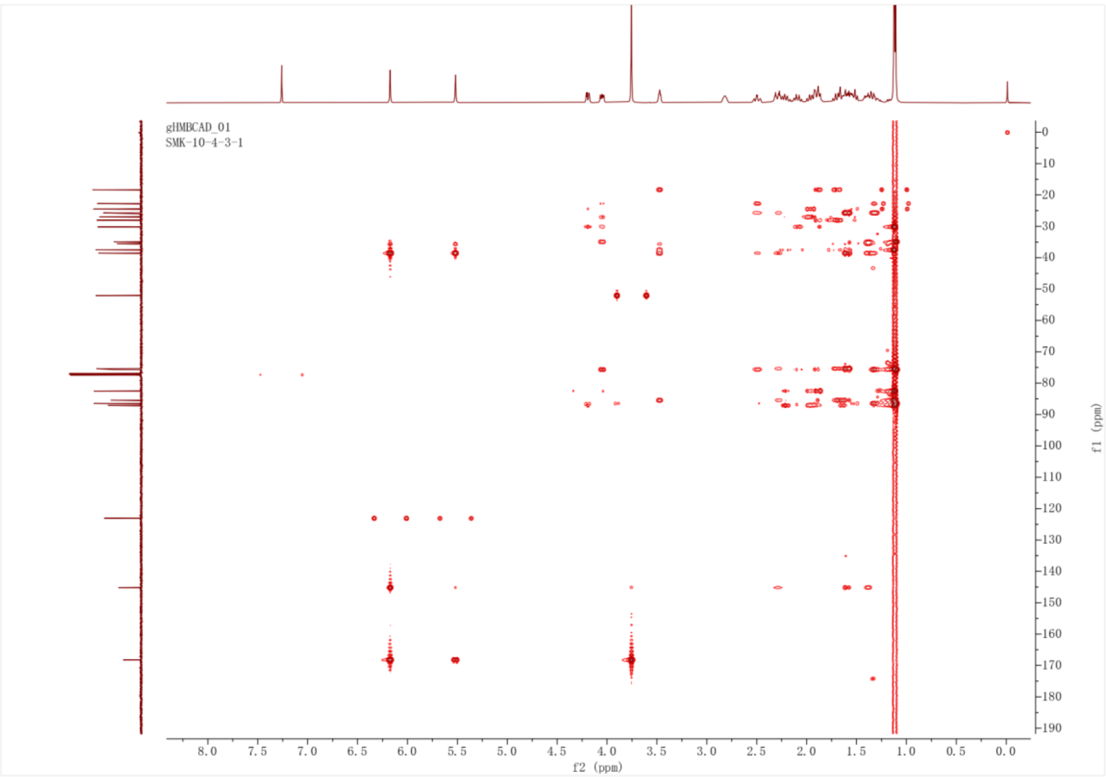
gHMBCAD_01
SMK-10-4-3-1
f2 (ppm)
f1 (ppm)

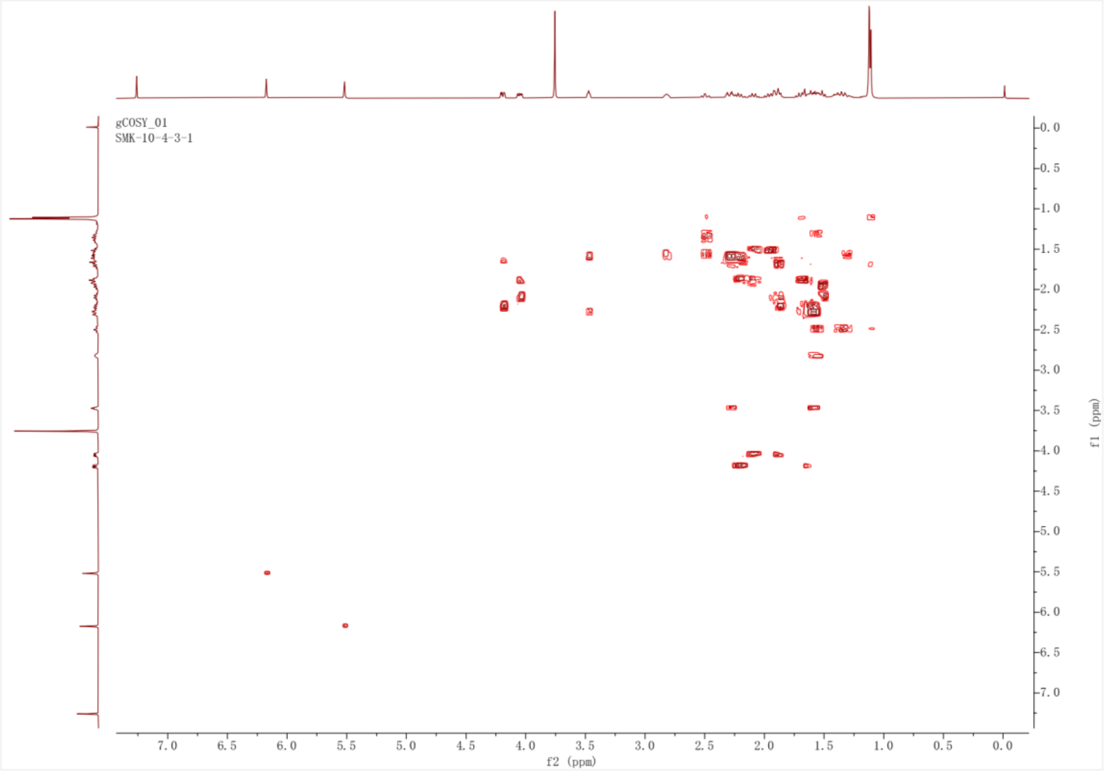
gCOSY_01
SMK-10-4-3-1
f2 (ppm)
f1 (ppm)

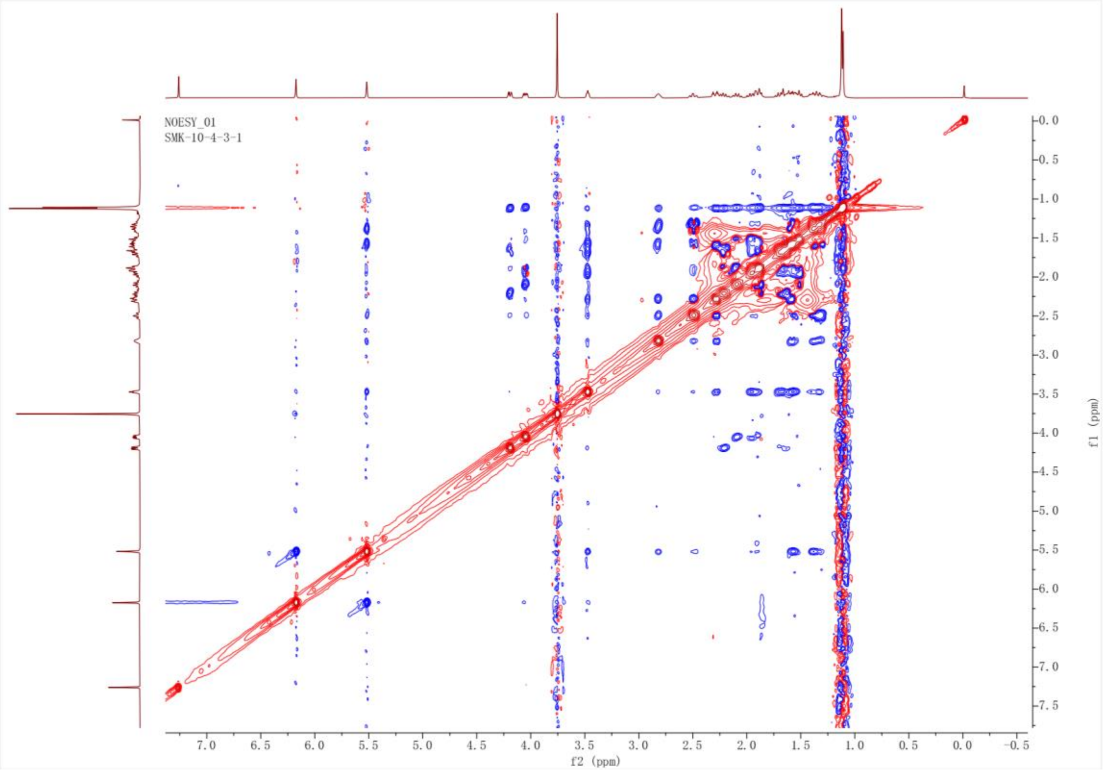
NOESY_01
SMK-10-4-3-1
f2 (ppm)
f1 (ppm)

Figure.S17 Spectra (HRESIMS, UV, ^{1}H NMR, ^{13}C NMR, HSQC, HMBC, ^{1}H–^{1}H COSY, NOESY) for compound 12

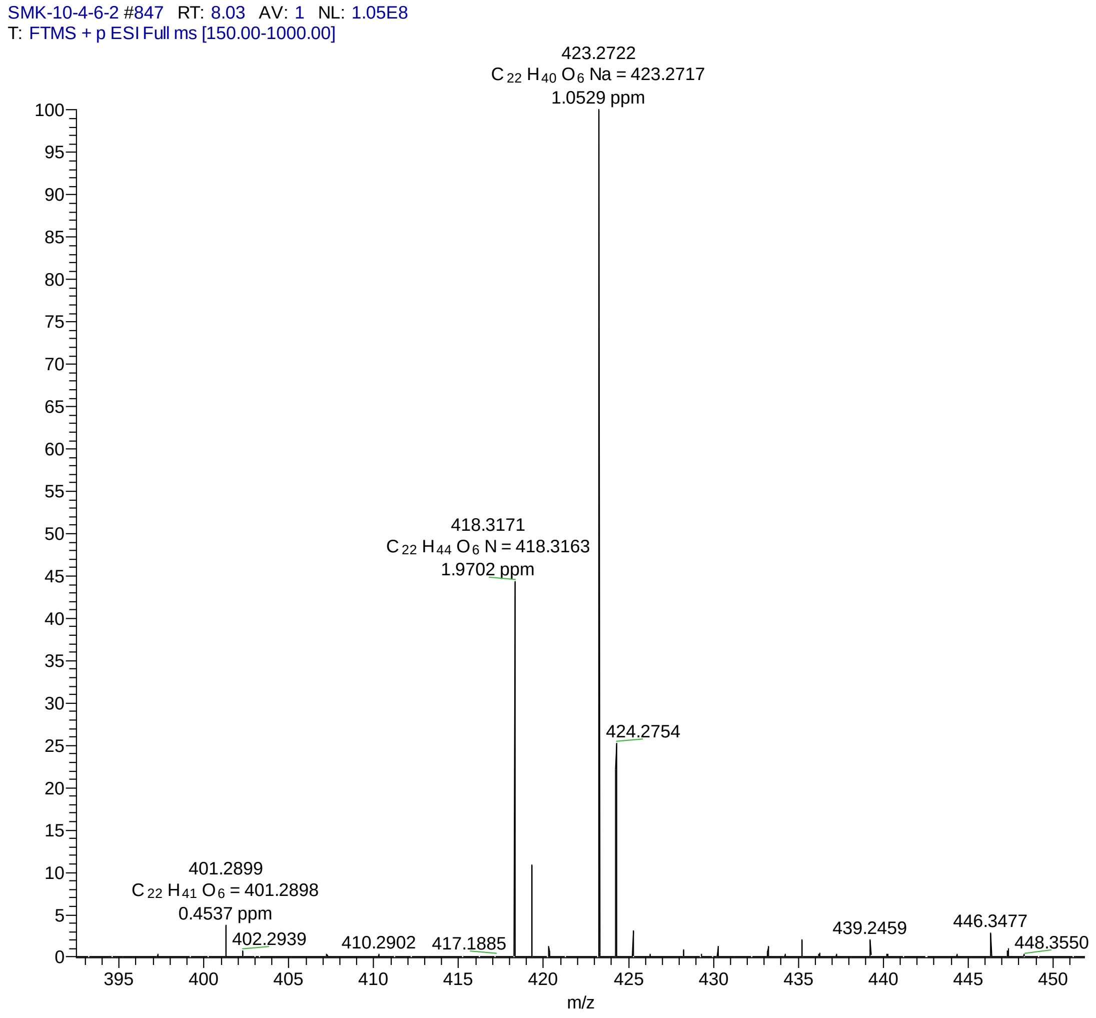

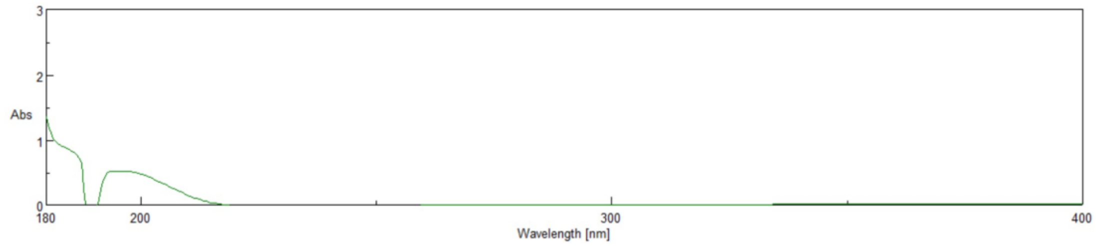

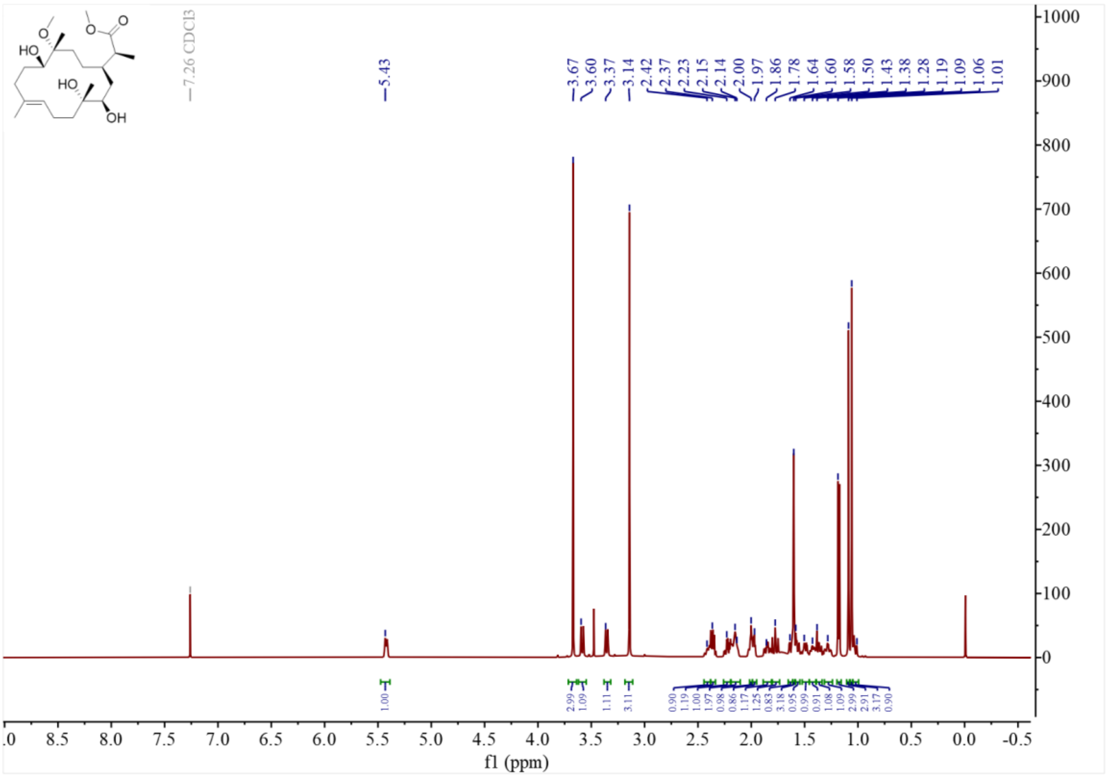
7.26 CDCl3
5.43
3.67
3.60
3.37
3.14
f1 (ppm)

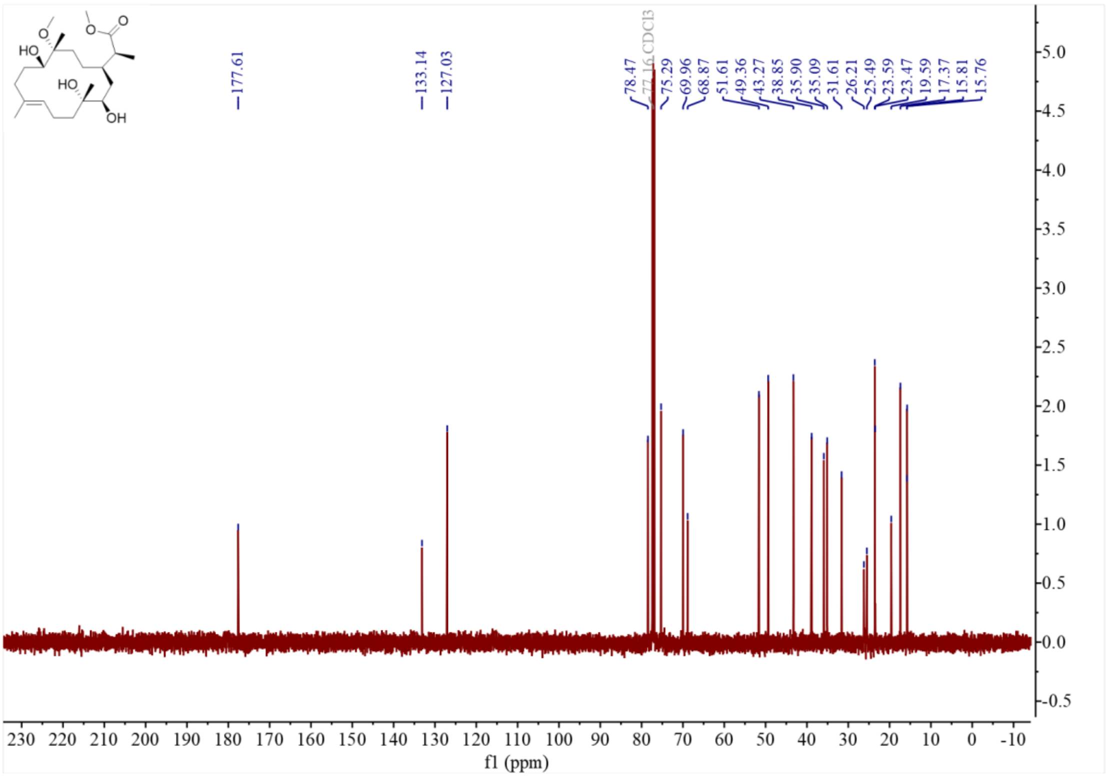
177.61
133.14
127.03
78.47
77.16 CDCl3
75.29
69.96
68.87
51.61
49.36
43.27
38.85
35.90
35.09
31.61
26.21
25.49
23.59
23.47
19.59
17.37
15.81
15.76
f1 (ppm)

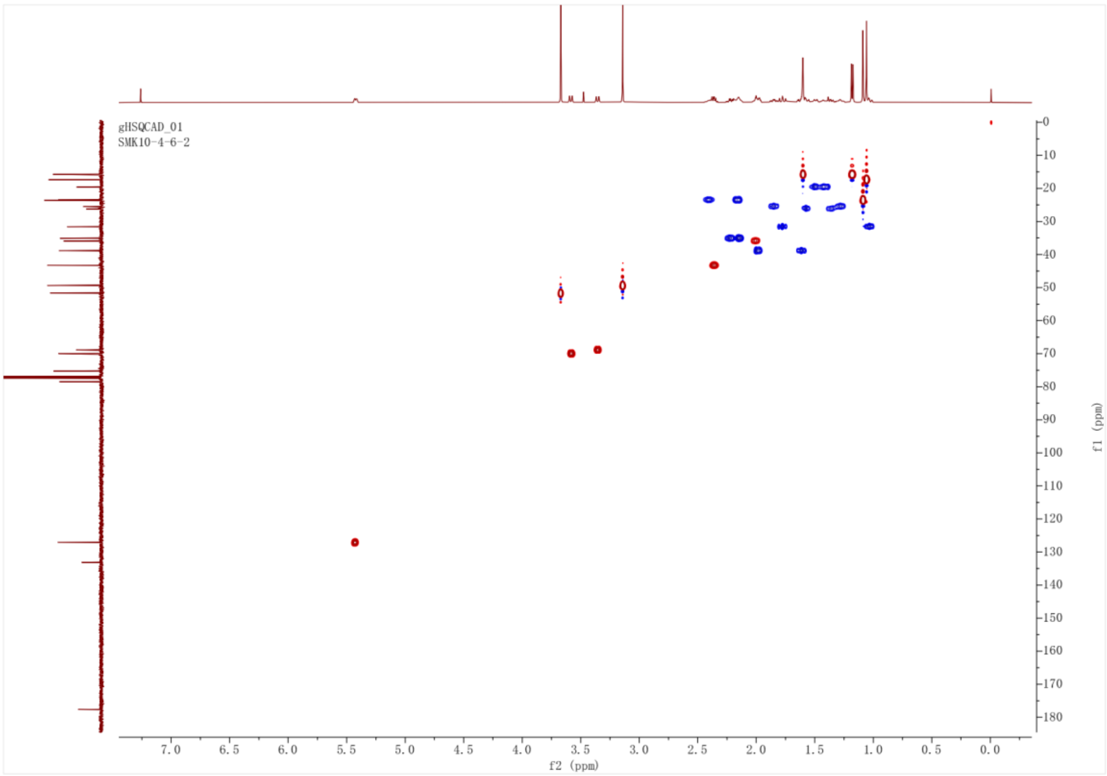
gHSQCAD_01
SMK10-4-6-2
f2 (ppm)
f1 (ppm)

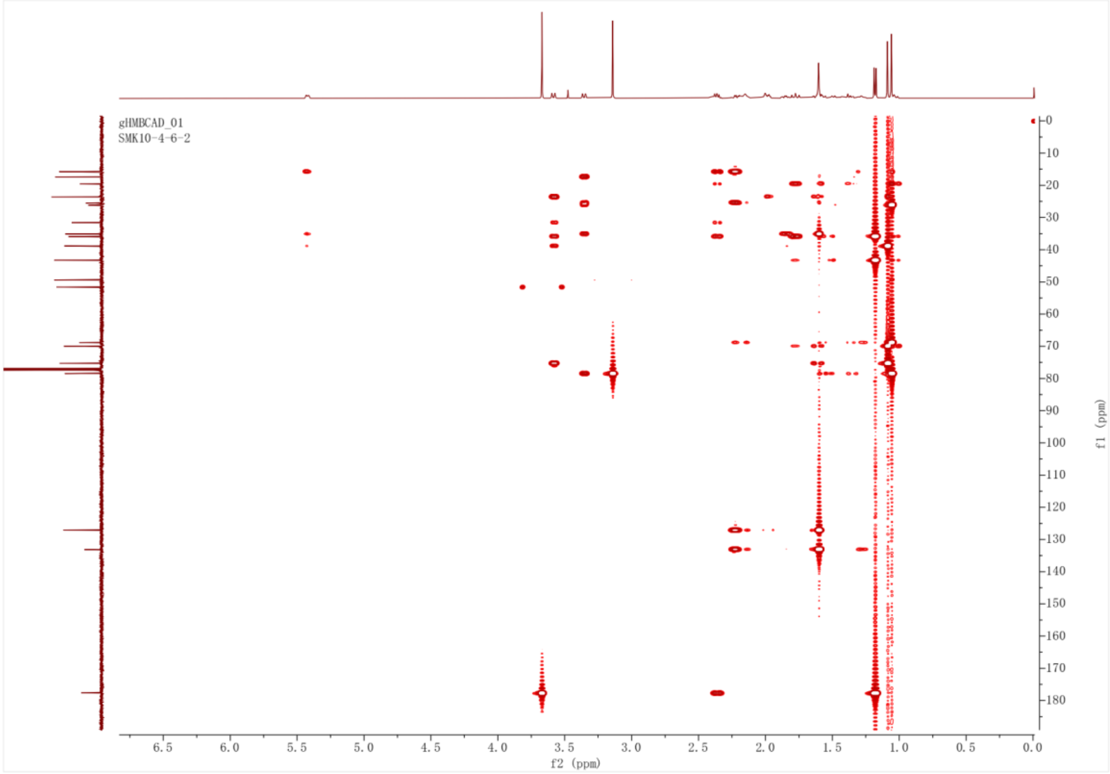
gHMBCAD_01
SMK10-4-6-2
f2 (ppm)
f1 (ppm)

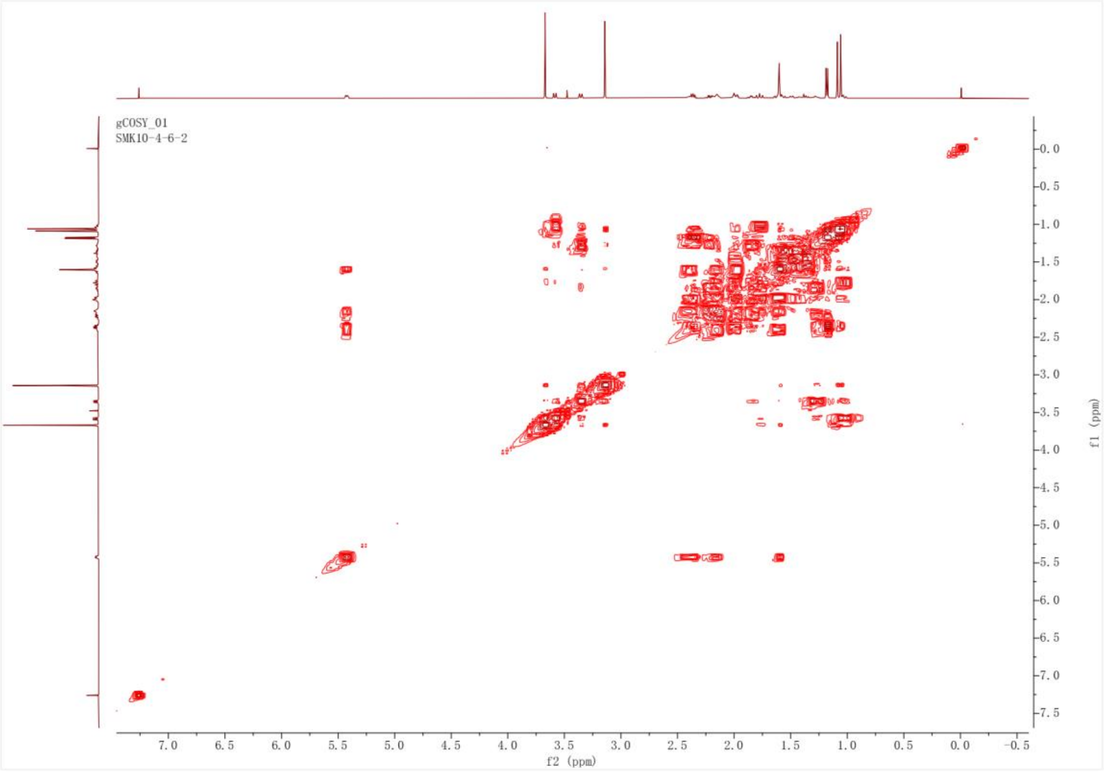
gCOSY_01
SMK10-4-6-2
f2 (ppm)
f1 (ppm)

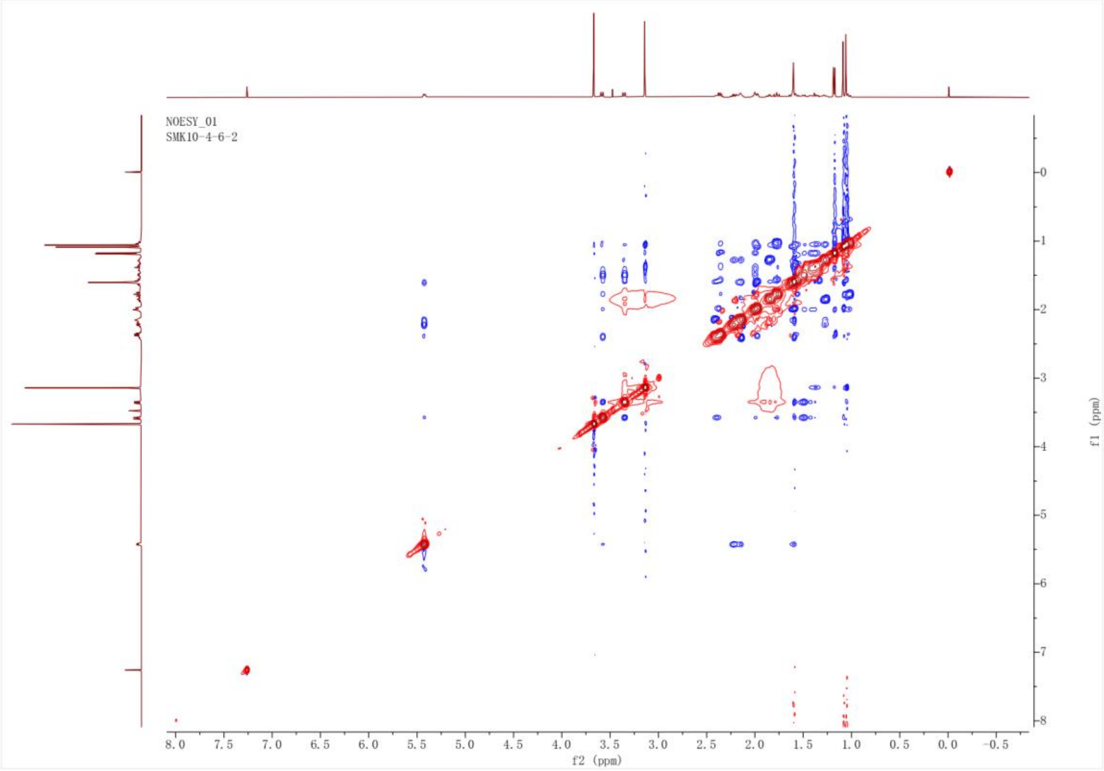
NOESY_01
SMK10-4-6-2
f2 (ppm)
f1 (ppm)

Figure.S18 Spectra (HRESIMS, UV, ^{1}H NMR, ^{13}C NMR, HSQC, HMBC, ^{1}H–^{1}H COSY, NOESY) for compound 13

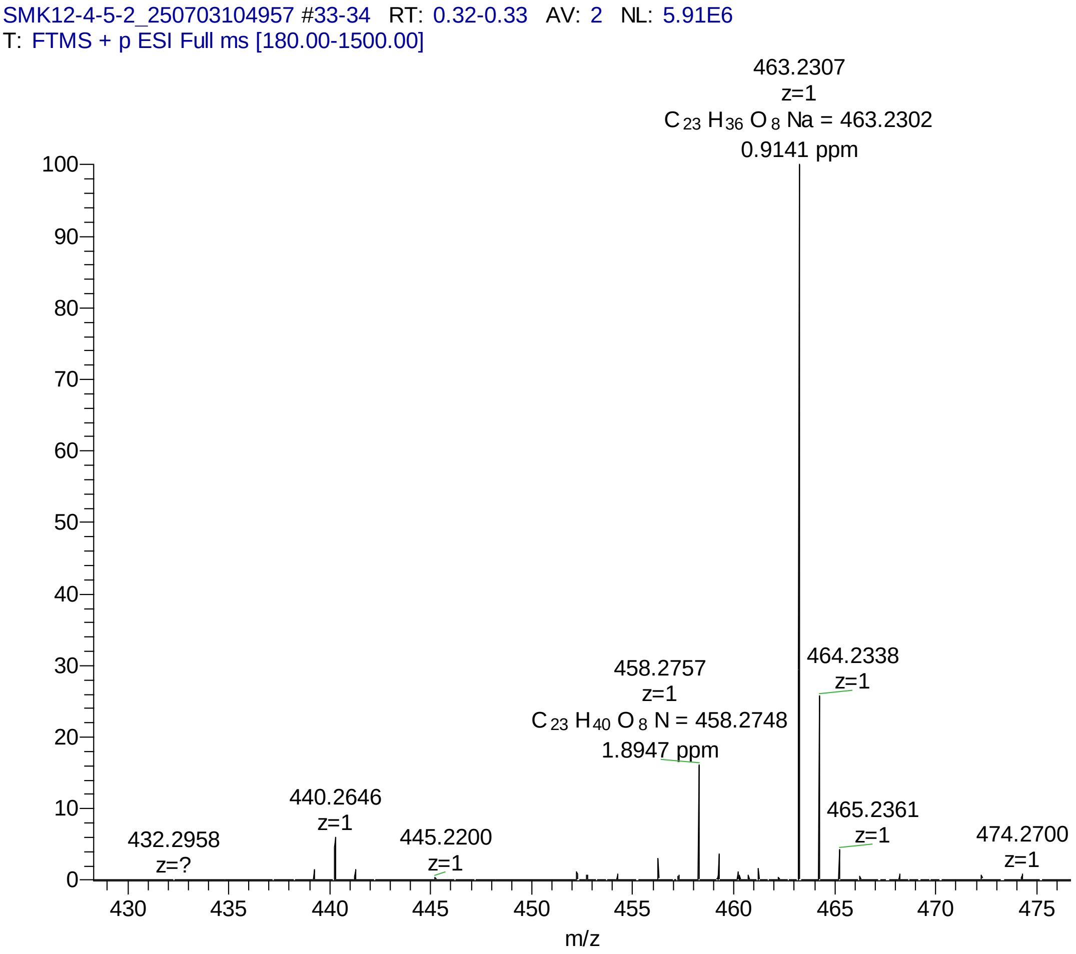

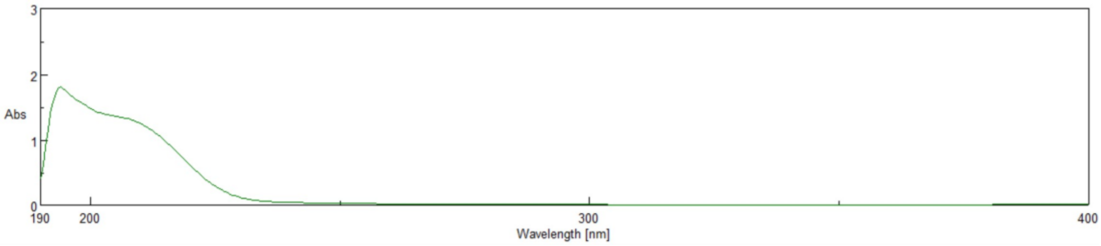

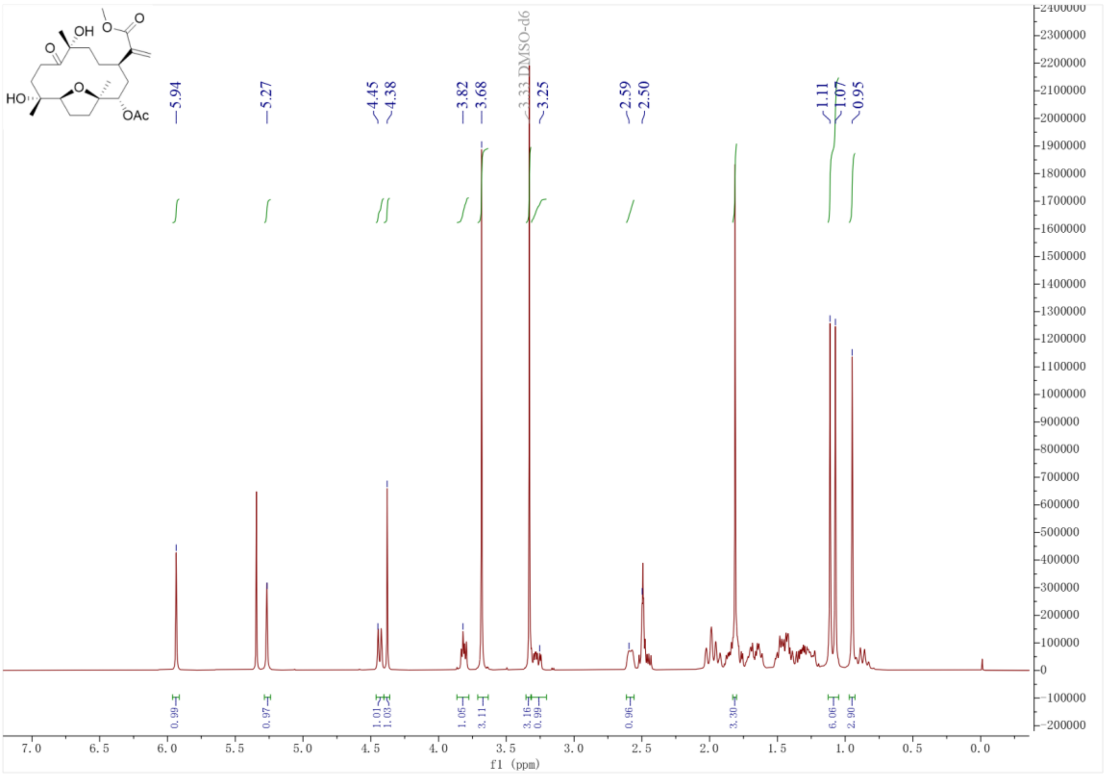
OH
O
O
O
HO
O
OAc
5.94
5.27
4.45
4.38
3.82
3.68
3.33 DMSO-d6
3.25
2.59
2.50
1.11
1.07
0.95
0.99
0.97
1.01
1.03
1.05
3.11
3.16
0.99
0.96
3.30
6.06
2.90
f1 (ppm)

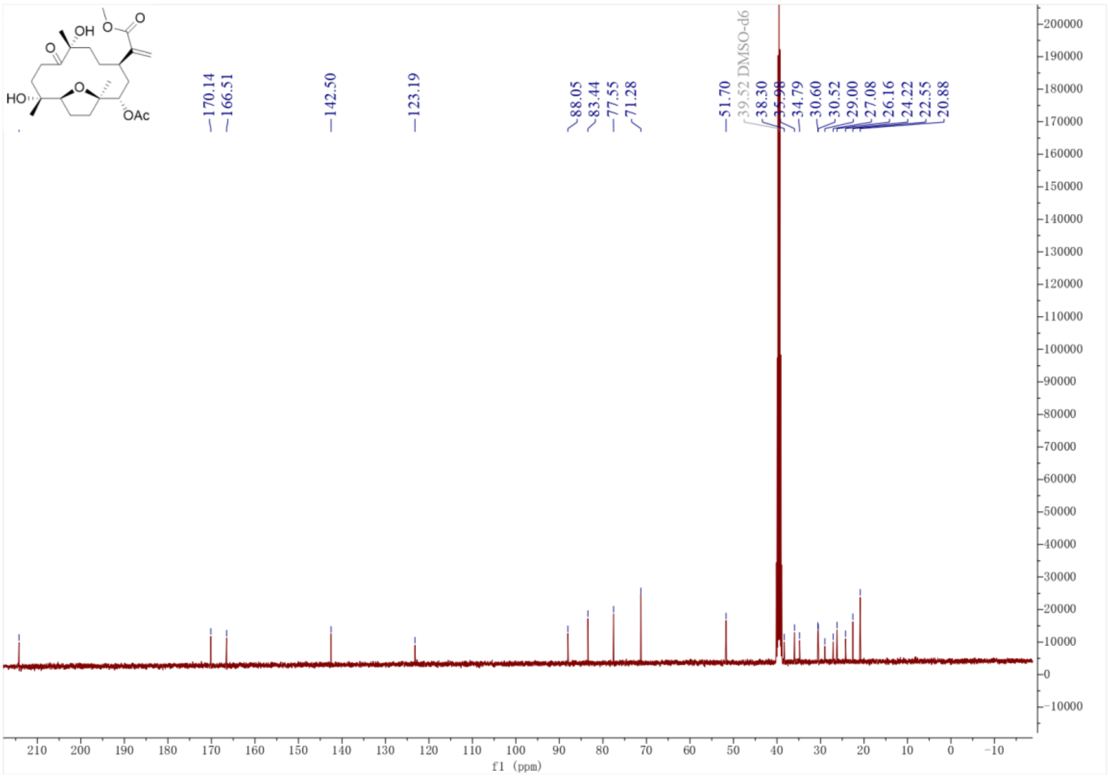
OH
O
O
O
HO
O
OAc
170.14
166.51
142.50
123.19
88.05
83.44
77.55
71.28
51.70
39.52 DMSO-d6
38.30
35.98
34.79
30.60
30.52
29.00
27.08
26.16
24.22
22.55
20.88
f1 (ppm)

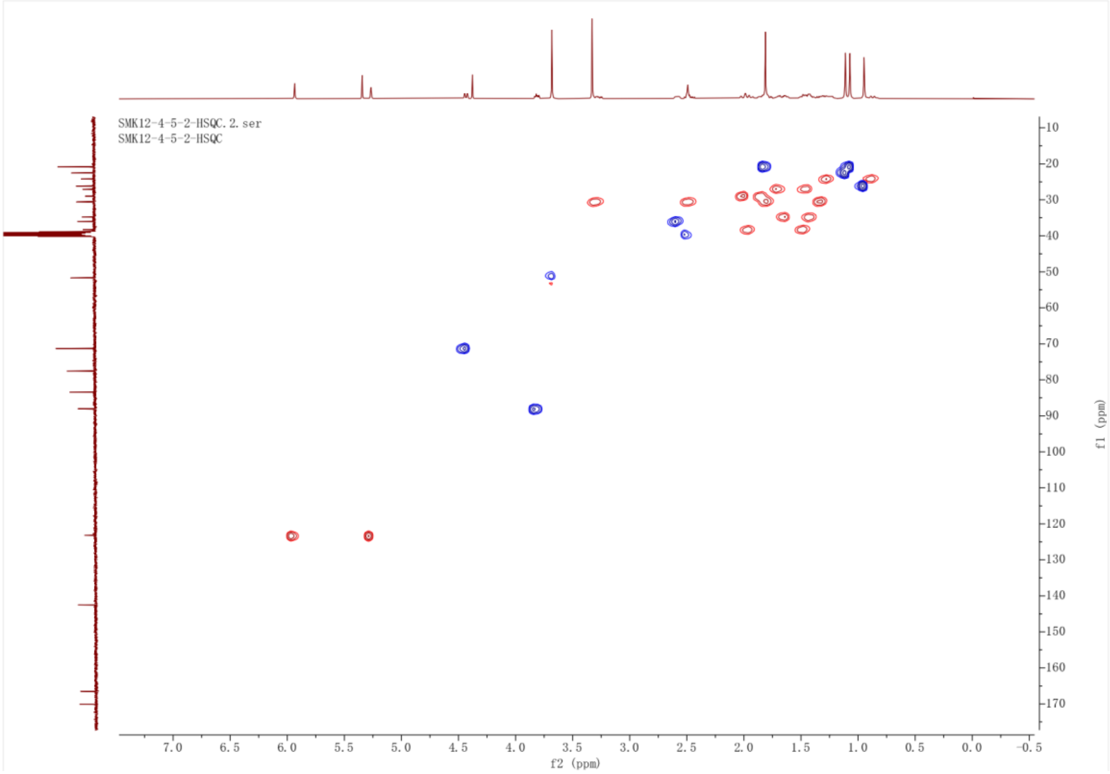
SMK12-4-5-2-HSQC.2.ser
SMK12-4-5-2-HSQC
f2 (ppm)
f1 (ppm)

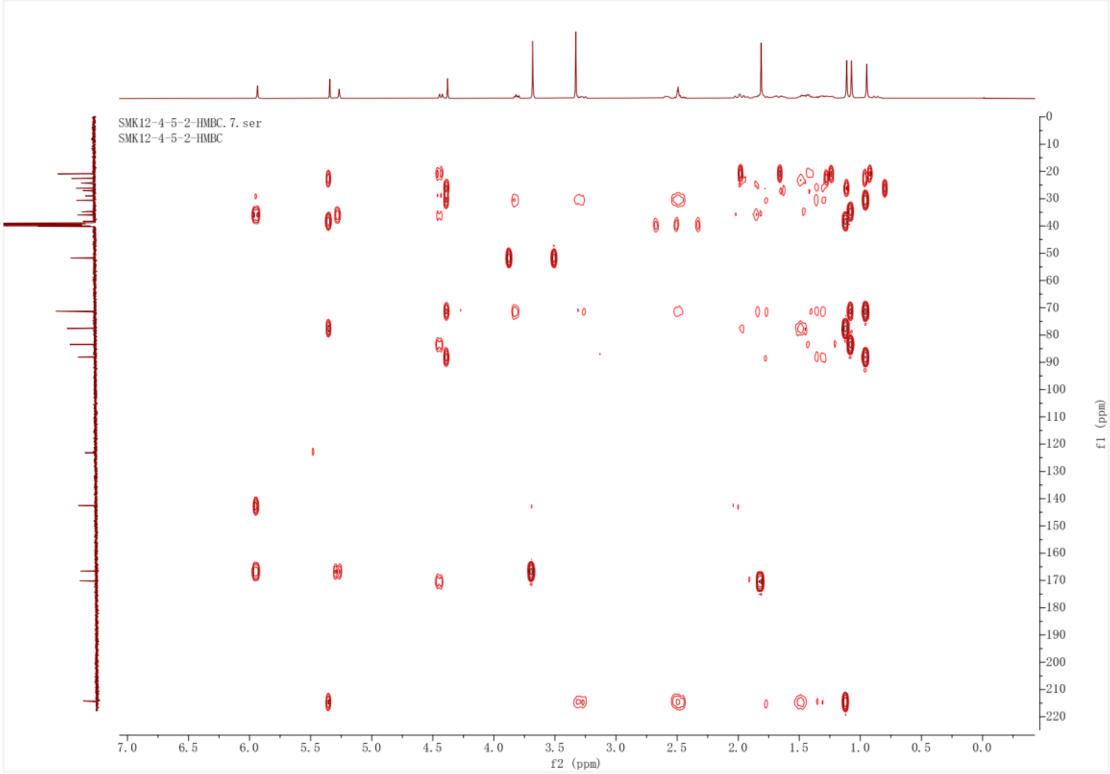
SMK12-4-5-2-HMBC.7.ser
SMK12-4-5-2-HMBC
f2 (ppm)
f1 (ppm)

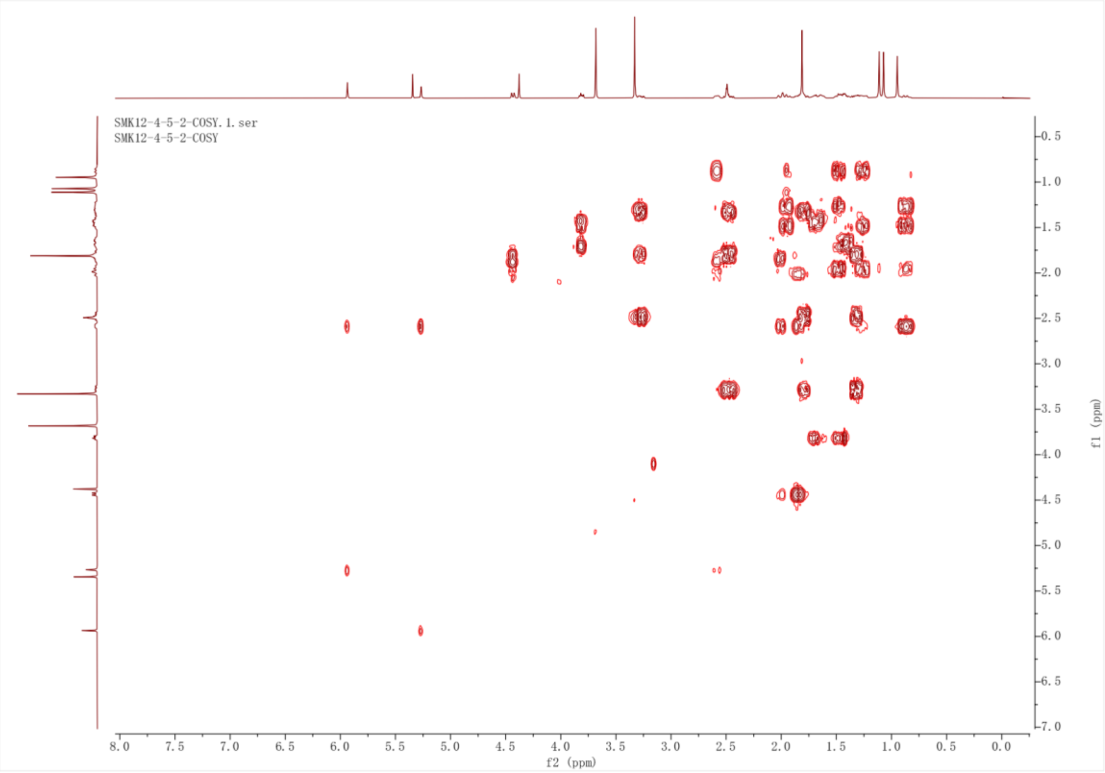
SMK12-4-5-2-COSY.1.ser
SMK12-4-5-2-COSY
f2 (ppm)
f1 (ppm)

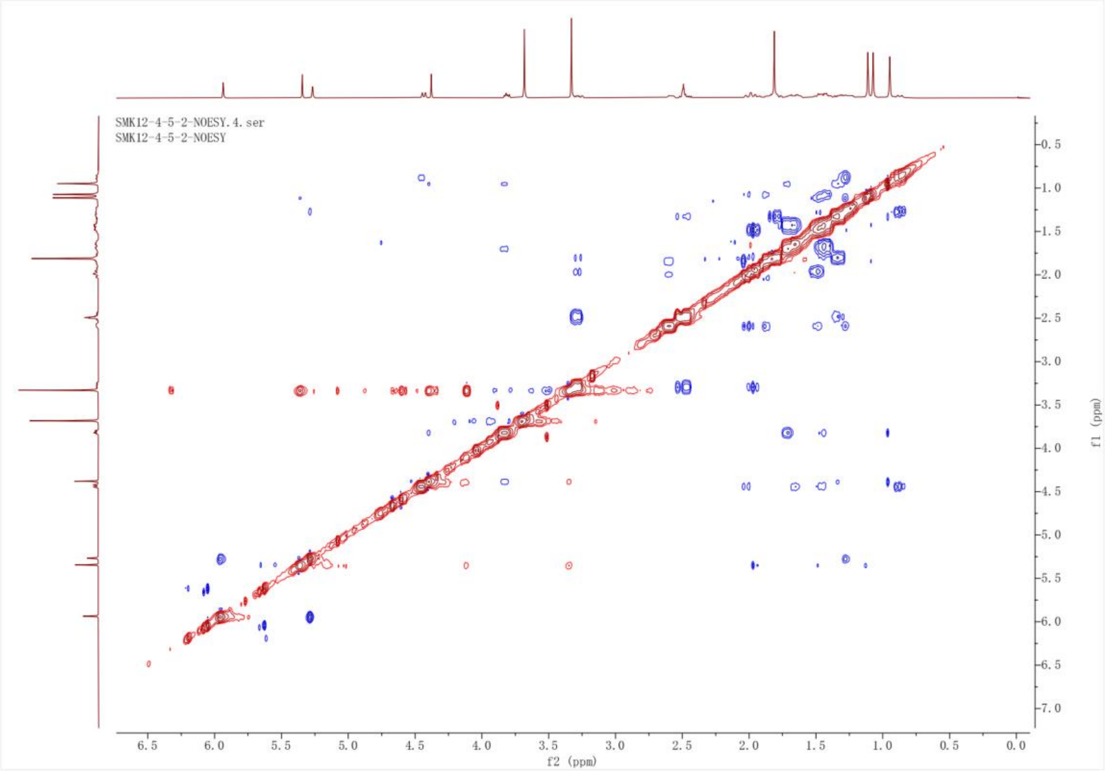
SMK12-4-5-2-NOESY.4.ser
SMK12-4-5-2-NOESY
f2 (ppm)
f1 (ppm)

Figure.S19 Spectra (HRESIMS, UV, ^{1}H NMR, ^{13}C NMR, HSQC, HMBC, ^{1}H–^{1}H COSY, NOESY) for compound 14

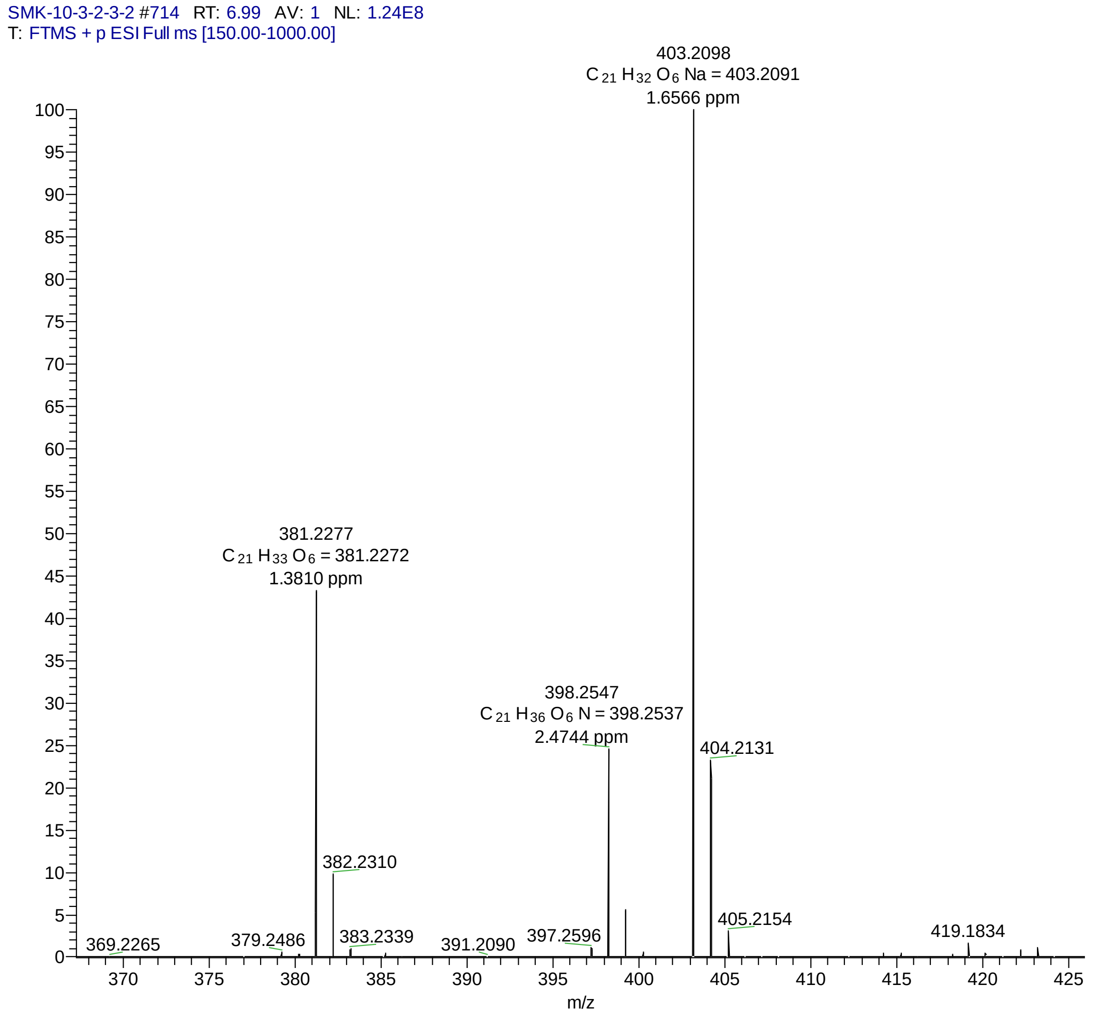

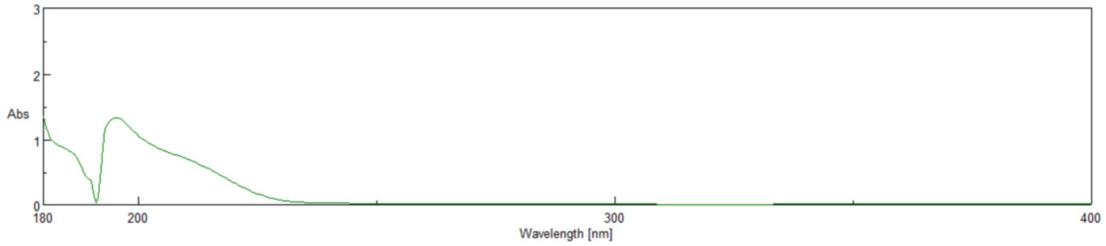

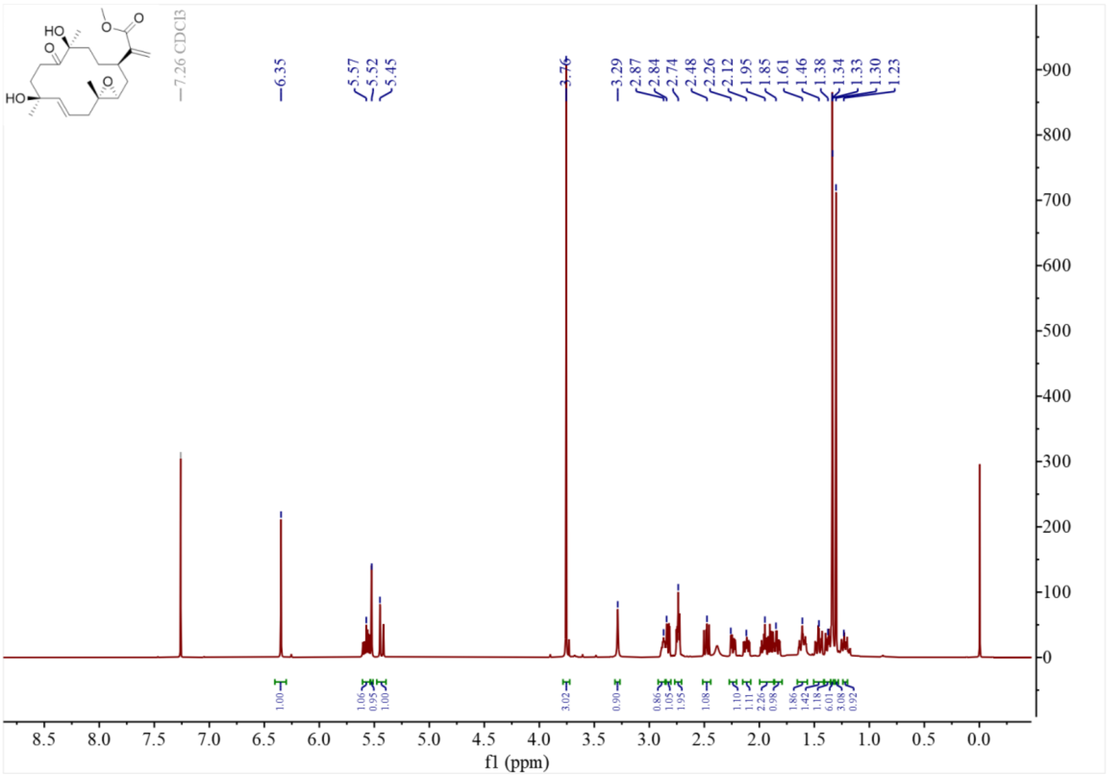
7.26 CDCl3
6.35
5.57
5.52
5.45
3.76
3.29
2.87
2.84
2.74
2.48
2.26
2.12
1.95
1.85
1.61
1.46
1.38
1.34
1.33
1.30
1.23
1.00
1.06
0.95
1.00
3.02
0.90
0.86
1.05
1.95
1.08
1.10
1.11
2.26
0.98
1.86
1.42
1.18
6.01
3.08
0.92
f1 (ppm)

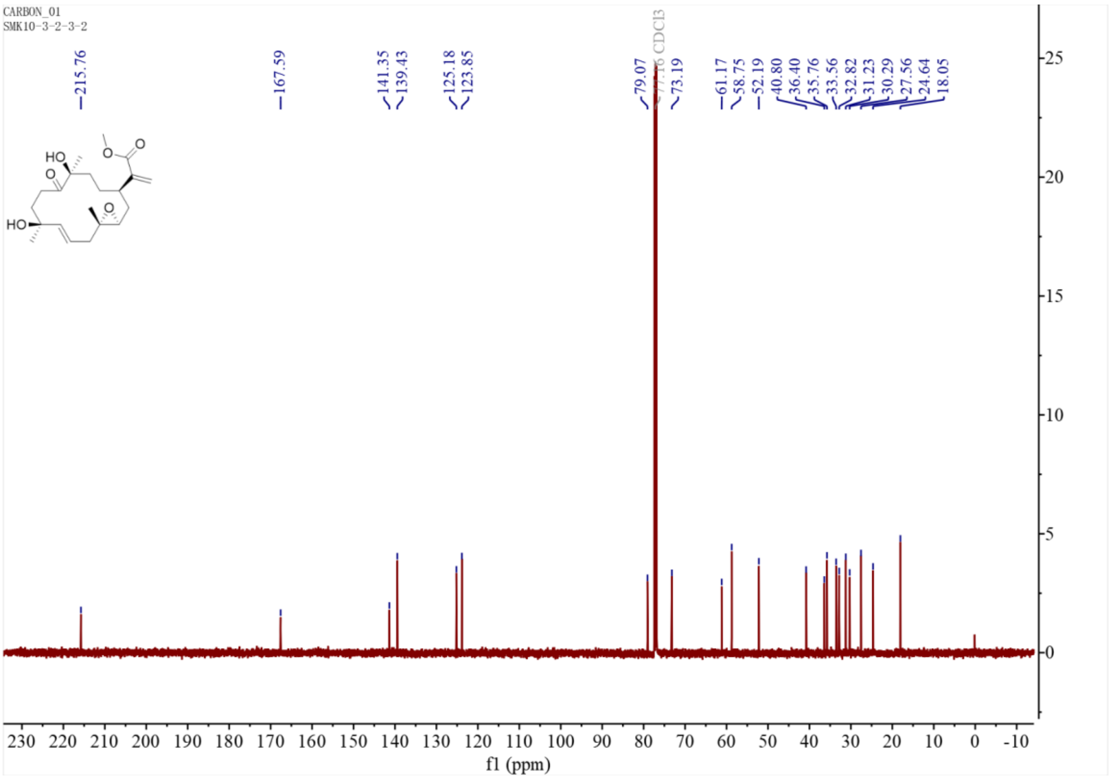
CARBON_01
SMK10-3-2-3-2
215.76
167.59
141.35
139.43
125.18
123.85
79.07
77.16 CDCl3
73.19
61.17
58.75
52.19
40.80
36.40
35.76
33.56
32.82
31.23
30.29
27.56
24.64
18.05
f1 (ppm)

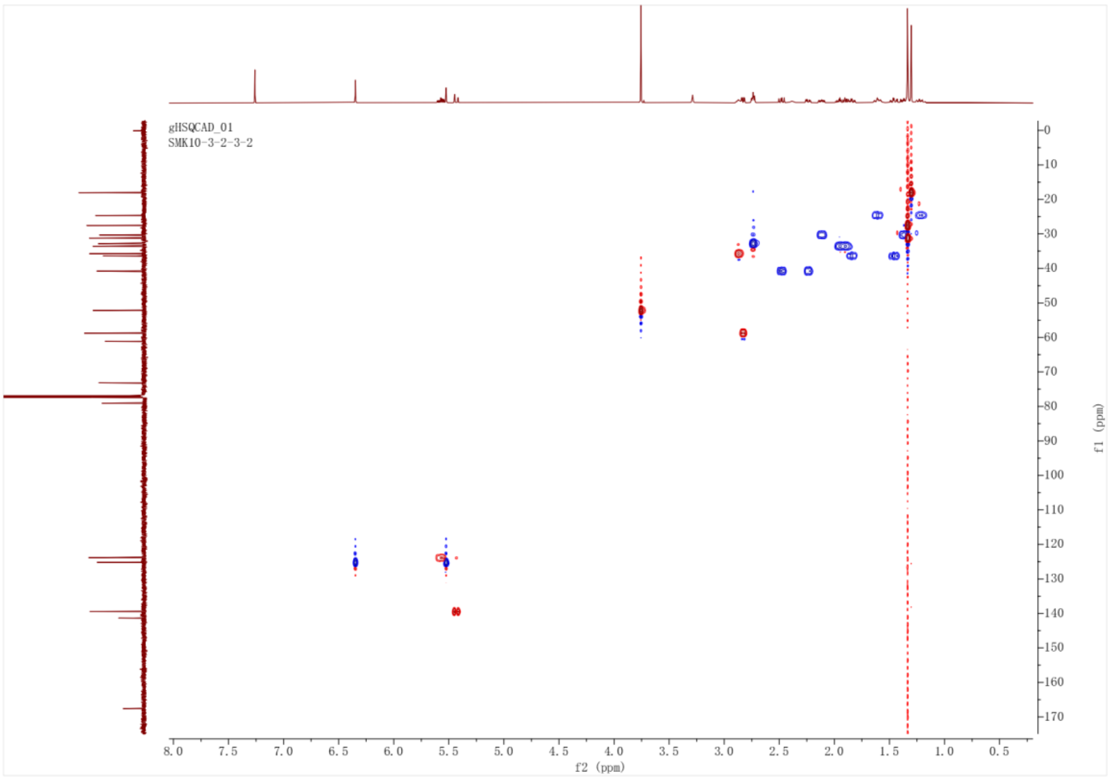
gHSQCAD_01
SMK10-3-2-3-2
f2 (ppm)
f1 (ppm)

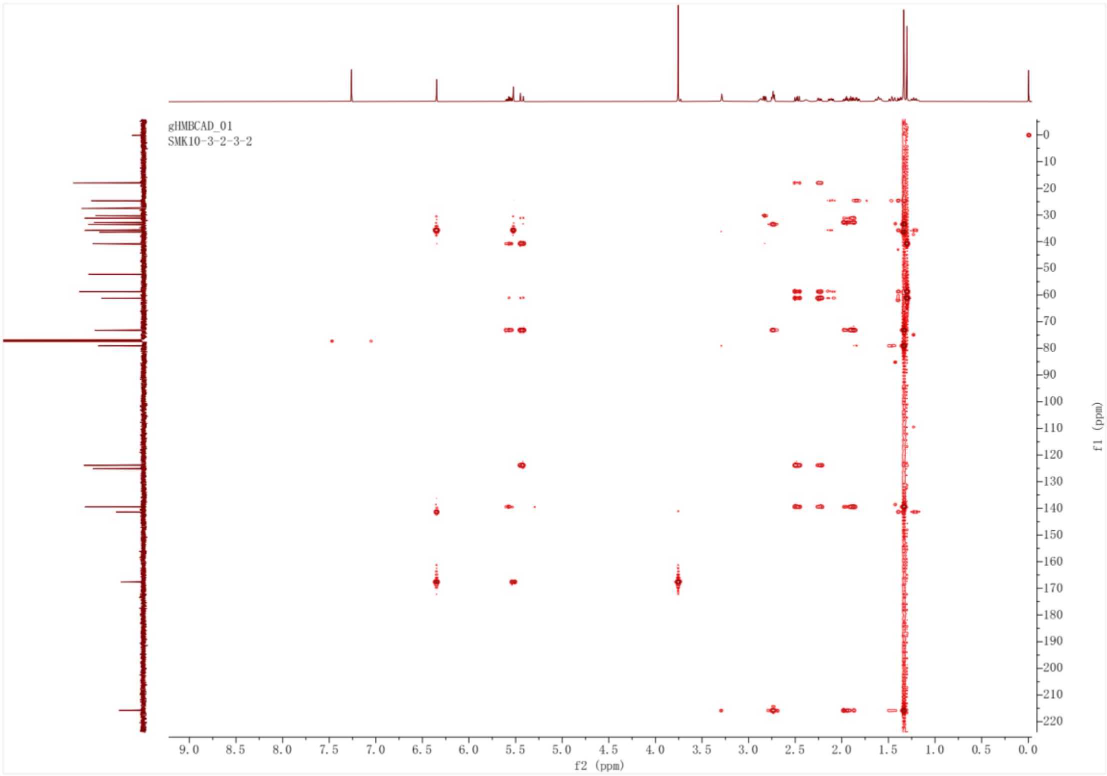
gHMBCAD_01
SMK10-3-2-3-2
f2 (ppm)
f1 (ppm)

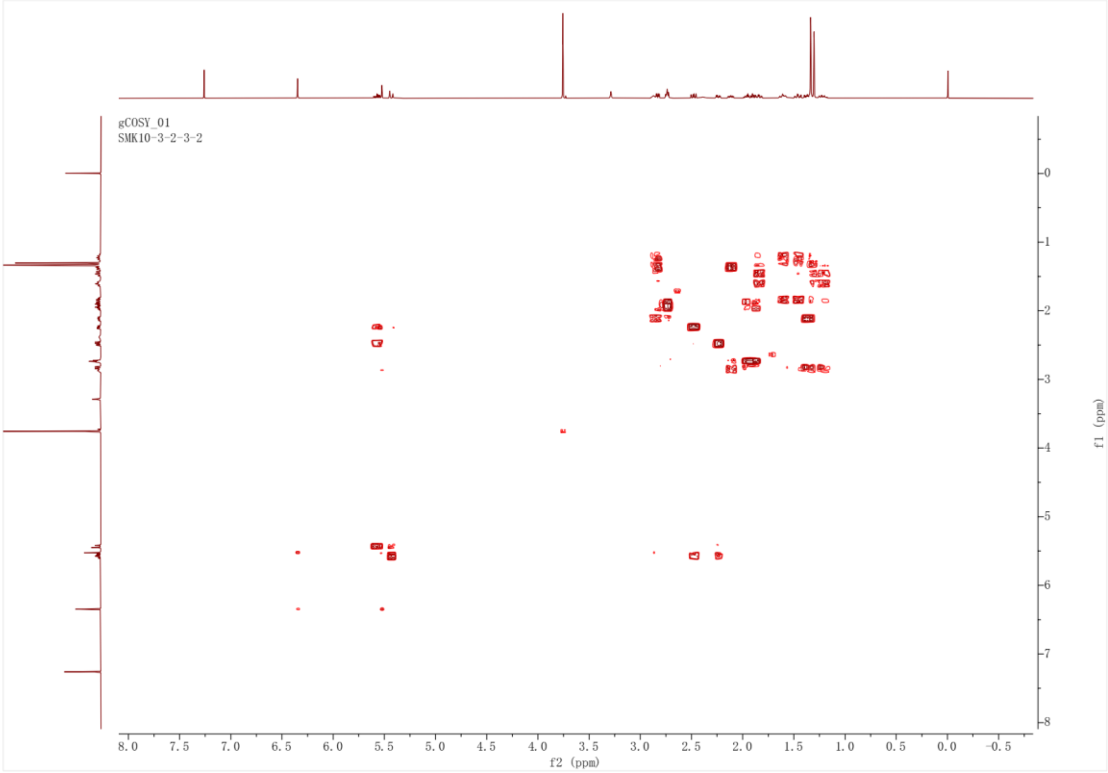

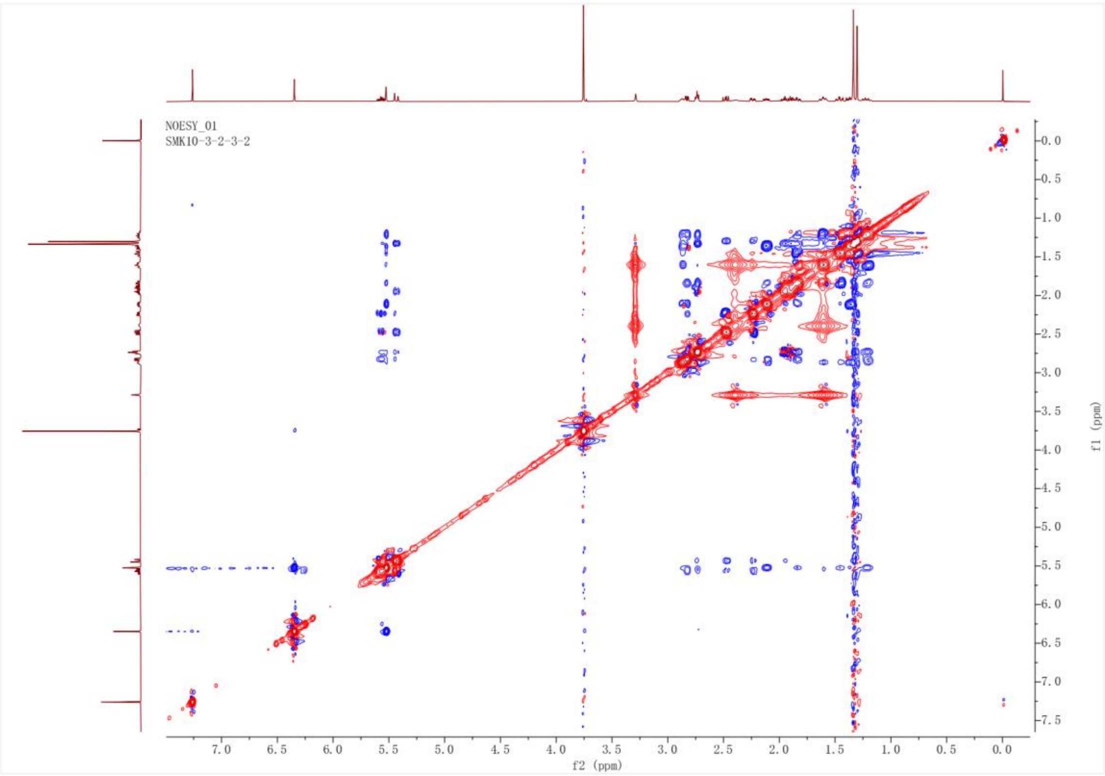

4. X-ray crystallographic analysis of 1,2,5,12, and 13

Table S1.Crystallographic data of 1

$C_{20}H_{32}O_5$, M=352. $C_{20}H_{34}O_6$, M_r = 370.47, triclinic, P1 (No. 1), a = 9.7362(12) Å, b = 9.9767(12) Å, c = 11.4605(12) Å, α= 78.550(8) °, β = 78.142(7) °, γ = 75.476(8) °, V = 1041.9(2) Å^3, T = 170.00 K, Z = 2, Z′ = 2, μ (Cu Kα) = 0.700, 24238 reflections measured, 7292 unique (R_{int} = 0.0823) which were used in all calculations. The final wR_2 was 0.2289 (all data) and R_1 was 0.0730

($I > 2\sigma(I)$). Flack parameter = 0.05 (17).

Identification code	cu_20250116B_0m_sq
Empirical formula	$C_{20}H_{34}O_6$
Formula weight	370.47
Temperature/K	170.00
Crystal system	triclinic
Space group	P1
a/Å	9.7362(12)
b/Å	9.9767(12)
c/Å	11.4605(12)
α/°	78.550(8)
β/°	78.142(7)
γ/°	75.476(8)
Volume/Å^3	1041.9(2)
Z	2
ρ_{calc}g/cm^3	1.181
μ/mm^{-1}	0.700
F (000)	404.0
Crystal size/mm^3	0.04 × 0.03 × 0.01
Radiation	Cu Kα (λ = 1.54178)
2Θ range for data collection/°	7.98 to 143.048
Index ranges	-11 ≤ h ≤ 11, -12 ≤ k ≤ 12, -13 ≤ l ≤ 13
Reflections collected	24238
Independent reflections	7292 [R_{int} = 0.0823, R_{sigma} = 0.0746]
Data/restraints/parameters	7292/5/486
Goodness-of-fit on F^2	1.094
Final R indexes [$I > 2\sigma(I)$]	R_1 = 0.0730, wR_2 = 0.2031
Final R indexes [all data]	R_1 = 0.0975, wR_2 = 0.2289
Largest diff. peak/hole / e Å^{-3}	0.35/-0.25
Flack parameter	0.05(17)

Table S2.Crystallographic data of 2

$C_{20}H_{36}O_6$, M_r = 372.49, orthorhombic, $P2_12_12_1$ (No. 19), a = 10.7089(3) Å, b = 11.4405(3) Å, c = 17.4612(4) Å, $\alpha = \beta = \gamma$ = 90°, V = 2139.26(10) Å^3, T = 170.00 K, Z = 4, Z' = 1, μ (Cu Kα) = 0.682, 20705 reflections measured, 4397 unique (R_{int} = 0.0590) which were used in all calculations.

The final wR_2 was 0.0874 (all data) and R_1 was 0.0364 ($I > 2\sigma(I)$). Flack parameter = –0.06 (11).

Identification code	cu_20250116A_0m
Empirical formula	$C_{20}H_{36}O_6$
Formula weight	372.49
Temperature/K	170.00
Crystal system	orthorhombic
Space group	$P2_12_12_1$
a/Å	10.7089(3)
b/Å	11.4405(3)
c/Å	17.4612(4)

α/°	90
β/°	90
γ/°	90
Volume/Å^3	2139.26(10)
Z	4
ρ_{calc}g/cm^3	1.157
μ/mm^{-1}	0.682
F (000)	816.0
Crystal size/mm^3	0.1 × 0.06 × 0.01
Radiation	Cu Kα (λ = 1.54178)
2Θ range for data collection/°	9.242 to 150.646
Index ranges	-12 ≤ h ≤ 13, -14 ≤ k ≤ 12, -21 ≤ l ≤ 21
Reflections collected	20705
Independent reflections	4397 [R_{int} = 0.0590, R_{sigma} = 0.0386]
Data/restraints/parameters	4397/0/245
Goodness-of-fit on F^2	1.051
Final R indexes [$I > 2\sigma(I)$]	R_1 = 0.0364, wR_2 = 0.0804
Final R indexes [all data]	R_1 = 0.0479, wR_2 = 0.0874
Largest diff. peak/hole / e Å^{-3}	0.14/-0.15
Flack parameter	-0.06(11)

Table S3.Crystallographic data of 5

$C_{22}H_{35.5}O_{7.75}$, M_r = 424.00, monoclinic, $P2_1$ (No. 4), a = 11.4438(3) Å, b = 31.2466(7) Å, c = 13.7686(4) Å, α = 112.317(2) °, $\beta = \gamma$ = 90°, V = 4554.6(2) Å^3, T = 295.00 K, Z = 8, Z' = 4, μ (CuKα) = 0.765, 67167 reflections measured, 18285 unique (R_{int} = 0.0747) which were used in all calculations. The final wR_2 was 0.1322 (all data) and R_1 was 0.0463 ($I > 2\sigma(I)$). Flack parameter = –0.06 (8).

Identification code	cu_20250117A_0m
Empirical formula	$C_{22}H_{35.5}O_{7.75}$
Formula weight	424.00
Temperature/K	295.00
Crystal system	monoclinic
Space group	P2$_1$
a/Å	11.4438(3)
b/Å	31.2466(7)
c/Å	13.7686(4)
α/°	90
β/°	112.317(2)
γ/°	90
Volume/Å^3	4554.6(2)
Z	8
ρ_{calc}g/cm^3	1.237
μ/mm^{-1}	0.765
F (000)	1836.0
Crystal size/mm^3	0.08 × 0.04 × 0.01
Radiation	Cu Kα (λ = 1.54178)

2Θ range for data collection/°	5.656 to 150.352
Index ranges	$-14 \le h \le 14$, $-39 \le k \le 39$, $-17 \le l \le 12$
Reflections collected	67167
Independent reflections	18285 [R_{int} = 0.0747, R_{sigma} = 0.0563]
Data/restraints/parameters	18285/3/1105
Goodness-of-fit on F^2	1.007
Final R indexes [$I > 2\sigma(I)$]	R_1 = 0.0463, wR_2 = 0.1109
Final R indexes [all data]	R_1 = 0.0748, wR_2 = 0.1322
Largest diff. peak/hole / e Å^{-3}	0.20/-0.24
Flack parameter	-0.06(8)

Table S4.Crystallographic data of 12

$C_{22}H_{42}O_7$, *Mr* = 418.55 g/mol, orthorhombic, space group P1, *a* = 10.1827(3) Å, *b* = 11.6231(4) Å, *c* = 12.2652(4) Å, α = 115.332(2)°, β = 111.536(2)°, γ = 94.871(2)°, *V* = 1168.82(7) Å3, *T* = 150 (2) K, *Z* = 2, μ (Cu Kα) = 0.707 mm^{-1}, 12974 reflections measured, 7651 independent reflections [R_{int} = 0.0278, R_{sigma} = 0.0342]. The final R1 value was 0.0785 ($I > 2\sigma(I)$). The final wR_2 value was 0.1792 ($I > 2\sigma(I)$). The final R1 value was 0.0807 (all data). Flack parameter = 0.09 (10).

Identification code	cu_0724_14_0m
Empirical formula	$C_{22}H_{42}O_7$
Formula weight	418.55
Temperature/K	150.00
Crystal system	triclinic
Space group	P1
a/Å	10.1827(3)
b/Å	11.6231(4)
c/Å	12.2652(4)
α/°	115.332(2)
β/°	111.536(2)
γ/°	94.871(2)
Volume/Å^3	1168.82(7)
Z	2
ρ_{calc}g/cm^3	1.189
μ/mm^{-1}	0.707
F (000)	460.0
Crystal size/mm^3	0.2 × 0.15 × 0.1
Radiation	Cu Kα (λ = 1.54178)
2Θ range for data collection/°	8.788 to 150.152
Index ranges	$-12 \le h \le 12$, $-14 \le k \le 14$, $-13 \le l \le 15$
Reflections collected	12974
Independent reflections	7651 [R_{int} = 0.0278, R_{sigma} = 0.0342]
Data/restraints/parameters	7651/3/547
Goodness-of-fit on F^2	1.083
Final R indexes [$I > 2\sigma(I)$]	R_1 = 0.0785, wR_2 = 0.1792
Final R indexes [all data]	R_1 = 0.0807, wR_2 = 0.1826
Largest diff. peak/hole / e Å^{-3}	0.49/-0.24
Flack parameter	0.09(10)

Table S5.Crystallographic data of 13

$C_{23}H_{36}O_8$•0.3(CH_4O) •0.5(H_2O), M = 459.14 g/mol, orthorhombic, space group $P2_12_12$ (no. 18), a = 10.5323(4) Å, b = 24.6245(9) Å, c = 10.1393(4) Å, α = 90°, β = 90°, γ = 90°, V = 2629.65(17) Å^3, T = 150(2) K, Z = 4, μ (Cu Kα) = 0.729 mm^{-1}, 36181 reflections measured, 5388 independent reflections (R_{int} = 0.0746). The final R_1 value was 0.0763 ($I > 2\sigma(I)$). The final $wR(F^2)$ value was 0.2196 ($I > 2\sigma(I)$). The final R_1 value was 0.0843 (all data). The final $wR(F^2)$ value was 0.2322 (all data). The goodness of fit on F^2 was 1.041. Flack parameter = –0.02(8).

Identification code	SMK12-4-5-2
Empirical formula	$C_{23.3}H_{38.2}O_{8.8}$
Formula weight	459.14
Temperature/K	150(2)
Crystal system	orthorhombic
Space group	$P2_12_12$
a/Å	10.5323(4)
b/Å	24.6245(9)
c/Å	10.1393(4)
α/°	90
β/°	90
γ/°	90
Volume/Å^3	2629.65(17)
Z	4
ρ_{calc}g/cm^3	1.160
μ/mm^{-1}	0.729
F (000)	994.0
Crystal size/mm^3	0.09 × 0.08 × 0.04
Radiation	Cu Kα (λ = 1.54178)
2Θ range for data collection/°	9.132 to 149.882
Index ranges	$-13 \le h \le 13$, $-30 \le k \le 29$, $-12 \le l \le 12$
Reflections collected	36181
Independent reflections	5388 [R_{int} = 0.0746, R_{sigma} = 0.0417]
Data/restraints/parameters	5388/19/314
Goodness-of-fit on F^2	1.041
Final R indexes [$I > 2\sigma(I)$]	R_1 = 0.0763, wR_2 = 0.2196
Final R indexes [all data]	R_1 = 0.0843, wR_2 = 0.2322
Largest diff. peak/hole / e Å^{-3}	0.77/-0.46
Flack parameter	-0.02(8)

5. Calculation Details

Conformational analyses were performed by the Spartan 10 software package using the MMFF minimization force field. The resulted conformers were further optimized with Gaussian 09 software by the density functional theory (DFT) calculations at the B3LYP/6-31+G(d,p) level in the gas phase. The optimized conformations, whose Boltzmann distributions of Gibbs free energies were more than 1.0 percent, were used for the NMR calculations using the GIAO method at the PCM/B3LYP/6-311+G(d,p) level, or the ECD using TD-DFT method with the basis set RB3LYP/DGDZVP.

Table S6.Calculation process of 1

Important thermodynamic parameters (a.u.) of the optimized **1** with simplified structures at B3LYP/6-31+G(d,p) level in the gas phase.

NO.	E+ZPE	G	p%	NO.	E+ZPE	G	p%	NO.	E+ZPE	G	p%
1a-1	-1157.51629	-1157.068813	16.70%	1a-9	-1157.515008	-1157.067445	3.92%	1b-1	-1157.514376	-1157.066515	1.19%
1a-2	-1157.516643	-1157.068973	19.79%	1a-10	-1157.516913	-1157.068046	7.41%	1b-2	-1157.518057	-1157.069535	29.24%
1a-3	-1157.515308	-1157.066971	2.37%	1a-11	-1157.514965	-1157.066402	1.30%	1b-3	-1157.518057	-1157.069539	29.37%
1a-4	-1157.516757	-1157.068271	9.41%	1a-12	-1157.515149	-1157.066182	1.03%	1b-4	-1157.515174	-1157.06685	1.70%
1a-5	-1157.515546	-1157.067864	6.11%	1a-13	-1157.51545	-1157.067678	5.02%	1b-5	-1157.518278	-1157.069579	30.64%
1a-6	-1157.515601	-1157.066859	2.11%	1a-14	-1157.516373	-1157.068322	9.93%	1b-6	-1157.514061	-1157.066837	1.68%
1a-7	-1157.515843	-1157.067932	6.57%	1a-15	-1157.515741	-1157.066267	1.12%				
1a-8	-1157.514926	-1157.066338	1.21%	1a-16	-1157.515203	-1157.067772	5.54%				

Optimized Z-Matrixes of **1** with simplified structures in the Gas Phase (Å) at B3LYP/6-31+G(d,p) level.

1a-1				1a-2				1a-3			
C	-0.61894	1.96769	-1.71484	C	-2.09758	-0.91285	-1.3702	C	-1.91549	-1.25968	-1.27815
C	-0.97995	3.16057	-0.74607	C	-2.96622	-1.59891	-0.24463	C	-2.51624	-2.27963	-0.23733
C	-0.42544	3.04647	0.69226	C	-2.66965	-1.13959	1.20134	C	-1.84966	-2.34905	1.15607
C	-1.37686	0.69432	-1.30361	C	-0.6383	-1.39214	-1.28587	C	-0.41589	-1.54582	-1.47396
C	0.10405	-1.14789	3.16348	C	1.75744	0.10808	2.89333	C	1.66323	0.17076	2.79568
C	1.21082	-1.83189	2.3468	C	2.00994	1.40292	2.10749	C	2.06157	1.45018	2.04906
C	0.7973	-2.9291	1.33549	C	2.93026	1.33505	0.86371	C	2.94012	1.3093	0.78549
C	0.09071	-2.42877	0.03741	C	2.33624	0.63819	-0.40103	C	2.25384	0.64195	-0.44278
C	-1.26567	2.23879	1.69738	C	-1.51852	-1.85289	1.93302	C	-2.03081	-1.10595	2.04123
C	-0.42424	1.27816	2.5284	C	-0.59354	-0.88455	2.65987	C	-0.83168	-0.27359	2.48737
C	-0.61936	-0.05403	2.43314	C	0.70221	-0.77797	2.29682	C	0.46584	-0.57081	2.26027
C	-1.15603	-0.55423	-2.14952	C	0.33987	-0.84726	-2.31996	C	0.32373	-0.67487	-2.48176
C	0.89516	-1.39537	-0.77829	C	1.01504	1.24372	-0.91503	C	0.8961	1.2607	-0.81523
C	0.28803	-1.06581	-2.17204	C	0.53312	0.67167	-2.27989	C	0.31593	0.82902	-2.18612
C	1.1575	-0.12176	-2.96511	C	-0.7141	1.36153	-2.77294	C	-1.06589	1.40146	-2.39537
C	1.57966	-0.39251	-4.21352	C	-0.81477	1.91044	-3.99675	C	-1.39064	2.15178	-3.46368
C	1.67283	1.11589	-2.35013	C	-1.85891	1.5584	-1.86583	C	-2.10722	1.23719	-1.35691
O	2.84351	1.45618	-2.48086	O	-2.40502	2.65087	-1.75834	O	-2.85401	2.15733	-1.04276
O	0.82635	1.82716	-1.58245	O	-2.2373	0.51501	-1.10518	O	-2.14771	0.05603	-0.70653
C	-0.9813	2.40703	-3.14667	C	-2.73483	-1.26345	-2.72817	C	-2.71211	-1.41797	-2.59144
C	-0.04575	-4.02303	2.01388	C	4.28988	0.71022	1.22188	C	4.26969	0.60394	1.09958
C	0.58701	1.93512	3.43389	C	-1.23574	-0.11032	3.78353	C	-1.26413	0.95107	3.26578
O	-2.38419	3.41487	-0.72153	O	-2.91962	-3.02226	-0.33615	O	-2.47073	-3.60637	-0.78893
O	2.01117	-3.58437	0.89217	O	3.24054	2.69624	0.47642	O	3.292	2.6498	0.35909
O	-0.12474	-3.56797	-0.814	O	3.29285	0.75148	-1.46794	O	3.12875	0.78982	-1.57279
H	-0.50717	4.05766	-1.17079	H	-4.00851	-1.30672	-0.43668	H	-3.57645	-2.03398	-0.08896
H	-0.373	4.06906	1.09306	H	-3.57585	-1.34291	1.78998	H	-0.81297	-2.68032	1.06483
H	0.61234	2.69806	0.65803	H	-2.55149	-0.05094	1.22265	H	-2.3274	-3.18575	1.68799
H	-1.0967	0.43061	-0.28675	H	-0.2363	-1.12462	-0.31184	H	0.10039	-1.44018	-0.51877
H	-2.45504	0.8969	-1.29198	H	-0.60808	-2.48688	-1.35126	H	-0.2812	-2.59057	-1.78188
H	-0.63195	-1.8769	3.51823	H	2.67856	-0.47262	3.00747	H	2.50709	-0.52545	2.85153
H	0.5547	-0.75389	4.08049	H	1.48371	0.38094	3.91802	H	1.46474	0.43185	3.84364
H	1.83995	-1.08053	1.8532	H	1.05583	1.87832	1.84668	H	1.17623	2.0565	1.82093
H	1.88949	-2.30194	3.07409	H	2.47305	2.11336	2.80863	H	2.6212	2.07108	2.76445
H	-0.89639	-2.02993	0.28349	H	2.20844	-0.42945	-0.2056	H	2.14005	-0.43069	-0.26113
H	-2.08018	1.70446	1.19798	H	-0.94793	-2.49198	1.25157	H	-2.50252	-1.45685	2.97128
H	-1.76587	2.9342	2.3847	H	-1.94105	-2.54188	2.67664	H	-2.77488	-0.43891	1.59114
H	-1.41539	-0.40843	1.78083	H	1.067	-1.42775	1.50319	H	0.72823	-1.46801	1.70843
H	-1.50735	-0.37574	-3.17365	H	0.03	-1.16024	-3.32502	H	-0.08047	-0.85532	-3.48628
H	-1.80843	-1.34695	-1.76199	H	1.31032	-1.33382	-2.15985	H	1.36253	-1.02473	-2.53107
H	1.90612	-1.78838	-0.95132	H	1.14832	2.32498	-1.05204	H	0.99778	2.35447	-0.84237
H	1.0174	-0.48328	-0.18586	H	0.24117	1.12253	-0.15063	H	0.18285	1.04657	-0.01618
H	0.25958	-2.01499	-2.7266	H	1.32957	0.89222	-3.00528	H	0.97437	1.27896	-2.94421
H	2.2435	0.28148	-4.74991	H	-1.70873	2.44441	-4.31053	H	-2.37935	2.59132	-3.57238

H	1.28303	-1.30082	-4.72807	H	-0.00241	1.84994	-4.71402	H	-0.67391	2.35546	-4.25265
H	-0.44024	3.32076	-3.41882	H	-3.77717	-0.92592	-2.76412	H	-3.77979	-1.24255	-2.41501
H	-0.70378	1.66317	-3.89748	H	-2.235	-0.77201	-3.56634	H	-2.41356	-0.70052	-3.35899
H	-2.0547	2.59729	-3.24927	H	-2.71401	-2.34202	-2.91562	H	-2.59238	-2.41759	-3.02185
H	-1.06224	-3.68051	2.2296	H	4.21654	-0.37109	1.37199	H	4.13218	-0.46531	1.28559
H	-0.11365	-4.92088	1.38903	H	5.0359	0.89455	0.44035	H	4.98442	0.71767	0.27652
H	0.41923	-4.34577	2.95263	H	4.69605	1.16228	2.13429	H	4.74874	1.05028	1.97864
H	1.28487	2.55084	2.85856	H	-0.55955	0.5964	4.26862	H	-0.43349	1.57089	3.61021
H	0.07581	2.58049	4.15592	H	-2.08723	0.47253	3.41967	H	-1.90069	1.58579	2.63977
H	1.1924	1.22479	4.00031	H	-1.59706	-0.80126	4.55239	H	-1.83611	0.65184	4.15048
H	-2.52116	4.24041	-0.22429	H	-3.5785	-3.37576	0.2872	H	-2.98786	-4.18564	-0.20226
H	2.46234	-3.94161	1.67767	H	2.42853	3.22839	0.55458	H	3.74946	3.08693	1.09939
H	0.73088	-4.04515	-0.80666	H	3.68257	1.64536	-1.37459	H	3.4442	1.7162	-1.51671
**1a-4**				**1a-5**				**1a-6**			
C	-2.10675	0.60761	-1.09052	C	-0.62184	1.97313	-1.70222	C	-1.88036	-1.20578	-1.34842
C	-2.16217	1.64815	0.08171	C	-0.98437	3.16467	-0.72977	C	-2.47465	-2.28975	-0.37015
C	-2.90773	1.27311	1.37324	C	-0.4276	3.0403	0.70676	C	-1.81298	-2.43095	1.02021
C	-1.59267	-0.75689	-0.60053	C	-1.37017	0.69681	-1.28345	C	-0.37531	-1.46189	-1.5468
C	1.78018	0.25964	2.74298	C	0.10731	-1.13702	3.19022	C	1.67073	0.0207	2.81902
C	2.63079	0.3725	1.46485	C	1.21646	-1.81803	2.37404	C	2.05525	1.34745	2.15303
C	2.99398	-0.92336	0.69302	C	0.8065	-2.91705	1.36326	C	2.93718	1.29713	0.88445
C	1.87692	-1.50135	-0.23379	C	0.10064	-2.41962	0.06367	C	2.27	0.70414	-0.39283
C	-2.1438	0.41618	2.40128	C	-1.27387	2.24139	1.71368	C	-2.00917	-1.24136	1.97303
C	-0.71962	0.8619	2.71076	C	-0.43273	1.2853	2.55204	C	-0.81936	-0.42605	2.47296
C	0.31555	0.0158	2.51603	C	-0.62028	-0.04799	2.45739	C	0.48177	-0.69646	2.23301
C	-0.95055	-1.69085	-1.6284	C	-1.14853	-0.55237	-2.12769	C	0.36119	-0.5332	-2.50395
C	1.38032	-0.53592	-1.33207	C	0.90397	-1.38643	-0.75375	C	0.90534	1.32128	-0.73728
C	0.3348	-1.15931	-2.30014	C	0.29624	-1.0618	-2.14848	C	0.33502	0.95289	-2.13158
C	-0.00656	-0.2228	-3.44064	C	1.16314	-0.11862	-2.94488	C	-1.05362	1.51713	-2.31208
C	0.5579	-0.31961	-4.65717	C	1.58651	-0.39391	-4.19195	C	-1.39351	2.31119	-3.34312
C	-1.06852	0.79781	-3.26976	C	1.67564	1.12287	-2.33592	C	-2.08386	1.29567	-1.27455
O	-1.73368	1.27865	-4.17259	O	2.84617	1.46324	-2.46709	O	-2.80607	2.20661	-0.88509
O	-1.12989	1.21234	-1.98896	O	0.8254	1.84431	-1.58218	O	-2.13699	0.07353	-0.70705
C	-3.47988	0.45722	-1.77055	C	-0.99258	2.40354	-3.1343	C	-2.66547	-1.30048	-2.67435
C	3.51865	-2.01298	1.64353	C	-0.0351	-4.01203	2.04176	C	4.26859	0.5806	1.16309
C	-0.6015	2.26344	3.25502	C	0.57034	1.95039	3.46048	C	-1.26635	0.74255	3.3259
O	-2.77087	2.86064	-0.40753	O	-2.38697	3.4235	-0.67551	O	-2.41141	-3.58208	-0.99621
O	4.09717	-0.60122	-0.18952	O	2.02194	-3.5707	0.92166	O	3.31019	2.66012	0.5599
O	2.40786	-2.65611	-0.90789	O	-0.11206	-3.56038	-0.7867	O	3.14521	0.93375	-1.50798
H	-1.13576	1.94301	0.32777	H	-0.5093	4.06799	-1.13902	H	-3.53818	-2.06461	-0.2134
H	-3.85486	0.77723	1.13015	H	-0.36228	4.06138	1.1098	H	-0.77281	-2.74699	0.91522
H	-3.20798	2.20202	1.87871	H	0.60621	2.68024	0.66969	H	-2.28477	-3.30066	1.50215
H	-0.8399	-0.58332	0.1642	H	-1.08303	0.43696	-0.26753	H	0.1326	-1.39735	-0.58361
H	-2.40943	-1.29912	-0.10816	H	-2.44917	0.89487	-1.2661	H	-0.22474	-2.48813	-1.90546
H	2.16634	-0.53068	3.39569	H	-0.62557	-1.86826	3.54678	H	2.52104	-0.66945	2.8351
H	1.93039	1.18098	3.31358	H	0.55712	-0.73936	4.10601	H	1.466	0.21489	3.88019
H	2.18844	1.11065	0.78569	H	1.84316	-1.06495	1.87997	H	1.16003	1.95201	1.96124
H	3.57861	0.83928	1.77241	H	1.89649	-2.28542	3.10176	H	2.60708	1.9335	2.90314
H	1.04269	-1.85675	0.37325	H	-0.88746	-2.02221	0.30787	H	2.16949	-0.37939	-0.28011
H	-2.15884	-0.63088	2.08043	H	-2.08528	1.7018	1.21489	H	-2.48308	-1.64844	2.87877
H	-2.71212	0.42601	3.34119	H	-1.77687	2.94087	2.39465	H	-2.75618	-0.55558	1.55741
H	0.08633	-0.99701	2.18937	H	-1.4125	-0.40598	1.80265	H	0.75518	-1.55655	1.62967
H	-1.69034	-1.96946	-2.38969	H	-1.4999	-0.3757	-3.15213	H	-0.03509	-0.66623	-3.51896
H	-0.70103	-2.62655	-1.11056	H	-1.79983	-1.34536	-1.73889	H	1.40411	-0.86884	-2.56422
H	2.23986	-0.22113	-1.9392	H	1.91557	-1.77827	-0.92566	H	0.98952	2.41601	-0.71018
H	0.98083	0.37289	-0.87405	H	1.02479	-0.47252	-0.16374	H	0.19377	1.05538	0.04771
H	0.80777	-2.04157	-2.7559	H	0.26922	-2.0126	-2.70033	H	0.98855	1.45027	-2.86375
H	0.27378	0.34063	-5.47328	H	2.24996	0.27856	-4.7308	H	-2.38825	2.74284	-3.42634
H	1.32008	-1.06229	-4.87024	H	1.29347	-1.3059	-4.70215	H	-0.68467	2.5576	-4.12715
H	-3.80763	1.39579	-2.23009	H	-0.48105	3.33539	-3.40189	H	-3.73676	-1.15057	-2.49632
H	-3.44727	-0.28421	-2.57639	H	-0.68615	1.67096	-3.88506	H	-2.37257	-0.53635	-3.39758

H	-4.24733	0.1325	-1.06138	H	-2.07098	2.55681	-3.24357	H	-2.5282	-2.27218	-3.15991
H	4.01558	-2.82007	1.09336	H	-1.05255	-3.67136	2.25592	H	4.13659	-0.49868	1.28364
H	4.27604	-1.604	2.32239	H	-0.10045	-4.91069	1.41781	H	4.98869	0.74571	0.3534
H	2.71469	-2.45195	2.24224	H	0.42947	-4.33285	2.98138	H	4.74024	0.97691	2.0698
H	-1.24836	2.37782	4.13153	H	1.26833	2.56724	2.88644	H	-0.44299	1.34746	3.71179
H	0.40926	2.52845	3.56933	H	0.05221	2.59603	4.17731	H	-1.90751	1.40925	2.73906
H	-0.90783	2.99852	2.50484	H	1.17578	1.24504	4.03301	H	-1.83778	0.38236	4.18796
H	-2.30883	3.08173	-1.23931	H	-2.63181	3.90442	-1.48498	H	-2.92156	-4.20089	-0.44477
H	4.84412	-0.30464	0.3606	H	2.47517	-3.92216	1.70863	H	2.52932	3.22823	0.68636
H	3.28278	-2.36849	-1.24085	H	0.74308	-4.03846	-0.77377	H	3.58161	1.7917	-1.32401
**1a-7**				**1a-8**				**1a-9**			
C	-1.93356	1.01993	-1.52164	C	-2.0751	0.76985	-1.40127	C	-2.41488	-0.51334	-0.98794
C	-2.93182	1.61978	-0.4537	C	-3.24253	0.97626	-0.36149	C	-3.31549	-0.56027	0.30738
C	-2.32403	1.88688	0.94234	C	-2.94668	0.62441	1.11395	C	-2.74535	0.16472	1.54751
C	-1.56091	-0.42474	-1.1478	C	-1.56783	-0.6811	-1.32576	C	-1.16433	-1.38948	-0.80542
C	1.1171	-0.83321	3.11063	C	1.01943	-0.81359	3.00808	C	2.0106	0.40486	2.70015
C	2.32161	-0.5355	2.20577	C	2.31464	-0.66242	2.20031	C	2.54041	1.27761	1.55309
C	2.70789	-1.5854	1.13477	C	2.61598	-1.71436	1.1092	C	3.25664	0.57562	0.37497
C	1.7597	-1.69667	-0.10075	C	1.67699	-1.68664	-0.13267	C	2.33901	-0.22951	-0.59944
C	-2.30939	0.70749	1.93122	C	-1.95478	1.55437	1.8298	C	-1.77668	-0.63666	2.43519
C	-0.97655	0.57321	2.65932	C	-0.62383	1.03018	2.36293	C	-0.53723	0.15988	2.82214
C	-0.21211	-0.52547	2.48448	C	-0.23165	-0.26187	2.37554	C	0.68611	-0.24669	2.42214
C	-0.61952	-1.17155	-2.08496	C	-0.45919	-1.0723	-2.29459	C	-0.1917	-1.46759	-1.97652
C	1.55942	-0.38522	-0.88627	C	1.52422	-0.29671	-0.77318	C	1.21133	0.60388	-1.24523
C	0.77346	-0.54916	-2.21971	C	0.8417	-0.27239	-2.16448	C	0.43829	-0.12955	-2.37837
C	0.68429	0.7457	-2.98727	C	0.64067	1.14453	-2.64651	C	-0.59471	0.75311	-3.03455
C	1.05797	0.86324	-4.27407	C	1.11469	1.58737	-3.82511	C	-0.63389	0.963	-4.36307
C	0.27632	1.98602	-2.30415	C	-0.01146	2.14608	-1.77421	C	-1.56223	1.51454	-2.22054
O	0.91972	3.02202	-2.42984	O	0.41202	3.2929	-1.68293	O	-1.83528	2.68254	-2.47354
O	-0.79156	1.92685	-1.48787	O	-1.05046	1.72786	-1.02235	O	-2.08682	0.89785	-1.14492
C	-2.61636	1.09219	-2.9003	C	-2.62941	1.11518	-2.80016	C	-3.27857	-0.99605	-2.16968
C	2.9087	-2.97068	1.77354	C	2.67827	-3.13378	1.69679	C	4.41494	-0.29952	0.88383
C	-0.62607	1.72873	3.56264	C	0.22814	2.14761	2.9266	C	-0.8005	1.38051	3.66831
O	-4.11998	0.84178	-0.31401	O	-4.38536	0.18621	-0.7295	O	-3.69225	-1.89659	0.63927
O	4.01305	-1.22398	0.61981	O	3.94569	-1.43486	0.60264	O	3.88171	1.6252	-0.39492
O	2.32392	-2.6508	-1.01629	O	2.22891	-2.56797	-1.12438	O	3.15677	-0.72513	-1.67695
H	-3.23948	2.60905	-0.82223	H	-3.55655	2.02886	-0.38517	H	-4.24359	-0.02619	0.05826
H	-2.94064	2.66726	1.41143	H	-2.71031	-0.43711	1.21409	H	-3.60296	0.41894	2.18703
H	-1.33076	2.33486	0.83042	H	-3.905	0.71253	1.6485	H	-2.31822	1.12664	1.24475
H	-1.08476	-0.42362	-0.17044	H	-1.19994	-0.88459	-0.31923	H	-0.59527	-1.01941	0.04416
H	-2.47472	-1.02473	-1.0543	H	-2.40703	-1.36878	-1.49125	H	-1.46703	-2.41507	-0.56019
H	1.11618	-1.87758	3.43922	H	0.84396	-1.86306	3.26837	H	2.73209	-0.37359	2.96951
H	1.2384	-0.26062	4.03623	H	1.1598	-0.3193	3.97854	H	1.94204	1.02882	3.59747
H	2.21081	0.4535	1.74316	H	2.39027	0.34621	1.77557	H	1.74537	1.93336	1.17613
H	3.19316	-0.41883	2.86725	H	3.14303	-0.69989	2.9234	H	3.26441	1.97708	1.99772
H	0.7927	-2.09712	0.21364	H	0.69516	-2.08361	0.14106	H	1.91674	-1.09838	-0.08751
H	-2.57183	-0.2329	1.43607	H	-2.48386	1.95148	2.70895	H	-1.49435	-1.58326	1.96332
H	-3.09541	0.85973	2.68289	H	-1.76037	2.43395	1.20537	H	-2.29581	-0.92652	3.3586
H	-0.59656	-1.31941	1.84674	H	-0.88599	-1.03017	1.97523	H	0.75623	-1.17338	1.85681
H	-1.08355	-1.27313	-3.07416	H	-0.83283	-1.00479	-3.32454	H	-0.68878	-1.92339	-2.84212
H	-0.51566	-2.1994	-1.715	H	-0.24116	-2.1372	-2.14498	H	0.607	-2.17068	-1.70818
H	2.54301	0.02872	-1.14489	H	2.52033	0.14981	-0.89879	H	1.64675	1.51025	-1.68704
H	1.07427	0.35297	-0.23999	H	0.99057	0.35386	-0.07665	H	0.52123	0.94282	-0.46627
H	1.35598	-1.24815	-2.83718	H	1.55098	-0.7545	-2.85381	H	1.18405	-0.37275	-3.14916
H	1.0306	1.81971	-4.79104	H	0.99918	2.62383	-4.13337	H	-1.35826	1.63826	-4.81282
H	1.41385	0.00922	-4.84168	H	1.64231	0.92896	-4.50769	H	0.06586	0.47781	-5.03579
H	-2.9119	2.12224	-3.13102	H	-3.03609	2.13316	-2.81013	H	-4.16856	-0.36567	-2.28033
H	-1.95375	0.78487	-3.71304	H	-1.86401	1.09124	-3.57898	H	-2.75308	-0.93803	-3.12603
H	-3.50785	0.45833	-2.9432	H	-3.42023	0.4228	-3.10651	H	-3.60391	-2.03254	-2.03266
H	1.95783	-3.43398	2.05345	H	1.68736	-3.50732	1.97132	H	5.10654	-0.56149	0.07507
H	3.43283	-3.65431	1.09587	H	3.12372	-3.83929	0.98613	H	5.0161	0.24316	1.62276

H	3.53782	-2.8975	2.66834	H	3.31737	-3.15865	2.58695	H	4.05595	-1.22648	1.341
H	-0.59234	2.66717	3.00102	H	0.44875	2.88053	2.14295	H	-1.27259	1.08446	4.611
H	-1.38059	1.8276	4.35006	H	-0.30595	2.65611	3.73628	H	0.10162	1.94301	3.91649
H	0.34509	1.62242	4.05001	H	1.18577	1.81158	3.33008	H	-1.47022	2.07606	3.1538
H	-4.69668	1.04044	-1.07198	H	-4.90569	0.70306	-1.36911	H	-4.35761	-1.84205	1.34792
H	4.041	-0.25639	0.51177	H	4.56361	-1.47074	1.35455	H	4.07961	1.23656	-1.27052
H	3.29165	-2.50186	-0.9833	H	3.18971	-2.37378	-1.1144	H	3.64275	-1.50129	-1.34603
**1a-10**				**1a-11**				**1a-12**			
C	-2.08814	0.60258	-1.07056	C	-1.93226	-1.2636	-1.27422	C	-1.87366	-1.21366	-1.34126
C	-2.144	1.64393	0.10086	C	-2.52909	-2.28679	-0.23309	C	-2.46882	-2.29987	-0.36507
C	-2.89008	1.26968	1.39226	C	-1.86586	-2.36152	1.16184	C	-1.80058	-2.44769	1.02056
C	-1.57719	-0.76222	-0.57903	C	-0.43381	-1.54931	-1.48058	C	-0.36647	-1.46263	-1.53049
C	1.79558	0.23725	2.76332	C	1.63439	0.17482	2.80866	C	1.67085	0.00517	2.84344
C	2.64224	0.36277	1.48442	C	2.03643	1.45104	2.05852	C	2.05289	1.33532	2.18288
C	3.00831	-0.92455	0.69914	C	2.91613	1.3041	0.79642	C	2.93665	1.29166	0.91545
C	1.89663	-1.50911	-0.23157	C	2.22766	0.63622	-0.43021	C	2.27251	0.70191	-0.36479
C	-2.12773	0.41084	2.41998	C	-2.05273	-1.12289	2.05149	C	-2.00188	-1.26625	1.98215
C	-0.7013	0.85093	2.72705	C	-0.85701	-0.28196	2.49247	C	-0.81665	-0.44651	2.48644
C	0.3302	0.00022	2.53278	C	0.44256	-0.57346	2.26915	C	0.48604	-0.7139	2.25186
C	-0.94262	-1.70051	-1.60716	C	0.30282	-0.66784	-2.48187	C	0.37103	-0.53151	-2.48427
C	1.39369	-0.54814	-1.32972	C	0.8729	1.26012	-0.80461	C	0.90695	1.31731	-0.70953
C	0.33936	-1.17373	-2.28791	C	0.29292	0.83429	-2.17725	C	0.3421	0.95331	-2.10718
C	-0.00725	-0.24021	-3.42938	C	-1.09011	1.40491	-2.38329	C	-1.04631	1.51644	-2.29149
C	0.53832	-0.35354	-4.65316	C	-1.41695	2.16062	-3.44707	C	-1.38153	2.31489	-3.32076
C	-1.05146	0.79785	-3.25055	C	-2.13106	1.23084	-1.34547	C	-2.08192	1.29009	-1.26064
O	-1.7037	1.3018	-4.15002	O	-2.88614	2.14327	-1.0301	O	-2.80834	2.19856	-0.8735
O	-1.10649	1.20394	-1.96667	O	-2.15267	0.0497	-0.69353	O	-2.14396	0.06402	-0.70269
C	-3.46001	0.4545	-1.75364	C	-2.73643	-1.41513	-2.58304	C	-2.64721	-1.30885	-2.67354
C	3.53778	-2.01482	1.64573	C	4.24257	0.59441	1.11343	C	4.26937	0.57726	1.19326
C	-0.57679	2.25186	3.27173	C	-1.29708	0.94407	3.26494	C	-1.27045	0.71956	3.33894
O	-2.75138	2.85656	-0.38901	O	-2.52463	-3.6172	-0.77908	O	-2.40587	-3.60218	-0.96936
O	4.12934	-0.60563	-0.1616	O	3.27349	2.64145	0.36599	O	3.30688	2.65662	0.59558
O	2.42885	-2.65963	-0.90985	O	3.10321	0.77419	-1.5605	O	3.14854	0.93817	-1.47818
H	-1.11757	1.93829	0.34739	H	-3.58497	-2.03038	-0.07505	H	-3.53	-2.07587	-0.18939
H	-3.83821	0.77577	1.14892	H	-0.82693	-2.68651	1.07371	H	-0.75905	-2.75785	0.91178
H	-3.18855	2.19895	1.89813	H	-2.34458	-3.20379	1.68418	H	-2.26791	-3.32454	1.49451
H	-0.82028	-0.58914	0.18174	H	0.0866	-1.45692	-0.52595	H	0.13609	-1.39435	-0.56488
H	-2.39395	-1.30063	-0.0826	H	-0.30068	-2.58916	-1.80402	H	-0.20987	-2.48875	-1.88703
H	2.17986	-0.56092	3.40731	H	2.47944	-0.51914	2.87453	H	2.52344	-0.68215	2.85975
H	1.94811	1.15168	3.34436	H	1.42831	0.4412	3.85382	H	1.46234	0.19526	3.90462
H	2.19357	1.10461	0.81313	H	1.15245	2.05829	1.82769	H	1.1564	1.9382	1.9918
H	3.59185	0.82757	1.79023	H	2.59652	2.07298	2.77264	H	2.60209	1.92011	2.93589
H	1.06352	-1.86768	0.37549	H	2.10828	-0.43511	-0.24485	H	2.17453	-0.3823	-0.25652
H	-2.14722	-0.63646	2.10021	H	-2.51626	-1.47962	2.98335	H	-2.47354	-1.68137	2.88534
H	-2.69464	0.42369	3.3607	H	-2.80409	-0.4607	1.60606	H	-2.75184	-0.58111	1.57054
H	0.09773	-1.01238	2.20719	H	0.71119	-1.47208	1.72348	H	0.7628	-1.57519	1.65147
H	-1.68717	-1.98013	-2.36342	H	-0.10242	-0.84201	-3.48716	H	-0.02267	-0.66239	-3.50055
H	-0.69203	-2.63507	-1.08779	H	1.34211	-1.01561	-2.53534	H	1.41451	-0.86571	-2.54273
H	2.24825	-0.24052	-1.94709	H	0.97807	2.35363	-0.82916	H	0.98841	2.41211	-0.67731
H	1.00153	0.36411	-0.87235	H	0.15817	1.04704	-0.00665	H	0.19349	1.04612	0.07202
H	0.80789	-2.05862	-2.74319	H	0.95038	1.28948	-2.93309	H	0.99785	1.45368	-2.83532
H	0.25113	0.30407	-5.47051	H	-2.40703	2.59807	-3.55287	H	-2.37567	2.7474	-3.40682
H	1.285	-1.10995	-4.87327	H	-0.70119	2.37171	-4.23496	H	-0.66847	2.56628	-4.09944
H	-3.78568	1.3938	-2.21312	H	-3.8022	-1.23421	-2.40084	H	-3.7229	-1.18674	-2.50107
H	-3.42685	-0.28619	-2.56012	H	-2.4375	-0.69846	-3.35122	H	-2.36806	-0.52815	-3.38441
H	-4.22925	0.13036	-1.04612	H	-2.62412	-2.41492	-3.01508	H	-2.48275	-2.26991	-3.17149
H	4.03702	-2.82025	1.09512	H	4.10065	-0.47375	1.30247	H	4.13971	-0.50275	1.30985
H	4.29558	-1.60439	2.3234	H	4.9583	0.70284	0.29051	H	4.99015	0.74685	0.3851
H	2.73658	-2.45841	2.24478	H	4.72277	1.04145	1.9915	H	4.73891	0.97147	2.10197
H	-1.21282	2.36489	4.15626	H	-0.47043	1.5718	3.60455	H	-0.45023	1.32433	3.7317
H	0.43794	2.51723	3.57269	H	-1.93877	1.57098	2.63629	H	-1.90851	1.38685	2.74941

H	-0.89247	2.98795	2.52643	H	-1.86586	0.64566	4.15198	H	-1.84706	0.35689	4.19649
H	-2.29388	3.07572	-1.22364	H	-1.63796	-3.99748	-0.65826	H	-3.20408	-3.71187	-1.51513
H	3.97911	0.27695	-0.54449	H	3.72765	3.08153	1.10668	H	2.52648	3.22352	0.73007
H	3.35377	-2.42756	-1.13318	H	3.42487	1.69885	-1.50791	H	3.58523	1.79471	-1.28804
**1a-13**				**1a-14**				**1a-15**			
C	-1.25819	1.73287	-1.63467	C	-2.18722	0.80991	-1.35277	C	-0.96172	1.84521	-1.59413
C	-2.0154	2.68634	-0.62696	C	-1.86357	1.44006	0.03797	C	-1.78049	2.82516	-0.66848
C	-1.48393	2.70592	0.82557	C	-2.79589	1.09847	1.2127	C	-1.88472	2.47441	0.83405
C	-1.54529	0.25677	-1.31119	C	-2.31802	-0.72713	-1.32583	C	-1.53617	0.4217	-1.47552
C	0.50601	-1.1519	3.12066	C	1.69666	-0.05573	2.85569	C	0.04095	-1.07485	3.09151
C	1.77225	-1.39405	2.28566	C	2.52621	0.44327	1.65915	C	1.16875	-1.85712	2.40752
C	1.74091	-2.52122	1.22448	C	3.12151	-0.6155	0.69607	C	0.7828	-2.91794	1.35138
C	0.90458	-2.2255	-0.0591	C	2.0877	-1.36289	-0.20088	C	0.18307	-2.37323	0.02052
C	-1.96896	1.60168	1.78059	C	-2.18606	0.12716	2.24339	C	-0.57548	2.57825	1.6313
C	-0.83701	0.96226	2.57469	C	-0.79985	0.51776	2.73858	C	0.0489	1.34762	2.28522
C	-0.55504	-0.34927	2.42441	C	0.25122	-0.30303	2.53018	C	-0.49796	0.11387	2.33717
C	-0.88908	-0.79368	-2.20116	C	-1.03369	-1.50868	-1.03827	C	-0.88671	-0.65757	-2.33306
C	1.3122	-0.94224	-0.81165	C	1.32127	-0.46862	-1.20437	C	1.01796	-1.26572	-0.64115
C	0.64328	-0.77349	-2.20517	C	0.19118	-1.24095	-1.94508	C	0.60826	-0.88831	-2.08834
C	1.14259	0.45031	-2.9328	C	-0.1606	-0.63235	-3.28843	C	1.41078	0.28944	-2.58635
C	1.64996	0.40319	-4.1779	C	0.15645	-1.23552	-4.44877	C	2.1298	0.2588	-3.72258
C	1.18604	1.75897	-2.25203	C	-0.89716	0.65813	-3.38514	C	1.51941	1.51268	-1.76177
O	2.15561	2.50227	-2.35009	O	-1.24085	1.19009	-4.42903	O	2.5917	2.08419	-1.60114
O	0.14837	2.06951	-1.45199	O	-1.06059	1.23642	-2.17703	O	0.4079	1.91573	-1.1126
C	-1.72163	2.10249	-3.05717	C	-3.46967	1.42144	-1.94977	C	-1.0508	2.38902	-3.03571
C	1.30905	-3.86085	1.84551	C	3.99713	-1.62191	1.46485	C	-0.14906	-3.98371	1.9508
C	-0.11534	1.89316	3.51728	C	-0.72106	1.86238	3.41575	C	1.38084	1.67759	2.92615
O	-3.43337	2.52582	-0.65801	O	-1.85691	2.87649	-0.09258	O	-3.1212	2.97928	-1.16483
O	3.10105	-2.71833	0.76621	O	4.01192	0.07434	-0.21057	O	1.98927	-3.63801	0.9948
O	1.08166	-3.32754	-0.9656	O	2.79715	-2.33487	-0.99292	O	0.08493	-3.46098	-0.91133
H	-1.83449	3.70717	-0.99285	H	-0.83108	1.19571	0.28989	H	-1.31862	3.81894	-0.73677
H	-1.81852	3.6552	1.26855	H	-3.73828	0.67074	0.85367	H	-2.40706	1.52482	0.96916
H	-0.39046	2.76709	0.80602	H	-3.08071	2.01843	1.74043	H	-2.58269	3.20443	1.27148
H	-1.21872	0.04743	-0.29512	H	-3.08274	-1.02666	-0.59928	H	-1.47055	0.09089	-0.43787
H	-2.62654	0.07661	-1.34025	H	-2.70266	-1.07645	-2.2942	H	-2.60401	0.43645	-1.72687
H	0.06615	-2.09898	3.45054	H	2.12155	-0.96949	3.28297	H	-0.79541	-1.73575	3.34332
H	0.80455	-0.65687	4.05058	H	1.78111	0.68486	3.6574	H	0.40644	-0.72638	4.06654
H	2.11299	-0.45555	1.83062	H	1.96496	1.19708	1.09308	H	1.91228	-1.16723	1.98932
H	2.57258	-1.64295	2.9984	H	3.37193	1.00935	2.0773	H	1.72079	-2.3821	3.2014
H	-0.15883	-2.19379	0.1899	H	1.3967	-1.92971	0.42616	H	-0.83382	-2.01287	0.20158
H	-2.53777	0.83292	1.24994	H	-2.17032	-0.87908	1.80884	H	-0.77281	3.27749	2.45751
H	-2.68118	2.03582	2.49505	H	-2.85938	0.06291	3.10772	H	0.18679	3.07868	1.02314
H	-1.18023	-0.93393	1.75283	H	0.05891	-1.27275	2.0773	H	-1.46451	-0.07155	1.88
H	-1.26701	-0.69683	-3.22689	H	-1.28684	-2.57456	-1.12667	H	-1.05517	-0.42992	-3.39369
H	-1.23072	-1.78173	-1.8674	H	-0.76429	-1.37047	0.00863	H	-1.42713	-1.59655	-2.15925
H	2.39834	-0.95559	-0.97456	H	2.03145	-0.09262	-1.95282	H	2.06796	-1.58505	-0.68414
H	1.1073	-0.07363	-0.1782	H	0.90744	0.4019	-0.69478	H	0.99912	-0.38373	0.00298
H	0.95092	-1.64547	-2.80048	H	0.60525	-2.2375	-2.15779	H	0.87679	-1.75475	-2.71109
H	2.04579	1.29067	-4.66642	H	-0.10087	-0.80027	-5.41171	H	2.73643	1.10541	-4.03601
H	1.69532	-0.52533	-4.73788	H	0.69091	-2.18016	-4.4707	H	2.13871	-0.61539	-4.36566
H	-1.50928	3.15644	-3.27105	H	-3.36873	2.50165	-2.10053	H	-0.66349	3.41348	-3.08311
H	-1.20267	1.53173	-3.83101	H	-3.68101	1.00081	-2.93963	H	-0.45611	1.81062	-3.74607
H	-2.7957	1.9329	-3.18593	H	-4.34225	1.23569	-1.31656	H	-2.08124	2.39115	-3.4059
H	0.2378	-3.88175	2.06715	H	4.64152	-2.19234	0.78618	H	-1.15521	-3.59426	2.13198
H	1.53639	-4.70176	1.18043	H	4.67457	-1.10218	2.15236	H	-0.23642	-4.85501	1.29154
H	1.86015	-4.05468	2.77305	H	3.39571	-2.33127	2.04107	H	0.25314	-4.36262	2.89753
H	0.32546	2.73479	2.97478	H	-1.46409	1.91994	4.21834	H	2.0788	2.04745	2.16717
H	-0.81806	2.29221	4.2563	H	0.25108	2.06697	3.86803	H	1.25043	2.45569	3.68573
H	0.69909	1.4152	4.065	H	-0.92067	2.66705	2.70192	H	1.85954	0.8251	3.41279
H	-3.66979	1.66119	-0.287	H	-1.30667	3.07389	-0.87531	H	-3.65658	2.23213	-0.84763
H	3.64944	-2.93804	1.54042	H	4.69448	0.52102	0.32131	H	2.71921	-2.9968	0.92647

H	2.0474	-3.49396	-0.96408	H	3.54573	-1.84121	-1.38552	H	0.85339	-4.03828	-0.71903
**1a-16**				**1b-1**				**1b-2**			
C	-1.18797	1.7744	-1.61672	C	-1.40211	2.14984	0.22126	C	-2.08672	1.64463	0.11269
C	-1.94199	2.72771	-0.60639	C	-1.40084	1.23891	1.49806	C	-1.59109	0.79526	1.32972
C	-1.43714	2.70786	0.85576	C	-2.67327	0.41147	1.77769	C	-2.48656	-0.41763	1.66135
C	-1.50677	0.29986	-1.31508	C	-0.05323	2.92402	0.11505	C	-1.06393	2.76985	-0.22414
C	0.41122	-1.24071	3.12205	C	-0.00292	-3.15187	-0.37938	C	0.91638	-2.97797	0.08929
C	1.69334	-1.4858	2.31359	C	1.24049	-2.48714	-0.99937	C	1.61878	-2.05539	-0.92923
C	1.67256	-2.58845	1.22623	C	2.16612	-1.70467	-0.02806	C	2.4553	-0.88406	-0.34815
C	0.87368	-2.26304	-0.07509	C	3.03584	-0.62847	-0.75901	C	2.79034	0.21561	-1.4093
C	-1.9659	1.59816	1.78112	C	-2.91835	-0.81102	0.8815	C	-1.80451	-1.45948	2.57114
C	-0.86469	0.91617	2.58313	C	-1.82555	-1.87134	0.92818	C	-0.58154	-2.21292	2.04714
C	-0.61352	-0.39969	2.416	C	-1.13414	-2.17182	-0.18992	C	-0.28795	-2.33273	0.73473
C	-0.86649	-0.75374	-2.21275	C	1.19149	2.06116	-0.16514	C	0.32197	2.29829	-0.71337
C	1.31594	-0.97777	-0.80146	C	2.32852	0.34661	-1.72982	C	1.66123	0.7556	-2.31624
C	0.66538	-0.76994	-2.1994	C	1.00141	1.00194	-1.26715	C	0.28762	1.10577	-1.68761
C	1.19946	0.45814	-2.89308	C	0.17792	1.60084	-2.39657	C	-0.81035	1.34925	-2.71544
C	1.71168	0.43524	-4.1365	C	0.64875	2.02362	-3.58283	C	-0.63048	1.71876	-3.99606
C	1.27879	1.74027	-2.16923	C	-1.29752	1.72166	-2.16624	C	-2.22565	1.15881	-2.26365
O	2.29484	2.42527	-2.19388	O	-2.10621	2.03694	-3.02596	O	-3.20496	1.26719	-2.98534
O	0.22208	2.08613	-1.41059	O	-1.66195	1.30393	-0.93063	O	-2.29476	0.71365	-0.9887
C	-1.62262	2.17113	-3.04046	C	-2.54096	3.1857	0.28261	C	-3.44831	2.29466	0.41371
C	1.201	-3.9297	1.81389	C	3.08556	-2.69078	0.71678	C	3.76773	-1.4249	0.25223
C	-0.1381	1.81122	3.55635	C	-1.63802	-2.5448	2.26061	C	0.24621	-2.82107	3.15248
O	-3.36221	2.60121	-0.66682	O	-1.16423	2.0588	2.65543	O	-1.45948	1.62167	2.49013
O	3.0422	-2.82544	0.81813	O	1.43778	-1.00803	0.99324	O	1.78021	-0.21195	0.72316
O	1.05054	-3.35116	-0.9968	O	3.73185	0.15408	0.22256	O	3.3887	1.33828	-0.74211
H	-1.73041	3.75027	-0.95034	H	-0.55685	0.54834	1.43445	H	-0.59367	0.42821	1.09093
H	-1.75691	3.65672	1.31037	H	-3.5548	1.06171	1.73938	H	-2.83813	-0.91775	0.7532
H	-0.3423	2.74242	0.85685	H	-2.64422	0.07105	2.82197	H	-3.37995	-0.06325	2.19127
H	-1.1917	0.07138	-0.29959	H	0.12448	3.50903	1.02631	H	-0.92238	3.42462	0.64524
H	-2.59122	0.14234	-1.35439	H	-0.12697	3.67129	-0.6873	H	-1.48718	3.41769	-1.00432
H	-0.05611	-2.18581	3.41757	H	-0.36119	-3.91958	-1.07684	H	0.55545	-3.86265	-0.45037
H	0.69373	-0.77323	4.07112	H	0.26727	-3.67607	0.53973	H	1.64385	-3.33975	0.81804
H	2.05668	-0.54164	1.8883	H	1.8386	-3.26162	-1.49826	H	2.27611	-2.67104	-1.55786
H	2.47458	-1.7649	3.03646	H	0.89273	-1.84095	-1.8114	H	0.84687	-1.6829	-1.60913
H	-0.19436	-2.21536	0.15134	H	3.81062	-1.14273	-1.34144	H	3.56229	-0.17895	-2.08229
H	-2.54329	0.85337	1.22605	H	-3.10903	-0.48508	-0.14622	H	-2.55505	-2.21041	2.85112
H	-2.68029	2.03642	2.49087	H	-3.85575	-1.28574	1.20165	H	-1.5232	-0.95192	3.50287
H	-1.24026	-0.95834	1.72377	H	-1.40598	-1.64046	-1.10465	H	-0.98246	-1.91291	0.00718
H	-1.23042	-0.63319	-3.24104	H	2.01666	2.73373	-0.43323	H	0.82826	3.15098	-1.18436
H	-1.2352	-1.73795	-1.89705	H	1.48893	1.57773	0.76976	H	0.91797	2.04743	0.16751
H	2.40226	-1.0125	-0.95744	H	3.03924	1.14182	-1.99325	H	2.04576	1.64696	-2.83054
H	1.12448	-0.116	-0.15456	H	2.13705	-0.21088	-2.65625	H	1.49315	-0.00232	-3.0937
H	0.96133	-1.63611	-2.80884	H	0.39843	0.19071	-0.85651	H	-0.00608	0.21761	-1.12477
H	2.13408	1.3262	-4.59565	H	-0.01226	2.43723	-4.34156	H	-1.47307	1.8709	-4.66757
H	1.73293	-0.47638	-4.72518	H	1.70248	1.98452	-3.83478	H	0.35216	1.89389	-4.41868
H	-1.39041	3.2245	-3.23575	H	-2.48603	3.80351	1.18429	H	-3.4009	2.96174	1.28013
H	-1.10008	1.60346	-3.81402	H	-2.50596	3.85422	-0.58572	H	-3.79802	2.87921	-0.44517
H	-2.69698	2.0204	-3.18872	H	-3.52296	2.70341	0.24276	H	-4.21879	1.53774	0.59508
H	0.12458	-3.93462	2.00999	H	2.50105	-3.42701	1.27939	H	4.38009	-1.92363	-0.50603
H	1.42737	-4.7653	1.14169	H	3.74208	-3.22883	0.02527	H	4.36121	-0.62019	0.70082
H	1.7272	-4.14908	2.75025	H	3.70958	-2.1742	1.45476	H	3.57277	-2.13684	1.06127
H	0.32835	2.65475	3.03882	H	-1.18101	-1.85439	2.97627	H	0.72971	-2.03126	3.73667
H	-0.84339	2.20913	4.2935	H	-2.60522	-2.86794	2.66014	H	-0.39497	-3.40349	3.82321
H	0.65825	1.30436	4.10477	H	-1.00681	-3.4345	2.20769	H	1.02392	-3.49882	2.79567
H	-3.62746	1.73842	-0.31135	H	-0.988	1.46214	3.40338	H	-0.88183	1.15381	3.11703
H	3.49739	-1.96629	0.75945	H	0.90878	-1.66037	1.48443	H	1.65966	-0.85062	1.44844
H	1.98228	-3.63476	-0.88927	H	3.1186	0.21035	0.98292	H	2.94828	1.37873	0.1307
**1b-3**				**1b-4**				**1b-5**			
C	-1.4176	2.24365	0.04689	C	-1.38857	2.13895	0.20492	C	-1.40693	2.23239	-0.06161

C	-1.5927	1.08585	1.08522	C	-1.4228	1.23281	1.48603	C	-1.6066	1.13235	1.03457
C	-2.87705	0.25256	0.89478	C	-2.69629	0.39652	1.73544	C	-2.86412	0.25585	0.84532
C	-0.04367	2.95318	0.23063	C	-0.03131	2.89956	0.12064	C	-0.03663	2.94918	0.1185
C	-0.13409	-3.10834	-0.42816	C	0.02705	-3.18267	-0.34414	C	-0.07249	-3.1327	-0.29691
C	1.08448	-2.35587	-1.0029	C	1.27517	-2.52132	-0.95744	C	1.14887	-2.40042	-0.89107
C	2.02667	-1.66458	0.01928	C	2.18582	-1.72497	0.0159	C	2.07347	-1.65883	0.11074
C	2.98674	-0.62183	-0.64403	C	3.06171	-0.65657	-0.71823	C	3.0338	-0.63968	-0.58627
C	-2.8484	-1.08171	1.66836	C	-2.91144	-0.8334	0.84157	C	-2.82522	-1.04022	1.68072
C	-1.82916	-2.14929	1.26351	C	-1.81535	-1.88733	0.91812	C	-1.79426	-2.11472	1.33198
C	-1.21803	-2.17702	0.05998	C	-1.1117	-2.20586	-0.18719	C	-1.16691	-2.1873	0.13875
C	1.20429	2.08685	-0.04187	C	1.20994	2.02798	-0.14614	C	1.21928	2.07972	-0.10401
C	2.40219	0.41695	-1.62856	C	2.36007	0.31169	-1.70026	C	2.44989	0.35885	-1.61223
C	1.08609	1.15061	-1.2603	C	1.02901	0.97095	-1.25247	C	1.12349	1.09495	-1.28533
C	0.47553	1.93246	-2.41686	C	0.22226	1.5743	-2.39213	C	0.52151	1.82553	-2.47988
C	1.1289	2.38349	-3.50243	C	0.70854	1.99017	-3.57463	C	1.18556	2.24385	-3.5721
C	-0.991	2.23054	-2.3446	C	-1.25385	1.70957	-2.17771	C	-0.9484	2.10742	-2.44019
O	-1.62163	2.82949	-3.20165	O	-2.05042	2.04521	-3.04097	O	-1.57649	2.658	-3.33114
O	-1.57213	1.64595	-1.27266	O	-1.63672	1.29212	-0.94743	O	-1.53787	1.56692	-1.34968
C	-2.53323	3.29123	0.20082	C	-2.5176	3.18777	0.23709	C	-2.52363	3.28918	0.00775
C	2.86301	-2.72491	0.76143	C	3.09626	-2.69841	0.78732	C	2.90557	-2.67802	0.91203
C	-1.58984	-3.16546	2.35275	C	-1.63479	-2.53133	2.26632	C	-1.56042	-3.08342	2.46499
O	-1.57043	1.57658	2.4291	O	-1.20131	2.03571	2.65933	O	-1.61158	1.69854	2.34969
O	1.30886	-0.94965	1.03407	O	1.44346	-1.01257	1.01322	O	1.33794	-0.90155	1.07698
O	3.67583	0.09997	0.38992	O	3.75236	0.13449	0.26049	O	3.71451	0.12359	0.42287
H	-0.74593	0.40879	0.98387	H	-0.57964	0.54003	1.44304	H	-0.7396	0.47412	0.99786
H	-3.07561	0.05068	-0.16256	H	-3.58135	1.03975	1.66829	H	-3.02634	0.00132	-0.20665
H	-3.73287	0.82611	1.27303	H	-2.6893	0.06535	2.78325	H	-3.74656	0.82266	1.16834
H	0.03522	3.3725	1.24156	H	0.13813	3.47915	1.03697	H	0.02399	3.40058	1.11712
H	0.00432	3.82149	-0.44121	H	-0.08672	3.65054	-0.67979	H	0.02015	3.79426	-0.5817
H	-0.56876	-3.71288	-1.23419	H	-0.31743	-3.96363	-1.03381	H	-0.49541	-3.77399	-1.0805
H	0.19682	-3.81184	0.3378	H	0.28677	-3.6905	0.58704	H	0.25351	-3.80067	0.50221
H	1.679	-3.06376	-1.596	H	1.88187	-3.2996	-1.43976	H	1.75436	-3.1309	-1.44428
H	0.70064	-1.63262	-1.72853	H	0.93532	-1.88539	-1.78091	H	0.77026	-1.71235	-1.6529
H	3.76176	-1.16829	-1.19639	H	3.83963	-1.17634	-1.29147	H	3.81393	-1.204	-1.11286
H	-3.84455	-1.5381	1.59958	H	-3.08085	-0.51422	-0.19202	H	-3.81593	-1.51026	1.62582
H	-2.69406	-0.84397	2.72926	H	-3.85287	-1.31211	1.14355	H	-2.68163	-0.75191	2.7305
H	-1.50493	-1.43437	-0.68399	H	-1.37578	-1.69348	-1.11463	H	-1.44339	-1.47534	-0.63794
H	2.06552	2.75593	-0.16805	H	2.04325	2.69524	-0.40221	H	2.0785	2.74843	-0.24523
H	1.40833	1.50645	0.86104	H	1.49158	1.54233	0.79227	H	1.41466	1.53767	0.82413
H	3.17928	1.17051	-1.81617	H	3.07272	1.10517	-1.96375	H	3.22288	1.11144	-1.81963
H	2.24199	-0.1068	-2.58094	H	2.17503	-0.25173	-2.62441	H	2.30341	-0.20051	-2.54646
H	0.37547	0.35953	-1.01284	H	0.4172	0.16127	-0.85144	H	0.41575	0.30869	-1.01444
H	0.61487	2.93533	-4.28694	H	0.05919	2.40743	-4.34133	H	0.67727	2.75958	-4.38424
H	2.19236	2.2318	-3.6472	H	1.76432	1.94072	-3.81611	H	2.25299	2.1004	-3.69449
H	-2.53675	3.74047	1.19888	H	-2.45853	3.83093	1.12029	H	-2.52828	3.82026	0.96455
H	-2.4142	4.09659	-0.53327	H	-2.47439	3.83292	-0.64845	H	-2.405	4.03303	-0.7887
H	-3.51881	2.85402	0.00879	H	-3.50424	2.71452	0.20841	H	-3.50864	2.83651	-0.14787
H	2.22382	-3.42153	1.3142	H	2.50526	-3.42785	1.35196	H	2.26296	-3.3535	1.48661
H	3.4849	-3.30178	0.06924	H	3.76343	-3.24474	0.11268	H	3.54132	-3.28106	0.25569
H	3.51688	-2.2637	1.51024	H	3.70887	-2.17054	1.52684	H	3.5452	-2.17876	1.64862
H	-1.0713	-2.69715	3.19573	H	-1.18303	-1.82512	2.96975	H	-1.05447	-2.57698	3.29345
H	-2.54607	-3.5613	2.71211	H	-2.60404	-2.84715	2.66679	H	-2.51804	-3.47062	2.83007
H	-0.99792	-4.02344	2.0285	H	-1.00249	-3.42133	2.23651	H	-0.95961	-3.95019	2.18339
H	-0.63942	1.72367	2.67091	H	-2.0375	2.47656	2.88837	H	-2.46885	2.13503	2.49166
H	0.76187	-1.59035	1.52451	H	0.91319	-1.6513	1.52061	H	0.79531	-1.51326	1.60614
H	3.04449	0.13735	1.13627	H	3.13293	0.19974	1.01583	H	3.06297	0.22085	1.14726
		**1b-6**									
C	-1.28374	2.25471	-0.58366								
C	-1.67104	1.31619	0.60581								
C	-2.98435	0.53439	0.39333								
C	0.10074	2.92174	-0.3312								

C	-0.3329	-3.13508	0.19728
C	0.99389	-2.56705	-0.34962
C	1.84125	-1.72882	0.64091
C	2.94733	-0.86778	-0.05385
C	-3.17976	-0.62611	1.38899
C	-2.16437	-1.76561	1.38777
C	-1.42573	-2.09776	0.3074
C	1.30995	1.96575	-0.26367
C	2.57347	-0.042	-1.30627
C	1.27902	0.81237	-1.28438
C	0.89423	1.37811	-2.64574
C	1.71698	1.5546	-3.69523
C	-0.53699	1.7768	-2.84449
O	-0.98757	2.24653	-3.87807
O	-1.30906	1.43621	-1.78912
C	-2.33178	3.36461	-0.77472
C	2.49967	-2.6611	1.67732
C	-2.06726	-2.46807	2.71839
O	-1.74878	2.06126	1.82494
O	1.02062	-0.83611	1.39783
O	3.48923	0.02647	0.93721
H	-0.86365	0.59674	0.73025
H	-3.05416	0.14328	-0.62689
H	-3.83177	1.21849	0.52783
H	0.07262	3.51358	0.59269
H	0.2995	3.64884	-1.13084
H	-0.6888	-3.89355	-0.51188
H	-0.1505	-3.66321	1.13427
H	1.60786	-3.40302	-0.71064
H	0.75069	-1.98724	-1.24477
H	3.76605	-1.53741	-0.34845
H	-4.17083	-1.06641	1.21846
H	-3.21453	-0.19154	2.39653
H	-1.59489	-1.55793	-0.62274
H	2.22376	2.55918	-0.39935
H	1.36453	1.56817	0.75251
H	3.42065	0.61887	-1.53457
H	2.50023	-0.74969	-2.14327
H	0.48317	0.12186	-0.99858
H	1.35539	1.96528	-4.63616
H	2.77134	1.30852	-3.66027
H	-2.43595	3.98722	0.11938
H	-2.05817	4.01688	-1.61223
H	-3.31196	2.94724	-1.0285
H	1.74747	-3.19377	2.26865
H	3.15126	-3.3984	1.19721
H	3.09783	-2.09478	2.40037
H	-1.62746	-1.79825	3.46467
H	-3.06672	-2.75776	3.06125
H	-1.46929	-3.38061	2.69337
H	-1.64909	1.41799	2.54941
H	1.65414	-0.26013	1.87391
H	4.27822	0.44731	0.55123

Detailed DP4+ probability for compound **1**. Isomer 1 is 1R*,3R*,4S*,11S*,12S*, isomer 2 is 1R*,3R*,4S*,11S*,12R*.

Functional	Solvent?	Basis Set	Type of Data		
B3LYP	PCM	6-311+G(d,p)	Unscaled Shifts		

	Isomer 1	Isomer 2	Isomer 3	Isomer 4	Isomer 5	Isomer 6
sDP4+ (H data)	0.00%	100.00%	–	–	–	–
sDP4+ (C data)	100.00%	0.00%	–	–	–	–
sDP4+ (all data)	98.78%	1.22%	–	–	–	–
uDP4+ (H data)	0.00%	100.00%	–	–	–	–
uDP4+ (C data)	0.00%	100.00%	–	–	–	–
uDP4+ (all data)	0.00%	100.00%	–	–	–	–
DP4+ (H data)	0.00%	100.00%	–	–	–	–
DP4+ (C data)	0.00%	100.00%	–	–	–	–
DP4+ (all data)	0.00%	100.00%	–	–	–	–

Table S7.Calculation process of 2

Important thermodynamic parameters (a.u.) of the optimized **2** with simplified structures at B3LYP/6-31+G(d,p) level in the gas phase.

NO.	E+ZPE	G	p%	NO.	E+ZPE	G	p%	NO.	E+ZPE	G	p%
2a1	-1158.74647	-1158.277391	43.08%	2b4	-1158.748566	-1158.279053	3.01%	2c15	-1158.745189	-1158.276066	8.81%
2a2	-1158.744783	-1158.275551	6.13%	2b5	-1158.750016	-1158.280051	8.67%	2c18	-1158.746077	-1158.276178	9.93%
2a3	-1158.74374	-1158.275763	7.67%	2b6	-1158.750932	-1158.279247	3.70%	2c22	-1158.745218	-1158.276487	13.77%
2a4	-1158.742905	-1158.274204	1.47%	2c1	-1158.747194	-1158.275921	7.56%	2c23	-1158.746637	-1158.275475	4,71%
2a5	-1158.744713	-1158.276061	10.52%	2c2	-1158.744293	-1158.275596	5.36%	2d1	-1158.749139	-1158.278016	22.96%
2a6	-1158.745444	-1158.275793	7.92%	2c3	-1158.743816	-1158.275331	4.04%	2d2	-1158.749346	-1158.278151	26.49%
2a7	-1158.743447	-1158.27388	1.04%	2c5	-1158.745218	-1158.276491	13.83%	2d3	-1158.747652	-1158.276386	4.08%
2a11	-1158.744689	-1158.275493	5.76%	2c6	-1158.744499	-1158.275417	4.43%	2d4	-1158.74565	-1158.275189	1.15%
2a13	-1158.744858	-1158.275991	9.77%	2c7	-1158.74608	-1158.275124	3.25%	2d5	-1158.749138	-1158.27802	23.06%
2a19	-1158.744682	-1158.274502	2.02%	2c8	-1158.744375	-1158.275423	4.46%	2d6	-1158.749209	-1158.277207	9.74%
2b1	-1158.751576	-1158.281403	36.32%	2c11	-1158.747194	-1158.275925	7.56%	2d7	-1158.749698	-1158.277444	12.52%
2b2	-1158.751576	-1158.2814	36.21%	2c13	-1158.745053	-1158.275384	4.28%				
2b3	-1158.750303	-1158.280366	12.10%	2c14	-1158.747056	-1158.27592	7.55%				

Optimized Z-Matrixes of **2** with simplified structures in the Gas Phase (Å) at B3LYP/6-31+G(d,p) level.

2a1				2a2				2a3			
O	1.56074	-1.81971	-3.39275	O	3.554	2.35122	1.11356	O	1.70845	-1.81213	-3.38253
O	0.40633	-3.95811	-2.08147	O	4.37784	-0.21519	1.09994	O	0.42777	-3.93654	-2.16603
C	2.62989	-3.57538	-1.3007	C	3.03958	0.1761	3.03627	C	2.61072	-3.6162	-1.2768
C	-3.23617	-0.82501	0.40671	C	0.07561	-3.76213	0.26617	C	-3.29058	-0.80849	0.24672
O	0.04095	1.91056	3.39026	O	-3.65484	-0.97606	0.82892	O	-0.08295	1.70726	3.49192
O	-1.17593	3.42671	-1.91766	O	-1.20202	2.02976	-3.25528	O	-1.04954	3.47302	-1.79357
O	-1.1482	2.08313	-0.10249	O	-1.37311	0.33764	-1.76723	O	-1.11543	2.05461	-0.03625
C	-0.70873	2.46244	-1.3272	C	-0.89717	1.53912	-2.17713	C	-0.62049	2.47995	-1.22245
C	1.29309	2.26244	-2.80911	C	0.07707	3.64813	-1.25067	C	1.43996	2.31745	-2.6255
C	-0.96559	3.92751	1.44938	C	-3.77866	0.38961	-1.81492	C	-0.96256	3.82849	1.59966
C	0.31521	1.49254	-1.91793	C	0.14315	2.11962	-1.21934	C	0.41373	1.52168	-1.81429
C	1.00683	0.62363	-0.8323	C	0.0413	1.5168	0.20897	C	1.04574	0.58692	-0.74748
C	1.81221	1.45497	0.19134	C	-1.31432	1.80283	0.89308	C	1.81643	1.35128	0.35241
C	1.9035	-0.50355	-1.4049	C	1.19688	1.92462	1.15321	C	1.9548	-0.51977	-1.34312
C	-2.43213	-0.23963	2.76499	C	-1.26836	-2.66864	-1.62053	C	-2.58354	-0.34887	2.66394
C	-2.2546	-1.09304	1.51962	C	0.00978	-2.70146	-0.79925	C	-2.36525	-1.14509	1.38817
C	-1.27882	-2.02191	1.45054	C	1.00774	-1.83328	-1.0598	C	-1.40237	-2.08702	1.31955
C	1.11181	2.70409	0.74707	C	-2.56248	1.59011	0.02302	C	1.11681	2.58837	0.9353
C	-0.4026	2.55086	1.05434	C	-2.537	0.34066	-0.90547	C	-0.41238	2.45658	1.17076
C	-0.63602	1.50639	2.19463	C	-2.51901	-0.97529	-0.05208	C	-0.71865	1.37109	2.25364
C	-2.12513	1.24994	2.52016	C	-2.5306	-2.31268	-0.82315	C	-2.22652	1.14106	2.50605
C	1.09642	-1.65146	-2.04404	C	2.57685	1.34357	0.78629	C	1.16507	-1.63468	-2.06087
C	-0.89028	-2.81372	0.22772	C	2.31593	-1.68048	-0.33306	C	-0.96332	-2.82601	0.08007
C	0.63823	-2.97505	0.13345	C	2.16552	-1.21284	1.12678	C	0.56572	-3.00196	0.05987
C	1.19225	-3.0285	-1.3088	C	3.00981	0.02275	1.50509	C	1.19098	-3.02908	-1.35158

H	1.1192	-2.6349	-3.70663	H	4.4163	1.89877	1.00771	H	1.60026	-0.96995	-3.85888
H	0.48271	-4.83375	-1.6619	H	4.69124	-1.01346	1.56178	H	0.75801	-3.79695	-3.07688
H	3.027	-3.66623	-2.31763	H	3.641	1.03964	3.34011	H	3.07071	-3.67998	-2.26901
H	3.31066	-2.95097	-0.7157	H	2.03809	0.27918	3.46298	H	3.26925	-3.03418	-0.62622
H	2.65599	-4.58668	-0.87754	H	3.51067	-0.69698	3.50378	H	2.58118	-4.64376	-0.89483
H	-3.31416	-1.65962	-0.29576	H	-0.20031	-4.73583	-0.15217	H	-3.33974	-1.60251	-0.50376
H	-4.24107	-0.67365	0.81561	H	1.07556	-3.87243	0.69407	H	-4.31279	-0.67111	0.61567
H	-2.9531	0.06971	-0.15368	H	-0.61332	-3.5272	1.08331	H	-2.97626	0.11302	-0.24986
H	-0.11305	1.22351	4.06161	H	-3.58	-1.75487	1.4073	H	-0.27779	0.99381	4.12442
H	0.75443	2.85138	-3.56018	H	0.15942	4.02081	-2.27798	H	0.94261	2.95726	-3.36333
H	1.90686	2.96203	-2.23382	H	-0.86777	4.02528	-0.84812	H	2.04482	2.97156	-1.99042
H	1.95858	1.588	-3.35462	H	0.89716	4.09771	-0.68413	H	2.1126	1.65953	-3.18224
H	-0.80829	4.65824	0.6478	H	-3.82279	1.336	-2.36633	H	-0.75326	4.58847	0.83815
H	-2.04799	3.8861	1.61	H	-3.74486	-0.39536	-2.57752	H	-2.05174	3.8065	1.71028
H	-0.49278	4.31547	2.3572	H	-4.70787	0.29026	-1.24526	H	-0.52278	4.16642	2.54337
H	-0.27196	0.82836	-2.56458	H	1.10495	1.81955	-1.65226	H	-0.1548	0.8994	-2.51708
H	0.21744	0.10987	-0.27139	H	0.10854	0.43327	0.11085	H	0.22581	0.05747	-0.2485
H	2.07416	0.80288	1.03508	H	-1.39655	1.15942	1.77899	H	2.02504	0.65379	1.17444
H	2.76878	1.76492	-0.24861	H	-1.33199	2.83229	1.27311	H	2.79882	1.66084	-0.02687
H	2.62145	-0.08797	-2.12029	H	1.25542	3.01868	1.20014	H	2.69913	-0.07743	-2.0138
H	2.51372	-0.89426	-0.58373	H	0.93787	1.62505	2.17412	H	2.53787	-0.95261	-0.52325
H	-3.46708	-0.34051	3.11493	H	-1.17094	-1.99285	-2.47712	H	-3.63655	-0.4378	2.95875
H	-1.79948	-0.60551	3.58346	H	-1.41044	-3.6631	-2.06431	H	-2.00101	-0.77013	3.49293
H	-0.65997	-2.19203	2.33124	H	0.87797	-1.13275	-1.88516	H	-0.82956	-2.31297	2.2187
H	1.64862	3.03434	1.64563	H	-3.44368	1.56178	0.67652	H	1.62068	2.86575	1.87009
H	1.25115	3.51453	0.01947	H	-2.69727	2.49181	-0.58893	H	1.30417	3.42773	0.25264
H	-0.1685	0.5673	1.89052	H	-1.63576	-0.96855	0.59435	H	-0.26062	0.43466	1.92762
H	-2.77765	1.60732	1.71646	H	-3.39796	-2.34665	-1.4932	H	-2.83523	1.55467	1.69493
H	-2.40672	1.80827	3.42186	H	-2.72236	-3.12237	-0.1053	H	-2.53111	1.66324	3.42181
H	0.04285	-1.37479	-2.14775	H	2.66921	1.21533	-0.29534	H	0.12366	-1.34069	-2.22444
H	-1.35196	-3.8064	0.28592	H	2.85509	-2.63572	-0.36284	H	-1.44005	-3.81302	0.06526
H	-1.27418	-2.33131	-0.67416	H	2.9353	-0.98413	-0.90957	H	-1.29164	-2.2921	-0.81495
H	1.15473	-2.18789	0.69562	H	2.49812	-2.0539	1.75186	H	1.06116	-2.23394	0.66588
H	0.88631	-3.91589	0.64468	H	1.11845	-1.04315	1.40117	H	0.77655	-3.95692	0.56177
2a4				2a5				2a6			
O	3.4634	2.29119	1.27312	O	2.25965	-2.19879	-2.76419	O	3.39987	0.58971	-2.35781
O	4.30857	-0.29023	0.90142	O	1.04642	-4.21329	-1.31686	O	3.32795	-2.17675	-2.11754
C	3.16101	-0.06954	2.98196	C	3.06674	-3.50245	-0.28113	C	4.46791	-1.2057	-0.27552
C	0.45677	-4.19528	0.09857	C	-3.20422	-1.14421	0.05931	C	-2.04475	-2.30497	-1.4448
O	-3.52495	-1.30426	0.1551	O	-0.66868	2.25549	3.08977	O	-2.24661	0.09729	3.17318
O	-1.29056	2.91811	-2.85367	O	-1.09035	2.95524	-2.51693	O	-2.23285	2.94417	-1.70365
O	-1.34011	0.88151	-1.87377	O	-1.27252	1.87239	-0.54348	O	-2.03403	1.13602	-0.36398
C	-0.94917	2.17854	-1.94134	C	-0.66424	2.12428	-1.72678	C	-1.52265	2.13917	-1.11715
C	-0.13535	4.02392	-0.46393	C	1.56095	1.91874	-2.84241	C	0.56426	3.48519	-1.38653
C	-3.74997	0.75717	-1.9518	C	-1.47966	3.91878	0.7206	C	-3.51416	2.04856	1.31681
C	0.04604	2.55223	-0.84238	C	0.50916	1.18487	-2.00598	C	-0.00031	2.06929	-1.24303
C	-0.01954	1.57978	0.36698	C	1.07722	0.53572	-0.71489	C	0.65638	1.2453	-0.10116
C	-1.39046	1.59238	1.07991	C	1.6349	1.56716	0.292	C	0.40666	1.84108	1.30286
C	1.11825	1.77518	1.39897	C	2.14461	-0.55815	-0.97935	C	2.17577	1.00163	-0.29594
C	-1.40494	-3.19115	-1.30623	C	-2.83639	-0.16879	2.39959	C	-2.59886	-2.45729	1.04979
C	0.01277	-3.04	-0.76132	C	-2.39618	-1.15689	1.33112	C	-1.54468	-2.53827	-0.04172
C	0.78944	-1.96707	-1.0156	C	-1.35077	-1.98113	1.5472	C	-0.25814	-2.80925	0.25941
C	-2.62485	1.52526	0.16406	C	0.76486	2.80769	0.54535	C	-1.03126	2.29038	1.6033
C	-2.508	0.57703	-1.0621	C	-0.76506	2.55848	0.63233	C	-2.15767	1.36642	1.06556
C	-2.371	-0.911	-0.59535	C	-1.1148	1.66104	1.86546	C	-2.10431	-0.03626	1.7544
C	-2.14572	-1.91248	-1.7506	C	-2.61316	1.29809	1.98658	C	-3.16213	-1.03533	1.23204
C	2.49704	1.29308	0.90116	C	1.55025	-1.85957	-1.55768	C	2.48998	-0.01109	-1.41786
C	2.14665	-1.64139	-0.45156	C	-0.70299	-2.88964	0.53292	C	0.91057	-2.75353	-0.69256
C	2.08828	-1.2714	1.04169	C	0.82682	-2.90162	0.70343	C	2.15266	-2.15823	-0.00474
C	2.9899	-0.08667	1.45253	C	1.61847	-3.10018	-0.60722	C	3.09522	-1.37612	-0.9464
H	4.32935	1.88919	1.05638	H	2.13324	-1.4668	-3.39335	H	4.12761	1.00275	-1.86066

H	4.65502	-1.12741	1.25896	H	1.47027	-4.19454	-2.19891	H	3.63462	-1.55608	-2.8095
H	3.82156	0.7439	3.30109	H	3.64695	-3.68374	-1.19253	H	4.41138	-0.62489	0.6491
H	2.20737	0.02859	3.50756	H	3.58539	-2.74682	0.3153	H	4.90069	-2.18391	-0.03331
H	3.63777	-0.9947	3.32767	H	3.08622	-4.44568	0.27794	H	5.18652	-0.72461	-0.94762
H	0.3361	-5.13692	-0.44761	H	-3.09152	-2.06467	-0.52048	H	-1.36721	-2.70372	-2.20509
H	1.50575	-4.13051	0.3982	H	-4.27145	-1.05448	0.28915	H	-3.00485	-2.81086	-1.59386
H	-0.14938	-4.24226	1.00912	H	-2.9157	-0.30232	-0.57547	H	-2.18381	-1.2378	-1.63552
H	-3.35895	-2.19482	0.50874	H	-1.27376	2.98204	3.31808	H	-2.19043	-0.79451	3.55844
H	-0.06337	4.66453	-1.35027	H	2.02546	2.73883	-2.28668	H	0.06828	4.02196	-2.20322
H	-1.11352	4.21308	-0.01181	H	2.34975	1.2427	-3.183	H	0.41769	4.07749	-0.47831
H	0.63715	4.35951	0.23314	H	1.10983	2.35502	-3.74089	H	1.63199	3.47212	-1.62127
H	-3.84632	1.79738	-2.28313	H	-1.25994	4.53314	-0.16004	H	-3.5538	3.03	0.83059
H	-3.67462	0.15584	-2.86413	H	-2.56784	3.79799	0.7393	H	-4.33919	1.46863	0.89016
H	-4.67115	0.48414	-1.42748	H	-1.17569	4.48371	1.60727	H	-3.70518	2.19338	2.38487
H	1.03073	2.4488	-1.31453	H	0.0851	0.38798	-2.63	H	0.17685	1.54304	-2.1895
H	0.11798	0.5692	-0.02324	H	0.24812	0.02207	-0.21448	H	0.19609	0.25049	-0.1086
H	-1.42788	0.74007	1.77111	H	1.79686	1.05828	1.25147	H	0.69675	1.09158	2.05111
H	-1.47656	2.48655	1.71032	H	2.62737	1.90589	-0.03207	H	1.0742	2.69629	1.46947
H	1.16781	2.8271	1.70147	H	2.9333	-0.16747	-1.63121	H	2.68676	1.9536	-0.47466
H	0.85176	1.23347	2.31251	H	2.64558	-0.77324	-0.02948	H	2.5829	0.63498	0.65244
H	-1.36502	-3.88428	-2.15619	H	-3.9029	-0.32633	2.60344	H	-3.41967	-3.14018	0.79764
H	-2.02012	-3.68021	-0.53995	H	-2.31639	-0.35666	3.34743	H	-2.19728	-2.80845	2.00866
H	0.42056	-1.19664	-1.69016	H	-0.87149	-1.96419	2.52636	H	-0.00174	-3.03579	1.29396
H	-3.49644	1.25146	0.77225	H	1.11928	3.30151	1.45924	H	-1.13605	2.42251	2.68783
H	-2.83324	2.54412	-0.18804	H	0.96704	3.52474	-0.26131	H	-1.15912	3.29616	1.18164
H	-1.5268	-0.96943	0.09546	H	-0.53982	0.73758	1.77146	H	-1.10687	-0.45097	1.59289
H	-1.59179	-1.45818	-2.57856	H	-3.14372	1.47611	1.04518	H	-3.58266	-0.70833	0.2751
H	-3.1188	-2.2174	-2.15658	H	-3.09332	1.93669	2.73828	H	-3.99984	-1.08868	1.93876
H	2.52814	1.27433	-0.19217	H	0.51298	-1.71509	-1.87526	H	1.59472	-0.21686	-2.01182
H	2.83259	-2.48248	-0.60782	H	-1.08796	-3.90705	0.66877	H	1.14059	-3.76941	-1.03438
H	2.55665	-0.81709	-1.04575	H	-0.96683	-2.57494	-0.47962	H	0.6517	-2.16408	-1.5754
H	2.41381	-2.16326	1.59516	H	1.17655	-1.99365	1.20917	H	1.87029	-1.52281	0.84315
H	1.06035	-1.0806	1.37247	H	1.05801	-3.72985	1.38816	H	2.70323	-3.00449	0.42994
2a7				2a8				2a9			
O	3.5517	2.33096	1.18309	O	3.51091	2.16731	1.32397	O	1.63262	-1.84826	-3.40233
O	4.40463	-0.23577	1.0309	O	4.31899	-0.41798	0.92373	O	0.46567	-4.00767	-2.09665
C	3.09813	0.02565	2.99253	C	3.17183	-0.21504	3.00575	C	2.64109	-3.59776	-1.26411
C	0.18851	-3.80252	0.04804	C	0.36036	-4.21825	0.03485	C	-3.25089	-0.82165	0.44414
O	-3.5987	-1.14903	0.81351	O	-3.53148	-1.2656	0.10831	O	0.07648	1.90121	3.40305
O	-1.29126	2.14273	-3.12529	O	-1.21813	3.03466	-2.75089	O	-1.20136	3.3729	-1.90389
O	-1.38962	0.36011	-1.73861	O	-1.30977	0.96351	-1.85243	O	-1.13615	2.03928	-0.08229
C	-0.95085	1.59441	-2.08645	C	-0.88699	2.25238	-1.87097	C	-0.71288	2.4202	-1.31207
C	-0.00314	3.67005	-1.06327	C	-0.02105	4.0231	-0.33406	C	1.28569	2.24227	-2.80203
C	-3.79649	0.35095	-1.7539	C	-3.71805	0.89903	-1.94053	C	-0.94888	3.90042	1.45122
C	0.093	2.14351	-1.11388	C	0.11817	2.56011	-0.76106	C	0.31768	1.46152	-1.90955
C	0.02324	1.46546	0.2827	C	0.02148	1.55129	0.41588	C	1.01961	0.59182	-0.83101
C	-1.33089	1.68039	0.99575	C	-1.35182	1.57717	1.12444	C	1.82453	1.42407	0.19286
C	1.17884	1.85449	1.23562	C	1.15887	1.68281	1.45832	C	1.92472	-0.52354	-1.41402
C	-1.21133	-2.64688	-1.75963	C	-1.45757	-3.12813	-1.35933	C	-2.41112	-0.25414	2.79404
C	0.0787	-2.68892	-0.95843	C	-0.0363	-3.0335	-0.80821	C	-2.25063	-1.09583	1.53854
C	1.05087	-1.78272	-1.18495	C	0.77797	-1.98233	-1.03521	C	-1.2719	-2.01951	1.44713
C	-2.58513	1.48231	0.13084	C	-2.58456	1.56812	0.20377	C	1.12596	2.67406	0.74831
C	-2.54142	0.28539	-0.86419	C	-2.48497	0.65912	-1.05275	C	-0.38598	2.5208	1.06583
C	-2.47808	-1.07368	-0.08372	C	-2.38148	-0.84616	-0.63489	C	-0.61087	1.48671	2.2172
C	-2.46964	-2.36723	-0.92639	C	-2.14298	-1.82141	-1.80938	C	-2.09774	1.23556	2.55852
C	2.57145	1.33056	0.82634	C	2.5277	1.18815	0.94592	C	1.13452	-1.67611	-2.06671
C	2.36531	-1.63579	-0.46584	C	2.14122	-1.71767	-0.45463	C	-0.89334	-2.78995	0.20901
C	2.22129	-1.25318	1.01916	C	2.08401	-1.37032	1.04429	C	0.63011	-2.98812	0.11881
C	3.0438	-0.02323	1.45607	C	3.00201	-0.20578	1.47628	C	1.20516	-3.04778	-1.31607
H	3.3535	2.64526	2.08298	H	4.36964	1.75054	1.10599	H	1.37638	-2.75135	-3.67628
H	4.81412	0.65363	1.00233	H	4.64877	-1.26993	1.26181	H	-0.44928	-3.6936	-2.19883

H	3.71021	0.86079	3.3487	H	3.8413	0.5858	3.33796	H	3.07448	-3.68238	-2.26668
H	2.1042	0.10218	3.44172	H	2.21909	-0.11431	3.53245	H	3.30375	-2.98041	-0.65137
H	3.57475	-0.8794	3.38871	H	3.6377	-1.15099	3.33691	H	2.64843	-4.61297	-0.84914
H	-0.0717	-4.75923	-0.41703	H	0.21445	-5.1454	-0.52965	H	-3.31996	-1.64066	-0.27747
H	1.19808	-3.91116	0.45305	H	1.40759	-4.19383	0.34631	H	-4.25332	-0.7008	0.86888
H	-0.4915	-3.62808	0.8875	H	-0.25636	-4.26159	0.93848	H	-2.99369	0.0913	-0.09884
H	-3.50353	-1.96388	1.33692	H	-4.26932	-1.39272	-0.5126	H	-0.06772	1.21825	4.08086
H	0.05429	4.09806	-2.07052	H	0.07103	4.69043	-1.19857	H	0.73935	2.83408	-3.54525
H	-0.94813	4.00585	-0.62586	H	-0.99426	4.22518	0.12327	H	1.90075	2.94037	-2.22645
H	0.81783	4.10586	-0.48731	H	0.75969	4.31281	0.37434	H	1.95016	1.57444	-3.35691
H	-3.87328	1.32474	-2.25136	H	-3.77302	1.94618	-2.25919	H	-0.79523	4.62453	0.64295
H	-3.75278	-0.38958	-2.55925	H	-3.66524	0.30583	-2.85972	H	-2.03059	3.85998	1.61629
H	-4.71467	0.19531	-1.17897	H	-4.65068	0.65847	-1.421	H	-0.47278	4.29649	2.35382
H	1.05405	1.88577	-1.57487	H	1.09995	2.44374	-1.23615	H	-0.26704	0.79565	-2.55668
H	0.11625	0.39076	0.12702	H	0.13512	0.5502	-0.00594	H	0.2366	0.06988	-0.2689
H	-1.38576	0.98937	1.84727	H	-1.41318	0.70587	1.78979	H	2.08722	0.77195	1.03635
H	-1.36923	2.68764	1.42998	H	-1.41792	2.45386	1.78127	H	2.78082	1.73419	-0.24772
H	1.20823	2.94584	1.3376	H	1.23034	2.72099	1.80114	H	2.63925	-0.09359	-2.12456
H	0.94133	1.49487	2.24244	H	0.87512	1.11179	2.34847	H	2.54003	-0.91581	-0.59741
H	-1.14432	-1.92376	-2.57971	H	-1.43992	-3.82248	-2.20902	H	-3.44279	-0.35261	3.15408
H	-1.3345	-3.61967	-2.25411	H	-2.0973	-3.5893	-0.5953	H	-1.77171	-0.63073	3.60236
H	0.8945	-1.04455	-1.9715	H	0.43946	-1.18324	-1.69131	H	-0.64102	-2.1968	2.3182
H	-3.45639	1.39655	0.79258	H	-3.46383	1.29263	0.80009	H	1.66822	3.00878	1.64193
H	-2.75051	2.41198	-0.42971	H	-2.76922	2.60268	-0.11431	H	1.25961	3.48196	0.01689
H	-1.58501	-1.07975	0.54901	H	-1.54295	-0.93749	0.05867	H	-0.14636	0.54476	1.91679
H	-3.34627	-2.38675	-1.5848	H	-1.54757	-1.36302	-2.60533	H	-2.75765	1.60364	1.76576
H	-2.63005	-3.21934	-0.25123	H	-3.10627	-2.09107	-2.26059	H	-2.36584	1.78773	3.46808
H	2.65264	1.26109	-0.26118	H	2.55235	1.18183	-0.14775	H	0.08458	-1.39997	-2.20053
H	2.92901	-2.5729	-0.5557	H	2.7956	-2.58215	-0.61869	H	-1.38255	-3.77058	0.23336
H	2.95742	-0.89142	-1.01021	H	2.58865	-0.90112	-1.03235	H	-1.25527	-2.2655	-0.67853
H	2.57996	-2.12107	1.59101	H	2.3949	-2.27549	1.58442	H	1.15929	-2.21436	0.68833
H	1.17435	-1.12305	1.31447	H	1.05792	-1.16929	1.37492	H	0.8518	-3.93633	0.62868
2a10				2b1				2b2			
O	2.20186	1.06911	-3.03652	O	2.90033	1.26292	-1.95945	O	2.90193	1.23539	-1.9604
O	2.88008	-1.56009	-3.21084	O	4.2419	-0.86774	-1.01661	O	4.25975	-0.90069	-0.97677
C	4.27687	-0.52894	-1.60241	C	4.09329	0.7543	0.73055	C	4.0732	0.73379	0.77382
C	-0.40478	-3.47472	1.47183	C	0.08422	-3.26823	2.34997	C	0.10114	-3.2756	2.35808
O	-1.7708	0.02473	3.62423	O	-2.98736	-0.47551	1.75769	O	-2.97968	-0.48661	1.78253
O	-2.86467	2.82168	-1.16636	O	-1.12502	2.29118	1.91184	O	-1.1093	2.27931	1.92804
O	-2.27247	1.04022	0.09402	O	-2.7611	1.97948	0.39939	O	-2.75035	1.96709	0.42077
C	-1.99394	2.07681	-0.73865	C	-1.52042	2.38319	0.75806	C	-1.50825	2.36961	0.77546
C	-0.08479	3.55439	-1.41246	C	0.06952	4.18416	0.11751	C	0.08082	4.16897	0.12783
C	-3.42848	1.81532	2.04674	C	-4.4099	0.59854	-0.55393	C	-4.40414	0.58835	-0.52716
C	-0.52782	2.11204	-1.16737	C	-0.74489	2.98948	-0.39	C	-0.7355	2.97391	-0.37571
C	0.37951	1.34517	-0.17263	C	0.10607	1.93345	-1.12753	C	0.11286	1.91661	-1.1148
C	0.40442	1.97856	1.23353	C	-0.72498	0.83746	-1.83463	C	-0.72092	0.81756	-1.8139
C	1.81778	1.08462	-0.66675	C	1.21711	1.30535	-0.23698	C	1.22969	1.29299	-0.22807
C	-2.2618	-2.01249	0.47404	C	-1.79869	-2.94181	0.6856	C	-1.79445	-2.95648	0.70639
C	-0.85486	-2.58809	0.33898	C	-0.29849	-2.8762	0.94411	C	-0.29253	-2.89166	0.95297
C	-0.12773	-2.35055	-0.77513	C	0.59908	-2.5431	-0.00743	C	0.5984	-2.56644	-0.00757
C	-0.97797	2.29974	1.8236	C	-2.25246	0.90736	-1.76616	C	-2.2482	0.89072	-1.74398
C	-2.09316	1.25788	1.51654	C	-2.88594	0.74517	-0.36468	C	-2.87954	0.73185	-0.34098
C	-1.73987	-0.11444	2.19437	C	-2.31554	-0.43773	0.48966	C	-2.30988	-0.45151	0.51353
C	-2.60998	-1.32414	1.80594	C	-2.43569	-1.82428	-0.15925	C	-2.43548	-1.83823	-0.13441
C	1.85586	0.2136	-1.93494	C	2.22732	0.43089	-1.00019	C	2.23383	0.40975	-0.99176
C	1.20145	-2.94959	-1.15773	C	2.10101	-2.57597	0.12083	C	2.10237	-2.5957	0.11131
C	2.44698	-2.09486	-0.86415	C	2.72759	-1.35947	0.82249	C	2.7267	-1.38408	0.82337
C	2.83653	-0.99219	-1.88485	C	3.29688	-0.26121	-0.10589	C	3.30162	-0.27809	-0.09299
H	2.6442	0.50074	-3.69852	H	3.66187	0.72247	-2.25605	H	3.41568	0.61712	-2.51302
H	1.97031	-1.76452	-3.48948	H	4.94547	-1.27813	-0.48224	H	4.94489	-0.23825	-1.17837
H	4.97971	-1.36333	-1.71375	H	4.93656	0.26709	1.23434	H	4.92991	0.25193	1.25969

H	4.59957	0.23245	-2.32119	H	4.52939	1.53537	0.09822	H	4.4849	1.54694	0.16573
H	4.39189	-0.12301	-0.59316	H	3.47403	1.22887	1.49773	H	3.44254	1.16965	1.55479
H	0.57918	-3.92063	1.31248	H	-0.24321	-4.29252	2.55662	H	-0.23416	-4.29488	2.57673
H	-0.3395	-2.90391	2.40328	H	1.15939	-3.22105	2.53854	H	1.17867	-3.23862	2.53495
H	-1.11779	-4.29328	1.61462	H	-0.39761	-2.59739	3.06944	H	-0.36674	-2.59409	3.07668
H	-2.69685	-0.04265	3.9159	H	-2.82491	0.39055	2.18276	H	-2.80906	0.37668	2.20991
H	-0.71632	4.02767	-2.17288	H	-0.60028	4.96268	0.50082	H	-0.58757	4.94836	0.51181
H	-0.16209	4.16638	-0.50869	H	0.74203	3.9166	0.93838	H	0.75538	3.9022	0.94728
H	0.94633	3.60144	-1.77386	H	0.66527	4.6226	-0.68955	H	0.6746	4.60593	-0.68149
H	-3.6776	2.76282	1.55489	H	-4.92992	0.54578	0.40904	H	-4.92244	0.53813	0.43688
H	-4.2591	1.13705	1.82733	H	-4.81949	1.47598	-1.06914	H	-4.81275	1.46596	-1.04286
H	-3.40098	1.99916	3.12518	H	-4.67047	-0.28817	-1.13947	H	-4.66778	-0.29862	-1.11093
H	-0.51251	1.58996	-2.13164	H	-1.4685	3.42149	-1.09344	H	-1.46098	3.40534	-1.07759
H	-0.04256	0.34316	-0.0547	H	0.62386	2.48233	-1.92759	H	0.62578	2.46358	-1.91931
H	0.92242	1.29388	1.91765	H	-0.44826	0.85513	-2.89812	H	-0.44513	0.82763	-2.87772
H	1.00568	2.89628	1.22646	H	-0.42094	-0.15228	-1.49086	H	-0.41861	-0.17036	-1.46363
H	2.33847	2.03551	-0.82892	H	1.77216	2.12281	0.23324	H	1.78939	2.11331	0.23171
H	2.34749	0.58	0.14856	H	0.75763	0.72305	0.56606	H	0.77427	0.71747	0.58234
H	-2.47995	-1.33444	-0.3574	H	-2.00712	-3.90595	0.20305	H	-2.0077	-3.9206	0.22595
H	-2.96116	-2.85079	0.34921	H	-2.32803	-2.97937	1.64674	H	-2.31653	-2.99261	1.67154
H	-0.57839	-1.72674	-1.54624	H	0.24264	-2.31561	-1.00935	H	0.23534	-2.34784	-1.0088
H	-0.87261	2.43349	2.90788	H	-2.63505	0.11222	-2.42018	H	-2.63327	0.09516	-2.39601
H	-1.28485	3.28253	1.44232	H	-2.58543	1.84974	-2.219	H	-2.58006	1.83289	-2.19805
H	-0.70437	-0.37402	1.96148	H	-1.2659	-0.24692	0.72333	H	-1.25914	-0.26281	0.74484
H	-3.66561	-1.03	1.78157	H	-2.00573	-1.83019	-1.16493	H	-2.0115	-1.84481	-1.14263
H	-2.55546	-2.07243	2.60815	H	-3.4965	-2.08107	-0.26872	H	-3.49725	-2.09379	-0.23744
H	0.86451	-0.16696	-2.1882	H	1.72084	-0.33421	-1.59416	H	1.71696	-0.35656	-1.57536
H	1.32702	-3.91023	-0.64512	H	2.3792	-3.4819	0.67508	H	2.38564	-3.5069	0.65431
H	1.16103	-3.22289	-2.21797	H	2.5326	-2.72237	-0.8762	H	2.52824	-2.72949	-0.88991
H	3.2825	-2.80937	-0.83563	H	2.03192	-0.92523	1.55006	H	2.02821	-0.95545	1.55162
H	2.37915	-1.67712	0.14731	H	3.56868	-1.75406	1.41055	H	3.5662	-1.78342	1.41075
2b3				2b4				2b5			
O	3.19977	0.65726	1.81007	O	3.0853	1.63794	-1.4051	O	2.98728	1.61585	-1.5155
O	3.08874	-1.93122	2.60376	O	4.39786	-0.57745	-0.6485	O	4.40511	-0.5376	-0.69566
C	1.5296	-0.69997	3.90479	C	3.98557	0.74533	1.28577	C	3.98715	0.81981	1.19577
C	-1.92731	-3.56334	0.0986	C	0.06813	-3.60229	1.82668	C	0.02647	-3.49143	1.97471
O	-3.06851	0.05875	-1.68996	O	-3.09277	-0.92015	1.36219	O	-3.0984	-0.82383	1.40144
O	-2.2256	2.0841	0.84746	O	-1.39693	1.83678	2.15024	O	-1.39406	1.96445	2.08235
O	-1.80479	2.54588	-1.31537	O	-2.86667	1.73612	0.44806	O	-2.87192	1.80006	0.39361
C	-1.45728	2.49811	-0.00924	C	-1.68995	2.11615	0.99668	C	-1.6891	2.19601	0.91832
C	-0.08288	3.84694	1.5642	C	-0.15653	4.06625	0.83458	C	-0.13393	4.11928	0.6678
C	-1.85065	1.91532	-3.58195	C	-4.3367	0.46897	-0.8822	C	-4.35871	0.49029	-0.87519
C	-0.07564	3.05388	0.25297	C	-0.84637	2.94355	0.05268	C	-0.84308	2.97469	-0.0637
C	1.00238	1.94987	0.20931	C	0.13176	2.06971	-0.76391	C	0.11868	2.05608	-0.84803
C	1.17243	1.29487	-1.18163	C	-0.56112	1.05857	-1.70665	C	-0.59185	1.0334	-1.76465
C	0.82857	0.86972	1.31679	C	1.20847	1.36551	0.11128	C	1.17821	1.35607	0.05183
C	-1.51231	-2.40265	-2.11592	C	-1.65905	-3.12191	0.03479	C	-1.68002	-3.07082	0.14963
C	-0.93884	-2.82352	-0.76884	C	-0.199	-3.02683	0.45759	C	-0.22213	-2.96779	0.58109
C	0.34283	-2.59745	-0.41047	C	0.76588	-2.51928	-0.33844	C	0.75396	-2.50009	-0.22609
C	0.31172	1.79428	-2.34512	C	-2.08954	1.05596	-1.79336	C	-2.12102	1.03754	-1.83084
C	-1.21351	1.56844	-2.22053	C	-2.84937	0.63997	-0.51108	C	-2.86692	0.67079	-0.52661
C	-1.63677	0.13286	-1.75837	C	-2.30096	-0.64524	0.19762	C	-2.31451	-0.58892	0.22258
C	-1.13871	-1.01037	-2.65433	C	-2.27891	-1.90704	-0.67729	C	-2.29385	-1.88046	-0.60689
C	2.02264	-0.09406	1.45418	C	2.3268	0.6537	-0.67498	C	2.29657	0.63595	-0.72288
C	1.02884	-3.09162	0.83934	C	2.24843	-2.50467	-0.05706	C	2.23395	-2.51272	0.06214
C	0.75041	-2.27122	2.10968	C	2.72643	-1.39255	0.891	C	2.75962	-1.34805	0.91647
C	1.83136	-1.236	2.49618	C	3.32864	-0.14082	0.21455	C	3.32684	-0.11694	0.16907
H	2.97232	1.24382	2.55305	H	2.54335	1.9249	-2.16013	H	3.90677	1.2944	-1.60875
H	3.7811	-1.25078	2.477	H	4.63313	0.21614	-1.17167	H	4.03275	-1.0593	-1.42713
H	2.30232	-0.00213	4.24402	H	4.4379	1.63921	0.84236	H	4.41325	1.7061	0.71287
H	0.55777	-0.19982	3.95426	H	3.27095	1.05938	2.05265	H	3.28246	1.1472	1.9663

H	1.52583	-1.51743	4.63589	H	4.80228	0.20885	1.78334	H	4.82397	0.31724	1.69563
H	-2.7933	-2.92542	0.30577	H	-0.222	-4.65787	1.85347	H	-0.24827	-4.54977	2.03302
H	-2.27541	-4.46589	-0.41456	H	1.11625	-3.54117	2.12901	H	1.06664	-3.40044	2.2963
H	-1.51705	-3.87034	1.06356	H	-0.51606	-3.06202	2.57942	H	-0.58035	-2.93436	2.6968
H	-3.35234	0.74065	-1.04885	H	-3.00657	-0.14131	1.94778	H	-3.02041	-0.01829	1.95122
H	0.91515	4.23497	1.79203	H	-0.90478	4.73752	1.27178	H	-0.87089	4.81667	1.08275
H	-0.76491	4.70148	1.48526	H	0.44989	3.68922	1.66399	H	0.47426	3.76895	1.50748
H	-0.42467	3.24998	2.41533	H	0.48575	4.6625	0.1785	H	0.51046	4.68092	-0.01619
H	-2.94313	1.8409	-3.54366	H	-4.94555	0.23906	-0.00075	H	-4.95889	0.29612	0.02068
H	-1.62955	2.95404	-3.85634	H	-4.74073	1.403	-1.2914	H	-4.76454	1.4093	-1.31545
H	-1.48553	1.26926	-4.38588	H	-4.48932	-0.31871	-1.62603	H	-4.52134	-0.32474	-1.58672
H	0.14092	3.79851	-0.52392	H	-1.52557	3.45889	-0.63906	H	-1.52054	3.47049	-0.77111
H	1.95307	2.46047	0.42108	H	0.67007	2.77261	-1.4154	H	0.67594	2.72549	-1.51943
H	2.22271	1.42751	-1.47696	H	-0.1793	1.24522	-2.72018	H	-0.22414	1.20225	-2.78652
H	1.04393	0.21373	-1.11152	H	-0.23795	0.04204	-1.47704	H	-0.26982	0.01883	-1.52432
H	0.69853	1.38248	2.27461	H	1.67907	2.12695	0.74101	H	1.64644	2.12337	0.67578
H	-0.08565	0.2996	1.13167	H	0.71772	0.65101	0.77812	H	0.68264	0.65135	0.72489
H	-1.20629	-3.15616	-2.85376	H	-1.75704	-3.99853	-0.61921	H	-1.77522	-3.96835	-0.47594
H	-2.60772	-2.46496	-2.07622	H	-2.27568	-3.34735	0.91474	H	-2.30424	-3.26731	1.03121
H	1.00607	-2.0993	-1.11341	H	0.49776	-2.16827	-1.33178	H	0.49159	-2.17813	-1.23111
H	0.67134	1.28137	-3.24731	H	-2.3597	0.36446	-2.60275	H	-2.40561	0.32118	-2.61328
H	0.51856	2.85951	-2.50794	H	-2.42818	2.04617	-2.12323	H	-2.45804	2.01824	-2.18961
H	-1.28815	-0.03971	-0.73784	H	-1.29136	-0.45468	0.5697	H	-1.30356	-0.38581	0.58225
H	-0.05743	-0.95175	-2.80818	H	-1.75574	-1.72616	-1.62066	H	-1.76752	-1.73544	-1.55461
H	-1.60317	-0.92221	-3.64417	H	-3.30773	-2.18627	-0.93571	H	-3.32274	-2.16554	-0.85848
H	2.27048	-0.55051	0.49263	H	1.92492	-0.02586	-1.43146	H	1.88459	-0.07241	-1.44606
H	0.71507	-4.12889	1.0155	H	2.52479	-3.47607	0.37365	H	2.47507	-3.45303	0.57539
H	2.10473	-3.1612	0.64084	H	2.78302	-2.46729	-1.01337	H	2.76977	-2.58459	-0.88991
H	-0.23736	-1.79799	2.06349	H	1.93492	-1.10797	1.59393	H	1.99984	-1.03405	1.6423
H	0.69527	-3.00266	2.92892	H	3.5221	-1.84387	1.50127	H	3.58534	-1.76817	1.50898
2b6				2c1				2c2			
O	2.82664	1.21167	-2.166	O	-0.65442	-0.98085	3.49945	O	3.12054	-2.642	-1.29923
O	4.27428	-0.83329	-1.1863	O	-0.87261	-1.88183	1.01191	O	1.06823	-4.29689	-0.50634
C	4.17018	0.8875	0.46903	C	0.09915	-3.59313	2.32807	C	0.84163	-3.62166	-2.78765
C	-0.12555	-3.04788	2.06976	C	1.72259	-1.15609	-2.91995	C	-1.88109	1.33099	-2.42631
O	-2.89696	-0.09473	2.30601	O	-2.96518	0.31345	-1.43831	O	-2.39422	1.45732	2.77683
O	-0.96098	2.4262	1.83264	O	1.20362	4.11151	-0.72997	O	2.17201	3.3978	0.08661
O	-2.73256	1.98056	0.51737	O	0.2386	2.13869	-1.24759	O	0.22413	2.27704	0.30771
C	-1.46604	2.40754	0.71957	C	0.89194	2.97267	-0.40728	C	1.57386	2.33748	0.20778
C	-0.01248	4.15514	-0.28765	C	1.34667	3.34389	2.01937	C	3.60793	1.00998	0.79059
C	-4.46125	0.53069	-0.11566	C	-1.86848	3.0553	-1.9765	C	-0.48325	3.65656	2.16714
C	-0.8143	2.87947	-0.55706	C	1.31773	2.30123	0.89748	C	2.22666	0.95711	0.13206
C	0.00507	1.7501	-1.20958	C	0.48372	1.03013	1.24121	C	1.30984	-0.17247	0.67973
C	-0.83559	0.51216	-1.60216	C	-1.00527	1.35315	1.46168	C	0.95114	0.00255	2.17343
C	1.23681	1.30975	-0.36385	C	1.13639	0.23954	2.40667	C	1.87569	-1.59858	0.45472
C	-1.60124	-2.70162	-0.00413	C	-0.81469	-1.23041	-3.27153	C	-3.45053	1.26357	-0.4052
C	-0.20256	-2.734	0.59749	C	0.39858	-1.74157	-2.50768	C	-2.52747	0.50481	-1.34342
C	0.87733	-2.53213	-0.1866	C	0.24302	-2.64345	-1.51744	C	-2.32087	-0.81813	-1.18356
C	-2.35886	0.54723	-1.45766	C	-1.65633	2.23939	0.38547	C	0.55932	1.41855	2.62062
C	-2.92347	0.63696	-0.02067	C	-1.20029	1.99532	-1.08235	C	-0.34487	2.21579	1.64282
C	-2.346	-0.40165	1.00719	C	-1.5602	0.55284	-1.56526	C	-1.74705	1.53901	1.50185
C	-2.65638	-1.89082	0.76373	C	-1.12966	0.25562	-3.01849	C	-2.69201	2.24884	0.5037
C	2.22785	0.41453	-1.12845	C	0.58913	-1.15465	2.79316	C	1.83221	-2.06758	-1.01899
C	2.3271	-2.57536	0.21017	C	1.29054	-3.19355	-0.58668	C	-1.28919	-1.64488	-1.90447
C	2.89027	-1.25887	0.7708	C	1.52244	-2.32372	0.66474	C	-0.68804	-2.70692	-0.9716
C	3.36305	-0.20229	-0.25875	C	0.35101	-2.22087	1.67297	C	0.74678	-3.15237	-1.32772
H	3.58484	0.67404	-2.47578	H	-0.47477	-0.41814	4.27358	H	3.10392	-3.51739	-0.86143
H	5.02251	-1.18552	-0.67155	H	-1.49261	-1.66822	1.74046	H	0.44537	-5.0104	-0.73282
H	5.05253	0.45771	0.9579	H	-0.2188	-4.33609	1.58915	H	1.79687	-4.12123	-2.98425
H	4.55042	1.63748	-0.23318	H	0.9946	-3.96542	2.83617	H	0.06301	-4.36132	-3.00756
H	3.57491	1.39395	1.23495	H	-0.71404	-3.54163	3.06117	H	0.73671	-2.79101	-3.49222

H	-0.6612	-3.97869	2.28362	H	2.57535	-1.63304	-2.4313	H	-1.06195	1.93152	-2.02299
H	0.89752	-3.1726	2.43219	H	1.86511	-1.27094	-3.99954	H	-1.48618	0.71965	-3.24279
H	-0.57526	-2.24352	2.65972	H	1.7654	-0.0906	-2.67506	H	-2.6167	2.00701	-2.87547
H	-2.62407	0.82179	2.51412	H	-3.08348	-0.65321	-1.4568	H	-3.2419	0.99801	2.64642
H	-0.68356	4.96343	0.02531	H	1.75545	2.93152	2.94571	H	4.17289	0.09025	0.6164
H	0.72128	4.02986	0.51461	H	1.98173	4.19362	1.74384	H	4.20662	1.83001	0.37805
H	0.51218	4.48543	-1.1899	H	0.3504	3.74464	2.22964	H	3.54062	1.17393	1.87015
H	-4.92922	0.60391	0.8723	H	-1.61064	4.06635	-1.64051	H	0.49898	4.13252	2.26654
H	-4.86773	1.36334	-0.70319	H	-1.51683	2.98564	-3.01132	H	-1.05125	4.28368	1.4722
H	-4.78972	-0.39888	-0.58859	H	-2.95921	2.96351	-1.97043	H	-0.97303	3.68792	3.14561
H	-1.61008	3.1836	-1.24999	H	2.35182	1.9762	0.7166	H	2.37699	0.78251	-0.94074
H	0.39581	2.17102	-2.14709	H	0.55104	0.37524	0.3704	H	0.36843	-0.12956	0.12099
H	-0.6123	0.28671	-2.65419	H	-1.58304	0.42822	1.50739	H	0.12132	-0.67683	2.4091
H	-0.47702	-0.35339	-1.0516	H	-1.1449	1.83185	2.43896	H	1.78977	-0.33049	2.7986
H	1.77612	2.20612	-0.04291	H	2.20145	0.11454	2.17078	H	2.89894	-1.65304	0.84347
H	0.88743	0.79808	0.53835	H	1.09481	0.86277	3.3084	H	1.31111	-2.30421	1.07644
H	-1.57213	-2.35451	-1.04286	H	-1.70375	-1.82866	-3.03562	H	-4.02795	0.57171	0.22089
H	-1.9549	-3.74043	-0.0551	H	-0.63325	-1.37709	-4.34391	H	-4.1862	1.81588	-1.00292
H	0.71718	-2.36818	-1.25193	H	-0.76429	-2.99628	-1.29407	H	-2.88577	-1.34304	-0.41326
H	-2.74583	-0.36812	-1.92476	H	-2.74619	2.13244	0.46207	H	0.08376	1.35377	3.60768
H	-2.74485	1.37928	-2.0603	H	-1.45494	3.28515	0.65363	H	1.48713	1.98058	2.79056
H	-1.26823	-0.25917	1.1229	H	-1.06278	-0.15239	-0.90066	H	-1.58823	0.50705	1.18042
H	-3.61236	-2.00334	0.24266	H	-1.9379	0.54667	-3.7015	H	-3.43231	2.84269	1.05451
H	-2.81958	-2.38553	1.73135	H	-0.2522	0.84703	-3.29978	H	-2.14453	2.95039	-0.13473
H	1.71146	-0.38546	-1.66128	H	1.29834	-1.5781	3.51674	H	1.7188	-1.21947	-1.69966
H	2.46922	-3.36244	0.96057	H	2.2426	-3.30659	-1.11724	H	-0.49369	-0.99977	-2.28757
H	2.91567	-2.91005	-0.65219	H	1.00726	-4.21226	-0.30014	H	-1.76019	-2.13972	-2.76136
H	2.18477	-0.81451	1.48273	H	2.40414	-2.70948	1.19333	H	-0.71388	-2.37181	0.07262
H	3.76986	-1.54827	1.36385	H	1.80613	-1.32987	0.30673	H	-1.34886	-3.58574	-0.982
2c3				2c4				2c5			
O	3.06313	-2.67134	-1.43645	O	3.47134	-1.37244	2.11924	O	0.76634	-3.95575	1.45347
O	1.02857	-4.31423	-0.58541	O	1.80912	-3.56447	2.0216	O	-1.89214	-3.4698	2.07523
C	0.73359	-3.62207	-2.8448	C	3.19112	-3.40591	0.07955	C	-1.65158	-4.34895	-0.1195
C	-2.0216	1.31088	-2.33784	C	0.27909	0.03667	-3.34319	C	-0.68076	0.26949	-3.25947
O	-2.34557	1.35882	2.88543	O	-3.85468	0.75881	-0.22368	O	-1.01275	3.7855	0.59825
O	2.09221	3.38422	0.03274	O	0.8142	3.93299	0.10893	O	3.77545	1.09051	-0.73483
O	0.16794	2.23908	0.33003	O	-0.45285	2.1833	-0.54509	O	1.55491	1.50061	-0.748
C	1.51066	2.31772	0.17792	C	0.51201	2.75243	0.2172	C	2.64957	0.81322	-0.34431
C	3.58267	1.01447	0.6769	C	1.67974	2.43828	2.40203	C	3.42104	-0.63644	1.53832
C	-0.48384	3.58585	2.23284	C	-2.49659	3.44657	-0.27804	C	1.85148	3.8402	-0.20429
C	2.17971	0.94758	0.0668	C	1.23626	1.74555	1.11051	C	2.30706	-0.40488	0.51358
C	1.30097	-0.20269	0.63134	C	0.41292	0.44765	1.34287	C	0.88451	-0.32555	1.13375
C	0.99136	-0.05063	2.13839	C	-0.93826	0.69873	2.05172	C	0.70481	0.87222	2.09422
C	1.88365	-1.61639	0.36992	C	1.1909	-0.65552	2.10614	C	0.44571	-1.62815	1.852
C	-3.51594	1.19515	-0.26226	C	-2.22434	-0.31812	-2.93222	C	-1.95401	2.23647	-2.2285
C	-2.6157	0.46294	-1.24256	C	-0.79949	-0.78813	-2.6894	C	-1.70124	0.73859	-2.25439
C	-2.38547	-0.85856	-1.10613	C	-0.55776	-1.87409	-1.92752	C	-2.36349	-0.08323	-1.41523
C	0.59932	1.3548	2.61745	C	-1.76095	1.89608	1.55353	C	1.27652	2.21683	1.62138
C	-0.35009	2.15338	1.68469	C	-1.78965	2.11112	0.01702	C	1.06602	2.55693	0.12177
C	-1.74907	1.46259	1.5878	C	-2.51386	0.92387	-0.69988	C	-0.45279	2.73786	-0.20139
C	-2.73894	2.17523	0.63626	C	-2.53072	1.02521	-2.24388	C	-0.74949	3.03135	-1.69087
C	1.79356	-2.07384	-1.10571	C	2.30872	-1.32715	1.27377	C	0.11925	-2.79729	0.89204
C	-1.37068	-1.66654	-1.87108	C	0.79144	-2.33248	-1.44258	C	-2.07574	-1.5443	-1.19232
C	-0.72424	-2.7275	-0.9687	C	0.69838	-2.90532	-0.0203	C	-2.25402	-1.91815	0.2865
C	0.69808	-3.15822	-1.38056	C	1.99149	-2.78978	0.81555	C	-1.4088	-3.11711	0.76477
H	3.73895	-1.97282	-1.37841	H	3.2947	-2.10379	2.74566	H	0.91015	-4.59287	0.73135
H	1.98791	-4.45008	-0.72635	H	1.65345	-4.48954	1.76009	H	-1.17707	-3.99359	2.49037
H	1.68507	-4.1082	-3.08809	H	3.48902	-2.81003	-0.78854	H	-2.72575	-4.53648	-0.23319
H	-0.04389	-4.37225	-3.03052	H	4.05767	-3.50456	0.74293	H	-1.21402	-4.23044	-1.11543
H	0.58662	-2.79092	-3.54122	H	2.95546	-4.42041	-0.26243	H	-1.23664	-5.256	0.33395
H	-1.20352	1.9263	-1.95538	H	0.44163	0.96945	-2.79715	H	0.33375	0.48882	-2.91741

H	-1.63873	0.71469	-3.17105	H	1.23404	-0.49303	-3.40545	H	-0.74888	-0.80378	-3.45878
H	-2.78542	1.97396	-2.75826	H	-0.00479	0.28301	-4.37204	H	-0.83786	0.77202	-4.22
H	-3.18228	0.87219	2.78443	H	-4.40054	1.47764	-0.58607	H	-1.96681	3.81916	0.40973
H	4.15571	0.10654	0.47019	H	2.33951	1.79983	2.99577	H	3.29631	-1.58861	2.061
H	4.15358	1.84933	0.25489	H	2.24172	3.35308	2.18209	H	4.40107	-0.67206	1.04911
H	3.55123	1.16288	1.76031	H	0.82763	2.72721	3.02443	H	3.46431	0.16269	2.28413
H	0.49633	4.07163	2.30034	H	-1.97175	4.28109	0.2008	H	2.91685	3.71388	0.01997
H	-1.08504	4.21536	1.56869	H	-2.50018	3.66948	-1.35003	H	1.79653	4.08443	-1.27022
H	-0.93564	3.59928	3.22981	H	-3.52964	3.45086	0.08318	H	1.48589	4.69775	0.36947
H	2.29539	0.78581	-1.01242	H	2.1406	1.47932	0.54907	H	2.32303	-1.24772	-0.18896
H	0.34033	-0.16805	0.10597	H	0.18051	0.02719	0.3582	H	0.17591	-0.18071	0.3108
H	0.17812	-0.74297	2.39334	H	-1.55107	-0.20701	1.9525	H	-0.36861	1.0004	2.28657
H	1.85475	-0.38137	2.73021	H	-0.77435	0.82544	3.12975	H	1.15179	0.63757	3.06899
H	2.92026	-1.65798	0.72273	H	1.60113	-0.24059	3.03386	H	1.21588	-1.92774	2.57185
H	1.35501	-2.33832	1.00459	H	0.47963	-1.41995	2.44215	H	-0.43321	-1.41032	2.47113
H	-4.06351	0.48652	0.37187	H	-2.94965	-1.06811	-2.5923	H	-2.83806	2.47455	-1.62363
H	-4.27805	1.74736	-0.82596	H	-2.3811	-0.21404	-4.01307	H	-2.18479	2.57388	-3.24655
H	-2.91579	-1.40165	-0.32351	H	-1.40948	-2.44104	-1.55032	H	-3.1336	0.34124	-0.77064
H	0.16174	1.27187	3.6206	H	-2.78376	1.8022	1.94048	H	0.86086	3.01351	2.25161
H	1.52596	1.92623	2.76015	H	-1.35816	2.79716	2.03469	H	2.35223	2.21534	1.84114
H	-1.59108	0.43659	1.24742	H	-1.99511	0.00634	-0.41415	H	-0.96858	1.82283	0.09892
H	-3.46711	2.74924	1.22309	H	-3.51727	1.36162	-2.58602	H	-0.96138	4.10005	-1.82154
H	-2.22435	2.89505	-0.00917	H	-1.80983	1.76785	-2.60201	H	0.11684	2.8074	-2.3224
H	1.65586	-1.22628	-1.78357	H	2.58007	-0.71414	0.41004	H	0.55969	-2.62961	-0.09539
H	-0.59682	-1.00848	-2.2762	H	1.49721	-1.49735	-1.45764	H	-1.05567	-1.77793	-1.50966
H	-1.8663	-2.15998	-2.71474	H	1.17332	-3.10484	-2.1198	H	-2.76106	-2.14258	-1.80332
H	-0.71401	-2.39858	0.07783	H	-0.13188	-2.44945	0.53301	H	-2.05776	-1.05538	0.93504
H	-1.37558	-3.61365	-0.95865	H	0.4137	-3.96473	-0.09558	H	-3.31721	-2.13992	0.45979
2c6				2c7				2c8			
O	2.55883	-1.55766	2.15688	O	3.06697	-2.64232	-1.48645	O	-0.28047	-1.32747	3.45808
O	0.51488	-2.1769	0.55172	O	1.06232	-4.326	-0.64219	O	-0.80282	-1.94483	0.9144
C	2.32778	-3.58461	-0.01472	C	0.74197	-3.61234	-2.89108	C	0.24425	-3.80821	1.936
C	-1.22173	-0.06469	-3.35125	C	-2.06439	1.28017	-2.32639	C	1.4507	-0.79282	-3.13021
O	-2.92849	-0.54061	1.46645	O	-2.36268	1.28619	2.90226	O	-2.92435	0.28299	-0.94256
O	-0.32887	4.34371	0.15591	O	2.06475	3.37924	0.06339	O	2.09651	3.00102	-1.246
O	-1.09871	2.24224	-0.12725	O	0.14632	2.22286	0.34732	O	-0.08896	2.71795	-1.02937
C	-0.1295	3.13606	0.17372	C	1.48813	2.30758	0.18891	C	1.15766	2.60196	-0.55384
C	2.04858	3.32734	1.37887	C	3.56947	1.00764	0.65311	C	1.51427	3.22981	1.84567
C	-3.011	2.45571	1.32458	C	-0.49158	3.55082	2.26369	C	-2.39571	3.16731	-1.23403
C	1.24072	2.4929	0.38005	C	2.16122	0.94188	0.05504	C	1.43965	2.08181	0.84172
C	1.16719	0.97777	0.73919	C	1.29264	-0.21791	0.61594	C	0.61019	0.85914	1.26598
C	0.44271	0.73289	2.07525	C	0.99159	-0.08102	2.12646	C	-0.79494	1.22424	1.75522
C	2.57213	0.32389	0.6541	C	1.88193	-1.62611	0.33899	C	1.39332	-0.02071	2.28118
C	-2.97601	-0.98619	-1.72636	C	-3.53193	1.13858	-0.23128	C	-1.10648	-0.75249	-3.26935
C	-1.59802	-1.13018	-2.35667	C	-2.63491	0.42224	-1.22719	C	0.14441	-1.39215	-2.68241
C	-0.79364	-2.15107	-1.99764	C	-2.3893	-0.89722	-1.09834	C	0.0354	-2.40789	-1.80144
C	-0.89817	1.46792	2.24523	C	0.59566	1.31753	2.6226	C	-1.58011	2.22639	0.90429
C	-1.78838	1.58816	0.97477	C	-0.36165	2.12233	1.70395	C	-1.38272	2.17155	-0.62173
C	-2.23414	0.1856	0.44747	C	-1.76104	1.42916	1.61035	C	-1.6081	0.75752	-1.24495
C	-3.12015	0.24772	-0.81641	C	-2.75816	2.13058	0.65698	C	-1.37228	0.68067	-2.76657
C	2.72285	-1.2102	0.7691	C	1.79025	-2.0686	-1.14105	C	0.86625	-1.44223	2.59559
C	0.62522	-2.41276	-2.42701	C	-1.38045	-1.69446	-1.88114	C	1.1339	-3.10666	-1.04463
C	1.67653	-1.69098	-1.5608	C	-0.71613	-2.75896	-0.99593	C	1.52982	-2.40373	0.26904
C	1.79665	-2.13983	-0.0827	C	0.70931	-3.16583	-1.42136	C	0.47226	-2.38055	1.40283
H	3.22417	-2.23654	2.36883	H	3.73233	-1.93451	-1.42237	H	0.00423	-0.8542	4.26014
H	0.70414	-2.21064	1.51172	H	2.02274	-4.44508	-0.78964	H	-1.33677	-1.80202	1.72411
H	3.30786	-3.67481	-0.49412	H	1.6988	-4.08178	-3.1459	H	-0.16547	-4.46297	1.15978
H	2.41873	-3.92859	1.02172	H	-0.02579	-4.37158	-3.08042	H	1.17329	-4.24938	2.31127
H	1.63808	-4.28679	-0.49463	H	0.57849	-2.77566	-3.5771	H	-0.49199	-3.81781	2.74786
H	-1.11367	0.90439	-2.85501	H	-1.24642	1.90097	-1.95201	H	1.53079	0.24642	-2.79765
H	-0.28016	-0.26921	-3.86609	H	-1.68754	0.69114	-3.16743	H	2.32306	-1.32284	-2.74067

H	-1.99624	0.02268	-4.12035	H	-2.83982	1.93831	-2.73304	H	1.5192	-0.81421	-4.22277
H	-2.92738	-1.47456	1.1907	H	-2.69167	2.15567	3.18796	H	-2.87068	-0.69085	-0.94316
H	3.07663	2.96671	1.47026	H	4.14458	0.10486	0.43018	H	1.78178	2.86973	2.84357
H	2.10727	4.37247	1.05408	H	4.13298	1.85001	0.2361	H	2.28157	3.95062	1.53961
H	1.59266	3.32719	2.37359	H	3.54696	1.14278	1.73852	H	0.58026	3.78915	1.92167
H	-2.69758	3.44142	1.68756	H	0.4853	4.04627	2.30179	H	-2.24472	4.17299	-0.82297
H	-3.63269	2.64577	0.44326	H	-1.12086	4.17654	1.62234	H	-2.25602	3.26544	-2.31619
H	-3.63172	1.99113	2.09726	H	-0.90875	3.55705	3.27544	H	-3.43233	2.87268	-1.04165
H	1.73372	2.57419	-0.59879	H	2.26747	0.79294	-1.02694	H	2.47203	1.70842	0.75021
H	0.57554	0.50417	-0.04657	H	0.32834	-0.18443	0.0971	H	0.53006	0.24811	0.37312
H	0.2411	-0.33228	2.19908	H	0.18337	-0.77984	2.37973	H	-1.40321	0.31905	1.8232
H	1.09747	1.00405	2.91279	H	1.86023	-0.41331	2.70969	H	-0.74316	1.61328	2.7804
H	3.02076	0.62071	-0.30317	H	2.91959	-1.66606	0.68894	H	2.4202	-0.14187	1.91238
H	3.2017	0.76403	1.43764	H	1.35846	-2.35667	0.96798	H	1.47414	0.53342	3.22529
H	-3.23779	-1.88449	-1.1532	H	-4.06031	0.4215	0.40961	H	-1.989	-1.37118	-3.06297
H	-3.71785	-0.92088	-2.53251	H	-4.30974	1.67982	-0.7842	H	-1.00773	-0.73368	-4.36215
H	-1.16299	-2.86399	-1.25988	H	-2.90203	-1.445	-0.30661	H	-0.96273	-2.75901	-1.53836
H	-1.46039	0.97993	3.05201	H	0.16297	1.22198	3.62677	H	-2.64339	2.0875	1.14363
H	-0.67648	2.47481	2.6235	H	1.52072	1.8912	2.76694	H	-1.33977	3.23772	1.25065
H	-1.33425	-0.38398	0.21818	H	-1.59587	0.40881	1.25768	H	-0.92807	0.06019	-0.77577
H	-4.17329	0.32006	-0.51611	H	-3.4909	2.70463	1.2375	H	-2.25913	1.05515	-3.29263
H	-2.90092	1.14123	-1.41002	H	-2.2485	2.8494	0.00658	H	-0.53052	1.31573	-3.06174
H	3.76758	-1.42869	0.50852	H	1.63538	-1.21583	-1.80856	H	1.6463	-1.94626	3.18123
H	0.76348	-2.1108	-3.47124	H	-0.61631	-1.02856	-2.29186	H	2.02405	-3.19505	-1.67799
H	0.80033	-3.49414	-2.42336	H	-1.8841	-2.18464	-2.72198	H	0.8249	-4.13938	-0.84918
H	2.65701	-1.80805	-2.04103	H	-0.70433	-2.4436	0.05471	H	2.4404	-2.88184	0.65381
H	1.44793	-0.62275	-1.61711	H	-1.35606	-3.65339	-0.99406	H	1.82678	-1.38567	0.00101
2c9				2c10				2c11			
O	-2.12585	-1.055	2.93187	O	3.24914	-1.08103	1.53015	O	3.45498	-1.52908	2.01489
O	-1.65594	-1.58422	0.34229	O	0.85097	-1.99207	0.73228	O	1.66333	-3.64953	2.01826
C	-1.71918	-3.63845	1.55706	C	2.55007	-3.44304	-0.10067	C	2.97118	-3.61	0.03499
C	2.32822	-1.24852	-2.03672	C	-1.8251	-0.52636	-2.77664	C	0.11392	-0.07747	-3.34415
O	-1.98401	1.41171	-2.32896	O	-2.41427	-0.47856	2.20754	O	-3.84897	0.92595	-0.07871
O	2.37573	3.60975	0.56405	O	-0.61782	4.31571	-0.29423	O	0.99918	3.85448	-0.02165
O	1.21814	2.07704	-0.61923	O	-1.26026	2.16034	-0.11913	O	-0.39512	2.16571	-0.56817
C	1.68432	2.60031	0.53753	C	-0.32247	3.13199	-0.20024	C	0.63	2.69901	0.13826
C	1.22018	2.61279	2.98985	C	2.07502	3.5826	0.32753	C	1.85799	2.37739	2.28909
C	-0.1258	3.67773	-1.82058	C	-2.74186	2.48156	1.75616	C	-2.36013	3.53398	-0.24164
C	1.38665	1.72515	1.75254	C	1.09989	2.5843	-0.30423	C	1.33595	1.67854	1.03056
C	0.20745	0.72934	1.52492	C	1.24361	1.13041	0.24242	C	0.46352	0.42577	1.32042
C	-1.126	1.456	1.26712	C	0.91568	1.0405	1.74485	C	-0.84365	0.75431	2.07775
C	0.15345	-0.31672	2.67091	C	2.62209	0.53144	-0.1463	C	1.22152	-0.69899	2.07265
C	0.29298	-0.61773	-3.46214	C	-3.20119	-1.3401	-0.77032	C	-2.38515	-0.29186	-2.82406
C	0.88902	-1.52042	-2.39061	C	-1.983	-1.53179	-1.6661	C	-0.97723	-0.83023	-2.6282
C	0.14136	-2.48459	-1.81542	C	-1.11787	-2.53978	-1.43206	C	-0.76156	-1.91247	-1.85337
C	-1.07043	2.6028	0.24166	C	-0.38638	1.73404	2.18276	C	-1.62622	1.98213	1.5891
C	-0.15374	2.37869	-0.99502	C	-1.58584	1.63342	1.19729	C	-1.70882	2.16996	0.05107
C	-0.6496	1.17875	-1.86696	C	-2.04065	0.15174	0.97884	C	-2.52168	1.0084	-0.61063
C	0.25743	0.87474	-3.08092	C	-3.21432	0.00273	-0.01424	C	-2.59463	1.07927	-2.15469
C	-0.81071	-1.52142	2.59057	C	2.93499	-0.9565	0.1335	C	2.26618	-1.44167	1.2055
C	0.53882	-3.39759	-0.68392	C	0.18852	-2.80317	-2.13396	C	0.57838	-2.43942	-1.41466
C	0.49641	-2.73857	0.70982	C	1.34222	-1.87874	-1.69247	C	0.51756	-2.96471	0.02731
C	-0.89687	-2.3641	1.27538	C	1.89117	-2.05726	-0.25355	C	1.84924	-2.89709	0.80404
H	-2.5511	-0.85025	2.07558	H	2.41936	-1.38125	1.95032	H	4.21259	-1.6475	1.41495
H	-2.00304	-2.18316	-0.33974	H	0.31858	-2.80265	0.66037	H	2.36339	-3.34494	2.63046
H	-1.89903	-4.21234	0.64209	H	3.37022	-3.57718	-0.81342	H	3.2759	-3.0519	-0.85552
H	-1.21496	-4.28874	2.27927	H	2.94571	-3.58556	0.91134	H	3.85435	-3.76336	0.66509
H	-2.7122	-3.39121	1.94929	H	1.82635	-4.2512	-0.24886	H	2.6495	-4.61065	-0.27674
H	2.76732	-2.02277	-1.40273	H	-1.55635	0.45449	-2.37426	H	0.34641	0.85719	-2.82736
H	2.93651	-1.19845	-2.94593	H	-1.05422	-0.80512	-3.49941	H	1.03718	-0.65728	-3.43201
H	2.42169	-0.29873	-1.5028	H	-2.76314	-0.42977	-3.33336	H	-0.19921	0.16104	-4.36633

H	-2.29644	0.58617	-2.73656	H	-1.59198	-0.7217	2.66803	H	-4.37389	1.65892	-0.44343
H	1.12904	2.02053	3.90426	H	3.11591	3.28677	0.17197	H	2.50799	1.72127	2.87425
H	2.09148	3.26465	3.12084	H	1.95832	4.57617	-0.12019	H	2.45412	3.25917	2.02801
H	0.34378	3.26309	2.91136	H	1.90469	3.69464	1.4026	H	1.04394	2.72057	2.93434
H	0.23626	4.51656	-1.21498	H	-2.42735	3.52163	1.90066	H	-1.77313	4.34948	0.1961
H	0.56505	3.59849	-2.66669	H	-3.58511	2.51842	1.05835	H	-2.39986	3.73607	-1.31704
H	-1.11773	3.93927	-2.20241	H	-3.09911	2.09812	2.71716	H	-3.37474	3.59805	0.16355
H	2.29837	1.12718	1.89045	H	1.30624	2.55195	-1.38311	H	2.20642	1.35737	0.4447
H	0.44877	0.16384	0.62167	H	0.50536	0.52775	-0.29071	H	0.17111	-0.00056	0.35436
H	-1.87095	0.74496	0.90513	H	0.83127	-0.00495	2.04555	H	-1.50301	-0.12185	2.02051
H	-1.52793	1.84899	2.20917	H	1.74524	1.44871	2.33535	H	-0.63002	0.89156	3.14573
H	1.16564	-0.72534	2.79181	H	2.75877	0.69106	-1.22418	H	1.69346	-0.2863	2.97156
H	-0.07733	0.21112	3.60464	H	3.40328	1.11852	0.35236	H	0.48984	-1.41967	2.45791
H	-0.72317	-0.94079	-3.72088	H	-3.28063	-2.15623	-0.04113	H	-3.13325	-0.9962	-2.43887
H	0.88734	-0.7335	-4.37743	H	-4.10108	-1.40347	-1.39509	H	-2.58062	-0.20189	-3.89987
H	-0.87748	-2.62345	-2.17282	H	-1.35402	-3.23646	-0.62899	H	-1.62521	-2.42629	-1.42911
H	-2.09439	2.82411	-0.08635	H	-0.67454	1.34311	3.16707	H	-2.63526	1.94679	2.01953
H	-0.74149	3.50583	0.77275	H	-0.15345	2.79294	2.35756	H	-1.15985	2.87082	2.03442
H	-0.69248	0.29617	-1.22822	H	-1.19359	-0.41033	0.58705	H	-2.04065	0.07056	-0.32469
H	-0.10347	1.43499	-3.95294	H	-4.16451	0.0715	0.53061	H	-3.57542	1.45952	-2.46605
H	1.28585	1.20553	-2.90239	H	-3.21658	0.81202	-0.75189	H	-1.85156	1.77601	-2.55698
H	-0.51555	-2.19559	3.40519	H	3.86923	-1.17308	-0.40072	H	2.52512	-0.85846	0.31673
H	1.55084	-3.77979	-0.86361	H	0.04664	-2.69941	-3.21641	H	1.33206	-1.65046	-1.48738
H	-0.10299	-4.28506	-0.70664	H	0.46216	-3.8536	-1.98712	H	0.88108	-3.25257	-2.0841
H	0.98583	-3.41699	1.42129	H	2.17382	-2.01821	-2.3961	H	-0.26238	-2.44786	0.59999
H	1.13594	-1.8528	0.66322	H	1.00217	-0.85	-1.84056	H	0.17383	-4.00902	-0.00062
2c12				2c13				2c14			
O	-0.3108	-2.08198	3.07801	O	3.1995	-2.64283	-1.13206	O	-0.11675	-2.23144	3.0646
O	-1.78789	-0.73591	1.32336	O	1.06306	-4.33069	-0.52224	O	-1.64345	-0.73181	1.44998
C	-2.80081	-2.84676	1.66993	C	1.01374	-3.58457	-2.80582	C	-2.72389	-2.82478	1.81782
C	-1.29646	0.14119	-3.35213	C	-1.77743	1.33018	-2.53696	C	-1.22758	0.05932	-3.11594
O	-0.86318	3.17969	0.83899	O	-2.62678	1.36543	2.6329	O	-0.80355	3.13094	0.89323
O	3.95537	1.07973	-1.29562	O	2.10316	3.37362	0.27779	O	3.96315	1.05722	-1.41432
O	1.72686	1.34774	-1.06575	O	0.15029	2.24339	0.34856	O	1.74713	1.34049	-1.108
C	2.8841	0.6796	-0.85975	C	1.50322	2.3089	0.33437	C	2.90456	0.65918	-0.94685
C	3.92419	-0.98919	0.67574	C	3.50559	0.98052	1.01407	C	3.97596	-1.03895	0.53333
C	1.89675	3.60325	-0.2407	C	-0.66382	3.59879	2.17787	C	1.94307	3.58233	-0.25362
C	2.68234	-0.66102	-0.15912	C	2.16534	0.9326	0.27498	C	2.71305	-0.69072	-0.26076
C	1.34534	-0.74487	0.63943	C	1.22436	-0.20965	0.75116	C	1.39752	-0.77829	0.57358
C	1.28057	0.28501	1.7827	C	0.76904	-0.05526	2.22115	C	1.37759	0.23524	1.73404
C	1.07325	-2.20599	1.08964	C	1.81681	-1.62927	0.55012	C	1.12347	-2.24687	1.00024
C	-2.14143	2.10552	-1.93445	C	-3.45945	1.21199	-0.60836	C	-2.14251	2.09842	-1.8552
C	-2.09748	0.60054	-2.16429	C	-2.47009	0.48235	-1.50163	C	-2.11818	0.58441	-2.02065
C	-2.74154	-0.2276	-1.31751	C	-2.25176	-0.83871	-1.34317	C	-2.85515	-0.19997	-1.20786
C	1.68359	1.72276	1.40734	C	0.3448	1.35265	2.66432	C	1.77957	1.67602	1.36949
C	1.25761	2.22183	-0.00391	C	-0.49832	2.16374	1.6449	C	1.31091	2.19801	-0.01855
C	-0.29965	2.3005	-0.14229	C	-1.89085	1.49021	1.41019	C	-0.25048	2.27514	-0.11267
C	-0.78329	2.72105	-1.55043	C	-2.77289	2.19886	0.35425	C	-0.77538	2.71369	-1.49988
C	-0.28531	-2.57881	1.72619	C	1.88873	-2.08359	-0.92838	C	-0.20691	-2.63125	1.68729
C	-2.75814	-1.73288	-1.31043	C	-1.17077	-1.64467	-2.0124	C	-2.87323	-1.70538	-1.15845
C	-1.60291	-2.36155	-0.50597	C	-0.62574	-2.72629	-1.06794	C	-1.66365	-2.33119	-0.43459
C	-1.60478	-2.12072	1.02449	C	0.83111	-3.16204	-1.33782	C	-1.56064	-2.11588	1.09661
H	0.44554	-2.47441	3.54855	H	3.32044	-3.31958	-0.44153	H	-0.53627	-1.34807	3.09611
H	-1.60896	-0.65569	2.28272	H	1.77512	-4.84513	-0.94152	H	-2.5604	-0.43195	1.33496
H	-3.75356	-2.4477	1.30644	H	1.9953	-4.04205	-2.97345	H	-3.69319	-2.41247	1.51895
H	-2.77269	-3.92264	1.4692	H	0.27351	-4.34418	-3.0825	H	-2.72632	-3.90012	1.61229
H	-2.81645	-2.70126	2.75618	H	0.91571	-2.73743	-3.49146	H	-2.6659	-2.68093	2.90269
H	-0.23188	0.34258	-3.20012	H	-0.99906	1.94638	-2.0798	H	-0.17408	0.18943	-2.85252
H	-1.39608	-0.92841	-3.55094	H	-1.31755	0.73381	-3.3302	H	-1.3833	-1.00256	-3.32207
H	-1.62481	0.66775	-4.2543	H	-2.49921	1.99236	-3.02722	H	-1.4224	0.59668	-4.05
H	-0.72012	4.09624	0.5496	H	-2.96253	2.24373	2.88139	H	-0.60188	4.05221	0.65706

H	3.8739	-1.99604	1.0988	H	4.08662	0.0682	0.85416	H	3.93137	-2.05154	0.94297
H	4.82907	-0.9506	0.05853	H	4.12159	1.81246	0.65426	H	4.863	-0.99675	-0.10905
H	4.06516	-0.27716	1.49456	H	3.37376	1.12122	2.09099	H	4.14526	-0.33997	1.35798
H	2.9896	3.54285	-0.18159	H	0.31124	4.07979	2.31597	H	3.03723	3.51965	-0.23348
H	1.67084	3.98052	-1.24371	H	-1.21165	4.22858	1.46931	H	1.68321	3.97696	-1.24161
H	1.56127	4.34101	0.49473	H	-1.18533	3.62002	3.13978	H	1.6346	4.30772	0.50548
H	2.62465	-1.39424	-0.97563	H	2.37907	0.77603	-0.78976	H	2.62749	-1.4109	-1.08639
H	0.54739	-0.49561	-0.06427	H	0.32046	-0.16689	0.13391	H	0.58249	-0.51236	-0.10397
H	0.26591	0.32972	2.18323	H	-0.07179	-0.7402	2.39317	H	0.3763	0.2891	2.16412
H	1.90905	-0.04459	2.61949	H	1.56672	-0.39528	2.89437	H	2.02643	-0.11321	2.54715
H	1.20776	-2.85558	0.21463	H	2.80818	-1.67778	1.01484	H	1.21112	-2.87207	0.1016
H	1.85303	-2.49398	1.8054	H	1.21524	-2.34824	1.11973	H	1.92972	-2.56325	1.67365
H	-2.8798	2.36357	-1.16469	H	-4.06063	0.50301	-0.02521	H	-2.87268	2.39665	-1.0923
H	-2.49574	2.58027	-2.85845	H	-4.16829	1.75952	-1.24192	H	-2.49288	2.53905	-2.79728
H	-3.28407	0.2168	-0.48141	H	-2.84992	-1.37833	-0.60804	H	-3.49493	0.28749	-0.47345
H	1.29738	2.40343	2.17712	H	-0.19224	1.27147	3.6181	H	1.42111	2.34694	2.16102
H	2.77648	1.78903	1.49366	H	1.25846	1.91369	2.90138	H	2.87495	1.73462	1.42233
H	-0.70124	1.31104	0.07723	H	-1.70568	0.46487	1.08278	H	-0.6411	1.28013	0.09995
H	-0.88938	3.81243	-1.59117	H	-3.55647	2.78338	0.85206	H	-0.88463	3.8053	-1.52334
H	-0.05103	2.45391	-2.31885	H	-2.18877	2.90934	-0.2403	H	-0.06586	2.45758	-2.29338
H	-0.30518	-3.67423	1.8004	H	1.81744	-1.22665	-1.60388	H	-0.23646	-3.72851	1.69814
H	-2.71843	-2.11441	-2.33677	H	-0.35596	-0.98605	-2.32608	H	-2.9176	-2.1019	-2.17992
H	-3.72501	-2.07192	-0.92257	H	-1.58163	-2.12067	-2.90995	H	-3.80908	-2.03113	-0.69152
H	-1.60367	-3.44416	-0.68901	H	-0.72404	-2.41711	-0.02003	H	-1.67538	-3.41194	-0.62921
H	-0.67521	-1.98961	-0.95046	H	-1.27899	-3.60795	-1.14322	H	-0.76456	-1.95379	-0.92986
2d1				2d2				2d3			
O	0.35667	3.60503	-0.55819	O	0.34641	3.66603	-0.46902	O	0.75685	3.17422	-1.61424
O	1.12292	3.36523	2.10847	O	1.13537	3.31133	2.20797	O	-0.69485	4.05371	0.62692
C	2.9619	3.42023	0.59646	C	2.9449	3.45654	0.69228	C	1.65272	4.15258	0.94308
C	2.23046	-2.41828	1.95056	C	2.41625	-2.4295	1.93303	C	-0.11592	-0.91019	3.66043
O	-1.13276	-3.33666	0.3688	O	-1.15314	-3.30528	0.40167	O	-1.14068	-3.24186	0.91498
O	-2.70875	-0.65703	1.66222	O	-2.70142	-0.58157	1.643	O	-3.09377	-0.89768	-0.50387
O	-3.11221	-1.45633	-0.40193	O	-3.09968	-1.42088	-0.40807	O	-1.86869	-2.36471	-1.69075
C	-2.89162	-0.44545	0.47157	C	-2.88629	-0.39497	0.44829	C	-2.33735	-1.11927	-1.43868
C	-3.6322	1.90647	0.78778	C	-3.64742	1.95754	0.70845	C	-3.0121	0.89129	-2.7354
C	-2.61625	-3.00915	-2.1007	C	-2.60473	-2.98672	-2.0948	C	-0.32283	-4.14105	-1.71282
C	-2.96584	0.91929	-0.17684	C	-2.95644	0.95713	-0.22434	C	-1.87701	-0.1006	-2.4585
C	-1.59165	1.4006	-0.69033	C	-1.56932	1.42887	-0.70984	C	-0.55282	0.58421	-2.05452
C	-0.99943	0.53629	-1.82681	C	-0.95782	0.5482	-1.82408	C	0.65999	-0.37037	-1.97418
C	-0.54254	1.62033	0.4383	C	-0.54637	1.64698	0.44345	C	-0.66036	1.44887	-0.76412
C	1.66476	-2.99276	-0.45572	C	1.65249	-3.02934	-0.40928	C	1.36496	-2.31404	2.14947
C	2.15042	-1.93353	0.52436	C	2.19699	-1.95131	0.51889	C	0.96185	-0.91853	2.60462
C	2.52003	-0.6966	0.12841	C	2.49823	-0.70573	0.0932	C	1.56373	0.19535	2.13508
C	-1.79855	-0.66572	-2.33362	C	-1.75485	-0.65488	-2.33433	C	0.4599	-1.84982	-2.30697
C	-2.03903	-1.80936	-1.32162	C	-2.02155	-1.7889	-1.31707	C	-0.4764	-2.64533	-1.36863
C	-0.78537	-2.24003	-0.4889	C	-0.78054	-2.22801	-0.4699	C	-0.25401	-2.40274	0.16196
C	0.44124	-2.64255	-1.31663	C	0.44274	-2.66648	-1.28542	C	1.17658	-2.6409	0.66012
C	0.75065	2.32825	-0.02788	C	0.74674	2.37278	0.01164	C	0.58757	2.3148	-0.47165
C	3.2017	0.34061	0.98348	C	3.20766	0.35905	0.89044	C	1.38411	1.59158	2.6759
C	2.2936	1.27368	1.79656	C	2.31789	1.24307	1.77472	C	0.21667	2.41377	2.11355
C	1.79005	2.56347	1.10636	C	1.78508	2.55889	1.15798	C	0.45362	3.19944	0.80375
H	0.15768	4.15026	0.23008	H	0.25223	4.22471	0.32909	H	1.6784	3.48759	-1.61436
H	1.7564	3.53088	2.8291	H	0.61776	2.6914	2.75243	H	-0.68307	4.33236	-0.31026
H	3.42656	2.99166	-0.29638	H	3.41308	3.08451	-0.22364	H	1.59769	4.71071	1.88503
H	2.63583	4.4384	0.35484	H	2.60772	4.48294	0.50696	H	2.60706	3.61861	0.9109
H	3.7304	3.52747	1.37072	H	3.71371	3.53144	1.47013	H	1.65754	4.90914	0.15008
H	2.51091	-1.63618	2.66021	H	2.75374	-1.64246	2.61163	H	-0.99862	-1.44851	3.29902
H	1.25522	-2.80071	2.27083	H	1.47957	-2.82151	2.34419	H	0.24348	-1.40352	4.56938
H	2.96468	-3.2262	2.03328	H	3.16411	-3.22901	1.94835	H	-0.44846	0.094	3.93402
H	-1.84867	-3.01345	0.95196	H	-1.87889	-2.96802	0.96401	H	-2.04878	-2.98145	0.66036
H	-3.67994	2.90903	0.35061	H	-3.68586	2.95313	0.25474	H	-3.8789	0.36742	-3.15472

H	-4.65769	1.58738	1.00728	H	-4.67753	1.63973	0.90722	H	-3.35807	1.39625	-1.82814
H	-3.11081	1.97197	1.74776	H	-3.15084	2.03915	1.6802	H	-2.69942	1.65237	-3.45757
H	-1.91556	-3.3908	-2.84929	H	-1.90356	-3.37738	-2.83826	H	-0.55567	-4.31835	-2.7699
H	-2.88994	-3.83003	-1.42856	H	-2.88901	-3.80259	-1.42097	H	0.69138	-4.50557	-1.52429
H	-3.54092	-2.72344	-2.6169	H	-3.52401	-2.69493	-2.61717	H	-1.02683	-4.75827	-1.14366
H	-3.65768	0.84656	-1.02633	H	-3.62807	0.86859	-1.08812	H	-1.72705	-0.62879	-3.40947
H	-1.78172	2.38891	-1.13355	H	-1.74211	2.41461	-1.16535	H	-0.33051	1.28123	-2.87562
H	-0.83408	1.19947	-2.68757	H	-0.77125	1.20091	-2.68848	H	1.4239	0.01504	-2.66408
H	-0.00022	0.18726	-1.55774	H	0.03368	0.19684	-1.53063	H	1.12769	-0.30748	-0.98946
H	-1.00018	2.23629	1.21939	H	-1.02737	2.25151	1.21906	H	-1.51827	2.12074	-0.87149
H	-0.29687	0.6586	0.89429	H	-0.29981	0.68368	0.89559	H	-0.86941	0.79751	0.08765
H	2.50049	-3.24463	-1.12228	H	2.47369	-3.35287	-1.06269	H	2.42167	-2.46469	2.40828
H	1.44782	-3.92131	0.0887	H	1.39031	-3.91785	0.18013	H	0.8208	-3.06233	2.74074
H	2.4396	-0.44131	-0.92705	H	2.33035	-0.45495	-0.95251	H	2.34274	0.08718	1.38251
H	-1.25081	-1.06779	-3.19666	H	-1.19395	-1.06802	-3.18356	H	1.45486	-2.31488	-2.28973
H	-2.75866	-0.31139	-2.72967	H	-2.70512	-0.29901	-2.75204	H	0.10901	-1.93563	-3.3432
H	-0.50143	-1.42681	0.18152	H	-0.48577	-1.41049	0.19078	H	-0.54215	-1.37925	0.40897
H	0.71634	-1.85553	-2.02453	H	0.73472	-1.89737	-2.00617	H	1.89946	-2.06273	0.07762
H	0.20577	-3.53282	-1.9123	H	0.1922	-3.56282	-1.86573	H	1.44052	-3.69729	0.5288
H	1.22908	1.78358	-0.84525	H	1.22629	1.86111	-0.82663	H	1.48646	1.69775	-0.39359
H	3.86844	-0.17658	1.68623	H	3.95185	-0.128	1.53409	H	1.2533	1.52347	3.76422
H	3.88651	0.91197	0.34818	H	3.80916	0.95968	0.2008	H	2.33236	2.12747	2.56149
H	1.45291	0.7163	2.2266	H	1.49323	0.65682	2.19819	H	-0.68438	1.79448	2.03075
H	2.87236	1.58694	2.67833	H	2.91461	1.52369	2.6555	H	-0.05046	3.15268	2.8842
2d4				2d5				2d6			
O	0.38557	3.63107	-0.39338	O	0.40548	3.57301	-0.56117	O	0.41868	3.64504	-0.69708
O	1.14899	3.31426	2.26745	O	1.13911	3.36893	2.15447	O	1.12572	3.55454	1.96528
C	2.97622	3.39414	0.75081	C	3.00453	3.35946	0.64068	C	3.01536	3.46081	0.51311
C	2.14214	-2.44674	1.89841	C	2.07471	-2.42736	2.00832	C	2.22547	-2.51686	1.58743
O	-1.0531	-3.37837	0.24641	O	-1.11261	-3.3646	0.3118	O	-1.5601	-3.24988	0.93703
O	-2.64728	-0.79895	1.68401	O	-2.72511	-0.70372	1.62112	O	-2.77221	-0.3562	1.64388
O	-3.07233	-1.4858	-0.41656	O	-3.08738	-1.47193	-0.46287	O	-3.15395	-1.38751	-0.31973
C	-2.85232	-0.52367	0.51075	C	-2.88322	-0.47486	0.4303	C	-2.90996	-0.29021	0.43123
C	-3.61751	1.7965	0.96775	C	-3.62352	1.87343	0.77294	C	-3.60375	2.09573	0.35439
C	-2.55984	-2.95108	-2.18786	C	-2.55444	-3.00332	-2.17113	C	-2.70262	-3.16732	-1.77411
C	-2.95429	0.87385	-0.06066	C	-2.94502	0.90033	-0.19763	C	-2.88772	0.97062	-0.39722
C	-1.59794	1.40711	-0.57495	C	-1.56441	1.39055	-0.6855	C	-1.45518	1.32628	-0.83642
C	-0.99834	0.60213	-1.74981	C	-0.95403	0.54228	-1.82413	C	-0.77897	0.25029	-1.7204
C	-0.5382	1.61548	0.54588	C	-0.53001	1.60322	0.45884	C	-0.51467	1.70606	0.34635
C	1.73895	-2.91428	-0.56487	C	1.70353	-2.96762	-0.44491	C	1.54872	-2.50038	-0.88548
C	2.16798	-1.90508	0.49038	C	2.1256	-1.92947	0.58515	C	2.1316	-1.73984	0.29864
C	2.58402	-0.66093	0.17049	C	2.55573	-0.69872	0.23341	C	2.55869	-0.4688	0.14137
C	-1.78403	-0.58439	-2.31075	C	-1.74287	-0.65385	-2.35943	C	-1.57652	-0.97549	-2.17352
C	-1.99845	-1.78077	-1.35478	C	-1.99637	-1.81136	-1.36667	C	-2.04364	-1.95816	-1.07473
C	-0.73299	-2.22929	-0.5495	C	-0.75713	-2.24814	-0.516	C	-0.93447	-2.42249	-0.06624
C	0.49968	-2.55828	-1.40014	C	0.49138	-2.62245	-1.32335	C	0.23135	-3.25321	-0.63611
C	0.75872	2.32785	0.09267	C	0.7745	2.30611	0.01178	C	0.7956	2.38781	-0.10571
C	3.22666	0.32503	1.1135	C	3.19538	0.31014	1.1532	C	3.20634	0.4258	1.16073
C	2.29008	1.23943	1.91423	C	2.26027	1.27498	1.89478	C	2.26706	1.42736	1.84879
C	1.79453	2.53673	1.23726	C	1.79723	2.55398	1.15766	C	1.80817	2.67002	1.04519
H	0.04088	3.52125	-1.29612	H	-0.11628	4.03729	0.11822	H	0.21913	4.22549	0.06592
H	0.69757	4.04161	1.79389	H	1.25327	4.30213	1.90513	H	1.74146	3.7659	2.68933
H	3.44995	2.9719	-0.14038	H	3.49321	2.86815	-0.20585	H	3.50878	2.94379	-0.31542
H	2.6593	4.41629	0.51369	H	2.71034	4.36445	0.31692	H	2.71919	4.45751	0.16679
H	3.7343	3.49114	1.53665	H	3.74331	3.50336	1.43743	H	3.75222	3.62618	1.30766
H	2.3797	-1.69683	2.65674	H	2.2983	-1.65389	2.74703	H	2.73764	-1.97727	2.3864
H	1.14372	-2.82981	2.13472	H	1.07192	-2.80273	2.23867	H	1.22823	-2.76369	1.9636
H	2.86112	-3.26617	1.99992	H	2.79118	-3.24335	2.14786	H	2.77506	-3.44962	1.42266
H	-1.75564	-3.09778	0.86684	H	-1.83601	-3.05654	0.89379	H	-2.23318	-2.69153	1.3766
H	-3.68886	2.82032	0.58666	H	-3.66632	2.88221	0.34973	H	-3.5573	3.03369	-0.20821
H	-4.63358	1.4494	1.18851	H	-4.65146	1.55113	0.97563	H	-4.66058	1.84163	0.49646

H	-3.0782	1.81809	1.9199	H	-3.11324	1.92515	1.73974	H	-3.18678	2.26802	1.35141
H	-1.8547	-3.28746	-2.95379	H	-1.83636	-3.37593	-2.90772	H	-2.02152	-3.68204	-2.45714
H	-2.82074	-3.80598	-1.55416	H	-2.84201	-3.83173	-1.51421	H	-3.08	-3.8937	-1.04577
H	-3.48935	-2.65515	-2.68937	H	-3.46779	-2.71325	-2.70473	H	-3.57503	-2.84479	-2.35601
H	-3.65997	0.8359	-0.90127	H	-3.62524	0.84145	-1.05762	H	-3.49678	0.79431	-1.29399
H	-1.8229	2.40499	-0.97772	H	-1.75202	2.38318	-1.12013	H	-1.55713	2.22253	-1.46485
H	-0.83988	1.30578	-2.57914	H	-0.77577	1.21705	-2.67323	H	-0.41854	0.75565	-2.62735
H	0.00437	0.25192	-1.49622	H	0.04102	0.19071	-1.54382	H	0.12429	-0.10734	-1.23018
H	-0.99234	2.22066	1.33754	H	-0.99476	2.21839	1.23642	H	-1.03949	2.39658	1.01408
H	-0.29265	0.64556	0.98529	H	-0.29517	0.63871	0.9149	H	-0.29606	0.80339	0.92353
H	2.58433	-3.07572	-1.24722	H	2.56256	-3.16728	-1.09954	H	1.41757	-1.83826	-1.74873
H	1.5703	-3.88983	-0.08998	H	1.50627	-3.92281	0.05947	H	2.29894	-3.23876	-1.19967
H	2.58168	-0.36417	-0.87681	H	2.56698	-0.43275	-0.82243	H	2.49325	-0.03059	-0.85352
H	-1.2377	-0.93476	-3.1969	H	-1.18108	-1.04329	-3.21924	H	-0.94075	-1.52451	-2.88106
H	-2.75269	-0.2248	-2.68044	H	-2.69753	-0.2958	-2.76524	H	-2.44299	-0.62897	-2.75124
H	-0.4647	-1.44605	0.16247	H	-0.49606	-1.44507	0.17565	H	-0.54825	-1.56223	0.48593
H	0.75137	-1.72941	-2.06787	H	0.77194	-1.82049	-2.01201	H	-0.05759	-3.73253	-1.57659
H	0.2855	-3.42303	-2.03982	H	0.28046	-3.50534	-1.93888	H	0.44809	-4.0888	0.04364
H	1.24766	1.80085	-0.73094	H	1.27059	1.74271	-0.78211	H	1.28413	1.81661	-0.89646
H	3.84092	-0.23603	1.83049	H	3.77989	-0.23379	1.90726	H	3.68498	-0.17754	1.93946
H	3.95927	0.91129	0.54874	H	3.95494	0.85761	0.58537	H	4.0401	0.94941	0.67973
H	1.44367	0.66728	2.31216	H	1.398	0.73381	2.30227	H	1.40013	0.90258	2.26779
H	2.84132	1.54283	2.8172	H	2.80158	1.60748	2.79363	H	2.79978	1.7949	2.73852
2d7											
O	0.41442	3.66867	-0.67383								
O	1.14258	3.55181	2.00459								
C	3.00742	3.48891	0.54481								
C	2.26832	-2.48906	1.58604								
O	-1.53098	-3.23608	0.95978								
O	-2.74105	-0.30896	1.64254								
O	-3.1261	-1.38125	-0.30024								
C	-2.88839	-0.26908	0.42955								
C	-3.60516	2.1084	0.30788								
C	-2.67822	-3.16713	-1.74923								
C	-2.8743	0.97723	-0.41986								
C	-1.43904	1.33462	-0.84783								
C	-0.7559	0.25295	-1.7192								
C	-0.511	1.71946	0.34386								
C	1.57137	-2.49145	-0.88136								
C	2.16038	-1.72077	0.29296								
C	2.58276	-0.44958	0.12452								
C	-1.54954	-0.97934	-2.16323								
C	-2.01759	-1.95445	-1.05753								
C	-0.90721	-2.41352	-0.04836								
C	0.25845	-3.24618	-0.61555								
C	0.79833	2.40919	-0.09425								
C	3.23712	0.45071	1.13495								
C	2.28952	1.42809	1.84432								
C	1.80856	2.67714	1.06352								
H	0.33144	4.28807	0.07984								
H	0.56508	3.0067	2.5688								
H	3.5054	2.99234	-0.29345								
H	2.7037	4.4894	0.21625								
H	3.74263	3.64734	1.3424								
H	2.78313	-1.94115	2.37769								
H	1.27523	-2.73876	1.97126								
H	2.82185	-3.4198	1.42321								
H	-2.2192	-2.68427	1.38312								
H	-3.56149	3.03747	-0.2695								
H	-4.66107	1.84802	0.44534								
H	-3.19821	2.30051	1.30537								

H	-1.99859	-3.68535	-2.4311								
H	-3.05417	-3.88979	-1.01648								
H	-3.5518	-2.84733	-2.33085								
H	-3.47412	0.78194	-1.31863								
H	-1.53547	2.22758	-1.48166								
H	-0.39429	0.75084	-2.62977								
H	0.14671	-0.09682	-1.22244								
H	-1.0478	2.4074	1.00448								
H	-0.29112	0.8192	0.92429								
H	1.43192	-1.83605	-1.74837								
H	2.32143	-3.22999	-1.19553								
H	2.50921	-0.01585	-0.87191								
H	-0.9107	-1.53297	-2.86439								
H	-2.41503	-0.64062	-2.74695								
H	-0.52132	-1.55024	0.49937								
H	-0.03463	-3.73783	-1.54838								
H	0.48204	-4.07322	0.0724								
H	1.29044	1.84706	-0.88964								
H	3.74047	-0.14942	1.9006								
H	4.05254	0.9907	0.6413								
H	1.43232	0.88217	2.25691								
H	2.8187	1.79237	2.7376								

Detailed DP4+ probability for compound **2**. Isomer 1 is 1R*,3R*,4R*,11S*,12R*,15S*, isomer 2 is 1R*,3R*,4R*,11S*,12S*,15S*, isomer 3 is 1R*,3R*,4S*,11S*,12R*,15S*, isomer 4 is 1R*, 3R*, 4S*, 11S*, 12S*, 15S*.

Functional	Solvent?		Basis Set		Type of Data	
B3LYP	PCM		6-311+G(d,p)		Unscaled Shifts	
	Isomer 1	Isomer 2	Isomer 3	Isomer 4	Isomer 5	Isomer 6
sDP4+ (H data)	99.94%	0.00%	0.06%	0.00%	–	–
sDP4+ (C data)	52.80%	0.00%	47.20%	0.00%	–	–
sDP4+ (all data)	99.94%	0.00%	0.06%	0.00%	–	–
uDP4+ (H data)	0.00%	0.00%	0.12%	99.88%	–	–
uDP4+ (C data)	0.00%	0.00%	100.00%	0.00%	–	–
uDP4+ (all data)	0.00%	0.00%	99.94%	0.06%	–	–
DP4+ (H data)	0.00%	0.00%	77.33%	22.67%	–	–
DP4+ (C data)	0.00%	0.00%	100.00%	0.00%	–	–
DP4+ (all data)	0.00%	0.00%	100.00%	0.00%	–	–

Table S8.Calculation process of 3

Important thermodynamic parameters (a.u.) of the optimized **3** with simplified structures at B3LYP/6-31+G(d,p) level in the gas phase.

NO.	E+ZPE	G	P%	NO.	E+ZPE	G	P%	NO.	E+ZPE	G	P%
3a1	-1233.728784	-1233.276462	19.85%	3b4	-1233.722741	-1233.268241	2.67%	3c2	-1233.71105	-1233.25533	13.68%
3a2	-1233.729987	-1233.277714	74.81%	3b5	-1233.720657	-1233.268189	2.53%	3d1	-1233.708239	-1233.257477	75.33%
3a3	-1233.726856	-1233.27406	1.56%	3b6	-1233.722884	-1233.267944	1.95%	3d2	-1233.708552	-1233.256104	17.58%
3a4	-1233.727743	-1233.274894	3.77%	3b7	-1233.723289	-1233.26983	14.28%	3d3	-1233.707748	-1233.253887	1.68%
3b1	-1233.725489	-1233.271219	62.69%	3b8	-1233.71955	-1233.268768	4.67%	3d4	-1233.707881	-1233.254393	2.87%
3b2	-1233.720657	-1233.268197	2.55%	3b9	-1233.71955	-1233.268759	4.62%				
3b3	-1233.722883	-1233.267945	1.95%	3c1	-1233.712146	-1233.257069	86.32%				

Optimized Z-Matrixes of **3** with simplified structures in the Gas Phase (Å) at B3LYP/6-31+G(d,p) level.

3a1				3a2				3a3			
C	-0.99222	-0.74113	3.2104	C	-2.32812	0.69355	2.56073	C	-2.82192	-0.09195	2.01443
O	2.3856	1.74744	4.41331	O	-1.30786	3.01881	-1.69586	O	-1.63762	3.82477	3.38057
C	3.32313	-0.35918	5.11331	C	-3.30396	4.04612	-0.82549	C	-1.14405	2.49394	5.32728
C	2.48568	0.53991	4.25673	C	-2.08306	3.18	-0.76429	C	-1.29325	2.75507	3.85992
O	1.86509	-0.18899	3.28898	O	-1.9743	2.62895	0.47347	O	-0.98675	1.63057	3.15732

C	-2.39797	0.65473	-4.43624	C	3.46719	-2.79835	-2.0042	C	3.49073	-2.81362	-2.48876
C	-3.86106	-1.62318	0.69346	C	-3.4735	-3.41647	-0.22293	C	-3.78076	-2.43269	-0.85023
O	-3.01292	1.06688	0.37015	O	-4.30911	-0.94836	0.93074	O	-3.40014	0.17525	-1.92616
O	-0.9794	0.38366	1.02878	O	-2.29167	-0.15869	0.23659	O	-2.0013	0.54158	-0.20984
C	-2.24024	0.15039	0.62122	C	-3.17859	-1.13353	0.51175	C	-2.73135	-0.27219	-1.00113
C	-2.60478	-1.25743	0.38259	C	-2.61046	-2.4532	0.14291	C	-2.63665	-1.73064	-0.76579
O	-1.50588	-1.55941	-3.89755	O	2.00972	-1.87805	-0.26256	O	2.00935	-1.62748	-0.95353
C	-1.61807	-2.17819	-0.28717	C	-1.12155	-2.68139	0.29097	C	-1.28683	-2.37439	-0.5685
C	-0.89355	-1.56538	-1.5102	C	-0.3362	-2.21587	-0.95974	C	-0.31142	-1.9396	-1.6833
C	-1.81909	-1.07577	-2.58655	C	1.08262	-2.70656	-0.96825	C	1.0672	-2.51389	-1.56023
C	-1.35631	-0.14013	-3.70235	C	2.24296	-1.9946	-1.67297	C	2.27139	-1.95641	-2.32577
C	0.08295	0.30323	-3.86217	C	2.05338	-0.71221	-2.45477	C	2.10146	-0.8319	-3.32747
C	0.3688	1.73319	-3.38375	C	2.92232	0.4537	-1.95216	C	2.54535	0.55613	-2.83727
C	-0.63202	-2.71571	0.75964	C	-0.61865	-2.09891	1.63195	C	-0.79449	-2.16225	0.86862
C	0.38205	-1.69806	1.28755	C	-0.32911	-0.59483	1.65944	C	-0.45563	-0.72451	1.25064
C	-0.1768	-0.41091	1.9426	C	-1.48034	0.3837	1.31649	C	-1.59273	0.31242	1.16965
C	1.00712	0.54441	2.37689	C	-0.88393	1.69416	0.69525	C	-1.08878	1.71665	1.70765
C	1.88949	1.08128	1.22336	C	0.18001	2.41649	1.54548	C	0.2944	2.20024	1.21458
C	0.39711	1.87548	-1.8847	C	2.13757	1.48202	-1.17706	C	1.46986	1.27956	-2.06665
C	1.27571	2.23805	0.41772	C	1.35835	2.92759	0.69645	C	0.38936	2.60465	-0.26152
C	1.46438	2.08094	-1.08401	C	2.2364	1.82146	0.12492	C	1.61394	2.03004	-0.9564
C	2.88913	2.20388	-1.55783	C	3.19402	1.19411	1.10239	C	2.9465	2.38066	-0.34986
H	-1.38129	0.17616	3.66736	H	-2.68799	-0.22645	3.03448	H	-3.31259	-0.9977	1.65422
H	-1.86341	-1.36559	2.998	H	-1.75354	1.24456	3.31215	H	-2.55653	-0.27181	3.06137
H	-0.388	-1.26882	3.95556	H	-3.21599	1.28136	2.30401	H	-3.5839	0.69554	1.97935
H	3.80941	0.23323	5.8936	H	-4.19265	3.4504	-0.60184	H	-1.82213	1.6927	5.63176
H	2.68986	-1.11095	5.59109	H	-3.20674	4.87368	-0.11847	H	-0.10815	2.2287	5.55284
H	4.09558	-0.83621	4.50498	H	-3.40707	4.45673	-1.834	H	-1.40208	3.39894	5.88468
H	-2.07395	0.8689	-5.45974	H	4.37309	-2.19335	-1.89982	H	4.3961	-2.19816	-2.47835
H	-3.3513	0.11795	-4.49486	H	3.57212	-3.66963	-1.34868	H	3.57899	-3.55426	-1.68664
H	-2.58499	1.60359	-3.92429	H	3.41255	-3.16065	-3.03573	H	3.45018	-3.3531	-3.44034
H	-4.56674	-0.92086	1.13192	H	-4.54329	-3.22854	-0.28176	H	-4.73324	-1.94497	-1.0462
H	-4.22288	-2.6295	0.50823	H	-3.13682	-4.41736	-0.47159	H	-3.79684	-3.51173	-0.73749
H	-2.17623	-3.04694	-0.66669	H	-0.9732	-3.76882	0.37381	H	-1.40689	-3.46243	-0.68036
H	-0.26221	-0.7322	-1.19657	H	-0.82183	-2.61737	-1.85849	H	-0.72093	-2.25771	-2.6511
H	-0.21563	-2.32179	-1.92675	H	-0.3784	-1.12846	-1.0565	H	-0.25643	-0.85033	-1.72085
H	-2.86337	-1.04275	-2.30386	H	1.15225	-3.78036	-0.8398	H	1.07937	-3.56219	-1.29042
H	0.32387	0.25159	-4.93223	H	1.00091	-0.41786	-2.50727	H	1.07259	-0.79941	-3.70419
H	0.77005	-0.39627	-3.3706	H	2.33819	-0.92418	-3.49407	H	2.71073	-1.07776	-4.20772
H	1.31747	2.05545	-3.82286	H	3.79465	0.09253	-1.40218	H	3.47121	0.45992	-2.26187
H	-0.37982	2.42946	-3.77871	H	3.32538	0.97847	-2.82865	H	2.77025	1.16915	-3.71886
H	-1.18056	-3.15776	1.60122	H	-1.32344	-2.34622	2.43681	H	-1.53616	-2.56028	1.57321
H	-0.0695	-3.54517	0.31021	H	0.31434	-2.61479	1.8961	H	0.10351	-2.77339	1.02748
H	1.02213	-2.22266	2.00864	H	0.07278	-0.35297	2.65188	H	-0.05789	-0.74325	2.27345
H	1.03306	-1.43882	0.44816	H	0.49739	-0.41237	0.96917	H	0.36488	-0.40723	0.60212
H	0.56513	1.40626	2.89641	H	-0.46682	1.40072	-0.27348	H	-1.84075	2.46764	1.42879
H	2.83547	1.44612	1.64684	H	-0.27349	3.28079	2.04728	H	0.57898	3.08099	1.80786
H	2.1975	0.25675	0.57343	H	0.57455	1.77624	2.33865	H	1.0515	1.45772	1.49105
H	-0.59015	1.82718	-1.42244	H	1.40705	2.01778	-1.78799	H	0.46421	1.15677	-2.46848
H	1.75637	3.17632	0.72641	H	1.98545	3.5811	1.31653	H	0.44793	3.69949	-0.32416
H	0.21888	2.38396	0.65992	H	0.97467	3.56981	-0.10617	H	-0.52239	2.33919	-0.80318
H	3.52792	1.47329	-1.05151	H	3.79529	0.39211	0.66994	H	3.77513	2.25599	-1.0524
H	3.00986	2.03455	-2.62938	H	3.88509	1.95105	1.48763	H	2.95734	3.43204	-0.04254
H	3.27158	3.20556	-1.33565	H	2.65137	0.76244	1.94884	H	3.14819	1.76091	0.52873
3a4				3b1				3b2			
C	-2.82256	0.14084	2.13664	C	1.72657	-2.79203	-1.43026	C	2.55526	-2.22638	0.52716
O	-1.44186	4.33886	2.63356	O	1.70512	2.05552	-2.61376	O	1.1656	-3.64493	-3.4737
C	-0.3519	3.38543	4.55881	C	3.17859	1.03039	-4.22041	C	-0.06776	-5.19137	-2.09799
C	-0.93038	3.37189	3.17724	C	2.14377	1.04231	-3.13798	C	0.61687	-3.89672	-2.41187
O	-0.79417	2.13244	2.6325	O	1.76689	-0.23038	-2.84462	O	0.52679	-3.05592	-1.3451
C	3.10865	-2.88088	-2.73983	C	-2.09915	-1.29368	3.72125	C	-2.47366	2.63252	2.42423

C	-3.9033	-2.72292	-0.26979	C	1.8304	2.04176	0.87053	C	3.93625	0.61358	2.53011
O	-3.9861	-0.23123	-1.621	O	4.25573	0.65031	0.14058	O	3.42828	1.92312	0.0148
O	-2.33427	0.44233	-0.25917	O	2.81494	-1.0008	-0.36786	O	2.27323	0.04634	-0.38422
C	-3.12301	-0.50939	-0.79663	C	3.14207	0.1531	0.25089	C	2.83942	0.93895	0.44698
C	-2.84176	-1.91089	-0.42092	C	2.10614	0.75008	1.12215	C	2.75313	0.68901	1.89237
O	1.7824	-1.79766	-0.99663	O	-1.89686	1.12158	3.35341	O	-0.49787	3.78758	1.57224
C	-1.41661	-2.394	-0.33126	C	1.58242	-0.10338	2.25781	C	1.42812	0.61934	2.6016
C	-0.59804	-1.97889	-1.57703	C	0.45343	0.51997	3.11947	C	0.64053	1.94923	2.64528
C	0.77055	-2.59134	-1.61659	C	-0.88126	0.67578	2.44002	C	-0.03892	2.43356	1.39176
C	1.9401	-2.00039	-2.40963	C	-2.13999	-0.15367	2.74074	C	-1.52571	2.83547	1.27846
C	1.76285	-0.76894	-3.27355	C	-3.38368	-0.13866	1.86747	C	-2.14699	3.12942	-0.07531
C	2.51505	0.47324	-2.77231	C	-3.42976	0.8564	0.70105	C	-3.16048	2.08937	-0.56171
C	-0.80537	-1.98439	1.01532	C	1.25197	-1.55445	1.84644	C	0.59574	-0.62397	2.27296
C	-0.53962	-0.49099	1.18804	C	0.55666	-1.67272	0.49434	C	0.35069	-0.92981	0.8005
C	-1.74694	0.45733	1.07662	C	1.46773	-1.43639	-0.73939	C	1.58093	-1.20184	-0.08331
C	-1.2992	1.95729	1.29039	C	0.8377	-0.41938	-1.74637	C	1.1424	-1.7585	-1.49735
C	-0.29087	2.50162	0.24067	C	-0.5016	-0.88478	-2.34955	C	0.23162	-0.80616	-2.30842
C	1.73924	1.24485	-1.73145	C	-2.77523	0.32925	-0.55114	C	-2.513	0.76891	-0.85678
C	1.20366	2.2596	0.54633	C	-1.44764	0.28601	-2.66173	C	-1.28257	-0.98642	-2.10291
C	2.09102	1.52891	-0.46053	C	-1.99449	0.99266	-1.42894	C	-2.00083	0.34774	-2.03049
C	3.41581	1.12513	0.14507	C	-1.64671	2.45478	-1.33154	C	-2.04277	1.12763	-3.31832
H	-3.24631	-0.8596	2.02408	H	2.40926	-2.68209	-2.28045	H	2.06426	-3.18696	0.71463
H	-2.4217	0.21077	3.15317	H	2.21609	-3.48699	-0.73716	H	3.40365	-2.39696	-0.14585
H	-3.66574	0.83578	2.04848	H	0.80437	-3.26159	-1.78592	H	2.99073	-1.89587	1.47144
H	-0.8798	2.665	5.18868	H	3.49517	2.05602	-4.4305	H	-1.12831	-5.01163	-1.90483
H	0.71466	3.1509	4.51566	H	4.05055	0.45868	-3.89267	H	0.02218	-5.86599	-2.95417
H	-0.47299	4.38202	4.99289	H	2.75618	0.6007	-5.13206	H	0.40722	-5.66169	-1.23333
H	4.04085	-2.3073	-2.72402	H	-1.43344	-1.10649	4.56784	H	-2.03793	2.92843	3.38372
H	3.21136	-3.70625	-2.0271	H	-3.0954	-1.47849	4.13786	H	-3.38116	3.23095	2.29112
H	2.98645	-3.31347	-3.73789	H	-1.76994	-2.20754	3.21739	H	-2.76338	1.57923	2.49056
H	-4.92086	-2.35648	-0.38738	H	2.30825	2.57177	0.04717	H	4.8788	0.71243	1.99542
H	-3.78631	-3.77579	-0.035	H	1.14257	2.62782	1.46843	H	3.99918	0.47065	3.60399
H	-1.43471	-3.49417	-0.32878	H	2.43018	-0.1901	2.95578	H	1.68012	0.47042	3.66536
H	-1.13046	-2.30675	-2.47935	H	0.35475	-0.07819	4.03179	H	-0.09927	1.86054	3.44908
H	-0.53184	-0.88856	-1.64094	H	0.77378	1.50964	3.47055	H	1.33227	2.73808	2.97292
H	0.76565	-3.65989	-1.44051	H	-0.81041	1.20886	1.50297	H	0.52399	2.25436	0.48654
H	0.70399	-0.53813	-3.43173	H	-3.56166	-1.15527	1.49332	H	-2.65939	4.09793	-0.00696
H	2.14708	-1.01	-4.27378	H	-4.23185	0.09013	2.52727	H	-1.35958	3.25508	-0.83018
H	3.51686	0.19134	-2.44231	H	-4.48599	1.01689	0.44805	H	-3.94636	1.93257	0.18635
H	2.6553	1.15274	-3.6229	H	-3.03631	1.82675	1.01203	H	-3.67686	2.47544	-1.4468
H	-1.44218	-2.33995	1.83553	H	2.17276	-2.15272	1.83184	H	1.06955	-1.49935	2.73643
H	0.1473	-2.51398	1.14552	H	0.62496	-2.01964	2.6162	H	-0.37956	-0.53354	2.76994
H	-0.05684	-0.35318	2.16372	H	0.10091	-2.66969	0.43003	H	-0.31489	-1.80085	0.77156
H	0.19876	-0.21795	0.43536	H	-0.29419	-0.98761	0.4694	H	-0.19807	-0.08548	0.38388
H	-2.21477	2.5565	1.17132	H	0.69423	0.52191	-1.2137	H	2.07319	-1.86513	-2.07484
H	-0.59573	2.17119	-0.75311	H	-0.31404	-1.42393	-3.28703	H	0.54395	0.23148	-2.14215
H	-0.41837	3.59321	0.22047	H	-1.01563	-1.58685	-1.68531	H	0.42504	-0.99303	-3.37397
H	0.78062	1.59106	-2.10834	H	-3.01468	-0.7112	-0.77232	H	-2.4386	0.1164	0.01185
H	1.29411	1.72254	1.4977	H	-2.29848	-0.09302	-3.24324	H	-1.49455	-1.57055	-1.20164
H	1.66926	3.23967	0.72106	H	-0.93786	1.00164	-3.31805	H	-1.69994	-1.57273	-2.93158
H	4.11791	0.68803	-0.56593	H	-0.57262	2.58129	-1.17248	H	-1.03018	1.32749	-3.68251
H	3.91024	2.00148	0.57843	H	-1.92137	2.96841	-2.25919	H	-2.57677	0.55493	-4.08375
H	3.25487	0.39138	0.94214	H	-2.16386	2.97133	-0.52045	H	-2.54364	2.09359	-3.22316
3b3				3b4				3b5			
C	2.46148	-1.87064	-1.59511	C	2.65188	-2.01248	1.01012	C	2.97675	-2.23304	0.31266
O	0.43871	2.48338	-2.58113	O	2.59658	1.82987	-2.35337	O	1.19051	-3.44435	-3.57257
C	2.37957	2.23735	-3.98456	C	4.94243	1.56906	-1.86135	C	0.26593	-5.21267	-2.22206
C	1.30646	1.76355	-3.05276	C	3.49176	1.20238	-1.80556	C	0.7938	-3.83604	-2.48567
O	1.4433	0.42829	-2.83741	O	3.32622	0.07361	-1.06474	O	0.75198	-3.09818	-1.34226
C	-2.5178	0.55045	3.58995	C	-3.8851	-0.60694	2.38481	C	-2.48825	3.20892	2.31324
C	3.48363	0.3801	2.95795	C	0.50444	2.86471	0.46713	C	3.11681	2.47627	2.10344

O	4.29872	-0.35041	0.25354	O	3.10178	2.46433	1.63508	O	4.36207	0.46531	0.48449
O	2.08584	0.07165	-0.10327	O	2.54719	0.28598	1.48909	O	2.22262	0.09161	-0.1792
C	3.15496	-0.22955	0.65727	C	2.24513	1.6016	1.4918	C	3.15936	0.56129	0.67003
C	2.74147	-0.30767	2.07405	C	0.80556	1.93128	1.38687	C	2.54171	1.30068	1.79232
O	-0.49346	1.80629	3.06542	O	-3.74644	1.48011	1.10953	O	-0.33189	3.54564	1.19363
C	1.59962	-1.21681	2.45817	C	-0.12486	1.27358	2.38894	C	1.39973	0.71293	2.5875
C	0.36157	-0.5064	3.06044	C	-1.62447	1.65573	2.28092	C	0.08841	1.53776	2.56883
C	-0.22324	0.63685	2.27765	C	-2.35915	1.12246	1.07687	C	-0.33632	2.11221	1.24854
C	-1.64619	1.1441	2.52238	C	-3.48368	0.06645	1.10158	C	-1.62596	2.92674	1.11768
C	-2.33393	1.95213	1.44231	C	-4.05483	-0.58359	-0.15094	C	-2.28183	3.07681	-0.2375
C	-3.37777	1.16459	0.63818	C	-3.2142	-0.50032	-1.42913	C	-3.28913	1.96099	-0.54926
C	1.25234	-2.2987	1.40369	C	0.06276	-0.25771	2.5055	C	1.16241	-0.81106	2.4395
C	0.57925	-1.82871	0.10995	C	0.3952	-0.92283	1.17292	C	0.72089	-1.32651	1.06586
C	1.43297	-0.9868	-0.86076	C	1.85234	-0.7345	0.69277	C	1.80158	-1.28592	-0.03374
C	0.53562	-0.19764	-1.88174	C	1.9569	-0.36888	-0.82873	C	1.24587	-1.74458	-1.43464
C	-0.4866	-0.99575	-2.71645	C	1.70138	-1.47814	-1.86757	C	0.18687	-0.811	-2.05742
C	-2.77207	0.03387	-0.14973	C	-1.96986	-1.34664	-1.37141	C	-2.59571	0.63699	-0.70695
C	-1.85781	-1.25887	-2.06775	C	0.3187	-2.13412	-1.92344	C	-1.28024	-1.16807	-1.77428
C	-2.53748	-0.03533	-1.47635	C	-0.86549	-1.20927	-2.13464	C	-2.14347	0.07721	-1.84611
C	-2.90227	1.03787	-2.46933	C	-0.75073	-0.21861	-3.26374	C	-2.35219	0.63641	-3.22843
H	3.16301	-1.26803	-2.18211	H	2.23519	-2.89703	0.51928	H	2.64203	-3.27098	0.41169
H	3.07279	-2.44943	-0.89513	H	3.7	-1.9085	0.70736	H	3.74579	-2.19319	-0.46765
H	1.97527	-2.58708	-2.26368	H	2.66375	-2.20077	2.09049	H	3.47997	-1.96336	1.2455
H	2.29071	3.31906	-4.11986	H	5.50871	0.76878	-2.34411	H	-0.78056	-5.15445	-1.91259
H	3.36258	2.02179	-3.55803	H	5.06241	2.48488	-2.4471	H	0.32759	-5.80629	-3.13861
H	2.26654	1.75175	-4.95698	H	5.31709	1.75125	-0.85092	H	0.86915	-5.69983	-1.4518
H	-1.95004	0.28947	4.4893	H	-3.81602	0.05251	3.2539	H	-1.89957	3.41773	3.21209
H	-3.29685	1.25856	3.89092	H	-4.92443	-0.9493	2.33118	H	-3.12502	4.0816	2.13465
H	-3.00249	-0.35962	3.22322	H	-3.24901	-1.47961	2.56192	H	-3.13436	2.35128	2.52473
H	4.32334	0.99346	2.63965	H	1.27236	3.29691	-0.17354	H	3.95975	2.86747	1.53829
H	3.27038	0.35092	4.02144	H	-0.49745	3.25128	0.32223	H	2.75602	3.08442	2.92666
H	1.99307	-1.81751	3.29535	H	0.18152	1.67905	3.3661	H	1.73377	0.78487	3.63687
H	-0.41806	-1.26076	3.22408	H	-2.11908	1.33635	3.20447	H	-0.73093	0.92205	2.9602
H	0.63485	-0.13943	4.0593	H	-1.71526	2.75	2.27869	H	0.20745	2.36013	3.28788
H	0.21816	0.80585	1.30868	H	-1.83786	1.31617	0.1506	H	0.01619	1.57146	0.38252
H	-2.83381	2.80519	1.91833	H	-4.26157	-1.64156	0.06006	H	-2.80326	4.04151	-0.27008
H	-1.59159	2.37735	0.75405	H	-5.03313	-0.12732	-0.35293	H	-1.51959	3.11703	-1.02701
H	-4.13633	0.74455	1.30945	H	-3.83026	-0.87577	-2.25581	H	-4.03113	1.87422	0.25303
H	-3.91739	1.86094	-0.00978	H	-2.97506	0.54464	-1.6508	H	-3.85369	2.22408	-1.44873
H	2.1542	-2.87469	1.16167	H	0.85203	-0.48183	3.23489	H	2.06001	-1.34679	2.76938
H	0.57209	-3.01799	1.88054	H	-0.84611	-0.71111	2.91844	H	0.38557	-1.10003	3.16105
H	0.21765	-2.71923	-0.41903	H	0.15273	-1.99045	1.23857	H	0.36486	-2.35737	1.18549
H	-0.30623	-1.26364	0.39875	H	-0.28447	-0.51428	0.42855	H	-0.14208	-0.73009	0.76427
H	0.02176	0.59041	-1.32052	H	1.27357	0.46333	-1.02151	H	2.11137	-1.71893	-2.1139
H	-0.663	-0.45833	-3.65854	H	1.92624	-1.06622	-2.86165	H	0.4044	0.22954	-1.78995
H	-0.05431	-1.95365	-3.02745	H	2.45398	-2.26794	-1.74265	H	0.31396	-0.8527	-3.14854
H	-2.49874	-0.81969	0.46878	H	-1.98771	-2.14439	-0.62904	H	-2.38249	0.13811	0.23774
H	-1.75955	-2.04644	-1.3136	H	0.18098	-2.74257	-1.02233	H	-1.388	-1.64612	-0.79529
H	-2.52375	-1.68458	-2.83019	H	0.32479	-2.84984	-2.7568	H	-1.63838	-1.90559	-2.50316
H	-2.0137	1.40243	-2.99141	H	-0.38111	-0.7177	-4.16592	H	-1.39587	0.92645	-3.67484
H	-3.59509	0.63377	-3.21497	H	-0.05865	0.58719	-3.00411	H	-2.81737	-0.11934	-3.87007
H	-3.38234	1.90742	-2.01685	H	-1.70958	0.23676	-3.52684	H	-2.99787	1.51677	-3.24746
3b6				3b7				3b8			
C	1.98346	-2.21474	0.98164	C	2.09034	-2.26991	0.73192	C	2.6103	-2.00275	-1.66367
O	-0.56478	-2.24238	-3.26436	O	-0.95042	-4.43376	2.71966	O	-1.28077	-3.64852	-3.20627
C	1.24876	-3.81416	-3.46208	C	0.05812	-3.43468	4.66635	C	-0.87357	-5.51186	-1.73286
C	0.34103	-2.86948	-2.7358	C	-0.37098	-3.48833	3.23229	C	-0.78677	-4.08419	-2.17713
O	0.66835	-2.84081	-1.41635	O	-0.01941	-2.33459	2.60125	O	-0.08494	-3.35657	-1.26516
C	-1.41101	3.66705	1.34633	C	0.33592	4.19573	0.35127	C	-1.7814	1.89111	3.4345
C	4.56866	0.66801	1.09976	C	3.51757	-0.83472	-2.32646	C	3.61654	2.15394	0.72955
O	3.64885	0.47472	-1.57887	O	0.7794	-1.1346	-3.14982	O	3.17099	0.97873	-1.80292

O	1.79676	-0.42302	-0.69663	O	0.47458	-1.48798	-0.95487	O	1.35132	-0.00806	-0.8743
C	2.9424	0.26849	-0.59923	C	1.16853	-0.99146	-1.99604	C	2.50403	0.6858	-0.8247
C	3.28597	0.81552	0.71964	C	2.43836	-0.30612	-1.71904	C	2.78525	1.10955	0.56576
O	0.02296	3.87483	-0.60675	O	-0.07674	3.59337	-1.99023	O	0.18729	3.06388	2.57057
C	2.26673	1.56554	1.53365	C	2.48861	0.95343	-0.89863	C	2.2012	0.33315	1.72563
C	1.65078	2.81664	0.8584	C	1.8392	2.16954	-1.59309	C	0.74588	0.66205	2.14766
C	0.53915	2.62155	-0.13585	C	0.34797	2.30717	-1.50491	C	0.18667	1.96134	1.66439
C	-0.93499	3.0412	0.06745	C	-0.40633	3.32115	-0.62163	C	-1.10648	2.51525	2.24974
C	-1.99535	2.5383	-0.9031	C	-1.91012	3.30663	-0.40819	C	-1.96129	3.36581	1.34031
C	-3.18753	1.79172	-0.28469	C	-2.71356	2.22541	-1.1366	C	-3.06428	2.56477	0.62696
C	1.28816	0.62246	2.23044	C	2.15366	0.77881	0.58882	C	2.43758	-1.19226	1.55278
C	0.40569	-0.24501	1.34326	C	0.88797	0.00331	0.95382	C	1.49569	-1.95527	0.60107
C	1.05693	-1.18175	0.32091	C	0.80422	-1.45682	0.46055	C	1.40985	-1.45064	-0.86531
C	-0.04468	-1.88904	-0.56443	C	-0.36388	-2.22708	1.19075	C	0.09008	-1.95258	-1.56997
C	-1.17048	-2.69095	0.12265	C	-1.77844	-1.61179	1.1325	C	-1.1745	-1.16711	-1.13306
C	-2.80293	0.4996	0.37942	C	-2.61137	0.88414	-0.45862	C	-2.54923	1.35851	-0.1194
C	-2.37134	-1.92357	0.711	C	-2.4566	-1.63008	-0.24634	C	-1.42212	0.08026	-2.00754
C	-2.88721	-0.75505	-0.10879	C	-2.56255	-0.33326	-1.03555	C	-2.02217	1.32409	-1.35982
C	-3.47701	-1.11174	-1.44797	C	-2.69462	-0.55863	-2.52276	C	-1.91782	2.54492	-2.24348
H	2.52576	-2.80444	0.23468	H	1.97063	-3.30144	0.38011	H	2.6189	-1.60053	-2.68299
H	2.75412	-1.74291	1.59681	H	2.97005	-1.8848	0.21646	H	3.56546	-1.72523	-1.20519
H	1.42487	-2.89745	1.62895	H	2.3376	-2.29475	1.79836	H	2.58175	-3.09575	-1.72802
H	1.10046	-4.83051	-3.08905	H	-0.44264	-2.60531	5.17206	H	-1.42719	-6.09034	-2.47794
H	1.0138	-3.7938	-4.5301	H	-0.22255	-4.36738	5.16392	H	0.13071	-5.93321	-1.64154
H	2.28853	-3.5035	-3.33119	H	1.1437	-3.32233	4.72414	H	-1.40459	-5.57138	-0.77957
H	-1.61828	2.89611	2.09416	H	-0.23174	5.10986	0.55725	H	-1.07347	1.63534	4.22928
H	-0.67873	4.35983	1.77199	H	0.47487	3.66457	1.29789	H	-2.51551	2.57898	3.867
H	-2.32621	4.24449	1.17876	H	1.31663	4.50996	-0.01466	H	-2.30387	0.97637	3.13796
H	5.29381	0.15604	0.47042	H	3.44116	-1.72075	-2.95344	H	4.04353	2.68419	-0.11861
H	4.93615	1.06633	2.03987	H	4.50278	-0.38993	-2.23099	H	3.88379	2.51769	1.71665
H	2.83723	2.00816	2.36739	H	3.55386	1.23957	-0.88055	H	2.81422	0.60711	2.59917
H	1.31096	3.48248	1.65984	H	2.32368	3.0709	-1.20114	H	0.05374	-0.10819	1.80388
H	2.456	3.36533	0.35093	H	2.10618	2.15105	-2.65895	H	0.70526	0.60855	3.24296
H	0.78135	1.91027	-0.9133	H	-0.16935	1.45114	-1.91448	H	0.35647	2.14733	0.61161
H	-2.39331	3.40908	-1.44061	H	-2.11282	3.24901	0.66943	H	-2.42737	4.16448	1.93019
H	-1.52718	1.89788	-1.66188	H	-2.29427	4.28339	-0.73234	H	-1.33601	3.86721	0.59042
H	-3.69127	2.42172	0.4577	H	-3.77094	2.51727	-1.10681	H	-3.79486	2.21273	1.36652
H	-3.93544	1.61941	-1.06556	H	-2.42237	2.19204	-2.18924	H	-3.62737	3.226	-0.0408
H	1.84749	-0.03193	2.91246	H	3.00555	0.28923	1.07978	H	3.47606	-1.36852	1.24298
H	0.63326	1.21983	2.87924	H	2.09773	1.77012	1.0562	H	2.34331	-1.65939	2.54267
H	-0.20279	-0.84012	2.0297	H	0.81731	0.01962	2.04876	H	1.80071	-3.01079	0.6023
H	-0.26203	0.42127	0.8061	H	0.02772	0.56442	0.58178	H	0.49849	-1.96465	1.05172
H	-0.4822	-1.11683	-1.20832	H	-0.41797	-3.23032	0.74483	H	0.21909	-1.83303	-2.65471
H	-0.74527	-3.33515	0.90173	H	-2.41513	-2.21189	1.79859	H	-2.06265	-1.80872	-1.18895
H	-1.57716	-3.39789	-0.6147	H	-1.77047	-0.62964	1.61119	H	-1.07269	-0.90768	-0.07758
H	-2.40595	0.63526	1.38451	H	-2.62112	0.95006	0.62606	H	-2.61075	0.44114	0.46083
H	-2.12728	-1.59889	1.728	H	-3.48838	-1.98353	-0.10913	H	-2.07216	-0.21422	-2.84227
H	-3.19749	-2.63464	0.84797	H	-1.97375	-2.39571	-0.86641	H	-0.47595	0.38642	-2.47009
H	-2.79671	-1.74729	-2.01912	H	-1.7949	-1.0347	-2.91917	H	-2.34486	3.44589	-1.79747
H	-4.41325	-1.66131	-1.30555	H	-3.54481	-1.22044	-2.72214	H	-0.86709	2.76041	-2.46533
H	-3.69058	-0.24076	-2.07146	H	-2.86569	0.35584	-3.09376	H	-2.44241	2.36842	-3.18846
3b9				3c1				3c2			
C	2.66582	-1.71147	-1.72619	C	0.40265	-1.66233	-1.14968	C	0.23138	-1.46388	-1.74873
O	0.67979	-4.04635	-3.26071	O	-1.63019	-4.78395	2.02669	O	-1.57569	-4.89865	1.46614
C	0.1947	-5.66191	-1.54154	C	-0.57495	-4.21331	4.11692	C	-0.5972	-4.25188	3.57102
C	0.3482	-4.28429	-2.10945	C	-0.99694	-3.98235	2.69854	C	-0.95539	-4.07853	2.12707
O	0.07689	-3.35764	-1.15176	O	-0.58695	-2.75281	2.28877	O	-0.50471	-2.87568	1.68128
C	-1.95386	1.73083	3.45462	C	0.2943	4.85972	-1.43276	C	0.04082	4.87025	-0.74196
C	3.33184	2.51973	0.56127	C	4.21171	-0.97473	-1.09268	C	4.03166	-0.78303	-2.11735
O	2.81423	1.33795	-1.94718	O	3.78474	-2.77961	1.01309	O	3.86027	-2.97522	-0.37797
O	1.20351	0.12421	-0.9119	O	1.59897	-2.32137	0.84311	O	1.69587	-2.47669	-0.0863

C	2.26184	0.95596	-0.92899	C	2.90097	-2.02856	0.60848	C	2.95377	-2.15207	-0.47621
C	2.58245	1.40901	0.44415	C	3.25507	-0.7998	-0.16014	C	3.23152	-0.79438	-1.03337
O	-0.14343	3.10782	2.54961	O	0.31277	2.77952	-0.1505	O	0.49377	2.67396	0.23736
C	2.12273	0.59992	1.63711	C	2.68632	0.56766	0.17706	C	2.76532	0.48247	-0.35008
C	0.6532	0.77726	2.0986	C	2.08774	1.31238	-1.0511	C	2.01895	1.45428	-1.3129
C	-0.04735	2.01446	1.63791	C	1.60385	2.70365	-0.75796	C	1.565	2.75212	-0.7049
C	-1.37813	2.42421	2.25601	C	0.39907	3.3622	-1.45585	C	0.17771	3.39006	-0.95804
C	-2.34028	3.17584	1.36747	C	-0.43241	2.6545	-2.50941	C	-0.9106	2.73769	-1.79495
C	-3.37158	2.2602	0.68522	C	-1.88774	2.3737	-2.08059	C	-2.23286	2.48144	-1.04113
C	2.51709	-0.89366	1.47968	C	1.71959	0.4824	1.35683	C	1.97385	0.15142	0.91317
C	1.62998	-1.7705	0.575	C	0.39105	-0.19368	1.03914	C	0.58985	-0.41855	0.63008
C	1.43379	-1.30241	-0.89254	C	0.41293	-1.59231	0.38765	C	0.47827	-1.68243	-0.24181
C	0.15292	-1.96291	-1.53728	C	-0.8459	-2.39951	0.89471	C	-0.71425	-2.57684	0.27797
C	-1.17655	-1.29963	-1.09221	C	-2.23267	-1.69887	0.89186	C	-2.15696	-2.03078	0.05507
C	-2.75778	1.10076	-0.06157	C	-2.13527	0.92241	-1.73288	C	-2.41931	1.04542	-0.59335
C	-1.56178	-0.08732	-1.96697	C	-2.79666	-1.10934	-0.41437	C	-2.67916	-0.92502	0.99937
C	-2.25656	1.10282	-1.31312	C	-2.64918	0.40137	-0.59743	C	-2.4976	0.55608	0.66318
C	-2.28373	2.32049	-2.20706	C	-3.17363	1.24996	0.53395	C	-2.41913	1.42322	1.89871
H	2.57594	-1.35047	-2.7569	H	0.15661	-2.6759	-1.48946	H	-0.05476	-2.40832	-2.22855
H	3.59034	-1.2859	-1.32148	H	-0.31405	-0.98249	-1.59389	H	-0.56257	-0.74027	-1.93803
H	2.79419	-2.79807	-1.75248	H	1.36586	-1.46066	-1.61551	H	1.10992	-1.14132	-2.30475
H	0.39042	-6.40057	-2.32408	H	-1.06554	-3.48896	4.77175	H	0.48697	-4.18888	3.69435
H	0.91399	-5.81068	-0.73232	H	-0.87061	-5.22067	4.42365	H	-1.09816	-3.48789	4.17069
H	-0.82701	-5.80223	-1.17975	H	0.51199	-4.13199	4.19828	H	-0.92777	-5.23754	3.9108
H	-1.2038	1.55255	4.23176	H	0.70355	5.2808	-2.35655	H	0.12366	5.39949	-1.69657
H	-2.74708	2.33589	3.9061	H	-0.75053	5.17385	-1.34379	H	-0.92865	5.11355	-0.29655
H	-2.38192	0.7652	3.16835	H	0.84694	5.29417	-0.5928	H	0.81918	5.25823	-0.07611
H	3.66911	3.07737	-0.30949	H	4.64824	-1.95311	-1.28434	H	4.40141	-1.70493	-2.56221
H	3.61951	2.9116	1.53169	H	4.60681	-0.14858	-1.6736	H	4.364	0.14095	-2.57764
H	2.72765	0.95662	2.48593	H	3.53435	1.17265	0.53174	H	3.67209	1.00385	-0.00944
H	0.03379	-0.05646	1.76447	H	2.8507	1.40167	-1.83352	H	2.68855	1.72073	-2.14022
H	0.64596	0.71398	3.19405	H	1.28331	0.72456	-1.48738	H	1.18337	0.94297	-1.78146
H	0.07652	2.22151	0.5827	H	2.38732	3.34371	-0.37014	H	2.39828	3.39905	-0.4566
H	-2.87195	3.92464	1.96723	H	0.0572	1.74278	-2.86458	H	-0.56782	1.81968	-2.27733
H	-1.79076	3.73584	0.59994	H	-0.46453	3.31151	-3.38913	H	-1.12624	3.42497	-2.62415
H	-4.0479	1.84639	1.44425	H	-2.18573	3.05436	-1.27921	H	-2.36204	3.19772	-0.22728
H	-4.01199	2.85451	0.02414	H	-2.54111	2.59822	-2.9337	H	-3.05751	2.68312	-1.73826
H	3.55701	-0.96337	1.1345	H	2.19808	-0.03385	2.20046	H	2.53696	-0.54085	1.55351
H	2.50834	-1.34925	2.47933	H	1.51536	1.48988	1.74043	H	1.86635	1.05348	1.52766
H	2.04816	-2.78648	0.57721	H	-0.22014	0.47115	0.43258	H	-0.01922	0.35429	0.17685
H	0.65687	-1.87933	1.0642	H	-0.12148	-0.24967	2.00814	H	0.15508	-0.59668	1.6197
H	0.20825	-1.86556	-2.62915	H	-0.91082	-3.32731	0.31203	H	-0.6349	-3.52491	-0.27426
H	-1.99119	-2.03375	-1.14689	H	-2.95322	-2.4633	1.21871	H	-2.32253	-1.77758	-0.99507
H	-1.09414	-1.0347	-0.03614	H	-2.27018	-0.96361	1.70383	H	-2.82313	-2.88707	0.23438
H	-2.72133	0.18756	0.52727	H	-1.86387	0.2358	-2.53434	H	-2.53073	0.3552	-1.42645
H	-2.20743	-0.44845	-2.77882	H	-3.87928	-1.30188	-0.41699	H	-2.27255	-1.11406	2.00039
H	-0.66328	0.29799	-2.46373	H	-2.41295	-1.66697	-1.27528	H	-3.76428	-1.06922	1.10261
H	-2.781	3.18269	-1.75718	H	-2.4327	1.32702	1.33503	H	-2.28087	2.48491	1.68996
H	-1.26218	2.62867	-2.45435	H	-3.44171	2.26126	0.21859	H	-3.33995	1.32207	2.48299
H	-2.81101	2.08887	-3.13854	H	-4.08712	0.80861	0.94751	H	-1.57729	1.11047	2.52566
3d1				3d2				3d3			
C	-0.82809	0.61369	1.79385	C	-0.66362	0.73233	1.81807	C	-0.77762	0.27599	1.56089
O	0.43056	-3.70873	2.85138	O	-0.89321	-4.28042	2.40625	O	0.41237	-3.34264	3.41283
C	-1.23071	-3.33871	4.55526	C	-1.40898	-3.35966	4.57156	C	-1.85648	-3.2084	4.21072
C	-0.57186	-3.12115	3.22775	C	-1.07229	-3.30059	3.11318	C	-0.74911	-3.01628	3.22057
O	-1.24252	-2.16535	2.53092	O	-1.00095	-2.00331	2.7083	O	-1.23065	-2.41517	2.10035
C	-0.80535	3.5337	-0.59465	C	-0.72144	3.55423	-0.65396	C	-0.99043	3.53537	-0.55593
C	3.86725	-0.62643	-0.98	C	3.86897	-0.68818	-1.13202	C	4.21719	-0.13922	-0.87436
O	1.36568	-1.91754	-1.24072	O	1.34483	-1.96057	-1.20248	O	2.02354	-1.87911	-1.26543
O	0.44312	-0.16041	-0.10783	O	0.49942	-0.13801	-0.11276	O	0.71888	-0.31704	-0.23755
C	1.47652	-0.8683	-0.63018	C	1.49636	-0.88324	-0.65297	C	1.90089	-0.82728	-0.66097

C	2.76153	-0.16358	-0.37245	C	2.80211	-0.18978	-0.48448	C	3.01527	0.10097	-0.3228
O	1.24829	3.59825	-1.85383	O	1.26585	3.54056	-2.01487	O	1.04033	3.61355	-1.85475
C	2.76049	1.0015	0.58813	C	2.86515	1.00474	0.43703	C	2.75656	1.22749	0.65077
C	2.10001	2.32847	0.08454	C	2.20246	2.32609	-0.07968	C	1.98447	2.47457	0.1058
C	1.47354	2.30265	-1.28541	C	1.50339	2.26275	-1.41302	C	1.31085	2.34833	-1.23581
C	0.06	2.82464	-1.59578	C	0.0821	2.79415	-1.669	C	-0.12111	2.81924	-1.5482
C	-0.60385	2.33089	-2.86716	C	-0.65204	2.2645	-2.88655	C	-0.77967	2.2687	-2.80007
C	-1.97868	1.66868	-2.69743	C	-2.02873	1.6359	-2.6263	C	-2.18133	1.67057	-2.63043
C	2.28718	0.5757	2.01315	C	2.4554	0.63114	1.89673	C	2.23257	0.72141	2.02981
C	1.30514	-0.61629	2.12782	C	1.45676	-0.53564	2.09762	C	1.4704	-0.62118	2.06937
C	0.04259	-0.526	1.2253	C	0.15588	-0.44464	1.24984	C	0.28145	-0.73067	1.07336
C	-0.77446	-1.86067	1.18558	C	-0.67655	-1.76787	1.30896	C	-0.29882	-2.18463	1.01246
C	-2.05491	-1.86732	0.30376	C	-2.01076	-1.78316	0.51156	C	-1.0132	-2.63371	-0.28823
C	-2.02255	0.44576	-1.81642	C	-2.05402	0.44752	-1.69831	C	-2.31045	0.51786	-1.67016
C	-1.86558	-2.02697	-1.217	C	-1.90874	-2.00347	-1.01047	C	-2.21973	-1.82634	-0.79586
C	-1.74094	-0.82849	-2.15378	C	-1.80573	-0.84318	-1.99745	C	-1.93274	-0.76423	-1.85208
C	-1.27974	-1.22309	-3.53771	C	-1.41098	-1.29789	-3.38343	C	-1.29116	-1.262	-3.12229
H	-1.11816	0.42277	2.83259	H	-1.59957	0.88557	1.2819	H	-1.00582	0.14465	2.62446
H	-0.30548	1.57252	1.76653	H	-0.9078	0.58454	2.87546	H	-0.44682	1.30659	1.41368
H	-1.741	0.76695	1.21882	H	-0.12371	1.67791	1.73248	H	-1.71667	0.1829	1.02333
H	-2.27253	-3.63475	4.40852	H	-2.38411	-2.89796	4.74559	H	-2.64634	-3.82169	3.76967
H	-0.71474	-4.14212	5.08873	H	-1.45556	-4.40472	4.8909	H	-1.46829	-3.72527	5.09301
H	-1.17003	-2.42534	5.15218	H	-0.63357	-2.85288	5.15156	H	-2.24986	-2.23645	4.5187
H	-0.21954	4.08619	0.14676	H	-0.09096	4.11494	0.04325	H	-0.40779	4.12729	0.15709
H	-1.44983	4.26226	-1.09836	H	-1.37085	4.28212	-1.15233	H	-1.66315	4.23011	-1.07016
H	-1.4439	2.82882	-0.05873	H	-1.35195	2.88245	-0.06852	H	-1.59852	2.8303	0.01537
H	3.83197	-1.48002	-1.65335	H	3.78762	-1.56131	-1.77578	H	4.37511	-0.96984	-1.55871
H	4.83799	-0.1678	-0.82309	H	4.85231	-0.23865	-1.04059	H	5.07737	0.48684	-0.66061
H	3.81389	1.28638	0.74126	H	3.92895	1.27641	0.53095	H	3.74355	1.64675	0.90709
H	1.38089	2.69035	0.82304	H	1.53196	2.73001	0.68229	H	1.27212	2.82814	0.85636
H	2.88904	3.09318	0.06314	H	3.0035	3.07279	-0.17263	H	2.72041	3.28586	0.01162
H	1.94988	1.61369	-1.96941	H	1.93464	1.54403	-2.09639	H	1.79057	1.64431	-1.90107
H	-0.72836	3.19064	-3.53843	H	-0.79387	3.10007	-3.58441	H	-0.85637	3.08734	-3.52784
H	0.06423	1.63404	-3.38837	H	-0.02234	1.53538	-3.41135	H	-0.12543	1.51793	-3.25998
H	-2.68938	2.39908	-2.29011	H	-2.70569	2.39529	-2.21449	H	-2.87392	2.45667	-2.30341
H	-2.37635	1.42079	-3.68922	H	-2.47786	1.35886	-3.588	H	-2.55687	1.36211	-3.61417
H	3.18163	0.28832	2.58464	H	3.3715	0.34242	2.43204	H	3.10529	0.59437	2.68683
H	1.88101	1.43956	2.55224	H	2.09353	1.51955	2.42744	H	1.63186	1.49687	2.51912
H	1.01327	-0.71869	3.18067	H	1.21055	-0.60087	3.16542	H	1.12046	-0.78327	3.09628
H	1.86181	-1.53222	1.88931	H	1.98034	-1.47327	1.86664	H	2.19285	-1.42594	1.8786
H	-0.13349	-2.67234	0.82136	H	-0.0455	-2.58787	0.94476	H	0.54273	-2.88516	1.10432
H	-2.63003	-2.75012	0.62375	H	-2.59058	-2.63561	0.89479	H	-0.27952	-2.76471	-1.08702
H	-2.72048	-1.03529	0.55005	H	-2.64052	-0.92519	0.76194	H	-1.39453	-3.64574	-0.0865
H	-2.37785	0.65523	-0.81478	H	-2.36698	0.69976	-0.69245	H	-2.82352	0.77937	-0.74692
H	-2.744	-2.58056	-1.58054	H	-2.81712	-2.54879	-1.30632	H	-2.78545	-1.4192	0.04878
H	-1.01897	-2.70283	-1.3833	H	-1.08627	-2.70446	-1.19473	H	-2.91053	-2.538	-1.27095
H	-0.30175	-1.71236	-3.48646	H	-0.43702	-1.79694	-3.35599	H	-0.23285	-1.48334	-2.9604
H	-1.99311	-1.92366	-3.98448	H	-2.15112	-2.00655	-3.76987	H	-1.78936	-2.17449	-3.46654
H	-1.17926	-0.37755	-4.22208	H	-1.33257	-0.48132	-4.10487	H	-1.35299	-0.54148	-3.942
		3d4									
C	-0.796	0.35237	1.54684								
O	-0.77536	-4.4836	2.37053								
C	-2.49398	-3.49492	3.738								
C	-1.45439	-3.51028	2.65957								
O	-1.38719	-2.28757	2.06733								
C	-0.71083	3.65082	-0.47076								
C	4.29018	-0.32614	-0.59906								
O	2.02503	-1.91425	-1.17852								
O	0.75393	-0.30649	-0.18287								
C	1.9279	-0.87453	-0.5493								
C	3.07279	-0.02558	-0.1151								

O	1.38608	3.63033	-1.65976							
C	2.82328	1.09108	0.8731							
C	2.16438	2.39709	0.31635							
C	1.55014	2.33895	-1.0582							
C	0.16553	2.90064	-1.43121							
C	-0.45764	2.41526	-2.72791							
C	-1.9014	1.90445	-2.64686							
C	2.18862	0.58513	2.20598							
C	1.35085	-0.71062	2.15926							
C	0.21982	-0.71315	1.09202							
C	-0.43918	-2.12743	0.98165							
C	-1.12725	-2.51467	-0.35182							
C	-2.15529	0.74219	-1.72345							
C	-2.25278	-1.62103	-0.89937							
C	-1.84597	-0.55696	-1.914							
C	-1.16674	-1.06749	-3.15948							
H	-1.07697	0.2265	2.59853							
H	-0.39676	1.36164	1.42598							
H	-1.71607	0.3214	0.97125							
H	-3.4712	-3.26278	3.3072							
H	-2.54535	-4.48203	4.20626							
H	-2.22499	-2.76047	4.50129							
H	-0.13328	4.18125	0.29286							
H	-1.30098	4.40438	-1.00331							
H	-1.40084	2.97707	0.04221							
H	4.43992	-1.147	-1.29704							
H	5.17175	0.23976	-0.31597							
H	3.81637	1.44215	1.19905							
H	1.4435	2.7878	1.03988							
H	2.95812	3.15649	0.2712							
H	2.02198	1.62262	-1.71609							
H	-0.44315	3.25008	-3.44106							
H	0.17255	1.63284	-3.1682							
H	-2.55978	2.72643	-2.33787							
H	-2.24165	1.6413	-3.65627							
H	3.01104	0.39058	2.9096							
H	1.60468	1.38275	2.67952							
H	0.92853	-0.89516	3.15581							
H	2.03766	-1.54884	1.98056							
H	0.37199	-2.85793	1.10695							
H	-0.36653	-2.67891	-1.11923							
H	-1.582	-3.50233	-0.19046							
H	-2.70374	1.0163	-0.82452							
H	-2.83392	-1.19742	-0.07347							
H	-2.96153	-2.2768	-1.42565							
H	-0.13327	-1.35389	-2.94723							
H	-1.69774	-1.94226	-3.5496							
H	-1.14285	-0.32908	-3.96515							

Detailed DP4+ probability for compound **3**. Isomer 1 is 1R*,3S*,4R*,11S*,12S*, isomer 2 is 1R*,3S*,4S*,11S*,12S*, isomer 3 is 1R*,3S*,4R*,11S*,12R*, isomer 4 is 1R*,3R*,4S*,11S*,12R*.

Functional	Solvent?	Basis Set	Type of Data
B3LYP	PCM	6-311+G(d,p)	Unscaled Shifts

	Isomer 1	Isomer 2	Isomer 3	Isomer 4	Isomer 5	Isomer 6
sDP4+ (H data)	0.01%	99.98%	0.02%	0.00%	–	–
sDP4+ (C data)	2.12%	0.00%	0.00%	97.88%	–	–
sDP4+ (all data)	99.18%	0.00%	0.47%	0.35%	–	–
uDP4+ (H data)	99.93%	0.00%	0.07%	0.00%	–	–
uDP4+ (C data)	45.95%	0.00%	53.89%	0.16%	–	–
uDP4+ (all data)	99.92%	0.00%	0.08%	0.00%	–	–
DP4+ (H data)	99.82%	0.00%	0.18%	0.00%	–	–
DP4+ (C data)	85.76%	0.00%	0.17%	14.07%	–	–
DP4+ (all data)	100.00%	0.00%	0.00%	0.00%	–	–

Table S9.Calculation process of 4

Important thermodynamic parameters (a.u.) of the optimized **4** with simplified structures at B3LYP/6-31+G(d,p) level in the gas phase.

NO.	E+ZPE	G	P%	NO.	E+ZPE	G	P%	NO.	E+ZPE	G	P%
4a1	-1005.835819	-1005.417492	12.59%	4a5	-1005.837003	-1005.418069	23.21%	4b3	-1005.8519	-1005.433858	1.62%
4a2	-1005.835463	-1005.417266	9.92%	4a6	-1005.834907	-1005.41706	7.97%	4b4	-1005.851226	-1005.433428	1.03%
4a3	-1005.834492	-1005.41619	3.17%	4b1	-1005.852889	-1005.433754	1.4%	4b5	-1005.856467	-1005.437608	86.5%
4a4	-1005.836875	-1005.418654	43.15%	4b2	-1005.851179	-1005.435318	7.60%				

Optimized Z-Matrixes of **4** with simplified structures in the Gas Phase (Å) at B3LYP/6-31+G(d,p) level.

4a1				4a2				4a3			
C	-2.82729	1.15926	0.55357	C	-2.82526	1.20408	0.57134	C	-2.8539	1.17936	0.50108
C	-2.59687	-0.33745	0.74487	C	-2.60978	-0.29621	0.75772	C	-2.62643	-0.3154	0.71292
C	-2.36215	1.90798	-0.71225	C	-2.35477	1.94873	-0.69402	C	-2.36616	1.91226	-0.76511
C	-0.94301	2.53684	-0.6798	C	-0.92791	2.56112	-0.66939	C	-0.94721	2.53608	-0.71299
C	0.26421	1.55621	-0.7927	C	0.27497	1.57329	-0.77485	C	0.25759	1.54897	-0.80291
C	-2.73964	-0.77035	2.18432	C	-2.77163	-0.73212	2.19462	C	-2.77554	-0.72993	2.15684
C	-2.31319	-1.2024	-0.24941	C	-2.32189	-1.16111	-0.23559	C	-2.34391	-1.19299	-0.27057
C	-2.07507	-2.68976	-0.15333	C	-2.09769	-2.65088	-0.13959	C	-2.11361	-2.6802	-0.15749
C	-0.70691	-3.13658	0.40153	C	-0.73472	-3.10928	0.41872	C	-0.75421	-3.12944	0.41627
C	0.53001	-2.5745	-0.28914	C	0.50859	-2.55946	-0.27085	C	0.49367	-2.57931	-0.26431
C	1.45019	-1.90683	0.43921	C	1.43223	-1.89715	0.45803	C	1.40921	-1.91193	0.47005
C	1.63208	2.28962	-0.786	C	1.64633	2.29814	-0.76136	C	1.63117	2.27225	-0.79008
C	2.86265	1.35613	-0.68613	C	2.86943	1.355	-0.6656	C	2.85528	1.33211	-0.66299
C	2.905	0.29857	0.4617	C	2.90333	0.2975	0.48183	C	2.87846	0.28279	0.49406
C	2.69927	-1.18686	-0.013	C	2.68819	-1.18731	0.00861	C	2.66889	-1.20471	0.02817
C	2.10609	0.71911	1.67565	C	2.10511	0.72537	1.69314	C	2.06864	0.71613	1.69633
C	0.67166	1.07851	1.49531	C	0.66841	1.07932	1.51552	C	0.6435	1.09753	1.49262
C	2.63215	0.83875	2.90661	C	2.63389	0.85285	2.92226	C	2.57764	0.83184	2.93468
O	0.22028	0.62703	0.30176	O	0.21694	0.64149	0.31614	O	0.20246	0.63218	0.30076
O	-0.00215	1.69301	2.30647	O	-0.01226	1.66584	2.34118	O	-0.02251	1.75579	2.27483
O	-0.86354	3.4879	-1.75617	O	-0.84607	3.52611	-1.73432	O	-0.87564	3.48021	-1.79589
C	0.19433	0.76632	-2.11466	C	0.20723	0.78308	-2.09719	C	0.19723	0.7457	-2.11711
C	0.6303	-2.85259	-1.76265	C	0.61101	-2.84387	-1.74284	C	0.6093	-2.86949	-1.73435
H	-3.91722	1.29239	0.61583	H	-3.91369	1.34821	0.63497	H	-3.94445	1.31418	0.54465
H	-2.42639	1.69468	1.42238	H	-2.4176	1.73348	1.4405	H	-2.46546	1.72357	1.37033
H	-3.06255	2.74764	-0.83701	H	-3.04134	2.80025	-0.81714	H	-3.05795	2.75611	-0.91059
H	-2.51588	1.31859	-1.62078	H	-2.52018	1.35997	-1.60111	H	-2.50836	1.31454	-1.67002
H	-0.84964	3.11546	0.24679	H	-0.83273	3.13772	0.25789	H	-0.88075	3.11958	0.2131
H	-3.71266	-0.45449	2.57575	H	-3.74873	-0.41474	2.57447	H	-3.74688	-0.40198	2.5425
H	-2.67621	-1.85238	2.3212	H	-2.71205	-1.81453	2.33009	H	-2.7212	-1.81078	2.30636
H	-1.95603	-0.31388	2.79705	H	-1.99479	-0.27916	2.8184	H	-1.98958	-0.27217	2.76563
H	-2.27483	-0.8341	-1.26816	H	-2.26863	-0.79194	-1.25273	H	-2.30546	-0.83596	-1.29363
H	-2.87	-3.14763	0.44856	H	-2.89804	-3.10231	0.45992	H	-2.91753	-3.12799	0.44011
H	-2.20726	-3.11422	-1.15735	H	-2.23102	-3.07363	-1.14418	H	-2.23704	-3.11372	-1.15876

H	-0.65665	-4.23182	0.33932	H	-0.69422	-4.20503	0.35859	H	-0.70896	-4.22539	0.36305
H	-0.67876	-2.90484	1.47385	H	-0.70671	-2.87564	1.49066	H	-0.73696	-2.88988	1.48706
H	1.27896	-1.85015	1.5149	H	1.25863	-1.83703	1.53333	H	1.2251	-1.84655	1.54313
H	1.73713	2.90508	-1.68889	H	1.75672	2.91786	-1.66069	H	1.75168	2.87405	-1.70018
H	1.67051	2.99843	0.05199	H	1.68789	3.00182	0.08073	H	1.66463	2.99228	0.0385
H	3.73137	2.01738	-0.5535	H	3.74388	2.00883	-0.53402	H	3.72549	1.99014	-0.52407
H	3.03408	0.87039	-1.6536	H	3.03486	0.86815	-1.63355	H	3.03786	0.83728	-1.62384
H	3.96505	0.31115	0.76442	H	3.96302	0.30285	0.78596	H	3.9351	0.2917	0.80859
H	3.5355	-1.77051	0.39842	H	3.51873	-1.7765	0.42359	H	3.49569	-1.79079	0.45489
H	2.82511	-1.24297	-1.09734	H	2.81684	-1.24596	-1.0753	H	2.80814	-1.27021	-1.05399
H	3.67475	0.6129	3.10384	H	3.67737	0.63063	3.11887	H	3.61355	0.5908	3.14872
H	2.03143	1.16484	3.75248	H	2.03461	1.18299	3.76772	H	1.9686	1.16843	3.77055
H	-1.53925	4.16847	-1.59033	H	-1.25101	3.13534	-2.52762	H	-0.27574	4.19413	-1.51836
H	-0.7304	0.20206	-2.21654	H	-0.73213	0.24665	-2.21628	H	-0.7246	0.17655	-2.21751
H	0.27633	1.42667	-2.98466	H	0.32536	1.43815	-2.96693	H	0.2814	1.39799	-2.99295
H	0.99328	0.02405	-2.18258	H	0.98577	0.01832	-2.14868	H	0.99899	0.00545	-2.17391
H	0.49893	-3.92386	-1.94979	H	0.4705	-3.91453	-1.92671	H	0.47623	-3.94175	-1.91444
H	-0.14429	-2.31206	-2.31392	H	-0.15687	-2.29808	-2.29841	H	-0.15719	-2.33073	-2.29852
H	1.59718	-2.57521	-2.18809	H	1.58186	-2.57667	-2.16586	H	1.58174	-2.59865	-2.15123
4a4				4a5				4a6			
C	-3.01633	1.02885	-0.06649	C	-3.02889	1.03034	-0.04929	C	-3.05051	1.00512	-0.04623
C	-2.67183	-0.38495	0.39646	C	-2.67107	-0.39778	0.35152	C	-2.69346	-0.42657	0.34985
C	-2.1772	2.17956	0.52056	C	-2.20994	2.16954	0.5885	C	-2.23102	2.13439	0.60573
C	-0.91657	2.66676	-0.23927	C	-0.92903	2.68793	-0.11506	C	-0.96767	2.67002	-0.11219
C	0.19684	1.62258	-0.56059	C	0.19637	1.66718	-0.46821	C	0.16294	1.65354	-0.46563
C	-2.73442	-0.62185	1.88288	C	-2.75121	-0.70246	1.8251	C	-2.76582	-0.73589	1.82233
C	-2.39902	-1.36255	-0.49304	C	-2.37782	-1.33148	-0.57796	C	-2.40484	-1.35669	-0.58447
C	-2.08438	-2.81236	-0.22806	C	-2.05701	-2.7904	-0.37445	C	-2.07886	-2.81474	-0.38883
C	-0.69877	-3.13026	0.3658	C	-0.67701	-3.12909	0.22067	C	-0.69812	-3.14952	0.20683
C	0.52042	-2.58963	-0.37307	C	0.54761	-2.54419	-0.47401	C	0.52539	-2.56019	-0.48655
C	1.45635	-1.90403	0.31704	C	1.46974	-1.88985	0.26326	C	1.44484	-1.90226	0.25095
C	1.58236	2.29532	-0.76013	C	1.57992	2.35757	-0.60449	C	1.54552	2.34674	-0.61272
C	2.7483	1.29168	-0.90138	C	2.755	1.37096	-0.77677	C	2.72389	1.36123	-0.78383
C	2.97168	0.25838	0.24581	C	2.96463	0.28584	0.32269	C	2.94161	0.2723	0.31264
C	2.72115	-1.23612	-0.16481	C	2.73575	-1.18887	-0.16481	C	2.71071	-1.20119	-0.17719
C	2.37065	0.67727	1.57077	C	2.3319	0.63767	1.65215	C	2.31852	0.61669	1.64861
C	0.93313	1.06816	1.62747	C	0.88318	0.98824	1.70438	C	0.88007	1.00376	1.70166
C	3.08351	0.76715	2.70708	C	3.02569	0.69722	2.80234	C	3.01195	0.64599	2.79992
O	0.28174	0.66613	0.51186	O	0.26031	0.65322	0.55031	O	0.24804	0.64767	0.55944
O	0.40649	1.65765	2.5577	O	0.31946	1.47748	2.67019	O	0.34354	1.56587	2.64254
O	-1.30686	3.35998	-1.43153	O	-1.28385	3.47794	-1.25783	O	-1.38378	3.41004	-1.26754
C	-0.09941	0.87802	-1.87693	C	-0.07214	0.99061	-1.82651	C	-0.10761	0.97169	-1.82072
C	0.59818	-2.91863	-1.83732	C	0.64741	-2.79798	-1.95167	C	0.62635	-2.81413	-1.9642
H	-3.04488	1.10582	-1.1587	H	-3.05261	1.14916	-1.13767	H	-3.06854	1.13503	-1.13369
H	-4.05694	1.19616	0.24758	H	-4.07351	1.17192	0.26417	H	-4.09614	1.14469	0.26466
H	-1.91235	1.95721	1.55798	H	-1.97415	1.91755	1.62653	H	-1.97978	1.86792	1.63618
H	-2.85387	3.04424	0.57987	H	-2.89083	3.03095	0.64959	H	-2.91625	2.99054	0.69059
H	-0.46894	3.43161	0.40955	H	-0.49833	3.40678	0.59445	H	-0.54836	3.41252	0.58035
H	-3.64314	-0.17511	2.30008	H	-3.67678	-0.29576	2.2462	H	-3.68193	-0.31807	2.25309
H	-2.75861	-1.68346	2.14293	H	-2.75391	-1.77476	2.03782	H	-2.78247	-1.80911	2.0301
H	-1.86847	-0.17952	2.3827	H	-1.9046	-0.26294	2.35875	H	-1.90801	-0.31074	2.35035
H	-2.42882	-1.11881	-1.55341	H	-2.39835	-1.04308	-1.62694	H	-2.43219	-1.06084	-1.63157
H	-2.85732	-3.23554	0.4258	H	-2.8354	-3.24703	0.24983	H	-2.85608	-3.27758	0.23231
H	-2.19445	-3.35513	-1.17634	H	-2.15269	-3.29006	-1.34771	H	-2.17093	-3.30981	-1.36473
H	-0.59779	-4.22288	0.41387	H	-0.56907	-4.22208	0.21543	H	-0.5867	-4.24217	0.20014
H	-0.68922	-2.79019	1.40929	H	-0.68339	-2.84162	1.27987	H	-0.70567	-2.86376	1.26647
H	1.30022	-1.81337	1.39272	H	1.29955	-1.85726	1.34033	H	1.27217	-1.86801	1.32746
H	1.5629	2.94258	-1.6467	H	1.57257	3.05106	-1.45558	H	1.53463	3.03424	-1.46844
H	1.80583	2.96625	0.07979	H	1.78214	2.98381	0.2744	H	1.74982	2.97951	0.26098
H	3.66211	1.89862	-0.9777	H	3.66553	1.98678	-0.80739	H	3.63254	1.97985	-0.81492
H	2.67862	0.77828	-1.86709	H	2.70539	0.90528	-1.76752	H	2.67461	0.89673	-1.77525

H	4.06575	0.28633	0.3815	H	4.05539	0.31553	0.48229	H	4.03338	0.30313	0.46515
H	3.54116	-1.82531	0.27108	H	3.55428	-1.79154	0.25509	H	3.5293	-1.80519	0.24072
H	2.84437	-1.33769	-1.2466	H	2.87696	-1.2365	-1.24813	H	2.85073	-1.24664	-1.26075
H	4.13886	0.51731	2.73789	H	4.08645	0.47249	2.83986	H	4.067	0.39552	2.83561
H	2.62917	1.09061	3.6408	H	2.55014	0.97068	3.74156	H	2.5409	0.9178	3.74191
H	-1.92452	4.06492	-1.16994	H	-1.91654	2.96935	-1.79258	H	-0.6568	4.00647	-1.51453
H	-1.1295	0.54593	-1.94645	H	-1.09719	0.64924	-1.92632	H	-1.13171	0.62842	-1.91861
H	0.07616	1.51502	-2.75146	H	0.10731	1.67541	-2.6632	H	0.06783	1.6546	-2.65989
H	0.5194	-0.01577	-1.9819	H	0.55905	0.11077	-1.96988	H	0.52556	0.09327	-1.96324
H	0.4573	-3.99456	-1.98657	H	0.51744	-3.866	-2.15704	H	0.4993	-3.88252	-2.16943
H	-0.17943	-2.38983	-2.39612	H	-0.1271	-2.2475	-2.49358	H	-0.14945	-2.26571	-2.5064
H	1.56039	-2.66082	-2.28518	H	1.61327	-2.51027	-2.37269	H	1.59156	-2.52396	-2.38502
4b1				4b2				4b3			
C	-2.82156	0.95805	0.17935	C	-2.93388	0.4654	-0.45507	C	-2.86227	0.6881	0.47598
C	-2.58526	-0.48598	0.59061	C	-2.91242	-0.93254	0.14007	C	-2.53697	-0.77861	0.72066
C	-2.4511	1.45821	-1.23017	C	-2.31726	1.49297	0.5103	C	-2.50347	1.34456	-0.86626
C	-0.9724	1.45462	-1.68445	C	-1.44089	2.58897	-0.13226	C	-1.48164	2.4959	-0.78776
C	0.11648	2.01422	-0.70638	C	0.09892	2.24452	-0.24121	C	0.00949	2.13205	-0.42194
C	-2.58415	-0.677	2.08776	C	-3.81755	-1.14936	1.32189	C	-2.72481	-1.1759	2.16636
C	-2.41169	-1.49995	-0.27904	C	-2.07203	-1.85241	-0.37252	C	-2.13487	-1.64667	-0.2291
C	-2.06971	-2.93292	0.0295	C	-1.75921	-3.22567	0.14757	C	-1.81203	-3.10752	-0.06008
C	-0.72319	-3.17637	0.73781	C	-0.47014	-3.23129	0.98925	C	-0.43616	-3.39154	0.5569
C	0.53184	-2.60745	0.08227	C	0.79201	-2.77073	0.2626	C	0.74859	-2.71264	-0.11509
C	1.31862	-1.75311	0.77168	C	1.44838	-1.66335	0.67503	C	1.47083	-1.80823	0.57733
C	1.5115	2.003	-1.38885	C	0.31866	0.99765	-1.11639	C	0.54249	1.0356	-1.36544
C	2.2645	0.67315	-1.29217	C	1.75329	0.65777	-1.51761	C	2.05359	0.82378	-1.37424
C	2.79683	0.31013	0.11234	C	2.75562	0.36233	-0.39377	C	2.72996	0.40122	-0.06102
C	2.67997	-1.2091	0.41189	C	2.7401	-1.09483	0.14753	C	2.75091	-1.12912	0.18318
C	2.34004	1.19672	1.25179	C	2.7993	1.41682	0.67713	C	2.34987	1.21374	1.14888
C	0.8979	1.4414	1.50394	C	1.68711	1.57785	1.62251	C	0.98558	1.18534	1.70495
C	3.20509	1.80154	2.08686	C	3.8661	2.20623	0.90078	C	3.2367	1.9453	1.84981
O	0.11043	1.14548	0.45047	O	0.50219	1.98792	1.13728	O	-0.02805	1.63805	0.94374
O	0.44599	1.7871	2.58632	O	1.85225	1.38543	2.82209	O	0.79074	0.80842	2.85677
O	-0.62809	0.13278	-2.10299	O	-2.0048	2.97774	-1.38536	O	-1.50727	3.16088	-2.06424
C	-0.24643	3.45573	-0.30706	C	0.81248	3.49538	-0.78512	C	0.80812	3.45215	-0.49109
C	0.83501	-3.13967	-1.29168	C	1.22487	-3.66969	-0.86542	C	1.04494	-3.1697	-1.51634
H	-3.89557	1.14795	0.31605	H	-2.41554	0.46948	-1.41955	H	-3.9523	0.77458	0.59376
H	-2.32607	1.60949	0.9085	H	-3.97116	0.74375	-0.68073	H	-2.44562	1.2842	1.29617
H	-2.80868	2.49471	-1.29521	H	-1.76375	1.00799	1.32284	H	-3.43528	1.77215	-1.26573
H	-3.04328	0.89464	-1.963	H	-3.1621	1.99928	0.99895	H	-2.20035	0.64228	-1.64494
H	-0.93864	2.05828	-2.60088	H	-1.52584	3.45607	0.53891	H	-1.84971	3.21994	-0.04832
H	-3.4287	-0.14249	2.53668	H	-4.85462	-0.93802	1.0407	H	-3.74083	-0.93112	2.49421
H	-2.69045	-1.72352	2.38446	H	-3.78899	-2.1709	1.70682	H	-2.57799	-2.24266	2.34942
H	-1.65882	-0.28544	2.52068	H	-3.5429	-0.48485	2.14696	H	-2.01611	-0.63199	2.79975
H	-2.50861	-1.30157	-1.34529	H	-1.47475	-1.57813	-1.24187	H	-2.02023	-1.31669	-1.25642
H	-2.07411	-3.49036	-0.91622	H	-1.66298	-3.90939	-0.70431	H	-1.87467	-3.58545	-1.04629
H	-2.87815	-3.37447	0.62552	H	-2.58039	-3.62489	0.75052	H	-2.58903	-3.59722	0.53932
H	-0.58442	-4.26301	0.82297	H	-0.29885	-4.25118	1.35771	H	-0.26452	-4.47596	0.53839
H	-0.7955	-2.82397	1.77349	H	-0.636	-2.61972	1.88651	H	-0.46832	-3.12675	1.62166
H	0.98844	-1.46271	1.77085	H	1.03868	-1.12645	1.53157	H	1.15102	-1.58508	1.59697
H	1.40287	2.2812	-2.44514	H	-0.09083	0.13203	-0.59696	H	0.06816	0.08083	-1.12381
H	2.15009	2.78525	-0.95703	H	-0.24627	1.1119	-2.04979	H	0.254	1.27561	-2.3967
H	1.67159	-0.14514	-1.70421	H	1.72299	-0.20724	-2.1938	H	2.28797	0.07919	-2.14674
H	3.13113	0.74041	-1.9652	H	2.14528	1.48127	-2.12911	H	2.52894	1.7486	-1.72753
H	3.88371	0.47404	0.0278	H	3.7334	0.41617	-0.90094	H	3.79363	0.62332	-0.24988
H	3.11691	-1.76584	-0.42413	H	3.1376	-1.73021	-0.64983	H	3.19671	-1.61383	-0.69206
H	3.33519	-1.43531	1.26544	H	3.477	-1.17061	0.95934	H	3.4653	-1.32918	0.99517
H	4.27826	1.67737	1.98468	H	4.76014	2.14379	0.28899	H	4.27975	2.0107	1.55816
H	2.86454	2.43122	2.90603	H	3.87985	2.92647	1.71594	H	2.94427	2.48855	2.74578
H	-0.467	-0.36791	-1.27801	H	-1.79021	3.91583	-1.52806	H	-2.40948	3.49995	-2.19904
H	-0.35545	4.09612	-1.18865	H	0.49323	3.72831	-1.80618	H	0.8274	3.86152	-1.50652

H	-1.17412	3.49663	0.27122	H	0.60337	4.36366	-0.14947	H	0.36474	4.20112	0.17577
H	0.52582	3.89672	0.33349	H	1.89901	3.39731	-0.80107	H	1.84544	3.35371	-0.17052
H	0.92874	-4.23007	-1.26136	H	1.25471	-4.71088	-0.5266	H	1.25613	-4.2439	-1.5255
H	0.0306	-2.88115	-1.98731	H	0.52005	-3.59869	-1.6997	H	0.18718	-2.98068	-2.1694
H	1.75907	-2.74165	-1.71603	H	2.21674	-3.43926	-1.25709	H	1.8995	-2.66088	-1.96706
4b4				4b5							
C	-2.88594	0.72707	0.46615	C	-3.20686	0.6635	-0.09954				
C	-2.56182	-0.74017	0.71223	C	-2.89541	-0.69285	0.52437				
C	-2.51203	1.38324	-0.87152	C	-2.10953	1.73482	0.09204				
C	-1.47479	2.52017	-0.7847	C	-0.90089	1.59893	-0.86493				
C	0.01259	2.14536	-0.41129	C	0.33945	2.49651	-0.52548				
C	-2.7479	-1.13375	2.15938	C	-3.04817	-0.77237	2.01897				
C	-2.1667	-1.61221	-0.237	C	-2.48961	-1.7206	-0.25023				
C	-1.8524	-3.07484	-0.06543	C	-2.04785	-3.09424	0.17327				
C	-0.48202	-3.36856	0.55891	C	-0.61691	-3.18976	0.74568				
C	0.71144	-2.69754	-0.10566	C	0.54422	-2.67025	-0.10332				
C	1.43654	-1.8004	0.5931	C	1.48573	-1.88717	0.46802				
C	0.54887	1.05106	-1.35559	C	1.58595	2.23188	-1.42358				
C	2.05895	0.82883	-1.34853	C	2.18845	0.81658	-1.47961				
C	2.7206	0.39823	-0.03035	C	2.84682	0.25906	-0.19809				
C	2.72598	-1.13264	0.21069	C	2.7491	-1.29623	-0.11411				
C	2.33621	1.21225	1.17698	C	2.49166	0.96279	1.0821				
C	0.96757	1.18935	1.72242	C	1.07751	1.2384	1.38221				
C	3.22123	1.93889	1.88513	C	3.38338	1.38488	1.99447				
O	-0.03831	1.63858	0.94911	O	0.69159	2.42652	0.8773				
O	0.76205	0.81253	2.87219	O	0.39829	0.56944	2.14403				
O	-1.50945	3.22049	-2.04263	O	-0.49956	0.24453	-0.94649				
C	0.81806	3.46143	-0.46663	C	-0.05703	3.97536	-0.72375				
C	1.01411	-3.15664	-1.50475	C	0.57098	-3.13121	-1.53359				
H	-3.97714	0.81267	0.57245	H	-3.42114	0.55712	-1.17075				
H	-2.47705	1.32292	1.29057	H	-4.13824	1.03304	0.34908				
H	-3.43305	1.82974	-1.27565	H	-1.77839	1.71969	1.13473				
H	-2.21516	0.67649	-1.64862	H	-2.56596	2.71897	-0.0682				
H	-1.84085	3.23454	-0.03541	H	-1.24072	1.85941	-1.87506				
H	-3.76032	-0.87895	2.49077	H	-4.06981	-0.5028	2.30639				
H	-2.61114	-2.20171	2.34313	H	-2.8544	-1.77255	2.41498				
H	-2.03169	-0.59575	2.78929	H	-2.35754	-0.08471	2.51532				
H	-2.05829	-1.28669	-1.26599	H	-2.40526	-1.54719	-1.32397				
H	-1.91246	-3.55268	-1.05187	H	-2.13352	-3.76015	-0.6951				
H	-2.63598	-3.55947	0.52958	H	-2.75368	-3.49751	0.90974				
H	-0.31778	-4.45415	0.54032	H	-0.41663	-4.24995	0.95193				
H	-0.51818	-3.1049	1.62378	H	-0.6122	-2.69325	1.72497				
H	1.11185	-1.57724	1.61136	H	1.3685	-1.67475	1.53245				
H	0.06407	0.09839	-1.12616	H	1.32457	2.5054	-2.45496				
H	0.27703	1.29832	-2.38903	H	2.39063	2.91567	-1.11979				
H	2.29631	0.08509	-2.12098	H	1.48082	0.10224	-1.906				
H	2.54381	1.75157	-1.69439	H	2.97946	0.8514	-2.24451				
H	3.78772	0.61168	-0.20935	H	3.92134	0.45086	-0.35221				
H	3.17401	-1.61971	-0.66212	H	2.97117	-1.71682	-1.09999				
H	3.43203	-1.34085	1.0279	H	3.56893	-1.64455	0.53051				
H	4.26693	1.9996	1.6021	H	4.44863	1.22833	1.86026				
H	2.9244	2.48301	2.77927	H	3.06879	1.88912	2.90489				
H	-1.51982	2.56352	-2.75941	H	-0.51764	-0.1391	-0.04746				
H	0.84304	3.87806	-1.47901	H	-0.39571	4.17046	-1.74652				
H	0.37592	4.20751	0.20425	H	-0.84921	4.27482	-0.02904				
H	1.85376	3.35549	-0.14337	H	0.79135	4.63685	-0.51065				
H	1.21814	-4.23222	-1.51264	H	0.49592	-4.22289	-1.57713				
H	0.16201	-2.96177	-2.16349	H	-0.26666	-2.70424	-2.09274				
H	1.87507	-2.65331	-1.94949	H	1.48528	-2.85145	-2.06091				

Detailed DP4+ probability for compound **4**. Isomer 1 is 1R*,11S*,12R*, isomer 2 is

1R*,11S*,12S*.

Functional	Solvent?	Basis Set	Type of Data			
B3LYP	PCM	6-311+G(d, p)	Unscaled Shifts			
	Isomer 1	Isomer 2	Isomer 3	Isomer 4	Isomer 5	Isomer 6
sDP4+ (H data)	37.54%	62.46%	–	–	–	–
sDP4+ (C data)	12.10%	87.90%	–	–	–	–
sDP4+ (all data)	7.64%	92.36%	–	–	–	–
uDP4+ (H data)	100.00%	0.00%	–	–	–	–
uDP4+ (C data)	100.00%	0.00%	–	–	–	–
uDP4+ (all data)	100.00%	0.00%	–	–	–	–
DP4+ (H data)	100.00%	0.00%	–	–	–	–
DP4+ (C data)	100.00%	0.00%	–	–	–	–
DP4+ (all data)	100.00%	0.00%	–	–	–	–

Table S10.Calculation process of 6

Important thermodynamic parameters (a.u.) of the optimized **6** with simplified structures at B3LYP/6-31+G(d,p) level in the gas phase.

NO.	E+ZPE	G	P%	NO.	E+ZPE	G	P%	NO.	E+ZPE	G	P%
6a1	-1424.713219	-1424.201352	24.97%	6b1	-1424.711679	-1424.19844	7.76%	6b6	-1424.712209	-1424.199968	39.18%
6a2	-1424.71123	-1424.200648	11.84	6b2	-1424.712099	-1424.198784	11.12%	6b7	-1424.71037	-1424.19843	7.68%
6a3	-1424.712213	-1424.200465	9.76%	6b3	-1424.711879	-1424.198524	8.48%	6b8	-1424.710672	-1424.197804	3.95%
6a4	-1424.713049	-1424.201541	30.51%	6b4	-1424.710321	-1424.198227	6.19%	6b9	-1424.711237	-1424.198525	8.49%
6a5	-1424.712619	-1424.201271	22.92%	6b5	-1424.710543	-1424.198062	5.20%	6b10	-1424.709357	-1424.196701	1.23%

Optimized Z-Matrixes of **6** with simplified structures in the Gas Phase (Å) at B3LYP/6-31+G(d,p) level.

6a1				6a2				6a3			
C	-1.87165	0.81405	1.52732	C	-0.82526	2.38628	-0.08334	C	-0.9268	2.37578	-0.05587
C	-2.62177	0.48436	0.18025	C	0.06084	2.36006	1.22045	C	-0.01096	2.3601	1.22738
C	-1.86211	-0.41665	-0.83841	C	1.05179	1.167	1.36376	C	1.01013	1.1892	1.34194
C	-1.69972	-0.48484	2.36201	C	0.07841	2.61718	-1.32565	C	-0.05819	2.6316	-1.31874
C	1.06168	-2.61605	-1.27666	C	2.59988	-1.66071	-0.41982	C	2.62086	-1.57912	-0.46919
C	2.3725	-2.76852	-0.52932	C	2.22432	-2.42772	-1.67324	C	2.2579	-2.34725	-1.72551
C	2.82487	-1.3231	-0.27281	C	0.70776	-2.63628	-1.54143	C	0.74985	-2.6079	-1.5829
C	2.6365	-0.83591	1.19867	C	-0.15427	-1.73319	-2.47714	C	-0.15678	-1.71821	-2.49155
C	-0.78897	0.28069	-1.6937	C	0.44571	-0.16249	1.84714	C	0.44478	-0.16084	1.8192
C	0.04066	-0.60818	-2.67409	C	1.3947	-1.40002	1.92707	C	1.4293	-1.37161	1.88831
C	1.28504	-1.33085	-2.05547	C	1.5987	-2.19208	0.59166	C	1.64968	-2.15179	0.54818
C	-0.6127	-0.48417	3.44677	C	-0.49421	2.21818	-2.69372	C	-0.64911	2.21369	-2.67444
C	1.18884	-0.98958	1.6881	C	0.14289	-0.23722	-2.29037	C	0.08616	-0.21513	-2.28703
C	0.82919	-0.19126	2.96381	C	-0.92156	0.73756	-2.84792	C	-1.02623	0.71788	-2.82236
C	1.03316	1.30268	2.84689	C	-2.30892	0.54416	-2.27957	C	-2.39254	0.47176	-2.2225
C	2.01562	1.95261	3.49616	C	-3.32856	0.04804	-3.00322	C	-3.3944	-0.10559	-2.90898
C	0.08425	2.12563	2.05434	C	-2.61529	0.98527	-0.89371	C	-2.69727	0.94532	-0.84888
O	-0.58888	1.37098	1.16327	O	-1.49079	1.10587	-0.16129	O	-1.56699	1.08304	-0.12705
O	-0.01731	3.33984	2.13259	O	-3.74053	1.14217	-0.44711	O	-3.81994	1.11755	-0.40195
C	-2.71208	1.8315	2.33122	C	-1.8462	3.54075	0.02882	C	-1.96694	3.50957	0.08722
C	-0.85607	-1.63594	-3.38646	C	2.76191	-1.01382	2.51795	C	2.7885	-0.94896	2.47301
C	4.28461	-1.13688	-0.70816	C	0.3493	-4.11124	-1.76879	C	0.43714	-4.08918	-1.83049
O	2.01384	-0.48675	-1.13622	O	0.37463	-2.31958	-0.16529	O	0.42563	-2.32113	-0.19894
O	3.47719	-1.54933	2.10709	O	0.06849	-2.07399	-3.84655	O	-0.00024	-2.02663	-3.87831
O	0.47714	0.24693	-3.77395	O	0.85276	-2.29775	2.94297	O	0.92044	-2.28973	2.90244
C	1.42317	1.26185	-3.46269	C	-0.38582	-2.93042	2.64627	C	-0.30307	-2.95417	2.61204
O	-2.96907	1.72773	-0.47598	O	-0.81047	2.37565	2.37687	O	-0.85604	2.34914	2.40321
O	-4.86633	0.78098	-1.35832	O	0.74502	3.55776	3.58399	O	0.70285	3.55163	3.58529
C	-4.10779	1.72947	-1.2182	C	-0.33698	2.99649	3.48961	C	-0.3708	2.97166	3.50987
C	-4.30438	3.07391	-1.84792	C	-1.34036	2.8951	4.5967	C	-1.34855	2.84484	4.63707
H	-3.54498	-0.03949	0.46638	H	0.65217	3.28671	1.20504	H	0.55915	3.29985	1.20536
H	-1.45194	-1.29261	-0.33989	H	1.61279	1.02546	0.44223	H	1.55982	1.07231	0.41021

H	-2.62074	-0.80139	-1.53205	H	1.79173	1.47111	2.11524	H	1.75396	1.50338	2.08525
H	-2.6596	-0.73556	2.83366	H	0.36569	3.67667	-1.36604	H	0.19644	3.69912	-1.36629
H	-1.49009	-1.33721	1.71023	H	1.02733	2.08512	-1.21655	H	0.90866	2.12887	-1.22973
H	0.24439	-2.5267	-0.55973	H	2.47617	-0.5918	-0.59961	H	2.45936	-0.5137	-0.63894
H	0.85236	-3.4737	-1.92256	H	3.63814	-1.83635	-0.1237	H	3.66763	-1.72176	-0.18553
H	3.08828	-3.29486	-1.17355	H	2.75389	-3.38889	-1.6735	H	2.81858	-3.29053	-1.7388
H	2.27242	-3.36908	0.3796	H	2.52235	-1.90835	-2.58881	H	2.53455	-1.80869	-2.63607
H	2.91607	0.22297	1.24038	H	-1.20632	-1.93359	-2.24373	H	-1.19527	-1.94802	-2.22975
H	-1.2929	1.05306	-2.29111	H	0.03568	0.00671	2.85249	H	0.03345	-0.01008	2.82689
H	-0.09781	0.83133	-1.0534	H	-0.41908	-0.425	1.23547	H	-0.41363	-0.44472	1.20806
H	1.98297	-1.55033	-2.87667	H	1.89698	-3.21719	0.85591	H	1.98717	-3.16643	0.80535
H	-0.61569	-1.48148	3.90753	H	0.28455	2.42173	-3.44142	H	0.10414	2.4459	-3.43972
H	-0.893	0.21069	4.2488	H	-1.32916	2.88242	-2.95121	H	-1.5132	2.84781	-2.91081
H	0.52413	-0.70015	0.8766	H	0.28258	-0.05136	-1.22712	H	0.22838	-0.03729	-1.22273
H	0.99236	-2.04885	1.89945	H	1.0947	0.00192	-2.78251	H	1.02236	0.06845	-2.78519
H	1.49018	-0.55641	3.76335	H	-0.97843	0.55799	-3.93133	H	-1.10009	0.53579	-3.90446
H	2.13177	3.03166	3.42446	H	-4.32715	-0.05575	-2.58312	H	-4.37907	-0.25049	-2.47063
H	2.73132	1.42364	4.11726	H	-3.20174	-0.26436	-4.03406	H	-3.25455	-0.46018	-3.92544
H	-2.75677	2.80309	1.8271	H	-2.57751	3.35469	0.82297	H	-2.67746	3.30349	0.89518
H	-3.73606	1.47144	2.47845	H	-1.34628	4.49312	0.23606	H	-1.4809	4.46974	0.29154
H	-2.28172	2.01508	3.32174	H	-2.41819	3.66354	-0.89734	H	-2.56022	3.62885	-0.82593
H	-0.29153	-2.17017	-4.16016	H	3.37606	-1.90677	2.68571	H	3.42874	-1.82473	2.63371
H	-1.68404	-1.13612	-3.90328	H	2.64037	-0.53833	3.49855	H	2.65917	-0.48095	3.45622
H	-1.27676	-2.37774	-2.70156	H	3.32441	-0.33214	1.87355	H	3.32784	-0.24925	1.82814
H	4.40256	-1.33674	-1.77958	H	0.85461	-4.75483	-1.03937	H	0.97279	-4.72772	-1.1185
H	4.60543	-0.10018	-0.55526	H	-0.72483	-4.27547	-1.62581	H	-0.62904	-4.29131	-1.67704
H	4.96268	-1.79761	-0.15921	H	0.62318	-4.44738	-2.77376	H	0.70888	-4.39851	-2.84461
H	4.38568	-1.22451	1.97941	H	-0.7017	-1.76314	-4.35238	H	0.91773	-1.84121	-4.13693
H	2.40903	0.83054	-3.27306	H	-1.15503	-2.2144	2.34897	H	-1.09308	-2.2581	2.322
H	1.51422	1.90945	-4.33971	H	-0.25755	-3.70018	1.88147	H	-0.1591	-3.71795	1.84405
H	1.10397	1.8815	-2.6219	H	-0.72852	-3.43009	3.55718	H	-0.62612	-3.46556	3.52361
H	-5.22409	3.06598	-2.43979	H	-0.95103	3.39883	5.48603	H	-0.95078	3.35195	5.52073
H	-4.39651	3.83705	-1.07106	H	-2.2714	3.38378	4.29883	H	-2.29513	3.31635	4.36132
H	-3.46505	3.29822	-2.51099	H	-1.51734	1.84455	4.8406	H	-1.49953	1.78978	4.87898
6a4				6a5				6b1			
C	-1.41896	2.13202	0.53384	C	-1.39811	2.11417	0.54357	C	1.55609	2.16463	0.40965
C	0.06545	2.02562	0.02489	C	0.08448	2.00883	0.02933	C	2.36394	0.88109	0.00819
C	0.98981	1.20239	0.94932	C	1.01324	1.19066	0.95306	C	1.55809	-0.40472	-0.26824
C	-2.38046	2.41046	-0.65252	C	-2.36273	2.40164	-0.63807	C	0.92468	2.09971	1.82855
C	1.84333	-2.02909	-1.31439	C	1.85096	-2.05454	-1.30599	C	-2.42158	-1.21041	-1.78386
C	0.7806	-3.11014	-1.38287	C	0.78013	-3.12811	-1.36948	C	-3.44329	-1.46852	-0.68941
C	-0.05132	-2.89674	-0.1069	C	-0.04976	-2.90325	-0.09395	C	-2.593	-1.6677	0.57823
C	-1.48303	-2.32125	-0.35762	C	-1.48185	-2.33205	-0.34588	C	-2.85193	-0.61488	1.699
C	2.26302	0.74867	0.20766	C	2.28125	0.72969	0.2064	C	0.85909	-0.4352	-1.63756
C	2.85052	-0.61622	0.67398	C	2.86483	-0.63605	0.67497	C	0.15847	-1.77234	-2.0007
C	2.04205	-1.87343	0.18603	C	2.04794	-1.89016	0.19371	C	-1.21533	-1.98254	-1.27523
C	-2.56481	1.26346	-1.67355	C	-2.55168	1.25998	-1.66432	C	-0.37347	1.30147	2.01285
C	-1.45777	-0.96828	-1.08094	C	-1.46117	-0.98356	-1.07978	C	-2.78709	0.87897	1.32204
C	-2.79159	-0.17306	-1.12159	C	-2.78811	-0.17653	-1.11677	C	-1.41974	1.44816	0.89188
C	-3.59217	-0.15226	0.16214	C	-3.58729	-0.15333	0.16684	C	-1.47552	2.89279	0.41618
C	-4.79495	-0.7418	0.27858	C	-4.80549	-0.71171	0.27797	C	-2.45661	3.77817	0.6677
C	-3.06704	0.57317	1.35244	C	-3.04582	0.55344	1.36295	C	-0.34485	3.38215	-0.43322
O	-1.76401	0.88091	1.18672	O	-1.74366	0.8581	1.18773	O	0.59031	2.42584	-0.63651
O	-3.69482	0.78988	2.37601	O	-3.6646	0.76027	2.39372	O	-0.30483	4.4771	-0.97275
C	-1.55212	3.25067	1.58158	C	-1.52604	3.22577	1.59923	C	2.55821	3.34748	0.40504
C	4.28617	-0.70334	0.11126	C	4.29835	-0.73233	0.10897	C	-0.08236	-1.82367	-3.52546
C	-0.15325	-4.21023	0.68352	C	-0.15537	-4.21015	0.70725	C	-2.83602	-3.07729	1.16148
O	0.70399	-1.96359	0.71283	O	0.70742	-1.96183	0.71562	O	-1.21333	-1.64039	0.1315
O	-2.2848	-3.21654	-1.12936	O	-2.26616	-3.25603	-1.10135	O	-4.17265	-0.82953	2.23178
O	2.93846	-0.50843	2.11152	O	2.95845	-0.52271	2.11189	O	0.97227	-2.89811	-1.58995
C	3.25855	-1.68438	2.83585	C	3.26541	-1.69988	2.84015	C	2.19141	-3.11485	-2.28732

O	0.66288	3.33354	-0.14006	O	0.67935	3.31686	-0.14284	O	3.3089	0.59302	1.07188
O	0.42931	3.24027	-2.42469	O	0.42314	3.22012	-2.4247	O	4.82588	-0.23564	-0.44642
C	0.79187	3.8101	-1.40605	C	0.79805	3.79072	-1.41088	C	4.49347	0.03507	0.69824
C	1.44461	5.15791	-1.37806	C	1.45499	5.13662	-1.39179	C	5.3454	-0.20539	1.90647
H	0.05716	1.51126	-0.93937	H	0.07349	1.49083	-0.93285	H	2.91696	1.12288	-0.91046
H	1.28309	1.80605	1.81719	H	1.31334	1.79974	1.81481	H	0.8608	-0.58958	0.54349
H	0.46904	0.32798	1.33789	H	0.49292	0.32048	1.35142	H	2.2531	-1.25038	-0.21315
H	-3.37645	2.65332	-0.2562	H	-3.35706	2.64409	-0.23736	H	0.6964	3.12713	2.14861
H	-2.06466	3.31292	-1.18968	H	-2.04732	3.30657	-1.17114	H	1.66173	1.74594	2.56013
H	1.44825	-1.11702	-1.77041	H	1.46313	-1.1426	-1.76865	H	-2.77719	-1.55155	-2.75993
H	2.75847	-2.31003	-1.84271	H	2.76486	-2.34582	-1.83083	H	-2.21044	-0.1376	-1.84397
H	1.26862	-4.09294	-1.37509	H	1.2601	-4.11478	-1.35845	H	-4.17656	-0.66112	-0.61784
H	0.19581	-3.06189	-2.3062	H	0.19575	-3.0782	-2.29299	H	-4.01319	-2.37547	-0.92752
H	-1.96326	-2.1893	0.61884	H	-1.95433	-2.20691	0.63536	H	-2.14786	-0.80236	2.51827
H	2.10699	0.73023	-0.87567	H	2.12008	0.70807	-0.8761	H	1.63189	-0.23022	-2.39045
H	3.01202	1.53489	0.37998	H	3.03428	1.51338	0.37257	H	0.13553	0.37555	-1.73989
H	2.56404	-2.76938	0.54931	H	2.56062	-2.78815	0.56494	H	-1.43853	-3.05778	-1.33569
H	-1.71931	1.25884	-2.37226	H	-1.7053	1.25239	-2.3618	H	-0.12775	0.24523	2.15791
H	-3.4374	1.52867	-2.28707	H	-3.42187	1.53253	-2.27801	H	-0.81813	1.62021	2.96607
H	-0.68183	-0.36519	-0.62663	H	-0.67686	-0.3807	-0.63882	H	-3.53145	1.08266	0.54203
H	-1.14909	-1.1228	-2.12348	H	-1.16258	-1.14849	-2.1237	H	-3.14401	1.44609	2.19411
H	-3.39663	-0.68837	-1.88299	H	-3.39799	-0.68505	-1.87887	H	-1.10729	0.84836	0.03717
H	-5.36228	-0.70514	1.20555	H	-5.36943	-0.66648	1.20761	H	-2.42059	4.79318	0.27629
H	-5.24392	-1.28024	-0.54994	H	-5.28116	-1.21901	-0.55472	H	-3.3267	3.53481	1.26595
H	-1.32354	4.23587	1.1629	H	-1.29476	4.21306	1.18703	H	2.05712	4.29594	0.62947
H	-2.56808	3.29231	1.98961	H	-2.54128	3.26787	2.009	H	3.3582	3.20927	1.13967
H	-0.88674	3.0676	2.43249	H	-0.86011	3.03468	2.44791	H	3.01078	3.47002	-0.5858
H	4.3045	-0.57034	-0.97545	H	4.31458	-0.60402	-0.97832	H	-0.5304	-2.78047	-3.81633
H	4.74808	-1.67028	0.33696	H	4.75615	-1.70046	0.33798	H	0.85267	-1.73019	-4.08833
H	4.92956	0.06056	0.56309	H	4.94652	0.03047	0.55581	H	-0.73327	-1.0109	-3.86307
H	0.83894	-4.57132	0.97785	H	0.83661	-4.58306	0.98694	H	-2.58154	-3.86301	0.44197
H	-0.70992	-4.05875	1.61532	H	-0.6935	-4.04495	1.64763	H	-2.19445	-3.24642	2.03405
H	-0.64683	-4.99773	0.10558	H	-0.67111	-4.99353	0.14301	H	-3.87843	-3.22462	1.46141
H	-2.59155	-3.9189	-0.52949	H	-3.20024	-3.03323	-0.94529	H	-4.23548	-0.33498	3.06729
H	4.16695	-2.15925	2.45773	H	4.16473	-2.18966	2.45926	H	2.7872	-2.20333	-2.37461
H	3.43344	-1.40021	3.87752	H	3.45003	-1.41341	3.87951	H	2.77343	-3.84994	-1.72412
H	2.42324	-2.38934	2.82202	H	2.41966	-2.39242	2.83406	H	1.99507	-3.53642	-3.27684
H	2.43692	5.0798	-0.92674	H	2.45008	5.05703	-0.94691	H	6.29658	-0.64794	1.59713
H	1.55597	5.52811	-2.40121	H	1.56073	5.50296	-2.41692	H	5.54999	0.7427	2.41014
H	0.82085	5.85971	-0.81881	H	0.83711	5.84213	-0.83071	H	4.84119	-0.90018	2.58279
6b2				6b3				6b4			
C	1.51151	2.21779	0.39496	C	1.53309	2.12474	0.67429	C	1.81869	2.08347	0.3735
C	2.34108	0.94594	-0.00023	C	2.36288	0.88092	0.19854	C	2.44946	0.8988	-0.43595
C	1.55716	-0.35547	-0.2687	C	1.57435	-0.37809	-0.21773	C	1.54729	-0.30922	-0.7572
C	0.89065	2.15393	1.81839	C	0.85169	1.94079	2.05916	C	1.52747	1.7478	1.86332
C	-2.40401	-1.22716	-1.76309	C	-2.33305	-1.05114	-1.96182	C	-2.69045	-0.65379	-1.43428
C	-3.41705	-1.51106	-0.66701	C	-3.39857	-1.39536	-0.93448	C	-3.50184	-1.08647	-0.22334
C	-2.55884	-1.70717	0.59574	C	-2.60189	-1.71019	0.34524	C	-2.44734	-1.61918	0.76618
C	-2.81591	-0.65837	1.72275	C	-2.90506	-0.75932	1.5454	C	-2.35623	-0.82699	2.10691
C	0.85891	-0.40498	-1.63774	C	0.93332	-0.29357	-1.61292	C	0.54735	-0.08214	-1.90481
C	0.1822	-1.75552	-1.9958	C	0.2545	-1.59555	-2.11774	C	-0.29062	-1.33196	-2.30668
C	-1.18603	-1.98961	-1.26792	C	-1.1482	-1.8667	-1.47221	C	-1.50604	-1.60434	-1.36234
C	-0.38605	1.32594	2.01842	C	-0.44617	1.12466	2.12937	C	0.26652	0.9378	2.19313
C	-2.79115	0.83519	1.34127	C	-2.83216	0.76294	1.31163	C	-2.26299	0.71011	2.04019
C	-1.44105	1.43637	0.90143	C	-1.45078	1.36676	0.98812	C	-0.99919	1.32276	1.40594
C	-1.53491	2.87536	0.41499	C	-1.49196	2.84712	0.64006	C	-1.07062	2.83362	1.23872
C	-2.53561	3.73772	0.66889	C	-2.47106	3.71321	0.95748	C	-1.89163	3.67176	1.89692
C	-0.42282	3.38564	-0.44601	C	-0.34626	3.39973	-0.14736	C	-0.15835	3.45677	0.23024
O	0.53534	2.45179	-0.64817	O	0.60813	2.47076	-0.38527	O	0.65499	2.54667	-0.35257
O	-0.41438	4.47576	-0.99676	O	-0.31296	4.52326	-0.62544	O	-0.20334	4.62748	-0.11485
C	2.49052	3.41945	0.37368	C	2.52712	3.30708	0.80499	C	2.85058	3.24054	0.35635

C	-0.06167	-1.814	-3.51986	C	0.07875	-1.51725	-3.65024	C	-0.79658	-1.10937	-3.74786
C	-2.78351	-3.12402	1.16802	C	-2.86928	-3.16423	0.79055	C	-2.72714	-3.10461	1.08761
O	-1.18183	-1.66649	0.14346	O	-1.20723	-1.64602	-0.04332	O	-1.18635	-1.59146	0.04624
O	-4.12105	-0.86948	2.29301	O	-4.22877	-1.01902	2.05116	O	-3.53297	-1.11222	2.88687
O	1.01632	-2.86615	-1.58474	O	1.05323	-2.75246	-1.77037	O	0.65863	-2.42322	-2.33048
C	2.23953	-3.0599	-2.28198	C	2.30372	-2.90743	-2.4277	C	0.16716	-3.73626	-2.53498
O	3.29311	0.6809	1.06305	O	3.26719	0.50584	1.2701	O	3.58538	0.39599	0.31658
O	4.81823	-0.13793	-0.45241	O	4.84282	-0.19663	-0.25188	O	4.8424	0.19883	-1.6013
C	4.4855	0.13915	0.69065	C	4.46635	-0.02066	0.89769	C	4.70002	0.07298	-0.39448
C	5.34551	-0.075	1.89811	C	5.27189	-0.36057	2.11383	C	5.75227	-0.46025	0.52882
H	2.88844	1.19202	-0.92114	H	2.95041	1.20041	-0.6738	H	2.78928	1.30914	-1.39682
H	0.86236	-0.54711	0.54355	H	0.84395	-0.63038	0.54523	H	1.04843	-0.66126	0.14225
H	2.26595	-1.18923	-0.20866	H	2.26943	-1.22531	-0.2043	H	2.21047	-1.1375	-1.03884
H	0.63939	3.17896	2.12868	H	0.60536	2.9374	2.45439	H	1.43316	2.69466	2.41532
H	1.64032	1.82604	2.54921	H	1.56433	1.53055	2.78543	H	2.39268	1.25348	2.32217
H	-2.75941	-1.56259	-2.74122	H	-2.64901	-1.30784	-2.97663	H	-3.26092	-0.74233	-2.36299
H	-2.20756	-0.15122	-1.81302	H	-2.11967	0.02227	-1.92313	H	-2.38049	0.38886	-1.31615
H	-4.16554	-0.71891	-0.5856	H	-4.12873	-0.58957	-0.82539	H	-4.11912	-0.27391	0.16728
H	-3.97121	-2.42608	-0.91173	H	-3.96347	-2.27328	-1.27222	H	-4.19968	-1.88065	-0.51764
H	-2.08937	-0.8155	2.52871	H	-2.21987	-1.01092	2.36272	H	-1.49986	-1.20846	2.67565
H	1.62783	-0.18926	-2.39161	H	1.73576	-0.02441	-2.31272	H	1.14768	0.22161	-2.77405
H	0.12101	0.39228	-1.74347	H	0.21071	0.52207	-1.6759	H	-0.12512	0.75244	-1.7014
H	-1.39644	-3.06676	-1.34028	H	-1.36648	-2.93233	-1.6344	H	-1.88624	-2.60665	-1.59237
H	-0.11263	0.2777	2.17184	H	-0.19907	0.06069	2.19109	H	0.47607	-0.12816	2.06316
H	-0.83334	1.64273	2.97103	H	-0.92629	1.35852	3.08992	H	0.07541	1.0583	3.26867
H	-3.54597	1.02069	0.56697	H	-3.54552	1.03953	0.52511	H	-3.15498	1.09934	1.53365
H	-3.15583	1.39354	2.21616	H	-3.22523	1.24367	2.21936	H	-2.3537	1.08315	3.07093
H	-1.11665	0.83759	0.05054	H	-1.10445	0.84375	0.09648	H	-0.93088	0.89599	0.40513
H	-2.52912	4.75074	0.27145	H	-2.42389	4.75894	0.65941	H	-1.88318	4.74311	1.70471
H	-3.39326	3.47477	1.2775	H	-3.34656	3.42363	1.52682	H	-2.59752	3.33281	2.64592
H	1.97231	4.35997	0.59262	H	3.01772	3.51091	-0.15387	H	3.07269	3.54861	-0.67194
H	3.29743	3.30311	1.10451	H	2.01086	4.23254	1.08454	H	2.46013	4.13074	0.86249
H	2.93472	3.54186	-0.62097	H	3.29838	3.11243	1.55732	H	3.78829	2.96037	0.84719
H	-0.49251	-2.7795	-3.80797	H	-0.35303	-2.44594	-4.04004	H	-1.48359	-1.90312	-4.0598
H	0.87002	-1.70409	-4.08529	H	1.03653	-1.37637	-4.16274	H	0.03649	-1.11962	-4.46027
H	-0.72871	-1.01421	-3.85699	H	-0.56027	-0.67883	-3.94515	H	-1.32261	-0.15419	-3.84915
H	-2.49911	-3.90093	0.45008	H	-2.58807	-3.88425	0.01449	H	-2.69873	-3.72699	0.18667
H	-2.15549	-3.28421	2.05199	H	-2.26288	-3.41334	1.66905	H	-1.95481	-3.50364	1.75538
H	-3.82844	-3.29503	1.44554	H	-3.92305	-3.33171	1.03522	H	-3.70438	-3.24695	1.55987
H	-4.04773	-1.60494	2.92614	H	-4.87316	-0.74225	1.37776	H	-3.36516	-0.80379	3.79439
H	2.81917	-2.13787	-2.36723	H	2.86285	-3.6883	-1.90434	H	-0.51197	-3.79414	-3.38845
H	2.83419	-3.78558	-1.71978	H	2.15409	-3.24175	-3.45788	H	1.02277	-4.38675	-2.73844
H	2.05125	-3.48309	-3.27237	H	2.89889	-1.99154	-2.40887	H	-0.31906	-4.10939	-1.62964
H	6.3035	-0.50316	1.58954	H	5.45493	0.54265	2.70128	H	6.6394	-0.73161	-0.05056
H	5.53472	0.88116	2.39247	H	4.74368	-1.10819	2.71083	H	6.03087	0.30722	1.25527
H	4.85603	-0.77249	2.5824	H	6.2352	-0.77704	1.80583	H	5.38164	-1.35462	1.03598
6b5				6b6				6b7			
C	1.80436	2.13305	0.27172	C	-0.31143	2.42034	0.94691	C	0.28937	2.6819	0.7436
C	2.50618	0.82205	-0.22447	C	-0.89509	2.29969	-0.5154	C	0.64794	2.4558	-0.76551
C	1.62136	-0.41829	-0.4621	C	-0.70561	0.96867	-1.27344	C	0.50528	1.02746	-1.32835
C	1.30561	2.0662	1.74255	C	1.23223	2.2484	1.02409	C	1.31109	2.05917	1.73631
C	-2.48034	-0.84652	-1.65766	C	0.38617	-3.45496	-1.76912	C	-2.14281	-2.21522	-0.33063
C	-3.46286	-1.0739	-0.51995	C	0.90657	-3.95686	-0.43175	C	-1.5608	-3.24957	0.62024
C	-2.57088	-1.44959	0.67918	C	1.52089	-2.70596	0.20378	C	-0.04537	-2.96727	0.60595
C	-2.62449	-0.44569	1.87376	C	1.50971	-2.70496	1.7586	C	0.54917	-2.53973	1.98481
C	0.80223	-0.38622	-1.76447	C	-1.61408	-0.16931	-0.7973	C	-0.94034	0.5927	-1.62788
C	-0.00737	-1.68121	-2.06899	C	-1.42923	-1.52772	-1.55242	C	-1.08372	-0.806	-2.29799
C	-1.3491	-1.78735	-1.27452	C	0.06991	-1.9815	-1.51271	C	-0.98531	-1.99819	-1.29182
C	-0.00498	1.32237	2.03084	C	1.82146	0.83411	1.17739	C	1.23122	0.55179	2.01129
C	-2.49284	1.05821	1.56487	C	1.93863	-1.38943	2.47236	C	-0.16629	-1.43236	2.78293
C	-1.14145	1.55846	1.01945	C	1.09058	-0.11771	2.15078	C	-0.18884	-0.01742	2.17411

C	-1.15807	3.01701	0.58603	C	0.5971	0.68735	3.33396	C	-1.05576	0.96507	2.94667
C	-2.0448	3.95236	0.97132	C	1.18559	0.78998	4.53715	C	-1.4288	0.85786	4.23466
C	-0.10331	3.45985	-0.37738	C	-0.68482	1.43123	3.12454	C	-1.56359	2.16006	2.20512
O	0.76338	2.46557	-0.67804	O	-1.037	1.50908	1.81291	O	-1.07204	2.23059	0.947
O	-0.07625	4.55285	-0.92184	O	-1.39504	1.84373	4.02884	O	-2.41404	2.92664	2.63073
C	2.86003	3.26586	0.19635	C	-0.64572	3.85804	1.43489	C	0.29278	4.21362	0.9834
C	-0.3032	-1.69805	-3.58394	C	-2.3456	-2.56321	-0.87013	C	-2.45323	-0.84348	-3.00903
C	-2.94458	-2.85917	1.18939	C	2.9364	-2.45153	-0.33429	C	0.71972	-4.21122	0.1028
O	-1.2248	-1.5609	0.14679	O	0.64805	-1.66066	-0.2395	O	0.14641	-1.93684	-0.39743
O	-3.88942	-0.56736	2.55078	O	0.20253	-3.04357	2.24574	O	0.607	-3.6802	2.86335
O	0.90371	-2.76623	-1.77767	O	-1.74732	-1.43163	-2.95505	O	-0.05362	-0.83961	-3.31238
C	0.41076	-4.09289	-1.84231	C	-3.04373	-0.96791	-3.3025	C	0.16228	-2.05278	-4.0115
O	3.50972	0.4511	0.75762	O	-0.27222	3.33175	-1.32951	O	2.02881	2.85892	-0.96416
O	5.01146	-0.09614	-0.89841	O	-2.208	3.60078	-2.5424	O	1.50445	3.87954	-2.95912
C	4.70068	-0.00176	0.27932	C	-1.04791	3.89578	-2.29609	C	2.31151	3.5447	-2.10518
C	5.59836	-0.36769	1.42139	C	-0.26582	4.94139	-3.02987	C	3.77778	3.84564	-2.16466
H	2.9902	1.05791	-1.1822	H	-1.97517	2.49064	-0.44432	H	-0.01798	3.10046	-1.35554
H	0.98847	-0.60546	0.40168	H	0.3488	0.69141	-1.24704	H	1.01067	0.31212	-0.68439
H	2.29097	-1.2874	-0.49888	H	-0.91164	1.13594	-2.33855	H	1.07476	0.99476	-2.2663
H	1.15671	3.09548	2.10104	H	1.59569	2.81754	1.89251	H	1.18599	2.55336	2.71112
H	2.08921	1.66376	2.39639	H	1.71059	2.73698	0.16592	H	2.33715	2.30784	1.43822
H	-2.92123	-1.07454	-2.63205	H	1.18129	-3.52959	-2.52146	H	-3.05166	-2.57442	-0.82129
H	-2.15577	0.19819	-1.6608	H	-0.44851	-4.04378	-2.1556	H	-2.38538	-1.30311	0.22299
H	-4.10631	-0.20703	-0.35244	H	0.05546	-4.32766	0.1496	H	-2.02216	-3.19685	1.60922
H	-4.1353	-1.90072	-0.78205	H	1.61729	-4.78246	-0.53606	H	-1.77556	-4.25628	0.24
H	-1.84768	-0.71378	2.59984	H	2.17465	-3.5041	2.10833	H	1.58854	-2.22956	1.82785
H	1.5291	-0.23875	-2.57577	H	-2.65406	0.16624	-0.8907	H	-1.34658	1.34608	-2.31752
H	0.13314	0.47447	-1.80453	H	-1.46426	-0.34397	0.27039	H	-1.57086	0.63264	-0.7385
H	-1.72727	-2.80902	-1.3995	H	0.61608	-1.39756	-2.26598	H	-0.87827	-2.91896	-1.87731
H	0.19924	0.2511	2.11909	H	1.90089	0.37578	0.19033	H	1.76273	0.00995	1.22337
H	-0.33986	1.62934	3.03153	H	2.8608	0.95874	1.51243	H	1.80793	0.35685	2.92625
H	-3.29904	1.35638	0.88329	H	1.86117	-1.60737	3.5448	H	-1.1925	-1.7539	3.00058
H	-2.71601	1.59663	2.49803	H	3.00014	-1.19914	2.27807	H	0.3185	-1.38633	3.76922
H	-0.94244	0.96437	0.12743	H	0.18442	-0.47947	1.66688	H	-0.63629	-0.12681	1.18576
H	-1.99003	4.97483	0.60276	H	0.74564	1.38641	5.33353	H	-2.06169	1.60673	4.70723
H	-2.85204	3.74205	1.66358	H	2.11987	0.29083	4.76777	H	-1.11717	0.03498	4.86735
H	3.22743	3.38789	-0.8292	H	-1.72757	4.03318	1.41068	H	-0.43406	4.71142	0.33105
H	2.42875	4.23224	0.48136	H	-0.33468	4.01211	2.47438	H	-0.00439	4.45661	2.00996
H	3.71482	3.07333	0.85285	H	-0.15341	4.62502	0.82828	H	1.27952	4.65378	0.80643
H	-0.96427	-2.5279	-3.85537	H	-2.45021	-3.47159	-1.47141	H	-2.66845	-1.83775	-3.41455
H	0.61934	-1.83196	-4.16064	H	-3.36045	-2.16992	-0.74106	H	-2.47197	-0.14954	-3.85741
H	-0.78183	-0.76964	-3.91309	H	-1.97951	-2.83369	0.12439	H	-3.26955	-0.57647	-2.32958
H	-2.79938	-3.62189	0.41661	H	2.95511	-2.40517	-1.42822	H	0.41138	-4.49557	-0.90908
H	-2.29673	-3.14815	2.02505	H	3.33079	-1.48975	0.00565	H	1.79412	-4.00191	0.04423
H	-3.98695	-2.91458	1.51896	H	3.62641	-3.23915	-0.01446	H	0.56789	-5.07832	0.75348
H	-3.82166	-1.32094	3.16299	H	-0.42985	-2.49007	1.75305	H	-0.30267	-3.93585	3.09297
H	-0.13276	-4.28615	-2.76975	H	-3.13965	-1.03253	-4.39033	H	-0.76817	-2.48106	-4.39079
H	1.26802	-4.77136	-1.80494	H	-3.82719	-1.58827	-2.86146	H	0.80902	-1.83665	-4.86683
H	-0.21748	-4.31203	-0.97485	H	-3.17805	0.07952	-3.0224	H	0.68755	-2.77254	-3.37801
H	6.54836	-0.74282	1.03015	H	0.05581	5.72005	-2.33366	H	4.00023	4.38388	-3.09046
H	5.79726	0.51543	2.03367	H	0.59642	4.48274	-3.52026	H	4.06111	4.47558	-1.31775
H	5.13393	-1.15523	2.02018	H	-0.90029	5.39872	-3.79443	H	4.34797	2.91327	-2.15929
	6b8				6b9				6b10		
C	0.5327	1.55963	2.29122	C	-0.33868	2.43984	0.91948	C	-0.4255	2.51863	0.95964
C	1.34888	0.23819	1.99353	C	-0.90195	2.31994	-0.55039	C	-0.9959	2.37066	-0.50351
C	0.97795	-0.3402	0.63024	C	-0.70559	0.98843	-1.30546	C	-0.79419	1.03114	-1.24154
C	-1.00893	1.34567	2.39991	C	1.20505	2.2815	1.01675	C	1.12104	2.37394	1.04751
C	-0.6178	-2.26108	-3.0927	C	0.40981	-3.42235	-1.84263	C	0.33077	-3.40503	-1.68969
C	-1.36706	-1.04128	-3.60617	C	0.87055	-3.92839	-0.48898	C	0.84079	-3.86618	-0.33715
C	-1.84567	-0.33925	-2.33248	C	1.52221	-2.69606	0.14727	C	1.45251	-2.59885	0.2617
C	-2.03381	1.197	-2.50271	C	1.44766	-2.67765	1.70463	C	1.43138	-2.56778	1.81649

C	1.69487	-1.58506	0.11439	C	-1.58623	-0.15844	-0.80323	C	-1.6914	-0.11523	-0.75624
C	1.36774	-1.91743	-1.38798	C	-1.40753	-1.52023	-1.54695	C	-1.49787	-1.4754	-1.51646
C	-0.17867	-1.8744	-1.67676	C	0.10193	-1.94817	-1.59184	C	0.00288	-1.92367	-1.48764
C	-1.85441	1.23219	1.1087	C	1.80859	0.87282	1.15677	C	1.73198	0.97039	1.2082
C	-2.45	2.03503	-1.25826	C	1.9144	-1.37127	2.40768	C	1.86416	-1.24509	2.50917
C	-1.42495	2.11795	-0.08216	C	1.06494	-0.10185	2.09543	C	1.0061	0.01525	2.17994
C	-1.12775	3.50129	0.47128	C	0.55367	0.67806	3.28782	C	0.49817	0.81663	3.35958
C	-1.93666	4.57349	0.41211	C	1.13636	0.77439	4.49484	C	1.08226	0.93225	4.56385
C	0.18381	3.68238	1.1766	C	-0.7343	1.41128	3.08354	C	-0.79244	1.54337	3.1448
O	0.89027	2.52707	1.26339	O	-1.07704	1.52751	1.7743	O	-1.1433	1.60812	1.83269
O	0.61952	4.75675	1.56158	O	-1.45756	1.78325	3.9959	O	-1.50803	1.95227	4.04648
C	1.03885	2.11551	3.6418	C	-0.68849	3.87332	1.40823	C	-0.78524	3.95674	1.42745
C	2.10447	-0.93135	-2.3171	C	-2.27146	-2.55976	-0.80465	C	-2.43938	-2.47974	-0.82302
C	-3.12418	-0.99309	-1.7888	C	2.97738	-2.54452	-0.32413	C	2.87214	-2.36064	-0.27234
O	-0.75363	-0.57273	-1.43344	O	0.77571	-1.59083	-0.37713	O	0.58902	-1.56383	-0.22359
O	-0.83913	1.78544	-3.03675	O	0.10968	-2.96346	2.13972	O	0.12196	-2.89684	2.3036
O	1.76547	-3.27661	-1.68319	O	-1.80365	-1.45085	-2.93431	O	-1.9567	-1.20805	-2.85722
C	3.13968	-3.59939	-1.52365	C	-3.11887	-1.00076	-3.22105	C	-1.90266	-2.26381	-3.8002
O	1.06301	-0.74082	3.01443	O	-0.26766	3.35089	-1.35695	O	-0.36017	3.38594	-1.3302
O	3.25873	-1.42518	3.04752	O	-2.19644	3.6479	-2.57545	O	-2.34215	3.82697	-2.41298
C	2.10802	-1.49383	3.45254	C	-1.03283	3.92515	-2.32546	C	-1.14966	4.02428	-2.23671
C	1.64032	-2.44292	4.5128	C	-0.23422	4.96068	-3.05586	C	-0.33676	5.0238	-3.00099
H	2.41562	0.49879	1.97656	H	-1.98295	2.51062	-0.49352	H	-2.07754	2.55524	-0.44677
H	-0.06406	-0.63843	0.71513	H	0.35414	0.72948	-1.29714	H	0.2628	0.76354	-1.21589
H	1.10194	0.44869	-0.11257	H	-0.93527	1.14716	-2.36711	H	-1.00467	1.19087	-2.30763
H	-1.42638	2.19978	2.9536	H	1.54914	2.84088	1.89937	H	1.46844	2.95142	1.91702
H	-1.22354	0.47783	3.03627	H	1.69114	2.78879	0.17388	H	1.59786	2.86821	0.19185
H	-1.2909	-3.12559	-3.04463	H	1.23781	-3.50274	-2.55805	H	1.13256	-3.49854	-2.43287
H	0.20375	-2.54749	-3.75515	H	-0.41566	-3.99702	-2.26882	H	-0.49305	-4.0184	-2.05909
H	-0.65656	-0.41814	-4.16131	H	-0.00574	-4.26209	0.07575	H	-0.01653	-4.21687	0.24779
H	-2.1808	-1.30049	-4.29051	H	1.54996	-4.78314	-0.56592	H	1.55006	-4.69631	-0.41076
H	-2.80871	1.35306	-3.264	H	2.06798	-3.50163	2.07975	H	2.09255	-3.3629	2.18264
H	1.42507	-2.44848	0.73567	H	-2.63222	0.16477	-0.86811	H	-2.73336	0.20957	-0.88492
H	2.77645	-1.45099	0.23497	H	-1.40802	-0.32687	0.25907	H	-1.5528	-0.27859	0.31417
H	-0.6581	-2.5737	-0.97789	H	0.58235	-1.37514	-2.39644	H	0.53722	-1.35558	-2.26031
H	-1.90503	0.18658	0.79807	H	1.92224	0.43381	0.16402	H	1.82386	0.50709	0.22415
H	-2.88928	1.4823	1.38404	H	2.83743	1.00404	1.52101	H	2.76702	1.1114	1.54982
H	-2.61213	3.04575	-1.65247	H	1.86268	-1.58498	3.48221	H	1.79951	-1.45106	3.58472
H	-3.42591	1.69454	-0.89435	H	2.973	-1.19247	2.19009	H	2.92279	-1.05318	2.30153
H	-0.48471	1.74783	-0.48804	H	0.17208	-0.46615	1.58913	H	0.10876	-0.35603	1.6874
H	-1.6432	5.5303	0.8391	H	0.68539	1.35774	5.29501	H	0.63183	1.52575	5.35661
H	-2.91582	4.53891	-0.05109	H	2.08044	0.29291	4.72206	H	2.02311	0.44795	4.79902
H	2.11761	2.3056	3.6031	H	-1.77049	4.04404	1.36527	H	-1.86948	4.11479	1.39425
H	0.56767	3.07588	3.8792	H	-0.39789	4.02171	2.45452	H	-0.48354	4.12808	2.46701
H	0.83477	1.42787	4.46883	H	-0.1888	4.64557	0.81447	H	-0.30096	4.72373	0.81444
H	2.08102	-1.27121	-3.35766	H	-2.36663	-3.49065	-1.37182	H	-2.44097	-3.46192	-1.30169
H	3.16268	-0.84573	-2.04664	H	-3.29346	-2.19303	-0.65591	H	-3.47669	-2.12707	-0.86766
H	1.68112	0.07519	-2.27456	H	-1.86873	-2.78266	0.18745	H	-2.17095	-2.62135	0.22843
H	-3.01636	-2.07735	-1.67944	H	3.05169	-2.54895	-1.41691	H	2.89973	-2.34976	-1.36705
H	-3.37323	-0.62114	-0.79117	H	3.40355	-1.5875	-0.00756	H	3.26089	-1.38679	0.0392
H	-3.9749	-0.80443	-2.45179	H	3.60644	-3.35216	0.06384	H	3.56086	-3.13606	0.07847
H	-0.09535	1.38025	-2.55435	H	0.09442	-2.93546	3.11165	H	-0.50982	-2.3452	1.80797
H	3.76917	-3.00997	-2.1944	H	-3.27578	-1.09811	-4.29936	H	-2.35262	-1.90461	-4.73041
H	3.46188	-3.47775	-0.48682	H	-3.87506	-1.60803	-2.71882	H	-0.86895	-2.53989	-4.02049
H	3.26694	-4.65296	-1.78921	H	-3.23983	0.05486	-2.96663	H	-2.4734	-3.13395	-3.46916
H	0.89872	-3.12822	4.09485	H	0.09088	5.73731	-2.35899	H	0.07227	5.77029	-2.31551
H	2.4903	-3.02686	4.87724	H	0.62646	4.49172	-3.53922	H	0.46608	4.51423	-3.53967
H	1.21861	-1.88322	5.35149	H	-0.85813	5.42299	-3.82612	H	-0.97669	5.53166	-3.72822

Detailed DP4+ probability for compound **6**. Isomer 1 is 1S*,3R*,4R*,7S*,8R*,11S*,12R*, isomer 2 is 1S*,3R*,4R*,7S*,8R*,11S*,12S*.

Functional	Solvent?	Basis Set	Type of Data
B3LYP	PCM	6-311+G(d,p)	Unscaled Shifts

	Isomer 1	Isomer 2	Isomer 3	Isomer 4	Isomer 5	Isomer 6
sDP4+ (H data)	0.00%	100.00%	–	–	–	–
sDP4+ (C data)	0.42%	99.58%	–	–	–	–
sDP4+ (all data)	0.00%	100.00%	–	–	–	–
uDP4+ (H data)	0.00%	100.00%	–	–	–	–
uDP4+ (C data)	0.16%	99.84%	–	–	–	–
uDP4+ (all data)	0.00%	100.00%	–	–	–	–
DP4+ (H data)	0.00%	100.00%	–	–	–	–
DP4+ (C data)	0.00%	100.00%	–	–	–	–
DP4+ (all data)	0.00%	100.00%	–	–	–	–

Table S11.Calculation process of 7

Important thermodynamic parameters (a.u.) of the optimized **7** with simplified structures at B3LYP/6-31+G(d,p) level in the gas phase.

NO.	E+ZPE	G	P%	NO.	E+ZPE	G	P%	NO.	E+ZPE	G	P%
7a1	-1384.220445	-1383.753365	21.80%	7b1	-1384.221153	-1383.756012	49.90%	7d1	-1384.218066	-1383.75609	23.40%
7a2	-1384.220446	-1383.753347	21.39%	7b2	-1384.218867	-1383.754344	8.52%	7d2	-1384.218067	-1383.756065	22.78%
7a3	-1384.217076	-1383.752834	12.42%	7b3	-1384.218867	-1383.754362	8.68%	7d3	-1384.218066	-1383.756086	23.29%
7a4	-1384.219434	-1383.75306	15.78%	7b4	-1384.218792	-1383.755165	20.33%	7d4	-1384.216766	-1383.754152	3.00%
7a5	-1384.217076	-1383.752836	12.44%	7b5	-1384.218745	-1383.752709	1.51%	7d5	-1384.217137	-1383.755065	7.89%
7a6	-1384.215972	-1383.752505	8.76%	7b6	-1384.218867	-1383.754341	8.49%	7d6	-1384.215963	-1383.75442	3.99%
7a7	-1384.214699	-1383.751697	3.72%	7c1	-1384.226185	-1383.762282	98.61%	7d7	-1384.216712	-1383.755046	7.74%
7a8	-1384.215202	-1383.75169	3.69%	7c2	-1384.221818	-1383.758246	1.39%	7d8	-1384.217137	-1383.755066	7.91%

Optimized Z-Matrixes of **7** with simplified structures in the Gas Phase (Å) at B3LYP/6-31+G(d,p) level.

7a1				7a2				7a3			
C	-2.7289	1.57457	-0.39096	C	-1.58621	2.64086	0.18945	C	-1.8366	2.63716	-0.45193
C	-2.20727	1.18609	1.01341	C	-0.83041	2.25958	1.48403	C	-1.81736	2.14179	1.01218
C	-1.24867	2.14031	1.72024	C	0.6509	2.60975	1.59258	C	-0.85659	2.81109	1.98375
C	-3.35859	0.39317	-1.16463	C	-2.92673	1.87037	0.08388	C	-2.44886	1.56541	-1.38305
C	2.61175	-0.52616	1.76457	C	2.61648	-1.59968	0.89074	C	2.22669	-0.63871	1.76734
C	2.80231	-1.82036	1.00153	C	1.93419	-2.8613	0.4043	C	2.14658	-1.81785	0.80695
C	1.38967	-2.15145	0.47546	C	0.43025	-2.519	0.46919	C	0.64234	-2.00883	0.53367
C	1.25479	-1.87035	-1.06066	C	-0.18726	-2.31351	-0.95698	C	0.26478	-2.10953	-0.97731
C	0.18078	1.59295	1.75337	C	1.54236	1.41359	1.24791	C	0.44334	2.01776	2.12789
C	0.49055	0.5441	2.85947	C	1.76756	0.37455	2.38146	C	0.39041	0.75726	3.03174
C	1.21664	-0.71695	2.32553	C	1.61238	-1.09312	1.90706	C	0.82294	-0.56093	2.33699
C	-2.50185	-0.87799	-1.295	C	-2.81056	0.33976	-0.02468	C	-1.64281	0.26123	-1.50262
C	-0.1964	-1.91141	-1.60938	C	-1.60821	-1.69122	-0.96511	C	0.59588	-0.90859	-1.8897
C	-1.00216	-0.60261	-1.42318	C	-1.63051	-0.14346	-0.88231	C	-0.11172	0.42602	-1.58218
C	-0.81527	0.46773	-2.46264	C	-1.62613	0.64355	-2.18041	C	0.24015	1.56596	-2.5283
C	-0.65962	0.27854	-3.78483	C	-2.04492	0.2094	-3.38226	C	0.84218	1.45659	-3.72573
C	-0.76297	1.8679	-1.99713	C	-1.114	2.05044	-2.12695	C	-0.11133	2.95425	-2.08909
O	-1.70286	2.29208	-1.12979	O	-0.66531	2.40973	-0.90395	O	-0.47544	2.98366	-0.78803
O	0.09682	2.63775	-2.41312	O	-0.98187	2.77047	-3.10545	O	0.00584	3.95907	-2.77128
C	-3.83591	2.621	-0.16281	C	-1.87679	4.14591	0.23195	C	-2.68261	3.91393	-0.50637
C	1.31113	1.19735	3.97974	C	3.15115	0.58782	3.0112	C	1.26715	0.98865	4.27314
C	1.03744	-3.61112	0.79715	C	-0.32452	-3.61633	1.23569	C	0.14634	-3.3064	1.2212
O	-2.55736	0.13704	1.56296	O	-1.39294	1.66746	2.41064	O	-2.54825	1.22696	1.40442
O	-0.71785	0.10198	3.49299	O	0.83938	0.57622	3.45485	O	-0.93547	0.55539	3.54043
O	0.48018	-1.31799	1.23646	O	0.33499	-1.30663	1.2617	O	-0.00694	-0.89546	1.19897
O	1.85611	-0.58758	-1.35597	O	0.7064	-1.49266	-1.74698	O	0.97572	-3.25673	-1.52494
O	2.51159	-1.33732	-3.42455	O	0.09965	-2.61667	-3.65671	O	-0.75484	-3.69249	-2.97387
C	2.4562	-0.46302	-2.57084	C	0.74804	-1.75467	-3.08176	C	0.34037	-3.95951	-2.50248
C	3.08932	0.88799	-2.70841	C	1.71756	-0.83085	-3.75268	C	1.18848	-5.11708	-2.93138
H	-1.63358	2.35168	2.72363	H	0.85391	2.99747	2.59669	H	-1.36086	2.95399	2.94561

H	-1.25029	3.10006	1.19313	H	0.86795	3.43389	0.90494	H	-0.63194	3.81907	1.61833
H	-4.30565	0.09689	-0.69437	H	-3.56747	2.10479	0.9442	H	-3.4669	1.31449	-1.05711
H	-3.61469	0.73528	-2.17672	H	-3.47656	2.22846	-0.79737	H	-2.56229	1.98366	-2.39262
H	3.37006	-0.38131	2.53887	H	2.73368	-0.89835	0.05769	H	2.99173	-0.77792	2.53644
H	2.65586	0.32261	1.07411	H	3.6025	-1.79663	1.32014	H	2.47995	0.26235	1.1992
H	3.15463	-2.5941	1.69559	H	2.17984	-3.68606	1.08538	H	2.59007	-2.71337	1.258
H	3.56041	-1.74126	0.21615	H	2.27677	-3.16972	-0.58831	H	2.72821	-1.6108	-0.09628
H	1.8324	-2.65882	-1.56293	H	-0.24659	-3.31371	-1.40804	H	-0.81493	-2.29711	-1.03148
H	0.86457	2.44419	1.87297	H	2.51796	1.79993	0.92338	H	1.19988	2.70838	2.52479
H	0.43268	1.19244	0.7671	H	1.14771	0.91825	0.35691	H	0.82389	1.75623	1.13899
H	1.23973	-1.4706	3.12563	H	1.61787	-1.74582	2.79178	H	0.68364	-1.36334	3.07342
H	-2.863	-1.45791	-2.15477	H	-3.75563	-0.05455	-0.42095	H	-2.00455	-0.28227	-2.38559
H	-2.672	-1.51432	-0.41723	H	-2.70918	-0.08087	0.98376	H	-1.89416	-0.36729	-0.64121
H	-0.14604	-2.16531	-2.67416	H	-2.11962	-2.02931	-1.87166	H	1.68242	-0.753	-1.89937
H	-0.73203	-2.74856	-1.15021	H	-2.19433	-2.12211	-0.14699	H	0.35011	-1.20656	-2.91781
H	-0.66914	-0.18983	-0.4744	H	-0.72223	0.12313	-0.34331	H	0.25978	0.71611	-0.59917
H	-0.50817	1.11397	-4.46531	H	-2.00655	0.84629	-4.26399	H	1.05638	2.3316	-4.33662
H	-0.67412	-0.7077	-4.23585	H	-2.44541	-0.78457	-3.54421	H	1.13775	0.50376	-4.14895
H	-3.44537	3.51568	0.33571	H	-0.95273	4.73282	0.27396	H	-2.25167	4.70485	0.11753
H	-4.25376	2.95922	-1.1182	H	-2.40334	4.46652	-0.67412	H	-2.72518	4.31327	-1.52582
H	-4.64969	2.22291	0.45322	H	-2.48585	4.4137	1.102	H	-3.70639	3.73254	-0.16192
H	0.75229	2.02231	4.43718	H	3.22669	1.59095	3.44727	H	0.90831	1.85306	4.84443
H	2.26605	1.59033	3.61689	H	3.95962	0.46973	2.28295	H	2.31547	1.16092	4.00935
H	1.51478	0.4802	4.78342	H	3.31925	-0.11865	3.8324	H	1.21741	0.1307	4.95375
H	1.15564	-3.81413	1.86821	H	0.12357	-3.78299	2.22244	H	0.36912	-3.32125	2.29257
H	-0.00978	-3.8346	0.57256	H	-1.36297	-3.3332	1.43184	H	-0.94329	-3.39015	1.13584
H	1.67404	-4.30883	0.24385	H	-0.31437	-4.56563	0.69072	H	0.5994	-4.20204	0.78402
H	-1.25711	-0.35734	2.81155	H	-0.06729	0.44886	3.10166	H	-1.50779	0.30414	2.78174
H	4.15983	0.80889	-2.50614	H	2.72774	-1.02502	-3.38396	H	0.66054	-5.68789	-3.70063
H	2.91995	1.26363	-3.7209	H	1.69675	-1.00568	-4.83209	H	2.12902	-4.75159	-3.35097
H	2.64192	1.59809	-2.0075	H	1.43339	0.20774	-3.56551	H	1.37648	-5.77462	-2.07885
7a4				7a5				7a6			
C	-0.43447	2.87346	-0.91316	C	0.40745	2.60919	-1.96839	C	1.37322	2.78755	0.09061
C	0.21891	2.36161	0.39792	C	0.91552	2.64343	-0.50764	C	1.90552	1.48884	0.7559
C	1.52757	1.58611	0.2805	C	2.40385	2.45007	-0.25976	C	2.51257	0.43895	-0.16661
C	-1.97403	2.73636	-0.8908	C	-1.10196	2.31896	-2.08211	C	0.15248	3.35556	0.83639
C	1.8428	-1.90131	1.69455	C	1.70799	-1.68853	1.64487	C	0.19646	-3.23695	1.74826
C	0.78476	-2.92382	1.31658	C	0.35849	-2.38519	1.73721	C	-1.09903	-3.01366	1.00073
C	-0.49639	-2.08493	1.09158	C	-0.67836	-1.24495	1.68547	C	-1.31462	-1.50332	1.12127
C	-1.01401	-2.18959	-0.38399	C	-1.75609	-1.40011	0.56943	C	-2.20678	-0.94773	-0.03461
C	2.3068	1.35446	1.57496	C	2.72008	1.06488	0.30633	C	3.08951	-0.78282	0.55179
C	1.69459	0.50571	2.7166	C	2.41335	0.84109	1.81295	C	2.21488	-2.06273	0.61815
C	1.00049	-0.83295	2.35975	C	1.38994	-0.28396	2.11907	C	0.96828	-1.92954	1.54121
C	-2.50305	1.33333	-0.54491	C	-1.59989	1.05993	-1.35798	C	-1.00402	2.36476	1.01996
C	-2.14893	-1.19429	-0.74973	C	-1.25799	-1.43541	-0.89143	C	-2.50275	0.57487	0.00444
C	-1.62961	0.21356	-1.10875	C	-0.6462	-0.13321	-1.44371	C	-1.25591	1.47208	-0.20029
C	-1.3573	0.49715	-2.5591	C	-0.13322	-0.24879	-2.85446	C	-1.23205	2.33649	-1.42968
C	-2.10048	0.08448	-3.60052	C	-0.66911	-0.99401	-3.83751	C	-2.28739	2.94795	-1.99447
C	-0.13526	1.26568	-2.8814	C	1.11151	0.47182	-3.19084	C	0.07692	2.49619	-2.10468
O	0.1984	2.30835	-2.09386	O	1.24912	1.74651	-2.78046	O	1.18492	2.62732	-1.3442
O	0.56497	0.96216	-3.84188	O	1.99911	-0.07161	-3.83975	O	0.15971	2.49339	-3.32854
C	-0.08585	4.3704	-0.99437	C	0.65699	4.01968	-2.53469	C	2.51783	3.81171	0.1978
C	2.81924	0.22629	3.73572	C	3.72525	0.54607	2.55772	C	1.84984	-2.54516	-0.79287
C	-1.60215	-2.53473	2.06635	C	-1.41451	-1.14665	3.04277	C	-1.91504	-1.15745	2.49568
O	-0.30893	2.58544	1.48989	O	0.15492	2.85468	0.44164	O	1.90043	1.35198	1.98217
O	0.7558	1.30565	3.45246	O	1.90394	2.04688	2.40167	O	3.06871	-3.08847	1.17281
O	-0.12471	-0.72232	1.45818	O	0.09416	-0.0293	1.53095	O	0.02286	-0.96121	1.05053
O	0.10474	-2.01389	-1.2884	O	-2.45393	-2.65353	0.81352	O	-1.56876	-1.25125	-1.30121
O	-0.91633	-3.35683	-2.84586	O	-4.39435	-1.78922	-0.06493	O	-3.6079	-1.39334	-2.34681
C	0.01874	-2.6554	-2.48657	C	-3.76827	-2.69699	0.46173	C	-2.3865	-1.43069	-2.3726
C	1.26138	-2.42738	-3.29147	C	-4.34442	-4.03583	0.80629	C	-1.56769	-1.67735	-3.60199

H	2.17769	2.17258	-0.3812	H	2.76012	3.25343	0.39436	H	3.33637	0.94004	-0.68984
H	1.3501	0.63112	-0.21753	H	2.94408	2.58571	-1.20271	H	1.77427	0.15196	-0.91709
H	-2.40921	3.43683	-0.16607	H	-1.67983	3.16678	-1.69064	H	0.44392	3.70789	1.83465
H	-2.37109	3.02499	-1.87323	H	-1.36828	2.23402	-3.14438	H	-0.22116	4.23746	0.29917
H	2.61126	-2.324	2.34679	H	2.03813	-1.6972	0.6012	H	0.72395	-4.13546	1.41611
H	2.32199	-1.5178	0.78697	H	2.47536	-2.18145	2.24819	H	-0.00684	-3.37807	2.81663
H	0.64907	-3.6359	2.1402	H	0.28667	-2.94739	2.67606	H	-1.92852	-3.6034	1.40324
H	1.08716	-3.51861	0.44916	H	0.24397	-3.11839	0.93327	H	-0.95668	-3.31601	-0.04261
H	-1.40219	-3.2112	-0.49971	H	-2.46401	-0.56924	0.67769	H	-3.16649	-1.4799	0.02358
H	2.55725	2.34351	1.98372	H	3.79175	0.89244	0.13596	H	3.42637	-0.52296	1.56359
H	3.25849	0.89343	1.27745	H	2.23527	0.29988	-0.30338	H	4.01729	-1.05759	0.02795
H	0.59184	-1.22844	3.30121	H	1.24025	-0.29194	3.20746	H	1.31249	-1.58324	2.52439
H	-3.53636	1.24163	-0.90323	H	-2.58343	0.79256	-1.76657	H	-1.90832	2.93279	1.27539
H	-2.54945	1.23185	0.54711	H	-1.77409	1.31168	-0.30572	H	-0.78992	1.73274	1.88896
H	-2.70713	-1.61786	-1.5914	H	-0.53873	-2.25617	-1.00859	H	-3.23648	0.77265	-0.78455
H	-2.87223	-1.14067	0.06971	H	-2.10834	-1.72411	-1.5226	H	-3.01451	0.82282	0.94086
H	-0.67376	0.28078	-0.60313	H	0.22041	0.06793	-0.81848	H	-0.40801	0.79907	-0.31534
H	-1.81867	0.31202	-4.62657	H	-0.21349	-1.04383	-4.82459	H	-2.18255	3.53715	-2.90316
H	-3.01026	-0.4916	-3.4712	H	-1.57552	-1.57271	-3.69933	H	-3.28482	2.88564	-1.57297
H	1.00051	4.51824	-1.02318	H	1.72374	4.27233	-2.51903	H	3.4107	3.46066	-0.33322
H	-0.48652	4.81473	-1.91282	H	0.3407	4.07967	-3.58257	H	2.23366	4.76342	-0.266
H	-0.47678	4.92938	-0.13717	H	0.12305	4.78736	-1.96389	H	2.80085	4.00181	1.23889
H	3.23765	1.16425	4.12037	H	3.5473	0.42418	3.63254	H	2.74129	-2.58549	-1.42988
H	3.63566	-0.3574	3.29902	H	4.42433	1.38488	2.45771	H	1.11173	-1.90326	-1.2795
H	2.43351	-0.31482	4.6078	H	4.21509	-0.35818	2.18277	H	1.45437	-3.5665	-0.77506
H	-1.23798	-2.56849	3.09925	H	-0.72006	-1.00604	3.87744	H	-1.32769	-1.57776	3.31861
H	-2.43972	-1.83055	2.07575	H	-2.08182	-0.27709	3.0537	H	-1.92151	-0.07908	2.67323
H	-1.9788	-3.53086	1.81242	H	-2.00707	-2.04265	3.25389	H	-2.93996	-1.53202	2.58526
H	0.09945	1.66033	2.81422	H	0.99875	2.18098	2.03936	H	3.38728	-2.77464	2.03768
H	1.98004	-3.22355	-3.0849	H	-4.25103	-4.21408	1.88055	H	-2.23321	-1.83486	-4.45543
H	1.00551	-2.41779	-4.35438	H	-5.40573	-4.05251	0.54243	H	-0.93686	-0.80844	-3.80612
H	1.70526	-1.45893	-3.04573	H	-3.83089	-4.81674	0.23998	H	-0.95668	-2.57337	-3.46658
7a7				7a8				7b1			
C	-2.51257	1.7671	-0.36648	C	-0.34522	2.38597	-1.97311	C	1.41979	1.9721	1.91676
C	-1.90675	1.51283	1.04022	C	0.70501	2.29435	-0.83084	C	2.14762	0.99774	0.95072
C	-0.56641	2.16639	1.35162	C	1.88502	1.35461	-1.0564	C	2.06467	1.303	-0.53881
C	-3.22538	0.48888	-0.85074	C	-1.76474	2.66412	-1.44435	C	0.75246	1.16745	3.05039
C	1.59676	-1.81969	2.73193	C	1.73881	-0.79077	3.17692	C	0.72734	-3.14662	-1.6101
C	2.00802	-2.29112	1.35658	C	0.57798	-1.7058	2.85405	C	-0.74893	-2.85104	-1.52139
C	0.74504	-2.06362	0.52087	C	-0.40929	-0.77977	2.14127	C	-0.85243	-2.05742	-0.21592
C	1.07587	-1.90742	-0.99532	C	-1.37949	-1.56959	1.20333	C	-2.08782	-1.11002	-0.21676
C	0.00806	1.81883	2.72639	C	2.93836	1.35054	0.05519	C	2.76713	0.28564	-1.43952
C	1.10642	0.72431	2.78815	C	2.90876	0.17741	1.07196	C	1.90389	-0.85045	-2.05845
C	0.58421	-0.7041	2.44816	C	1.70781	0.254	2.05571	C	1.3872	-1.8986	-1.01899
C	-2.28614	-0.713	-1.02807	C	-2.2603	1.68609	-0.37143	C	-0.3244	0.18546	2.56395
C	-0.11499	-1.67344	-1.96468	C	-2.43505	-0.71335	0.45475	C	-2.35314	-0.2735	1.06614
C	-0.93549	-0.38414	-1.69751	C	-1.83827	0.23423	-0.61548	C	-1.23336	0.73056	1.44319
C	-1.22171	0.5157	-2.8836	C	-2.09847	-0.10624	-2.05545	C	-1.67073	2.12169	1.85789
C	-1.26788	0.1408	-4.17362	C	-3.22628	-0.63562	-2.5598	C	-2.86528	2.45383	2.37733
C	-1.4974	1.95916	-2.58226	C	-0.98798	0.12627	-3.00661	C	-0.66823	3.22485	1.69053
O	-1.47711	2.24901	-1.25869	O	-0.25724	1.25483	-2.88603	O	0.50751	2.80857	1.16182
O	-1.61095	2.82538	-3.43689	O	-0.73128	-0.68944	-3.88633	O	-0.91075	4.40505	1.89399
C	-3.52861	2.90627	-0.22611	C	0.0832	3.58663	-2.83729	C	2.48042	2.91144	2.50168
C	2.32637	1.11944	1.94237	C	2.99659	-1.18105	0.35959	C	2.76621	-1.51796	-3.14625
C	-0.24403	-3.22179	0.74962	C	-1.19361	0.06413	3.16204	C	-0.89593	-3.03124	0.97527
O	-2.5243	0.86702	1.89133	O	0.61773	3.00588	0.17232	O	2.81683	0.05372	1.38032
O	1.5504	0.69536	4.16335	O	4.1091	0.30847	1.865	O	0.78508	-0.25327	-2.73254
O	0.19577	-0.83935	1.063	O	0.44575	0.09914	1.37905	O	0.37429	-1.28711	-0.17377
O	1.99893	-0.797	-1.1525	O	-0.59413	-2.29096	0.22073	O	-1.93359	-0.1861	-1.33062
O	3.0486	-1.85005	-2.90326	O	-2.20557	-3.91922	0.06384	O	-4.20306	-0.16336	-1.68131
C	2.9236	-0.90103	-2.14358	C	-1.13702	-3.43374	-0.27747	C	-3.07239	0.20017	-1.96836

C	3.77633	0.32975	-2.17407	C	-0.23141	-4.01562	-1.3186	C	-2.73698	1.14157	-3.08383
H	-0.74791	3.24784	1.31911	H	2.37219	1.70291	-1.97566	H	2.56288	2.27235	-0.66784
H	0.14512	1.92851	0.55959	H	1.5044	0.3506	-1.25043	H	1.02278	1.42908	-0.83797
H	-4.02183	0.20227	-0.15147	H	-1.82239	3.67413	-1.01764	H	1.50428	0.60017	3.61467
H	-3.73102	0.68446	-1.80573	H	-2.47065	2.64696	-2.2855	H	0.29806	1.85544	3.77573
H	1.10305	-2.63426	3.27546	H	2.68483	-1.33154	3.26974	H	1.03738	-3.41706	-2.61954
H	2.45199	-1.51703	3.34255	H	1.57217	-0.29824	4.14248	H	0.96734	-4.0127	-0.98117
H	2.35259	-3.32998	1.34991	H	0.15684	-2.18742	3.74198	H	-1.3642	-3.75631	-1.52861
H	2.83919	-1.66878	1.00784	H	0.93566	-2.49799	2.18679	H	-1.04181	-2.25156	-2.39014
H	1.57519	-2.83638	-1.30415	H	-1.91478	-2.29007	1.83742	H	-2.97066	-1.74177	-0.38698
H	-0.7857	1.5697	3.44203	H	2.9445	2.30133	0.60321	H	3.64232	-0.14745	-0.94021
H	0.44768	2.74205	3.1322	H	3.92129	1.32354	-0.43823	H	3.16254	0.87892	-2.27691
H	-0.30705	-0.89372	3.06016	H	1.71259	1.24811	2.5216	H	2.22343	-2.20169	-0.37791
H	-2.81964	-1.47893	-1.60631	H	-3.35359	1.76466	-0.30726	H	-0.92871	-0.11828	3.42891
H	-2.11069	-1.14803	-0.04098	H	-1.87733	2.00915	0.60392	H	0.18725	-0.71712	2.22451
H	0.32227	-1.62377	-2.96808	H	-3.14021	-1.40908	-0.01189	H	-3.27738	0.28201	0.86797
H	-0.76605	-2.55483	-1.96866	H	-3.03153	-0.15399	1.18365	H	-2.57894	-0.9378	1.90771
H	-0.32958	0.21406	-1.01325	H	-0.75804	0.1764	-0.50692	H	-0.64641	0.8582	0.53222
H	-1.48315	0.85482	-4.96603	H	-3.31758	-0.87486	-3.61729	H	-3.10057	3.48011	2.65232
H	-1.10765	-0.88438	-4.48808	H	-4.0936	-0.84466	-1.94308	H	-3.64474	1.72203	2.558
H	-3.04203	3.82208	0.12949	H	1.0819	3.43012	-3.26207	H	3.25097	2.36237	3.05337
H	-3.98189	3.14788	-1.19404	H	-0.59826	3.71885	-3.6857	H	2.97685	3.48182	1.7078
H	-4.32767	2.65425	0.47931	H	0.10702	4.51773	-2.26054	H	2.02443	3.64458	3.17646
H	2.13286	1.05718	0.86902	H	3.23656	-1.98436	1.06457	H	2.17051	-2.14681	-3.81472
H	3.19001	0.48631	2.1725	H	3.80911	-1.17941	-0.37638	H	3.21964	-0.76678	-3.80438
H	2.6412	2.14409	2.17254	H	2.07041	-1.4477	-0.15553	H	3.57108	-2.11714	-2.70861
H	-0.53261	-3.31289	1.80185	H	-1.74442	0.87279	2.6734	H	0.00812	-3.64615	1.03219
H	-1.17961	-3.0793	0.20552	H	-1.89942	-0.55289	3.72746	H	-0.95262	-2.51383	1.9346
H	0.18716	-4.17593	0.42831	H	-0.53341	0.5632	3.87889	H	-1.76279	-3.69721	0.90703
H	1.88747	1.57881	4.39196	H	4.87449	0.23595	1.26892	H	0.09066	-0.13592	-2.05379
H	4.29625	0.44373	-1.21947	H	0.74084	-4.2472	-0.87636	H	-3.6598	1.47405	-3.56753
H	4.52217	0.23475	-2.96832	H	-0.66883	-4.94123	-1.70334	H	-2.2183	2.01746	-2.68575
H	3.15521	1.20491	-2.38099	H	-0.11957	-3.3113	-2.14676	H	-2.11701	0.63087	-3.82482
		7b2				7b3				7b4	
C	1.19315	2.9942	-0.29851	C	1.37452	2.64261	1.11538	C	1.82027	1.85712	1.6468
C	1.49076	2.53084	-1.74663	C	2.13206	1.82641	0.03714	C	2.6345	0.76574	0.91089
C	0.36203	1.96544	-2.60068	C	1.86897	2.1409	-1.43026	C	3.0594	1.02138	-0.52972
C	2.33976	2.54576	0.64715	C	1.54959	2.06988	2.54192	C	1.30557	1.3292	3.01377
C	-1.69344	-2.58628	-1.33358	C	-0.67258	-2.12607	-2.45831	C	-0.53577	-1.7875	-2.70058
C	-1.02009	-3.53159	-0.36447	C	-0.56598	-3.24189	-1.44607	C	-1.28776	-2.75097	-1.81194
C	0.22729	-2.77172	0.10986	C	0.13606	-2.58848	-0.24407	C	-0.86713	-2.3458	-0.38702
C	0.03998	-2.04455	1.48649	C	-0.88291	-2.14311	0.86292	C	-1.98385	-1.50405	0.32649
C	0.46561	0.43701	-2.63142	C	1.8874	0.88993	-2.31827	C	2.8668	-0.20414	-1.43277
C	-0.84628	-0.34197	-2.35216	C	0.51886	0.18555	-2.56107	C	1.5411	-0.28552	-2.24994
C	-0.52611	-1.80693	-1.92443	C	0.59169	-1.32236	-2.19306	C	0.75581	-1.58578	-1.92524
C	2.38852	1.04356	0.99675	C	1.20104	0.58252	2.72088	C	0.30039	0.16273	2.94462
C	1.18689	-1.05222	1.8541	C	-0.27145	-1.38603	2.07299	C	-1.63031	-0.97917	1.74376
C	1.02393	0.39694	1.31522	C	0.04274	0.11337	1.83585	C	-0.67823	0.24143	1.76045
C	0.19367	1.36054	2.14489	C	-1.0881	1.09137	2.00992	C	-1.28416	1.63249	1.72073
C	-0.04081	1.29218	3.46684	C	-2.07381	1.00277	2.9217	C	-2.52988	1.98041	2.08675
C	-0.41742	2.5204	1.4199	C	-1.15399	2.2556	1.0963	C	-0.39226	2.74183	1.24797
O	-0.10203	2.51387	0.10532	O	-0.00886	2.86888	0.73838	O	0.77216	2.27579	0.74255
O	-1.19238	3.32108	1.9183	O	-2.23645	2.67102	0.69306	O	-0.70499	3.92152	1.21915
C	1.12655	4.52771	-0.34968	C	2.00119	4.05012	1.10341	C	2.73872	3.06169	1.88278
C	-1.75941	-0.31894	-3.57936	C	0.10328	0.36924	-4.02624	C	1.86211	-0.21294	-3.749
C	1.43004	-3.72399	0.14699	C	1.20156	-3.53532	0.31954	C	-0.49725	-3.59156	0.42673
O	2.63007	2.6084	-2.22093	O	3.01293	1.01946	0.35229	O	3.01065	-0.25808	1.49118
O	-1.56775	0.3165	-1.30308	O	-0.50251	0.81153	-1.77382	O	0.70574	0.84227	-1.9678
O	0.48892	-1.78352	-0.89802	O	0.82039	-1.43441	-0.77214	O	0.33992	-1.56189	-0.54277
O	-1.2209	-1.33445	1.46589	O	-1.9004	-1.31996	0.24487	O	-2.32728	-0.38625	-0.52664
O	-1.61282	-1.89542	3.65881	O	-3.48172	-2.12643	1.70155	O	-4.50667	-0.45484	0.19224

C	-1.94682	-1.3492	2.61788	C	-3.15692	-1.41676	0.76052	C	-3.62228	0.02796	-0.49962
C	-3.23103	-0.60341	2.42057	C	-4.10155	-0.55603	-0.02083	C	-3.81359	1.16594	-1.45465
H	0.48554	2.36698	-3.61384	H	2.68719	2.80546	-1.73613	H	4.13019	1.25773	-0.47511
H	-0.60209	2.34192	-2.25154	H	0.94884	2.71557	-1.55935	H	2.581	1.9136	-0.9401
H	3.3142	2.83538	0.2331	H	2.58602	2.20368	2.87909	H	2.14998	1.01971	3.64364
H	2.25646	3.09997	1.59254	H	0.92606	2.65216	3.23403	H	0.82276	2.15342	3.55671
H	-2.39285	-1.94445	-0.78957	H	-1.5777	-1.53888	-2.27352	H	-1.09986	-0.85372	-2.79614
H	-2.27104	-3.10976	-2.10166	H	-0.72503	-2.49485	-3.48714	H	-0.3694	-2.18619	-3.70545
H	-0.74162	-4.44262	-0.90912	H	0.04938	-4.04355	-1.87359	H	-0.96355	-3.77331	-2.04386
H	-1.67637	-3.84284	0.45437	H	-1.53533	-3.68536	-1.19755	H	-2.36968	-2.72029	-1.97534
H	0.0114	-2.83536	2.24804	H	-1.34026	-3.06765	1.24216	H	-2.85269	-2.17005	0.42349
H	1.23061	0.14216	-1.90568	H	2.61807	0.16547	-1.93798	H	3.00592	-1.12839	-0.85825
H	0.86943	0.12193	-3.60295	H	2.30162	1.18901	-3.29113	H	3.70793	-0.21028	-2.14008
H	-0.09467	-2.35703	-2.77009	H	1.44141	-1.79676	-2.7007	H	1.4067	-2.46009	-2.05841
H	3.06942	0.91333	1.84811	H	0.97918	0.39738	3.78033	H	-0.25145	0.11767	3.89268
H	2.84343	0.50282	0.15671	H	2.08902	-0.02157	2.49574	H	0.85987	-0.77892	2.87739
H	1.2744	-1.03297	2.94569	H	-0.96645	-1.48574	2.91422	H	-2.56418	-0.73169	2.25852
H	2.14376	-1.46267	1.51709	H	0.63118	-1.90909	2.40438	H	-1.20717	-1.79996	2.33187
H	0.52972	0.28552	0.35295	H	0.37577	0.18544	0.80396	H	-0.07541	0.13837	0.8626
H	-0.64927	2.03752	3.97592	H	-2.86626	1.74675	2.97692	H	-2.87125	3.01237	2.03036
H	0.35876	0.5066	4.09762	H	-2.12947	0.19713	3.64526	H	-3.25484	1.2679	2.46289
H	0.32107	4.8595	-1.01556	H	3.07757	4.01769	1.3047	H	3.61752	2.78737	2.47609
H	0.91019	4.94908	0.63817	H	1.85459	4.54279	0.13498	H	3.08923	3.48764	0.93591
H	2.06468	4.96281	-0.71098	H	1.52481	4.69479	1.85125	H	2.20438	3.86484	2.40259
H	-2.71634	-0.80896	-3.36777	H	-0.90755	-0.01208	-4.20758	H	0.9496	-0.15721	-4.35263
H	-2.00076	0.70998	-3.8693	H	0.07324	1.43354	-4.28826	H	2.42812	0.69765	-3.97887
H	-1.29607	-0.81946	-4.43564	H	0.79484	-0.13675	-4.70774	H	2.44698	-1.07793	-4.07835
H	1.55716	-4.22634	-0.81958	H	1.87574	-3.87883	-0.47426	H	0.23938	-4.20233	-0.10889
H	2.37067	-3.19344	0.31987	H	1.84798	-3.03294	1.04558	H	-0.01664	-3.32715	1.37376
H	1.30964	-4.49025	0.91936	H	0.74975	-4.41377	0.79071	H	-1.37462	-4.21269	0.6322
H	-1.24432	-0.03784	-0.45591	H	-0.71098	0.20512	-1.03858	H	0.1906	0.61162	-1.1723
H	-3.85982	-1.13631	1.70316	H	-4.32678	-1.03385	-0.97709	H	-3.65406	0.81878	-2.47844
H	-3.76099	-0.53619	3.37485	H	-5.02682	-0.42645	0.54744	H	-4.83526	1.54582	-1.36542
H	-3.02422	0.41075	2.06962	H	-3.66468	0.43184	-0.18464	H	-3.12182	1.97668	-1.21223
7b5				7b6				7c1			
C	1.72068	2.30317	1.5741	C	1.37701	2.69982	1.07521	C	-3.0729	0.71469	0.98757
C	2.39202	1.94371	0.22077	C	2.24273	1.91143	0.05938	C	-3.06892	-0.50941	0.03916
C	1.49381	1.76097	-0.99021	C	2.02788	2.14078	-1.43094	C	-2.3439	-1.77164	0.48242
C	1.87687	1.09436	2.51844	C	1.49912	2.13869	2.51209	C	-2.41173	1.89946	0.25238
C	-0.52264	-2.03369	-2.76223	C	-0.72016	-2.06315	-2.41558	C	1.54707	-1.82376	-2.16783
C	-0.88793	-2.99208	-1.65434	C	-0.62056	-3.23541	-1.46758	C	2.7005	-1.50526	-1.24637
C	-0.42568	-2.28736	-0.36147	C	0.12511	-2.66051	-0.25398	C	2.00705	-0.85462	-0.03504
C	-1.63177	-1.76762	0.47493	C	-0.85407	-2.17994	0.87724	C	2.0453	0.70756	-0.13365
C	2.09076	0.84109	-2.06155	C	1.98487	0.81884	-2.21061	C	-2.02137	-2.72937	-0.67037
C	1.0396	0.03598	-2.87169	C	0.56832	0.21226	-2.43263	C	-0.97948	-2.26424	-1.72843
C	0.69531	-1.32424	-2.19262	C	0.57987	-1.31786	-2.1507	C	0.47524	-2.24693	-1.17497
C	1.00949	-0.12364	2.16049	C	1.20432	0.63722	2.675	C	-0.89582	1.76735	0.04609
C	-1.33572	-1.12595	1.85961	C	-0.19699	-1.37861	2.03336	C	1.42236	1.48908	1.05239
C	-0.47783	0.1696	1.85212	C	0.09295	0.119	1.75426	C	-0.09202	1.29333	1.27351
C	-0.94085	1.28776	2.76894	C	-1.06093	1.07829	1.86667	C	-0.65918	1.91675	2.53527
C	-1.68268	1.15029	3.88133	C	-2.0619	1.01154	2.7635	C	-0.08753	2.88847	3.267
C	-0.51654	2.67925	2.40096	C	-1.12209	2.20488	0.90863	C	-1.97631	1.3839	3.01726
O	0.35061	2.70606	1.3578	O	0.00887	2.88661	0.63297	O	-2.43993	0.37555	2.23775
O	-0.97409	3.69295	2.90552	O	-2.19474	2.54141	0.41192	O	-2.53183	1.73476	4.04654
C	2.46262	3.51937	2.14782	C	1.95556	4.12884	1.08903	C	-4.54142	1.04033	1.29744
C	1.57237	-0.22406	-4.28712	C	0.10152	0.5085	-3.86374	C	-1.063	-3.21903	-2.93212
C	0.44718	-3.25637	0.44659	C	1.12046	-3.69396	0.2885	C	2.63786	-1.36522	1.26558
O	3.61605	1.80935	0.1304	O	3.17751	1.19727	0.44139	O	-3.66738	-0.48079	-1.04441
O	-0.14316	0.83349	-3.02807	O	-0.34705	0.86517	-1.5504	O	-1.30499	-0.96246	-2.22332
O	0.38283	-1.16523	-0.79428	O	0.89779	-1.55826	-0.7639	O	0.63918	-1.32964	-0.073
O	-2.35107	-0.79897	-0.33496	O	-1.92782	-1.40871	0.29173	O	3.44082	1.10891	-0.21482

O	-4.35892	-1.49753	0.53375	O	-3.40656	-2.21096	1.85745	O	2.87994	2.94593	-1.47837
C	-3.70448	-0.77215	-0.20007	C	-3.15001	-1.52375	0.87922	C	3.70872	2.2376	-0.92667
C	-4.28351	0.28598	-1.08775	C	-4.16056	-0.7156	0.12434	C	5.18375	2.49756	-0.9329
H	1.28684	2.75151	-1.4102	H	2.89253	2.72813	-1.76531	H	-3.01398	-2.28063	1.18582
H	0.55046	1.34953	-0.64535	H	1.1502	2.76313	-1.62272	H	-1.43795	-1.52562	1.03062
H	2.92592	0.7712	2.55094	H	2.50983	2.31472	2.90379	H	-2.59996	2.82716	0.80982
H	1.6368	1.39706	3.54672	H	0.8174	2.69626	3.16872	H	-2.88108	2.05096	-0.72859
H	-1.35902	-1.34538	-2.9232	H	-1.60191	-1.46272	-2.1781	H	1.25395	-0.92109	-2.71559
H	-0.31665	-2.54241	-3.70812	H	-0.81361	-2.37568	-3.46024	H	1.79523	-2.60504	-2.89142
H	-0.34886	-3.93571	-1.80627	H	-0.03167	-4.02454	-1.9522	H	3.19569	-2.44231	-0.96129
H	-1.95402	-3.24192	-1.65786	H	-1.59349	-3.67025	-1.21833	H	3.45734	-0.87928	-1.7271
H	-2.27823	-2.637	0.66051	H	-1.27513	-3.099	1.30868	H	1.53164	0.99458	-1.05935
H	2.82326	0.14448	-1.63474	H	2.63479	0.08245	-1.72235	H	-2.96789	-2.9477	-1.18388
H	2.66271	1.48667	-2.74178	H	2.45583	0.98227	-3.18938	H	-1.68515	-3.68295	-0.24266
H	1.57205	-1.98413	-2.24073	H	1.37872	-1.7867	-2.74081	H	0.71662	-3.24583	-0.78629
H	1.08137	-0.83106	2.99712	H	0.95614	0.44255	3.72705	H	-0.7195	1.07834	-0.78501
H	1.47384	-0.6206	1.30535	H	2.12179	0.068	2.47936	H	-0.51917	2.74301	-0.28895
H	-2.31983	-0.89083	2.2828	H	-0.84835	-1.46161	2.91129	H	1.62257	2.55824	0.89929
H	-0.89805	-1.87537	2.52887	H	0.7294	-1.88117	2.33065	H	1.97153	1.23913	1.96936
H	-0.56844	0.56401	0.8395	H	0.4675	0.1669	0.73439	H	-0.22558	0.21927	1.4018
H	-1.97209	2.01092	4.48128	H	-2.86552	1.74482	2.77336	H	-0.56109	3.27511	4.16709
H	-2.01994	0.18428	4.24022	H	-2.1155	0.23326	3.51676	H	0.86184	3.33672	2.99703
H	3.52327	3.30866	2.32168	H	3.01787	4.13642	1.35693	H	-5.02971	0.19141	1.79049
H	2.39497	4.37174	1.46153	H	1.85343	4.60688	0.10749	H	-4.62244	1.89124	1.98279
H	2.01879	3.84284	3.09588	H	1.41163	4.7627	1.79911	H	-5.10894	1.27644	0.39086
H	0.82687	-0.73675	-4.90473	H	-0.93692	0.19643	-4.01958	H	-0.42899	-2.87981	-3.75842
H	1.79021	0.71948	-4.80115	H	0.1259	1.58646	-4.062	H	-2.08214	-3.24894	-3.33544
H	2.48588	-0.82723	-4.26913	H	0.73049	0.00496	-4.60502	H	-0.77009	-4.23793	-2.65883
H	1.30547	-3.59462	-0.14577	H	1.78944	-4.04396	-0.50685	H	2.61057	-2.46067	1.30658
H	0.87176	-2.78957	1.33747	H	1.77756	-3.26407	1.05045	H	3.67888	-1.04413	1.36837
H	-0.12395	-4.13606	0.76136	H	0.60707	-4.56277	0.71237	H	2.0739	-1.02764	2.1412
H	-0.67861	0.71272	-2.22212	H	-1.24183	0.5089	-1.68076	H	-2.27931	-0.90132	-2.31323
H	-4.03549	0.07109	-2.13028	H	-4.41189	-1.22329	-0.8098	H	5.39108	3.39571	-1.52165
H	-5.37209	0.292	-0.9824	H	-5.06335	-0.60669	0.73183	H	5.53534	2.66075	0.08888
H	-3.89853	1.26592	-0.79456	H	-3.76882	0.28338	-0.08065	H	5.70671	1.65392	-1.39037
7c2				7d1				7d2			
C	-3.08076	1.12921	0.38472	C	0.34679	0.78585	3.1521	C	-2.30112	2.37727	-0.01337
C	-3.11158	0.25606	-0.89229	C	1.29195	-0.35839	2.69605	C	-1.64815	2.11555	-1.39728
C	-3.27974	-1.25023	-0.77024	C	2.44083	-0.01289	1.76155	C	-1.96922	0.8106	-2.10879
C	-2.14971	2.34921	0.17367	C	-1.11228	0.33973	2.91503	C	-1.19309	2.7888	0.98027
C	1.71582	-2.12384	-1.90015	C	1.78568	-1.82486	-2.50806	C	1.75453	-2.02905	-2.27286
C	2.8277	-1.97536	-0.89146	C	0.3491	-1.45478	-2.81517	C	2.27537	-1.98033	-0.85137
C	2.12857	-1.29924	0.29631	C	0.13188	-0.14716	-2.05226	C	1.02211	-1.75372	-0.00568
C	2.11634	0.26756	0.15993	C	-1.37189	0.044	-1.6821	C	1.36349	-1.04134	1.33954
C	-1.97189	-2.00355	-0.5182	C	3.17591	-1.21576	1.16092	C	-1.10557	0.51008	-3.33804
C	-0.85098	-1.86184	-1.58572	C	2.52421	-2.01207	-0.00709	C	0.35074	-0.00562	-3.14868
C	0.4985	-2.4055	-1.02242	C	2.09974	-1.10375	-1.19355	C	0.43444	-1.25331	-2.22685
C	-0.63662	2.05764	0.1594	C	-1.51133	0.23011	1.43558	C	-0.21826	1.6602	1.34953
C	1.40554	1.03499	1.30914	C	-1.71504	1.3149	-0.8608	C	0.16459	-0.69057	2.25677
C	-0.13972	1.0625	1.22982	C	-1.06099	1.40027	0.53614	C	-0.86652	0.29596	1.66557
C	-0.8501	1.35283	2.53992	C	-1.23236	2.71128	1.27719	C	-2.13153	0.5049	2.47411
C	-0.31579	1.95931	3.61407	C	-2.16136	3.6491	1.02679	C	-2.29836	0.21796	3.7761
C	-2.28331	0.92757	2.64005	C	-0.25665	2.98537	2.38499	C	-3.31021	1.0722	1.73683
O	-2.71549	0.31176	1.51313	O	0.68985	2.01789	2.48387	O	-3.05828	1.22523	0.41226
O	-2.97286	1.03353	3.64231	O	-0.24059	4.00192	3.06076	O	-4.40446	1.28431	2.23457
C	-4.52282	1.61037	0.60496	C	0.59601	1.00099	4.65115	C	-3.29788	3.52939	-0.19974
C	-1.22458	-2.59526	-2.87906	C	1.36782	-2.90173	0.46093	C	1.31053	1.0972	-2.69037
C	2.78028	-1.76637	1.60409	C	0.68195	1.05247	-2.843	C	0.26643	-3.07368	0.22873
O	-3.03166	0.74897	-2.02541	O	1.15378	-1.50427	3.13526	O	-0.94128	2.97429	-1.93381
O	-0.64366	-0.48427	-1.90622	O	3.55922	-2.89734	-0.48764	O	0.78285	-0.41064	-4.4661
O	0.78647	-1.8185	0.27125	O	0.93067	-0.32642	-0.8676	O	0.21646	-0.9038	-0.84496

O	3.50224	0.70505	0.13263	O	-2.12796	0.09629	-2.91945	O	2.21943	-1.91249	2.12467
O	2.93286	2.55262	-1.11298	O	-3.95324	-0.83581	-1.87989	O	4.01988	-0.50797	1.85498
C	3.76218	1.85199	-0.55221	C	-3.39993	-0.38562	-2.87217	C	3.51037	-1.52368	2.30339
C	5.23035	2.1459	-0.51281	C	-4.03664	-0.27961	-4.22364	C	4.23543	-2.53649	3.13553
H	-3.75574	-1.61252	-1.68859	H	3.15776	0.55075	2.37229	H	-3.01056	0.90572	-2.44233
H	-3.99506	-1.45519	0.03429	H	2.10561	0.6604	0.97331	H	-1.94059	-0.02499	-1.41037
H	-2.33536	3.07982	0.97357	H	-1.79734	1.04558	3.40392	H	-1.64957	3.16441	1.90607
H	-2.409	2.8748	-0.75459	H	-1.3021	-0.62839	3.39669	H	-0.61194	3.63078	0.58201
H	1.61425	-1.19392	-2.46918	H	1.92549	-2.90916	-2.47067	H	1.56028	-3.06653	-2.57057
H	1.90105	-2.92679	-2.62014	H	2.45032	-1.45386	-3.29758	H	2.48403	-1.63427	-2.98603
H	3.18238	-2.9778	-0.61975	H	-0.30044	-2.24328	-2.41462	H	2.9529	-1.12155	-0.76816
H	3.68604	-1.42157	-1.28204	H	0.15055	-1.37538	-3.88849	H	2.83767	-2.87702	-0.57337
H	1.6389	0.52749	-0.79113	H	-1.68248	-0.83735	-1.10721	H	1.87795	-0.10154	1.10941
H	-2.20603	-3.06941	-0.39634	H	3.4451	-1.91839	1.96161	H	-1.08699	1.38897	-3.99721
H	-1.59867	-1.67953	0.45582	H	4.14369	-0.84294	0.79426	H	-1.64241	-0.25206	-3.92199
H	0.41207	-3.48532	-0.84715	H	2.9194	-0.3996	-1.39001	H	-0.3662	-1.94491	-2.52165
H	-0.35512	1.7006	-0.83496	H	-1.10205	-0.7056	1.0426	H	0.48987	1.54049	0.52394
H	-0.11643	3.01765	0.27988	H	-2.60331	0.12486	1.38825	H	0.37562	1.99666	2.20965
H	1.77332	2.06957	1.33528	H	-2.80628	1.36861	-0.74927	H	0.57095	-0.27583	3.19014
H	1.73126	0.61238	2.26524	H	-1.45539	2.20181	-1.45294	H	-0.33621	-1.6189	2.56071
H	-0.43998	0.06135	0.92995	H	0.01095	1.32381	0.35367	H	-1.20379	-0.1672	0.73823
H	-0.89584	2.12863	4.51872	H	-2.2093	4.56932	1.6055	H	-3.24758	0.3931	4.27858
H	0.70847	2.31435	3.62836	H	-2.9064	3.53456	0.24762	H	-1.50326	-0.19478	4.38652
H	-5.19798	0.76162	0.76541	H	1.63619	1.29684	4.83179	H	-4.07667	3.25417	-0.92077
H	-4.59816	2.24212	1.49688	H	-0.03309	1.8068	5.04514	H	-3.80886	3.76651	0.73992
H	-4.89478	2.18047	-0.25334	H	0.39725	0.09439	5.23273	H	-2.80785	4.43905	-0.56318
H	-0.45495	-2.46034	-3.64699	H	1.06049	-3.59506	-0.32994	H	2.35233	0.76446	-2.75787
H	-2.14921	-2.19574	-3.30918	H	1.67601	-3.53028	1.30419	H	1.23588	1.97496	-3.34236
H	-1.35912	-3.66797	-2.70727	H	0.49079	-2.32579	0.76035	H	1.12576	1.41254	-1.66237
H	2.8124	-2.86165	1.65174	H	1.71933	0.89403	-3.15674	H	0.06514	-3.60248	-0.70904
H	3.80283	-1.39166	1.70956	H	0.08975	1.25202	-3.74146	H	0.82721	-3.75229	0.87895
H	2.19573	-1.46197	2.47765	H	0.69838	1.95535	-2.22354	H	-0.71571	-2.88795	0.67606
H	-1.37076	-0.16287	-2.47847	H	3.78232	-3.50834	0.23741	H	0.80199	0.38604	-5.02615
H	5.43354	3.0552	-1.08567	H	-5.05207	-0.68355	-4.17861	H	3.75414	-2.62668	4.1126
H	5.54849	2.30513	0.52048	H	-4.09105	0.76911	-4.52612	H	4.24525	-3.50018	2.62018
H	5.78588	1.31999	-0.96424	H	-3.46321	-0.86101	-4.95001	H	5.26849	-2.20998	3.28547
	7d3				7d4				7d5		
C	-3.11491	0.21642	0.90974	C	-2.26855	2.49929	-0.17601	C	-0.72762	0.70869	3.11931
C	-2.88371	-1.11181	0.13987	C	-1.52383	2.62815	-1.53433	C	0.14577	-0.56607	2.96763
C	-1.93945	-2.1447	0.73702	C	-1.55618	1.46909	-2.51595	C	1.59162	-0.39734	2.52606
C	-2.72312	1.39439	-0.01096	C	-1.28688	2.84103	0.97072	C	-2.05106	0.48909	2.35512
C	1.98003	-2.06699	-1.81246	C	2.07666	-1.36572	-2.03714	C	2.2991	-2.07051	-1.80033
C	2.9389	-1.1865	-1.03713	C	2.54378	-2.14346	-0.8251	C	1.12257	-1.50518	-2.56808
C	2.04369	-0.4574	-0.01124	C	1.33375	-2.08305	0.1303	C	0.83061	-0.17552	-1.87058
C	1.90567	1.06813	-0.31531	C	1.50237	-0.93105	1.17927	C	-0.66792	0.2273	-2.03899
C	-1.47268	-3.24284	-0.22755	C	-0.28411	0.63799	-2.34961	C	2.32551	-1.69195	2.15957
C	-0.44218	-2.90588	-1.34518	C	-0.34943	-0.81435	-2.86716	C	2.05213	-2.372	0.78487
C	0.89335	-2.34513	-0.78654	C	0.61284	-1.75394	-2.09406	C	2.21617	-1.40636	-0.42274
C	-1.21451	1.58001	-0.23921	C	-0.24628	1.75398	1.30093	C	-1.90028	0.43605	0.82737
C	1.11884	1.90402	0.7319	C	0.25671	-0.63866	2.06033	C	-1.10598	1.53555	-1.32931
C	-0.33043	1.45345	1.016	C	-0.78536	0.30579	1.41054	C	-0.99555	1.52336	0.21128
C	-1.01434	2.11916	2.19353	C	-2.1567	0.31938	2.0554	C	-1.23615	2.84621	0.9106
C	-0.66389	3.28641	2.75926	C	-2.45439	-0.08437	3.30177	C	-1.87437	3.91099	0.39658
C	-2.18254	1.38775	2.78715	C	-3.28403	0.82679	1.20559	C	-0.6919	2.97437	2.30402
O	-2.4111	0.20177	2.16764	O	-2.85587	1.19212	-0.02983	O	0.00506	1.87414	2.68521
O	-2.80161	1.75287	3.7739	O	-4.45825	0.83542	1.53774	O	-0.76636	3.98283	2.98805
C	-4.61617	0.28365	1.23414	C	-3.40967	3.52756	-0.22006	C	-1.00668	0.88486	4.61811
C	-1.04421	-2.02316	-2.44233	C	-0.08106	-0.85957	-4.37089	C	0.69427	-3.07897	0.75502
C	2.57737	-0.71375	1.40707	C	1.12712	-3.45267	0.7898	C	1.77826	0.92944	-2.36879
O	-3.50016	-1.34899	-0.90396	O	-0.87701	3.64306	-1.81659	O	-0.30427	-1.67636	3.26455
O	-0.12338	-4.16677	-1.97664	O	-1.67359	-1.3299	-2.65954	O	3.03727	-3.42179	0.66228

O	0.74213	-1.08362	-0.12213	O	0.18566	-1.82396	-0.70843	O	1.13741	-0.4555	-0.49141
O	3.24075	1.63213	-0.39265	O	2.60247	-1.29076	2.0534	O	-0.92451	0.38314	-3.45882
O	2.52591	3.19109	-1.92237	O	3.08527	0.92969	2.38689	O	-3.11002	-0.26401	-3.16599
C	3.40472	2.68321	-1.24182	C	3.31088	-0.25539	2.58241	C	-2.18555	0.0928	-3.88104
C	4.83132	3.13871	-1.22092	C	4.41348	-0.77906	3.44986	C	-2.28373	0.28118	-5.36359
H	-2.50316	-2.62368	1.54799	H	-1.6152	1.87764	-3.5312	H	2.10875	0.0511	3.38424
H	-1.08454	-1.6606	1.2044	H	-2.47538	0.89941	-2.36423	H	1.66192	0.32173	1.71064
H	-3.11575	2.32965	0.41143	H	-1.8585	3.04387	1.88701	H	-2.75774	1.29255	2.60324
H	-3.21018	1.29362	-0.98975	H	-0.754	3.77633	0.75569	H	-2.53807	-0.43734	2.68653
H	2.45158	-2.97564	-2.19677	H	2.62167	-1.62698	-2.9483	H	3.24001	-1.78729	-2.28745
H	1.59993	-1.48956	-2.66106	H	2.21123	-0.29533	-1.85145	H	2.28123	-3.16391	-1.77535
H	3.48754	-0.51392	-1.70367	H	3.46683	-1.74048	-0.39761	H	0.27516	-2.19238	-2.45124
H	3.68931	-1.81243	-0.53885	H	2.755	-3.17666	-1.12888	H	1.32254	-1.40465	-3.63936
H	1.40831	1.16266	-1.28944	H	1.77036	-0.02524	0.62378	H	-1.2755	-0.60049	-1.65272
H	-2.35279	-3.70946	-0.69174	H	-0.02175	0.62358	-1.2933	H	2.18269	-2.43579	2.95543
H	-1.03571	-4.04455	0.38537	H	0.55357	1.1673	-2.82387	H	3.39982	-1.45975	2.19877
H	1.28534	-3.06354	-0.0526	H	0.50876	-2.7759	-2.48354	H	3.14458	-0.83894	-0.27346
H	-0.88437	0.85412	-0.98655	H	0.54475	1.79615	0.54452	H	-1.51202	-0.5513	0.55897
H	-1.06532	2.56662	-0.69803	H	0.23357	2.0369	2.24739	H	-2.90452	0.49537	0.38729
H	1.11144	2.95074	0.39908	H	0.57938	-0.20231	3.01497	H	-2.14534	1.75006	-1.61172
H	1.6937	1.91938	1.66764	H	-0.21868	-1.58657	2.33394	H	-0.53079	2.37424	-1.74191
H	-0.2525	0.40328	1.29735	H	-0.94579	-0.08873	0.40752	H	0.04734	1.28548	0.42002
H	-1.20712	3.68999	3.61135	H	-3.47299	-0.04532	3.68255	H	-1.99758	4.82906	0.96752
H	0.16552	3.8819	2.3947	H	-1.70099	-0.45942	3.98516	H	-2.30077	3.90653	-0.60017
H	-4.91382	-0.56446	1.86211	H	-4.11026	3.29206	-1.02994	H	-0.07045	1.01858	5.17285
H	-4.86094	1.19187	1.79582	H	-3.98967	3.52113	0.70917	H	-1.61331	1.77782	4.80509
H	-5.2324	0.2665	0.32875	H	-3.03602	4.54463	-0.38097	H	-1.5288	0.01989	5.04106
H	-0.41974	-2.02262	-3.3423	H	-0.16931	-1.88376	-4.751	H	0.59021	-3.75676	1.61005
H	-2.0124	-2.41815	-2.77083	H	-0.82085	-0.26414	-4.91782	H	-0.14265	-2.37887	0.76203
H	-1.17893	-0.99057	-2.12066	H	0.91668	-0.48405	-4.61878	H	0.59693	-3.70888	-0.13635
H	2.68429	-1.78805	1.59791	H	0.99898	-4.23181	0.02865	H	2.82873	0.62595	-2.3044
H	3.55172	-0.24054	1.56404	H	1.97226	-3.73419	1.42531	H	1.57346	1.2016	-3.40884
H	1.88194	-0.3516	2.17075	H	0.21164	-3.479	1.38817	H	1.6949	1.82657	-1.74639
H	-0.9431	-4.511	-2.37479	H	-1.71792	-1.59068	-1.71756	H	3.92044	-3.0303	0.77186
H	4.95514	3.97457	-1.91537	H	3.99167	-1.35213	4.27928	H	-1.57715	-0.38201	-5.86896
H	5.09737	3.47706	-0.21642	H	5.08864	-1.40036	2.85609	H	-3.29516	0.02903	-5.69482
H	5.48538	2.32281	-1.53868	H	4.98253	0.0606	3.85902	H	-2.08265	1.32469	-5.61839
7d6				7d7				7d8			
C	-2.4362	2.42509	-0.06168	C	-2.55485	2.02634	-0.25778	C	-0.55287	0.87395	3.10061
C	-1.83253	2.23009	-1.47554	C	-1.91924	1.80201	-1.65648	C	0.31318	-0.41442	3.07299
C	-1.78476	0.82928	-2.06261	C	-1.94621	0.40115	-2.25111	C	1.69499	-0.3477	2.43877
C	-1.29337	2.87474	0.87149	C	-1.47748	2.61437	0.68232	C	-1.88056	0.59053	2.36252
C	2.11737	-1.29515	-2.06664	C	2.39808	-1.31179	-2.00775	C	1.43671	-2.54658	-1.6872
C	2.49454	-1.99842	-0.77943	C	2.42866	-2.13544	-0.73558	C	1.27179	-1.44708	-2.71582
C	1.23146	-1.84839	0.10209	C	1.14957	-1.72038	0.02373	C	0.87819	-0.20659	-1.88392
C	1.49067	-0.79981	1.23287	C	1.45964	-0.93613	1.3376	C	-0.61466	0.20303	-2.09416
C	-0.50215	0.60186	-2.87047	C	-1.00365	0.16617	-3.4387	C	2.33868	-1.69898	2.10013
C	-0.20564	-0.87715	-3.2144	C	0.53163	0.05795	-3.20357	C	1.81091	-2.53072	0.89272
C	0.64385	-1.63303	-2.15502	C	0.91015	-1.08029	-2.21644	C	1.88882	-1.77115	-0.46057
C	-0.24652	1.78794	1.15525	C	-0.38681	1.62887	1.13237	C	-1.77374	0.47917	0.83351
C	0.30182	-0.47921	2.1715	C	0.23572	-0.5869	2.22846	C	-1.07002	1.50936	-1.38694
C	-0.80553	0.40503	1.55888	C	-0.88604	0.23475	1.55708	C	-0.90235	1.54797	0.14723
C	-2.0546	0.57174	2.40455	C	-2.17256	0.36984	2.34705	C	-1.12775	2.89387	0.80659
C	-2.15727	0.3299	3.72287	C	-2.31196	0.20054	3.67267	C	-1.80988	3.92828	0.28692
C	-3.29663	1.03659	1.70133	C	-3.40531	0.70846	1.56093	C	-0.51054	3.08468	2.16106
O	-3.09852	1.22048	0.37258	O	-3.15168	0.80866	0.23131	O	0.18478	1.99115	2.56511
O	-4.39225	1.14384	2.22937	O	-4.52869	0.78723	2.03217	O	-0.53208	4.12942	2.79222
C	-3.50483	3.52233	-0.1593	C	-3.69361	3.04153	-0.44736	C	-0.83356	1.1873	4.57933
C	0.47148	-0.96271	-4.589	C	1.14244	1.41099	-2.82229	C	0.42131	-3.10922	1.16855
C	0.81853	-3.2156	0.66642	C	0.28572	-2.96423	0.28812	C	1.84049	0.94866	-2.20059
O	-1.38652	3.1897	-2.11362	O	-1.4556	2.75381	-2.29234	O	-0.08841	-1.45158	3.609

O	-1.45572	-1.57736	-3.35268	O	1.07398	-0.29867	-4.49583	O	2.67795	-3.68348	0.78424
O	0.1762	-1.41836	-0.80318	O	0.42936	-0.85434	-0.88605	O	1.07983	-0.5898	-0.50083
O	2.56737	-1.31314	2.06505	O	2.35337	-1.74741	2.14337	O	-0.82912	0.39403	-3.51709
O	3.28346	0.82527	2.50232	O	3.33486	0.14139	3.00911	O	-3.01807	-0.2732	-3.30653
C	3.38856	-0.38554	2.63015	C	3.23925	-1.07411	2.92742	C	-2.07587	0.10966	-3.98392
C	4.44696	-1.06577	3.44235	C	4.0895	-2.04506	3.68729	C	-2.13222	0.33972	-5.46275
H	-2.67142	0.70682	-2.69451	H	-2.97551	0.25186	-2.6021	H	2.33189	0.15286	3.17968
H	-1.84679	0.09008	-1.26779	H	-1.77561	-0.3467	-1.47913	H	1.68464	0.29684	1.5625
H	-1.7078	3.21623	1.82951	H	-1.96251	3.01304	1.58402	H	-2.59951	1.38928	2.59133
H	-0.7777	3.74784	0.45016	H	-0.98907	3.47815	0.2125	H	-2.34159	-0.32999	2.74399
H	2.699	-1.65305	-2.92026	H	2.87703	-1.81543	-2.85201	H	2.14885	-3.31663	-1.99619
H	2.2762	-0.21696	-1.95671	H	2.93165	-0.37508	-1.81858	H	0.46073	-3.01785	-1.53419
H	3.40289	-1.5763	-0.33892	H	3.35183	-1.95938	-0.17458	H	0.53847	-1.72129	-3.48027
H	2.70749	-3.05414	-0.98957	H	2.41614	-3.20289	-0.98732	H	2.22406	-1.29138	-3.23766
H	1.82758	0.122	0.74532	H	1.964	-0.00452	1.04943	H	-1.23501	-0.62897	-1.73638
H	0.36109	1.04876	-2.36302	H	-1.18608	0.93721	-4.20042	H	2.32849	-2.33802	2.99409
H	-0.61505	1.17382	-3.80203	H	-1.325	-0.76865	-3.92096	H	3.40589	-1.50356	1.92129
H	0.53142	-2.71031	-2.34792	H	0.46153	-2.01458	-2.58233	H	2.9322	-1.46074	-0.61429
H	0.37719	1.67462	0.26067	H	0.33971	1.5206	0.32271	H	-1.3892	-0.51262	0.58294
H	0.41802	2.1643	1.94409	H	0.16131	2.09378	1.96275	H	-2.7924	0.51455	0.42456
H	0.69206	0.02151	3.06803	H	0.59911	-0.04575	3.11255	H	-2.12483	1.68524	-1.63764
H	-0.12635	-1.41631	2.54642	H	-0.17831	-1.52115	2.6312	H	-0.53734	2.35551	-1.84132
H	-1.12733	-0.12454	0.66338	H	-1.16343	-0.33334	0.6691	H	0.1495	1.32404	0.32472
H	-3.09674	0.46855	4.25449	H	-3.27881	0.30895	4.16005	H	-1.91669	4.86669	0.82731
H	-1.31809	-0.00688	4.32061	H	-1.47543	-0.04357	4.31764	H	-2.28939	3.87783	-0.68414
H	-4.30074	3.22492	-0.85222	H	-4.45013	2.64869	-1.13699	H	0.10348	1.35902	5.1222
H	-3.98165	3.69801	0.8113	H	-4.20632	3.24464	0.49929	H	-1.43027	2.09995	4.68562
H	-3.08763	4.47175	-0.51161	H	-3.33133	3.99364	-0.84984	H	-1.36789	0.37097	5.07719
H	0.69419	-2.00385	-4.84986	H	2.2348	1.3997	-2.90083	H	0.39199	-3.59426	2.15112
H	-0.19391	-0.58734	-5.37549	H	0.81597	2.19185	-3.51865	H	-0.36175	-2.35241	1.12824
H	1.40146	-0.38661	-4.62418	H	0.88411	1.71303	-1.80721	H	0.16762	-3.90158	0.45585
H	0.67905	-3.94518	-0.14006	H	0.09459	-3.51479	-0.64044	H	2.8831	0.63917	-2.06179
H	1.56356	-3.6162	1.36049	H	0.76639	-3.64665	0.99611	H	1.72302	1.30283	-3.22947
H	-0.14778	-3.15824	1.17748	H	-0.70237	-2.69446	0.67382	H	1.69538	1.79182	-1.51786
H	-1.74543	-1.82862	-2.45579	H	2.02205	-0.08234	-4.50473	H	3.60025	-3.37577	0.77543
H	3.98354	-1.63733	4.25032	H	3.46072	-2.65737	4.33846	H	-1.41024	-0.30815	-5.9662
H	5.04335	-1.7193	2.80075	H	4.64833	-2.67338	2.98918	H	-3.13339	0.0961	-5.82959
H	5.10707	-0.31227	3.8814	H	4.80242	-1.4944	4.30761	H	-1.92602	1.39024	-5.68223

Detailed DP4+ probability for compound **7**. Isomer 1 is 1S*,3S*,4S*,7R*,8R*,12S*, isomer 2 is 1S*,3R*,4S*,7R*,8S*,12R*, isomer 3 is 1S*,3S*,4S*,7R*,8R*,12R*, isomer 4 is 1S*,3R*,4S*,7R*,8S*,12S*.

Functional	Solvent?	Basis Set	Type of Data
B3LYP	PCM	6-311+G(d,p)	Unscaled Shifts

	Isomer 1	Isomer 2	Isomer 3	Isomer 4	Isomer 5	Isomer 6
sDP4+ (H data)	0.00%	0.00%	0.03%	99.97%	-	-
sDP4+ (C data)	0.01%	9.69%	0.00%	90.30%	-	-
sDP4+ (all data)	0.00%	0.00%	0.00%	100.00%	-	-
uDP4+ (H data)	0.01%	0.00%	0.05%	99.94%	-	-
uDP4+ (C data)	0.79%	0.01%	0.01%	99.19%	-	-
uDP4+ (all data)	0.00%	0.00%	0.00%	100.00%	-	-
DP4+ (H data)	0.00%	0.00%	0.00%	100.00%	-	-
DP4+ (C data)	0.00%	0.00%	0.00%	100.00%	-	-
DP4+ (all data)	0.00%	0.00%	0.00%	100.00%	-	-

Table S12.Calculation process of 8

Important thermodynamic parameters (a.u.) of the optimized **8** with simplified structures at B3LYP/6-31+G(d,p) level in the gas phase.

NO.	E+ZPE	G	P%	NO.	E+ZPE	G	P%	NO.	E+ZPE	G	P%

8a1	-1007.030914	-1006.591334	4.79%	8a4	-1007.033258	-1006.593537	49.45%	8b1	-1007.042277	-1006.601048	82.90%
8a2	-1007.033828	-1006.593033	28.99%	8a5	-1007.030997	-1006.590791	2.70%	8b2	-1007.039341	-1006.598679	6.73
8a3	-1007.03218	-1006.591794	7.80%	8a6	-1007.030546	-1006.590688	2.41%	8b3	-1007.039536	-1006.599087	10.38%

Optimized Z-Matrixes of **8** with simplified structures in the Gas Phase (Å) at B3LYP/6-31+G(d,p) level.

8a1				8a2				8a3			
C	-2.28567	0.98715	0.16347	C	-2.28964	0.93004	0.17239	C	-2.29448	0.96408	0.16273
C	-2.00954	-0.48153	0.64699	C	-2.02367	-0.56084	0.589	C	-2.02475	-0.51069	0.63181
C	-2.54122	-1.58311	-0.29176	C	-2.47674	-1.62207	-0.43353	C	-2.53521	-1.60018	-0.33036
C	-1.56349	2.02986	1.06221	C	-1.5839	1.92659	1.13409	C	-1.56527	2.0015	1.06202
C	1.81391	-2.51631	-0.78833	C	1.90785	-2.49192	-0.8427	C	1.82004	-2.55531	-0.79576
C	2.18665	-1.07429	-1.029	C	2.25059	-1.03398	-1.02519	C	2.18554	-1.11084	-1.03448
C	2.61961	-0.14716	-0.14493	C	2.65659	-0.13199	-0.103	C	2.62023	-0.18404	-0.15092
C	2.94664	1.25993	-0.6213	C	2.95877	1.29828	-0.52298	C	2.94482	1.2234	-0.62724
C	-2.00936	-2.99436	0.04815	C	-1.92821	-3.03242	-0.121	C	-2.00891	-3.00923	0.02447
C	-0.49575	-3.09917	0.18765	C	-0.41865	-3.09673	0.07076	C	-0.49535	-3.10354	0.18102
C	0.3161	-2.66523	-0.79879	C	0.41599	-2.67947	-0.90383	C	0.32422	-2.71405	-0.81745
C	-0.03048	1.93606	1.11189	C	-0.05014	1.84437	1.19553	C	-0.03247	1.89715	1.10931
C	2.24137	2.45177	0.05751	C	2.22674	2.44865	0.19763	C	2.24084	2.41311	0.05632
C	0.70064	2.60421	-0.06882	C	0.68542	2.58056	0.05872	C	0.70049	2.56722	-0.06976
C	0.15886	2.28487	-1.44769	C	0.16777	2.31455	-1.34049	C	0.15865	2.25233	-1.44944
C	0.37923	3.11943	-2.48077	C	0.39179	3.19458	-2.33424	C	0.38815	3.08519	-2.48194
C	-0.62941	1.02038	-1.63092	C	-0.60096	1.049	-1.5867	C	-0.64108	0.99516	-1.63533
C	-3.79814	1.28459	0.25389	C	-3.80253	1.22982	0.24527	C	-3.80521	1.2654	0.25967
O	-1.97468	1.19909	-1.22253	O	-1.95593	1.20289	-1.19658	O	-1.98302	1.18855	-1.2203
O	-2.53828	-0.6756	1.96461	O	-2.59179	-0.85627	1.87158	O	-2.58069	-0.76356	1.92856
O	4.35495	1.44281	-0.42749	O	4.36245	1.50183	-0.31507	O	4.35325	1.40774	-0.43638
C	2.86509	-0.43076	1.31458	C	2.89501	-0.46325	1.34783	C	2.87327	-0.46878	1.30704
C	-0.02197	-3.65767	1.50228	C	0.01393	-3.56987	1.43285	C	-0.04079	-3.57753	1.53503
H	-0.93178	-0.61097	0.73539	H	-0.94984	-0.67991	0.72109	H	-0.94933	-0.6488	0.7347
H	-3.63547	-1.62326	-0.23157	H	-3.57132	-1.68728	-0.44386	H	-3.63063	-1.64095	-0.29883
H	-2.28715	-1.35401	-1.33215	H	-2.16604	-1.34012	-1.44494	H	-2.25347	-1.36724	-1.36252
H	-1.83514	3.04407	0.74056	H	-1.86105	2.95484	0.86613	H	-1.82954	3.01727	0.73869
H	-1.93473	1.92265	2.08998	H	-1.96489	1.7591	2.15021	H	-1.93752	1.90197	2.08972
H	2.23037	-3.12086	-1.60322	H	2.36267	-3.05815	-1.66482	H	2.24576	-3.15745	-1.60778
H	2.27485	-2.89746	0.12365	H	2.35251	-2.89779	0.0665	H	2.27707	-2.93717	0.1178
H	2.06984	-0.77182	-2.07081	H	2.13616	-0.69502	-2.05577	H	2.06336	-0.80751	-2.0754
H	2.78752	1.34087	-1.70299	H	2.80388	1.41741	-1.60169	H	2.7829	1.30561	-1.70837
H	-2.48628	-3.3243	0.9799	H	-2.43112	-3.40698	0.77979	H	-2.49552	-3.33458	0.95296
H	-2.34405	-3.70224	-0.72069	H	-2.21437	-3.71901	-0.92777	H	-2.33183	-3.7215	-0.74525
H	-0.14898	-2.32399	-1.72429	H	-0.02462	-2.38063	-1.85521	H	-0.13416	-2.41953	-1.76197
H	0.2943	2.44887	2.0282	H	0.26004	2.3115	2.14082	H	0.29674	2.40357	2.02757
H	0.29481	0.90357	1.23635	H	0.28428	0.80977	1.26834	H	0.28691	0.86202	1.22775
H	2.53321	2.50222	1.11523	H	2.50547	2.45848	1.26	H	2.53261	2.45909	1.11427
H	2.69178	3.36015	-0.37177	H	2.66653	3.38182	-0.18716	H	2.69206	3.32285	-0.36925
H	0.50926	3.6794	0.08191	H	0.47287	3.64412	0.25597	H	0.51031	3.64227	0.08357
H	-0.01572	2.91399	-3.47154	H	0.01451	3.02576	-3.33866	H	-0.00632	2.88305	-3.4737
H	0.94171	4.04041	-2.35827	H	0.94126	4.11601	-2.16489	H	0.95883	4.00108	-2.35902
H	-0.1628	0.1779	-1.1283	H	-0.13483	0.19153	-1.10789	H	-0.17967	0.14617	-1.13775
H	-0.66039	0.75145	-2.69336	H	-0.61035	0.82136	-2.65914	H	-0.67962	0.73111	-2.69872
H	-4.18154	1.18752	1.27449	H	-4.20024	1.12856	1.25984	H	-4.18457	1.1635	1.28133
H	-4.01301	2.30251	-0.09365	H	-4.01069	2.25084	-0.09746	H	-4.01878	2.28569	-0.08154
H	-4.37214	0.61834	-0.3993	H	-4.36861	0.56833	-0.41959	H	-4.38318	0.6038	-0.39474
H	-2.16839	-1.50557	2.30893	H	-3.55035	-0.97155	1.75578	H	-2.05589	-0.26418	2.57664
H	4.79225	0.67517	-0.83397	H	4.81569	0.7482	-0.73026	H	4.79052	0.64174	-0.84633
H	2.45411	-1.38994	1.63817	H	2.39478	0.25151	2.00623	H	2.47984	-1.43566	1.62869
H	3.94031	-0.44115	1.52027	H	2.52446	-1.45165	1.62805	H	3.94882	-0.4621	1.51115
H	2.40679	0.33072	1.95075	H	3.9668	-0.43497	1.56923	H	2.40302	0.28304	1.94606
H	-0.28671	-2.97873	2.31894	H	-0.34051	-2.87817	2.20389	H	-0.35631	-2.86968	2.30842
H	-0.49804	-4.62628	1.68955	H	-0.40984	-4.55943	1.63535	H	-0.48576	-4.55345	1.75726

H	1.05614	-3.82418	1.54271	H	1.09602	-3.66082	1.54152	H	1.04106	-3.69711	1.616
8a4				8a5				8a6			
C	-2.18099	0.76236	0.47816	C	-2.23182	1.03051	0.15593	C	-2.26205	0.955	0.15857
C	-1.98727	-0.70912	0.99036	C	-2.02718	-0.42535	0.70849	C	-2.00266	-0.52366	0.61999
C	-2.58169	-1.78832	0.0603	C	-2.56696	-1.55474	-0.19164	C	-2.4962	-1.6135	-0.35262
C	-1.38175	1.73554	1.38365	C	-1.44177	2.07457	0.99383	C	-1.52618	1.97607	1.07077
C	1.71103	-2.26153	-1.06236	C	1.75227	-2.67874	-0.64491	C	1.8642	-2.55901	-0.80494
C	2.06118	-0.80492	-1.22974	C	2.15262	-1.26437	-0.98199	C	2.22379	-1.11434	-1.04633
C	2.65898	0.03258	-0.35406	C	2.64504	-0.30051	-0.17071	C	2.65084	-0.18316	-0.16291
C	2.94234	1.46587	-0.76321	C	3.00401	1.06106	-0.73994	C	2.96913	1.22366	-0.64747
C	-1.91218	-3.16622	0.2478	C	-2.0774	-2.95518	0.24082	C	-1.96572	-3.0213	-0.00014
C	-0.40415	-3.23457	0.02708	C	-0.56716	-3.07747	0.39276	C	-0.45425	-3.10728	0.16467
C	0.23251	-2.42022	-0.84083	C	0.25178	-2.79943	-0.64287	C	0.36873	-2.72592	-0.83422
C	0.12975	1.69819	1.1145	C	0.08758	1.91851	1.01176	C	0.00737	1.87843	1.10398
C	2.19607	2.60428	-0.03452	C	2.35051	2.31276	-0.11542	C	2.26957	2.41851	0.03659
C	0.63496	2.62205	-0.02502	C	0.81281	2.50506	-0.21511	C	0.72691	2.5661	-0.07269
C	-0.05596	2.50132	-1.3887	C	0.23372	2.13174	-1.56429	C	0.17584	2.2609	-1.45031
C	0.53439	2.85281	-2.54843	C	0.45994	2.89911	-2.64699	C	0.38744	3.1054	-2.47707
C	-1.51523	2.10598	-1.42379	C	-0.60412	0.88904	-1.65812	C	-0.61045	0.99609	-1.63984
C	-3.67736	1.1251	0.50049	C	-3.72395	1.41708	0.24641	C	-3.76994	1.27371	0.25335
O	-1.68547	0.8102	-0.8712	O	-1.93446	1.1536	-1.24289	O	-1.95499	1.17785	-1.22532
O	-2.54564	-0.86797	2.30218	O	-2.56694	-0.56395	2.02949	O	-2.53971	-0.76532	1.92711
O	4.34625	1.69584	-0.58205	O	4.42327	1.19087	-0.58608	O	4.38395	1.4069	-0.51018
C	3.09494	-0.36752	1.03074	C	2.93427	-0.50606	1.29356	C	2.87983	-0.47543	1.29659
C	0.29288	-4.26702	0.8727	C	-0.10945	-3.4358	1.78139	C	-0.00337	-3.55924	1.52778
H	-0.91518	-0.88377	1.09595	H	-0.95685	-0.58748	0.82179	H	-0.92714	-0.65232	0.72773
H	-3.65129	-1.90581	0.27575	H	-3.66313	-1.5635	-0.16321	H	-3.59168	-1.65965	-0.33689
H	-2.50558	-1.49938	-0.9928	H	-2.27796	-1.38782	-1.23451	H	-2.20345	-1.37361	-1.38014
H	-1.73706	2.76685	1.26658	H	-1.67816	3.08615	0.6378	H	-1.79775	2.99758	0.77294
H	-1.56478	1.48657	2.43644	H	-1.79162	2.02387	2.03341	H	-1.88792	1.84823	2.0996
H	1.98676	-2.78461	-1.9858	H	2.15238	-3.34413	-1.41985	H	2.29742	-3.16238	-1.61207
H	2.30276	-2.71287	-0.26326	H	2.20873	-3.01492	0.28653	H	2.31873	-2.93478	0.11268
H	1.77117	-0.40564	-2.20306	H	2.01871	-1.02283	-2.03678	H	2.11916	-0.81629	-2.09052
H	2.78362	1.57271	-1.83989	H	2.82012	1.07262	-1.82093	H	2.78072	1.29608	-1.72464
H	-2.13292	-3.5028	1.26908	H	-2.56622	-3.21494	1.18854	H	-2.45684	-3.35293	0.92372
H	-2.39033	-3.89181	-0.42202	H	-2.42414	-3.70043	-0.48617	H	-2.28043	-3.7316	-0.77508
H	-0.36757	-1.74856	-1.45492	H	-0.20622	-2.57363	-1.60597	H	-0.08376	-2.44229	-1.7847
H	0.62295	2.01604	2.04324	H	0.45588	2.45675	1.89643	H	0.34224	2.37472	2.02569
H	0.45255	0.67241	0.94207	H	0.37476	0.8796	1.17322	H	0.33155	0.84319	1.20687
H	2.56329	2.6647	0.99975	H	2.66977	2.41427	0.93059	H	2.57167	2.47324	1.09105
H	2.542	3.5509	-0.47599	H	2.81509	3.18184	-0.60623	H	2.71371	3.32591	-0.40093
H	0.3677	3.6357	0.31465	H	0.65605	3.59175	-0.11611	H	0.53264	3.63849	0.09313
H	0.00483	2.79497	-3.49589	H	0.03562	2.65117	-3.6159	H	-0.01164	2.90631	-3.46756
H	1.54409	3.24379	-2.60255	H	1.05289	3.80679	-2.58907	H	0.95094	4.02522	-2.35185
H	-1.87444	2.04469	-2.45768	H	-0.16444	0.06003	-1.10967	H	-0.14287	0.1491	-1.14411
H	-2.12884	2.87161	-0.93887	H	-0.66379	0.55746	-2.70142	H	-0.64598	0.73483	-2.70402
H	-4.108	1.01733	1.50109	H	-4.08917	1.43517	1.27796	H	-4.14725	1.20828	1.2786
H	-3.85096	2.15777	0.18146	H	-3.89252	2.4102	-0.1878	H	-3.9745	2.2859	-0.11666
H	-4.24317	0.49538	-0.19456	H	-4.34645	0.72617	-0.33255	H	-4.35695	0.59858	-0.3788
H	-2.2083	-1.70894	2.65508	H	-3.53232	-0.65028	1.95162	H	-3.50121	-0.88136	1.83903
H	4.8028	0.95774	-1.02112	H	4.6987	1.95002	-1.12581	H	4.58995	1.40228	0.43823
H	2.6561	-1.31419	1.35648	H	2.51025	0.29987	1.89831	H	2.39267	-1.39553	1.62787
H	4.18415	-0.4677	1.07143	H	2.51744	-1.43608	1.68623	H	3.95157	-0.56969	1.49822
H	2.79399	0.38322	1.76753	H	4.01516	-0.52688	1.466	H	2.4861	0.32342	1.93026
H	-0.21463	-5.23316	0.77725	H	-0.40377	-2.65505	2.49017	H	-0.32189	-2.83994	2.28918
H	1.33423	-4.4298	0.58488	H	-0.57174	-4.37828	2.09422	H	-0.44813	-4.53203	1.76387
H	0.27248	-3.96806	1.92563	H	0.97035	-3.56978	1.86567	H	1.07819	-3.67743	1.61394
8b1				8b2				8b3			
C	-2.0353	0.4551	-1.23057	C	-2.00769	0.42288	-1.25428	C	-2.01424	0.40603	-1.25035
C	-1.87844	-0.7861	-0.29155	C	-1.85219	-0.80904	-0.30235	C	-1.87681	-0.81421	-0.28144
C	-1.70827	-2.15109	-0.97026	C	-1.68059	-2.18033	-0.96814	C	-1.70682	-2.19529	-0.92698

C	-0.82804	0.65733	-2.18047	C	-0.79832	0.61564	-2.20358	C	-0.79355	0.58094	-2.18855
C	2.21348	-2.60637	1.15999	C	2.2226	-2.60362	1.19822	C	2.18747	-2.60477	1.25362
C	2.66342	-1.28876	0.5887	C	2.67483	-1.28915	0.62306	C	2.6429	-1.30129	0.6569
C	2.5831	-0.05226	1.12731	C	2.5802	-0.04582	1.14467	C	2.57494	-0.05363	1.17159
C	2.99237	1.14585	0.28902	C	3.00402	1.13776	0.29012	C	3.00348	1.12324	0.31708
C	-1.47984	-3.30631	0.02968	C	-1.45929	-3.32752	0.04243	C	-1.49469	-3.32807	0.10187
C	-0.30085	-3.0822	0.96222	C	-0.28806	-3.09763	0.98322	C	-0.32509	-3.0866	1.04144
C	0.94867	-3.0198	0.46067	C	0.96437	-3.02385	0.49056	C	0.92934	-3.03441	0.55113
C	0.52605	0.95874	-1.52488	C	0.55338	0.92592	-1.54697	C	0.55314	0.89406	-1.52302
C	1.96725	2.28542	0.13028	C	1.98427	2.2791	0.09763	C	1.97875	2.25865	0.11475
C	0.52292	1.91512	-0.31319	C	0.54337	1.90037	-0.34989	C	0.53859	1.87906	-0.33458
C	-0.39594	3.12572	-0.54529	C	-0.37486	3.10578	-0.60616	C	-0.37284	3.08728	-0.60756
C	0.00991	4.41001	-0.52821	C	0.03129	4.38988	-0.62376	C	0.03485	4.37115	-0.61549
C	-1.87256	2.84869	-0.76153	C	-1.85216	2.82251	-0.81256	C	-1.84792	2.80913	-0.8359
C	-3.32176	0.3504	-2.07051	C	-3.29252	0.30902	-2.09563	C	-3.29018	0.28866	-2.10467
O	-2.22282	1.55174	-0.29974	O	-2.19675	1.5296	-0.33498	O	-2.20732	1.52497	-0.34763
O	-3.02565	-0.85527	0.57537	O	-3.00091	-0.86943	0.56329	O	-3.03607	-0.85696	0.57114
O	4.175	1.70243	0.86754	O	4.20069	1.68806	0.84356	O	4.17513	1.67442	0.92258
C	2.1006	0.24353	2.5222	C	2.05164	0.25316	2.52175	C	2.09544	0.27118	2.5606
C	-0.66888	-2.86566	2.40529	C	-0.66678	-2.89077	2.4246	C	-0.70671	-2.83973	2.47602
H	-1.02673	-0.59791	0.36937	H	-1.00172	-0.61444	0.35847	H	-1.03269	-0.61548	0.38615
H	-2.60966	-2.39545	-1.54462	H	-2.5794	-2.42839	-1.54493	H	-2.60354	-2.44842	-1.50485
H	-0.87289	-2.12249	-1.6773	H	-0.84181	-2.15901	-1.67138	H	-0.86414	-2.18725	-1.6258
H	-1.0418	1.48101	-2.87434	H	-1.01122	1.43156	-2.90685	H	-0.99516	1.38827	-2.90494
H	-0.71221	-0.21716	-2.83261	H	-0.67973	-0.26588	-2.84563	H	-0.67239	-0.3094	-2.81785
H	2.98756	-3.3581	0.9646	H	2.9996	-3.35516	1.01382	H	2.96308	-3.3609	1.08277
H	2.10825	-2.57055	2.24419	H	2.11009	-2.56045	2.28165	H	2.07133	-2.54496	2.33559
H	3.04796	-1.37989	-0.42831	H	3.09367	-1.39019	-0.37951	H	3.03869	-1.41492	-0.3529
H	3.27975	0.82045	-0.71856	H	3.28651	0.78881	-0.71069	H	3.31301	0.77029	-0.6748
H	-2.39492	-3.46687	0.61331	H	-2.37936	-3.48266	0.61972	H	-2.41681	-3.4706	0.67909
H	-1.32149	-4.23687	-0.53064	H	-1.29709	-4.2627	-0.509	H	-1.33446	-4.27222	-0.43463
H	1.07763	-3.21405	-0.60449	H	1.10265	-3.21377	-0.57428	H	1.06987	-3.2509	-0.50813
H	1.19596	1.3538	-2.30008	H	1.22661	1.3099	-2.32483	H	1.23411	1.26829	-2.29894
H	0.95288	-0.00186	-1.22251	H	0.97906	-0.03004	-1.22855	H	0.97279	-0.06029	-1.19291
H	2.40337	2.98587	-0.59576	H	2.42868	2.9617	-0.64019	H	2.42231	2.93865	-0.62605
H	1.91669	2.84914	1.07285	H	1.92666	2.86445	1.02629	H	1.91904	2.84818	1.04081
H	0.09318	1.37382	0.53792	H	0.1089	1.37169	0.50667	H	0.09813	1.35986	0.52498
H	-0.69357	5.22445	-0.68177	H	-0.67343	5.19962	-0.79653	H	-0.66686	5.18168	-0.79714
H	1.0387	4.707	-0.35834	H	1.06104	4.6916	-0.47024	H	1.06037	4.67306	-0.43816
H	-2.16825	3.00111	-1.80457	H	-2.15273	2.96236	-1.85597	H	-2.12969	2.93757	-1.88599
H	-2.45949	3.55273	-0.15981	H	-2.43755	3.53179	-0.21556	H	-2.44025	3.52901	-0.25869
H	-3.28794	-0.49712	-2.76184	H	-3.25714	-0.54564	-2.77803	H	-3.25198	-0.57486	-2.77568
H	-4.20832	0.24904	-1.43517	H	-4.18025	0.21405	-1.46095	H	-4.18555	0.20678	-1.47895
H	-3.47976	1.2582	-2.66338	H	-3.44978	1.21031	-2.6985	H	-3.43545	1.18316	-2.72055
H	-3.15218	0.06562	0.88221	H	-3.13115	0.05495	0.85731	H	-3.15737	0.0701	0.86059
H	4.76224	0.95554	1.07529	H	3.99896	1.98345	1.7458	H	4.52186	2.34904	0.31622
H	1.17896	0.83144	2.50131	H	1.21089	0.95067	2.47939	H	1.19694	0.89373	2.52805
H	1.89493	-0.65574	3.10639	H	1.69506	-0.63405	3.04887	H	1.8491	-0.61423	3.15012
H	2.85794	0.81249	3.07149	H	2.83622	0.70069	3.13986	H	2.86957	0.81525	3.11156
H	-1.23467	-3.72624	2.77817	H	-1.24288	-3.74967	2.78543	H	-1.27736	-3.69169	2.86106
H	0.19466	-2.7455	3.06197	H	0.19243	-2.78526	3.08949	H	0.15053	-2.7071	3.13853
H	-1.29117	-1.97217	2.51295	H	-1.28236	-1.99281	2.53404	H	-1.32884	-1.94346	2.55891

Detailed DP4+ probability for compound **8**. Isomer 1 is 1R*,3S*,11R*,12R*, isomer 2 is 1R*,3S*,11R*,12S*.

Functional		Solvent?		Basis Set		Type of Data	
B3LYP		PCM		6-311+G(d,p)		Unscaled Shifts	
	Isomer 1	Isomer 2	Isomer 3	Isomer 4	Isomer 5	Isomer 6	
sDP4+ (H data)	0.10%	99.90%	–	–	–	–	
sDP4+ (C data)	0.05%	99.95%	–	–	–	–	
sDP4+ (all data)	0.00%	100.00%	–	–	–	–	
uDP4+ (H data)	0.00%	100.00%	–	–	–	–	
uDP4+ (C data)	0.00%	100.00%	–	–	–	–	
uDP4+ (all data)	0.00%	100.00%	–	–	–	–	
DP4+ (H data)	0.00%	100.00%	–	–	–	–	
DP4+ (C data)	0.00%	100.00%	–	–	–	–	
DP4+ (all data)	0.00%	100.00%	–	–	–	–	

Table S13.Calculation process of 9

Important thermodynamic parameters (a.u.) of the optimized **9** with simplified structures at B3LYP/6-31+G(d,p) level in the gas phase.

NO.	E+ZPE	G	P%	NO.	E+ZPE	G	P%	NO.	E+ZPE	G	P%
9a1	-1272.027154	-1271.555344	1.39%	9b1	-1272.027033	-1271.557599	31.43%	9b7	-1272.024393	-1271.554643	1.37%
9a2	-1272.02787	-1271.557463	13.05%	9b2	-1272.024674	-1271.5554	3.06%	9b8	-1272.025183	-1271.554931	1.86%
9a3	-1272.030314	-1271.559066	71.37%	9b3	-1272.026326	-1271.556102	6.43%	9b9	-1272.024643	-1271.554394	1.05%
9a4	-1272.025388	-1271.555283	1.30%	9b4	-1272.025373	-1271.556009	5.83%	9b10	-1272.027755	-1271.556702	12.15%
9a5	-1272.028334	-1271.557101	8.89%	9b5	-1272.026252	-1271.556917	15.25%	9b11	-1272.026688	-1271.555514	3.45%
9a6	-1272.024903	-1271.555432	1.52%	9b6	-1272.026008	-1271.55569	4.16%	9b12	-1272.030146	-1271.556584	10.72%

Optimized Z-Matrixes of **9** with simplified structures in the Gas Phase (Å) at B3LYP/6-31+G(d,p) level.

9a1				9a2				9a3			
O	-0.98014	0.58484	-4.05542	O	-1.71799	-2.8484	-0.54281	O	-2.08544	-0.68868	-1.32866
O	-0.52835	-2.08552	-3.57879	O	-1.66039	-2.71384	-3.16085	O	1.12522	-0.51978	-3.16347
O	1.79046	4.55204	0.97312	O	0.65234	-0.66072	5.14503	O	1.36568	4.90443	0.00291
O	-0.72212	1.3847	3.59342	O	-0.11739	2.59249	1.63993	O	3.14718	1.08965	2.34841
C	-1.79522	2.11677	1.53847	C	2.23932	1.98302	1.96457	C	0.77473	1.5317	2.72012
C	-0.2844	3.70535	-2.14213	C	2.78524	-2.4254	2.25253	C	-2.16758	3.58259	0.36168
C	-2.614	-1.37654	-2.65218	C	0.68867	-3.15243	-3.39922	C	-0.8499	-1.76963	-3.73378
O	-0.17717	-4.11122	2.90837	O	-0.50214	4.13566	-2.32462	O	-1.72301	-1.95129	1.03992
C	2.18155	-5.26866	3.32832	C	-2.18872	6.04232	-1.54894	C	-2.89567	-3.73446	2.63884
O	2.08481	-4.07593	2.54917	O	-2.464	4.67065	-1.26734	O	-1.48187	-3.75267	2.43912
C	0.82093	-3.59818	2.42352	C	-1.50998	3.81304	-1.71072	C	-1.02868	-2.78995	1.59957
C	1.9122	-1.81163	1.10096	C	-3.0412	2.06408	-0.86334	C	1.15479	-3.92734	1.8368
C	0.78093	-2.34024	1.60528	C	-1.82962	2.39207	-1.35121	C	0.45524	-2.85128	1.42991
C	-0.61279	-1.74602	1.40851	C	-0.69692	1.39801	-1.60753	C	1.09387	-1.63172	0.77104
C	-1.00457	-0.82881	2.59288	C	0.40485	1.52682	-0.51768	C	2.24574	-1.07168	1.63865
C	-0.87536	-1.03169	0.0616	C	-1.16475	-0.07044	-1.81069	C	1.58133	-1.93996	-0.66528
C	1.19531	2.70196	-0.34228	C	0.55664	-1.41452	2.91469	C	0.12675	2.9694	-0.4953
C	0.75426	2.74569	-1.61846	C	1.28853	-2.30099	2.20912	C	-1.21885	2.91285	-0.60459
C	1.35426	1.83737	-2.68138	C	0.58036	-3.22702	1.23318	C	-1.95661	2.21124	-1.74052
C	-0.14787	0.39859	2.73142	C	-0.08832	1.36664	0.89778	C	2.62321	0.3316	1.25569
C	-0.50077	1.8069	2.23147	C	0.80012	1.59434	2.12092	C	1.92844	1.59747	1.76326
C	0.60911	2.83803	2.15003	C	0.38442	1.03933	3.46673	C	2.1767	2.90507	1.03778
C	0.76374	3.57116	0.80868	C	1.03119	-0.31123	3.81575	C	0.9283	3.66822	0.57138
C	-0.62625	-1.9138	-1.17074	C	-0.12306	-0.93102	-2.55161	C	0.4896	-2.31272	-1.69092
C	0.91231	0.36907	-2.59926	C	0.66667	-2.63653	-0.17932	C	-1.2146	1.15972	-2.58366
C	-0.60642	0.17326	-2.7313	C	-0.43594	-3.11826	-1.12657	C	-0.86246	-0.10831	-1.78405
C	-1.08503	-1.29226	-2.51077	C	-0.38242	-2.46064	-2.54357	C	-0.02799	-1.15183	-2.58684
H	-0.74039	-0.17272	-4.62746	H	-2.3441	-2.92287	-1.29423	H	-1.92039	-1.11299	-0.45814
H	-0.84342	-2.99987	-3.4624	H	-1.6357	-2.33655	-4.0587	H	0.84307	-0.04813	-3.96635
H	1.93173	4.95649	0.0994	H	0.92519	-1.58473	5.27958	H	0.57968	5.32588	-0.38572
H	-2.61572	1.47645	1.87506	H	2.61253	2.46114	2.87621	H	0.85456	0.69162	3.41651
H	-2.09735	3.15077	1.7357	H	2.8506	1.09669	1.76779	H	0.72317	2.44278	3.32531

H	-1.69527	1.98729	0.45709	H	2.39433	2.6886	1.14244	H	-0.16793	1.42194	2.17567
H	-0.66907	4.39158	-1.38582	H	3.24259	-1.83625	3.05052	H	-1.67841	4.26634	1.05782
H	0.14432	4.31992	-2.94114	H	3.07022	-3.46842	2.42684	H	-2.90621	4.17637	-0.18829
H	-1.1395	3.15933	-2.55145	H	3.22251	-2.10208	1.30271	H	-2.70036	2.82633	0.94737
H	-2.93588	-1.09363	-3.66073	H	1.69411	-3.00736	-2.99265	H	-1.18662	-1.00708	-4.4435
H	-2.96335	-2.40477	-2.50302	H	0.49383	-4.22842	-3.47279	H	-0.23655	-2.4666	-4.31719
H	-3.12733	-0.73099	-1.93332	H	0.67505	-2.76977	-4.42636	H	-1.72548	-2.31219	-3.36365
H	3.23417	-5.56212	3.36885	H	-1.25573	6.35382	-1.06869	H	-3.15174	-4.5481	3.32302
H	1.83277	-5.08902	4.35014	H	-2.14438	6.21044	-2.62956	H	-3.20624	-2.78853	3.09355
H	1.61396	-6.0793	2.86067	H	-3.0054	6.64291	-1.13893	H	-3.41895	-3.90038	1.69189
H	1.90741	-0.89515	0.5221	H	-3.29376	1.04587	-0.58719	H	2.23157	-3.98398	1.71091
H	2.88852	-2.26146	1.25838	H	-3.82245	2.80112	-0.70188	H	0.68339	-4.78978	2.29919
H	-1.32865	-2.58058	1.41798	H	-0.23393	1.68106	-2.5639	H	0.35009	-0.83312	0.72308
H	-2.0629	-0.56024	2.50044	H	1.19509	0.78963	-0.69009	H	1.96538	-1.10782	2.69853
H	-0.92762	-1.39699	3.52912	H	0.88834	2.50578	-0.62956	H	3.13778	-1.70326	1.53969
H	-0.2639	-0.12751	0.00008	H	-2.08688	-0.08934	-2.40559	H	2.2866	-2.78122	-0.61279
H	-1.9201	-0.69826	0.05391	H	-1.40865	-0.50017	-0.83798	H	2.16878	-1.10033	-1.04892
H	1.98987	1.99787	-0.09622	H	-0.52847	-1.44266	2.79745	H	0.74295	2.50462	-1.26244
H	1.13257	2.2326	-3.68071	H	1.02735	-4.22771	1.25872	H	-2.85809	1.73962	-1.32782
H	2.44873	1.87457	-2.59615	H	-0.46404	-3.35716	1.54046	H	-2.31462	2.99439	-2.42242
H	0.89062	0.1567	2.92154	H	-0.9112	0.67297	1.00744	H	3.16876	0.37701	0.32108
H	0.43053	3.59425	2.92745	H	0.64392	1.76851	4.246	H	2.74424	3.56572	1.70821
H	1.57017	2.38075	2.42275	H	-0.70952	0.94898	3.52422	H	2.84249	2.74184	0.17918
H	-0.16048	4.11236	0.5958	H	2.11856	-0.20466	3.80566	H	0.32372	3.91619	1.44652
H	-1.12866	-2.88124	-1.03601	H	0.88428	-0.73779	-2.16985	H	-0.34614	-2.83896	-1.21953
H	0.4377	-2.16643	-1.24698	H	-0.11446	-0.58462	-3.59471	H	0.96224	-3.04609	-2.36057
H	1.42091	-0.17295	-3.40676	H	0.59664	-1.54838	-0.08519	H	-1.88509	0.89004	-3.40879
H	1.2829	-0.04858	-1.65983	H	1.6523	-2.85637	-0.60582	H	-0.32277	1.60833	-3.03285
H	-1.13668	0.82565	-2.03092	H	-0.38428	-4.20921	-1.22658	H	-0.28701	0.21692	-0.91661
9a4				9a5				9a6			
O	-1.76059	-2.83544	-0.58881	O	-2.42449	-1.04864	-1.09619	O	-1.14092	-3.15137	-0.13432
O	-1.7167	-2.66495	-3.20424	O	0.53495	-0.22881	-3.16236	O	-2.19859	-2.661	-2.47361
O	0.5798	-0.80906	5.14202	O	1.36631	5.00221	0.3532	O	2.05868	-1.14114	4.81134
O	-0.06636	2.52804	1.68175	O	3.26905	1.04693	2.34741	O	-1.51043	1.73664	3.33696
C	2.26861	1.83792	2.00069	C	0.91038	1.46455	2.85361	C	0.71996	2.32536	2.49693
C	2.7667	-2.44383	2.14905	C	-1.98281	3.28098	-0.07628	C	3.51142	-1.40766	1.33626
C	0.62372	-3.13659	-3.46302	C	-1.25182	-1.76942	-3.63026	C	-0.12388	-2.39591	-3.65788
O	-0.41365	4.1545	-2.24575	O	-1.65808	-1.88568	1.35781	O	0.72683	2.63545	-1.85247
C	-2.07448	6.07923	-1.46064	C	-2.55333	-3.79585	2.98977	C	0.28125	4.78183	-3.36178
O	-2.37686	4.70884	-1.20142	O	-1.19599	-3.79223	2.54646	O	-0.80693	4.0578	-2.78724
C	-1.43427	3.84057	-1.64871	C	-0.8859	-2.76451	1.7186	C	-0.42798	2.99009	-2.03967
C	-3.00439	2.10839	-0.83825	C	1.2702	-3.93715	1.42819	C	-2.86145	2.55762	-1.87131
C	-1.78498	2.42011	-1.31725	C	0.54517	-2.81082	1.2907	C	-1.61112	2.28861	-1.44873
C	-0.67317	1.40693	-1.59029	C	1.09891	-1.5243	0.6855	C	-1.3048	1.25911	-0.36506
C	0.4281	1.49005	-0.49527	C	2.31614	-1.03585	1.5046	C	-1.99299	1.65149	0.95999
C	-1.17198	-0.04684	-1.82347	C	1.46258	-1.67811	-0.81053	C	-1.67262	-0.17995	-0.80117
C	0.53525	-1.51289	2.90927	C	0.48426	3.02641	-0.56467	C	1.32676	-1.55738	2.61281
C	1.26708	-2.35487	2.15066	C	-0.80764	2.85491	-0.91798	C	2.13659	-1.95126	1.60791
C	0.55343	-3.26835	1.16696	C	-1.16346	2.27354	-2.27686	C	1.64507	-3.00273	0.62666
C	-0.07308	1.31608	0.91597	C	2.68242	0.39181	1.21716	C	-1.47802	0.90949	2.16521
C	0.81738	1.49242	2.14598	C	2.03183	1.61117	1.86815	C	-0.21119	1.24527	2.95979
C	0.37649	0.93125	3.4805	C	2.25864	2.98267	1.27115	C	0.31337	0.29462	4.01823
C	1.00242	-0.42909	3.83401	C	1.00942	3.66095	0.69442	C	1.57808	-0.48768	3.63593
C	-0.14811	-0.91413	-2.58142	C	0.33438	-2.16141	-1.7459	C	-0.94763	-0.61608	-2.08917
C	0.62896	-2.65658	-0.23692	C	-2.03105	1.009	-2.23403	C	1.0933	-2.32111	-0.63161
C	-0.48513	-3.1145	-1.18238	C	-1.36429	-0.21908	-1.58799	C	-0.18356	-2.96522	-1.18716
C	-0.43125	-2.43967	-2.59153	C	-0.441	-1.06088	-2.52416	C	-0.8588	-2.1447	-2.33354
H	-2.3919	-2.89634	-1.33683	H	-2.20619	-1.28339	-0.16728	H	-1.98917	-3.29528	-0.60527
H	-1.69059	-2.27835	-4.09797	H	0.12647	0.16281	-3.95359	H	-2.61695	-2.195	-3.2196
H	0.92636	-0.14571	5.76111	H	0.6091	5.37923	-0.12719	H	2.71911	-1.79271	4.51899
H	2.65286	2.28959	2.9212	H	1.02274	0.58145	3.48966	H	1.27714	2.74335	3.34184

H	2.85272	0.93623	1.79194	H	0.8641	2.33449	3.51714	H	1.43935	1.92142	1.77771
H	2.44892	2.55118	1.19042	H	-0.04662	1.37946	2.32928	H	0.19356	3.15402	2.01377
H	3.23329	-1.87232	2.9543	H	-1.71186	3.97537	0.72228	H	3.86994	-0.73473	2.11867
H	3.08105	-3.48507	2.2775	H	-2.72546	3.79685	-0.69459	H	4.23318	-2.22859	1.26854
H	3.16975	-2.07629	1.20016	H	-2.45861	2.4077	0.38066	H	3.52291	-0.85887	0.38924
H	1.63383	-3.01245	-3.06114	H	-1.7811	-1.05503	-4.26801	H	0.90866	-2.03487	-3.62669
H	0.41173	-4.20838	-3.54935	H	-0.58531	-2.32403	-4.30178	H	-0.11294	-3.46449	-3.90101
H	0.60965	-2.74032	-4.48498	H	-1.98132	-2.47343	-3.21756	H	-0.63674	-1.8993	-4.4897
H	-1.1416	6.36742	-0.96584	H	-2.69512	-4.66337	3.64015	H	0.9291	5.18705	-2.57812
H	-2.01511	6.26165	-2.53823	H	-2.77033	-2.89116	3.56632	H	0.84994	4.142	-4.04385
H	-2.88504	6.68839	-1.05114	H	-3.23482	-3.88624	2.13803	H	-0.13218	5.61655	-3.93456
H	-3.27883	1.09036	-0.58272	H	2.30772	-3.98469	1.11228	H	-3.72237	2.04918	-1.44839
H	-3.77075	2.85808	-0.66447	H	0.85778	-4.84868	1.85112	H	-3.07173	3.28806	-2.64701
H	-0.20145	1.69869	-2.53978	H	0.34508	-0.73748	0.76967	H	-0.22598	1.25609	-0.16434
H	1.20179	0.73833	-0.67927	H	2.11457	-1.16013	2.57612	H	-1.88226	2.73205	1.12053
H	0.93444	2.45946	-0.58777	H	3.19326	-1.66004	1.29059	H	-3.07314	1.46859	0.89121
H	-2.09371	-0.03449	-2.41919	H	2.28393	-2.40439	-0.88695	H	-2.75578	-0.28205	-0.94147
H	-1.42624	-0.49039	-0.85972	H	1.87584	-0.73664	-1.18686	H	-1.40736	-0.83837	0.02671
H	-0.55135	-1.5729	2.82965	H	1.25914	2.72136	-1.26864	H	0.35717	-2.04937	2.70691
H	1.00413	-4.26768	1.17463	H	-1.71546	3.04457	-2.83121	H	2.46402	-3.68151	0.35937
H	-0.48893	-3.40637	1.47753	H	-0.26005	2.09195	-2.86695	H	0.89089	-3.6348	1.10939
H	-0.91496	0.64294	1.01044	H	3.17726	0.514	0.26151	H	-1.88192	-0.09285	2.24383
H	0.6318	1.65405	4.26707	H	2.67167	3.63194	2.05578	H	0.52754	0.87922	4.92387
H	-0.71912	0.85248	3.52171	H	3.04242	2.94282	0.50207	H	-0.47442	-0.40835	4.32384
H	2.09141	-0.34316	3.85309	H	0.24491	3.72233	1.47266	H	2.35357	0.2116	3.31267
H	0.86444	-0.74143	-2.20351	H	-0.36388	-2.82782	-1.22927	H	0.06389	-0.19247	-2.10746
H	-0.14002	-0.55425	-3.61996	H	0.83779	-2.78758	-2.49728	H	-1.48073	-0.16027	-2.93462
H	0.56665	-1.56987	-0.12433	H	-2.96172	1.23573	-1.69676	H	0.88714	-1.27487	-0.38503
H	1.60907	-2.87598	-0.67619	H	-2.35669	0.77368	-3.25283	H	1.86882	-2.30522	-1.40675
H	-0.44721	-4.20466	-1.29585	H	-0.79405	0.13596	-0.72807	H	0.04734	-3.97543	-1.54654
9b1				9b2				9b3			
O	2.51568	2.73526	2.31342	O	2.76315	2.89066	1.82132	O	2.91718	3.02403	0.97603
O	3.53226	1.87195	-0.03638	O	3.53708	1.70024	-0.47368	O	3.46127	1.63084	-1.27618
O	-4.9587	1.98244	2.00703	O	-4.71231	2.05372	2.37632	O	-4.46609	2.51734	2.28102
O	-3.07843	-1.77193	-0.40778	O	-2.95524	-2.05205	0.50349	O	-3.05204	-1.75261	0.48562
C	-3.52589	0.2071	-1.79143	C	-3.63804	-0.38439	-1.17022	C	-3.8163	-0.14154	-1.20481
C	-1.98975	3.86292	0.02323	C	-1.9721	3.59482	-0.12575	C	-1.95607	3.77858	-0.61855
C	3.88053	0.19298	1.63539	C	4.12926	0.2975	1.36802	C	4.13856	0.34769	0.62809
O	1.96287	-4.30826	-0.10702	O	1.04667	-1.58093	-2.80481	O	2.40891	-4.87825	-1.76893
C	3.1727	-5.74377	-1.99164	C	2.25267	-3.22395	-4.50726	C	2.52446	-5.44508	0.82728
O	2.50877	-4.52337	-2.32131	O	2.00306	-3.62685	-3.16024	O	1.74217	-4.34728	0.35591
C	1.93237	-3.90364	-1.26019	C	1.37205	-2.68935	-2.40725	C	1.77071	-4.18497	-0.98997
C	1.35964	-2.15967	-2.92065	C	1.47591	-4.42707	-0.65186	C	0.75628	-2.85735	-2.72689
C	1.23551	-2.63879	-1.66786	C	1.13244	-3.17423	-1.01075	C	0.91799	-3.02244	-1.39987
C	0.39432	-1.98534	-0.57095	C	0.45914	-2.242	-0.0067	C	0.30042	-2.12775	-0.32857
C	-0.80134	-1.14635	-1.10371	C	-0.79751	-1.51563	-0.55605	C	-0.98014	-1.37388	-0.78925
C	1.28285	-1.1816	0.42068	C	1.46563	-1.29197	0.699	C	1.36435	-1.16404	0.27148
C	-2.64529	1.96331	1.56946	C	-2.44411	1.9618	1.75403	C	-2.28348	2.31226	1.42374
C	-1.75827	2.88446	1.14132	C	-1.61776	2.80779	1.10595	C	-1.50358	3.07533	0.63128
C	-0.38278	2.94742	1.78369	C	-0.18798	2.97485	1.59376	C	-0.02954	3.23118	0.96562
C	-1.91231	-0.99931	-0.09474	C	-1.80088	-1.20342	0.52555	C	-1.86378	-0.96096	0.36112
C	-3.25315	-0.34421	-0.42425	C	-3.19734	-0.65842	0.23611	C	-3.25723	-0.36437	0.16823
C	-4.14708	0.13795	0.69755	C	-3.98509	0.01034	1.3417	C	-3.91146	0.3937	1.30289
C	-4.0002	1.63559	1.01075	C	-3.83545	1.53994	1.3766	C	-3.71877	1.91675	1.22636
C	2.17713	-0.11164	-0.22768	C	2.23023	-0.31637	-0.20739	C	2.10653	-0.29446	-0.75812
C	0.65672	2.29382	0.86296	C	0.76904	2.20394	0.67503	C	0.81535	2.35002	0.03543
C	1.8205	1.6732	1.64602	C	2.03245	1.72944	1.4044	C	2.11064	1.87054	0.70259
C	2.83124	0.88805	0.75602	C	2.96185	0.83357	0.52848	C	2.93389	0.87183	-0.16636
H	3.11167	3.10689	1.63061	H	3.26613	3.16134	1.025	H	3.3665	3.21767	0.12727
H	4.21835	1.40406	-0.54558	H	4.06931	1.14674	-1.07285	H	4.02423	1.03344	-1.80107
H	-4.75928	2.89455	2.28103	H	-4.51036	3.00171	2.46237	H	-4.23155	3.46171	2.28671

H	-3.08603	1.2043	-1.89154	H	-3.25221	0.58365	-1.50491	H	-3.42362	0.78921	-1.62606
H	-3.11448	-0.4236	-2.58564	H	-3.29022	-1.14755	-1.87328	H	-3.56789	-0.95455	-1.89403
H	-4.60344	0.28854	-1.9681	H	-4.73076	-0.35852	-1.23548	H	-4.9083	-0.06834	-1.17109
H	-1.3568	3.61774	-0.83542	H	-1.4345	3.20528	-0.99589	H	-1.50249	3.31732	-1.50143
H	-3.02599	3.8869	-0.32075	H	-3.03992	3.57715	-0.35473	H	-3.03982	3.76408	-0.75267
H	-1.74385	4.87728	0.35515	H	-1.69663	4.64635	0.00841	H	-1.65616	4.83147	-0.58632
H	4.6081	-0.34864	1.02004	H	4.80039	-0.31485	0.75486	H	4.73556	-0.34246	0.02091
H	4.45695	0.92234	2.21549	H	4.74163	1.11567	1.76347	H	4.81258	1.16428	0.91028
H	3.42198	-0.51636	2.33116	H	3.78015	-0.30925	2.20913	H	3.82871	-0.17422	1.53866
H	3.59221	-6.16017	-2.91163	H	2.75125	-4.05049	-5.02103	H	2.42199	-5.49108	1.91499
H	3.99256	-5.55704	-1.29095	H	1.31209	-3.01149	-5.02513	H	2.15927	-6.38656	0.40506
H	2.46355	-6.46682	-1.57648	H	2.91448	-2.35247	-4.53001	H	3.58114	-5.29413	0.58523
H	0.86955	-1.24539	-3.23786	H	1.29477	-4.79594	0.3535	H	0.16958	-2.04738	-3.14546
H	1.96183	-2.65491	-3.67718	H	1.9415	-5.12971	-1.33656	H	1.21654	-3.53051	-3.44748
H	-0.06286	-2.80215	0.00716	H	0.08071	-2.89051	0.7997	H	-0.03743	-2.78022	0.48882
H	-1.21032	-1.63483	-1.99733	H	-1.27585	-2.1496	-1.31328	H	-1.5608	-2.02934	-1.45062
H	-0.47313	-0.14923	-1.41404	H	-0.52843	-0.5786	-1.05211	H	-0.72409	-0.48477	-1.37411
H	0.63568	-0.72724	1.17626	H	0.92054	-0.73843	1.46954	H	0.87901	-0.53257	1.02081
H	1.92647	-1.88093	0.96855	H	2.19909	-1.90517	1.23909	H	2.10813	-1.75324	0.82219
H	-2.35874	1.32561	2.40709	H	-2.07089	1.46712	2.65167	H	-1.83478	1.87439	2.3166
H	-0.10967	3.98778	1.9966	H	0.0808	4.03723	1.62803	H	0.27083	4.28216	0.8792
H	-0.40314	2.43574	2.75428	H	-0.10087	2.60611	2.62361	H	0.14491	2.95211	2.01249
H	-1.57998	-0.97044	0.93413	H	-1.35891	-0.95797	1.48185	H	-1.32834	-0.69768	1.26305
H	-3.96995	-0.4482	1.61033	H	-3.70845	-0.40889	2.31941	H	-3.5483	0.0248	2.27252
H	-5.19278	-0.06876	0.43301	H	-5.04772	-0.24051	1.22308	H	-4.98692	0.17109	1.30614
H	-4.23672	2.21731	0.11684	H	-4.15446	1.95646	0.41828	H	-4.13046	2.28728	0.28475
H	2.97616	-0.608	-0.79512	H	2.96847	-0.87849	-0.79484	H	2.78234	-0.93189	-1.34452
H	1.62259	0.45106	-0.98704	H	1.56553	0.12415	-0.95694	H	1.41007	0.10806	-1.50217
H	0.16388	1.52098	0.26683	H	0.23914	1.34007	0.26473	H	0.2198	1.485	-0.26877
H	1.03247	3.05252	0.16494	H	1.04081	2.85021	-0.16912	H	1.04658	2.92103	-0.87262
H	1.42328	1.01053	2.42321	H	1.74658	1.18721	2.31265	H	1.87297	1.40874	1.66755
9b4				9b5				9b6			
O	2.56639	3.29825	1.30721	O	3.35052	1.71853	-2.19587	O	2.43219	-2.48685	1.91132
O	3.24279	2.09174	-1.02018	O	2.1758	-0.48361	-3.3066	O	2.67224	-2.98048	-0.84378
O	-4.80909	1.8649	2.34566	O	-1.71097	4.85574	2.39757	O	0.50253	3.1504	4.41021
O	-2.87834	-2.10042	0.34783	O	-2.31663	0.06243	2.72551	O	-3.08865	1.75237	1.52047
C	-3.72591	-0.45902	-1.27031	C	-3.67188	1.14101	0.98423	C	-1.42395	3.23802	0.53418
C	-2.36052	3.59635	-0.34099	C	-1.30185	3.86303	-1.48657	C	2.59873	2.70882	0.93191
C	4.06031	0.80923	0.81758	C	3.70136	-1.18126	-1.61587	C	0.52271	-3.46152	0.03186
O	2.375	-4.11177	0.33589	O	1.19118	-4.24689	1.75717	O	-2.04853	0.36477	-3.43926
C	3.55844	-5.72498	-1.41671	C	0.58113	-6.76111	1.13765	C	-2.72608	-1.04491	-5.58926
O	2.71264	-4.66436	-1.86137	O	-0.11864	-5.67334	0.53339	O	-2.60673	-1.67583	-4.31377
C	2.17856	-3.92447	-0.85647	C	0.29916	-4.44946	0.94557	C	-2.26404	-0.8304	-3.30813
C	1.1966	-2.60073	-2.7044	C	-1.35278	-3.61741	-0.6998	C	-2.61687	-2.81029	-1.86855
C	1.29705	-2.82915	-1.38083	C	-0.47577	-3.34689	0.28596	C	-2.15028	-1.55385	-2.00199
C	0.54602	-2.0399	-0.3089	C	-0.1914	-1.94699	0.82932	C	-1.52553	-0.81455	-0.82365
C	-0.80102	-1.43208	-0.79173	C	-1.38103	-0.95578	0.68872	C	-2.4309	0.35038	-0.35996
C	1.46586	-0.97915	0.35964	C	1.12904	-1.37119	0.24372	C	-0.08169	-0.33288	-1.08795
C	-2.58813	1.95462	1.57639	C	-0.54373	3.46629	0.89996	C	1.25059	1.6313	2.78833
C	-1.87208	2.85594	0.87403	C	-0.29698	3.50862	-0.42511	C	2.26722	1.60379	1.90169
C	-0.43823	3.14612	1.27805	C	1.08176	3.13104	-0.93863	C	3.23902	0.43235	1.88335
C	-1.77176	-1.18902	0.33693	C	-1.32887	0.16926	1.69214	C	-2.1631	0.7553	1.06609
C	-3.20911	-0.72071	0.11246	C	-2.44953	1.1963	1.85046	C	-1.70386	2.14346	1.52158
C	-3.98905	-0.11571	1.25939	C	-2.17468	2.50343	2.56184	C	-1.19738	2.33345	2.9365
C	-3.96249	1.42069	1.28867	C	-1.84739	3.67082	1.61713	C	0.27544	2.74381	3.05697
C	2.11972	0.01851	-0.61097	C	1.19145	-1.31883	-1.292	C	0.94386	-1.41797	-1.46073
C	0.53468	2.4586	0.31115	C	1.05136	1.71502	-1.52926	C	3.03957	-0.51693	0.69502
C	1.86869	2.08613	0.97319	C	2.39995	0.99541	-1.39705	C	1.92555	-1.54724	0.9481
C	2.80011	1.23822	0.05252	C	2.35746	-0.48813	-1.87823	C	1.48999	-2.32485	-0.32894
H	2.08898	3.72151	2.042	H	4.23928	1.50034	-1.86257	H	3.06003	-3.04642	1.41148
H	3.46515	2.94137	-0.5863	H	2.66199	0.30014	-3.63434	H	2.41316	-3.46967	-1.64513

H	-4.71242	2.83172	2.39317	H	-1.4054	5.55395	1.79276	H	1.45595	3.32631	4.49229
H	-3.42091	0.53726	-1.60546	H	-4.00643	0.1155	0.79923	H	-0.44482	3.08696	0.07015
H	-3.3561	-1.18714	-1.99895	H	-4.50464	1.67336	1.45547	H	-2.17318	3.28201	-0.2627
H	-4.81962	-0.50747	-1.29037	H	-3.4702	1.60921	0.01571	H	-1.4279	4.21676	1.02528
H	-1.83912	3.24546	-1.23707	H	-1.54957	2.98203	-2.08695	H	2.51618	2.34983	-0.09857
H	-3.43345	3.48388	-0.51124	H	-2.23292	4.26496	-1.08123	H	1.94754	3.57991	1.01944
H	-2.17036	4.66909	-0.2284	H	-0.89057	4.62986	-2.15171	H	3.6239	3.05804	1.09439
H	4.73401	0.23872	0.16789	H	3.67168	-2.22515	-1.94919	H	0.12618	-3.93918	-0.87073
H	4.63407	1.6782	1.15896	H	4.51171	-0.70962	-2.18254	H	1.03703	-4.25639	0.58518
H	3.81684	0.1945	1.68951	H	3.96632	-1.16519	-0.55428	H	-0.31232	-3.12078	0.64653
H	3.93248	-6.25227	-2.29866	H	0.16861	-7.69222	0.73935	H	-3.01338	-1.80739	-6.31853
H	4.41443	-5.32678	-0.86285	H	1.64574	-6.71919	0.88686	H	-3.50548	-0.27675	-5.56648
H	2.99428	-6.43426	-0.80297	H	0.43691	-6.755	2.22264	H	-1.76674	-0.61647	-5.896
H	0.57476	-1.80725	-3.10487	H	-1.91566	-2.83568	-1.1986	H	-2.56077	-3.33823	-0.92148
H	1.74405	-3.18304	-3.44003	H	-1.53137	-4.6262	-1.06094	H	-3.08339	-3.35172	-2.6866
H	0.27032	-2.75736	0.47821	H	-0.03417	-2.05374	1.91297	H	-1.47951	-1.51126	0.01915
H	-1.26903	-2.12121	-1.50622	H	-2.32125	-1.50313	0.83317	H	-3.48086	0.03264	-0.41236
H	-0.63834	-0.49013	-1.325	H	-1.41897	-0.52794	-0.31808	H	-2.33144	1.20345	-1.03747
H	0.8844	-0.44458	1.11634	H	1.28684	-0.37361	0.66329	H	-0.09137	0.39796	-1.90612
H	2.26152	-1.4957	0.91062	H	1.97108	-1.97646	0.60165	H	0.27847	0.23385	-0.22372
H	-2.12637	1.49927	2.45351	H	0.28012	3.21318	1.56858	H	1.12645	0.77298	3.45107
H	-0.26159	4.22812	1.29731	H	1.41987	3.85529	-1.6892	H	4.25735	0.8409	1.83981
H	-0.26211	2.79281	2.30196	H	1.80979	3.1829	-0.11914	H	3.19432	-0.12598	2.8268
H	-1.30502	-0.94288	1.28094	H	-0.32886	0.4717	1.97153	H	-2.01587	-0.09048	1.72685
H	-3.62808	-0.50866	2.22035	H	-1.36172	2.38124	3.29144	H	-1.37749	1.42927	3.53411
H	-5.03274	-0.45179	1.19629	H	-3.05214	2.77077	3.16585	H	-1.81182	3.11019	3.413
H	-4.37684	1.80755	0.35476	H	-2.68368	3.82726	0.93185	H	0.46485	3.6254	2.44287
H	2.8718	-0.50667	-1.21549	H	1.26643	-2.34201	-1.68495	H	0.55668	-2.04292	-2.27554
H	1.39201	0.37649	-1.34787	H	0.24809	-0.94578	-1.70633	H	1.80006	-0.9056	-1.92164
H	0.05667	1.55602	-0.07958	H	0.277	1.13675	-1.01759	H	2.84718	0.06512	-0.20996
H	0.71207	3.1271	-0.54076	H	0.76142	1.78332	-2.58541	H	3.98686	-1.04851	0.53637
H	1.68711	1.54033	1.90653	H	2.73663	1.04311	-0.35465	H	1.05215	-1.07348	1.40668
9b7				9b8				9b9			
O	2.55521	2.79524	2.23811	O	3.15496	2.88441	0.5378	O	2.5108	-2.41578	1.1121
O	3.56535	1.86499	-0.08752	O	3.44569	1.30117	-1.66766	O	2.56985	-2.64845	-1.70756
O	-4.90062	2.03415	2.09349	O	-4.13399	2.91633	2.36084	O	1.00171	3.22068	4.05787
O	-3.07614	-1.73742	-0.34563	O	-3.08708	-1.53856	0.77721	O	-2.83621	2.1571	1.35147
C	-3.51105	0.24276	-1.73051	C	-3.88962	-0.00335	-0.96594	C	-1.08621	3.45101	0.25061
C	-1.95977	3.82045	-0.10181	C	-1.76093	3.82864	-0.77375	C	2.91731	2.84658	0.50557
C	3.90321	0.22734	1.62724	C	4.20096	0.10899	0.25051	C	0.48653	-3.20329	-0.72352
O	1.94729	-4.31066	-0.03867	O	1.60104	-5.36579	-1.58658	O	-3.20641	-3.41738	-2.62309
C	3.16577	-5.76306	-1.90438	C	2.20857	-5.57443	0.99171	C	-3.9445	-3.76496	-0.08082
O	2.50217	-4.54647	-2.24851	O	1.56278	-4.40364	0.49055	O	-3.28986	-2.56489	-0.49335
C	1.92158	-3.91664	-1.19562	C	1.30984	-4.44097	-0.8419	C	-2.97733	-2.53299	-1.81209
C	1.34301	-2.19621	-2.87896	C	0.45019	-3.04763	-2.61214	C	-2.40969	-0.83187	-3.42297
C	1.22766	-2.65482	-1.61768	C	0.63025	-3.18118	-1.28408	C	-2.3049	-1.24226	-2.14559
C	0.3994	-1.97947	-0.5242	C	0.1638	-2.17074	-0.24108	C	-1.54836	-0.4996	-1.05495
C	-0.79679	-1.1426	-1.0594	C	-1.09933	-1.36786	-0.66595	C	-2.42944	0.64546	-0.50838
C	1.30041	-1.16424	0.44662	C	1.32769	-1.24351	0.21169	C	-0.16448	0.02667	-1.4998
C	-2.61881	2.03395	1.57243	C	-2.03121	2.52333	1.38265	C	1.55491	1.7324	2.33048
C	-1.72565	2.91372	1.07438	C	-1.2646	3.18348	0.49107	C	2.53657	1.69868	1.40545
C	-0.34539	3.01036	1.70241	C	0.23519	3.2689	0.71655	C	3.42018	0.46745	1.26628
C	-1.89864	-0.97722	-0.04314	C	-1.87167	-0.82729	0.51137	C	-2.037	1.06357	0.88104
C	-3.23425	-0.30798	-0.3641	C	-3.24211	-0.16535	0.37645	C	-1.42118	2.40889	1.27587
C	-4.11552	0.18103	0.76368	C	-3.76922	0.70343	1.49785	C	-0.83025	2.57367	2.66135
C	-3.98405	1.68447	1.05856	C	-3.49662	2.20363	1.30372	C	0.66973	2.88797	2.70687
C	2.19831	-0.11381	-0.228	C	2.03243	-0.47948	-0.922	C	0.82471	-1.02369	-2.03982
C	0.69104	2.32503	0.80137	C	0.96127	2.28033	-0.20576	C	3.11064	-0.37277	0.02083
C	1.85241	1.72025	1.60019	C	2.26996	1.76068	0.40342	C	1.97802	-1.38589	0.26263
C	2.85826	0.90636	0.73033	C	2.96522	0.66829	-0.46735	C	1.43793	-2.03968	-1.04333
H	3.14575	3.15081	1.54238	H	3.82405	2.65384	1.20678	H	3.09181	-2.94792	0.53237

H	4.2515	1.38061	-0.58078	H	3.7148	2.20475	-1.40477	H	2.23773	-3.09052	-2.50983
H	-5.79672	1.9001	1.7431	H	-3.87057	3.84825	2.26662	H	1.96895	3.32067	4.09217
H	-3.0659	1.23697	-1.83526	H	-3.48008	0.87214	-1.4795	H	-0.12789	3.22169	-0.22433
H	-3.10775	-0.39183	-2.52583	H	-3.7377	-0.87428	-1.61099	H	-1.84347	3.52199	-0.53625
H	-4.58911	0.33029	-1.90111	H	-4.97051	0.13618	-0.86023	H	-1.01609	4.44158	0.71216
H	-1.3304	3.52088	-0.94575	H	-1.39909	3.28046	-1.64928	H	2.76838	2.57479	-0.54393
H	-2.99639	3.82477	-0.44491	H	-2.85035	3.87555	-0.83448	H	2.34145	3.75575	0.68522
H	-1.71065	4.85308	0.16495	H	-1.39691	4.85969	-0.83697	H	3.97219	3.10387	0.64863
H	4.62749	-0.33417	1.02599	H	4.68788	-0.66198	-0.35795	H	-0.00351	-3.57025	-1.63217
H	4.48402	0.96761	2.18884	H	4.95685	0.88591	0.40931	H	1.03644	-4.06036	-0.31644
H	3.44018	-0.46137	2.34057	H	3.94425	-0.32781	1.22046	H	-0.28069	-2.93305	0.00363
H	3.58952	-6.18786	-2.81858	H	2.35848	-5.44385	2.067	H	-3.29497	-4.63072	-0.24386
H	3.98252	-5.56892	-1.20207	H	1.58033	-6.45686	0.8347	H	-4.15328	-3.68577	0.98961
H	2.45549	-6.48274	-1.48533	H	3.18731	-5.70449	0.51933	H	-4.89425	-3.88601	-0.61116
H	0.85527	-1.28411	-3.20596	H	-0.02813	-2.17728	-3.04747	H	-1.96423	0.09441	-3.76864
H	1.93594	-2.70627	-3.63294	H	0.79083	-3.80481	-3.31532	H	-2.95847	-1.40947	-4.16401
H	-0.05692	-2.78439	0.07097	H	-0.15139	-2.74009	0.64503	H	-1.3601	-1.18301	-0.22345
H	-1.21564	-1.6416	-1.94262	H	-1.76199	-2.02557	-1.24277	H	-3.47325	0.30836	-0.45071
H	-0.46661	-0.1516	-1.38661	H	-0.83425	-0.53506	-1.32478	H	-2.42311	1.49068	-1.20513
H	0.66185	-0.69082	1.19784	H	0.94106	-0.54228	0.95651	H	-0.30366	0.78293	-2.28392
H	1.94258	-1.85824	1.00301	H	2.0792	-1.84478	0.73799	H	0.30173	0.57014	-0.67122
H	-2.33279	1.45886	2.45372	H	-1.54999	2.11747	2.27339	H	1.39304	0.84082	2.93879
H	-0.07679	4.06047	1.86804	H	0.58856	4.29182	0.54046	H	4.46295	0.80724	1.21113
H	-0.35405	2.54042	2.69398	H	0.46607	3.04572	1.7659	H	3.36879	-0.15408	2.16891
H	-1.55818	-0.94621	0.98325	H	-1.25637	-0.53479	1.35104	H	-1.93479	0.22094	1.55527
H	-3.91919	-0.39101	1.68153	H	-3.35898	0.37648	2.46376	H	-1.03713	1.68572	3.27453
H	-5.16212	-0.04206	0.51653	H	-4.85254	0.54779	1.58939	H	-1.36708	3.39056	3.16382
H	-4.25386	2.26326	0.17227	H	-3.94999	2.5362	0.36709	H	0.87298	3.78248	2.11622
H	2.99425	-0.62737	-0.78445	H	2.62364	-1.1874	-1.51899	H	0.37565	-1.57009	-2.8787
H	1.6454	0.4329	-1.00015	H	1.30225	-0.07964	-1.63441	H	1.64962	-0.47268	-2.51283
H	0.19416	1.53797	0.22792	H	0.2959	1.43762	-0.41227	H	2.88249	0.28597	-0.82109
H	1.07038	3.06144	0.08173	H	1.1616	2.7786	-1.16272	H	4.0259	-0.91935	-0.24143
H	1.4524	1.08064	2.39506	H	2.07229	1.37068	1.40892	H	1.15279	-0.92395	0.81349
9b10				9b11				9b12			
O	0.55917	1.40568	-2.04728	O	2.65466	2.22254	1.92184	O	0.97484	1.06413	-1.71175
O	1.61434	-0.78702	-3.05572	O	3.03605	2.93711	-0.65755	O	1.91859	-1.30668	-2.35148
O	-1.74923	4.56775	2.28953	O	-4.7265	0.36232	2.39017	O	-1.41726	4.68107	2.13217
O	-3.5907	0.14989	1.80003	O	-2.91543	-1.65145	-1.62857	O	-3.50526	0.35741	1.81072
C	-3.09538	1.30883	-0.29147	C	-3.35686	0.73957	-1.87313	C	-2.692	1.2837	-0.29297
C	-0.05408	4.48919	-1.06784	C	-2.24528	3.29544	1.32019	C	0.40389	4.2749	-1.15253
C	3.65351	-0.71028	-1.78975	C	4.21597	0.91409	-0.17317	C	3.92771	-1.16268	-1.05974
O	1.28187	-3.7628	1.65667	O	2.38065	-2.32536	0.93246	O	0.01118	-3.50691	-1.61442
C	1.05121	-6.41524	1.59108	C	2.64373	-4.92665	1.43353	C	-2.11804	-5.04032	-2.18063
O	0.15764	-5.58828	0.84537	O	1.83999	-4.47292	0.34409	O	-1.84862	-4.62876	-0.8408
C	0.39359	-4.25842	0.97862	C	1.79764	-3.12382	0.21248	C	-0.74203	-3.85601	-0.71726
C	-1.33008	-4.01309	-0.77827	C	0.38398	-3.61357	-1.75835	C	-0.36104	-4.45158	1.595
C	-0.58983	-3.44756	0.19435	C	0.93177	-2.69815	-0.9373	C	-0.57277	-3.46547	0.70581
C	-0.67502	-1.96881	0.56045	C	0.73389	-1.18251	-1.05418	C	-0.60085	-1.995	1.07969
C	-2.13535	-1.54656	0.84686	C	-0.60704	-0.75728	-1.71258	C	-2.0639	-1.50716	1.17065
C	-0.04718	-1.069	-0.52729	C	1.95455	-0.57588	-1.78755	C	0.20395	-1.08812	0.12913
C	-0.28728	3.17691	1.08604	C	-2.50234	0.81175	1.73364	C	0.04854	3.16739	1.09735
C	0.44883	3.59152	0.03207	C	-1.74808	1.92975	1.71011	C	0.841	3.47289	0.04598
C	1.91186	3.20218	-0.09399	C	-0.26737	1.84815	2.04476	C	2.3035	3.05389	0.0114
C	-2.23166	-0.27819	1.65002	C	-1.79827	-1.15089	-0.87978	C	-2.18134	-0.1788	1.86492
C	-2.71647	1.08782	1.14294	C	-3.13229	-0.4105	-0.93921	C	-2.48419	1.16625	1.18841
C	-2.50564	2.31502	2.00957	C	-4.05616	-0.52368	0.25306	C	-2.24951	2.44408	1.97289
C	-1.73283	3.47591	1.36654	C	-3.91716	0.64026	1.25231	C	-1.39981	3.52649	1.28747
C	1.46687	-1.28366	-0.68978	C	2.15707	0.94573	-1.68191	C	1.70172	-1.42158	0.07324
C	2.18919	1.69821	-0.23939	C	0.57115	2.03132	0.77061	C	2.57686	1.54339	0.0793
C	1.90226	1.12803	-1.63712	C	1.98567	1.45474	0.90682	C	2.29058	0.77961	-1.22411
C	2.1493	-0.40602	-1.76806	C	2.82435	1.52459	-0.40452	C	2.4478	-0.76964	-1.11702

H	0.42134	0.82524	-2.82479	H	2.94417	3.03708	1.46234	H	0.83837	0.40314	-2.42299
H	1.79669	-1.73579	-3.17991	H	3.65845	3.01263	-1.40282	H	1.5165	-2.18276	-2.1677
H	-1.1579	5.2473	1.92218	H	-4.50814	1.03472	3.0588	H	-0.80206	5.32115	1.73412
H	-3.55883	0.42787	-0.74489	H	-2.96961	1.66639	-1.4394	H	-3.15921	0.39361	-0.72416
H	-3.81941	2.12604	-0.37685	H	-2.8639	0.58846	-2.83928	H	-3.35053	2.12783	-0.52321
H	-2.21284	1.56619	-0.88442	H	-4.42572	0.87312	-2.06934	H	-1.73745	1.44018	-0.80297
H	-0.0004	3.98129	-2.03512	H	-1.88228	3.56632	0.32402	H	0.48588	3.67639	-2.06481
H	-1.08477	4.81984	-0.92583	H	-3.33562	3.36711	1.3168	H	-0.62363	4.63732	-1.08754
H	0.56342	5.39254	-1.12154	H	-1.88685	4.04453	2.03447	H	1.04404	5.15707	-1.26303
H	3.83412	-1.77024	-2.00333	H	4.81256	0.94411	-1.09218	H	4.04238	-2.25158	-1.11314
H	4.15324	-0.14898	-2.58737	H	4.78156	1.48459	0.57226	H	4.47351	-0.75621	-1.91878
H	4.13536	-0.46782	-0.83795	H	4.15499	-0.12422	0.16554	H	4.41004	-0.81082	-0.14283
H	0.77243	-7.45819	1.41705	H	2.60686	-6.01941	1.44949	H	-3.02646	-5.64893	-2.17244
H	2.08144	-6.27091	1.25077	H	3.68474	-4.61732	1.29703	H	-2.28973	-4.17007	-2.82197
H	0.96307	-6.20749	2.6621	H	2.24872	-4.55304	2.38349	H	-1.29547	-5.6504	-2.56687
H	-2.03897	-3.43245	-1.36039	H	-0.25088	-3.3379	-2.59345	H	-0.21721	-4.23981	2.6498
H	-1.2534	-5.06623	-1.03242	H	0.54508	-4.68055	-1.63237	H	-0.33288	-5.49578	1.29509
H	-0.11449	-1.79928	1.48928	H	0.70088	-0.78907	-0.03403	H	-0.14961	-1.88892	2.077
H	-2.62498	-2.33181	1.43869	H	-0.69169	-1.16382	-2.72714	H	-2.64913	-2.22869	1.75641
H	-2.70031	-1.47015	-0.08801	H	-0.62123	0.33001	-1.82814	H	-2.51806	-1.48703	0.17407
H	-0.5516	-1.22734	-1.48832	H	2.87899	-1.07464	-1.47532	H	-0.24207	-1.12147	-0.87114
H	-0.23046	-0.03015	-0.24092	H	1.85069	-0.81643	-2.85546	H	0.10057	-0.05297	0.46906
H	0.20036	2.57172	1.84922	H	-2.0399	-0.1162	2.07154	H	0.48679	2.62477	1.93341
H	2.38017	3.73766	-0.92942	H	-0.00786	2.60834	2.79092	H	2.79562	3.4631	-0.88006
H	2.42729	3.56267	0.80679	H	-0.03878	0.87699	2.5015	H	2.8016	3.53426	0.86458
H	-1.63288	-0.31575	2.55272	H	-1.54571	-1.67371	0.03441	H	-1.69693	-0.17783	2.83438
H	-2.01822	2.03302	2.95309	H	-3.89126	-1.4754	0.7775	H	-1.81261	2.20977	2.95333
H	-3.4932	2.69309	2.30962	H	-5.0964	-0.57268	-0.09456	H	-3.22969	2.88484	2.20381
H	-2.26597	3.80448	0.47137	H	-4.30535	1.54946	0.78554	H	-1.88071	3.81143	0.34916
H	1.95521	-1.12443	0.27955	H	2.80608	1.22422	-2.52641	H	2.16699	-1.12997	1.02286
H	1.63785	-2.33446	-0.95561	H	1.22866	1.48252	-1.90158	H	1.83427	-2.50727	0.00047
H	3.24707	1.5301	-0.00496	H	0.05461	1.55212	-0.06251	H	3.63334	1.40169	0.33762
H	1.61552	1.16074	0.52183	H	0.62498	3.1034	0.54187	H	2.00197	1.12623	0.91053
H	2.53509	1.64969	-2.36638	H	1.94077	0.42356	1.26818	H	2.96804	1.15123	-2.00347

Detailed DP4+ probability for compound **9**. Isomer 1 is 1R*,3S*,4S*,6R*,11R*,12S*, isomer 2 is 1S*,3S*,4S*,6R*,11S*,12R*.

Functional	Solvent?		Basis Set		Type of Data	
B3LYP	PCM		6-311+G(d,p)		Unscaled Shifts	
	Isomer 1	Isomer 2	Isomer 3	Isomer 4	Isomer 5	Isomer 6
sDP4+ (H data)	92.92%	7.08%	–	–	–	–
sDP4+ (C data)	0.16%	99.84%	–	–	–	–
sDP4+ (all data)	2.00%	98.00%	–	–	–	–
uDP4+ (H data)	100.00%	0.00%	–	–	–	–
uDP4+ (C data)	100.00%	0.00%	–	–	–	–
uDP4+ (all data)	100.00%	0.00%	–	–	–	–
DP4+ (H data)	100.00%	0.00%	–	–	–	–
DP4+ (C data)	99.16%	0.84%	–	–	–	–
DP4+ (all data)	100.00%	0.00%	–	–	–	–

Table S14.Calculation process of 10

Important thermodynamic parameters (a.u.) of the optimized **10** with simplified structures at B3LYP/6-31+G(d,p) level in the gas phase.

NO.	E+ZPE	G	P%	NO.	E+ZPE	G	P%	NO.	E+ZPE	G	P%
10a1	-1272.037096	-1271.565556	36.97%	10a6	-1272.035929	-1271.562629	1.67%	10b3	-1272.030034	-1271.557272	3.02%
10a2	-1272.036347	-1271.564492	11.97%	10a7	-1272.035137	-1271.562895	2.20%	10b4	-1272.030844	-1271.556271	10.47%
10a3	-1272.037843	-1271.564169	8.50%	10a8	-1272.035958	-1271.563354	3.58%	10b5	-1272.032097	-1271.559938	50.98%
10a4	-1272.037241	-1271.565211	25.65%	10b1	-1272.032874	-1271.558842	15.96%	10b6	-1272.030783	-1271.559115	21.21%
10a5	-1272.036062	-1271.564194	8.73%	10b2	-1272.031041	-1271.557801	5.30%	10b7	-1272.029379	-1271.556956	2.16%

Optimized Z-Matrixes of **10** with simplified structures in the Gas Phase (Å) at B3LYP/6-31+G(d,p) level.

10a1				10a2				10a3			
C	0.29223	1.97506	1.84117	C	-0.03717	0.62712	2.7357	C	0.41911	1.34094	2.34507
C	0.68723	2.57761	0.45449	C	1.38961	0.95322	2.1893	C	0.98145	-0.11779	2.3972
C	-0.43157	2.55466	-0.59686	C	1.40879	1.87041	0.95841	C	2.07696	-0.40166	1.35393
C	-0.45545	0.62017	1.7858	C	-1.07443	0.204	1.66811	C	0.07266	1.87103	0.93164
C	1.11333	0.1121	-3.02935	C	2.74553	-0.52174	-1.64101	C	0.37255	-2.91405	-0.68105
C	0.71021	-1.26906	-3.46955	C	2.11838	-1.09064	-2.88446	C	-0.09339	-2.92816	-2.11287
C	0.03142	-2.10042	-2.40408	C	0.63735	-1.37772	-2.77183	C	-0.50506	-1.57556	-2.65024
C	0.81718	-2.47745	-1.14996	C	0.15594	-2.38514	-1.73066	C	-1.73076	-0.88645	-2.054
C	-0.13084	3.31645	-1.90429	C	2.80317	2.3717	0.5287	C	2.86156	-1.7168	1.54031
C	0.82249	2.65161	-2.92791	C	3.71516	1.38878	-0.24555	C	2.15991	-3.03358	1.13961
C	0.41408	1.23316	-3.28038	C	3.02584	0.77953	-1.45143	C	1.65559	-3.01197	-0.28904
C	0.3154	-0.53009	1.11944	C	-0.73041	-1.06803	0.87328	C	-0.97791	1.06003	0.15371
C	0.2068	-2.91294	0.15091	C	-1.2487	-2.48695	-1.21177	C	-2.03334	0.5813	-2.15712
C	-0.55694	-1.78654	0.88555	C	-1.67922	-1.29215	-0.32814	C	-1.07267	1.47306	-1.33546
C	-1.23947	-2.32595	2.13754	C	-3.13688	-1.43994	0.0953	C	-1.35375	2.9504	-1.56647
C	-2.58237	-2.3666	2.23476	C	-3.58834	-2.46675	0.84069	C	-0.51155	3.71853	-2.28433
C	-0.37351	-2.80609	3.25935	C	-4.06046	-0.3472	-0.34279	C	-2.55034	3.64129	-0.99878
O	-1.0945	-3.4918	4.18308	O	-5.35875	-0.73267	-0.25334	O	-3.42294	2.74602	-0.48041
O	0.83033	-2.607	3.31611	O	-3.67206	0.74139	-0.74058	O	-2.71206	4.8535	-1.01422
C	-0.31788	-4.00054	5.26805	C	-6.29097	0.26027	-0.6817	C	-4.58123	3.33419	0.11176
C	-0.55112	2.98119	2.63624	C	-0.58698	1.82435	3.5251	C	1.39773	2.31594	3.01297
O	1.52881	1.78943	2.56028	O	0.12757	-0.46563	3.66426	O	-0.78506	1.36909	3.13196
C	-1.46562	-2.13487	-2.44522	C	-0.28321	-0.36025	-3.37345	C	0.54006	-0.81661	-3.40893
O	0.62017	-3.4016	-2.22855	O	0.28991	-2.73146	-3.11561	O	-1.78545	-1.58566	-3.30535
C	0.83359	3.48714	-4.21677	C	4.96588	2.13825	-0.72739	C	3.13594	-4.20591	1.31816
O	1.83544	1.87191	-0.0356	O	2.05806	-0.28088	1.89467	O	-0.13559	-1.00373	2.24165
O	2.16082	2.68897	-2.44725	O	4.19789	0.37442	0.62753	O	1.07241	-3.26122	2.03871
H	1.02466	3.61097	0.60217	H	1.97672	1.41417	2.99332	H	1.37063	-0.30632	3.40538
H	-1.32473	3.01649	-0.15921	H	0.80848	2.76031	1.18208	H	2.8161	0.40684	1.40387
H	-0.704	1.5242	-0.84549	H	0.93008	1.37786	0.10626	H	1.65852	-0.37754	0.34308
H	-1.42106	0.75562	1.28338	H	-1.24279	1.03686	0.97462	H	0.9914	1.94495	0.33678
H	-0.68285	0.31348	2.8153	H	-2.03197	0.02713	2.17555	H	-0.31327	2.89376	1.03643
H	2.07021	0.17913	-2.51513	H	3.02038	-1.24708	-0.87788	H	-0.41742	-2.82099	0.06201
H	0.05946	-1.2024	-4.35097	H	2.2969	-0.41695	-3.73242	H	0.68492	-3.35577	-2.75768
H	1.61529	-1.78085	-3.82276	H	2.65914	-2.01284	-3.13531	H	-0.93698	-3.62812	-2.17727
H	1.81148	-2.06581	-1.01894	H	0.8803	-2.80077	-1.03983	H	-2.22111	-1.34547	-1.20322
H	0.25935	4.30759	-1.63772	H	3.32979	2.72594	1.42464	H	3.17081	-1.7771	2.59227
H	-1.09825	3.48461	-2.39793	H	2.63747	3.25433	-0.10486	H	3.7838	-1.62514	0.94977
H	-0.53632	1.1427	-3.80397	H	2.74951	1.48691	-2.23195	H	2.42676	-3.0723	-1.05668
H	1.20885	-0.77122	1.70469	H	-0.73735	-1.93905	1.53936	H	-1.9499	1.13232	0.652
H	0.67087	-0.19833	0.14187	H	0.28379	-0.97941	0.47996	H	-0.70048	0.00486	0.17756
H	-0.472	-3.75903	-0.01934	H	-1.95109	-2.58668	-2.04996	H	-2.00618	0.89556	-3.20881
H	1.01542	-3.29932	0.78301	H	-1.32182	-3.42319	-0.64421	H	-3.06928	0.72343	-1.83051
H	-1.35119	-1.45493	0.20315	H	-1.58377	-0.39789	-0.95434	H	-0.06828	1.27765	-1.73651
H	-3.09442	-2.74267	3.11569	H	-4.62731	-2.55328	1.14539	H	-0.69802	4.77775	-2.44694
H	-3.21831	-2.02356	1.42439	H	-2.92506	-3.25873	1.17409	H	0.3895	3.31277	-2.73207
H	-0.99024	-4.53656	5.94359	H	-6.13056	0.50813	-1.73568	H	-4.29763	3.99431	0.93751
H	0.4381	-4.70283	4.90293	H	-7.29819	-0.15129	-0.57266	H	-5.16131	3.87853	-0.63994
H	0.14892	-3.18059	5.82282	H	-6.21295	1.15487	-0.05592	H	-5.20275	2.52824	0.51162
H	-0.73214	2.61872	3.65487	H	-1.53494	1.5685	4.01266	H	0.95707	3.31676	3.09169
H	-0.02255	3.9353	2.743	H	0.10066	2.11	4.32924	H	1.62186	2.0018	4.03882
H	-1.51925	3.17033	2.16316	H	-0.75699	2.69558	2.88575	H	2.33902	2.39877	2.4618
H	1.32308	1.338	3.39934	H	-0.74127	-0.64115	4.06862	H	-1.19245	0.4896	2.98077
H	-1.87419	-1.14933	-2.20053	H	0.00405	-0.14853	-4.40883	H	0.97354	-1.44345	-4.19538
H	-1.8152	-2.4089	-3.44626	H	-1.32499	-0.69446	-3.38979	H	0.13913	0.07847	-3.89405
H	-1.88774	-2.86292	-1.74586	H	-0.23343	0.57588	-2.80836	H	1.34699	-0.50823	-2.73669
H	1.53797	3.07342	-4.94894	H	5.6592	1.45989	-1.23973	H	2.65663	-5.16122	1.07206

H	1.16911	4.51109	-4.01335	H	5.51867	2.55778	0.12161	H	3.46093	-4.28479	2.36223
H	-0.15649	3.53662	-4.68336	H	4.71683	2.95597	-1.41276	H	4.02493	-4.09737	0.68721
H	2.37299	1.71008	0.77268	H	1.77982	-0.87644	2.62613	H	0.22523	-1.91408	2.32575
H	2.15495	2.26235	-1.56329	H	3.41119	-0.02871	1.05387	H	0.70246	-4.13465	1.80872
10a4				10a5				10a6			
C	-0.07236	1.83348	2.31673	C	-0.42584	0.78015	2.4971	C	-0.14647	1.73532	2.44852
C	1.40237	1.33186	2.22675	C	0.96776	0.07736	2.44044	C	1.33661	1.28427	2.25673
C	2.17373	1.80941	0.99213	C	1.9876	0.75887	1.51684	C	2.03362	1.7338	0.96461
C	-0.99783	1.43424	1.1341	C	-1.04948	1.12853	1.12326	C	-1.21894	0.82524	1.79468
C	2.16852	-0.87042	-1.5529	C	2.34587	-1.9608	-1.06587	C	1.98992	-0.81951	-1.83462
C	1.46134	-0.7859	-2.87759	C	1.98783	-2.00735	-2.52643	C	1.32329	-0.64703	-3.1718
C	0.15509	-0.03204	-2.78593	C	0.74936	-1.2231	-2.89912	C	-0.00333	0.06753	-3.06576
C	-1.00871	-0.7401	-2.10012	C	-0.59777	-1.65062	-2.32099	C	-1.17746	-0.72543	-2.50231
C	3.63115	1.30322	0.89462	C	3.42954	0.21692	1.60433	C	3.47068	1.19231	0.78825
C	3.85539	-0.05302	0.17481	C	3.73697	-1.13544	0.91599	C	3.62398	-0.13516	-0.00099
C	3.18553	-0.07344	-1.18373	C	3.29273	-1.16788	-0.53402	C	2.98319	-0.04176	-1.37124
C	-1.21565	-0.07759	0.94407	C	-1.28993	-0.05735	0.17243	C	-1.1425	0.63804	0.27545
C	-2.16741	-0.02449	-1.46538	C	-1.82393	-0.78695	-2.24661	C	-2.37574	-0.10767	-1.84607
C	-2.41271	-0.41882	0.01445	C	-1.70215	0.39053	-1.25111	C	-2.34111	-0.16747	-0.29575
C	-2.89394	-1.86894	0.12662	C	-2.94954	1.26641	-1.28437	C	-2.47805	-1.60809	0.20109
C	-2.14586	-2.89479	0.57642	C	-2.91223	2.5104	-1.7997	C	-1.4513	-2.37948	0.6076
C	-4.32589	-2.08895	-0.2652	C	-4.22012	0.69905	-0.73198	C	-3.88458	-2.1316	0.2313
O	-4.61648	-3.40957	-0.37994	O	-5.17387	1.65756	-0.60253	O	-3.92576	-3.4447	0.57113
O	-5.11016	-1.17335	-0.46993	O	-4.36034	-0.47471	-0.42445	O	-4.85928	-1.44194	-0.03247
C	-5.96098	-3.67686	-0.77858	C	-6.4066	1.17978	-0.0635	C	-5.24133	-3.99863	0.59818
C	-0.11466	3.35718	2.50077	C	-0.35216	2.06107	3.34251	C	-0.35952	3.21049	2.09932
O	-0.60856	1.24301	3.52032	O	-1.32307	-0.13366	3.15893	O	-0.38165	1.60295	3.87452
C	0.22752	1.4593	-2.91521	C	0.97533	0.15084	-3.4508	C	0.04511	1.56572	-3.0555
O	-0.93055	-0.59946	-3.53028	O	-0.20335	-1.98751	-3.65862	O	-1.04623	-0.44972	-3.90653
C	5.35904	-0.2928	-0.00819	C	5.25024	-1.39201	0.96725	C	5.11435	-0.45276	-0.17575
O	1.41651	-0.09696	2.29705	O	0.78376	-1.28801	2.04468	O	1.41688	-0.13833	2.39608
O	3.3875	-1.13393	0.9732	O	3.1509	-2.20225	1.65233	O	3.07348	-1.23401	0.71759
H	1.9386	1.66922	3.12339	H	1.37508	0.02931	3.45778	H	1.92482	1.6903	3.09141
H	2.21539	2.90487	1.01898	H	2.0348	1.82244	1.77915	H	2.10472	2.82866	0.98874
H	1.62753	1.54654	0.08479	H	1.65313	0.71002	0.47572	H	1.43411	1.49508	0.08734
H	-0.62836	1.87691	0.20343	H	-0.42505	1.87716	0.62009	H	-2.20982	1.22865	2.04776
H	-1.98329	1.8834	1.31857	H	-2.02118	1.60631	1.30598	H	-1.19445	-0.15239	2.29131
H	1.78922	-1.61481	-0.85554	H	1.8126	-2.66467	-0.42999	H	1.60644	-1.62528	-1.21177
H	2.10723	-0.31507	-3.62903	H	2.83629	-1.65833	-3.12898	H	1.98057	-0.10296	-3.86141
H	1.28931	-1.80882	-3.23641	H	1.85887	-3.06266	-2.80135	H	1.18897	-1.64193	-3.61539
H	-0.86604	-1.74727	-1.73128	H	-0.61644	-2.44994	-1.58889	H	-1.02792	-1.7586	-2.2143
H	4.05826	1.25927	1.90478	H	3.70509	0.15378	2.66534	H	3.93326	1.08691	1.77816
H	4.19617	2.07242	0.34957	H	4.07952	0.98283	1.15851	H	4.04272	1.96824	0.26027
H	3.54795	0.66967	-1.89234	H	3.81798	-0.47833	-1.19321	H	3.35892	0.75745	-2.00814
H	-1.40509	-0.52624	1.92678	H	-2.03279	-0.73794	0.60211	H	-0.20772	0.13887	0.0118
H	-0.29973	-0.53106	0.55181	H	-0.36367	-0.62679	0.07637	H	-1.13063	1.62453	-0.20114
H	-2.03583	1.06043	-1.52012	H	-2.06368	-0.39185	-3.24269	H	-2.50265	0.93551	-2.15456
H	-3.06726	-0.24258	-2.05423	H	-2.66272	-1.43713	-1.97375	H	-3.2679	-0.63136	-2.21245
H	-3.24268	0.20709	0.37291	H	-0.86496	1.00666	-1.60626	H	-3.232	0.37922	0.04595
H	-2.53359	-3.90619	0.66023	H	-3.78751	3.15187	-1.84263	H	-1.58903	-3.39651	0.96371
H	-1.10992	-2.76667	0.87325	H	-1.99564	2.92594	-2.20662	H	-0.42407	-2.02855	0.59682
H	-6.16155	-3.24696	-1.76499	H	-6.25617	0.78345	0.94564	H	-5.70335	-3.93898	-0.39228
H	-6.08617	-4.76132	-0.84218	H	-7.09948	2.02364	-0.00356	H	-5.1594	-5.05241	0.87853
H	-6.66557	-3.29016	-0.03559	H	-6.84334	0.41959	-0.7188	H	-5.85708	-3.48852	1.34561
H	-1.13115	3.69442	2.73499	H	-1.35412	2.47355	3.50893	H	-1.36623	3.5383	2.38328
H	0.51098	3.66539	3.34607	H	0.06037	1.85243	4.33619	H	0.33715	3.84465	2.65947
H	0.22027	3.8887	1.60511	H	0.26229	2.83225	2.86861	H	-0.22828	3.40856	1.03184
H	-1.51718	1.57511	3.63606	H	-1.10147	-0.14517	4.10759	H	-1.28231	1.92362	4.06118
H	0.90971	1.74372	-3.72332	H	1.69595	0.11956	-4.27486	H	0.70308	1.93189	-3.85089
H	-0.74513	1.90405	-3.14891	H	0.05801	0.60143	-3.84185	H	-0.93585	2.02038	-3.2212
H	0.59384	1.90898	-1.98904	H	1.37565	0.80978	-2.67386	H	0.43421	1.93242	-2.10191

H	5.54882	-1.26387	-0.48167	H	5.49974	-2.36725	0.53132	H	5.2562	-1.4032	-0.70467
H	5.86812	-0.31938	0.96254	H	5.60486	-1.42005	2.0045	H	5.60254	-0.56835	0.79918
H	5.82808	0.48498	-0.62086	H	5.81638	-0.62196	0.43161	H	5.63936	0.33144	-0.73225
H	0.81641	-0.30183	3.04709	H	-0.1187	-1.52672	2.35159	H	1.00181	-0.32444	3.26445
H	2.57072	-0.83323	1.42614	H	2.19279	-2.00302	1.72427	H	2.39199	-0.8836	1.32927
10a7				10a8				10b1			
C	-0.53078	0.87971	2.48491	C	0.85706	1.79339	2.06545	C	-2.6701	0.60437	-0.73128
C	0.87585	0.20251	2.43652	C	1.56445	0.44332	2.39982	C	-2.02274	-0.79728	-0.97059
C	1.89382	0.91103	1.53136	C	2.59284	-0.02176	1.36369	C	-0.82032	-0.78076	-1.9245
C	-1.14303	1.23495	1.10802	C	0.06298	1.83077	0.72997	C	-1.68616	1.71285	-0.28718
C	2.3191	-1.77615	-1.0877	C	1.02505	-2.55527	-0.82166	C	1.49569	-1.9087	0.28938
C	1.97696	-1.81231	-2.5525	C	0.73344	-2.35743	-2.28382	C	2.18423	-1.69104	1.61648
C	0.72538	-1.05125	-2.92993	C	0.32378	-0.93833	-2.60232	C	1.51142	-0.6543	2.48697
C	-0.61663	-1.50384	-2.35885	C	-1.07397	-0.50601	-2.17142	C	1.88389	0.80966	2.25945
C	3.34246	0.38819	1.62322	C	3.28973	-1.36294	1.6885	C	-0.20795	-2.1414	-2.31911
C	3.67523	-0.94956	0.91798	C	2.59353	-2.65526	1.18572	C	-0.06713	-3.25362	-1.25103
C	3.24293	-0.96956	-0.53606	C	2.25865	-2.56337	-0.28876	C	0.67186	-2.93842	0.03068
C	-1.34474	0.05627	0.14023	C	-1.12328	0.85413	0.6362	C	-0.80082	1.33156	0.90374
C	-1.857	-0.6599	-2.28955	C	-1.47113	0.92234	-1.9284	C	1.00289	1.96937	2.61769
C	-1.76149	0.50855	-1.28074	C	-2.09922	1.17147	-0.53211	C	0.07795	2.46937	1.47271
C	-3.00766	1.37978	-1.32484	C	-3.45857	0.48202	-0.40402	C	0.80846	3.34208	0.45563
C	-2.99559	2.59507	-1.90512	C	-3.69722	-0.62463	0.326	C	0.64535	4.68025	0.46465
C	-4.29648	0.96552	-0.69577	C	-4.64918	1.06049	-1.10867	C	1.68069	2.69785	-0.57462
O	-4.31205	-0.3686	-0.46872	O	-4.34141	2.26416	-1.6543	O	2.37244	3.62017	-1.2921
O	-5.20819	1.73695	-0.43295	O	-5.74381	0.51992	-1.17139	O	1.76567	1.48949	-0.73192
C	-5.51003	-0.84252	0.14639	C	-5.42381	2.87628	-2.35652	C	3.21941	3.06393	-2.29799
C	-0.49099	2.15027	3.34796	C	1.86484	2.95161	2.08776	C	-3.83254	0.48563	0.26899
O	-1.41976	-0.06225	3.12077	O	-0.07578	2.0184	3.14409	O	-3.25836	1.04172	-1.9714
C	0.92849	0.32708	-3.48009	C	1.4295	0.04183	-2.85277	C	0.24781	-1.08682	3.17193
O	-0.21004	-1.83308	-3.69407	O	-0.75167	-0.80939	-3.54101	O	2.39136	0.05863	3.37349
C	5.19178	-1.18423	0.97771	C	3.51974	-3.85656	1.4117	C	0.59674	-4.47818	-1.90368
O	0.71776	-1.16156	2.02406	O	0.57314	-0.56797	2.60104	O	-3.03261	-1.64924	-1.53828
O	3.09942	-2.03552	1.63445	O	1.4205	-2.92583	1.94398	O	-1.39634	-3.64969	-0.87604
H	1.27294	0.15001	3.45767	H	2.0702	0.54765	3.36866	H	-1.73476	-1.21829	-0.00126
H	1.92323	1.97137	1.80908	H	3.37879	0.74006	1.29765	H	-0.02783	-0.15845	-1.50783
H	1.5695	0.87235	0.48667	H	2.13215	-0.07208	0.37582	H	-1.1151	-0.28927	-2.86132
H	-0.5281	2.00295	0.62269	H	0.74238	1.67355	-0.11397	H	-2.25276	2.62288	-0.04854
H	-2.12652	1.68983	1.28487	H	-0.34244	2.84521	0.6141	H	-1.06184	1.98986	-1.14539
H	1.79761	-2.50138	-0.46632	H	0.15859	-2.6664	-0.17256	H	1.71999	-1.16925	-0.47569
H	2.82404	-1.43751	-3.14137	H	1.60426	-2.63505	-2.89058	H	2.24755	-2.63629	2.17051
H	1.87435	-2.8667	-2.84168	H	-0.06054	-3.05924	-2.56976	H	3.22338	-1.40248	1.41175
H	-0.62391	-2.30339	-1.627	H	-1.71865	-1.22518	-1.68318	H	2.61619	1.05964	1.50396
H	3.61006	0.3135	2.68552	H	3.44524	-1.42642	2.7732	H	0.77227	-1.94243	-2.77302
H	3.98508	1.16966	1.19404	H	4.28836	-1.31766	1.23169	H	-0.83766	-2.53765	-3.129
H	3.75879	-0.25927	-1.18056	H	3.11319	-2.44551	-0.95324	H	0.51315	-3.65579	0.83587
H	-2.0653	-0.65386	0.55875	H	-1.6858	0.90305	1.57648	H	-0.16816	0.49415	0.6101
H	-0.40097	-0.4836	0.04169	H	-0.74587	-0.16868	0.53699	H	-1.43492	0.96555	1.71972
H	-2.09175	-0.25822	-3.28415	H	-0.61507	1.593	-2.04997	H	0.37713	1.72085	3.48243
H	-2.68842	-1.3259	-2.03434	H	-2.18537	1.21279	-2.70865	H	1.64455	2.79534	2.95114
H	-0.92682	1.13578	-1.6236	H	-2.28944	2.25108	-0.45589	H	-0.63934	3.13663	1.97695
H	-3.87906	3.22924	-1.92677	H	-4.68894	-1.06845	0.38643	H	1.13051	5.33756	-0.251
H	-2.10379	2.99148	-2.37934	H	-2.91779	-1.13718	0.87952	H	0.00724	5.16851	1.19512
H	-6.37305	-0.656	-0.50039	H	-6.25682	3.07875	-1.67604	H	3.71379	3.88868	-2.81864
H	-5.41216	-1.9221	0.29006	H	-5.06894	3.82805	-2.76144	H	3.98794	2.43046	-1.84406
H	-5.64957	-0.37447	1.12593	H	-5.74688	2.24485	-3.19018	H	2.63011	2.49823	-3.02656
H	-1.50223	2.54257	3.50671	H	1.35103	3.918	2.02499	H	-3.50365	0.09004	1.23449
H	-0.08724	1.93589	4.34402	H	2.42355	2.96423	3.03043	H	-4.30275	1.46161	0.43608
H	0.11568	2.93829	2.89204	H	2.57697	2.89274	1.25933	H	-4.62456	-0.16664	-0.11615
H	-1.23968	-0.04953	4.07843	H	-0.49624	2.88523	3.00041	H	-3.66832	0.23281	-2.34326
H	1.65077	0.30884	-4.30309	H	2.20597	-0.40518	-3.48253	H	-0.5558	-1.23833	2.44561
H	0.00433	0.76263	-3.87194	H	1.07885	0.94065	-3.37014	H	0.40783	-2.0346	3.69729

H	1.31639	0.99187	-2.70183	H	1.89232	0.34952	-1.91192	H	-0.09928	-0.36402	3.91541
H	5.4593	-2.14953	0.53051	H	3.03707	-4.79086	1.09977	H	0.64401	-5.3261	-1.20936
H	5.53876	-1.22127	2.01725	H	3.75236	-3.97148	2.47701	H	0.01436	-4.82389	-2.76589
H	5.75035	-0.39849	0.45711	H	4.4628	-3.75789	0.86289	H	1.61503	-4.25455	-2.24032
H	-0.17605	-1.42142	2.33828	H	-0.08432	-0.14069	3.19242	H	-2.67958	-2.56022	-1.4269
H	2.13872	-1.84915	1.70434	H	0.98884	-2.06583	2.13549	H	-1.31949	-4.46202	-0.34434
10b2				10b3				10b4			
C	-2.63144	0.63384	-0.66869	C	-2.19941	0.64704	-1.57602	C	-2.61926	0.2249	-1.09496
C	-2.17786	-0.84378	-0.89693	C	-2.3288	-0.64113	-0.70122	C	-1.87366	-1.14585	-1.04085
C	-1.07807	-1.00873	-1.95695	C	-1.41281	-1.79445	-1.13806	C	-0.57161	-1.20894	-1.84517
C	-1.48566	1.6322	-0.37596	C	-0.75574	1.17715	-1.75092	C	-1.75536	1.45871	-0.73156
C	1.32152	-2.32486	0.05605	C	0.41699	-2.03488	1.71571	C	1.56924	-1.78897	0.71757
C	2.16	-2.14465	1.30089	C	1.31144	-1.32028	2.70297	C	2.11448	-1.31242	2.04249
C	1.68965	-1.01887	2.19286	C	1.32245	0.18193	2.53584	C	1.29409	-0.21209	2.67644
C	2.17751	0.39278	1.87215	C	2.28278	0.77393	1.50597	C	1.5727	1.22375	2.23677
C	-0.67705	-2.44838	-2.34389	C	-1.58262	-3.14769	-0.41493	C	0.10988	-2.59093	-1.93611
C	-0.55453	-3.52551	-1.23855	C	-1.81791	-3.16734	1.11518	C	0.20002	-3.48187	-0.66927
C	0.34613	-3.24283	-0.05667	C	-0.83035	-2.44443	2.00408	C	0.81422	-2.89203	0.57968
C	-0.52359	1.18322	0.72959	C	0.02211	1.33364	-0.43956	C	-0.98614	1.32711	0.58745
C	1.44884	1.6451	2.27397	C	2.0826	2.10595	0.83796	C	0.58319	2.34691	2.34458
C	0.54855	2.22164	1.14468	C	1.4173	1.99753	-0.56386	C	-0.25547	2.60475	1.06201
C	1.39981	2.81985	0.02622	C	2.38157	1.38428	-1.57307	C	0.51768	3.38068	0.00254
C	1.78401	2.15903	-1.08199	C	2.44414	0.07629	-1.88532	C	0.22205	4.66532	-0.27611
C	1.80919	4.24757	0.24315	C	3.34297	2.27162	-2.30505	C	1.59794	2.69647	-0.76533
O	2.39758	4.77412	-0.86078	O	3.33282	3.52118	-1.77739	O	2.52111	3.58694	-1.2091
O	1.6196	4.84454	1.29311	O	4.04623	1.90609	-3.23576	O	1.64868	1.48782	-0.93184
C	2.79441	6.13767	-0.71337	C	4.23152	4.42898	-2.41574	C	3.60905	2.99493	-1.91976
C	-3.69476	0.69617	0.44044	C	-3.11538	1.75369	-1.02715	C	-3.87248	0.16985	-0.2053
O	-3.28104	1.08762	-1.87105	O	-2.69152	0.33917	-2.89352	O	-3.11591	0.40049	-2.43875
C	0.46896	-1.2964	3.02153	C	0.1176	0.91434	3.0512	C	0.00901	-0.63685	3.32535
O	2.72726	-0.37505	2.95209	O	2.55198	0.82466	2.9141	O	2.04156	0.70706	3.49182
C	-0.10926	-4.84742	-1.88591	C	-1.91281	-4.63012	1.58018	C	0.98951	-4.75251	-1.02527
O	-3.32866	-1.58914	-1.32758	O	-3.69031	-1.09189	-0.79228	O	-2.7323	-2.17444	-1.56723
O	-1.87259	-3.73835	-0.7104	O	-3.10023	-2.56591	1.3514	O	-1.09699	-3.94088	-0.27487
H	-1.84818	-1.25885	0.06168	H	-2.1327	-0.37395	0.34283	H	-1.67213	-1.39518	0.00593
H	-0.17789	-0.47653	-1.64487	H	-0.3678	-1.49194	-1.0526	H	0.14552	-0.48919	-1.45112
H	-1.40113	-0.51697	-2.88426	H	-1.57338	-1.99118	-2.20651	H	-0.76815	-0.89334	-2.87796
H	-1.91477	2.60881	-0.11485	H	-0.78561	2.14059	-2.27722	H	-2.39783	2.34863	-0.69798
H	-0.93747	1.81149	-1.30781	H	-0.22124	0.50619	-2.43292	H	-1.05287	1.65117	-1.55132
H	1.56859	-1.67182	-0.77576	H	0.85307	-2.22733	0.7398	H	1.83976	-1.18636	-0.14506
H	2.18232	-3.07624	1.88072	H	1.01285	-1.56454	3.73046	H	2.1918	-2.15195	2.74519
H	3.19724	-1.97429	0.98433	H	2.3255	-1.72541	2.59064	H	3.14595	-0.97431	1.87863
H	2.83591	0.54651	1.02675	H	2.91867	0.11109	0.93279	H	2.34139	1.40843	1.49909
H	0.26545	-2.38883	-2.90471	H	-0.70745	-3.76453	-0.6598	H	1.1186	-2.4298	-2.34011
H	-1.43238	-2.79545	-3.06386	H	-2.44128	-3.64459	-0.88944	H	-0.43324	-3.15828	-2.70558
H	0.17422	-3.88677	0.80654	H	-1.19208	-2.24728	3.01383	H	0.59626	-3.46879	1.48027
H	-0.03107	0.2631	0.41347	H	0.12924	0.35231	0.02377	H	-0.2691	0.51187	0.48856
H	-1.10552	0.932	1.62424	H	-0.57267	1.9406	0.25326	H	-1.68006	1.02994	1.38237
H	0.83295	1.47435	3.16342	H	1.48048	2.77384	1.46326	H	-0.10685	2.17052	3.17742
H	2.19478	2.39312	2.57076	H	3.0627	2.59064	0.74797	H	1.13113	3.26079	2.60878
H	-0.02416	3.04708	1.59153	H	1.20198	3.02302	-0.89458	H	-1.06365	3.27541	1.39459
H	2.39067	2.62064	-1.85594	H	3.14768	-0.30354	-2.62345	H	0.74715	5.23285	-1.03949
H	1.51109	1.12498	-1.2536	H	1.80328	-0.65999	-1.41716	H	-0.56346	5.19491	0.25462
H	3.25036	6.46109	-1.6532	H	4.14058	5.39951	-1.92022	H	4.28774	3.7949	-2.22812
H	1.92439	6.77145	-0.51481	H	5.26459	4.08272	-2.3116	H	4.15876	2.30417	-1.27264
H	3.53769	6.23687	0.08394	H	3.96722	4.55293	-3.47063	H	3.2473	2.48227	-2.81658
H	-3.31744	0.30848	1.39135	H	-2.86473	2.01848	0.00433	H	-3.62622	-0.04661	0.83841
H	-4.034	1.72681	0.59618	H	-3.04589	2.65723	-1.64387	H	-4.41725	1.1204	-0.24246
H	-4.58897	0.12418	0.168	H	-4.16819	1.45077	-1.05673	H	-4.57696	-0.59319	-0.55541
H	-3.83529	0.32903	-2.15027	H	-3.49342	-0.20166	-2.7351	H	-2.37178	0.67556	-3.00303
H	-0.42555	-1.33416	2.39328	H	-0.75182	0.72654	2.41475	H	-0.72516	-0.93643	2.57193

H	0.56523	-2.26318	3.52742	H	-0.12792	0.57612	4.0637	H	0.18221	-1.49383	3.9852
H	0.30692	-0.54492	3.79941	H	0.2722	1.99565	3.10549	H	-0.43602	0.15228	3.93779
H	-0.08536	-5.66402	-1.15391	H	-2.15942	-4.69638	2.64696	H	1.01408	-5.45735	-0.18485
H	-0.81632	-5.1575	-2.66435	H	-2.71497	-5.1563	1.04941	H	0.51175	-5.28716	-1.85495
H	0.88609	-4.76219	-2.33561	H	-0.97406	-5.16936	1.41225	H	2.02177	-4.52285	-1.31182
H	-3.08157	-2.53125	-1.19336	H	-3.79909	-1.71487	-0.03947	H	-3.28471	-1.72783	-2.24333
H	-1.84534	-4.54839	-0.16985	H	-3.35296	-2.76472	2.27101	H	-1.77096	-3.53954	-0.8622
10b5				10b6				10b7			
C	-1.53274	-0.01221	-2.11396	C	-2.90325	0.19738	0.83748	C	-2.89356	-0.09152	-0.74429
C	-1.60907	-1.37915	-1.36375	C	-2.6942	-1.26557	0.3307	C	-2.11255	-1.21838	-1.49327
C	-0.37924	-2.27413	-1.58461	C	-1.41292	-1.93313	0.84894	C	-1.30498	-2.18513	-0.61357
C	-0.31182	0.85605	-1.71712	C	-1.66508	1.12518	0.76251	C	-2.07446	1.18891	-0.42306
C	1.2207	-2.62551	1.41418	C	1.16449	-2.41375	-1.29604	C	1.96259	-2.03755	0.28291
C	1.94796	-1.7808	2.43181	C	2.29841	-1.77825	-2.05797	C	2.83684	-1.36366	1.3064
C	2.35159	-0.3802	2.00234	C	2.41992	-0.28841	-1.83138	C	2.10326	-0.29853	2.08888
C	2.39841	-0.00435	0.51977	C	1.35634	0.62737	-2.42958	C	2.0283	1.10507	1.49243
C	-0.43676	-3.69199	-0.98002	C	-1.2218	-3.37368	0.33641	C	-0.35203	-3.08921	-1.42025
C	-0.85035	-3.81478	0.50428	C	0.21098	-3.92203	0.55124	C	0.53831	-3.99977	-0.54032
C	-0.08282	-2.95234	1.48447	C	1.31105	-3.25744	-0.25871	C	1.48937	-3.29091	0.39859
C	-0.24542	1.21182	-0.22564	C	-1.03183	1.28987	-0.63101	C	-0.81912	1.00544	0.4361
C	2.33872	1.39606	-0.00957	C	1.05996	2.0148	-1.93857	C	0.98642	2.12336	1.85883
C	0.9645	2.07914	0.20829	C	0.32564	2.03932	-0.57691	C	-0.0952	2.33663	0.76636
C	0.86518	3.4965	-0.35749	C	0.24106	3.48015	-0.07937	C	0.48096	3.10317	-0.42606
C	1.58814	3.96512	-1.39237	C	-0.75003	4.33046	-0.40813	C	0.85942	2.54285	-1.591
C	-0.10172	4.39442	0.361	C	1.36161	3.91168	0.81887	C	0.58268	4.58789	-0.2271
O	-0.22825	5.59596	-0.25757	O	1.16962	5.17007	1.29064	O	1.22283	5.19146	-1.26054
O	-0.70143	4.05865	1.37269	O	2.32007	3.20051	1.08479	O	0.14564	5.16087	0.76076
C	-1.14948	6.48361	0.37608	C	2.20779	5.63217	2.15466	C	1.36276	6.6046	-1.11285
C	-2.84165	0.77509	-1.93736	C	-3.42345	0.18809	2.28092	C	-3.63832	-0.61744	0.48554
O	-1.41492	-0.27934	-3.52357	O	-3.93992	0.78626	0.031	O	-3.9015	0.34914	-1.69062
C	2.32785	0.65619	3.08818	C	3.2818	0.14351	-0.68377	C	1.13145	-0.78555	3.12287
O	3.5817	-0.33886	1.24782	O	2.64875	0.46909	-3.0326	O	2.93336	0.7741	2.55929
C	-0.76065	-5.28428	0.94047	C	0.2279	-5.42862	0.26226	C	1.33866	-4.96574	-1.42167
O	-2.76145	-2.09629	-1.83201	O	-2.69435	-1.23661	-1.10968	O	-1.27039	-0.61908	-2.48665
O	-2.23181	-3.43528	0.58536	O	0.52949	-3.74099	1.93276	O	-0.31838	-4.78202	0.29852
H	-1.75877	-1.17815	-0.29861	H	-3.56806	-1.86505	0.61628	H	-2.83471	-1.82649	-2.05513
H	0.51161	-1.77044	-1.21025	H	-1.42237	-1.94404	1.94486	H	-2.0033	-2.81376	-0.04775
H	-0.2201	-2.40663	-2.66266	H	-0.54907	-1.33571	0.54911	H	-0.73418	-1.63219	0.13013
H	-0.33305	1.77616	-2.3154	H	-0.90302	0.76866	1.46679	H	-2.73904	1.90808	0.07634
H	0.60828	0.34841	-2.02826	H	-1.96536	2.12121	1.11263	H	-1.81156	1.6799	-1.36793
H	1.83584	-3.00277	0.59966	H	0.17304	-2.14228	-1.65009	H	1.69653	-1.4273	-0.57675
H	1.3233	-1.70095	3.3314	H	3.24948	-2.2646	-1.80773	H	3.25434	-2.10016	2.00426
H	2.84644	-2.32592	2.74827	H	2.13564	-1.98281	-3.12431	H	3.69757	-0.9291	0.78214
H	2.08763	-0.71598	-0.22952	H	0.51435	0.18342	-2.94699	H	2.48621	1.29593	0.53021
H	0.5457	-4.15837	-1.13192	H	-1.48658	-3.4616	-0.72284	H	0.28394	-2.48936	-2.08171
H	-1.14357	-4.27577	-1.58616	H	-1.93675	-4.00262	0.88424	H	-0.97475	-3.71783	-2.07112
H	-0.65321	-2.58457	2.33622	H	2.32675	-3.50789	0.04531	H	1.78663	-3.87793	1.26753
H	-0.23793	0.29343	0.37066	H	-1.73205	1.7859	-1.31222	H	-0.11904	0.33823	-0.07047
H	-1.16695	1.73361	0.0577	H	-0.84571	0.30029	-1.0517	H	-1.10756	0.52363	1.37696
H	3.12422	2.01054	0.44883	H	1.99156	2.59106	-1.86885	H	0.48388	1.85089	2.793
H	2.57933	1.34419	-1.07763	H	0.44904	2.52164	-2.6969	H	1.49636	3.07258	2.06631
H	0.87452	2.17834	1.2983	H	0.93225	1.46628	0.1355	H	-0.86649	2.97585	1.21971
H	1.49603	4.98593	-1.75272	H	-0.78865	5.35234	-0.04252	H	1.25219	3.12574	-2.41918
H	2.29399	3.34718	-1.93549	H	-1.56222	4.03573	-1.06423	H	0.79224	1.47407	-1.76807
H	-1.17483	7.41296	-0.19961	H	1.95642	6.64762	2.47315	H	1.88895	6.98649	-1.99208
H	-2.15557	6.05257	0.38177	H	2.27762	4.99799	3.04403	H	0.38025	7.08428	-1.06215
H	-0.82039	6.71585	1.3938	H	3.16421	5.66309	1.62329	H	1.9569	6.8407	-0.22442
H	-3.0858	0.93438	-0.8829	H	-2.68679	-0.21937	2.97985	H	-2.9573	-0.95169	1.27328
H	-2.77606	1.75194	-2.43031	H	-3.68466	1.20144	2.60771	H	-4.29112	0.15539	0.90753
H	-3.68517	0.25736	-2.40811	H	-4.34305	-0.40289	2.36078	H	-4.29198	-1.45382	0.21263
H	-2.08235	-0.97315	-3.7016	H	-3.76241	0.45819	-0.8745	H	-4.45102	1.02094	-1.24885

H	1.29539	0.87054	3.38169	H	2.81404	-0.12966	0.26724	H	0.20127	-1.11414	2.64998
H	2.86553	0.29469	3.97123	H	4.25977	-0.34671	-0.73429	H	1.54944	-1.6354	3.67304
H	2.79791	1.59576	2.78229	H	3.46224	1.22231	-0.6782	H	0.88706	-0.01806	3.86332
H	-1.10938	-5.41561	1.97203	H	1.21428	-5.86247	0.46671	H	1.91216	-5.67521	-0.81289
H	-1.40492	-5.91491	0.31665	H	-0.47753	-5.95828	0.91318	H	0.67066	-5.57032	-2.04625
H	0.26425	-5.66552	0.87496	H	-0.03326	-5.64386	-0.77962	H	2.03417	-4.43251	-2.07891
H	-2.93938	-2.76008	-1.13114	H	-2.9899	-2.1138	-1.41175	H	-1.87397	-0.07145	-3.02651
H	-2.54771	-3.67413	1.47512	H	1.41871	-4.10092	2.08664	H	-0.87708	-5.32499	-0.28244

Detailed DP4+ probability for compound **10**. Isomer 1 is 1R*,3R*,4R*,8R*,11S*,12R*, isomer 2 is 1S*,3R*,4R*,8S*,11S*,12R*.

Functional	Solvent?	Basis Set	Type of Data
B3LYP	PCM	6-311+G(d,p)	Unscaled Shifts

	Isomer 1	Isomer 2	Isomer 3	Isomer 4	Isomer 5	Isomer 6
sDP4+ (H data)	95.75%	4.25%	-	-	-	-
sDP4+ (C data)	0.44%	99.56%	-	-	-	-
sDP4+ (all data)	9.07%	90.93%	-	-	-	-
uDP4+ (H data)	0.00%	100.00%	-	-	-	-
uDP4+ (C data)	29.85%	70.15%	-	-	-	-
uDP4+ (all data)	0.00%	100.00%	-	-	-	-
DP4+ (H data)	0.05%	99.95%	-	-	-	-
DP4+ (C data)	0.19%	99.81%	-	-	-	-
DP4+ (all data)	0.00%	100.00%	-	-	-	-

Table S15.Calculation process of 11

Important thermodynamic parameters (a.u.) of the optimized **11** with simplified structures at B3LYP/6-31+G(d,p) level in the gas phase.

NO.	E+ZPE	G	P%	NO.	E+ZPE	G	P%	NO.	E+ZPE	G	P%
11a1	-1272.072421	-1271.594905	22.88%	11a4	-1272.070719	-1271.592029	1.09%	11a7	-1272.074009	-1271.59462	16.92%
11a2	-1272.072421	-1271.594906	22.91%	11a5	-1272.07492	-1271.594567	15.995	11a8	-1272.072708	-1271.593655	6.08%
11a3	-1272.07238	-1271.593428	4.78%	11a6	-1272.07238	-1271.593417	4.73%	11a9	-1272.072632	-1271.592544	1.88%

Optimized Z-Matrixes of **11** with simplified structures in the Gas Phase (Å) at B3LYP/6-31+G(d,p) level.

11a1				11a2				11a3			
O	-1.90164	-0.49484	-2.22072	O	1.07693	0.74045	2.45219	O	-1.70622	-0.61758	-2.13211
O	-1.31109	0.72476	0.23389	O	-1.24812	0.93937	0.87496	O	-0.96187	0.89282	0.1104
O	-0.17064	-2.68679	-2.97488	O	3.76033	0.84847	1.23356	O	0.02064	-2.86945	-2.75659
O	3.67078	-2.95725	2.89898	O	1.66062	0.07922	-5.09715	O	2.03297	-3.48303	3.37172
O	3.869	-0.95158	1.81524	O	0.69507	-1.54534	-3.80672	O	0.30345	-1.98487	3.52839
C	-1.11114	1.63508	1.32926	C	-2.51812	0.53847	0.33555	C	-0.72085	1.90604	1.10248
C	-2.46265	0.82263	-1.96488	C	-0.18818	0.28071	2.98593	C	-2.26921	0.71627	-1.99139
C	1.1869	0.58895	2.12567	C	-1.48376	-0.21426	-1.99072	C	1.62788	0.95827	1.86991
C	-0.61743	-0.34003	-2.87443	C	1.96297	-0.37573	2.24452	C	-0.47399	-0.53251	-2.88805
C	1.34706	-0.74298	-0.09609	C	0.85098	-0.43757	-0.85113	C	1.64869	-0.58431	-0.20673
C	0.41297	1.81438	1.57407	C	-2.316	-0.57222	-0.73831	C	0.81202	2.1208	1.2452
C	0.98853	-0.77102	1.40916	C	-0.07897	0.39772	-1.77212	C	1.38893	-0.46884	1.31672
C	-2.56165	1.01992	-0.4225	C	-1.31888	1.10914	2.30774	C	-2.26199	1.09319	-0.48014
C	0.3737	-1.4566	-2.43385	C	2.76988	-0.16766	0.92616	C	0.56668	-1.57807	-2.38865
C	-1.77441	2.91796	0.82493	C	-3.30856	0.00786	1.53599	C	-1.43051	3.12335	0.50717
C	3.18511	-1.8648	2.25454	C	0.96983	-0.37308	-4.0188	C	1.39246	-2.31992	3.08662
C	0.47596	-1.71303	-0.91014	C	1.97678	0.42345	-0.26464	C	0.76845	-1.66382	-0.85515
C	1.74602	-1.27721	-3.08282	C	3.5684	-1.40663	0.52016	C	1.89338	-1.45027	-3.13756
C	-2.9777	2.41358	0.04943	C	-2.74964	0.77739	2.71202	C	-2.6678	2.52897	-0.14312
C	1.69179	-1.91145	2.14229	C	0.58693	0.74824	-3.1025	C	2.18352	-1.49115	2.12419
C	-3.86643	0.84474	-2.57985	C	-0.13819	0.54089	4.49697	C	-3.71333	0.65527	-2.50117
C	-0.20931	1.11126	-2.70906	C	1.16075	-1.65317	2.42351	C	-0.08005	0.93177	-2.93156
C	-1.83236	1.11581	2.58582	C	-3.19524	1.78519	-0.24956	C	-1.36728	1.51069	2.44206
C	-1.54388	1.81113	-2.69133	C	-0.25886	-1.21589	2.6913	C	-1.42033	1.61994	-2.89242

C	0.98761	-2.92958	2.67353	C	0.82046	2.03081	-3.44203	C	3.51869	-1.64416	2.03891
C	5.09246	-2.97479	3.02727	C	2.06781	-0.94718	-6.00187	C	1.31391	-4.33635	4.26177
O	0.61907	2.90575	2.48455	O	-3.6021	-1.03652	-1.18001	O	1.05566	3.30066	2.02607
H	-1.10679	-2.70003	-2.69338	H	3.25851	1.59731	1.6106	H	-0.89142	-2.87385	-2.40391
H	0.93526	0.47122	3.18923	H	-2.0761	0.46603	-2.61875	H	1.44753	0.95701	2.95438
H	2.24973	0.86071	2.12786	H	-1.40235	-1.12975	-2.58986	H	2.69279	1.21266	1.77127
H	-0.8374	-0.50063	-3.94017	H	2.68166	-0.32604	3.07464	H	-0.7643	-0.82373	-3.90829
H	2.40105	-0.99359	-0.26044	H	1.27958	-1.29256	-1.38337	H	2.70109	-0.80086	-0.4216
H	1.23822	0.26694	-0.47243	H	0.2706	-0.86026	-0.03655	H	1.46358	0.37478	-0.67381
H	0.88019	2.11233	0.62798	H	-1.83409	-1.43312	-0.26207	H	1.22391	2.32452	0.24962
H	-0.08475	-0.97915	1.48239	H	-0.25891	1.35123	-1.25988	H	0.32891	-0.68844	1.47503
H	-3.27269	0.27897	-0.03406	H	-1.12039	2.17192	2.49512	H	-2.92844	0.39543	0.04379
H	-2.0465	3.61609	1.62248	H	-4.39185	0.126	1.43028	H	-1.66863	3.89816	1.2423
H	-1.1076	3.44538	0.13174	H	-3.11731	-1.06027	1.69178	H	-0.81157	3.58141	-0.27424
H	0.87094	-2.72677	-0.75031	H	2.68604	0.6697	-1.06732	H	1.20776	-2.64191	-0.6113
H	-0.54023	-1.75719	-0.49995	H	1.57061	1.39669	0.04244	H	-0.21997	-1.69095	-0.38016
H	2.24422	-0.3606	-2.75364	H	4.17878	-1.76495	1.35706	H	2.39069	-0.49591	-2.94101
H	2.39859	-2.12723	-2.85243	H	2.93023	-2.227	0.18173	H	2.57694	-2.26013	-2.85761
H	1.65722	-1.246	-4.17494	H	4.2687	-1.16843	-0.28913	H	1.73826	-1.54001	-4.21898
H	-3.84743	2.33149	0.71228	H	-3.31438	1.70956	2.83586	H	-3.5018	2.51669	0.56876
H	-3.26774	3.09519	-0.75488	H	-2.83913	0.22734	3.65187	H	-3.00572	3.116	-1.00153
H	-4.51231	0.0984	-2.10341	H	-0.02961	1.61226	4.7008	H	-4.31137	-0.0354	-1.89577
H	-4.34012	1.82646	-2.48575	H	-1.03369	0.17968	5.01044	H	-4.19493	1.63759	-2.4871
H	-3.83072	0.57873	-3.64278	H	0.73222	0.05333	4.95124	H	-3.74913	0.26733	-3.52589
H	0.43881	1.45768	-3.51967	H	1.54937	-2.21258	3.28321	H	0.49582	1.18138	-3.82795
H	0.30016	1.31927	-1.77302	H	1.21068	-2.33595	1.57176	H	0.50055	1.25668	-2.07337
H	-2.90637	0.98482	2.41709	H	-3.32454	2.56213	0.51226	H	-2.44706	1.35559	2.34721
H	-1.46448	0.12978	2.88397	H	-4.17865	1.55301	-0.67017	H	-0.97146	0.56338	2.81799
H	-1.70508	1.80107	3.4301	H	-2.57954	2.23833	-1.03354	H	-1.2036	2.27898	3.20469
H	-1.87405	1.95422	-3.72852	H	-0.72158	-1.78795	3.50304	H	-1.82846	1.64169	-3.91132
H	-1.49413	2.80302	-2.23626	H	-0.85351	-1.43109	1.8028	H	-1.35097	2.65768	-2.55789
H	1.45527	-3.75599	3.19984	H	1.28994	2.31468	-4.37884	H	4.05867	-2.36872	2.64176
H	-0.09449	-2.96929	2.59096	H	0.54713	2.84684	-2.77959	H	4.11916	-1.03802	1.36919
H	5.56696	-2.98148	2.04094	H	2.61391	-0.47676	-6.8242	H	1.16434	-3.8445	5.22808
H	5.43494	-2.11907	3.61756	H	2.73661	-1.65556	-5.50293	H	0.35518	-4.6293	3.82233
H	5.37384	-3.89178	3.55237	H	1.19516	-1.46209	-6.41582	H	1.9106	-5.23861	4.422
H	1.58088	3.02305	2.58119	H	-3.44725	-1.75383	-1.81996	H	2.01916	3.41966	2.08633
11a4				11a5				11a6			
O	-1.61313	0.44926	-2.05922	O	1.619	1.86421	1.6055	O	1.20995	0.66634	2.29233
O	-1.02796	0.19406	0.67261	O	-0.76272	1.99504	0.14492	O	-1.16507	0.57332	0.79361
O	0.55239	-0.20866	-3.86883	O	3.86303	1.53907	-0.05338	O	3.85604	0.78615	0.97875
O	1.48039	-3.40348	-0.23151	O	-0.1298	-3.97085	-1.57594	O	1.02882	1.77249	-4.83493
O	3.35058	-4.22973	0.80231	O	-2.1145	-2.79991	-1.48277	O	-0.7862	2.1205	-3.47806
C	-0.9373	0.33262	2.10083	C	-2.06101	1.50761	-0.23911	C	-2.42747	0.0657	0.3336
C	-2.48974	1.11703	-1.10996	C	0.36217	1.64135	2.2929	C	-0.00913	0.19837	2.91858
C	1.66562	-0.13876	2.23861	C	-0.93685	0.3424	-2.2712	C	-1.41479	-0.84324	-1.94517
C	-0.55007	1.35781	-2.43825	C	2.41039	0.65474	1.6223	C	2.15014	-0.41562	2.16306
C	1.85295	0.12533	-0.3243	C	1.15683	-0.85166	-1.21527	C	0.94221	-0.82189	-0.86222
C	0.48398	0.83936	2.47126	C	-1.8725	0.19521	-1.05104	C	-2.1929	-1.12891	-0.63948
C	1.75162	-0.85926	0.8656	C	0.1873	-0.72119	-2.42447	C	-0.05225	-0.119	-1.83021
C	-2.39735	0.36637	0.25227	C	-0.75294	2.40999	1.52648	C	-1.20734	0.88737	2.20155
C	0.78306	0.59206	-2.68273	C	2.99761	0.4021	0.2022	C	2.90739	-0.29802	0.80394
C	-2.02282	1.36622	2.4063	C	-2.80798	1.2563	1.07368	C	-3.15405	-0.38837	1.60365
C	2.62287	-3.29844	0.48882	C	-0.98282	-2.97231	-1.90756	C	0.1595	1.43939	-3.84613
C	1.18979	-0.43087	-1.59348	C	1.99294	0.41266	-0.97799	C	2.0523	0.13587	-0.41211
C	1.92382	1.54796	-3.03175	C	3.91191	-0.8242	0.17855	C	3.75373	-1.53263	0.49114
C	-3.12877	1.01778	1.42668	C	-2.17717	2.23584	2.03873	C	-2.60354	0.51582	2.68491
C	2.89249	-1.87043	0.83748	C	-0.32809	-2.07232	-2.87877	C	0.51753	0.1219	-3.22823
C	-3.91618	1.01513	-1.66116	C	0.5321	2.19717	3.7137	C	0.07304	0.61898	4.39134
C	-0.56707	2.4988	-1.4391	C	1.57795	-0.43216	2.27895	C	1.42544	-1.71014	2.48947
C	-1.26314	-1.00552	2.78772	C	-2.75617	2.605	-1.05634	C	-3.18531	1.21635	-0.34124

C	-2.02883	2.57845	-1.08072	C	0.17743	0.12705	2.3123	C	-0.00364	-1.32301	2.78354
C	4.16933	-1.57084	1.14318	C	-0.20976	-2.53612	-4.13498	C	1.27406	-0.75958	-3.90913
C	1.16158	-4.73558	-0.63182	C	-0.66747	-4.89837	-0.63405	C	0.75746	3.03501	-5.44312
O	0.50188	1.22644	3.8536	O	-3.14665	-0.29345	-1.47882	O	-3.46017	-1.70799	-0.98924
H	-0.27297	-0.70149	-3.69038	H	3.3198	2.32511	0.16001	H	3.32884	1.54847	1.28888
H	1.64745	-0.8954	3.03602	H	-0.44149	1.31955	-2.24967	H	-2.07322	-0.27773	-2.61917
H	2.59028	0.42684	2.41797	H	-1.53944	0.34235	-3.18987	H	-1.26459	-1.80454	-2.45764
H	-0.87599	1.7639	-3.40717	H	3.25761	0.87754	2.28561	H	2.88784	-0.24771	2.96034
H	2.9009	0.36669	-0.54275	H	1.84504	-1.68176	-1.42034	H	1.37516	-1.72174	-1.31046
H	1.40061	1.07095	-0.04983	H	0.61082	-1.12995	-0.31426	H	0.40724	-1.16978	0.01626
H	0.68312	1.75109	1.89559	H	-1.46311	-0.55346	-0.37081	H	-1.64672	-1.91182	-0.10169
H	0.8149	-1.41472	0.77748	H	0.81027	-0.34561	-3.25153	H	-0.25325	0.86977	-1.4017
H	-2.78386	-0.65038	0.10318	H	-0.48905	3.47486	1.51963	H	-1.06886	1.97348	2.27329
H	-2.36012	1.35653	3.44729	H	-3.89193	1.38415	0.98889	H	-4.24466	-0.33547	1.52364
H	-1.66173	2.37726	2.18113	H	-2.63546	0.23787	1.44016	H	-2.90176	-1.4261	1.85103
H	1.88361	-1.15849	-2.03855	H	2.56245	0.60346	-1.89975	H	2.7228	0.32516	-1.26194
H	0.30624	-1.02714	-1.33583	H	1.3489	1.28963	-0.88313	H	1.61661	1.11959	-0.1904
H	2.17304	2.21722	-2.20308	H	4.70735	-0.72858	0.92683	H	4.40319	-1.78135	1.33833
H	2.82604	0.99077	-3.30929	H	3.37329	-1.75556	0.37199	H	3.14715	-2.40974	0.25132
H	1.66278	2.16174	-3.90165	H	4.41499	-0.91343	-0.79137	H	4.41958	-1.34104	-0.35862
H	-3.81799	0.29309	1.87647	H	-2.70351	3.19593	1.97165	H	-3.21711	1.42302	2.74568
H	-3.73204	1.88874	1.15629	H	-2.24752	1.90774	3.07862	H	-2.63121	0.04982	3.67292
H	-4.23001	-0.0321	-1.73853	H	0.71923	3.27673	3.68492	H	0.1294	1.71002	4.47869
H	-4.63711	1.5496	-1.03538	H	-0.34853	2.01515	4.33627	H	-0.7855	0.26903	4.97147
H	-3.97159	1.42292	-2.67727	H	1.39952	1.75254	4.21455	H	0.98229	0.22693	4.86173
H	-0.18493	3.43175	-1.86424	H	1.93018	-0.5843	3.3069	H	1.88007	-2.17317	3.37367
H	-0.00077	2.29681	-0.53476	H	1.63291	-1.40648	1.78823	H	1.47447	-2.4611	1.69703
H	-2.26023	-1.37091	2.52045	H	-2.83367	3.53584	-0.48331	H	-3.33567	2.05322	0.34994
H	-0.57369	-1.79713	2.48092	H	-3.76434	2.30677	-1.36076	H	-4.16567	0.89593	-0.7075
H	-1.21848	-0.91441	3.87785	H	-2.18312	2.8554	-1.95517	H	-2.61653	1.6253	-1.18242
H	-2.54441	3.16122	-1.85519	H	-0.39877	-0.22798	3.17286	H	-0.39388	-1.82994	3.67297
H	-2.20341	3.09052	-0.13167	H	-0.34355	-0.22015	1.42299	H	-0.62497	-1.6637	1.95404
H	4.95317	-2.32486	1.12447	H	-0.58478	-3.51471	-4.42232	H	1.64971	-0.55022	-4.90681
H	4.4735	-0.56539	1.41143	H	0.26407	-1.94483	-4.9124	H	1.53417	-1.72998	-3.50269
H	1.94985	-5.14308	-1.27256	H	0.09042	-5.66218	-0.43942	H	1.5107	3.20704	-6.21695
H	1.00516	-5.37236	0.24452	H	-0.90124	-4.39396	0.30887	H	-0.22944	3.02888	-5.91644
H	0.2316	-4.70292	-1.20612	H	-1.55681	-5.38854	-1.04253	H	0.8296	3.84027	-4.70525
H	1.38706	1.58004	4.04875	H	-3.04276	-1.26234	-1.60083	H	-3.29152	-2.43595	-1.61177
	11a7				11a8				11a9		
O	2.333	0.35591	1.64609	O	-0.4982	2.99243	0.16459	O	-1.89669	2.32312	-0.99989
O	0.38408	1.56813	0.16479	O	-1.93139	0.61467	0.51555	O	-1.62529	0.07881	0.49786
O	2.0178	-1.68966	0.01904	O	0.19415	3.48519	-2.5194	O	-0.70204	2.08568	-3.4874
O	-1.11891	-2.87246	-4.66006	O	1.98	-4.4343	-0.80418	O	2.6623	-4.13664	-1.22326
O	-2.47662	-2.61055	-2.83654	O	2.06319	-2.3255	0.08188	O	3.58089	-3.98842	0.87968
C	-0.81692	2.23709	-0.24053	C	-2.07145	-0.72211	1.02834	C	-1.27712	-0.83172	1.552
C	1.66069	1.48227	2.2586	C	-0.58067	2.41647	1.49238	C	-2.23195	2.4685	0.41316
C	-1.70869	0.20265	-1.6741	C	-1.18021	-1.58346	-1.25166	C	0.32567	-2.00378	-0.09055
C	1.94095	-0.87909	2.29135	C	0.8771	2.99432	-0.28434	C	-0.52162	2.74672	-1.19617
C	-0.78264	-1.89005	-0.41738	C	0.9712	-0.13849	-1.70371	C	1.54499	-0.05624	-1.32329
C	-1.94532	1.19918	-0.51808	C	-1.06014	-1.64024	0.28446	C	0.17658	-1.31411	1.29038
C	-0.56053	-0.8351	-1.53734	C	0.15261	-1.41904	-2.03607	C	1.55253	-1.56394	-0.93647
C	1.11207	2.37628	1.11434	C	-1.89273	1.5829	1.58318	C	-2.57075	1.05402	0.97417
C	1.26187	-1.81227	1.23349	C	0.94272	2.49951	-1.76003	C	0.16674	1.94455	-2.33905
C	-1.1621	3.12896	0.9559	C	-1.75481	-0.6216	2.52299	C	-1.41945	0.01953	2.81245
C	-1.43022	-2.38897	-3.42922	C	1.73711	-3.09689	-0.80839	C	3.08345	-3.48256	-0.11511
C	-0.22627	-1.48496	0.95709	C	0.23586	1.15688	-2.07432	C	0.31281	0.41433	-2.1187
C	1.38432	-3.27493	1.68144	C	2.36846	2.55008	-2.31179	C	1.49537	2.59766	-2.72901
C	0.1865	3.51393	1.52959	C	-2.11936	0.80479	2.87329	C	-2.61107	0.90922	2.49542
C	-0.3319	-1.53012	-2.88235	C	0.99352	-2.6923	-2.03994	C	2.84309	-2.01741	-0.26239
C	2.72023	2.24567	3.06565	C	-0.61352	3.58997	2.48224	C	-3.46937	3.36654	0.5113
C	1.12292	-0.49347	3.5123	C	1.70745	2.28324	0.76975	C	0.12382	2.73462	0.17376

C	-0.49711	3.08638	-1.48065	C	-3.52403	-1.16364	0.80945	C	-2.27556	-2.00327	1.56842
C	0.60045	0.89134	3.19073	C	0.69582	1.59754	1.65424	C	-1.02229	3.16522	1.05028
C	0.83249	-1.38543	-3.54363	C	1.10228	-3.4435	-3.15374	C	3.80429	-1.22961	0.25307
C	-2.12945	-3.70131	-5.23352	C	2.66174	-4.90091	0.35912	C	2.84337	-5.55142	-1.16353
O	-3.16061	1.91697	-0.79745	O	-1.1905	-3.00132	0.70557	O	0.58144	-2.20882	2.32943
H	2.26227	-0.74016	-0.02359	H	-0.65112	3.58805	-2.03664	H	-1.60466	1.92581	-3.14248
H	-1.57629	0.77432	-2.60252	H	-1.83276	-0.75704	-1.55342	H	-0.56305	-1.83765	-0.70727
H	-2.65354	-0.33449	-1.83113	H	-1.68169	-2.49315	-1.60995	H	0.36176	-3.0915	0.05732
H	2.87961	-1.34952	2.60926	H	1.17321	4.05255	-0.28763	H	-0.61029	3.79051	-1.53065
H	-0.3546	-2.85958	-0.69955	H	1.91112	-0.179	-2.26844	H	2.43004	0.12982	-1.94721
H	-1.86146	-2.05793	-0.3038	H	1.24861	-0.11711	-0.65223	H	1.68437	0.55244	-0.43204
H	-2.13939	0.62552	0.39406	H	-0.06002	-1.32018	0.56942	H	0.83882	-0.44528	1.34625
H	0.35513	-0.28124	-1.31362	H	-0.17151	-1.27202	-3.07878	H	1.48163	-2.1005	-1.89317
H	1.96515	2.78342	0.55642	H	-2.7375	2.26002	1.40572	H	-3.53755	0.74522	0.55612
H	-1.77362	3.99745	0.69168	H	-2.29096	-1.35698	3.13148	H	-1.55745	-0.56731	3.72555
H	-1.71229	2.5593	1.71462	H	-0.6859	-0.77057	2.71367	H	-0.53434	0.6516	2.95166
H	-0.39506	-0.42397	1.07335	H	0.03954	1.12424	-3.15616	H	0.35994	-0.07529	-3.10226
H	-0.84155	-1.95867	1.73339	H	-0.7604	1.17596	-1.62584	H	-0.61633	0.04423	-1.68468
H	2.43593	-3.55615	1.8107	H	2.3768	2.32335	-3.38431	H	2.21517	2.59768	-1.90513
H	0.86046	-3.45613	2.62533	H	2.78752	3.55793	-2.20991	H	1.94459	2.08311	-3.58627
H	0.98075	-3.95623	0.92443	H	3.04026	1.84976	-1.80865	H	1.33996	3.63584	-3.04519
H	0.53162	4.45036	1.07517	H	-3.18324	0.84832	3.13717	H	-3.53934	0.40376	2.78841
H	0.14622	3.68612	2.60889	H	-1.56285	1.18356	3.73402	H	-2.5953	1.85608	3.04154
H	3.52865	2.59387	2.41249	H	-1.50522	4.20612	2.3193	H	-4.32086	2.91686	-0.012
H	2.2949	3.11175	3.58179	H	-0.6118	3.25218	3.52253	H	-3.75584	3.55	1.55146
H	3.19235	1.59746	3.81255	H	0.24484	4.25626	2.34028	H	-3.29203	4.33191	0.02327
H	1.78736	-0.42734	4.38285	H	2.25648	3.0283	1.35914	H	0.99251	3.39452	0.24387
H	0.33544	-1.20723	3.7682	H	2.45664	1.59531	0.37163	H	0.41991	1.73822	0.49139
H	-0.08357	2.4679	-2.28426	H	-4.22404	-0.47619	1.29755	H	-3.30713	-1.65798	1.69454
H	0.26986	3.83919	-1.26779	H	-3.70463	-2.16998	1.20021	H	-2.26733	-2.55107	0.62074
H	-1.3839	3.6061	-1.85637	H	-3.78662	-1.15255	-0.25364	H	-2.05345	-2.70736	2.37662
H	0.44312	1.49104	4.0938	H	1.02988	1.51641	2.69367	H	-1.12241	4.2564	0.98091
H	-0.37534	0.81686	2.70565	H	0.56338	0.58234	1.28818	H	-0.85938	2.9315	2.10418
H	1.03521	-1.87229	-4.49202	H	1.70664	-4.34488	-3.19423	H	4.68917	-1.65041	0.72719
H	1.63566	-0.7783	-3.1362	H	0.58263	-3.17651	-4.06882	H	3.75273	-0.14755	0.22608
H	-1.77606	-4.03861	-6.21184	H	2.79946	-5.98125	0.26103	H	3.90487	-5.79867	-1.06297
H	-2.30561	-4.58152	-4.60722	H	3.64751	-4.43224	0.44003	H	2.26551	-5.97711	-0.33713
H	-3.05488	-3.13464	-5.37659	H	2.06528	-4.70825	1.25649	H	2.47532	-5.97995	-2.09981
H	-3.86259	1.25697	-0.93769	H	-0.74569	-3.07747	1.56767	H	1.50626	-2.46846	2.16456

Table S16.Calculation process of 14

Important thermodynamic parameters (a.u.) of the optimized **14** with simplified structures at B3LYP/6-31+G(d,p) level in the gas phase.

NO.	E+ZPE	G	P%	NO.	E+ZPE	G	P%	NO.	E+ZPE	G	P%
14a1	-1270.834877	-1270.385354	3.28%	14b1	-1270.836837	-1270.389845	63.94%	14c3	-1270.8329	-1270.384662	35.83%
14a2	-1270.835048	-1270.386834	15.73%	14b2	-1270.834776	-1270.388579	16.72%	14c4	-1270.832489	-1270.383735	13.41%
14a3	-1270.833283	-1270.385875	5.69%	14b3	-1270.834834	-1270.387593	5.88%	14c5	-1270.831653	-1270.382318	2.99%
14a4	-1270.836387	-1270.387642	37.02%	14b4	-1270.834834	-1270.387591	5.87%	14d1	-1270.831179	-1270.381741	2.90%
14a5	-1270.833195	-1270.387003	18.81%	14b5	-1270.834258	-1270.38715	3.68%	14d2	-1270.832515	-1270.381151	1.55%
14a6	-1270.833283	-1270.385876	5.70%	14b6	-1270.833071	-1270.386609	2.07%	14d3	-1270.8329	-1270.384662	63.97%
14a7	-1270.832464	-1270.385107	2.52%	14c1	-1270.831179	-1270.381741	1.62%	14d4	-1270.832489	-1270.383735	23.95%
14a8	-1270.835278	-1270.386444	10.40%	14c2	-1270.833479	-1270.384855	43.96%	14d5	-1270.831653	-1270.382318	5.34%

Optimized Z-Matrixes of **14** with simplified structures in the Gas Phase (Å) at B3LYP/6-31+G(d,p) level.

14a1				14a2				14a3			
C	-1.57395	1.20305	-2.3382	C	-1.04378	-1.52669	-2.23683	C	0.15485	2.52902	-1.66369
C	-2.52712	0.84332	-1.17381	C	-0.76002	-0.0839	-2.71763	C	-1.09475	2.58543	-0.75388
C	-2.5543	1.74062	0.05458	C	0.57563	0.55553	-2.36791	C	-0.93966	2.26287	0.7249
C	-0.30708	0.32497	-2.32324	C	-0.50767	-1.84969	-0.83447	C	1.13489	1.38973	-1.34284
C	-0.33038	-0.09708	2.66501	C	-0.38451	3.16181	0.21496	C	-2.61628	-1.12324	0.99612

C	0.94938	0.43781	3.2463	C	0.03271	3.33398	1.6487	C	-2.09205	-2.46386	1.42793
C	1.87329	0.95455	2.16734	C	0.12396	2.03415	2.41726	C	-0.7021	-2.77056	0.9154
C	2.51461	-0.09001	1.25806	C	-1.10471	1.12667	2.46762	C	-0.44863	-2.71905	-0.59072
C	-3.41872	1.19386	1.20047	C	0.77416	1.94829	-2.98393	C	-2.23597	2.42616	1.53219
C	-2.78221	0.03763	2.00972	C	-0.00309	3.1006	-2.29943	C	-3.28841	1.30511	1.34277
C	-1.52707	0.51141	2.71763	C	0.425	3.26671	-0.85325	C	-2.74136	-0.04021	1.78286
C	0.63791	0.54738	-1.13361	C	-1.20363	-1.07665	0.30266	C	0.57622	-0.02151	-1.61391
C	2.97302	0.17989	-0.14311	C	-1.06464	-0.34026	2.77053	C	0.90004	-2.54912	-1.22103
C	1.95554	-0.26842	-1.22958	C	-0.4277	-1.18068	1.6394	C	1.49502	-1.13417	-1.04003
C	1.80671	-1.79019	-1.25574	C	-0.23647	-2.62308	2.09687	C	2.91196	-1.11806	-1.59927
C	0.78341	-2.47112	-0.70521	C	-1.25839	-3.42397	2.45387	C	3.21221	-0.95254	-2.90098
C	2.90483	-2.51572	-1.97879	C	1.17233	-3.12683	2.10112	C	4.07192	-1.27221	-0.67078
O	2.73946	-3.86161	-1.92648	O	1.2906	-4.26086	2.83687	O	3.67496	-1.87031	0.47972
O	3.83076	-1.94177	-2.53411	O	2.08823	-2.56146	1.52065	O	5.21201	-0.91471	-0.92692
C	3.7628	-4.60036	-2.59387	C	2.61849	-4.78166	2.89526	C	4.73136	-2.08981	1.41481
O	-2.28845	0.86294	-3.54382	O	-2.45837	-1.74417	-2.23701	O	-0.27074	2.38245	-3.02206
C	-1.26747	2.69358	-2.44598	C	-0.43895	-2.48508	-3.26659	C	0.86575	3.88119	-1.556
C	-3.78125	-0.49292	3.04043	C	0.25221	4.41083	-3.05213	C	-4.54079	1.63124	2.1634
C	1.69477	2.38733	1.76652	C	1.50504	1.52206	2.69288	C	0.41105	-2.66855	1.91238
O	-3.2599	-0.15139	-1.26074	O	-1.58716	0.4945	-3.43488	O	-2.17577	2.9645	-1.22354
O	-2.44339	-1.04375	1.14286	O	-1.4092	2.8754	-2.33932	O	-3.70504	1.20795	-0.01579
O	3.25332	0.57951	2.29211	O	-0.74052	1.97178	3.5662	O	-0.6274	-3.95843	0.10686
H	-1.53296	1.92775	0.39273	H	0.68922	0.59882	-1.28245	H	-0.54362	1.25185	0.84211
H	-2.98618	2.69667	-0.2629	H	1.35652	-0.10402	-2.76321	H	-0.19905	2.96377	1.12656
H	-0.616	-0.72658	-2.36196	H	-0.65547	-2.92112	-0.64523	H	2.02855	1.52221	-1.96567
H	0.24628	0.49201	-3.25753	H	0.57382	-1.67352	-0.79932	H	1.46997	1.46297	-0.30129
H	-0.23902	-1.03847	2.12655	H	-1.44314	2.95933	0.05811	H	-2.94156	-1.06287	-0.04173
H	0.743	1.23347	3.97232	H	0.99403	3.86079	1.69826	H	-2.10694	-2.53667	2.5227
H	1.43293	-0.3686	3.81188	H	-0.69093	4.00359	2.13133	H	-2.79663	-3.22872	1.07637
H	2.31812	-1.14053	1.43472	H	-1.98766	1.40349	1.90403	H	-1.24954	-2.40663	-1.25026
H	-3.64798	2.02427	1.88193	H	1.84777	2.17889	-2.95513	H	-1.96558	2.48311	2.59531
H	-4.38016	0.86105	0.78736	H	0.49445	1.90409	-4.04498	H	-2.68808	3.39324	1.27455
H	-1.61701	1.44094	3.27702	H	1.47841	3.48864	-0.69215	H	-2.43612	-0.11143	2.82518
H	0.90537	1.60899	-1.09031	H	-1.28735	-0.02179	0.02	H	-0.41274	-0.10254	-1.15051
H	0.11387	0.31489	-0.20338	H	-2.22879	-1.44636	0.428	H	0.42179	-0.16554	-2.68976
H	3.2021	1.24094	-0.28889	H	-0.51713	-0.51218	3.70658	H	1.59493	-3.29724	-0.81793
H	3.92379	-0.34712	-0.29253	H	-2.0936	-0.67327	2.95605	H	0.8043	-2.77622	-2.29094
H	2.39434	0.02799	-2.19364	H	0.55703	-0.74101	1.44444	H	1.54383	-0.92306	0.03511
H	0.70836	-3.55386	-0.75286	H	-1.11174	-4.45467	2.76319	H	4.24101	-0.93762	-3.25394
H	-0.02597	-1.97659	-0.17781	H	-2.28537	-3.07216	2.43667	H	2.44332	-0.83094	-3.65677
H	3.53358	-5.66467	-2.49161	H	2.60055	-5.68752	3.50751	H	4.3073	-2.58249	2.29415
H	3.78287	-4.35156	-3.65957	H	2.9706	-5.04772	1.89364	H	5.49491	-2.74576	0.98504
H	4.73669	-4.41021	-2.13191	H	3.29351	-4.05949	3.36519	H	5.169	-1.13714	1.72911
H	-2.71125	-0.00402	-3.37701	H	-2.81687	-1.21571	-2.97908	H	-1.1139	2.87367	-3.10116
H	-0.74168	3.084	-1.57031	H	0.65282	-2.41011	-3.30061	H	0.18066	4.70325	-1.79517
H	-0.65862	2.90192	-3.33328	H	-0.71075	-3.5231	-3.04364	H	1.26069	4.05428	-0.54961
H	-2.19218	3.27074	-2.56471	H	-0.82282	-2.27214	-4.27138	H	1.69284	3.94793	-2.27192
H	-3.35552	-1.32984	3.60704	H	-0.30286	5.24102	-2.59863	H	-5.31058	0.86119	2.03143
H	-4.67871	-0.88273	2.54609	H	-0.09863	4.33724	-4.08822	H	-4.98932	2.57374	1.82804
H	-4.0877	0.28318	3.75017	H	1.31531	4.67492	-3.06629	H	-4.31925	1.71642	3.23288
H	1.67582	3.03038	2.65296	H	2.1066	2.29341	3.18536	H	0.56555	-1.62454	2.20234
H	2.50372	2.75117	1.12673	H	1.50624	0.64445	3.34614	H	0.17068	-3.24048	2.81488
H	0.75007	2.52298	1.23275	H	2.00164	1.25019	1.75602	H	1.35592	-3.0608	1.52598
H	-3.1185	-1.09208	0.43525	H	-1.61496	2.25968	-3.07165	H	-3.54563	2.06377	-0.46298
14a4				14a5				14a6			
C	0.0018	-2.61381	-1.48339	C	-0.91056	-1.70575	-1.93518	C	-1.12298	-1.75635	-2.05704
C	0.18063	-1.48269	-2.52465	C	-0.57566	-0.37007	-2.64051	C	-0.75353	-0.37738	-2.65318
C	1.56019	-0.86923	-2.69759	C	0.81753	0.21785	-2.47116	C	0.60765	0.22011	-2.32805
C	0.07174	-2.10987	-0.02851	C	-0.30392	-1.86043	-0.53141	C	-0.63257	-1.98568	-0.61922
C	1.1528	2.30977	-0.6686	C	0.20397	3.20936	-0.24301	C	-0.26149	3.06244	0.02945
C	1.88104	2.58663	0.61695	C	0.72754	3.55834	1.12193	C	0.13911	3.32808	1.45359

C	1.82733	1.39584	1.54569	C	0.80335	2.37687	2.06353	C	0.14074	2.0918	2.3257
C	0.45801	1.04847	2.12561	C	-0.47297	1.58287	2.34114	C	-1.14117	1.26463	2.42325
C	1.59547	0.32614	-3.66291	C	1.05561	1.49148	-3.296	C	0.88535	1.55034	-3.04365
C	0.99918	1.64541	-3.11342	C	0.39836	2.78045	-2.74235	C	0.15271	2.78661	-2.46481
C	1.73198	2.0719	-1.85694	C	0.93835	3.12152	-1.3657	C	0.56807	3.04594	-1.02837
C	-1.0052	-1.07223	0.33219	C	-0.86466	-0.87884	0.51641	C	-1.30666	-1.08107	0.43071
C	0.06336	-0.32458	2.58291	C	-0.50798	0.1742	2.85251	C	-1.1945	-0.17289	2.8425
C	-1.19357	-0.88595	1.86427	C	-0.01932	-0.86401	1.81633	C	-0.58297	-1.13801	1.80027
C	-2.45151	-0.09641	2.24159	C	0.13859	-2.23854	2.4575	C	-0.521	-2.54268	2.38349
C	-3.09405	0.76073	1.42477	C	1.35521	-2.78316	2.65111	C	-1.61062	-3.28811	2.64731
C	-2.98177	-0.37631	3.61823	C	-1.10209	-2.9628	2.87737	C	0.79613	-3.15957	2.71817
O	-3.98085	0.47877	3.95459	O	-0.84655	-4.27343	3.12542	O	1.78492	-2.59186	1.98236
O	-2.53935	-1.26009	4.33816	O	-2.19964	-2.43352	2.95714	O	0.94663	-4.06376	3.52634
C	-4.50974	0.26846	5.26383	C	-1.99919	-5.02603	3.50471	C	3.0806	-3.13325	2.24174
O	-1.27978	-3.22464	-1.68763	O	-2.33061	-1.83019	-1.8268	O	-2.54648	-1.8997	-2.0725
C	1.03489	-3.71835	-1.71093	C	-0.42857	-2.84075	-2.84359	C	-0.55025	-2.8302	-2.98647
C	1.10571	2.74732	-4.17024	C	0.67484	3.94895	-3.69438	C	0.47713	4.02106	-3.31264
C	2.94208	0.40249	1.42685	C	2.16736	1.80681	2.30471	C	1.48417	1.5256	2.67161
O	-0.78294	-1.11474	-3.20864	O	-1.41279	0.15472	-3.38643	O	-1.5329	0.17734	-3.43913
O	-0.38538	1.48815	-2.81065	O	-1.01714	2.64817	-2.66015	O	-1.26108	2.61947	-2.51022
O	1.48668	1.68061	2.90797	O	0.04274	2.54285	3.27289	O	-0.74363	2.17314	3.45774
H	1.95907	-0.59656	-1.71853	H	1.01101	0.40646	-1.41293	H	0.70781	0.34131	-1.24723
H	2.2056	-1.64963	-3.11621	H	1.52724	-0.54404	-2.81306	H	1.35973	-0.50579	-2.65745
H	-0.05555	-2.97372	0.63734	H	-0.51246	-2.87812	-0.17568	H	-0.84325	-3.02753	-0.34452
H	1.0619	-1.6918	0.17697	H	0.78733	-1.76565	-0.58356	H	0.45621	-1.86583	-0.57116
H	0.06779	2.2609	-0.58895	H	-0.87125	3.04328	-0.30021	H	-1.3261	2.89738	-0.13163
H	2.92439	2.86184	0.42108	H	1.71614	4.0267	1.0363	H	1.12833	3.80206	1.48247
H	1.42149	3.46348	1.09021	H	0.0741	4.33248	1.54467	H	-0.55344	4.07423	1.86416
H	-0.38946	1.68229	1.89842	H	-1.38305	1.84635	1.81495	H	-1.9978	1.54645	1.82261
H	2.64349	0.49892	-3.94321	H	2.14044	1.64887	-3.36661	H	1.96822	1.73159	-3.00992
H	1.0693	0.04798	-4.58578	H	0.7001	1.31586	-4.32009	H	0.62237	1.43806	-4.10395
H	2.81569	2.13775	-1.93542	H	2.00894	3.30862	-1.30765	H	1.62788	3.23352	-0.86628
H	-0.75519	-0.11898	-0.14595	H	-0.87611	0.12993	0.08986	H	-1.30162	-0.04787	0.06737
H	-1.96408	-1.39593	-0.09128	H	-1.90856	-1.12923	0.73695	H	-2.36062	-1.36302	0.54292
H	0.8857	-1.03515	2.45796	H	0.09843	0.09394	3.76414	H	-0.67953	-0.29889	3.80408
H	-0.12384	-0.28057	3.66305	H	-1.53869	-0.04044	3.15638	H	-2.24498	-0.43266	3.02584
H	-1.34472	-1.903	2.25517	H	0.98082	-0.53916	1.49866	H	0.43334	-0.78091	1.60617
H	-3.9971	1.28522	1.72421	H	1.49144	-3.75553	3.1157	H	-1.53349	-4.28523	3.07485
H	-2.74222	0.9783	0.42161	H	2.26141	-2.2626	2.35812	H	-2.6132	-2.92408	2.44697
H	-5.30224	1.00316	5.43095	H	-1.68616	-6.05893	3.68053	H	3.80013	-2.60495	1.6101
H	-4.9435	-0.73308	5.34639	H	-2.42725	-4.63178	4.43173	H	3.35975	-2.97733	3.28856
H	-3.73311	0.41925	6.02023	H	-2.7419	-5.02181	2.7007	H	3.11035	-4.19683	1.98507
H	-1.81415	-2.58578	-2.20341	H	-2.71064	-1.36862	-2.6018	H	-2.86275	-1.42404	-2.86758
H	2.05087	-3.38618	-1.47435	H	0.66125	-2.84399	-2.94838	H	0.54443	-2.81497	-3.00021
H	0.81033	-4.59812	-1.09684	H	-0.74452	-3.81553	-2.45488	H	-0.88166	-3.82963	-2.68254
H	1.02155	-4.05397	-2.75455	H	-0.86324	-2.75016	-3.84619	H	-0.90224	-2.68533	-4.01478
H	0.66618	3.68449	-3.8079	H	0.20368	4.8715	-3.33397	H	-0.04568	4.90795	-2.93441
H	0.546	2.47426	-5.07244	H	0.24626	3.75221	-4.68412	H	0.13712	3.88207	-4.34561
H	2.14527	2.94257	-4.45521	H	1.74777	4.13484	-3.81432	H	1.55122	4.23592	-3.32938
H	3.91045	0.91427	1.42775	H	2.85711	2.59225	2.63139	H	2.12197	2.29874	3.11302
H	2.95901	-0.31095	2.25634	H	2.16884	1.03252	3.07773	H	1.42236	0.70582	3.39357
H	2.85921	-0.15895	0.49281	H	2.56299	1.36884	1.38283	H	1.9796	1.14953	1.77084
H	-0.74683	0.75588	-3.34998	H	-1.31331	1.95402	-3.28322	H	-1.48583	1.9742	-3.21088
14a7				14a8				14b1			
C	0.14801	2.38992	-1.39902	C	-0.08446	-2.47681	-1.53057	C	-2.73107	-0.81391	-1.24207
C	-1.11633	2.42301	-0.508	C	0.06508	-1.33487	-2.56479	C	-2.99661	-0.49938	0.24973
C	-0.96067	2.22493	0.99276	C	1.43319	-0.69891	-2.74781	C	-2.32408	0.71855	0.86163
C	1.2046	1.35619	-0.97858	C	-0.00381	-1.98465	-0.07219	C	-1.26121	-0.65538	-1.66149
C	-2.39041	-1.2517	1.53203	C	1.01366	2.44962	-0.66378	C	0.8344	1.34592	2.72046
C	-1.77677	-2.50929	2.08106	C	1.7575	2.71538	0.61527	C	2.09284	0.549	2.95119
C	-0.35793	-2.74797	1.61356	C	1.72728	1.51011	1.52652	C	2.64329	-0.10415	1.70428

C	-0.08477	-2.80389	0.11057	C	0.36862	1.14403	2.12002	C	2.02574	-1.41937	1.24009
C	-2.27782	2.3515	1.77243	C	1.43751	0.51204	-3.69393	C	-2.67708	0.90778	2.34251
C	-3.2401	1.14232	1.66142	C	0.82983	1.81269	-3.1137	C	-1.66449	1.78885	3.10002
C	-2.6051	-0.11746	2.22128	C	1.5775	2.23162	-1.86329	C	-0.35036	1.05406	3.28679
C	0.75359	-0.10886	-1.13422	C	-1.08966	-0.96392	0.30979	C	-0.29698	-1.58679	-0.90159
C	1.25558	-2.57308	-0.51987	C	-0.01138	-0.23822	2.56084	C	2.12989	-1.94902	-0.16186
C	1.72035	-1.10081	-0.43692	C	-1.27088	-0.79964	1.84625	C	1.18539	-1.20934	-1.13795
C	3.17559	-0.96156	-0.85991	C	-2.52787	-0.02021	2.23591	C	1.60773	-1.43222	-2.58592
C	4.15167	-0.73972	0.04121	C	-3.19209	0.83035	1.42968	C	1.70593	-2.65627	-3.13886
C	3.60294	-1.02241	-2.2892	C	-3.11353	-0.20155	3.60432	C	1.90408	-0.19702	-3.37877
O	2.6374	-1.58309	-3.05318	O	-2.51984	-1.24124	4.2423	O	2.44332	-0.4974	-4.58743
O	4.68325	-0.62022	-2.69685	O	-4.00524	0.49297	4.07024	O	1.68806	0.93336	-2.96541
C	2.95918	-1.65588	-4.44233	C	-3.02237	-1.47871	5.5576	C	2.75293	0.64386	-5.38727
O	-0.24451	2.10621	-2.74523	O	-1.35938	-3.10539	-1.72215	O	-3.11638	-2.17083	-1.49743
C	0.7553	3.79569	-1.39188	C	0.96187	-3.56395	-1.78058	C	-3.63334	0.08044	-2.09265
C	-4.53073	1.43937	2.43227	C	0.89897	2.93241	-4.15461	C	-2.23015	2.18056	4.46975
C	0.72424	-2.47605	2.61318	C	2.84862	0.52861	1.37605	C	3.42449	0.77599	0.77445
O	-2.21506	2.68452	-1.0153	O	-0.91116	-0.97861	-3.23673	O	-3.7647	-1.22286	0.89383
O	-3.61755	0.90291	0.30911	O	-0.54584	1.62537	-2.78873	O	-1.46114	2.98502	2.3512
O	-0.17313	-3.98965	0.91048	O	1.40451	1.76932	2.89826	O	3.27327	-1.37415	1.94783
H	-0.4937	1.25773	1.19076	H	1.84254	-0.43584	-1.77047	H	-1.24544	0.59091	0.75098
H	-0.27976	3.00724	1.34695	H	2.08325	-1.4636	-3.18776	H	-2.6257	1.60475	0.29385
H	2.09372	1.49768	-1.60689	H	-0.11228	-2.85586	0.58736	H	-1.17408	-0.88013	-2.73253
H	1.52012	1.54137	0.05514	H	0.98318	-1.55578	0.12556	H	-0.94792	0.38762	-1.53228
H	-2.7026	-1.30332	0.48961	H	-0.06976	2.39046	-0.56929	H	0.92122	2.19227	2.04251
H	-1.80678	-2.493	3.17782	H	2.79552	3.0027	0.40878	H	2.85153	1.21954	3.3734
H	-2.4148	-3.35194	1.78475	H	1.29676	3.58089	1.10779	H	1.91448	-0.2152	3.71938
H	-0.89753	-2.61415	-0.5806	H	-0.4861	1.77647	1.916	H	1.16661	-1.80777	1.7731
H	-2.02943	2.5115	2.83039	H	2.47863	0.70498	-3.9865	H	-3.67269	1.37011	2.38289
H	-2.79707	3.25846	1.43508	H	0.90199	0.24117	-4.61358	H	-2.7665	-0.06067	2.85171
H	-2.31269	-0.07735	3.2689	H	2.65921	2.30898	-1.95695	H	-0.40544	0.18599	3.94479
H	-0.24366	-0.22229	-0.69541	H	-0.85342	-0.00179	-0.15739	H	-0.5112	-1.53307	0.17032
H	0.6443	-0.35198	-2.19665	H	-2.04764	-1.29274	-0.11183	H	-0.4801	-2.62789	-1.19503
H	2.00568	-3.22121	-0.04826	H	0.81524	-0.94056	2.41886	H	3.16696	-1.86939	-0.51157
H	1.18928	-2.90042	-1.56325	H	-0.18747	-0.20946	3.64316	H	1.89567	-3.02084	-0.1447
H	1.67444	-0.8247	0.6258	H	-1.41235	-1.82405	2.21887	H	1.27442	-0.14248	-0.90113
H	5.19181	-0.62657	-0.25683	H	-4.08785	1.35156	1.76157	H	1.99982	-2.80948	-4.17302
H	3.94309	-0.67027	1.10362	H	-2.87054	1.048	0.41694	H	1.48212	-3.5534	-2.56987
H	2.11139	-2.11017	-4.96249	H	-2.47834	-2.32843	5.97922	H	3.17576	0.29085	-6.33192
H	3.11991	-0.65374	-4.8521	H	-2.85137	-0.60695	6.19699	H	1.84614	1.21632	-5.60648
H	3.84013	-2.28613	-4.59914	H	-4.0865	-1.73236	5.52195	H	3.49757	1.27168	-4.88791
H	-1.1215	2.52264	-2.87068	H	-1.90669	-2.47467	-2.23478	H	-3.84133	-2.36768	-0.8684
H	0.01564	4.54269	-1.70369	H	1.97589	-3.21832	-1.55513	H	-3.56869	-0.19202	-3.15216
H	1.11908	4.07677	-0.3982	H	0.75884	-4.4517	-1.17048	H	-3.37333	1.13879	-1.98909
H	1.58738	3.86734	-2.10152	H	0.93942	-3.89172	-2.82654	H	-4.68418	-0.03825	-1.80324
H	-5.23832	0.60506	2.35339	H	0.45023	3.85618	-3.76982	H	-1.52544	2.81453	5.02129
H	-5.04049	2.31435	2.01215	H	0.32797	2.6639	-5.05107	H	-3.14852	2.76845	4.35699
H	-4.33935	1.62895	3.49413	H	1.92988	3.14977	-4.45455	H	-2.45539	1.30253	5.08492
H	0.75555	-1.40919	2.85615	H	3.81272	1.04838	1.38004	H	2.76133	1.49233	0.27963
H	0.53668	-3.03035	3.53891	H	2.87864	-0.20297	2.18916	H	4.18189	1.34046	1.32848
H	1.71392	-2.77246	2.25309	H	2.76245	-0.01253	0.43045	H	3.94749	0.2093	-0.00147
H	-3.52526	1.73256	-0.20181	H	-0.90866	0.90806	-3.34676	H	-0.86082	3.54654	2.87151
14b2				14b3				14b4			
C	-2.93078	-0.48535	-0.20858	C	-2.76016	0.85786	-1.50506	C	-2.62162	0.785	-1.51594
C	-2.74974	0.82863	0.58893	C	-2.29627	2.04256	-0.62431	C	-2.15457	1.97943	-0.64957
C	-1.81034	1.88603	0.03211	C	-0.82465	2.42345	-0.64163	C	-0.66851	2.2947	-0.59657
C	-1.61955	-1.06286	-0.76672	C	-1.8253	-0.36184	-1.46119	C	-1.73271	-0.46274	-1.3897
C	1.78596	2.77337	0.46333	C	2.06075	1.46741	1.55807	C	2.14943	1.35417	1.65737
C	2.96985	2.06079	1.0656	C	2.27346	0.39511	2.59626	C	2.32858	0.30453	2.7243
C	3.09443	0.61494	0.64329	C	1.86704	-0.98768	2.14195	C	1.84549	-1.06931	2.3197
C	2.24617	-0.42738	1.36531	C	0.39739	-1.37967	2.24387	C	0.35571	-1.37742	2.43289

C	-1.73263	3.13533	0.91839	C	-0.5067	3.60378	0.28584	C	-0.34971	3.50124	0.29537
C	-0.46895	3.98094	0.66583	C	0.96523	3.64034	0.74168	C	1.1145	3.53056	0.7761
C	0.76473	3.2679	1.18586	C	1.24632	2.52406	1.72999	C	1.36398	2.43717	1.79778
C	-0.58373	-1.41717	0.31853	C	-1.66485	-0.96736	-0.05282	C	-1.67427	-1.03828	0.03821
C	1.91213	-1.78129	0.80482	C	-0.23835	-2.48154	1.44529	C	-0.33548	-2.47816	1.67934
C	0.81439	-1.70679	-0.28158	C	-0.55944	-2.05549	-0.0058	C	-0.57029	-2.11256	0.194
C	0.82776	-2.93336	-1.18442	C	-0.87706	-3.28717	-0.84321	C	-0.86242	-3.35891	-0.62709
C	1.14104	-2.83716	-2.4907	C	-2.08044	-3.88908	-0.8852	C	-1.898	-4.18412	-0.38438
C	0.49834	-4.25722	-0.56991	C	0.18402	-3.88596	-1.70845	C	0.03858	-3.74434	-1.75316
O	0.21605	-5.1847	-1.52073	O	1.41165	-3.49404	-1.28613	O	0.72177	-2.66206	-2.20318
O	0.47018	-4.45342	0.63528	O	-0.03438	-4.63124	-2.65202	O	0.1267	-4.87522	-2.20793
C	-0.13858	-6.46375	-0.99467	C	2.48868	-4.04369	-2.04558	C	1.59736	-2.94333	-3.29542
O	-3.51429	-1.47002	0.6516	O	-4.05241	0.43369	-1.0525	O	-3.94839	0.4215	-1.11548
C	-3.92787	-0.22848	-1.33922	C	-2.9254	1.3581	-2.94033	C	-2.69799	1.24572	-2.97181
C	-0.60232	5.34297	1.35632	C	1.27868	4.99138	1.39466	C	1.4358	4.89449	1.39748
C	3.68167	0.35865	-0.71214	C	2.8165	-1.70042	1.22695	C	2.75512	-1.8672	1.43363
O	-3.3898	0.9981	1.6325	O	-3.13151	2.66541	0.04082	O	-2.99531	2.65964	-0.05107
O	-0.35206	4.20663	-0.73784	O	1.79593	3.50212	-0.40928	O	1.96304	3.35048	-0.35608
O	3.63733	-0.23875	1.66465	O	1.37013	-1.82443	3.20106	O	1.30246	-1.83446	3.40957
H	-0.8206	1.43423	-0.05908	H	-0.25121	1.54584	-0.33696	H	-0.15602	1.40826	-0.21765
H	-2.15467	2.16481	-0.9691	H	-0.54214	2.67612	-1.66887	H	-0.31661	2.48582	-1.61559
H	-1.84818	-1.97972	-1.32576	H	-2.2313	-1.13763	-2.1227	H	-2.12492	-1.24439	-2.0533
H	-1.17914	-0.35891	-1.48323	H	-0.83987	-0.09291	-1.86055	H	-0.71887	-0.2327	-1.73828
H	1.78356	2.86795	-0.62	H	2.60662	1.3462	0.62527	H	2.69295	1.19174	0.72942
H	3.87973	2.60193	0.77788	H	3.336	0.38755	2.86912	H	3.39416	0.25261	2.97991
H	2.9243	2.12246	2.16108	H	1.73397	0.65434	3.5169	H	1.81922	0.61813	3.64522
H	1.53052	-0.09494	2.10747	H	-0.31883	-0.64195	2.58433	H	-0.31949	-0.58488	2.73145
H	-2.62787	3.73727	0.71144	H	-0.74553	4.52291	-0.26624	H	-0.56469	4.40482	-0.29107
H	-1.78181	2.86573	1.98145	H	-1.15695	3.5995	1.17015	H	-1.01712	3.53367	1.16637
H	0.79191	3.1398	2.26884	H	0.70344	2.60629	2.67255	H	0.81995	2.55964	2.73524
H	-0.49903	-0.58504	1.02452	H	-1.40472	-0.16763	0.64698	H	-1.48299	-0.22653	0.74687
H	-0.94214	-2.27195	0.90365	H	-2.6234	-1.36693	0.29772	H	-2.65339	-1.45162	0.31028
H	2.81616	-2.24819	0.39445	H	0.41403	-3.36364	1.44262	H	0.25378	-3.40127	1.74915
H	1.58884	-2.41882	1.63592	H	-1.15993	-2.7907	1.95514	H	-1.2916	-2.68913	2.17473
H	1.05652	-0.83622	-0.9076	H	0.33502	-1.57127	-0.41813	H	0.36206	-1.65643	-0.15709
H	1.16779	-3.70112	-3.14853	H	-2.2628	-4.76124	-1.50934	H	-2.07303	-5.07549	-0.98261
H	1.38841	-1.88282	-2.94467	H	-2.92241	-3.53784	-0.29847	H	-2.60438	-3.9932	0.41695
H	-0.34755	-7.12985	-1.83634	H	3.42547	-3.67074	-1.62231	H	2.36219	-3.66741	-2.99754
H	0.69085	-6.88491	-0.41787	H	2.48877	-5.13606	-1.97699	H	1.03056	-3.30827	-4.1577
H	-1.04129	-6.38931	-0.38013	H	2.42711	-3.71915	-3.08904	H	2.09504	-2.01157	-3.57812
H	-4.03232	-0.97547	1.32017	H	-4.48706	1.23263	-0.68808	H	-4.37092	1.2466	-0.79834
H	-4.17948	-1.15987	-1.85906	H	-3.3716	0.58493	-3.57601	H	-3.13967	0.46881	-3.60609
H	-3.53874	0.48495	-2.07272	H	-1.96933	1.66059	-3.3795	H	-1.71129	1.50156	-3.37111
H	-4.86889	0.1731	-0.94503	H	-3.60106	2.2208	-2.97707	H	-3.33982	2.12944	-3.06778
H	0.28609	5.96226	1.18332	H	2.32437	5.03887	1.72178	H	2.47646	4.93679	1.74091
H	-1.45156	5.9035	0.94854	H	1.14377	5.80971	0.67783	H	1.32416	5.6947	0.6566
H	-0.74321	5.24038	2.43786	H	0.63894	5.18349	2.26297	H	0.78442	5.11879	2.24924
H	2.98576	0.67464	-1.49548	H	2.76511	-1.27743	0.21909	H	2.78968	-1.43118	0.43036
H	4.61327	0.92034	-0.838	H	3.84617	-1.59845	1.58564	H	3.77321	-1.87343	1.83696
H	3.91624	-0.69728	-0.87592	H	2.60613	-2.77176	1.15982	H	2.43967	-2.91021	1.33674
H	0.39709	4.81451	-0.8639	H	2.71433	3.61989	-0.11056	H	2.87802	3.45581	-0.0425
14b5				14b6				14c1			
C	-2.71672	0.56606	-1.87486	C	-0.94521	-0.6213	-3.05558	C	1.56243	1.81281	1.74195
C	-2.91429	1.11272	-0.43996	C	-1.54838	0.72881	-2.59773	C	2.49632	0.66698	1.28087
C	-1.83607	2.01144	0.14225	C	-0.62301	1.74876	-1.9559	C	2.96827	0.64683	-0.16717
C	-1.33908	-0.08657	-2.09602	C	-0.12524	-1.32736	-1.95915	C	0.08822	1.36064	1.79578
C	1.3809	2.21898	1.85981	C	1.06357	2.82835	1.09248	C	0.44392	-1.68464	-1.92273
C	2.44129	1.16079	2.0246	C	1.32647	2.06635	2.3655	C	-0.49136	-1.381	-3.0565
C	2.69759	0.38493	0.75367	C	1.77016	0.64347	2.11781	C	-0.97478	0.05354	-3.11464
C	1.64574	-0.62482	0.30865	C	0.71583	-0.37188	1.68965	C	-1.94046	0.67405	-2.09966
C	-2.15004	2.44466	1.58048	C	-1.37388	2.99809	-1.47656	C	3.66725	-0.65823	-0.5734

C	-0.96778	3.14546	2.27953	C	-0.51355	3.92715	-0.59693	C	2.7227	-1.86356	-0.79964
C	0.18403	2.17979	2.47221	C	-0.15644	3.24361	0.70757	C	1.78489	-1.60164	-1.96479
C	-1.07904	-1.29733	-1.17913	C	-0.94361	-1.65253	-0.69461	C	-0.44886	0.77025	0.48639
C	1.50504	-1.04287	-1.12851	C	1.07398	-1.62218	0.9349	C	-2.47064	-0.04043	-0.89356
C	0.30024	-1.97539	-1.41363	C	-0.13911	-2.42069	0.39306	C	-1.98695	0.58149	0.44453
C	0.47204	-3.31297	-0.69012	C	-0.99777	-2.95068	1.54045	C	-2.55608	-0.23699	1.59922
C	-0.2336	-3.70115	0.38927	C	-2.21331	-2.48054	1.88008	C	-1.99697	-1.377	2.04888
C	1.49093	-4.23127	-1.30114	C	-0.49825	-4.09888	2.36608	C	-3.81633	0.29857	2.20617
O	1.7358	-5.30757	-0.51091	O	0.62558	-4.62634	1.81791	O	-4.16094	-0.38391	3.32739
O	2.03133	-4.00944	-2.37573	O	-1.04112	-4.50588	3.38327	O	-4.43478	1.24329	1.73745
C	2.71733	-6.20162	-1.03489	C	1.166	-5.72666	2.54987	C	-5.35335	0.09	3.95327
O	-3.71894	-0.42538	-2.13276	O	-2.01211	-1.49918	-3.4366	O	1.94639	2.12784	3.09372
C	-2.94545	1.69942	-2.8746	C	-0.09408	-0.39028	-4.30384	C	1.74586	3.105	0.94927
C	-1.41246	3.70165	3.63608	C	-1.26731	5.23023	-0.31206	C	3.54336	-3.12454	-1.08308
C	3.66549	0.98221	-0.22181	C	3.22367	0.4276	1.82458	C	-0.16174	0.98694	-3.96215
O	-3.94315	0.82951	0.18333	O	-2.74846	0.95474	-2.78682	O	2.85422	-0.20212	2.08697
O	-0.55797	4.2419	1.46434	O	0.67202	4.25062	-1.32138	O	1.95153	-2.11308	0.37421
O	2.8687	-1.02798	0.94237	O	1.22131	-0.31585	3.03259	O	-2.39028	0.14388	-3.35375
H	-0.89721	1.4545	0.12026	H	-0.13467	1.26348	-1.10859	H	2.12678	0.85216	-0.8326
H	-1.73595	2.89285	-0.49844	H	0.14156	2.03053	-2.68634	H	3.68933	1.46549	-0.27368
H	-1.27471	-0.43765	-3.1345	H	0.25983	-2.27483	-2.35892	H	-0.01976	0.6201	2.59842
H	-0.55537	0.666	-1.96747	H	0.74444	-0.71472	-1.7029	H	-0.53415	2.21154	2.10317
H	1.61086	3.01875	1.15932	H	1.92518	2.99405	0.44957	H	-0.03291	-2.02073	-1.00442
H	3.36766	1.64394	2.35787	H	2.09437	2.59926	2.93915	H	-0.00719	-1.63679	-4.00802
H	2.16011	0.46942	2.8299	H	0.42822	2.06975	2.99698	H	-1.34496	-2.06735	-2.98234
H	0.73633	-0.68787	0.88907	H	-0.28905	-0.01153	1.52022	H	-1.99547	1.75167	-1.99976
H	-3.00878	3.12857	1.54041	H	-1.70909	3.54546	-2.36807	H	4.23548	-0.46984	-1.49427
H	-2.46916	1.57778	2.17443	H	-2.28086	2.70691	-0.92999	H	4.40582	-0.91556	0.19728
H	-0.0235	1.34927	3.14775	H	-1.00296	3.03829	1.36369	H	2.25962	-1.30621	-2.89896
H	-1.18184	-0.98924	-0.13387	H	-1.3553	-0.72562	-0.28252	H	0.04242	-0.18802	0.31638
H	-1.86539	-2.04164	-1.36103	H	-1.80243	-2.26981	-0.98916	H	-0.15986	1.42759	-0.33968
H	1.42443	-0.14374	-1.74844	H	1.7324	-1.35445	0.10181	H	-2.2234	-1.10632	-0.92889
H	2.42863	-1.53658	-1.45671	H	1.67801	-2.27259	1.58002	H	-3.56705	0.01335	-0.9272
H	0.32291	-2.19496	-2.49116	H	0.26733	-3.29526	-0.13433	H	-2.39414	1.60079	0.49563
H	-0.08922	-4.66931	0.86044	H	-2.77957	-2.90647	2.70601	H	-2.42117	-1.95318	2.86599
H	-0.98301	-3.06793	0.85018	H	-2.68922	-1.65749	1.36032	H	-1.09309	-1.78404	1.60471
H	2.84041	-7.02367	-0.32434	H	2.0658	-6.06866	2.03105	H	-5.53958	-0.52599	4.83738
H	2.38572	-6.61789	-1.99133	H	1.4474	-5.41459	3.56056	H	-5.23089	1.129	4.27498
H	3.68024	-5.693	-1.14536	H	0.45028	-6.55408	2.58315	H	-6.20774	-0.0091	3.27633
H	-4.45891	-0.23138	-1.52023	H	-2.77818	-0.9241	-3.64447	H	2.08984	1.27081	3.54409
H	-2.9522	1.31935	-3.90251	H	0.23379	-1.34255	-4.73603	H	1.45937	3.00409	-0.10106
H	-2.17721	2.47531	-2.79802	H	0.79218	0.21536	-4.08969	H	1.15449	3.91738	1.38745
H	-3.92056	2.17264	-2.70955	H	-0.67383	0.12272	-5.0802	H	2.79122	3.434	0.9824
H	-0.58623	4.2152	4.1423	H	-0.66433	5.90877	0.30326	H	4.19582	-3.36205	-0.23474
H	-2.2061	4.44702	3.50879	H	-1.48031	5.76714	-1.24372	H	4.16651	-3.01472	-1.97731
H	-1.7828	2.91236	4.29934	H	-2.215	5.04859	0.20654	H	2.89157	-3.99558	-1.22171
H	3.22314	1.85808	-0.70673	H	3.46765	0.79647	0.8234	H	-0.07128	0.60225	-4.98316
H	4.58042	1.29937	0.28936	H	3.8462	0.96376	2.54841	H	-0.61291	1.98326	-4.02064
H	3.96006	0.27524	-1.00377	H	3.50605	-0.6289	1.87387	H	0.84386	1.09992	-3.54496
H	0.12536	4.72407	1.96151	H	1.15633	4.90758	-0.79172	H	2.51158	-1.92285	1.15416
14c2				14c3				14c4			
C	1.65602	1.87681	1.2335	C	0.30983	2.78609	0.50906	C	1.67136	1.75191	1.36158
C	2.62793	0.71498	0.92025	C	1.09215	1.97388	1.56855	C	2.62987	0.5766	1.05674
C	3.21445	0.62177	-0.47867	C	2.48595	1.48101	1.21507	C	3.21974	0.47062	-0.34008
C	0.25273	1.63706	0.6377	C	-0.23892	1.89889	-0.62746	C	0.26694	1.52689	0.76247
C	0.55664	-1.60705	-1.8031	C	1.3663	-1.82605	0.01203	C	0.52862	-1.71878	-1.67112
C	-0.47771	-1.26879	-2.83535	C	1.40984	-2.35515	-1.39147	C	-0.49817	-1.36736	-2.70662
C	-0.97203	0.15897	-2.76024	C	1.22236	-1.29386	-2.45293	C	-0.97389	0.06694	-2.63279
C	-1.87112	0.67671	-1.63262	C	-0.12703	-0.63087	-2.73984	C	-1.86472	0.59665	-1.50429
C	3.88458	-0.72484	-0.78716	C	3.0567	0.45043	2.19982	C	3.86783	-0.88756	-0.64408
C	2.91965	-1.92597	-0.9333	C	2.38591	-0.94339	2.16522	C	2.88258	-2.07179	-0.79085

C	1.88418	-1.64726	-2.00504	C	2.43722	-1.52423	0.7658	C	1.85623	-1.77847	-1.86785
C	-0.51848	0.46412	1.27004	C	-1.36875	0.94051	-0.20829	C	-0.52462	0.37131	1.40212
C	-2.36265	-0.15454	-0.48717	C	-1.40209	-0.95469	-2.01869	C	-2.36677	-0.22897	-0.35906
C	-2.03145	0.42907	0.9082	C	-2.1036	0.28468	-1.40998	C	-2.03814	0.35709	1.03621
C	-2.84208	-0.35089	1.9482	C	-3.54787	-0.054	-1.03866	C	-2.85874	-0.40846	2.07392
C	-2.41896	-1.4926	2.5244	C	-4.58326	0.53121	-1.67076	C	-2.45241	-1.54247	2.67602
C	-4.17706	0.23894	2.29048	C	-3.77139	-1.05325	0.05413	C	-4.19983	0.11215	2.48583
O	-4.765	-0.41754	3.32283	O	-5.09353	-1.22663	0.30992	O	-4.62882	1.05578	1.61045
O	-4.65477	1.19847	1.70222	O	-2.86399	-1.62993	0.63391	O	-4.81942	-0.27583	3.46544
C	-6.03958	0.10709	3.69459	C	-5.36441	-2.16549	1.35099	C	-5.91504	1.59276	1.92015
O	1.5431	2.01358	2.65477	O	-0.78385	3.45333	1.1496	O	1.55464	1.89941	2.78109
C	2.22634	3.20352	0.72761	C	1.19566	3.89318	-0.06796	C	2.25984	3.06862	0.84989
C	3.70669	-3.18873	-1.29268	C	3.09557	-1.88786	3.13935	C	3.64841	-3.34964	-1.14236
C	-0.21355	1.16458	-3.57467	C	2.46563	-0.5932	-2.91475	C	-0.20139	1.06183	-3.44722
O	2.93504	-0.09383	1.80505	O	0.59967	1.77294	2.68549	O	2.92477	-0.23157	1.94603
O	2.25153	-2.19306	0.29821	O	1.0282	-0.86727	2.58754	O	2.20454	-2.32142	0.43878
O	-2.39416	0.26327	-2.90781	O	0.37778	-1.72165	-3.53055	O	-2.39474	0.19189	-2.77911
H	2.43898	0.84572	-1.2151	H	2.48617	1.08612	0.19655	H	2.45085	0.7062	-1.07981
H	3.97713	1.40553	-0.55498	H	3.14401	2.35772	1.22773	H	3.99569	1.24139	-0.41509
H	-0.34285	2.54653	0.79128	H	-0.6406	2.55841	-1.40819	H	-0.31584	2.44639	0.90515
H	0.33767	1.48703	-0.44144	H	0.57998	1.33281	-1.07911	H	0.35429	1.36577	-0.3148
H	0.17013	-1.85192	-0.81723	H	0.37109	-1.70713	0.43756	H	0.13596	-1.95773	-0.68613
H	-0.07895	-1.45931	-3.84007	H	2.36297	-2.87122	-1.56525	H	-0.09888	-1.56321	-3.71008
H	-1.31347	-1.97114	-2.72199	H	0.64353	-3.13507	-1.48615	H	-1.34358	-2.05842	-2.59553
H	-1.93428	1.73998	-1.43492	H	-0.15847	0.32692	-3.24416	H	-1.91055	1.66052	-1.3059
H	4.456	-0.60792	-1.71788	H	4.1272	0.3386	1.98065	H	4.44413	-0.78237	-1.57317
H	4.61558	-0.94204	0.00296	H	2.98684	0.86026	3.2162	H	4.59243	-1.11575	0.14883
H	2.27143	-1.43319	-2.99964	H	3.43125	-1.67321	0.34825	H	2.25091	-1.5718	-2.86104
H	-0.43454	0.55486	2.36029	H	-2.10381	1.51011	0.37396	H	-0.44227	0.46959	2.49184
H	-0.03668	-0.48044	1.00008	H	-0.96896	0.17648	0.46519	H	-0.05703	-0.58298	1.14096
H	-2.00254	-1.18552	-0.55342	H	-1.23725	-1.71781	-1.25507	H	-2.01181	-1.26195	-0.41995
H	-3.45492	-0.21779	-0.58918	H	-2.08023	-1.40914	-2.75409	H	-3.45825	-0.28882	-0.46837
H	-2.3701	1.47346	0.93183	H	-2.14868	1.04371	-2.20461	H	-2.35789	1.40699	1.0577
H	-3.01282	-2.03231	3.25622	H	-5.6183	0.31124	-1.42646	H	-3.06732	-2.04955	3.41635
H	-1.46136	-1.93788	2.27298	H	-4.42537	1.25857	-2.46104	H	-1.49932	-2.0074	2.44626
H	-6.42194	-0.49019	4.52696	H	-6.44887	-2.22758	1.47702	H	-6.16339	2.3368	1.15824
H	-5.94483	1.14514	4.02851	H	-4.99035	-3.15777	1.07987	H	-6.67547	0.80588	1.8961
H	-6.74457	0.02982	2.86088	H	-4.92521	-1.82901	2.29543	H	-5.89918	2.08861	2.89579
H	1.77048	1.14066	3.03626	H	-0.98908	2.9332	1.95437	H	1.76934	1.02621	3.16961
H	2.26214	3.24712	-0.36571	H	2.0142	3.49206	-0.67434	H	2.29921	3.10581	-0.24354
H	1.62711	4.04911	1.08466	H	0.61043	4.57806	-0.69253	H	1.67038	3.92371	1.20055
H	3.24289	3.35946	1.10752	H	1.63224	4.49951	0.73443	H	3.27721	3.21383	1.23186
H	4.43174	-3.43015	-0.50663	H	3.03156	-1.50603	4.16503	H	4.36569	-3.60116	-0.35237
H	4.24784	-3.079	-2.2388	H	4.15323	-2.01926	2.8861	H	4.19539	-3.25296	-2.08653
H	3.04191	-4.0571	-1.37516	H	2.61877	-2.87551	3.14849	H	2.96855	-4.20627	-1.22461
H	-0.19523	0.87147	-4.62937	H	3.20324	-1.3161	-3.27806	H	-0.18649	0.76804	-4.50179
H	-0.66303	2.16148	-3.51323	H	2.2587	0.10958	-3.72899	H	-0.63735	2.06475	-3.38643
H	0.819	1.24196	-3.22057	H	2.91374	-0.02742	-2.09232	H	0.83192	1.12529	-3.09254
H	2.78179	-1.82728	1.03461	H	0.88596	-0.0398	3.08844	H	2.74401	-1.97358	1.17714
	14c5				14d1				14d2		
C	0.40918	2.85159	0.41893	C	1.9198	-1.96829	1.0964	C	2.11081	-1.63795	1.45115
C	1.10106	2.13024	1.60365	C	2.67381	-0.63406	0.87513	C	2.63222	-0.59994	0.42598
C	2.43734	1.44805	1.34472	C	2.38575	0.5321	1.81164	C	2.5092	0.88162	0.75966
C	-0.1166	1.89728	-0.67127	C	0.74026	-2.1267	0.11443	C	0.71566	-2.17642	1.07011
C	1.27157	-1.79366	-0.11852	C	-0.29398	2.57704	-0.06649	C	-0.71608	1.93795	-1.01404
C	1.44333	-2.24886	-1.53835	C	-1.69338	2.91465	0.35785	C	-1.98711	2.50367	-0.45267
C	1.25325	-1.14553	-2.55761	C	-2.35185	1.88495	1.25291	C	-2.28903	2.09812	0.97443
C	-0.10164	-0.48127	-2.82952	C	-2.78809	0.49377	0.78168	C	-2.71143	0.68681	1.39446
C	2.81394	0.34762	2.34451	C	2.98707	1.86529	1.34495	C	2.8587	1.81508	-0.40802
C	2.06862	-0.99231	2.15816	C	2.24157	2.54397	0.1703	C	1.78136	1.91595	-1.51457
C	2.25872	-1.5378	0.75573	C	0.83104	2.93015	0.57888	C	0.49737	2.51411	-0.96588

C	-1.27871	0.99353	-0.22677	C	-0.26729	-0.97082	0.13184	C	-0.34957	-1.09705	0.85522
C	-1.37849	-0.84202	-2.13254	C	-2.55788	-0.03976	-0.59984	C	-2.8226	-0.483	0.46004
C	-2.06244	0.33789	-1.39728	C	-1.58725	-1.25142	-0.6311	C	-1.81014	-1.61115	0.80082
C	-3.45286	-0.08705	-0.92058	C	-1.40002	-1.69193	-2.07955	C	-2.02943	-2.83665	-0.07232
C	-4.56529	0.44795	-1.45917	C	-0.53111	-1.10863	-2.92759	C	-2.58388	-3.95969	0.42273
C	-3.53513	-1.11328	0.16973	C	-2.27476	-2.82325	-2.52496	C	-1.61094	-2.88882	-1.50533
O	-4.81315	-1.28063	0.59694	O	-1.93005	-3.26557	-3.76076	O	-1.32262	-1.64906	-1.96102
O	-2.5646	-1.71073	0.61273	O	-3.17509	-3.28261	-1.83715	O	-1.53711	-3.91805	-2.16082
C	-4.95521	-2.23086	1.65279	C	-2.72793	-4.35096	-4.23348	C	-0.85936	-1.61079	-3.31189
O	-0.69405	3.62066	0.91907	O	2.85651	-3.01774	0.78792	O	3.01392	-2.75754	1.39132
C	1.38121	3.86502	-0.19315	C	1.50384	-2.20001	2.54727	C	2.16463	-1.14345	2.89537
C	2.56809	-2.01375	3.18507	C	3.00062	3.79619	-0.27655	C	2.29432	2.78896	-2.66331
C	2.4938	-0.41452	-2.97722	C	-2.19742	2.10286	2.72912	C	-1.76418	3.01355	2.04118
O	0.61722	2.23133	2.7363	O	3.5007	-0.53501	-0.0412	O	3.14877	-0.97991	-0.63305
O	0.68821	-0.75602	2.40652	O	2.17912	1.66241	-0.94946	O	1.50478	0.62748	-2.0586
O	0.4194	-1.53474	-3.65824	O	-3.70259	1.59602	0.85167	O	-3.66735	1.72941	1.15987
H	2.46638	1.06648	0.32193	H	1.30782	0.63021	1.95832	H	1.50476	1.09398	1.132
H	3.19493	2.23919	1.40723	H	2.83525	0.27682	2.77821	H	3.21799	1.08024	1.57186
H	-0.47408	2.50742	-1.51127	H	1.14421	-2.24014	-0.8996	H	0.80826	-2.77446	0.154
H	0.70495	1.28453	-1.04812	H	0.22486	-3.07282	0.32677	H	0.38399	-2.88051	1.84469
H	0.23948	-1.66534	0.19465	H	-0.21274	2.01478	-0.99433	H	-0.82355	0.98489	-1.52598
H	2.43681	-2.69603	-1.67067	H	-1.69639	3.88795	0.86569	H	-1.95839	3.59925	-0.51709
H	0.73362	-3.06566	-1.72191	H	-2.29256	3.0692	-0.54896	H	-2.81014	2.20515	-1.11506
H	-0.13923	0.49639	-3.29398	H	-2.94746	-0.29413	1.50838	H	-2.59493	0.38243	2.4279
H	3.89413	0.1699	2.25522	H	3.00543	2.5513	2.20252	H	3.04689	2.81703	0.0008
H	2.63874	0.72035	3.36213	H	4.03464	1.69792	1.06168	H	3.80526	1.48047	-0.85278
H	3.29048	-1.68758	0.44148	H	0.74637	3.52947	1.48384	H	0.59403	3.48656	-0.48622
H	-1.9865	1.60103	0.35168	H	0.21716	-0.08846	-0.28682	H	-0.10885	-0.56609	-0.06608
H	-0.90215	0.235	0.46264	H	-0.51391	-0.73573	1.17199	H	-0.28187	-0.36371	1.66553
H	-1.23237	-1.69155	-1.46339	H	-2.20315	0.74913	-1.2709	H	-2.72214	-0.16099	-0.57915
H	-2.07108	-1.20231	-2.90637	H	-3.52861	-0.35978	-1.0016	H	-3.83883	-0.88893	0.55172
H	-2.20359	1.12841	-2.1493	H	-2.06554	-2.0661	-0.06991	H	-2.03496	-1.9114	1.83639
H	-5.56432	0.16675	-1.13964	H	-0.41759	-1.42836	-3.95933	H	-2.73022	-4.84621	-0.19062
H	-4.50933	1.19525	-2.24476	H	0.09541	-0.27605	-2.62058	H	-2.90927	-4.02935	1.45553
H	-6.01363	-2.28143	1.92287	H	-3.77732	-4.05005	-4.31271	H	-1.59977	-2.05039	-3.98763
H	-4.63484	-3.22345	1.32097	H	-2.37074	-4.62496	-5.23003	H	-0.71682	-0.56412	-3.59357
H	-4.38811	-1.91384	2.53369	H	-2.62137	-5.22	-3.57664	H	0.10103	-2.12839	-3.39733
H	-0.81091	3.34086	1.85277	H	3.3249	-2.73632	-0.02408	H	3.18768	-2.9189	0.44155
H	2.22257	3.3769	-0.69555	H	0.76487	-1.47496	2.89896	H	1.49487	-0.29928	3.08079
H	0.87194	4.50736	-0.92087	H	1.08401	-3.20444	2.67585	H	1.90091	-1.94755	3.5922
H	1.78696	4.52947	0.57891	H	2.3734	-2.14336	3.21269	H	3.18167	-0.8303	3.15943
H	2.38954	-1.65694	4.20609	H	2.50046	4.27779	-1.12547	H	1.55363	2.85387	-3.46954
H	3.63861	-2.21655	3.07168	H	4.00837	3.53695	-0.62158	H	3.19568	2.3529	-3.1097
H	2.02794	-2.96363	3.09215	H	3.09092	4.53008	0.5317	H	2.5314	3.80543	-2.33071
H	3.23141	-1.11182	-3.38754	H	-2.76312	1.36799	3.31184	H	-2.09021	2.70551	3.04042
H	2.28372	0.33877	-3.74399	H	-1.14485	2.01847	3.01724	H	-0.66968	3.01721	2.03371
H	2.94285	0.09728	-2.12033	H	-2.55469	3.09825	3.01233	H	-2.11454	4.03793	1.87828
H	0.2283	-1.61336	2.41696	H	3.01056	1.14668	-0.97935	H	2.32521	0.09448	-2.02617
	14d3				14d4				14d5		
C	2.24141	-0.90613	1.50746	C	2.40456	-0.2592	1.36654	C	1.93009	-0.89326	1.9878
C	2.716	0.07465	0.40879	C	2.74306	0.85041	0.34322	C	2.74374	-0.19256	0.86994
C	2.63113	1.56644	0.68788	C	2.27293	2.26651	0.63289	C	2.72299	1.32884	0.81562
C	0.70407	-0.96521	1.62149	C	0.93046	-0.70655	1.27243	C	0.40306	-0.76843	1.81959
C	-0.69725	1.99691	-0.85298	C	-0.94653	1.92659	-1.19106	C	-0.50295	1.8989	-0.99466
C	-1.98724	2.34367	-0.16936	C	-2.32622	1.91555	-0.60251	C	-1.75559	2.49875	-0.42572
C	-2.10114	1.79969	1.23752	C	-2.40968	1.23378	0.74553	C	-2.05146	2.05713	0.99195
C	-2.33273	0.31771	1.54216	C	-2.27076	-0.28122	0.9277	C	-2.45422	0.62302	1.35528
C	2.84234	2.45041	-0.5497	C	2.3501	3.21073	-0.57525	C	3.07859	1.93913	-0.5458
C	1.69692	2.42173	-1.58946	C	1.32955	2.9256	-1.70299	C	1.96272	1.86378	-1.61107
C	0.37922	2.79674	-0.94031	C	-0.0856	2.95741	-1.15921	C	0.68476	2.51425	-1.11688
C	-0.00621	-1.61957	0.42232	C	0.56437	-1.43506	-0.03385	C	-0.17102	-1.51087	0.60137

C	-2.47793	-0.75056	0.4989	C	-2.0431	-1.25421	-0.18886	C	-2.60392	-0.49269	0.36581
C	-1.50343	-1.9395	0.68725	C	-0.80876	-2.16819	0.0093	C	-1.70916	-1.72527	0.64995
C	-1.95642	-3.13333	-0.15419	C	-0.87972	-3.28742	-1.02926	C	-2.10495	-2.87562	-0.27802
C	-2.36854	-4.27642	0.4268	C	-0.3673	-3.20292	-2.27179	C	-2.68274	-3.99149	0.20653
C	-1.92104	-2.99015	-1.64446	C	-1.53999	-4.58286	-0.67556	C	-1.82229	-2.72172	-1.74265
O	-2.30603	-4.13445	-2.26567	O	-2.27478	-4.44423	0.45653	O	-2.0811	-3.86962	-2.42029
O	-1.58301	-1.96351	-2.21353	O	-1.43777	-5.61048	-1.32946	O	-1.40519	-1.68913	-2.24755
C	-2.28364	-4.05607	-3.69116	C	-2.95687	-5.63361	0.85498	C	-1.80973	-3.79429	-3.8198
O	2.73768	-2.21575	1.20707	O	3.25096	-1.38838	1.12374	O	2.27137	-2.28669	2.01184
C	2.85538	-0.52506	2.85695	C	2.72572	0.20797	2.78797	C	2.35678	-0.33534	3.34911
C	2.00329	3.39674	-2.72967	C	1.47729	3.96404	-2.81779	C	2.42744	2.54596	-2.90182
C	-1.54678	2.66659	2.32889	C	-2.19538	2.11052	1.94355	C	-1.51525	2.93659	2.08211
O	3.18816	-0.35894	-0.6493	O	3.4075	0.58355	-0.66587	O	3.47384	-0.87174	0.13995
O	1.57433	1.13124	-2.17865	O	1.57012	1.64942	-2.29252	O	1.73157	0.48978	-1.89692
O	-3.40415	1.27783	1.53313	O	-3.55039	0.37352	0.86617	O	-3.42036	1.67489	1.1874
H	1.6773	1.79413	1.16923	H	1.25833	2.23867	1.03748	H	1.75624	1.69876	1.16358
H	3.42566	1.79941	1.40638	H	2.92886	2.66353	1.41644	H	3.46999	1.66511	1.54547
H	0.45293	-1.55456	2.51337	H	0.72959	-1.3955	2.10336	H	-0.0715	-1.18748	2.71673
H	0.30911	0.0412	1.78325	H	0.27943	0.16036	1.40931	H	0.1252	0.28663	1.77418
H	-0.66685	1.01754	-1.32792	H	-0.647	1.0122	-1.6967	H	-0.60256	0.86847	-1.32336
H	-2.11328	3.43381	-0.14326	H	-2.7012	2.94298	-0.51035	H	-1.69437	3.59406	-0.45783
H	-2.81301	1.97742	-0.79262	H	-2.99857	1.42929	-1.32092	H	-2.58966	2.23781	-1.08963
H	-2.07653	-0.07653	2.51768	H	-2.01004	-0.68741	1.8974	H	-2.32862	0.26865	2.37096
H	2.9841	3.48228	-0.2009	H	2.2032	4.23554	-0.20838	H	3.34367	2.99224	-0.38136
H	3.78056	2.15344	-1.03693	H	3.36675	3.16952	-0.98823	H	3.97955	1.44348	-0.93023
H	0.33465	3.78701	-0.49084	H	-0.39396	3.88611	-0.68239	H	0.77252	3.55348	-0.8038
H	0.50939	-2.55952	0.18897	H	1.34085	-2.18509	-0.22992	H	0.30341	-2.49895	0.5444
H	0.1035	-0.9819	-0.46	H	0.59726	-0.72939	-0.86959	H	0.12018	-0.98594	-0.31082
H	-2.38556	-0.33219	-0.50562	H	-1.97867	-0.7495	-1.1573	H	-2.46488	-0.13185	-0.65462
H	-3.50594	-1.13169	0.57071	H	-2.94198	-1.88307	-0.24705	H	-3.65208	-0.82033	0.41381
H	-1.57901	-2.24552	1.74093	H	-0.86936	-2.62508	1.00559	H	-1.93976	-2.04216	1.67795
H	-2.69361	-5.14005	-0.14577	H	-0.43389	-4.02942	-2.97591	H	-2.97249	-4.82385	-0.42786
H	-2.39237	-4.38533	1.50665	H	0.12737	-2.30668	-2.63151	H	-2.8837	-4.1086	1.26702
H	-1.26872	-3.856	-4.0489	H	-2.24076	-6.43282	1.07052	H	-0.75067	-3.58007	-3.99468
H	-2.60571	-5.02265	-4.08837	H	-3.51475	-5.41345	1.76935	H	-2.04357	-4.76621	-4.263
H	-2.97891	-3.288	-4.0443	H	-3.66725	-5.94567	0.08298	H	-2.44262	-3.03687	-4.29283
H	2.8882	-2.23666	0.23897	H	3.52365	-1.33416	0.18446	H	2.79946	-2.44459	1.19947
H	2.45957	0.42229	3.23704	H	2.64313	-0.62068	3.5008	H	2.0537	0.70855	3.47956
H	2.6655	-1.30131	3.6073	H	3.75738	0.57331	2.85301	H	1.92512	-0.92348	4.16722
H	3.94465	-0.43041	2.77556	H	2.0596	1.01131	3.11882	H	3.44553	-0.38923	3.46718
H	1.21431	3.37296	-3.49113	H	0.77905	3.76229	-3.63909	H	1.66182	2.47492	-3.68377
H	2.93196	3.11611	-3.24028	H	2.48289	3.92521	-3.25265	H	3.32161	2.05331	-3.30111
H	2.10468	4.42721	-2.37202	H	1.29736	4.98219	-2.45586	H	2.65752	3.60524	-2.74367
H	-1.72699	2.2382	3.3207	H	-2.32541	1.55901	2.88085	H	-1.80312	2.57635	3.07552
H	-0.46581	2.78728	2.20964	H	-1.18215	2.52392	1.93765	H	-0.42199	2.96916	2.04245
H	-2.00913	3.65873	2.30556	H	-2.90716	2.9424	1.9446	H	-1.89465	3.95793	1.9745
H	2.40095	0.62759	-2.04121	H	2.50368	1.3988	-2.14131	H	1.12371	0.43314	-2.65451

Detailed DP4+ probability for compound **14**. Isomer 1 is 1S*,3R*,4R*,8R*,12R*, isomer 2 is 1S*,3R*,4R*,8S*,12R*, isomer 3 is 1S*,3S*,4R*,8S*,12R*, isomer 4 is 1S*,3S*,4R*,8S*,12R*.

Functional	Solvent?	Basis Set	Type of Data
B3LYP	PCM	6-311+G(d,p)	Unscaled Shifts

	Isomer 1	Isomer 2	Isomer 3	Isomer 4	Isomer 5	Isomer 6
sDP4+ (H data)	100.00%	0.00%	0.00%	0.00%	-	-
sDP4+ (C data)	25.27%	74.73%	0.00%	0.00%	-	-
sDP4+ (all data)	100.00%	0.00%	0.00%	0.00%	-	-
uDP4+ (H data)	100.00%	0.00%	0.00%	0.00%	-	-
uDP4+ (C data)	90.57%	9.43%	0.00%	0.00%	-	-
uDP4+ (all data)	100.00%	0.00%	0.00%	0.00%	-	-
DP4+ (H data)	100.00%	0.00%	0.00%	0.00%	-	-
DP4+ (C data)	76.46%	23.54%	0.00%	0.00%	-	-
DP4+ (all data)	100.00%	0.00%	0.00%	0.00%	-	-
